# *Trans*-Anethole Alleviates Subclinical Necro-Haemorrhagic Enteritis-Induced Intestinal Barrier Dysfunction and Intestinal Inflammation in Broilers

**DOI:** 10.3389/fmicb.2022.831882

**Published:** 2022-03-21

**Authors:** Caiyun Yu, Dong Wang, Yichun Tong, Qiming Li, Weiren Yang, Tian Wang, Zaibin Yang

**Affiliations:** ^1^College of Animal Sciences and Technology, Nanjing Agricultural University, Nanjing, China; ^2^Heilongjiang Key Laboratory of Experimental Animals and Comparative Medicine, College of Veterinary Medicine, Northeast Agricultural University, Harbin, China; ^3^College of Animal Sciences and Technology, Shandong Agricultural University, Tai’an, China

**Keywords:** *Trans*-anethole, broiler, subclinical necro-haemorrhagic enteritis, intestinal inflammation, gut microbiota

## Abstract

This study investigated the alleviative potential of *trans*-anethole (TA) on the impaired intestinal barrier and intestinal inflammation and its regulatory effects on gut microbiota in broilers with subclinical necro-hemorrhagic enteritis (NE) challenge. Subclinical NE challenge led to a severe decline in the 21-day body weight (BW) and average daily gain (ADG), but an increase in feed conversion ratio (FCR) and intestinal lesion score of birds compared with controls (*P* < 0.05). Compared with the subclinical NE group, the TA administration group exhibited lower (*P* < 0.05) intestinal lesion score and crypt depth (CD), serum diamine oxidase activity, and D-lactate concentration, but higher (*P* < 0.05) intestinal tight junction protein expressions, villus height (VH), VH/CD, and numbers of proliferating cell nuclear antigen (PCNA)-positive cells. The administration of TA also inhibited (*P* < 0.05) the expression of intestinal pro-inflammatory cytokines including interleukin (IL)-1β, IL-8, interferon-gamma (IFN-γ), and tumor necrosis factor-alpha (TNF-α) but increased (*P* < 0.05) jejunal IL-10 and secretory immunoglobulin A (sIgA) concentration. TA inclusion also led to a remarkable reduction of intestinal NF-kappa-B inhibitor alpha (IκBα) degradation and nuclear factor kappa beta (NF-κB) translocation. Moreover, TA modulated the cecal microbiota abundance and diversity of NE birds, as confirmed by reducing the phylum Firmicutes and genera *Ruminococcaceae_UCG-014*, *Eubacterium*_*coprostanoligenes_group*, and *Ruminococcaceae_NK4A214_group* when supplemented at 600 mg/kg and reducing genera *Butyricicoccus*, *Oscillibacter*, and *Flavonifractor* when supplemented at 400 mg/kg (*P* < 0.05). Supplementation of TA in broiler diets could alleviate subclinical NE infection by restoring intestinal barrier integrity, inhibiting NF-κB signaling pathway, and modulating gut microbiota. A 600-mg/kg dose of TA is the optimum concentration for ameliorating subclinical NE in broilers.

## Introduction

Necro-hemorrhagic enteritis (NE), an intestinal bacterial disease in poultry mainly caused by *Clostridium perfringens* infection, has increased in incidence with the banning of antibiotics in feed (McDevitt et al., 2006). NE could result in 1% of flock mortality per day and cost approximately 5–6 billion dollars annually worldwide (Wade and Keyburn, 2015). Ongoing research into the pathology, attenuation, and prevention of NE has been intensified. Subclinical NE results in intestinal lesion, thereby anorexia, dyspepsia, and finally poor growth performance with low mortality rates in broilers (Emami and Dalloul, 2021; Kan et al., 2021). It is well known that NE cannot be experimentally induced by inoculation with *C. perfringens* alone (Shojadoost et al., 2012). Dierick et al. (2021) indicated that coccidiosis is the most important predisposing factor for NE incidence and severity, and *Eimeria* is the most currently used in establishing subclinical NE model. Taken together, we established an *Eimeria*/*C. perfringens* type A strain co-infection model to induce subclinical NE in broilers in this study. Currently, the use of antibiotics and ionophore coccidiostats with antibiotic effect is the most effective strategy to control and prevent NE in poultry. However, due to great concerns for antibiotic resistance and its potential negative impact on the environment and human health, prohibition of antibiotics in feed has accelerated the research for seeking natural dietary alternatives to prevent NE occurrence in the post-antibiotic era. More specifically, phytogenic substances with many biological activities have gained increasing interest with respect to their potential to alleviate infectious diseases in poultry (Veldhuizen et al., 2006; Burt et al., 2007).

*Trans*-anethole (TA), 1-methoxy-4-(1-propenyl) benzene, is the main constituent of the essential oils of fennel (*Foeniculum vulgare*) and star anise (*Illicium verum* and *Pimpinella anisum*) and seems to be responsible for the bioactivities of these oils (Ritter et al., 2013). TA has been generally recognized as safe by the United States Food and Drug Administration (FDA). It is a flavoring agent that is widely used in food, pharmaceutical, and cosmetic industries (Soares et al., 2007; Domiciano et al., 2013). Many trials conducted in animals and cell line demonstrated the pharmacological activities of TA, such as antimicrobial (Huang et al., 2010; Kwiatkowski et al., 2019), antioxidant (Sá et al., 2018; Wieczyńska and Cavoski, 2018; Chaudhari et al., 2020), and anti-inflammatory properties (Cho et al., 2013; da Rocha et al., 2017; Kim et al., 2017). An earlier study showed that TA decreased the survival of invasive *Eimeria acervulina* sporozoites, indicating the underlying inhibitory effects of TA on avian coccidiosis (Cho et al., 2013). Furthermore, dietary supplementation of essential oils containing star anise inhibited *C. perfringens* and *E.coli* proliferation in broilers (Cho et al., 2014). These widely reported preclinical pharmacologic activities, particularly anti-inflammatory property, provide evidence that TA may protect broilers against infectious diseases. Therefore, the objective of this study is to investigate the alleviative potential of TA on subclinical NE infection and the mechanism of action by determining intestinal barrier function, intestinal morphology, inflammatory response, and intestinal microbiota.

## Materials and Methods

### Ethics Approval

All procedures in the animal experiments were reviewed and approved by the Institutional Animal Care and Use Committee of Nanjing Agricultural University (Permit No. SYXK-2017-0027).

### Animals, Diets, and Experimental Design

A total of 256 1-day-old male Arbor Acres broilers with similar body weight (42.43 ± 0.88 g) were purchased from Yantai Land Animal Husbandry (Shandong, China) and then randomly divided into four groups (eight replicates per group, 32 cages) of eight birds each. Each replicate was reared in a separate cage (90 × 70 × 70 cm^3^). This trial lasted for 21 days. The four groups were as follows: CON group (basal diet), NE group (basal diet + subclinical NE challenge), NE + TA400 group (basal diet extra 400 mg/kg of TA + subclinical NE challenge), and NE + TA600 group (basal diet extra 600 mg/kg of TA + subclinical NE challenge). TA (purity 98.35%, Nanjing Dilger Medical Technology, Nanjing, China) was stored in glass bottles in the dark at 4°C until use. The antibiotic-free and coccidiostat-free corn-soybean meal basal diet (Supplementary Table 1) was prepared according to NY/T 33-2004. TA was firstly mixed with soybean oil and then mixed with other ingredients. All of the diets were pelleted and crumbled, and birds received food and water *ad libitum* throughout the entire trial period. The temperature was gradually reduced from 35 to 22°C by 0.5°C per day, and the relative humidity was maintained at 65%. Artificial light (10–20 lux) was provided in a 23-h light/1-h dark program throughout the entire experimental period. The body weight (BW) and feed intake of birds of each replicate were recorded weekly to calculate the average daily feed intake (ADFI), average daily gain (ADG), and feed conversion ratio (FCR).

### Subclinical Necrotic-Hemorrhagic Enteritis Challenge

An attenuated live coccidiosis vaccine (Foshan Standard Biotech, Guangdong, China) providing oocysts containing a mixture of *Eimeria acervulina*, *Eimeria maxima*, *Eimeria necatrix*, and *Eimeria tenella* and *Clostridium perfringens* type A strain (125404; BeNa Culture Collection, Xinyang, China) was used for co-infection in this study. Freeze-dried powder of *C. perfringens* was cultured anaerobically with fluid thioglycolate (FT) medium (HB5190; Hopebio Biotech, Qingdao, China) in panel for 24 h at 37°C; then, a single colony was aseptically transferred into 2-L conical flask with FT medium and incubated anaerobically for 13 h at 37°C. The procedure of subclinical NE challenge was performed referring to Liu et al. (2010) and Zhang et al. (2017) with little modifications. Briefly, 10-day-old chickens in the infected groups were each orally gavaged with live coccidiosis vaccine suspended in 500 μl of normal saline with coccidia suspension agent and subsequently gavaged with 2 ml of *C. perfringens* type A solution (3 × 10^8^ CFU/ml) once a day during days 14–19. Birds in the CON group were orally gavaged with same amount of normal saline and sterile FT medium solution at the corresponding times.

### Sample Collection and Intestinal Lesion Score

At 22 days of age, eight birds (one bird per replicate) per group with similar weight were selected for sampling. Peripheral blood samples from the wing vein were collected into tubes coated with coagulant and centrifuged at 3,500 × *g* for 10 min at 4°C for withdrawal of serum samples, then stored at −80°C until analysis. After blood sampling, the birds were stunned and subsequently killed by cervical dislocation. Approximately 1 cm of the middle segments of the jejunum and ileum were cut off carefully and fixed in 4% paraformaldehyde solution for histomorphology analysis. Subsequently, about 3 cm of jejunal and ileal middle segments were dissected longitudinally, flushed with ice-cold normal saline to remove contents, rapidly frozen in liquid nitrogen, and stored at −80°C until analysis of mRNA and protein expression. Meanwhile, the cecal content was collected into a sterile freezing tube, snap-frozen in liquid nitrogen, and later stored at −80°C until microbiota analysis. The jejunum and ileum of each bird were opened lengthwise and washed with ice-cold physiologic saline. The macroscopic intestinal lesions were scored 0 (none) to 4 (severe) by an observer blind to the treatments according to the following scoring criteria: 0 = no obvious lesions; 1 = thin and friable intestine with 1–5 hemorrhagic spots; 2 = small gas production and focal necrosis (hemorrhagic spots 6–15); 3 = gas-filled intestine and bleeding ulcers; 4 = severe ulceration, mucosal edema, and diffuse necrosis with great amounts of gas in the intestine (Deshmukh, 2010; Gholamiandehkordi et al., 2010).

### Hematoxylin–Eosin Staining

The fixed jejunum and ileum segments were dehydrated in graded ethanol, transparentized with xylol, and embedded in paraffin. Then, each sample was sliced into 5-μm cross sections, deparaffinized in xylene, subjected to graded rehydration, and finally stained with hematoxylin–eosin for morphology measurement. Ten well-oriented villi and crypts per sample were selected for measuring the villus height (VH) and crypt depth (CD) with a light microscope (Olympus CX31, Tokyo, Japan) and Image-Pro Plus 6.0 software (Media Cybernetics, Inc., Rockville, MD, United States).

### Measurement of Serum Diamine Oxidase and D-Lactate

Serum diamine oxidase (DAO) activity was measured by a colorimetric assay kit (A008-1; Nanjing Jiancheng Bioengineering Institute, Nanjing, China) according to the manufacturer’s instructions. Serum D-lactate was determined using a colorimetric assay kit (E-BC-K002-M; Elabscience Biotechnology, Wuhan, China).

### Measurement of Cytokines and Secretory Immunoglobulin A

The frozen jejunum and ileum tissues were homogenized with 0.9% physiological saline in the ratio of 1:9 (wt/vol) and then centrifuged at 5,000 × *g* for 10 min at 4°C. The supernatants were used to quantify protein levels of interleukin (IL)-1β, IL-8, IL-10, tumor necrosis factor-alpha (TNF-α), interferon-gamma (IFN-γ), and secretory immunoglobulin A (sIgA) using ELISA kits (Hongyi Biotechnology, Nanjing, China) following manufacturer’s instructions. The total protein concentration of intestinal tissues was assayed using bicinchoninic acid protein assay kit (P0010; Beyotime Biotechnology, Nanjing, China). The results were expressed as the concentration of cytokines or sIgA per microgram of protein in the intestinal tissues of broilers. Additionally, the concentrations of IL-1β, IL-10, TNF-α, and IFN-γ in the serum were also detected using the same ELISA kits.

### Measurement of Jejunal and Ileal Apoptosis and Proliferation

A TUNEL Cell Apoptosis Detection Kit (G1501; Servicebio technology, Wuhan, China) was used to determine the apoptotic index of the jejunum and ileum. Briefly, the sections were firstly subjected to deparaffinization and graded rehydration; then, proteinase K (20 μg/ml) was added and incubated at 37°C, followed by permeabilization and equilibrium at room temperature. Later, the slices were dipped in TUNEL mixture and incubated at 37°C. Finally, the slices were counterstained with the 4,6-diamidino-2-phenylindole (DAPI) solution. The images were collected through a fluorescence microscope (Nikon DS-U3, Nikon, Tokyo, Japan). The ratio of positive apoptosis cells to total cells was quantified from 10 full-length villi each slice using Image-Pro Plus 6.0 software.

Immunohistochemical staining was performed to determine the intestinal expression of proliferating cell nuclear antigen (PCNA). After deparaffinizing with xylene and rehydrating through graded alcohols, the tissue sections were placed in a repair box filled with citric acid (pH 6.0) buffer and heated in a microwave oven for 20 min. Then, the sections were later dipped in 3% hydrogen peroxide. The sections were incubated with 3% bovine serum albumin (BSA) and sealed for 30 min, followed by PCNA primary antibody (ab29; Abcam, Cambridge, MA, United States) incubation overnight at 4°C. Subsequently, the sections were washed with PBS and incubated with secondary antibody (G1213 Goat anti-mouse IgG, Servicebio Technology, Wuhan, China) labeled with horseradish peroxidase at room temperature for 50 min. The DAB color developing time was controlled under a microscope (Nikon E100, Nikon, Tokyo, Japan). The sections were counterstained with hematoxylin stain solution, dehydrated in graded alcohols, cleared by xylene, and then mounted with neutral gum. Each section was captured with a microscope (Nikon DS-U3, Nikon, Tokyo, Japan), and PCNA-positive cells from 10 different randomly selected intact crypts were counted regardless of the staining intensity using Image-Pro Plus 6.0 software.

### Total RNA Extraction and Quantitative Real-Time PCR

Total RNA from each jejunal and ileal tissue was isolated by TRIzol reagent (9108; TaKaRa Biotechnology, Dalian, Liaoning, China). The concentration and purity of extracted RNA were measured by a microspectrophotometer (NanoDrop-1000, Thermo Fisher Scientific, Waltham, MA, United States), and the integrity of RNA was determined with 2.0% agarose gel electrophoresis. Then, PrimeScript™ RT reagent Kit (RR036A; TaKaRa Biotechnology) was used to produce the complementary DNA. A qRT-PCR procedure was carried out to determine the mRNA abundance of *occludin* (*OCLN*), *zonula occludens-1* (*ZO-1*), *claudin-1* (*CLDN-1*), *B-cell lymphoma/leukemia 2* (*Bcl2*), *Bcl2-associated X* (*Bax*), *caspase-3*, *IL-1*β, *IL-2*, *IL-4*, *IL-8*, *IL-10*, *TNF-*α, *IFN-*γ, *inducible nitric oxide synthase* (*iNOS*), *nuclear factor kappa beta* (*NF-*κ*B*), *NF-kappa-B inhibitor alpha* (*I*κ*B*α), and *beta-actin* (β*-actin*) using ChamQ SYBR ^®^ qPCR Master Mix Kit (Q311-02; Vazyme Biotechnology, Nanjing, China) based on Applied Biosystems 7500 Real-time PCR System (Life Technologies, CA, United States). The relative mRNA abundance of the target genes was calculated using the 2^–ΔΔCt^ method and normalized with expression level of endogenous reference gene (β*-actin*). The qRT-PCR primers for target genes were commercially synthesized by Sangon Biotechnology (Shanghai, China) and listed in Supplementary Table 2.

### Western Blot Analysis for Protein Expressions

Total protein was extracted using radioimmunoprecipitation assay lysis buffer and protease inhibitor (P0013B and ST506; Beyotime Biotechnology). Nuclear protein was isolated by Nuclear and Cytoplasmic Protein Extraction Kit (P0027; Beyotime Biotechnology) for detection of p65 NF-κB protein expression. The concentration of total protein and nuclear protein was assayed using bicinchoninic acid protein assay kit (P0010; Beyotime Biotechnology). Equal amounts of denatured protein were separated on 10% SDS-PAGE gel and then electrotransferred onto polyvinylidene difluoride (PVDF) membranes. Thereafter, the PVDF membranes were incubated with blocking buffer (5% skimmed milk in Tris-buffered saline containing 1% Tween-20) for 2 h, followed by incubation with the primary antibody against OCLN (DF7504; Affinity Biosciences, Jiangsu, China), ZO-1 (DF2250; Affinity), CLDN-1 (AF0127; Affinity), NF-κB (10745-1-AP; Proteintech Group, Inc., Wuhan, China), IκBα (10268-1-AP; Proteintech), and β-actin (20536-1-AP; Proteintech) overnight at 4°C and then incubation with secondary antibody for 1.5 h at room temperature. Ultimately, the expression of target proteins was detected with ECL reagents (E412-01; Vazyme Biotechnology) and Imager-Bio-Rad (Bio-Rad Laboratories, Inc., Hercules, CA, United States), then quantified by ImageJ software.

### 16S rDNA Sequencing

Extraction of total bacterial DNA from cecal contents was performed by using HiPure Stool DNA kits (D3141, Magen Biotech, Guangzhou, China) according to manufacturer’s protocols. The bacterial 16S rDNA V3–V4 region was amplified by PCR procedure using barcoded primers 341F (5′-CCTACGGGNGGCWGCAG − 3′) and 806R (5′-GGACT ACHVGGGTATCTAAT-3′). The amplicons were extracted from 2% agarose gels and purified using the AxyPrep DNA Gel Extraction Kit (AP-GX-250, Axygen Biosciences, Union City, CA, United States). Then, the purified amplicons were pooled equimolarly and paired-end sequenced (2 × 250 bp) on an Illumina HiSeq 2500 platform (Gene *Denovo* Biotechnology, Guangzhou, China) according to the standard protocols.

The sequencing raw data were firstly filtered to get high-quality clean reads using FASTP. Then, the paired-end clean reads were merged as raw tags using FLASH (version 1.2.11). Raw tags were then filtered by QIIME (version 1.9.1) pipeline and chimeric tags removed using UCHIME (version 8.1) algorithm to obtain effective tags. UPARSE (version 9.2.64) was used to cluster effective tags into operational taxonomic units (OTUs) of ≥ 97% similarity. Venn analysis between groups was performed in R project VennDiagram package (version 1.6.16) to identify unique and shared OTUs. Taxonomy annotation was performed to classify OTU sequences into organisms by an RDP classifier (version 2.2) based on SILVA database (version 132) with the confidence threshold value of 0.8. Alpha diversity, including observed species, Chao 1, and Shannon index, was calculated by QIIME (version 1.9.1), and the significance of these indices among groups was investigated by Tukey’s HSD test. Multivariate statistical techniques including principal component analysis (PCoA) and non-metric multi-dimensional scaling (NMDS) based on weighted UniFrac analysis in R software (version 2.5.3) were performed to investigate beta diversity between groups. Furthermore, non-parametric MANOVA (Adonis) for PCoA and the analysis of similarities (Anosim) test for NMDS were also calculated.

### Statistical Analysis

All data not specifically mentioned were analyzed by using one-way ANOVA and Tukey’s HSD test of SAS (SAS Institute, 2001). Shapiro–Wilk test was used to determine the dataset normality and homogeneity of variances. The results were presented as means with their standard errors. Statistically significant differences were recognized as *P* < 0.05, and 0.05 < *P* < 0.10 was considered as a tendency to be significantly different.

## Results

### Growth Performance

No mortality was observed in the groups during the experimental period. NE infection led to a severe decline in the final BW and ADG, but an increase in FCR at days 10–21 and days 1–21 compared with controls (Table 1, *P* < 0.05). TA administration at 600 mg/kg reversely improved the growth performance of NE birds (*P* < 0.05). No remarkable difference was found with regard to initial BW and ADFI at days 0–9, 10–21, and 1–21 among groups (*P* > 0.05). Moreover, the ADG and FCR were not altered by TA supplementation before NE infection (*P* > 0.05).

**TABLE 1:** The effect of dietary TA supplementation on the growth performance of broilers challenged with subclinical necro-hemorrhagic enteritis^1^.

Items^2^	CON	NE	NE + TA400	NE + TA600	*P*-value
Initial BW, kg	42.33 ± 0.86	42.00 ± 0.50	42.67 ± 1.12	42.70 ± 0.78	0.319
Final BW, kg	873.13 ± 22.62^a^	794.05 ± 22.53^c^	828.12 ± 48.42^bc^	840.14 ± 36.14^ab^	0.001
**Pre-challenge (0–9 days)**					
ADFI, g/day	27.79 ± 1.20	28.09 ± 0.92	27.71 ± 0.63	27.81 ± 1.21	0.884
ADG, g/day	23.07 ± 0.66	23.31 ± 0.58	23.27 ± 1.25	23.23 ± 1.45	0.973
F/G, g/g	1.20 ± 0.03	1.21 ± 0.05	1.19 ± 0.04	1.20 ± 0.07	0.957
**Post-challenge (10–21 days)**					
ADFI, g/day	67.76 ± 1.73	65.51 ± 2.26	66.29 ± 4.49	67.47 ± 2.61	0.409
ADG, g/day	56.19 ± 1.75^a^	49.18 ± 2.06^b^	52.11 ± 4.57^ab^	53.19 ± 2.81^a^	0.008
F/G, g/g	1.21 ± 0.02^b^	1.34 ± 0.09^a^	1.28 ± 0.14^ab^	1.27 ± 0.07^ab^	0.064
**Overall (1–21 days)**					
ADFI, g/day	50.36 ± 2.99	49.54 ± 2.81	48.14 ± 2.54	50.17 ± 2.26	0.351
ADG, g/day	40.53 ± 1.10^a^	36.69 ± 1.12^b^	38.31 ± 2.34^b^	38.90 ± 1.79^a^	0.001
F/G, g/g	1.24 ± 0.06^b^	1.35 ± 0.10^a^	1.26 ± 0.08^ab^	1.29 ± 0.06^ab^	0.045

*^a–c^Means within a row with different letters differ significantly (P < 0.05).*

*^1^Data are means for eight replicates of six birds per replicate. No birds died during the experimental period. The data in each group was expressed as mean with their standard errors (n = 8).*

*^2^ADFI, average daily feed intake; ADG, average daily gain; FCR, feed conversion ratio; BW, body weight.*

### Intestinal Lesion Score and Morphology

**FIGURE 1:**
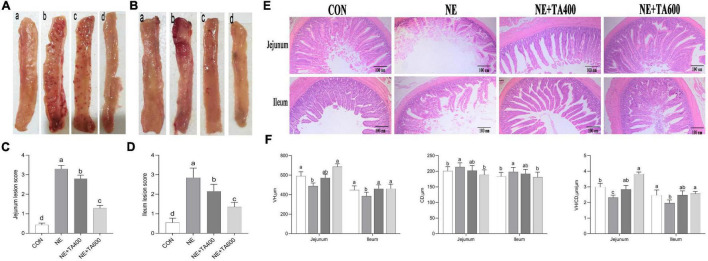
The effects of TA on intestinal macroscopic changes and lesion score and the morphology of subclinical NE broilers. **(A)** Images of jejunal tissues; **(B)** images of ileal tissues; **(C)** jejunum lesion score; **(D)** ileum lesion score; **(E)** hematoxylin and eosin staining; **(F)** VH, CD, and VH/CD of intestine. Values were expressed as mean with standard error represented by vertical bars. ^a–c^Means with different letters differ significantly among the groups (*P* < 0.05). VH, villus height; CD, crypt depth; TA, *trans*-anethole; NE, necro-hemorrhagic enteritis.

The macroscopic assessment of the jejunum and ileum in the CON group revealed no epithelium damage, whereas subclinical NE challenge led to severe mucosal injury, intestinal wall thinning, focal necrosis, and multiple hemorrhagic spots. In contrast, TA administration ameliorated the macroscopic lesions compared with the subclinical NE group (Figure 1). Furthermore, subclinical NE infection increased the macroscopic damage score in comparison with the control group (Figure 1, *P* < 0.05). However, the inclusion of TA produced remarkable reduction of the intestinal lesion score compared with the subclinical NE group (*P* < 0.05). A 600-mg/kg concentration of TA showed better ameliorative effects than 400 mg/kg of TA (*P* < 0.05).

There was some damage to jejunal and ileal villi development after NE infection, as found by broken and shortened villi (Figure 1). Consistent with the histological observations, NE infection significantly reduced the VH and VH/CD in jejunal and ileal tissues, but increased the CD as compared with the control (Figure 1), which was reversed by 600 mg/kg of TA supplementation (*P* < 0.05).

### Intestinal Barrier Integrity

**FIGURE 2:**
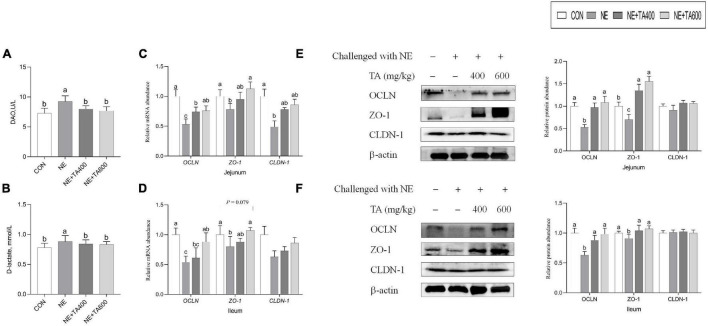
The effects of TA on the intestinal barrier function of subclinical NE broilers. **(A)** Serum DAO activity; **(B)** serum D-lactate concentration; **(C,D)** the mRNA abundance of jejunal and ileal *OCLN*, *ZO-1*, and *CLDN-1*; **(E)** the protein levels of jejunal OCLN, ZO-1, and CLDN-1; **(F)** the protein levels of ileal OCLN, ZO-1, and CLDN-1. Values were presented as mean with standard error represented by vertical bars. ^a,b^Means with different letters differ significantly among the groups (*P* < 0.05). OCLN, occludin; ZO-1, zonula occludens-1; CLDN-1, claudin-1.

Compared with the CON group, serum DAO activity and D-lactate concentration were both distinctly increased by NE challenge, but they were reversely decreased after administration of TA (Figure 2, *P* < 0.05). qRT-PCR analysis found that compared with the broilers in the CON group, NE-challenged broilers had lower mRNA levels of jejunal and ileal *OCLN* and jejunal *ZO-1* and *CLDN-1* (*P* < 0.05), but had no remarkable difference in ileal *CLDN-1* expression (*P* > 0.05). Besides, a tendency was detected that the ileal mRNA expression of *ZO-1* of the NE group was lower than that of the CON group and the TA supplementation group (*P* = 0.079). However, these adverse alterations of NE infection on mRNA abundance of tight junction proteins were abolished by TA supplementation (Figure 2). Western blot analysis revealed that TA administration could partially increase (*P* < 0.05) jejunal and ileal OCLN and ZO-1 expressions but had no effect on CLDN-1 expression at translational level compared with the NE group (Figure 2).

### Intestinal Cell Apoptosis and Proliferation

**FIGURE 3:**
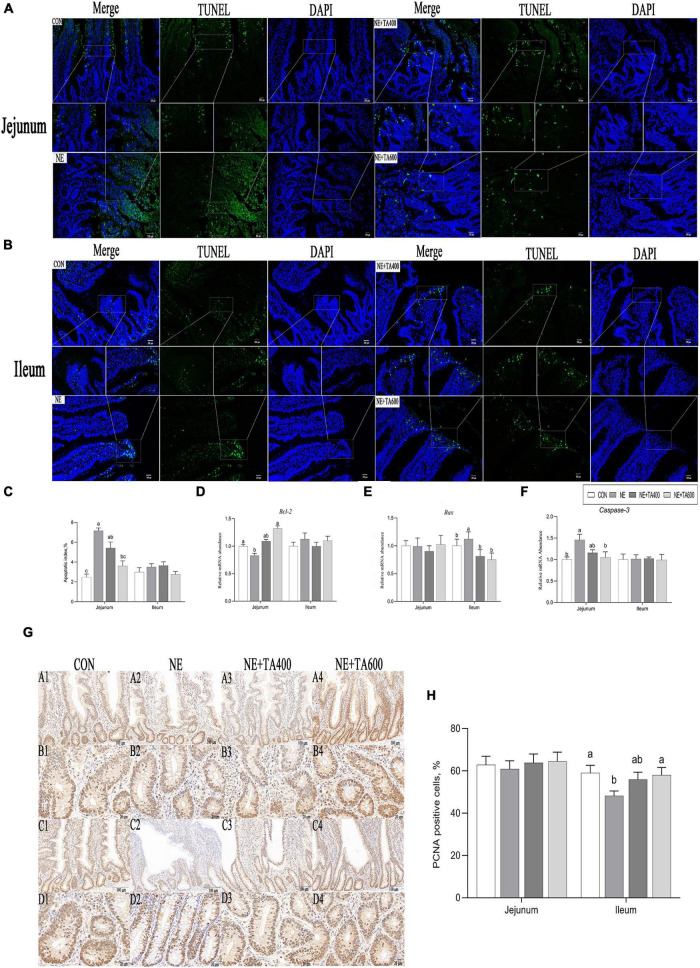
Effects of TA on intestinal apoptosis and proliferation of subclinical NE broilers. **(A–C)** Jejunal and ileal TUNEL apoptosis and apoptotic index, **(D–F)** jejunal and ileal mRNA abundance of apoptotic genes, **(G,H)** jejunal and ileal proliferating cell nuclear antigen (PCNA) staining and numbers of PCNA-positive cells (PCNA-positive cells/total numbers of cells ×100). Values were expressed as mean with standard error represented by vertical bars. ^a,b^Means with different letters differ significantly among the groups (*P* < 0.05). *Bcl2*, B-cell lymphoma/leukemia 2; *Bax*, Bcl2-associated X; PCNA, proliferating cell nuclear antigen.

Compared with the CON group, NE infection increased (*P* < 0.05) the number of jejunal TUNEL-positive cells and apoptotic index and mRNA expressions of ileal Bax and jejunal caspase-3, while it reduced the mRNA levels of jejunal Bcl2, indicating remarkable activation of jejunal apoptosis. These negative alterations were abrogated by TA administration at 600 mg/kg (Figure 3, *P* < 0.05). There was no significant difference in ileal apoptotic index, mRNA levels of Bcl-2 and caspase-3, and jejunal *Bax* among treatment groups (Figure 3, *P* > 0.05).

Compared with the CON group, NE infection reduced the numbers of PCNA-positive cells in the ileum (Figure 3), indicating remarkable inhibition of proliferation following NE infection. This negative alteration was abrogated by TA administration at 600 mg/kg (*P* < 0.05).

### Expression of Inflammatory Cytokines

**FIGURE 4:**
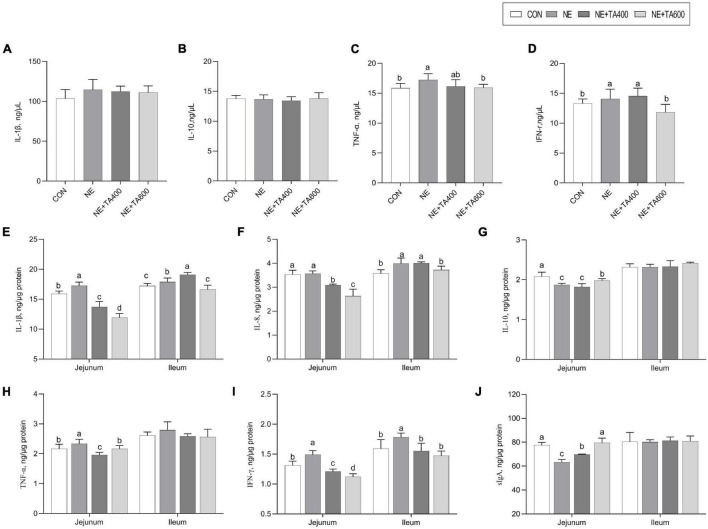
The effects of TA on the concentration of inflammatory cytokines and sIgA of subclinical NE broilers. **(A–D)** The serum concentration of IL-1β, IL-10, TNF-α, and IFN-γ; **(E–I)** jejunal and ileal concentration of IL-1β, IL-8, IL-10, TNF-α, and IFN-γ; **(J)** Jejunal and ileal sIgA concentration. Values were expressed as mean with standard error represented by vertical bars. ^a,b^Means with different letters differ significantly among the groups (*P* < 0.05). IL-1β, interleukin-1β; IL-8, interleukin-8; IL-10, interleukin-10; TNF-α, tumor necrosis factor-α; IFN-γ, interferon-γ; sIgA, secretory immunoglobulin A.

To address whether TA supplementation could ameliorate gut inflammation caused by subclinical NE infection, we determined the protein and mRNA levels of inflammatory cytokines and sIgA concentration in the jejunum and ileum. We found that the serum concentrations of TNF-α and IFN-γ were significantly increased after NE infection when compared to the CON group, although a similar IL-1β concentration was observed (Figure 4). The jejunal and ileal IL-1β and IFN-γ, jejunal TNF-α, and ileal IL-8 concentrations in the NE group were also elevated (*P* < 0.05) in comparison with the CON group. As expected, the administration of TA, especially at 600 mg/kg, exhibited remarkable suppressive effects on the levels of jejunal and ileal IL-1β and IFN-γ, jejunal TNF-α, and ileal IL-8 (*P* < 0.05). In addition, jejunal IL-10 and sIgA concentrations were down-regulated after NE infection as compared with the CON group, which could be increased by 600 mg/kg of TA supplementation (Figure 4, *P* < 0.05).

**FIGURE 5:**
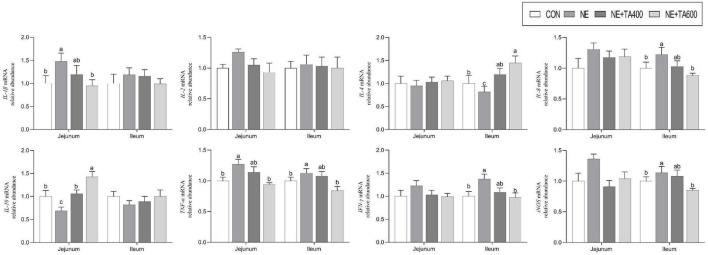
The effects of TA on mRNA abundance of jejunal and ileal inflammatory cytokines of subclinical NE broilers. Values were expressed as mean with standard error represented by vertical bars. ^a–c^Means with different letters differ significantly among the groups (*P* < 0.05). *IL-1β*, interleukin-1β; *IL-2*, interleukin-2; *IL-4*, interleukin-4; *IL-8*, interleukin-8; *IL-10*, interleukin-10; *TNF-α*, tumor necrosis factor-α; *IFN-*γ, interferon-γ; *iNOS*, inducible nitric oxide synthase.

Moreover, the mRNA expressions of jejunal IL-1β and TNF-α and ileal IL-8, TNF-α, IFN-γ, and iNOS were up-regulated after NE infection compared with those in the CON group (Figure 5, *P* < 0.05). After the intervention with 600 mg/kg of TA, the production of these cytokines was significantly declined (*P* < 0.05). Moreover, the NE infection group showed lower mRNA expressions of ileal IL-4 and jejunal IL-10 compared to the TA administration group (*P* < 0.05). Taken together, 600 mg/kg of TA administration may ameliorate NE through modulating the production of pro-inflammatory and anti-inflammatory cytokines.

### Expression of Molecules Involved in NF-κB Signaling Pathway

**FIGURE 6:**
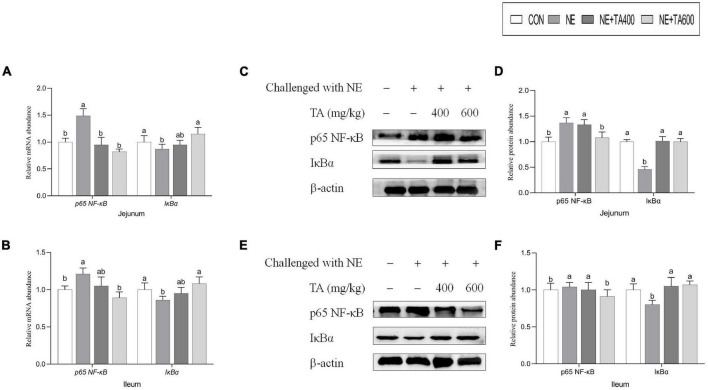
The effects of TA on the intestinal NF-κB signaling pathway of subclinical NE broilers. **(A,B)** Jejunal and ileal mRNA abundance of p65 *NF-*κ*B* and *I*κ*B*α, **(C,D)** jejunal nuclear translocation level of p65 NF-κB and cytoplasmic level of IκBα, **(E,F)** ileal nuclear translocation level of p65 NF-κB and cytoplasmic level of IκBα. Values were expressed as mean with standard error represented by vertical bars. ^a,b^Means with different letters differ significantly among the groups (*P* < 0.05). NF-κB, nuclear factor kappa beta; IκBα, NF-kappa-B inhibitor alpha.

To ascertain whether the gut-projective effects of TA in broilers challenged with subclinical NE were achieved through down-regulating inflammatory signaling pathway, we detected the expression of p65 NF-κB and IκBα in the jejunum and ileum. As illustrated in Figure 6, subclinical NE infection increased the mRNA levels of *p65 NF-*κ*B* but decreased IκBα in jejunal and ileal tissues of broilers as compared with the controls (*P* < 0.05). Moreover, western blot analysis showed that the NE birds had higher protein levels of jejunal and ileal p65 NF-κB, but lower jejunal and ileal IκBα (an inhibitor of NF-κB), compared with the birds in the CON group (Figure 6, *P* < 0.05). However, the activation of NE infection on NF-κB signaling pathway was suppressed by TA administration (*P* < 0.05).

### Cecal Microbiota Composition

**FIGURE 7:**
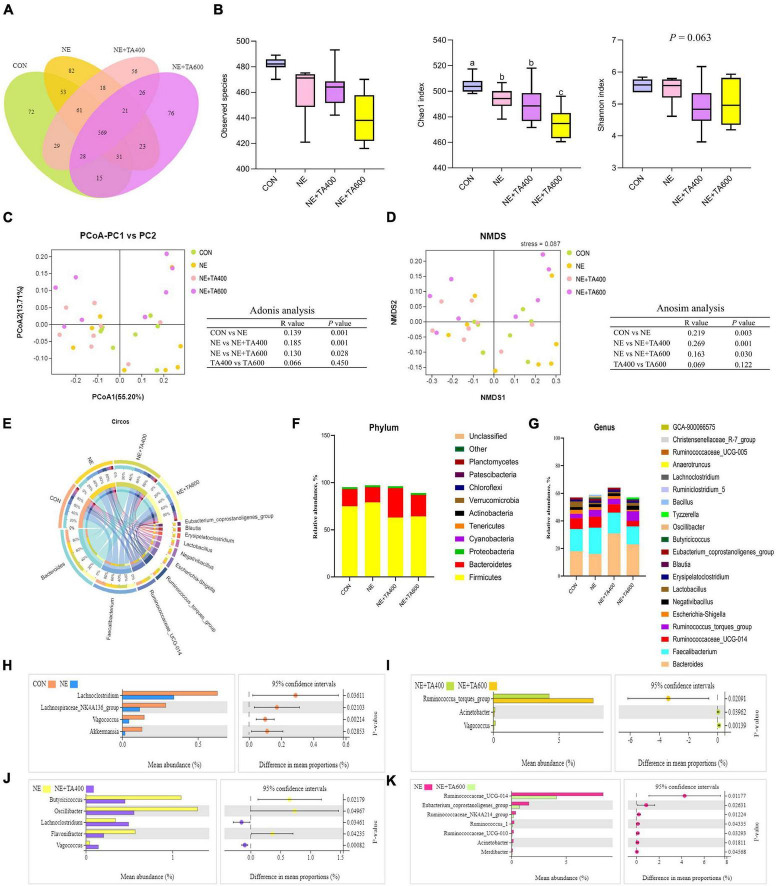
The effects of TA on the cecal microbial composition of subclinical NE broilers (*n* = 8). **(A)** Venn diagram of shared and unique OTUs between groups; **(B)** alpha diversity index: observed species, Chao 1, and Shannon. ^a,b^Means with different letters differ significantly among the groups (*P* < 0.05). **(C)** Principal coordinate analysis (PCoA) and Adonis analysis based on weighted UniFrac distances among samples; **(D)** non-metric multi-dimensional scaling (NMDS) and Anosim analysis based on weighted UniFrac distances among samples; **(E)** In the Circos plot, the upper semicircle represented the relative abundance of each genus per group, and the lower semicircle exhibited the microbial genera composition; relative abundance of microbial community at the phylum **(F)** and genus **(G)** levels. Significant differences on phylotypes at the genus level were observed between the CON and NE groups **(H)**, NE + TA400 and NE + TA600 groups **(I)**, NE and NE + TA400 groups **(J)**, and NE and NE + TA600 groups **(K)**, and Welch’s *t-*test was conducted to determine the significant difference among the groups. Significant difference among the groups was declared at *P* < 0.05.

To reveal the impact of TA on gut microbiota community of broilers under subclinical NE challenge, we conducted the analysis of the V3–V4 region of 16S rDNA gene sequences of microbiota in cecal contents. The rarefaction curve (Supplementary Figure 1) showed clear asymptotes, thus confirming the completeness of the sampling. Sequences were classified into 1,160 OTUs from 32 samples (four groups, *n* = 8). The Venn diagram showed that there were 569 shared OTUs among four groups, and 72, 82, 56, and 76 OTUs were unique in the CON, NE, NE + TA400, and NE + TA600 groups, respectively. The results of alpha diversity analysis showed that the Chao 1 index of the NE + TA600 group was markedly lower than that of the CON and NE groups (*P* < 0.01), and the Shannon index tended to be decreased by TA inclusion (*P* = 0.063). For the beta diversity, the PCoA (PC1 55.20% vs. PC2 13.71%) and NMDS (stress = 0.087) analyses based on weighted UniFrac distances showed a distinct clustering of the microbial community for each group. The results of Adonis and Anosim analyses further confirmed that the microbiota community of the NE group was markedly separate from that of the CON and TA inclusion groups (*P* < 0.05). However, the diversity of bacterial community was similar between 400 and 600 mg/kg of TA administration (Figure 7, *P* > 0.05).

As shown in Figure 7E, *Bacteroides*, *Faecalibacterium*, *Ruminococcaceae_UCG-014*, *Ruminococcus_torques_group*, *Escherichia-Shigella*, *Negativibacillus*, *Lactobacillus*, *Erysipelatoclostridium*, *Blautia*, and *Eubacterium_ coprostanoligenes_group* were the most dominant genera in the four experimental groups. At the phylum level, Firmicutes, Bacteroidetes, and Proteobacteria were the predominant bacteria in the cecum of broilers. We found that subclinical NE increased (*P* < 0.05) the relative abundance of Firmicutes whereas TA inclusion reversed that (Figure 7 and Supplementary Table 1). Differences in the microbiota composition at the genus level were compared, and the significant differences between the cecal microbiota of the CON and NE, NE + TA400 and NE + TA600, NE and NE + TA400, and NE and NE + TA600 groups were further investigated. The discrepancy of cecal microbiota among the CON and NE + TA400 or NE + TA600 groups is shown in Supplementary Figure 2. When compared with the CON group, the dysbiosis of gut microbiota in the NE group was characterized by a lower abundance of *Akkermansia* (*P* = 0.029), *Vagococcus* (*P* = 0.002), *Lachnospiraceae*_*NK4A136_group* (*P* = 0.021), and *Lachnoclostridium* (*P* = 0.036). Moreover, 600 mg/kg of TA administration significantly reduced the abundance of *Ruminococcaceae_UCG-014* (*P* = 0.012), *Eubacterium*_*coprostanoligenes_group* (*P* = 0.026), *Ruminococcaceae_NK4A214_group* (*P* = 0.012), *Ruminococcus_1* (*P* = 0.043), and *Ruminococcaceae_UCG-010* (*P* = 0.033) in comparison with the NE birds and notably promoted the abundance of *Ruminococcus*_*torques_group* compared with that in the CON group (*P* = 0.003) and 400 mg/kg of TA-supplemented group (*P* = 0.021). *Butyricicoccus* (*P* = 0.022), *Oscillibacter* (*P* = 0.049), and *Flavonifractor* (*P* = 0.042) were less abundant, whereas *Lachnoclostridium* (*P* = 0.035) and *Vagococcus* (*P* = 0.0008) were more abundant following 400 mg/kg of TA supplementation in contrast to the NE group.

## Discussion

It is well known that birds infected with clinical NE show a range of symptoms including ruffled feathers, apocleisis, diarrhea, huddling, pathologic intestinal necrotic foci, and sudden mass death (To et al., 2017), while subclinical NE birds showed poor digestion and growth performance due to intestinal lesion (Wang et al., 2017). Numerous studies have investigated the effects of different kinds of additives, including probiotics, prebiotics, and phytogenic substances on poultry immunity and performance during subclinical NE challenge, whereas information on TA is lacking. Hence, we conducted this trial to investigate the alleviative potential of TA on subclinical NE infection in broilers. In the present study, subclinical NE-infected birds showed severe intestinal mucosal injury, focal necrosis, and multiple hemorrhagic spots. In contrast, the administration of TA reduced the macroscopic intestinal lesions. This is similar with the research on the anti-inflammatory activity of *Foeniculum vulgare* essential oil (81.08% TA) against acetic acid-induced colitis in rats (Rezayat et al., 2018). This study revealed that the final BW and ADG of broilers were significantly decreased, but FCR was increased after NE infection. Accordingly, the intestinal barrier integrity was damaged. We found that serum DAO activity and D-lactate concentration associated with intestinal barrier integrity (Wu et al., 2018) were distinctly increased, which is in accordance with the down-regulated intestinal protein levels of occludin and ZO-1 during NE infection. Interestingly, the intestinal protein level of CLDN-1 was not affected by NE infection. Intestinal mucous barrier consisting of tight junction proteins is the first defense line against invasion of harmful bacteria (Broom and Kogut, 2018), while the actual function of CLDN-1 is controversial. Li et al. (2021) indicated that CLDN-1 may be involved in increasing paracellular permeability. Previous studies elucidated that NE infection could trigger specific mucosal immune responses and induce intestinal inflammation, thereby affecting bird’s growth performance (Park et al., 2008; Forder et al., 2012). Furthermore, reduced intestinal VH and VH/CD and increased CD were observed after NE infection in the present study, suggesting that NE infection may decrease nutrient digestion and absorption. However, TA administration at 600 mg/kg restored the intestinal barrier integrity, maintained the normal villus/crypt ratio, and thereby improved the growth performance of birds. These direct observations are similar with the results of Yi et al. (2021) who revealed the alleviative effects of TA on Enterotoxigenic *Escherichia coli*-induced intestinal barrier disruption and inflammation in piglets. An earlier study reported that TA had remarkable gastroprotective and mucous-protective activities (Freire et al., 2005). Additionally, IL-10 was reported to be involved in maintaining the intestinal barrier integrity (Morhardt et al., 2019). In this study, TA administration elevated jejunal IL-10 level during NE challenge, suggesting the protective effects of TA on intestinal barrier integrity. TA administration also led to a remarkable inhibition of apoptosis and promotion of PCNA proliferation in the present study. An earlier study revealed that TNF is one of the most apoptosis-inducing cytokines among all the cytokines, and TA abolished TNF-induced apoptosis through suppression of caspase activation (Chainy et al., 2000). This observation validated our results and indicated that TA may inhibit intestinal excessive apoptosis due to NE infection with the inhibitory effect on TNF-α. Due to short lifespan, intestinal epithelium cells require to be rapidly renewed and proliferated *via* replication of undifferentiated cells. These proliferative cells, which can be detected by an endogenous protein called PCNA, are continuously generated in the crypt regions, eventually differentiating into secretory cells to synthesize intestinal mucous layers against pathogenic microorganisms (Emami and Dalloul, 2021). Thus, the elevated PCNA proliferation level in the ileum further confirmed that TA had the ability to restore the integrity of intestinal epithelium of subclinical NE birds.

NF-κB is a major transcription factor involved in inflammatory diseases. p65 protein is responsible for its function and Rel homology domain where IκBα binds (Napetschnig and Wu, 2013). As an inhibitor of NF-κB, IκBα restrains the activation of NF-κB through forming an inactive NF-κB/IκBα complex in cytoplasm. In the presence of extracellular stimuli, phosphorylation-mediated degradation of IκBα occurred, following nuclear translocation of active NF-κB and induction of downstream gene transcription (Feng et al., 2019). The current results demonstrated that TA resulted in a remarkable reduction of intestinal IκBα degradation and NF-κB activation during subclinical NE infection. Consistent with our results, there are considerable evidence confirming that TA may ameliorate inflammatory process through inhibition of NF-κB signaling pathway. Cho et al. (2013) revealed that TA protected against hepatic ischemia/reperfusion (I/R) injury through down-regulating nuclear NF-κB level. In the study conducted by Rezayat et al. (2018), *Foeniculum vulgare* essential oil mainly containing 81.08% of TA exhibited alleviative effects on acetic acid-induced colitis in rats *via* inhibiting the protein expression of NF-κB. These powerful reports confirmed the underlying therapeutic effects of TA against NE infection by inhibition of NF-κB signaling pathway. In addition, activated NF-κB promoted the transcription of several genes encoding pro-inflammatory mediators including TNF-α, iNOS, and IL-6 (Sung et al., 2012; Wang et al., 2012). In the present study, we found that the concentrations of serum TNF-α and IFN-γ and intestinal IL-1β, IL-8, TNF-α, and IFN-γ were elevated, but jejunal levels of IL-10 and sIgA were decreased after subclinical NE infection. SIgA, which plays important roles in mucosal immunity, is mainly secreted from plasma cells of intestinal lamina propria (Macpherson et al., 2018). IL-10, an anti-inflammatory cytokine, has a potential to inhibit many pro-inflammatory signals when intestinal inflammatory diseases occur (Li et al., 2021). Apparently, our data clearly demonstrated the inhibitory effect of TA on the pro-inflammatory cytokines. This observation might be attributed to the suppression of NF-κB pathway by TA. Prior to that, TA showed an anti-inflammatory activity in various inflammatory models by targeting pro-inflammatory cytokines such as TNF-α and IL-6 during chronic obstructive pulmonary disease (Kim et al., 2017), inhibiting TNF-α expression in colitis rats (Rezayat et al., 2018), and decreasing serum TNF-α and IL-6 concentration during I/R injury in mice (Cho et al., 2013).

There is increasing evidence proving that intestinal pathogen infection could alter gut microbiota and thereby induce immune response (Broom and Kogut, 2018; Clavijo and Flórez, 2018). TA has been reported to inhibit a wide range of pathogenic bacteria (Huang et al., 2010; Kwiatkowski et al., 2019). To clarify the microbiota shifts of broilers during subclinical NE infection, we investigated the cecal microbiota population and diversity by 16S rDNA sequencing. The results showed that the alpha diversity of the cecal microbiota in the NE + TA600 group was significantly lower than that in the CON and NE groups. The analysis on beta diversity revealed that the microbiota community of the NE group was markedly separate from that of the CON and TA inclusion groups. This observation suggests that TA has positive effects on modifying the alterations of intestinal microbiota community cause by NE. Wu et al. (2014) revealed that *Eimeria* infection had minor changes in microbiota of broilers but reduced cecal *Lachnospiraceae* groups. Our results revealed that the beneficial bacteria *Lachnoclostridium*, *Lachnospiraceae_NK4A136_group*, and *Akkermansia* were decreased due to NE infection compared to the CON group. *Lachnospiraceae_NK4A136_group* could colonize in the gut and produce butyric acid, which is reported to be involved in restoring intestinal barrier (Etxeberria et al., 2016). *Akkermansia* contributed to promote intestinal barrier function by increasing mucosal production (Grander et al., 2017). We found that administration of 600 mg/kg of TA notably decreased the abundance of Firmicutes phylum and *Ruminococcaceae*, *Eubacterium*, *Ruminococcus*, and *Acinetobacter* genera during NE infection. In favor of our findings, broilers fed diets containing probiotic/essential oils appeared to have a lower *Ruminococcaceae UCG_014* and better performance compared to the CON group (Khodambashi et al., 2020). Additionally, *Butyricicoccus*, *Oscillibacter*, and *Flavonifractor* genera were less abundant, whereas *Lachnoclostridium* were more abundant following 400 mg/kg of TA supplementation in contrast to the NE group. The genus *Oscillibacter* was observed to be closely related to gut permeability and inflammation (Li et al., 2021). Information on the effects of TA on intestinal microbiota in broilers is unavailable. In the previous study conducted by us, we have confirmed that TA inclusion could decrease cecal *Escherichia coli* population but increase cecal *Bifidobacterium* population and ileal *Bifidobacterium* in broilers (Yu et al., 2021), indicating the antimicrobial activity of TA on the gut of broilers. Further research requires to be characterized to investigate the inhibitory effect of TA on *C. perfringens in vitro* and *in vivo* studies. In addition, it is necessary to further investigate the influence of different doses of TA supplementation on the microbial community of broilers subjected to subclinical NE infection and the interactions between the altered microbiota and intestinal immune function.

Interestingly, TA administration at 600 mg/kg had better ameliorating effects against subclinical NE infection. Our previous study revealed that high level of TA (800 mg/kg) had an inferior effect on nutrient digestibility and intestinal barrier function (Yu et al., 2021). Ding et al. (2020) also demonstrated that SAO improved the growth performance antioxidant status of broilers in a dose-dependent manner. As mentioned earlier, TA, which is faint yellow with a highly anise flavor, may be responsible for the bioactivities of SAO. The pungent anise smell may influence the diet’s taste and broilers’ appetite, thereby leading to stress response of broilers when supplemented at a high concentration. Therefore, we speculated that the alleviating effects of TA against subclinical NE infection may be in a dose-dependent manner. Further research on the ameliorating effects of high concentration of TA on the subclinical NE infection in broilers requires to be characterized.

## Conclusion

Dietary supplementation of TA at 600 mg/kg could alleviate the undesirable effects of subclinical NE infection by enhancing intestinal barrier integrity and intestinal immune response and modulating gut microbiota, and it could further improve growth performance in broilers.

## Data Availability Statement

The data presented in the study are deposited in the National Center for Biotechnology Information (NCBI) Sequence Read Archive (SRA) repository, accession number PRJNA789352.

## Ethics Statement

All procedures in the animal experiments were reviewed and approved by the Institutional Animal Care and Use Committee of Nanjing Agricultural University (Permit No. SYXK-2017-0027).

## Author Contributions

CY made significant contributions in the conceptualization, methodology, and writing—original draft. DW and WY made significant contributions in the supervision of the study. YT and QL made significant contributions in the investigation and supervision of the study. TW made significant contributions in the supervision, project administration, and funding acquisition. ZY made significant contributions in the visualization, writing—review and editing, and funding acquisition. All authors contributed to the article and approved the submitted version.

## Conflict of Interest

The authors declare that the research was conducted in the absence of any commercial or financial relationships that could be construed as a potential conflict of interest.

## Publisher’s Note

All claims expressed in this article are solely those of the authors and do not necessarily represent those of their affiliated organizations, or those of the publisher, the editors and the reviewers. Any product that may be evaluated in this article, or claim that may be made by its manufacturer, is not guaranteed or endorsed by the publisher.

## Supplementary Material

Supplementary Figure 1 Rarefaction curve of the cecal microbiota based on operational taxonomic units (OTUs).

Supplementary Figure 2 Significant differences on phylotypes at the genus level were observed between the CON and NE + TA400 groups **(A)** and the CON and NE + TA600 groups **(B)**. Welch’s *t*-test was conducted to determine the significant difference among the groups. Significant difference among the groups was declared at *P* < 0.05. CON, control group; NE + TA400, NE birds supplemented with 400 mg/kg of TA; NE + TA600, NE birds supplemented with 600 mg/kg of TA.
