# Supplementary figures and images for: Trans-Anethole Alleviates Subclinical Necro-Haemorrhagic Enteritis-Induced Intestinal Barrier Dysfunction and Intestinal Inflammation in Broilers (part 1 of 5)
Source: Front Microbiol. 2022 Mar 21;13:831882. doi: 10.3389/fmicb.2022.831882 (PMC8977854; doi:10.3389/fmicb.2022.831882)

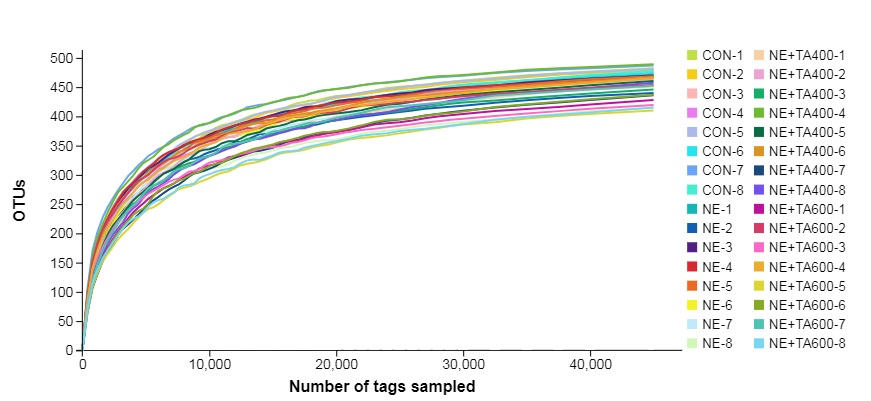

Supplement: Supplementary Figure 1 — Rarefaction curve of the cecal microbiota based on operational taxonomic units (OTUs). [file Image_1.JPEG]

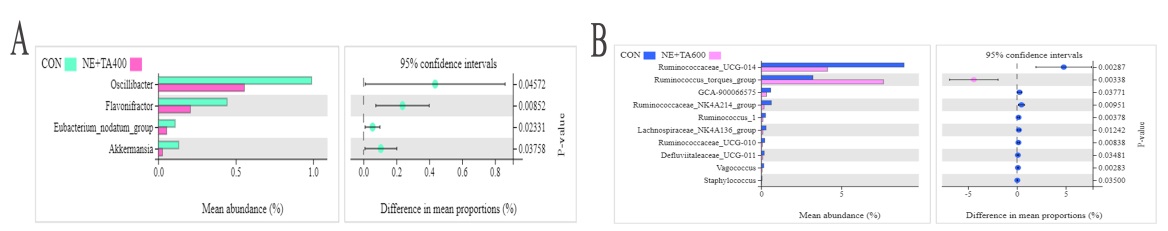

Supplement: Supplementary Figure 2 — Significant differences on phylotypes at the genus level were observed between the CON and NE + TA400 groups (A) and the CON and NE + TA600 groups (B). Welch’s t-test was conducted to determine the significant difference among the groups. Significant difference among the groups was declared at P < 0.05. CON, control group; NE + TA400, NE birds supplemented with 400 mg/kg of TA; NE + TA600, NE birds supplemented with 600 mg/kg of TA. [file Image_2.JPEG]

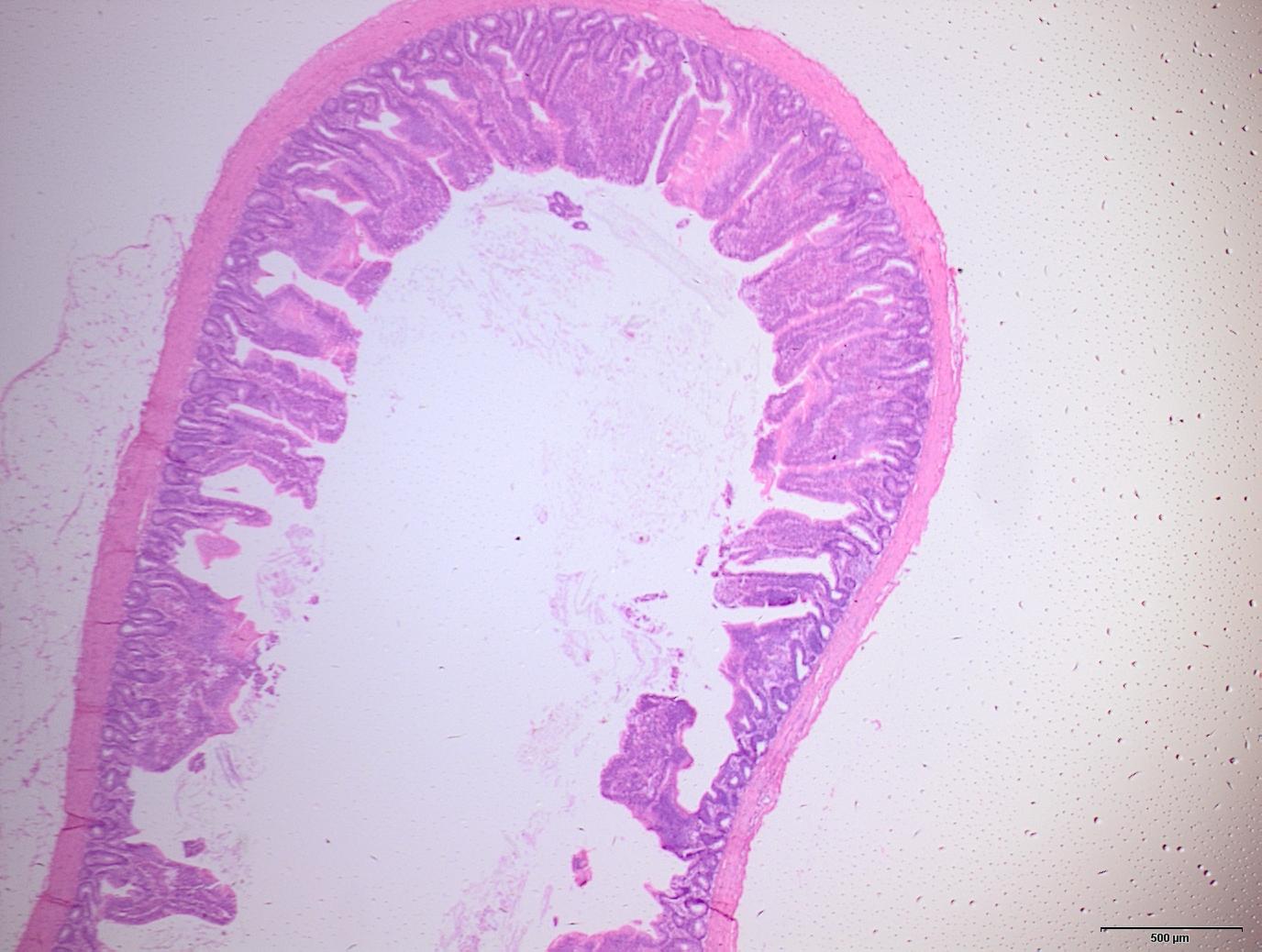

Supplement: Supplementary file 6 [file Data_Sheet_1.ZIP › Data sheet/Hematoxylin-eosin Staining/Ileum/CON group/1.jpg]

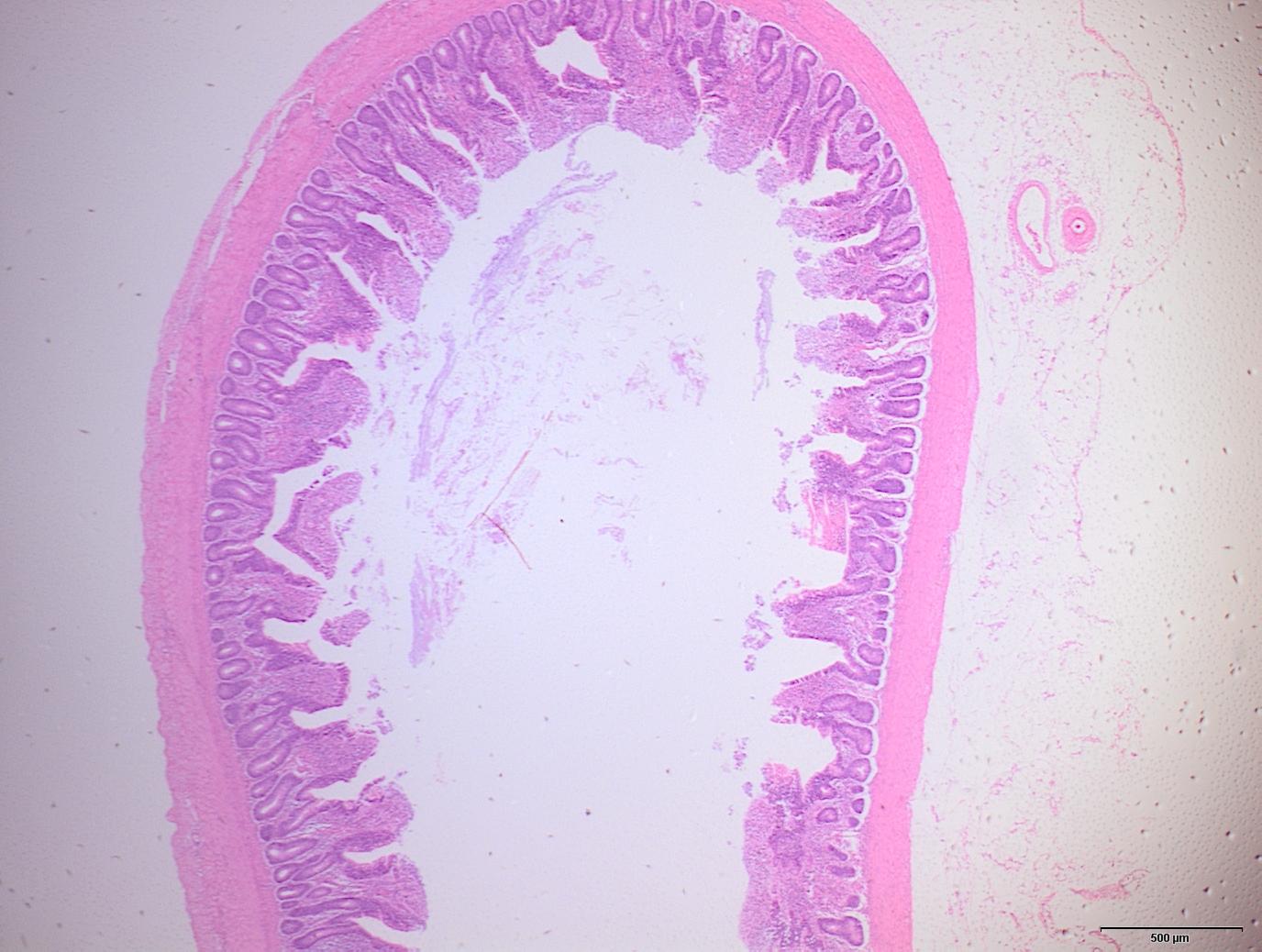

Supplement: Supplementary file 6 [file Data_Sheet_1.ZIP › Data sheet/Hematoxylin-eosin Staining/Ileum/CON group/2.jpg]

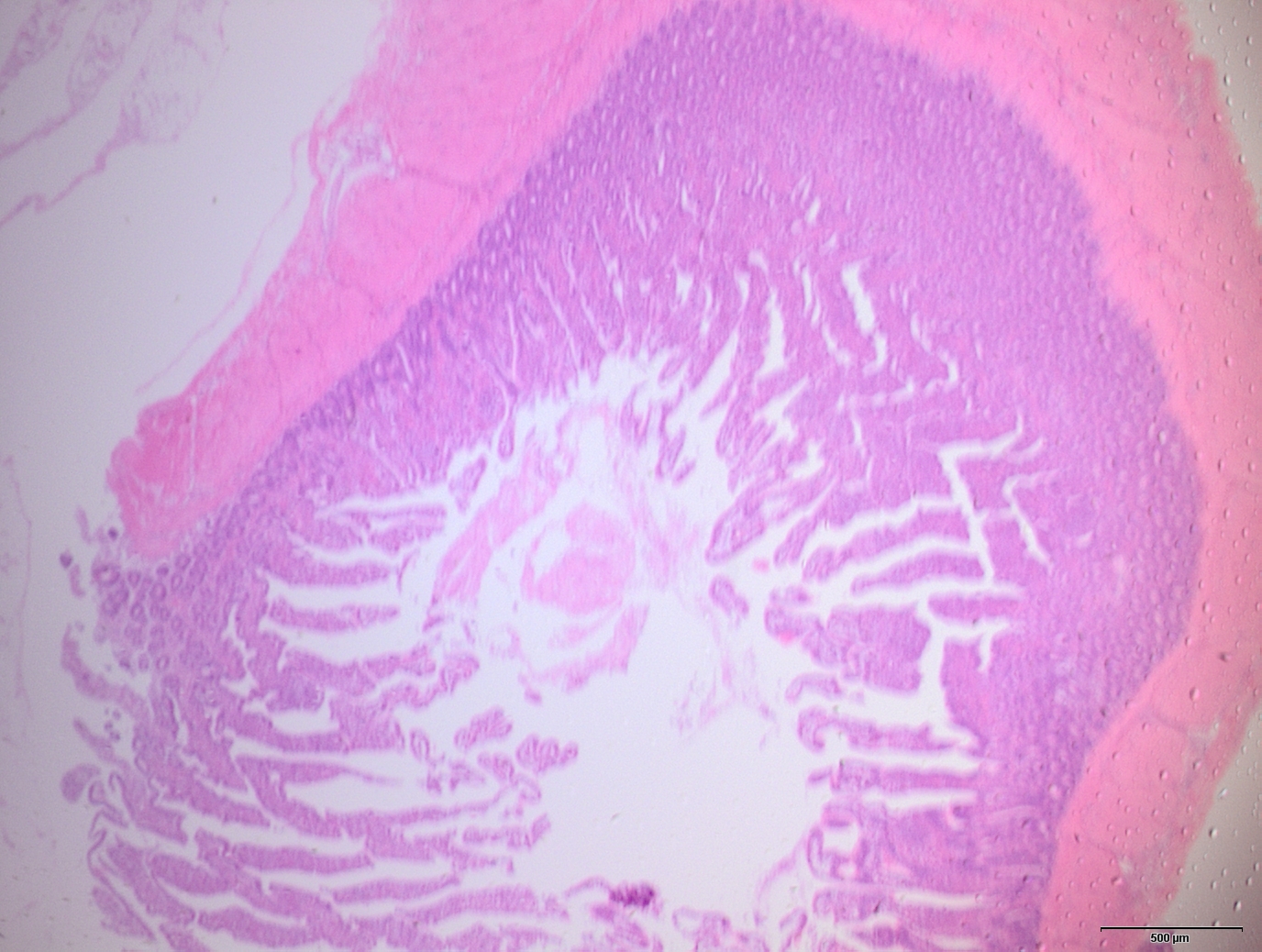

Supplement: Supplementary file 6 [file Data_Sheet_1.ZIP › Data sheet/Hematoxylin-eosin Staining/Ileum/CON group/3.jpg]

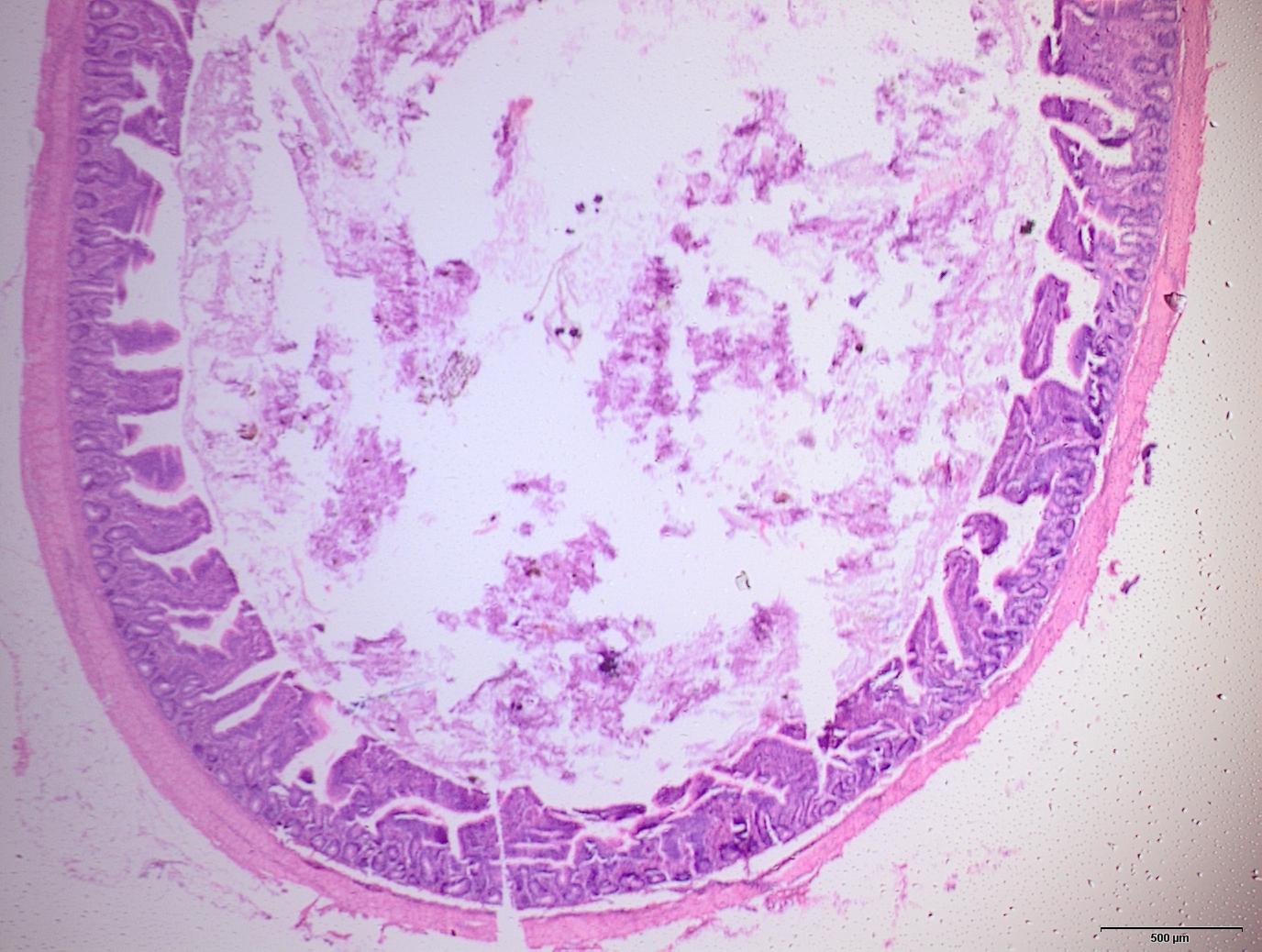

Supplement: Supplementary file 6 [file Data_Sheet_1.ZIP › Data sheet/Hematoxylin-eosin Staining/Ileum/CON group/4.jpg]

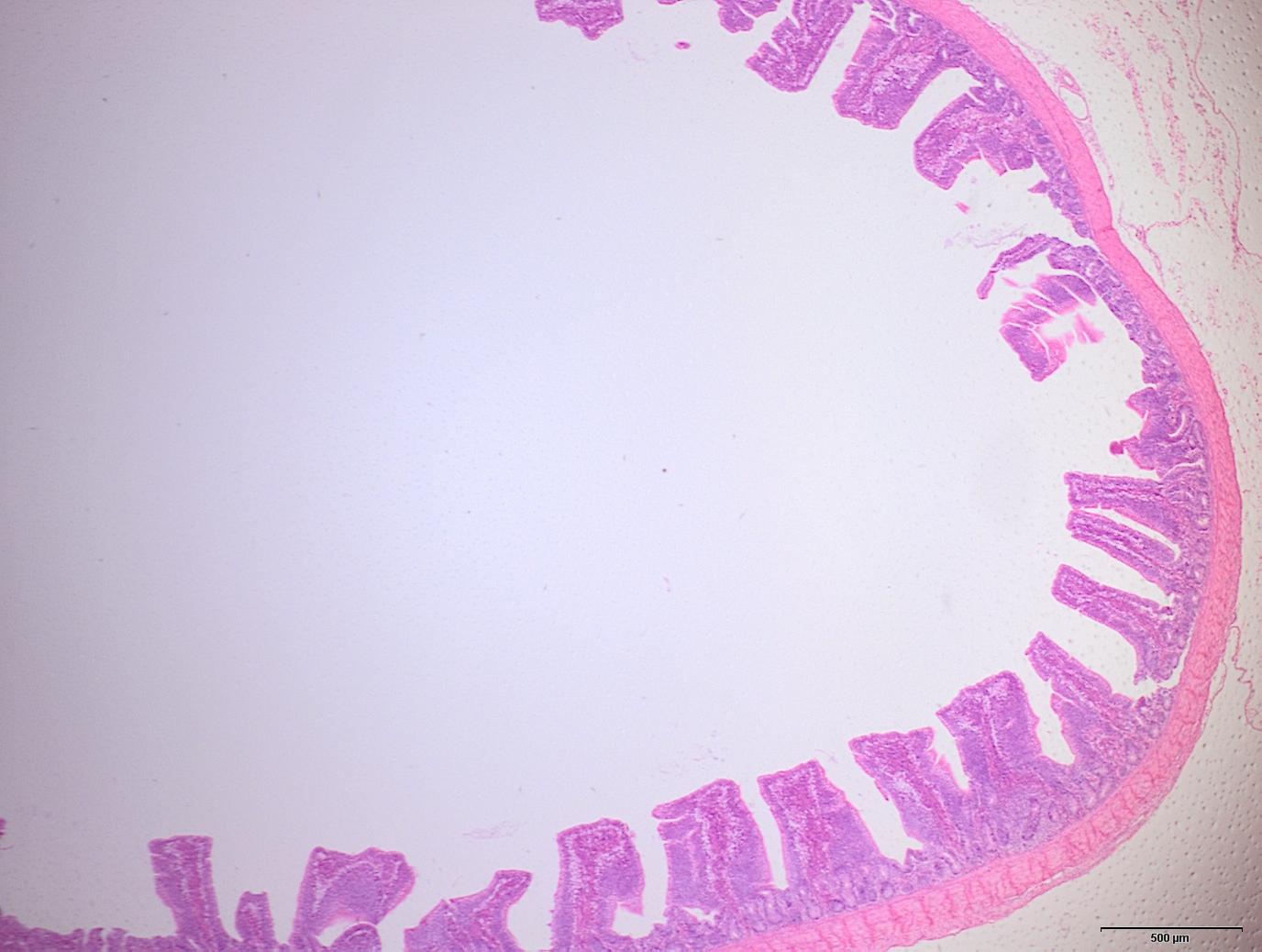

Supplement: Supplementary file 6 [file Data_Sheet_1.ZIP › Data sheet/Hematoxylin-eosin Staining/Ileum/CON group/5.jpg]

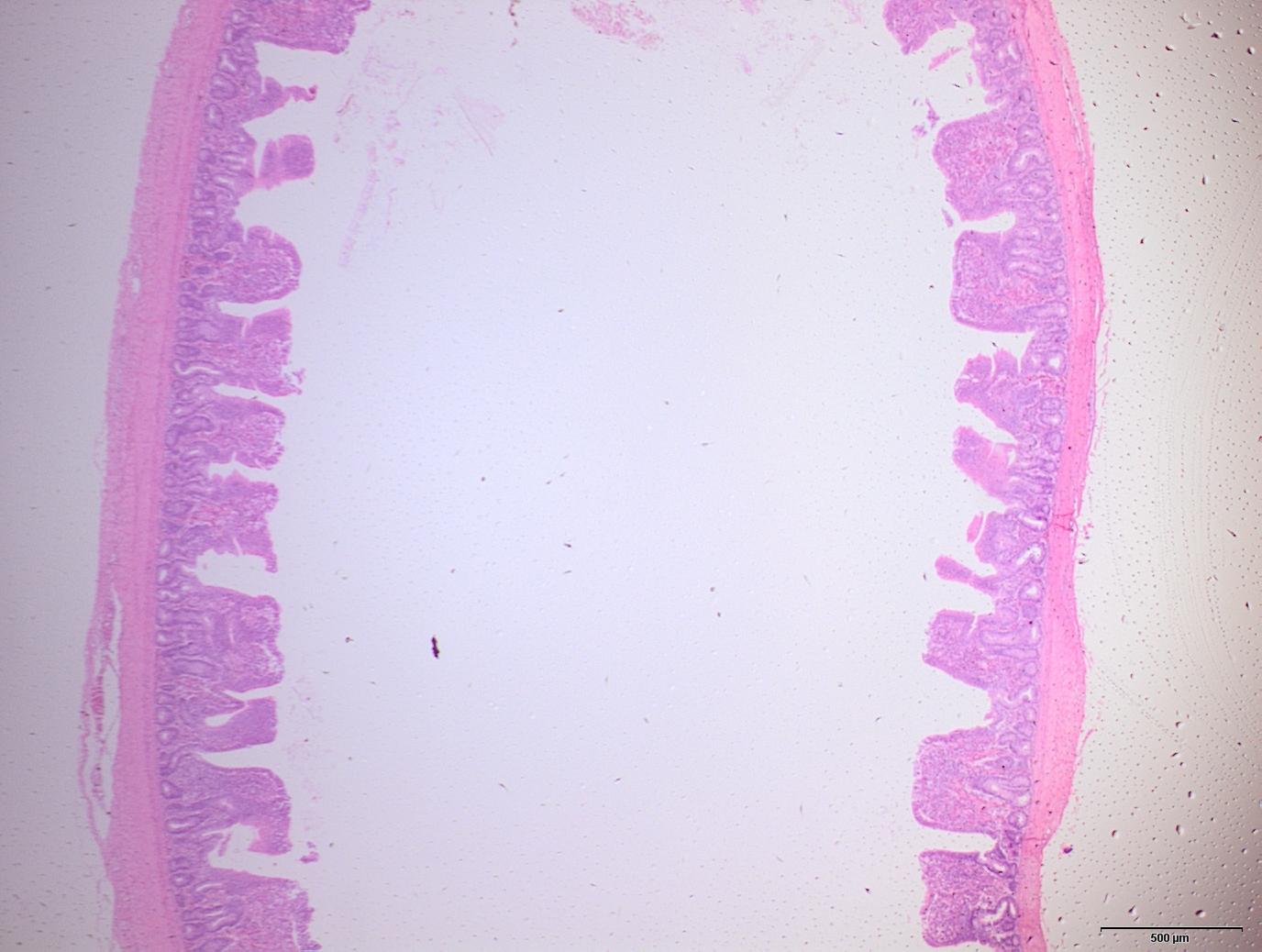

Supplement: Supplementary file 6 [file Data_Sheet_1.ZIP › Data sheet/Hematoxylin-eosin Staining/Ileum/CON group/6.jpg]

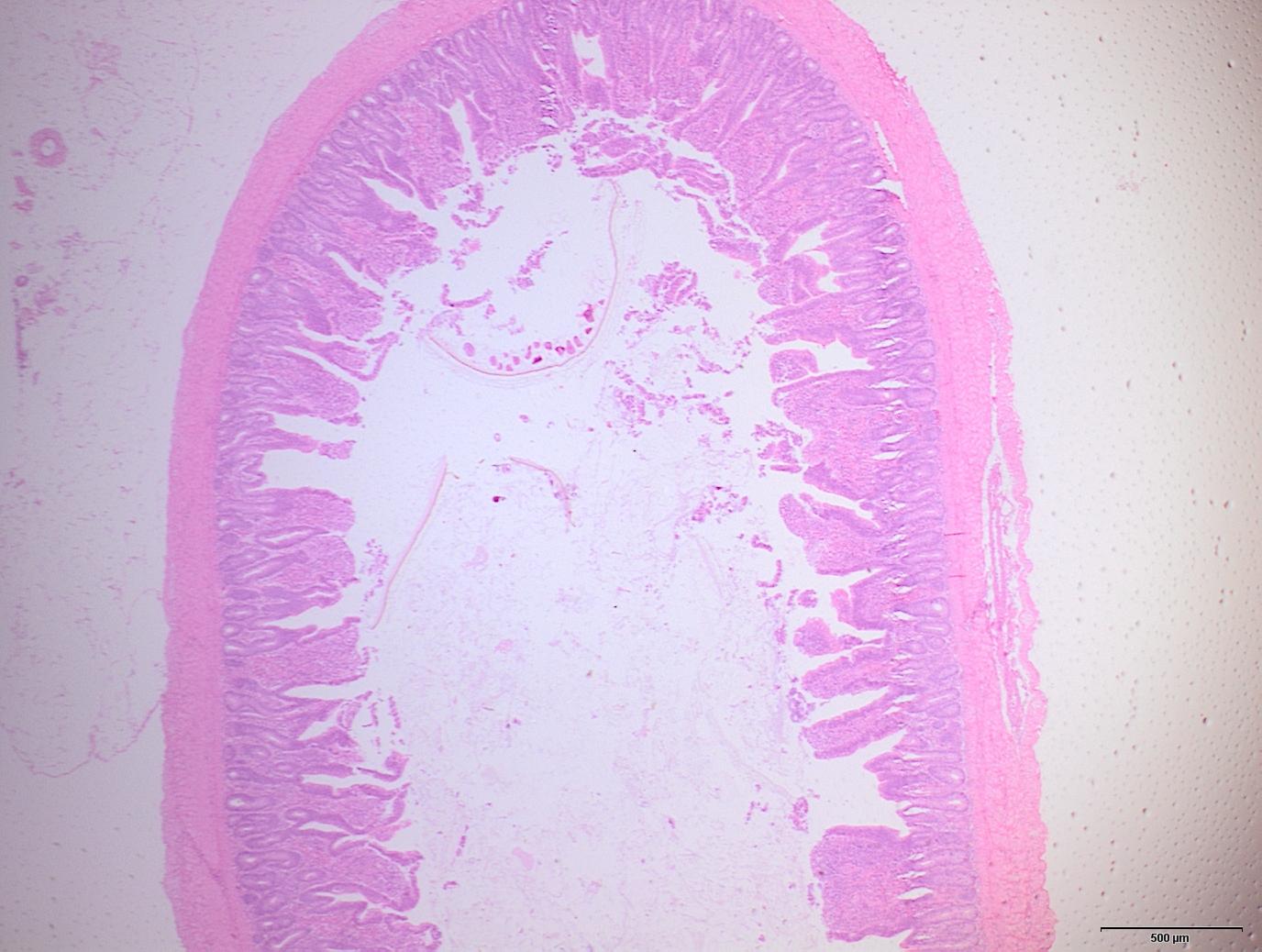

Supplement: Supplementary file 6 [file Data_Sheet_1.ZIP › Data sheet/Hematoxylin-eosin Staining/Ileum/CON group/7.jpg]

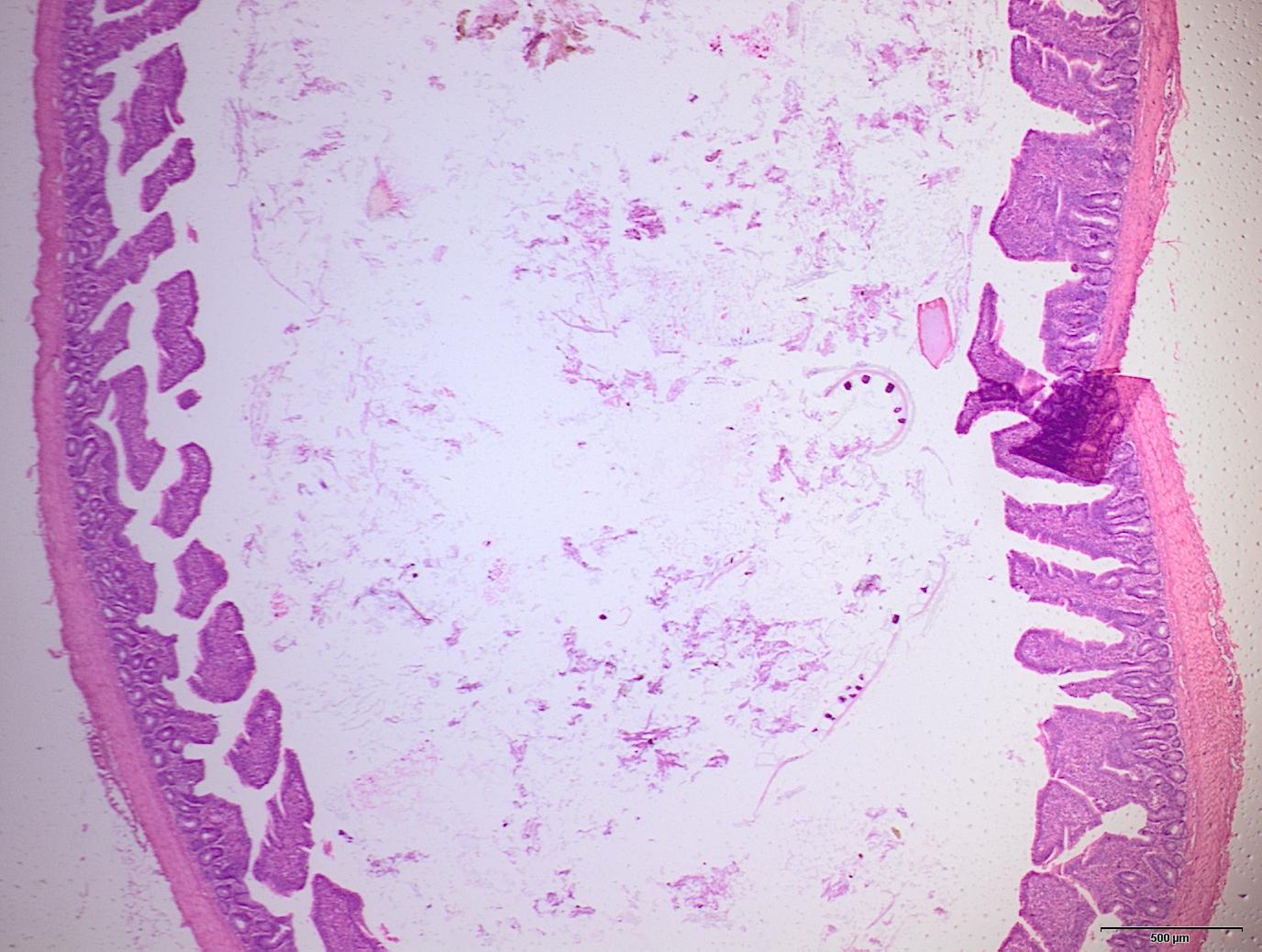

Supplement: Supplementary file 6 [file Data_Sheet_1.ZIP › Data sheet/Hematoxylin-eosin Staining/Ileum/CON group/8.jpg]

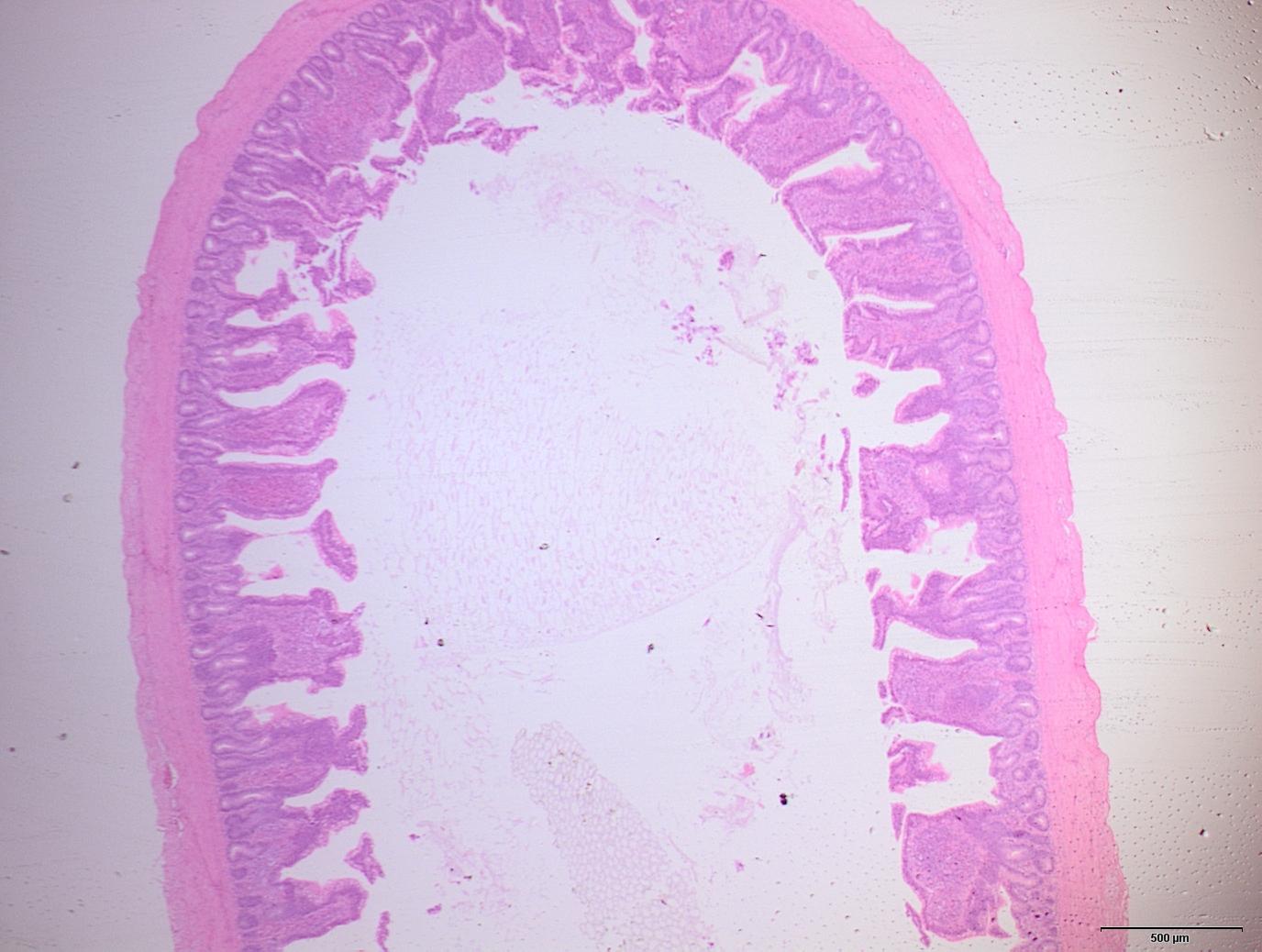

Supplement: Supplementary file 6 [file Data_Sheet_1.ZIP › Data sheet/Hematoxylin-eosin Staining/Ileum/NE group/1.jpg]

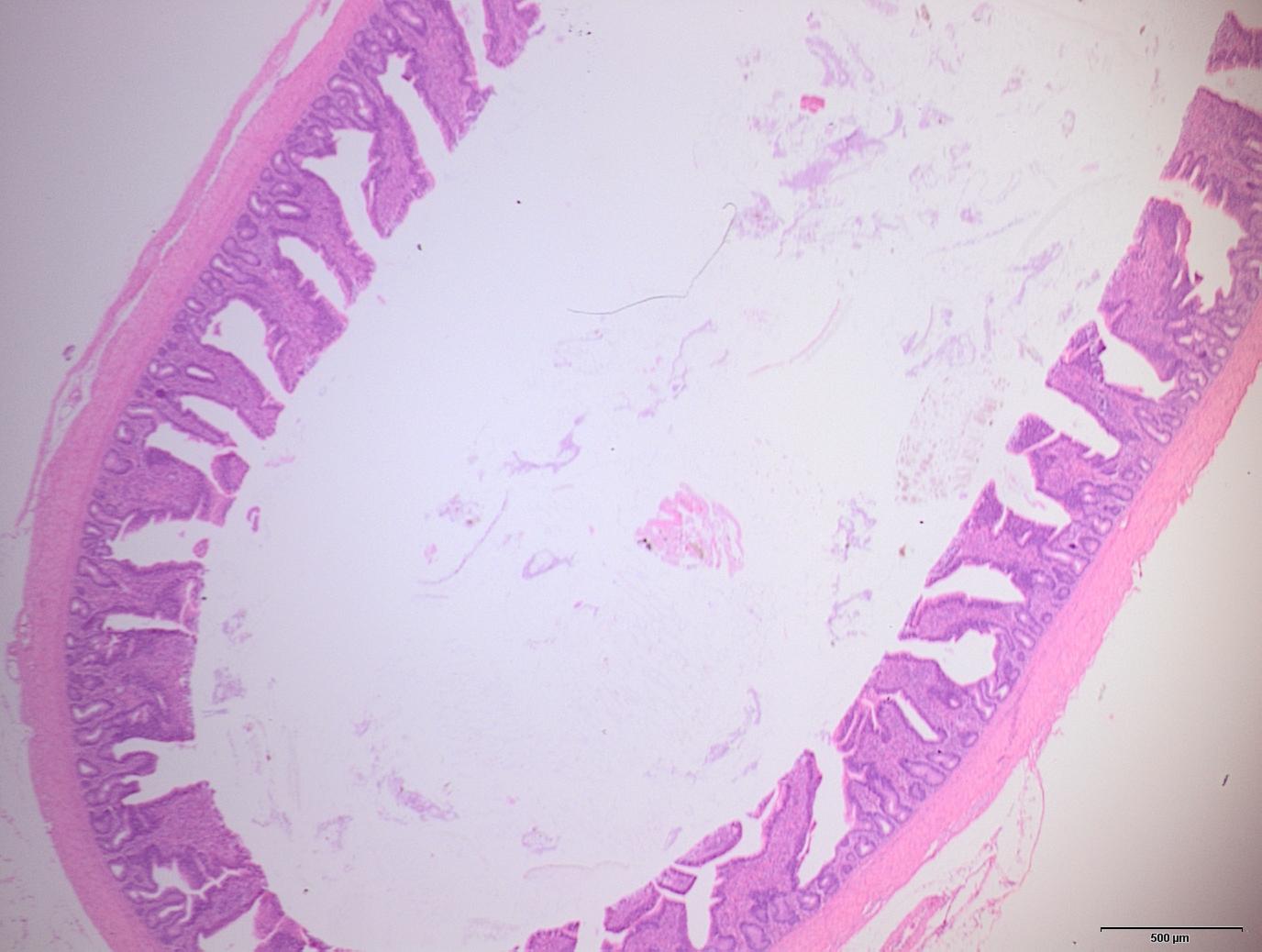

Supplement: Supplementary file 6 [file Data_Sheet_1.ZIP › Data sheet/Hematoxylin-eosin Staining/Ileum/NE group/2.jpg]

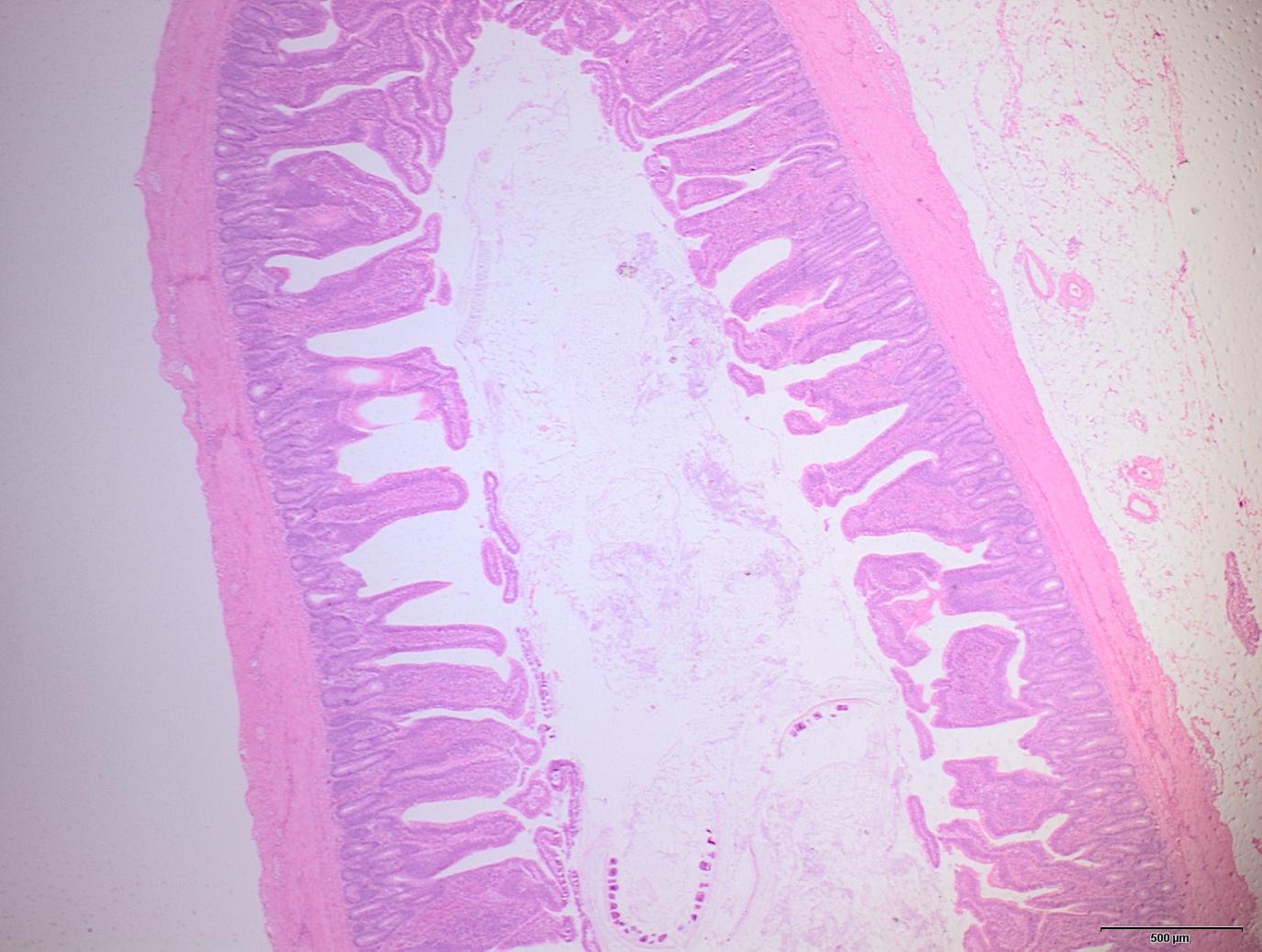

Supplement: Supplementary file 6 [file Data_Sheet_1.ZIP › Data sheet/Hematoxylin-eosin Staining/Ileum/NE group/3.jpg]

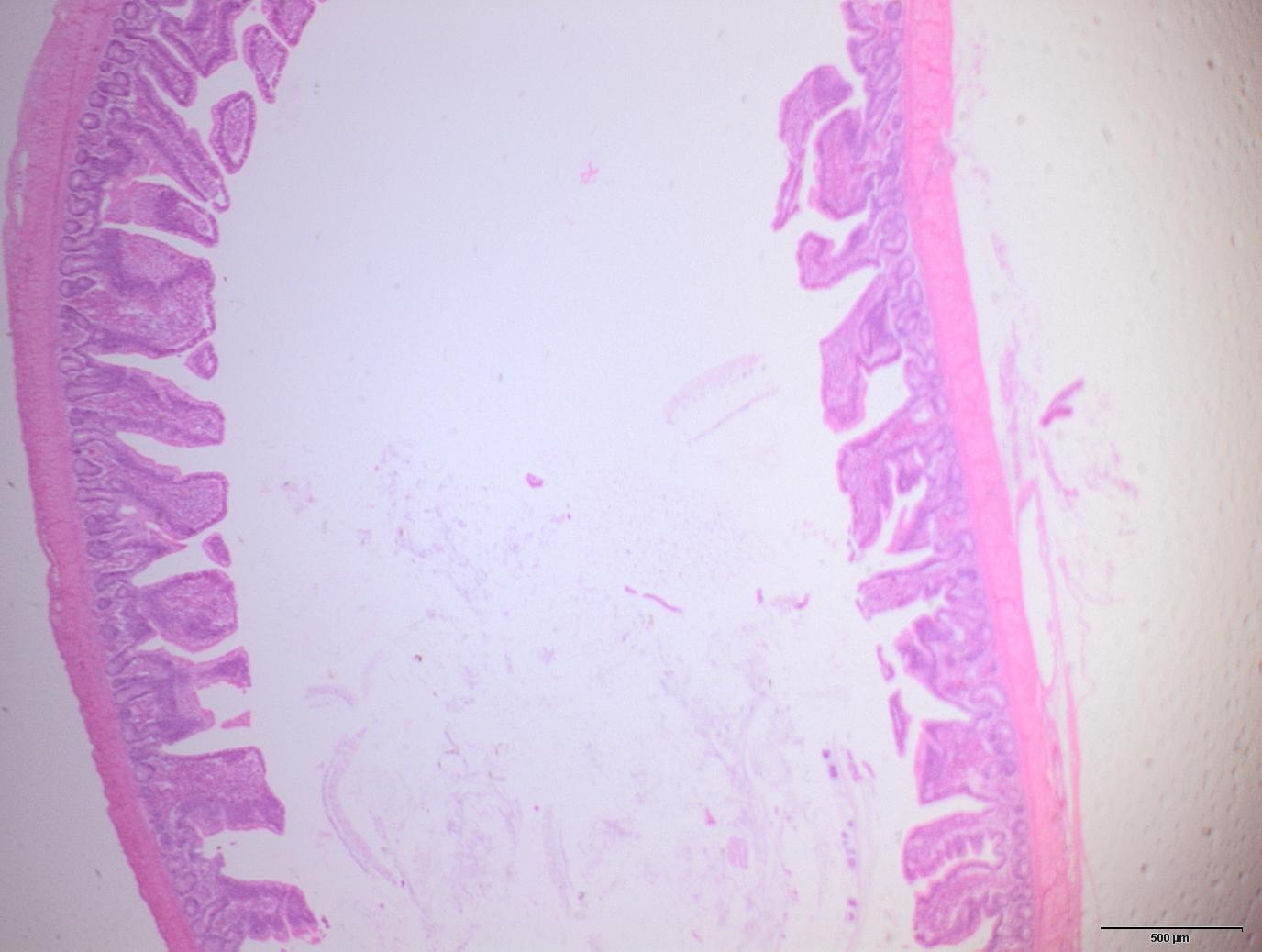

Supplement: Supplementary file 6 [file Data_Sheet_1.ZIP › Data sheet/Hematoxylin-eosin Staining/Ileum/NE group/4.jpg]

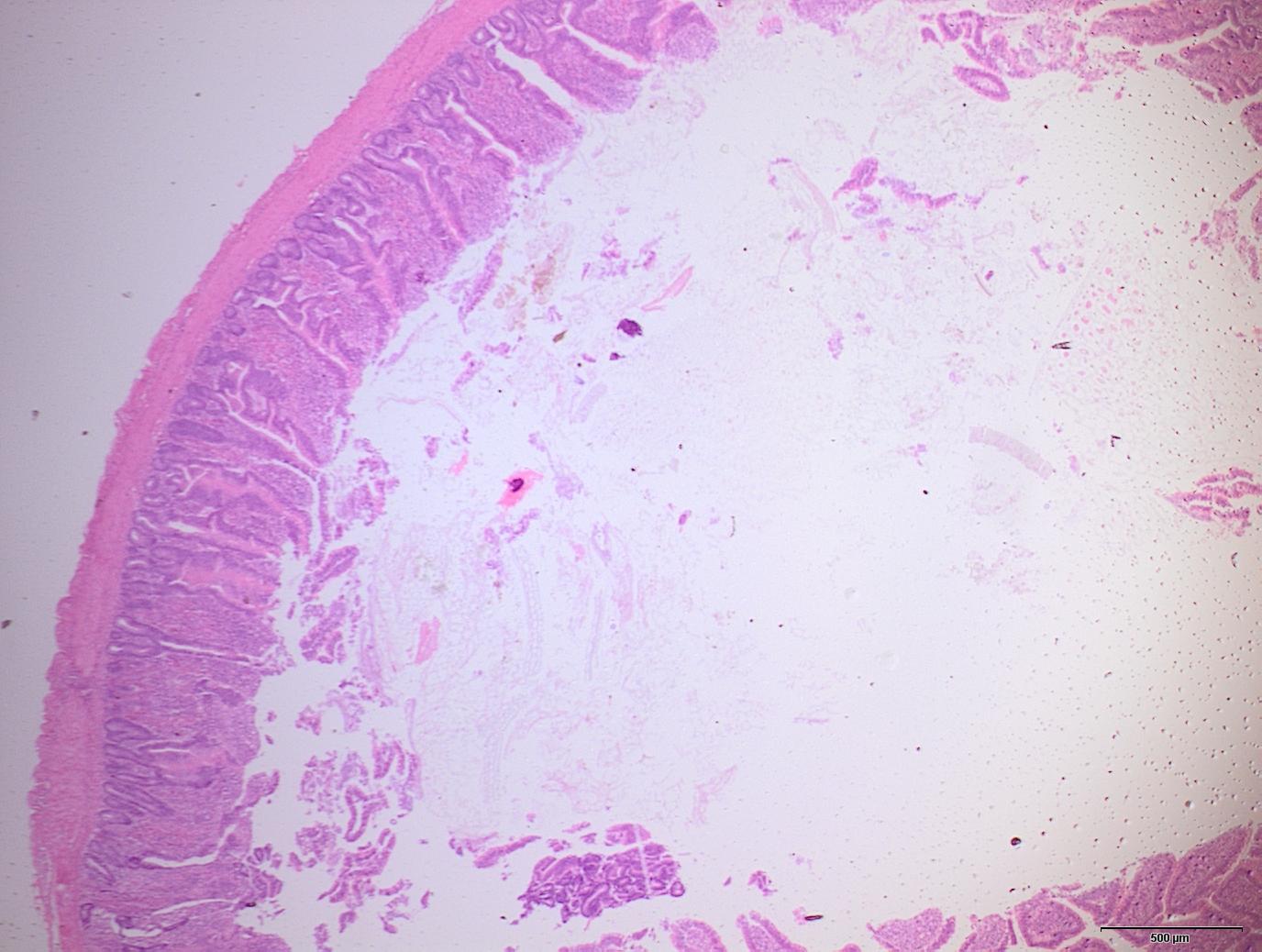

Supplement: Supplementary file 6 [file Data_Sheet_1.ZIP › Data sheet/Hematoxylin-eosin Staining/Ileum/NE group/5.jpg]

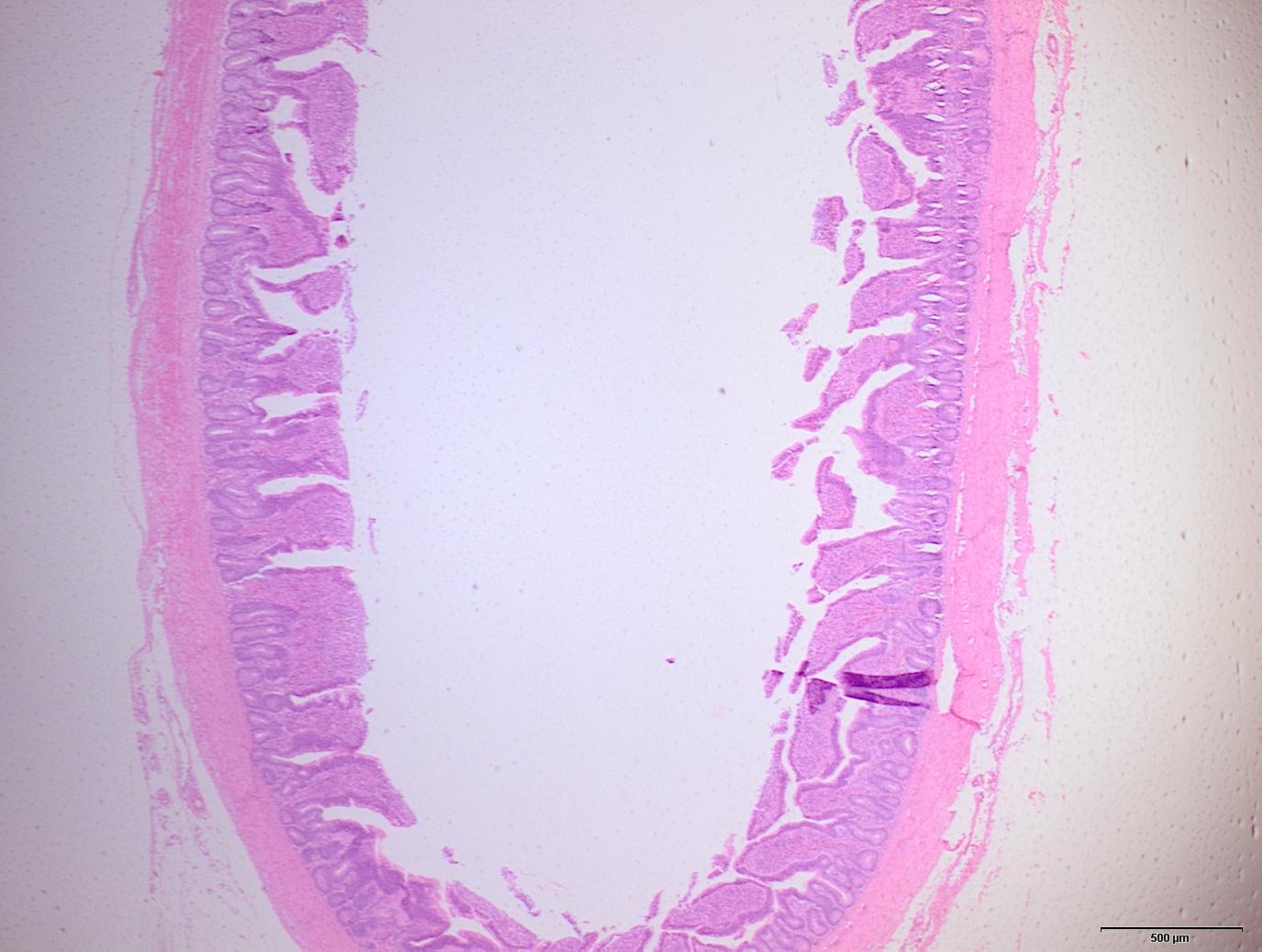

Supplement: Supplementary file 6 [file Data_Sheet_1.ZIP › Data sheet/Hematoxylin-eosin Staining/Ileum/NE group/6.jpg]

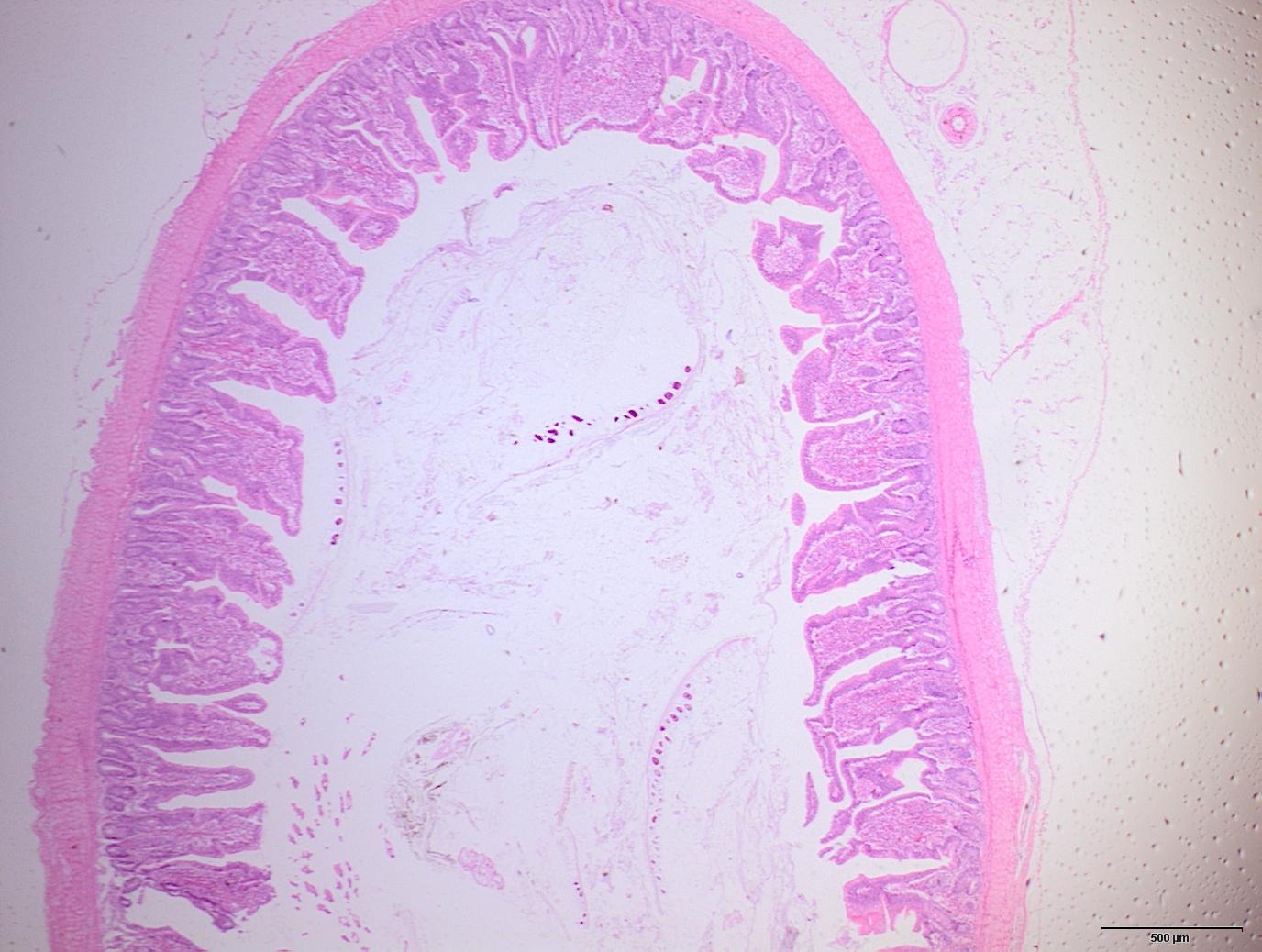

Supplement: Supplementary file 6 [file Data_Sheet_1.ZIP › Data sheet/Hematoxylin-eosin Staining/Ileum/NE group/7.jpg]

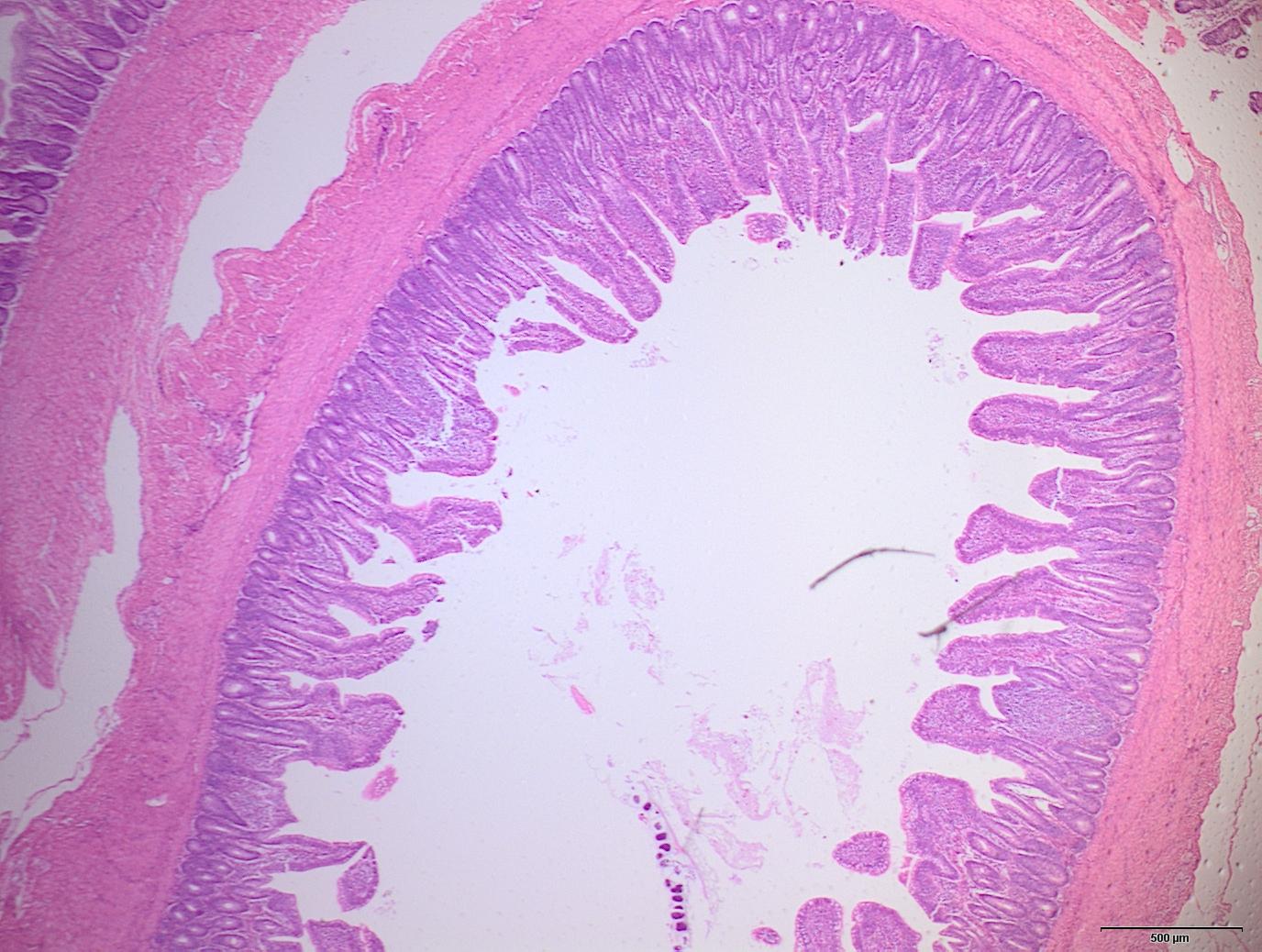

Supplement: Supplementary file 6 [file Data_Sheet_1.ZIP › Data sheet/Hematoxylin-eosin Staining/Ileum/NE group/8.jpg]

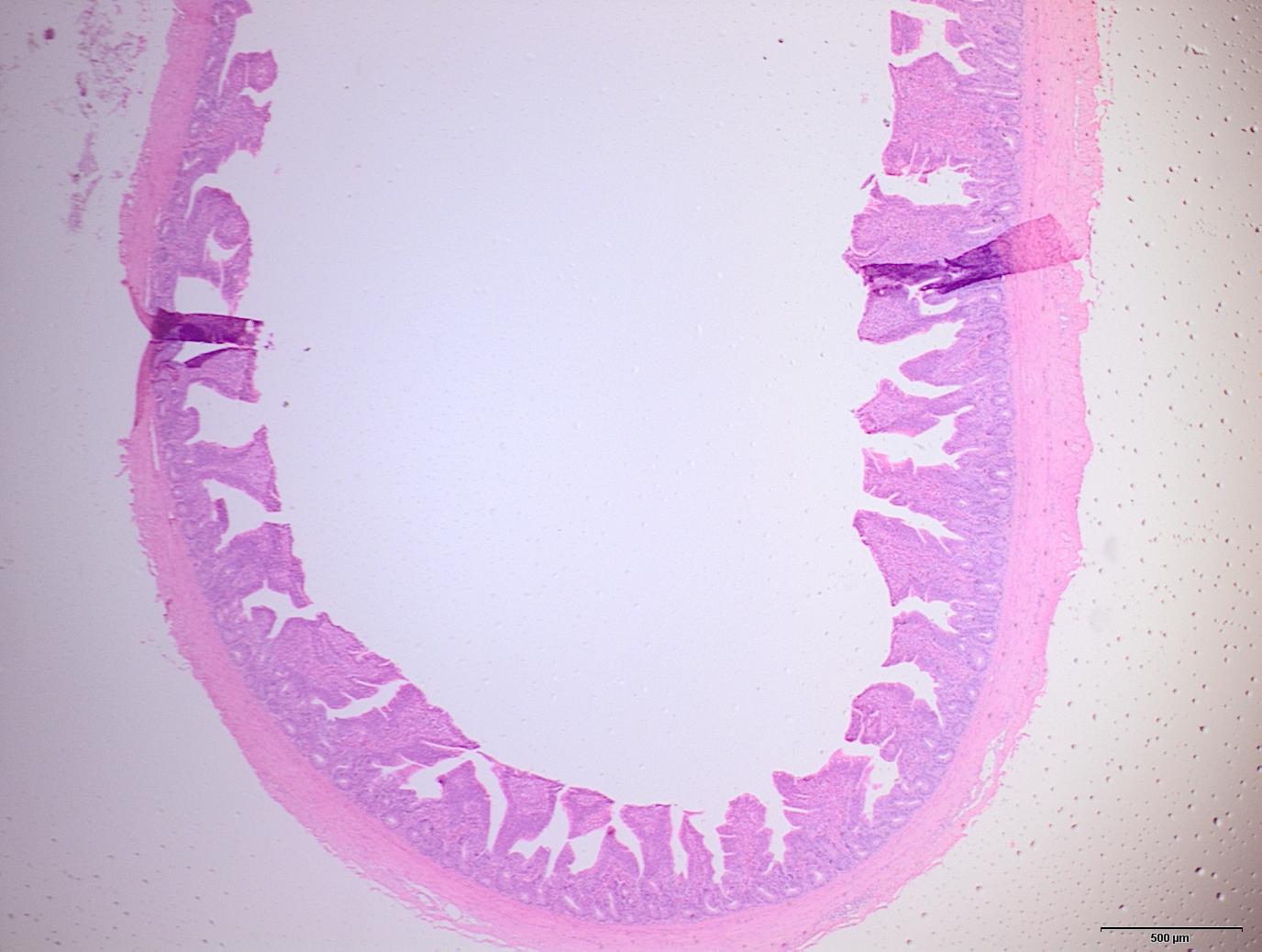

Supplement: Supplementary file 6 [file Data_Sheet_1.ZIP › Data sheet/Hematoxylin-eosin Staining/Ileum/NE+TA400 group/1.jpg]

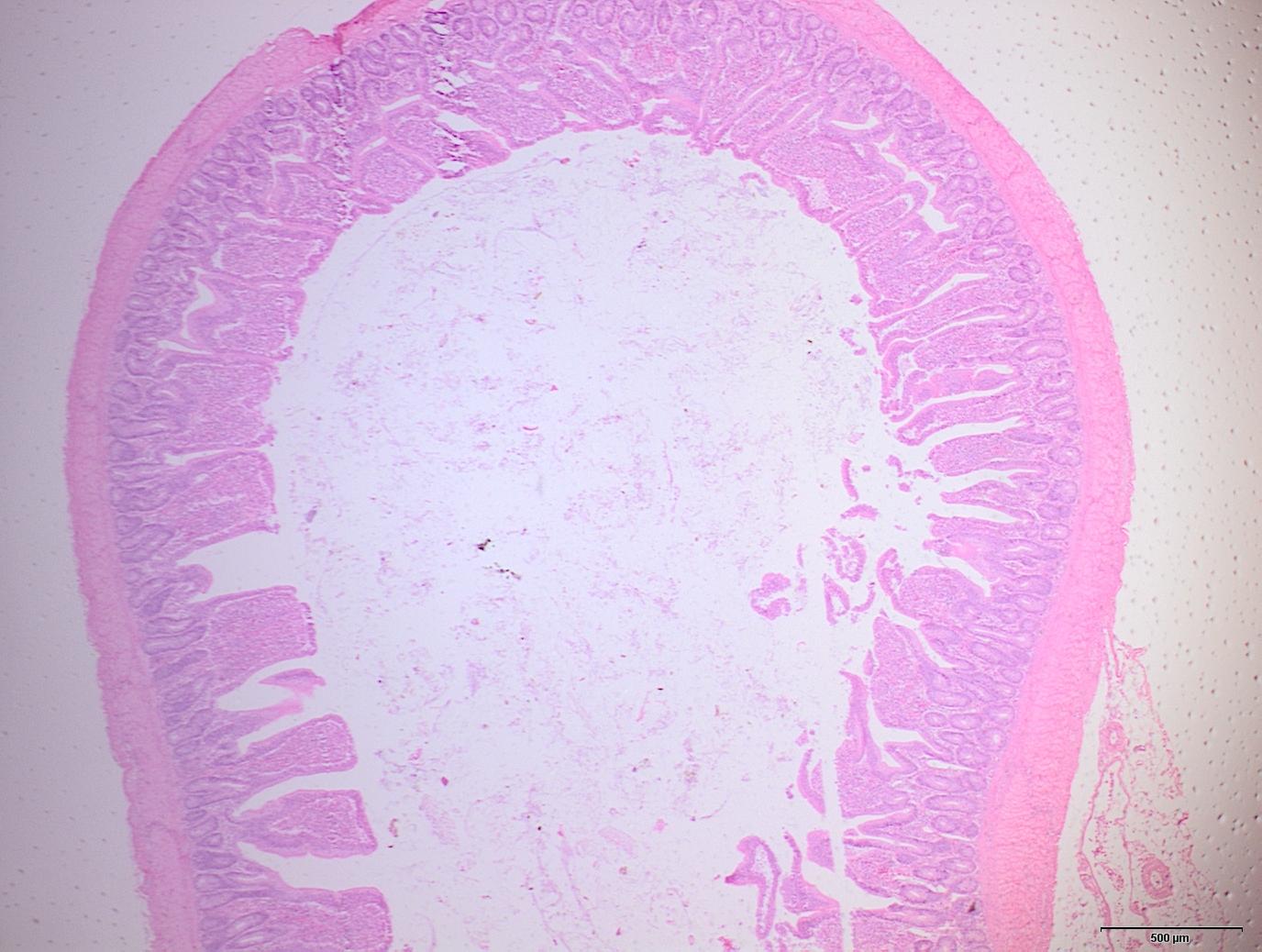

Supplement: Supplementary file 6 [file Data_Sheet_1.ZIP › Data sheet/Hematoxylin-eosin Staining/Ileum/NE+TA400 group/2.jpg]

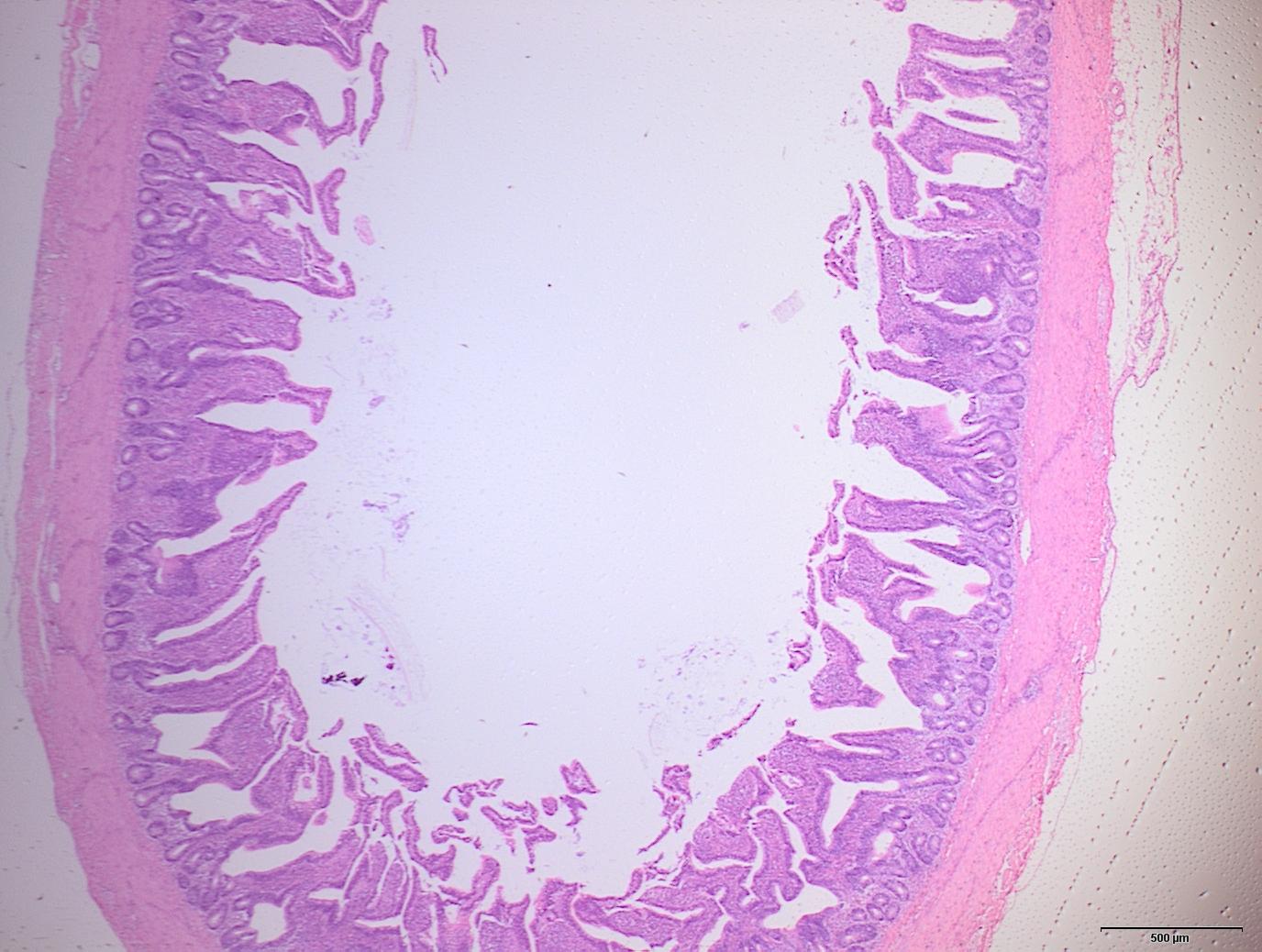

Supplement: Supplementary file 6 [file Data_Sheet_1.ZIP › Data sheet/Hematoxylin-eosin Staining/Ileum/NE+TA400 group/3.jpg]

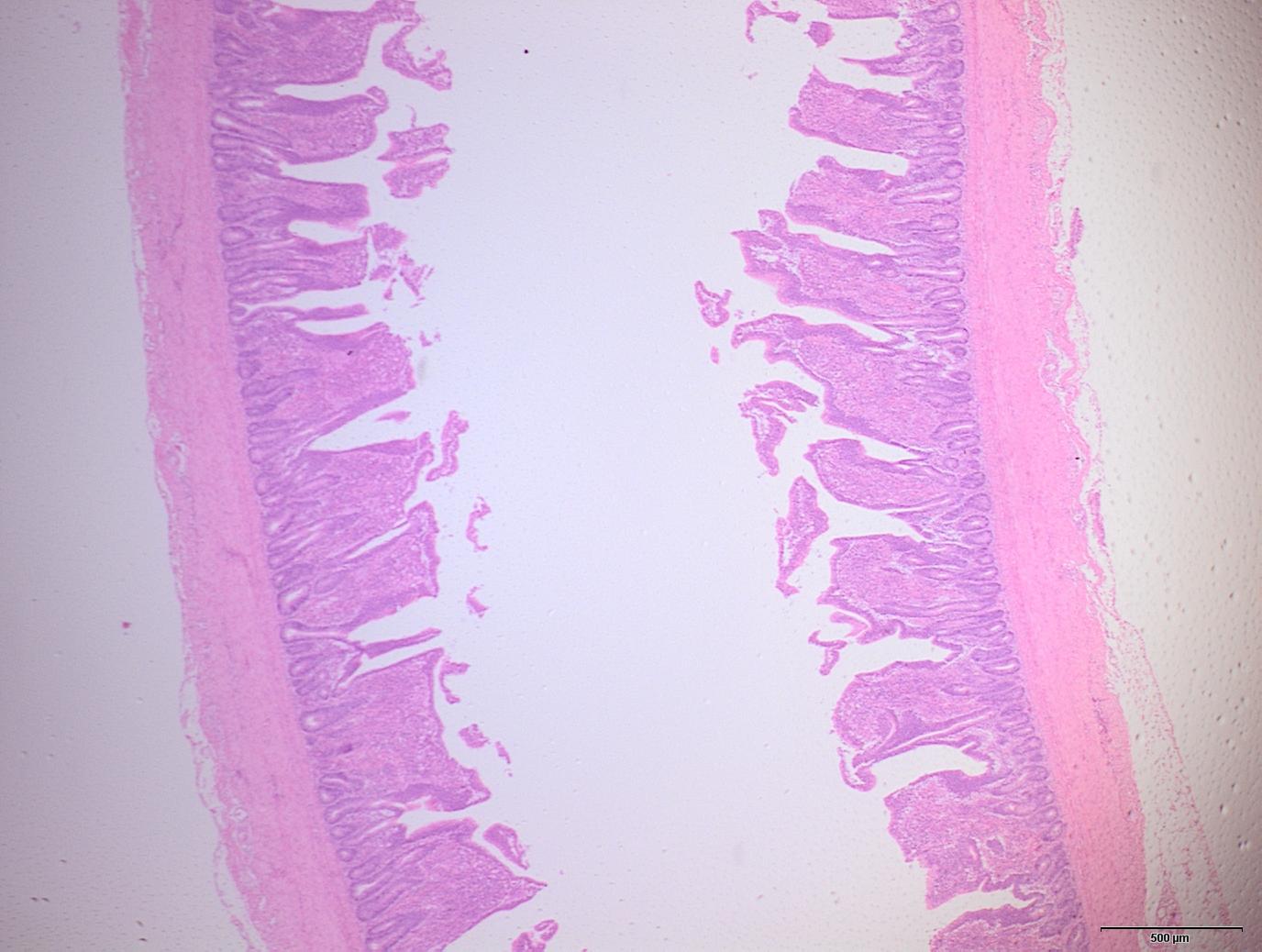

Supplement: Supplementary file 6 [file Data_Sheet_1.ZIP › Data sheet/Hematoxylin-eosin Staining/Ileum/NE+TA400 group/4.jpg]

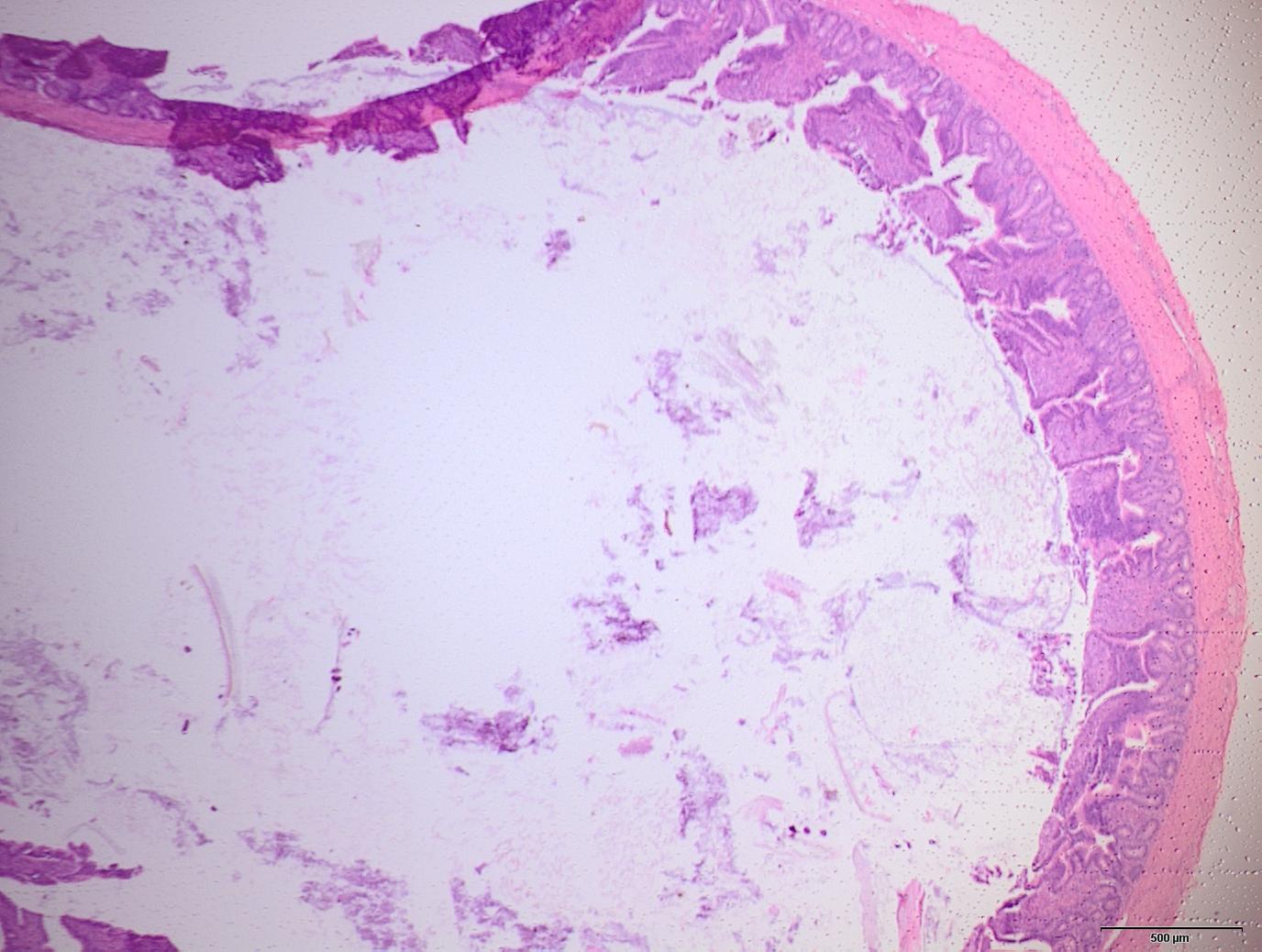

Supplement: Supplementary file 6 [file Data_Sheet_1.ZIP › Data sheet/Hematoxylin-eosin Staining/Ileum/NE+TA400 group/5.jpg]

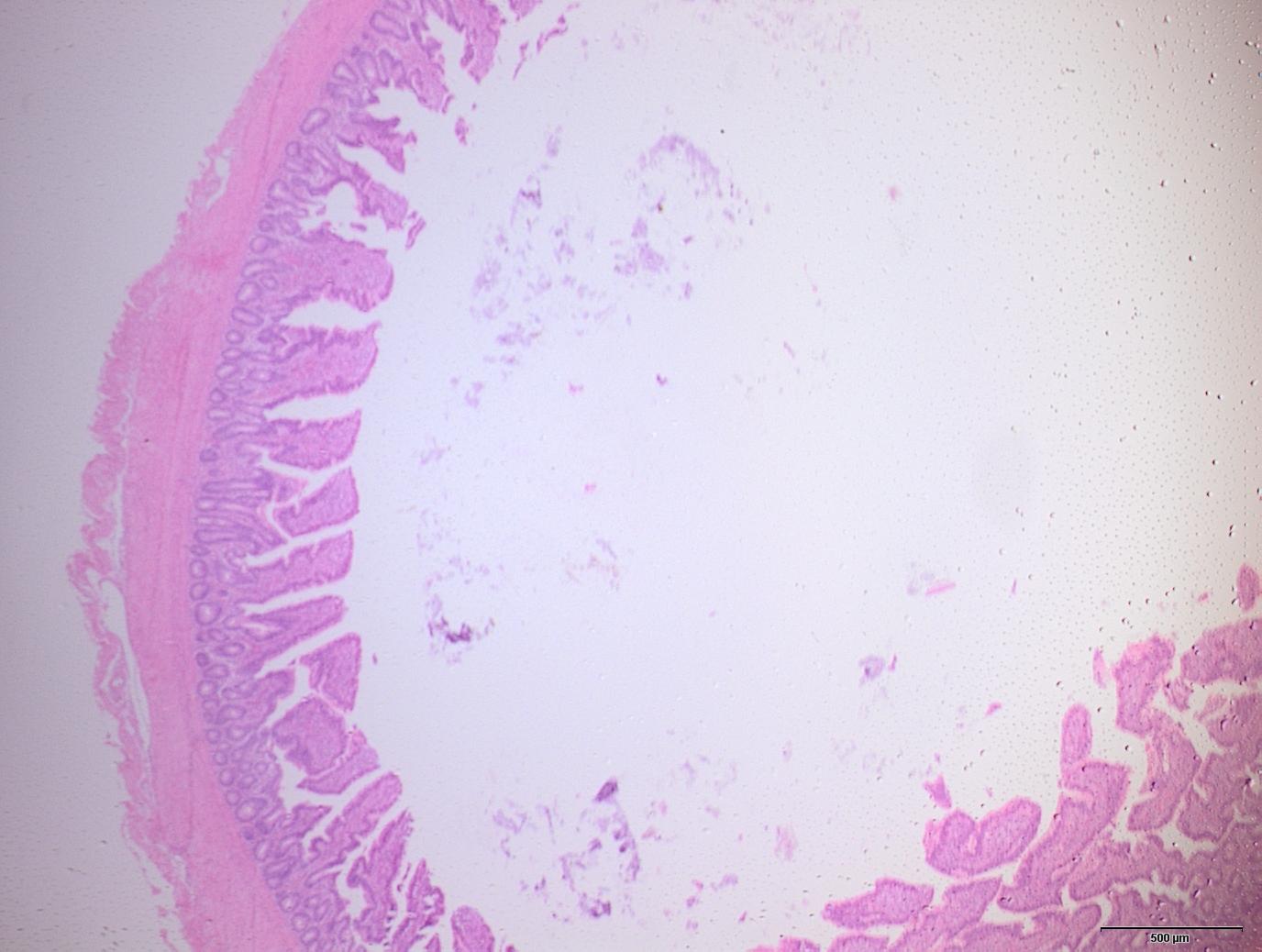

Supplement: Supplementary file 6 [file Data_Sheet_1.ZIP › Data sheet/Hematoxylin-eosin Staining/Ileum/NE+TA400 group/6.jpg]

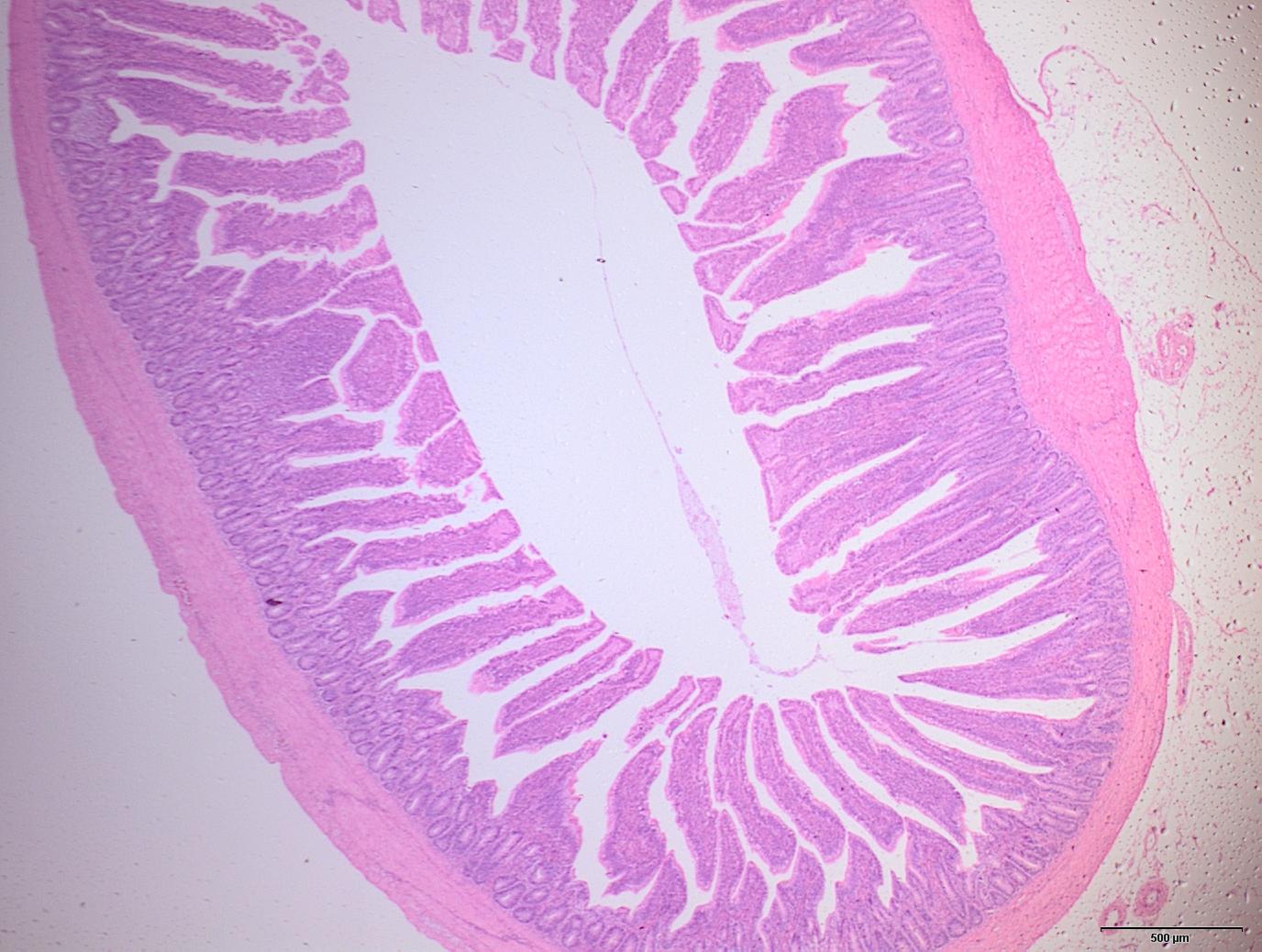

Supplement: Supplementary file 6 [file Data_Sheet_1.ZIP › Data sheet/Hematoxylin-eosin Staining/Ileum/NE+TA400 group/7.jpg]

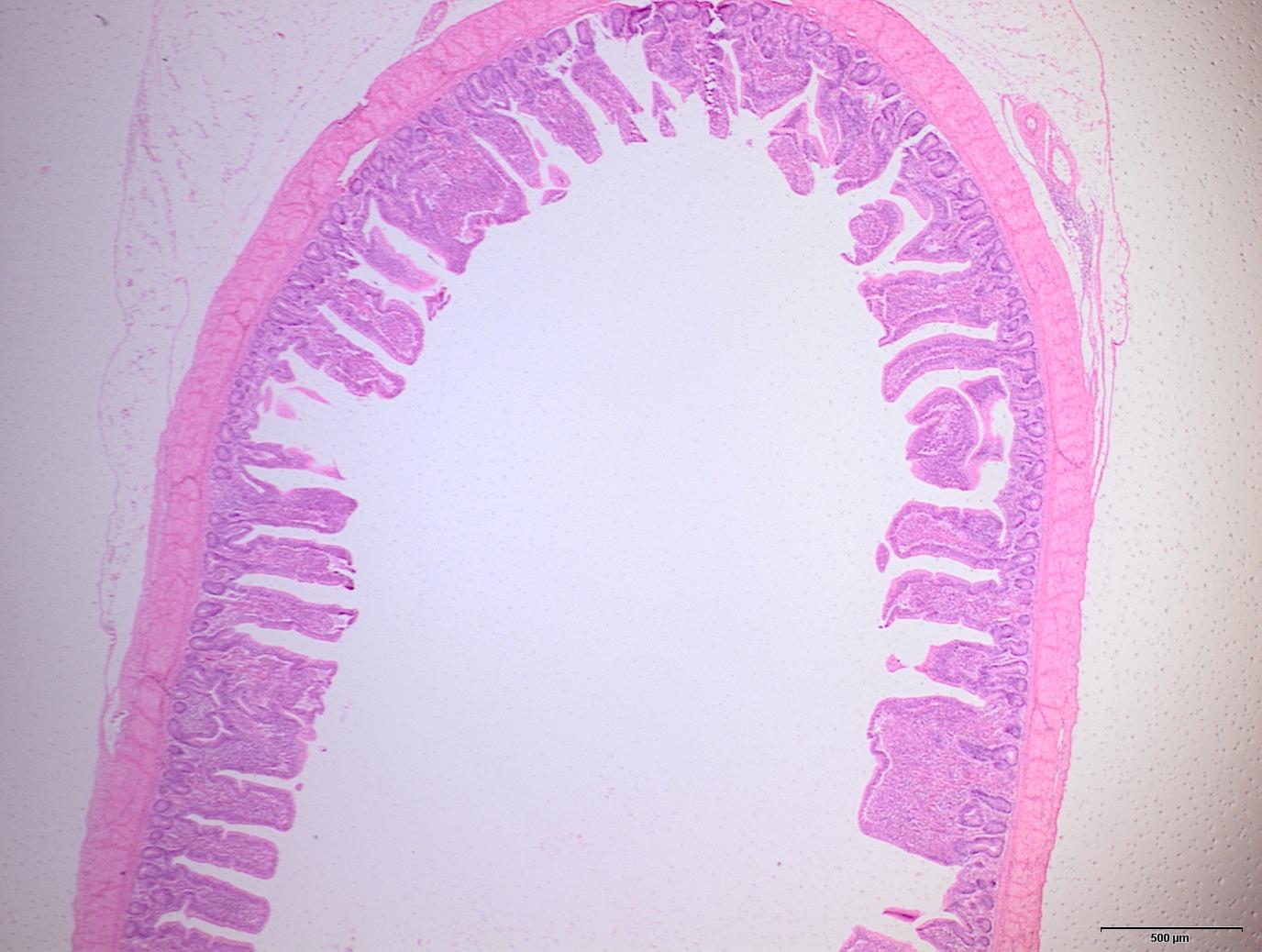

Supplement: Supplementary file 6 [file Data_Sheet_1.ZIP › Data sheet/Hematoxylin-eosin Staining/Ileum/NE+TA400 group/8.jpg]

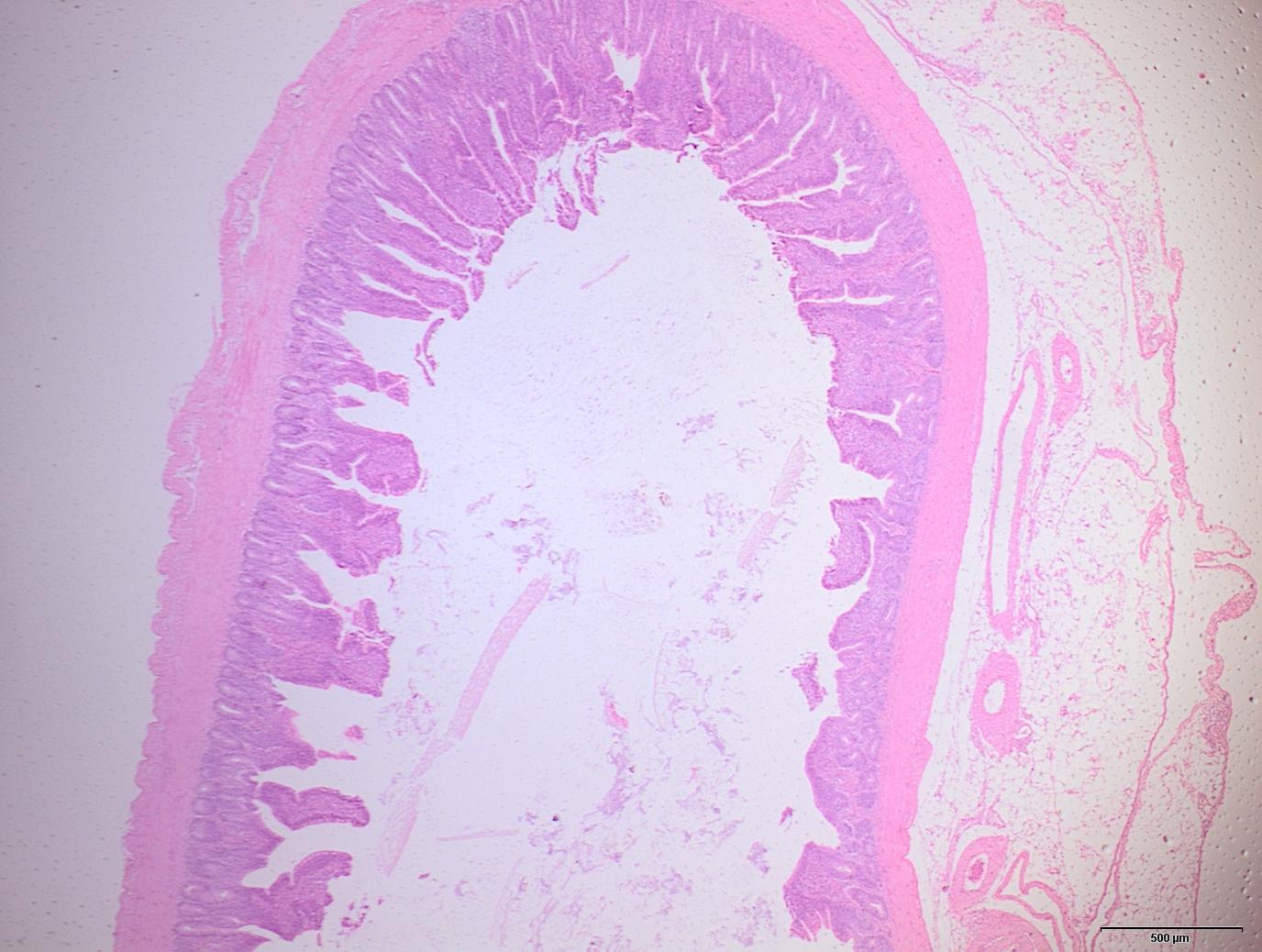

Supplement: Supplementary file 6 [file Data_Sheet_1.ZIP › Data sheet/Hematoxylin-eosin Staining/Ileum/NE+TA600 group/1.jpg]

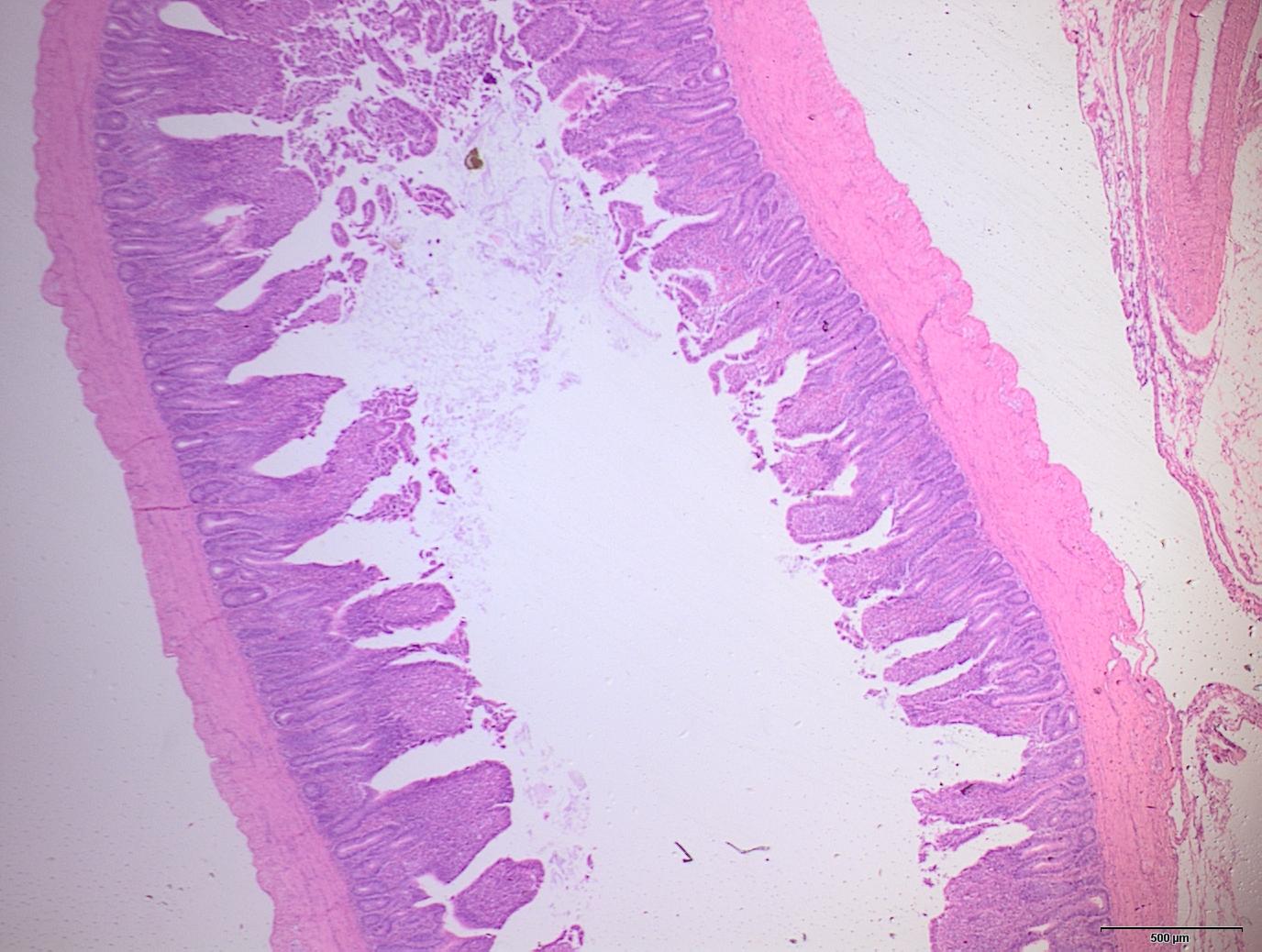

Supplement: Supplementary file 6 [file Data_Sheet_1.ZIP › Data sheet/Hematoxylin-eosin Staining/Ileum/NE+TA600 group/2.jpg]

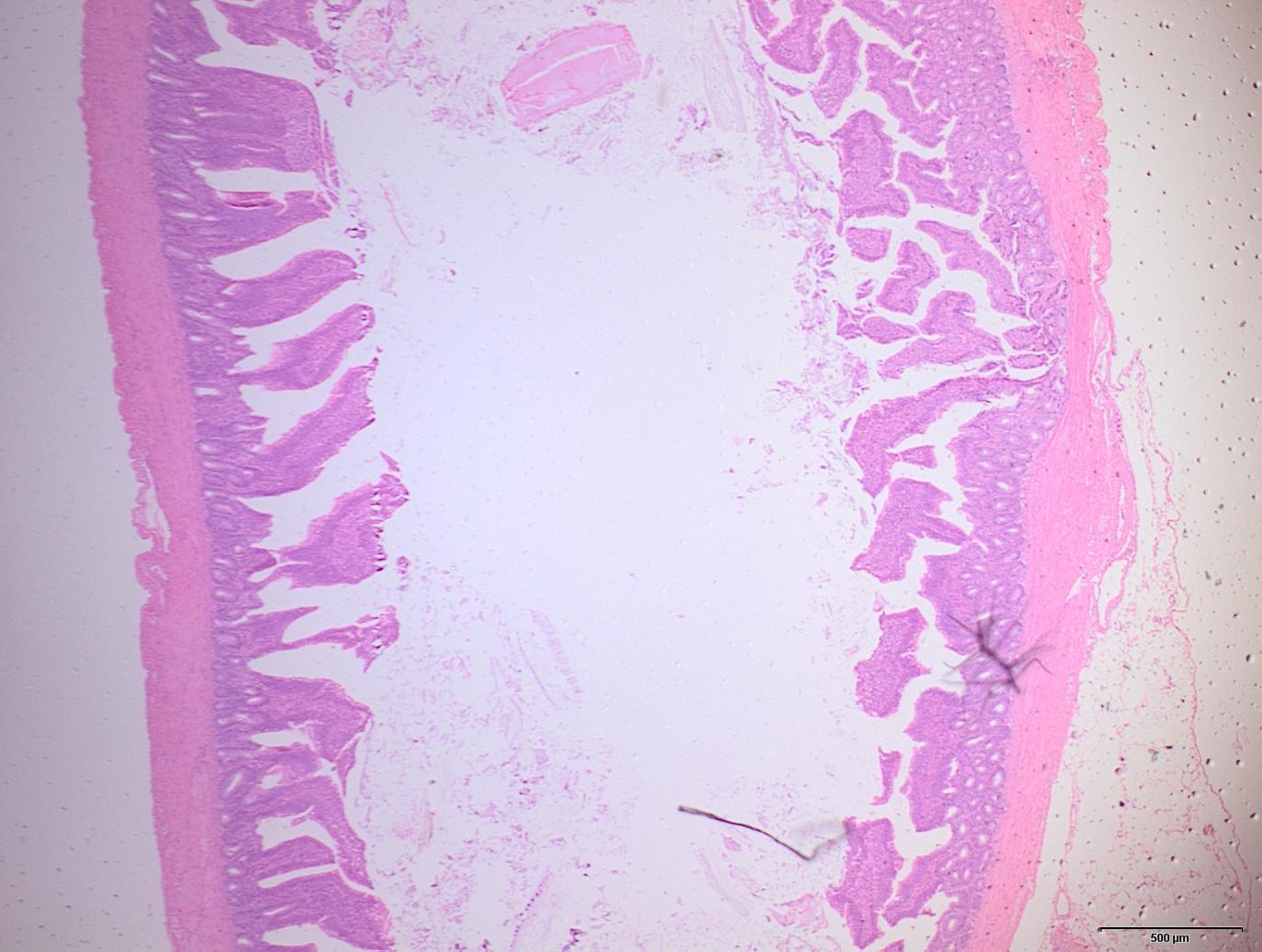

Supplement: Supplementary file 6 [file Data_Sheet_1.ZIP › Data sheet/Hematoxylin-eosin Staining/Ileum/NE+TA600 group/3.jpg]

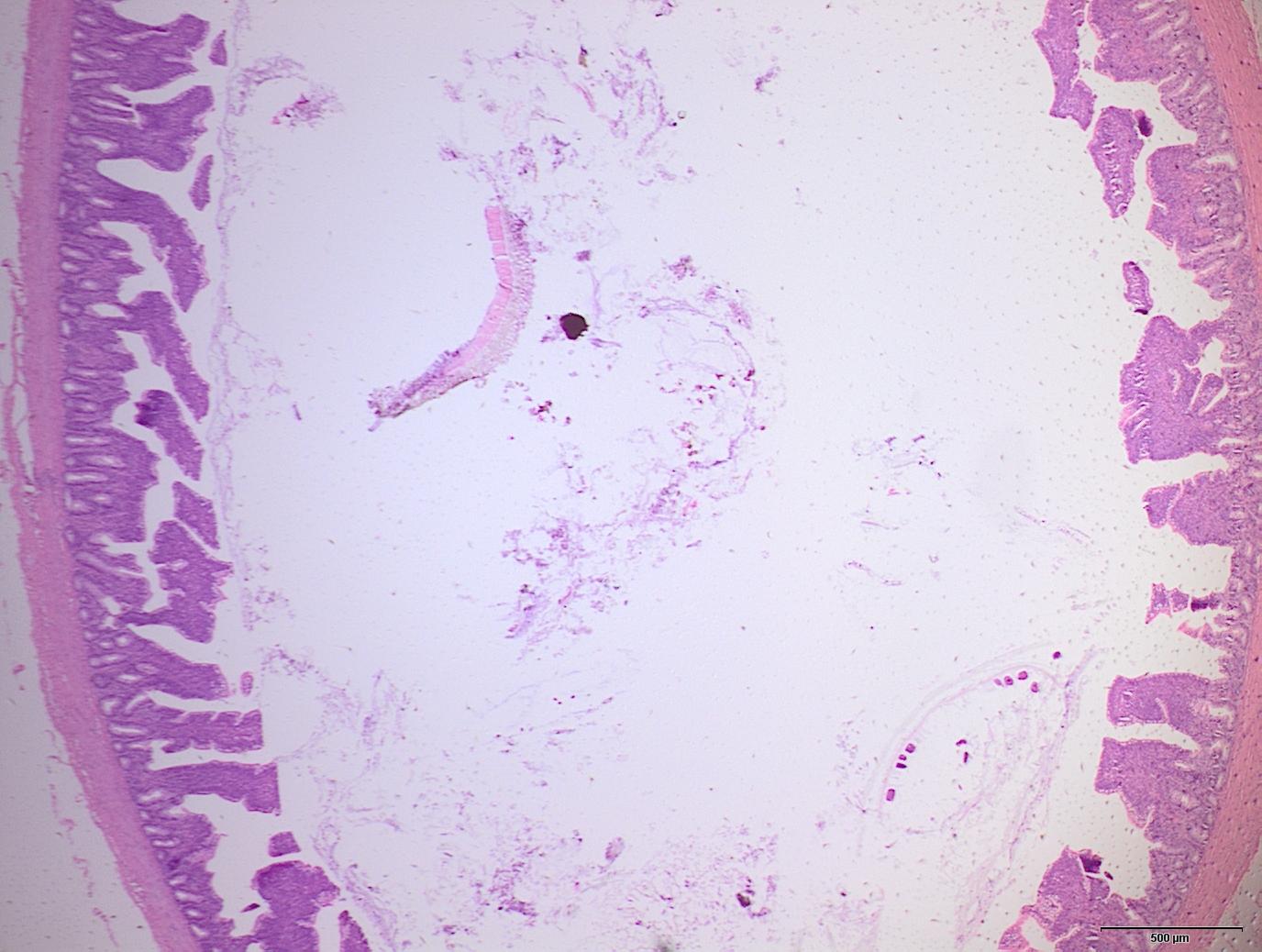

Supplement: Supplementary file 6 [file Data_Sheet_1.ZIP › Data sheet/Hematoxylin-eosin Staining/Ileum/NE+TA600 group/4.jpg]

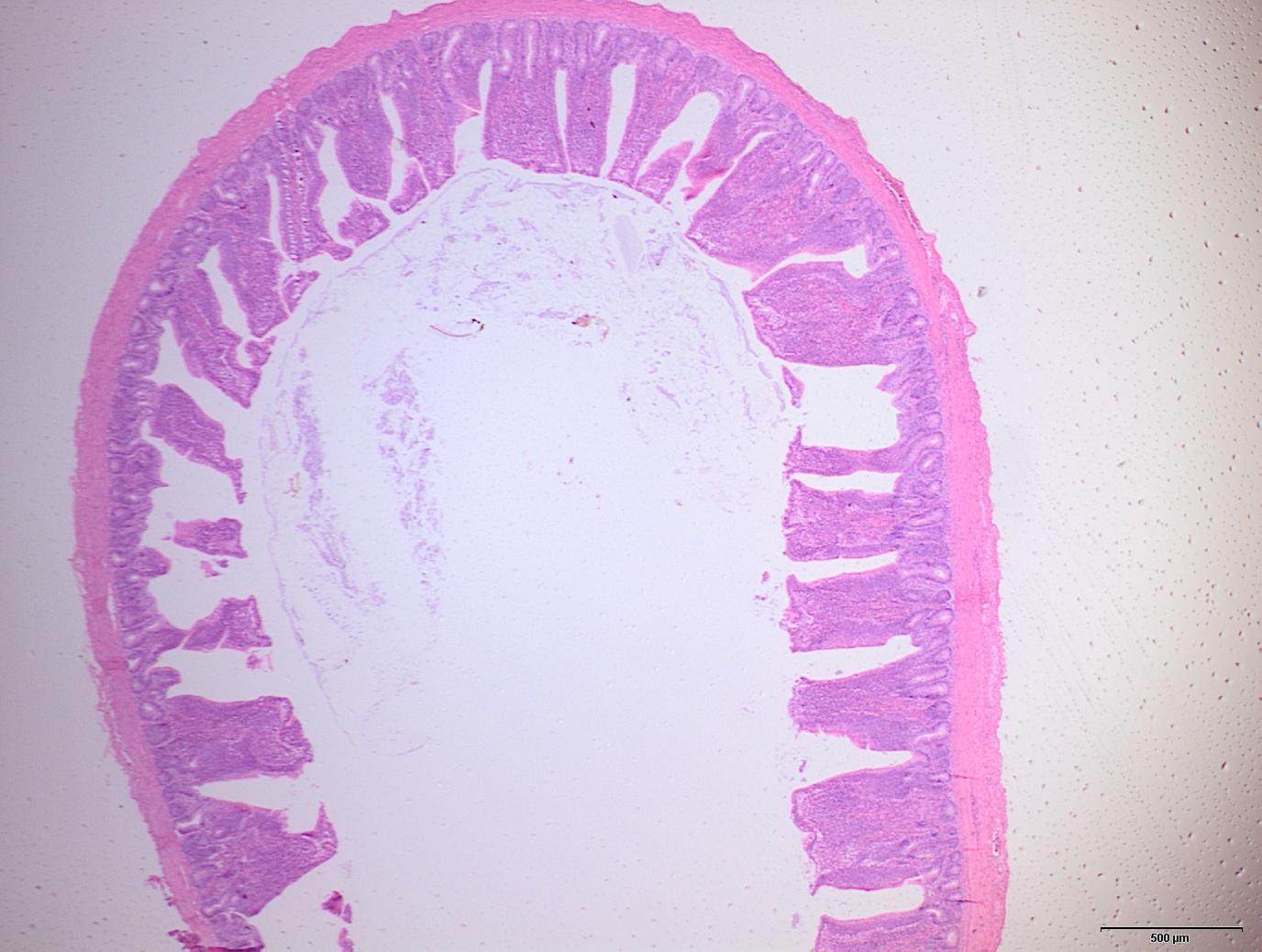

Supplement: Supplementary file 6 [file Data_Sheet_1.ZIP › Data sheet/Hematoxylin-eosin Staining/Ileum/NE+TA600 group/5.jpg]

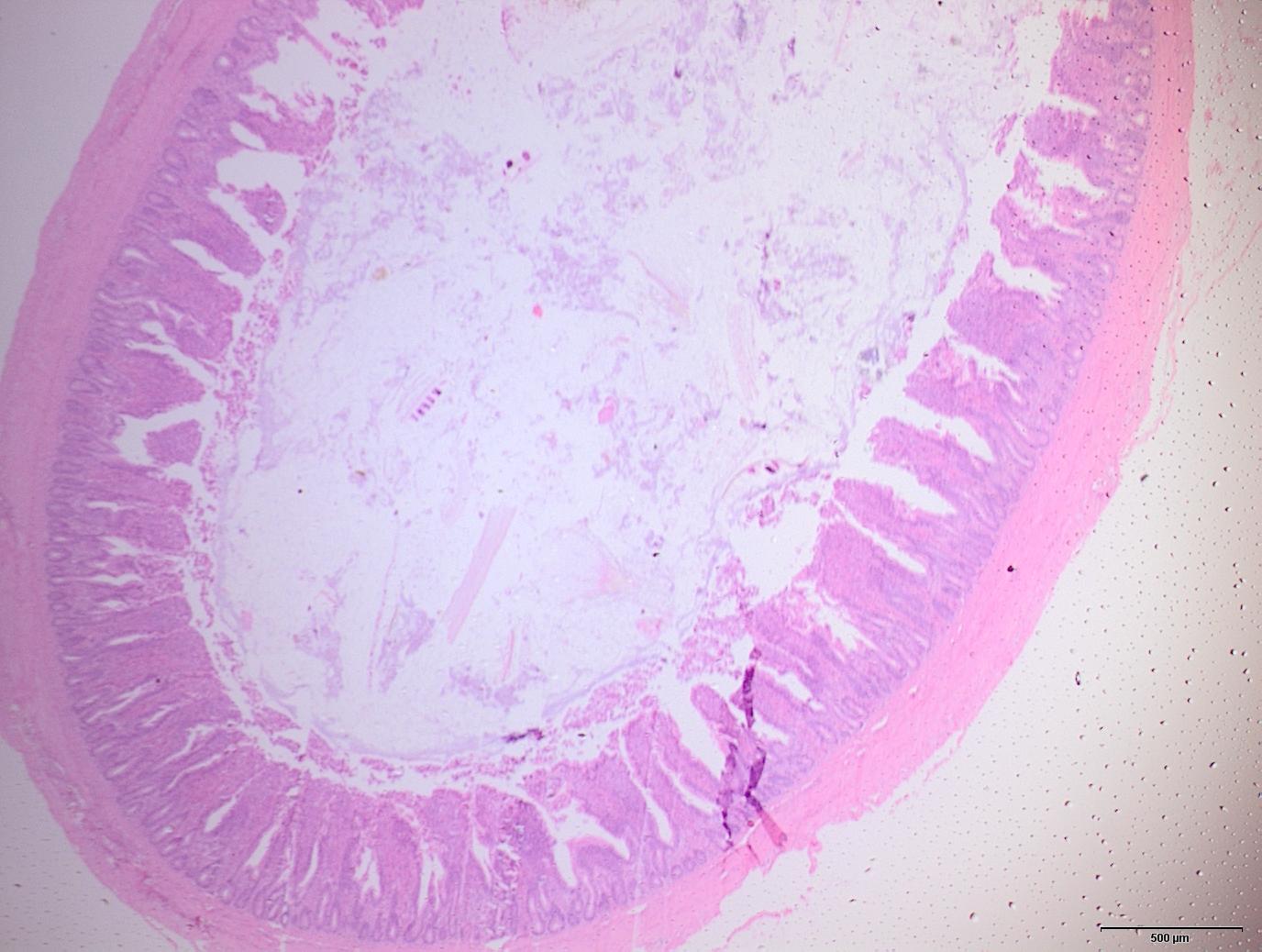

Supplement: Supplementary file 6 [file Data_Sheet_1.ZIP › Data sheet/Hematoxylin-eosin Staining/Ileum/NE+TA600 group/6.jpg]

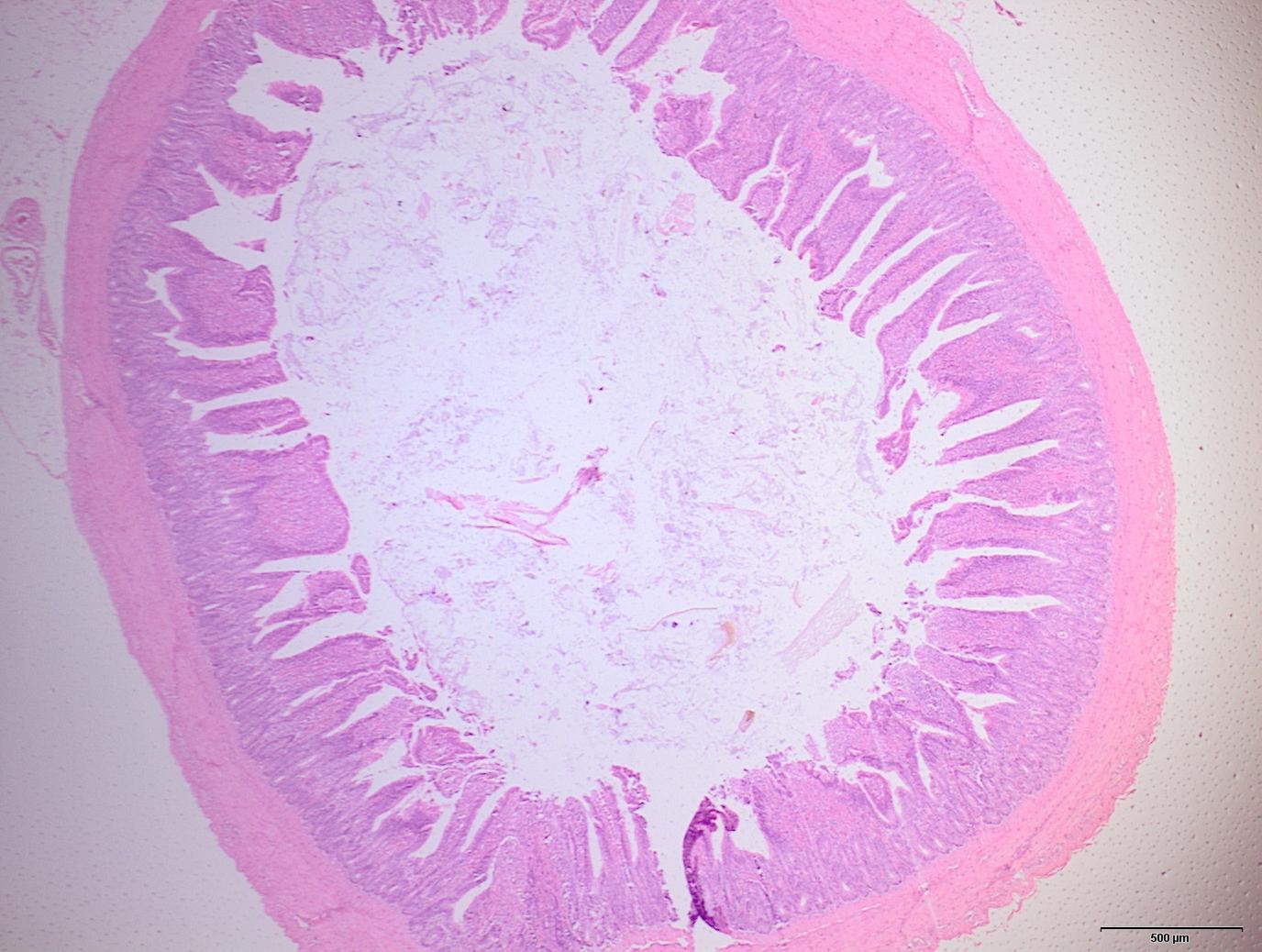

Supplement: Supplementary file 6 [file Data_Sheet_1.ZIP › Data sheet/Hematoxylin-eosin Staining/Ileum/NE+TA600 group/7.jpg]

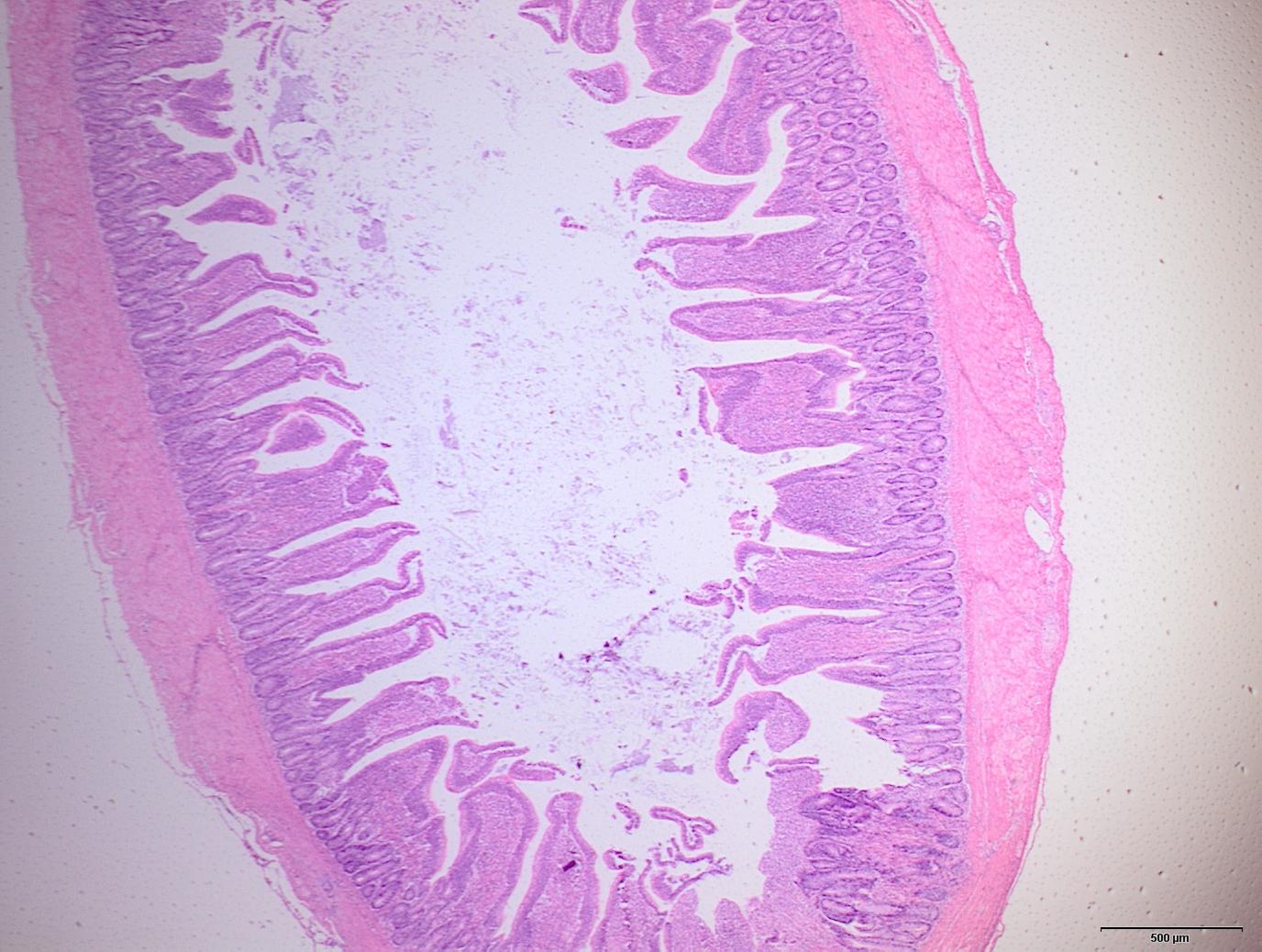

Supplement: Supplementary file 6 [file Data_Sheet_1.ZIP › Data sheet/Hematoxylin-eosin Staining/Ileum/NE+TA600 group/8.jpg]

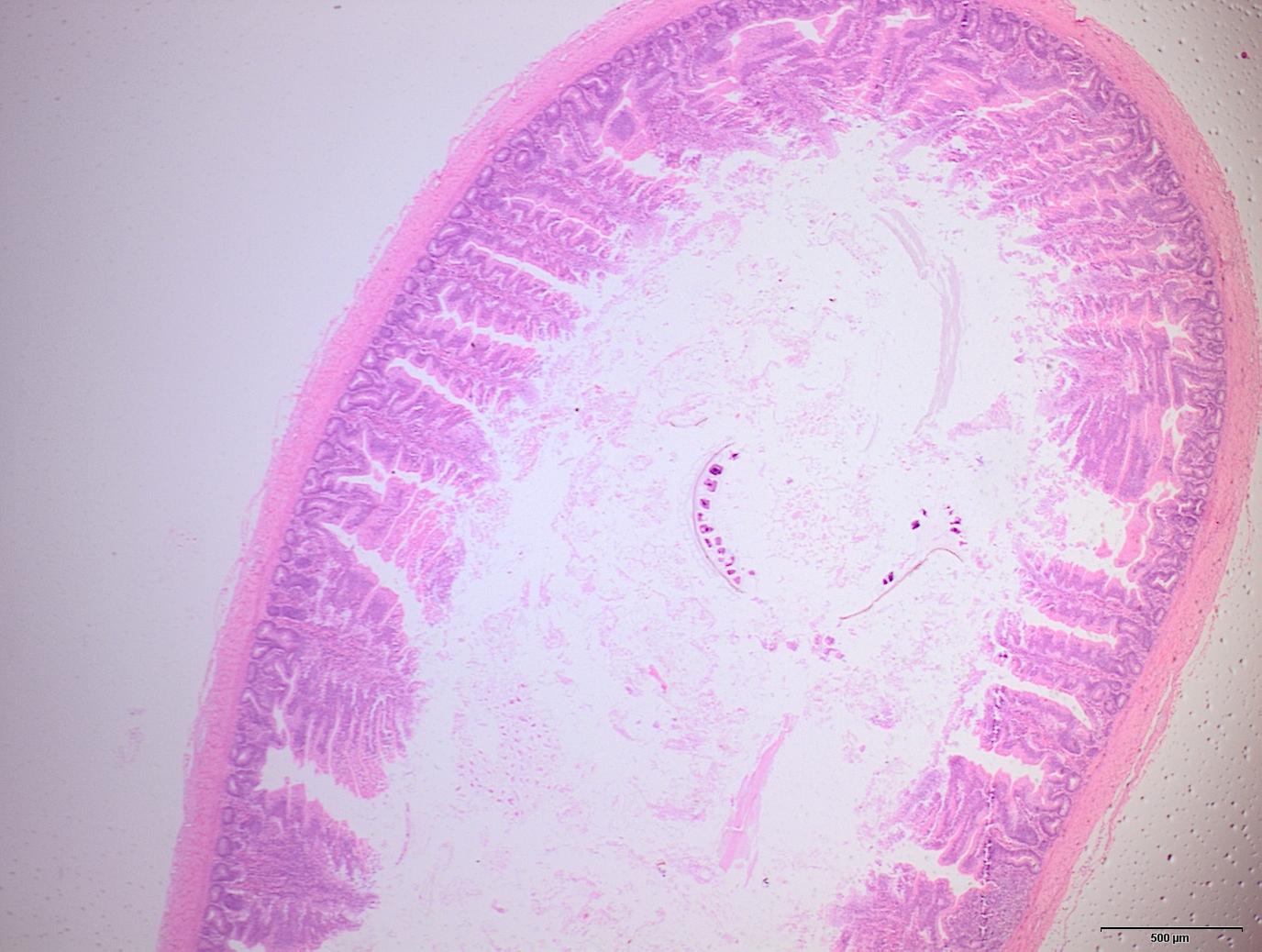

Supplement: Supplementary file 6 [file Data_Sheet_1.ZIP › Data sheet/Hematoxylin-eosin Staining/Jejunum/CON group/1.jpg]

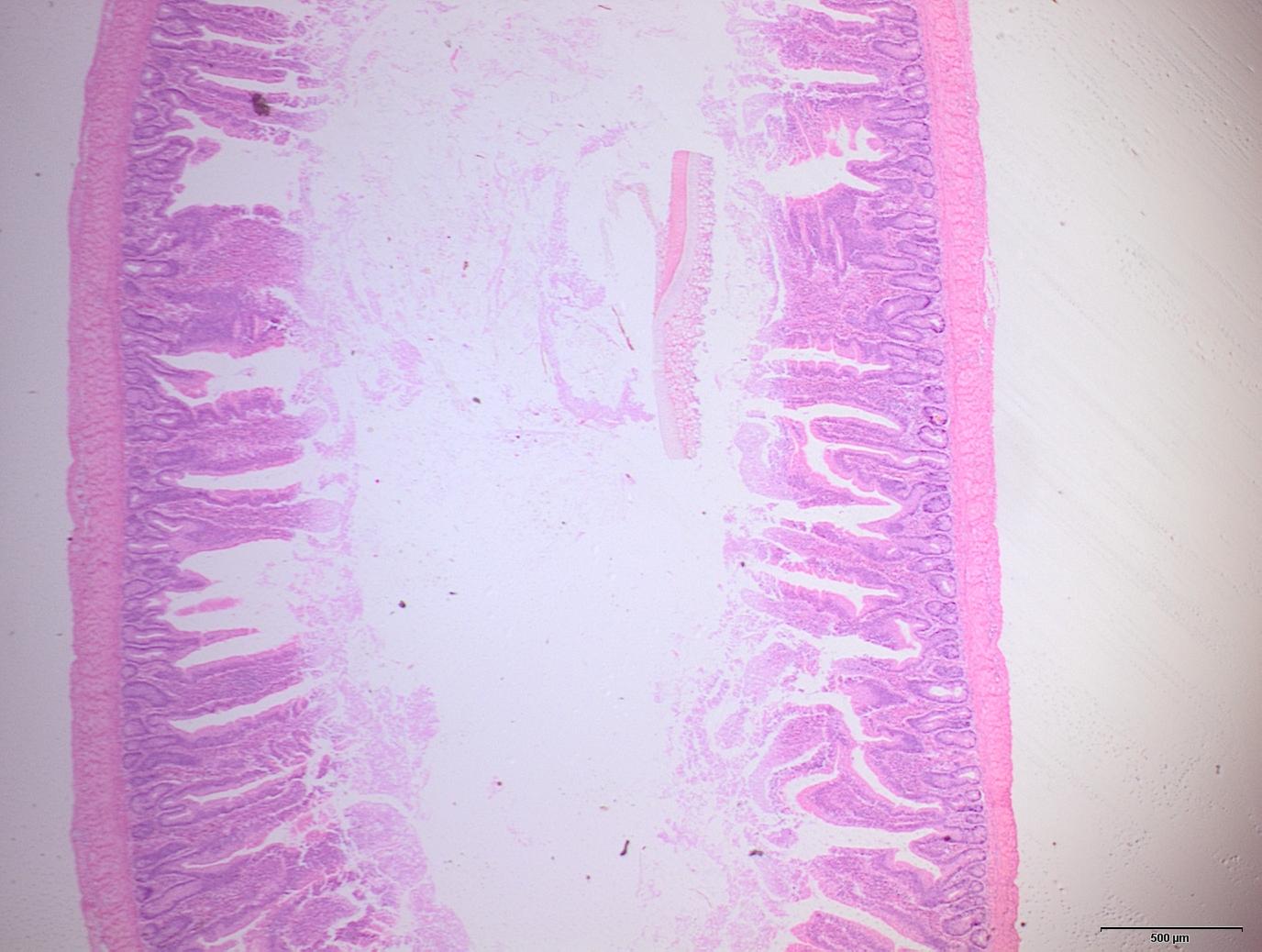

Supplement: Supplementary file 6 [file Data_Sheet_1.ZIP › Data sheet/Hematoxylin-eosin Staining/Jejunum/CON group/2.jpg]

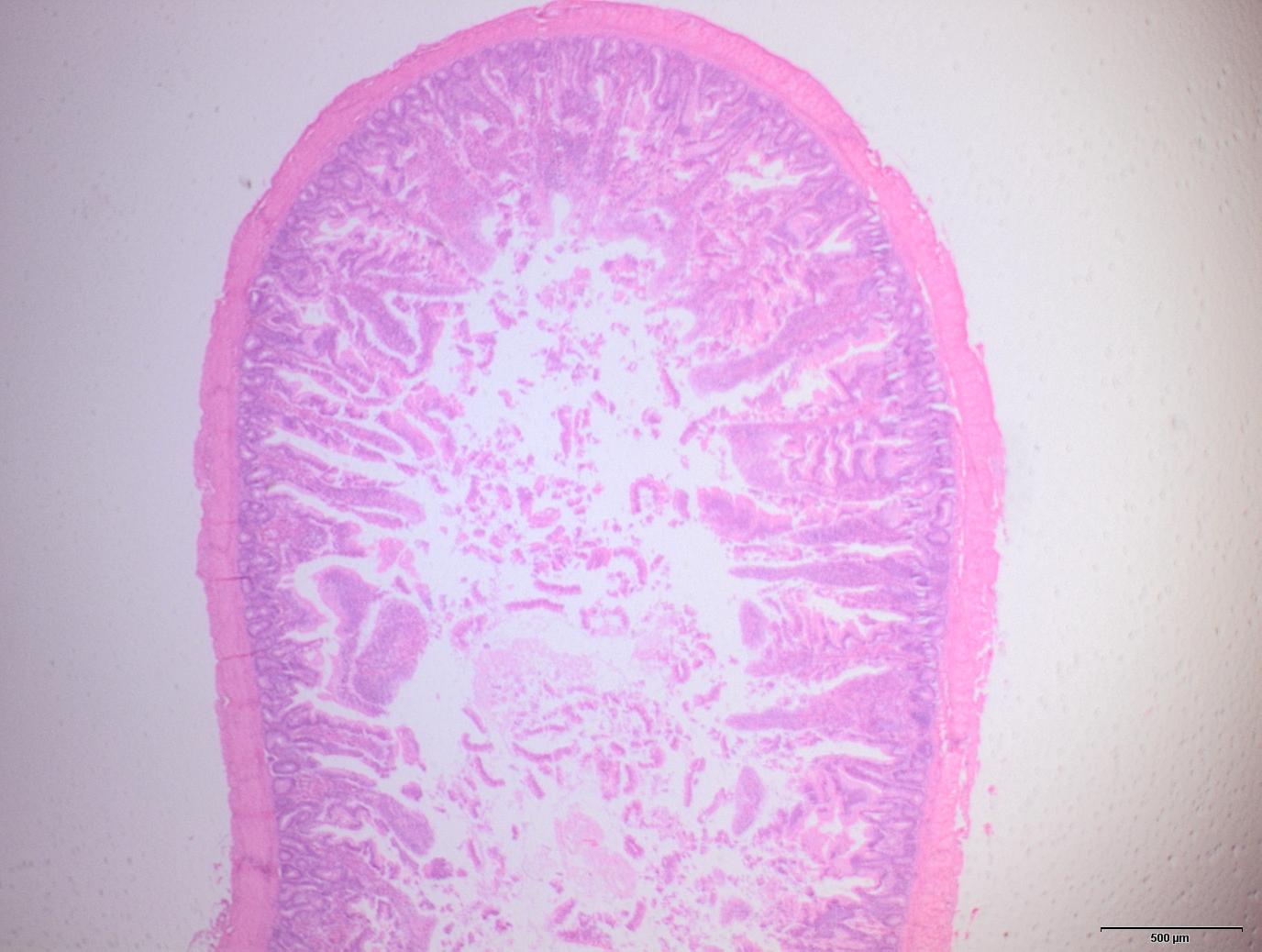

Supplement: Supplementary file 6 [file Data_Sheet_1.ZIP › Data sheet/Hematoxylin-eosin Staining/Jejunum/CON group/3.jpg]

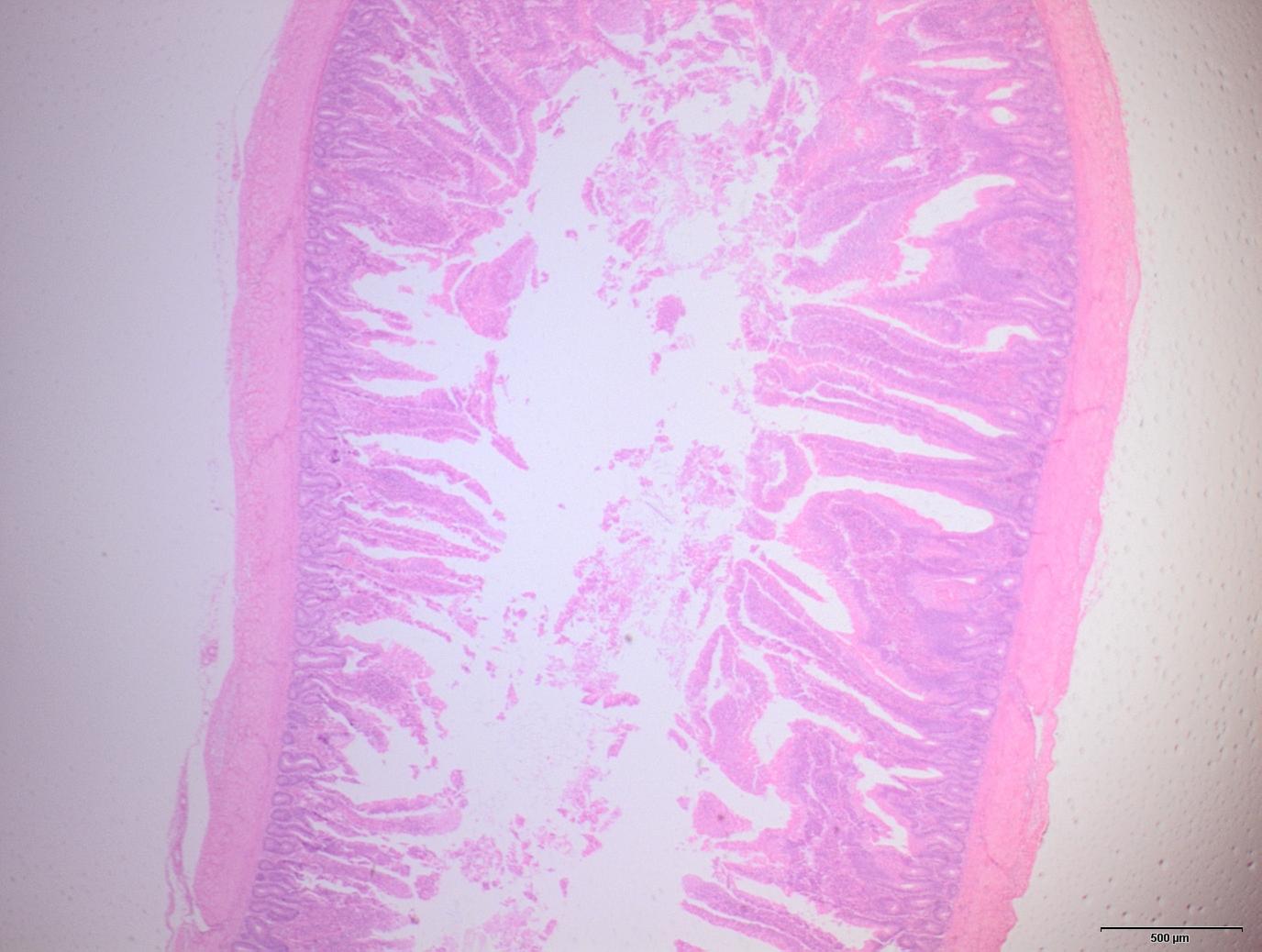

Supplement: Supplementary file 6 [file Data_Sheet_1.ZIP › Data sheet/Hematoxylin-eosin Staining/Jejunum/CON group/4.jpg]

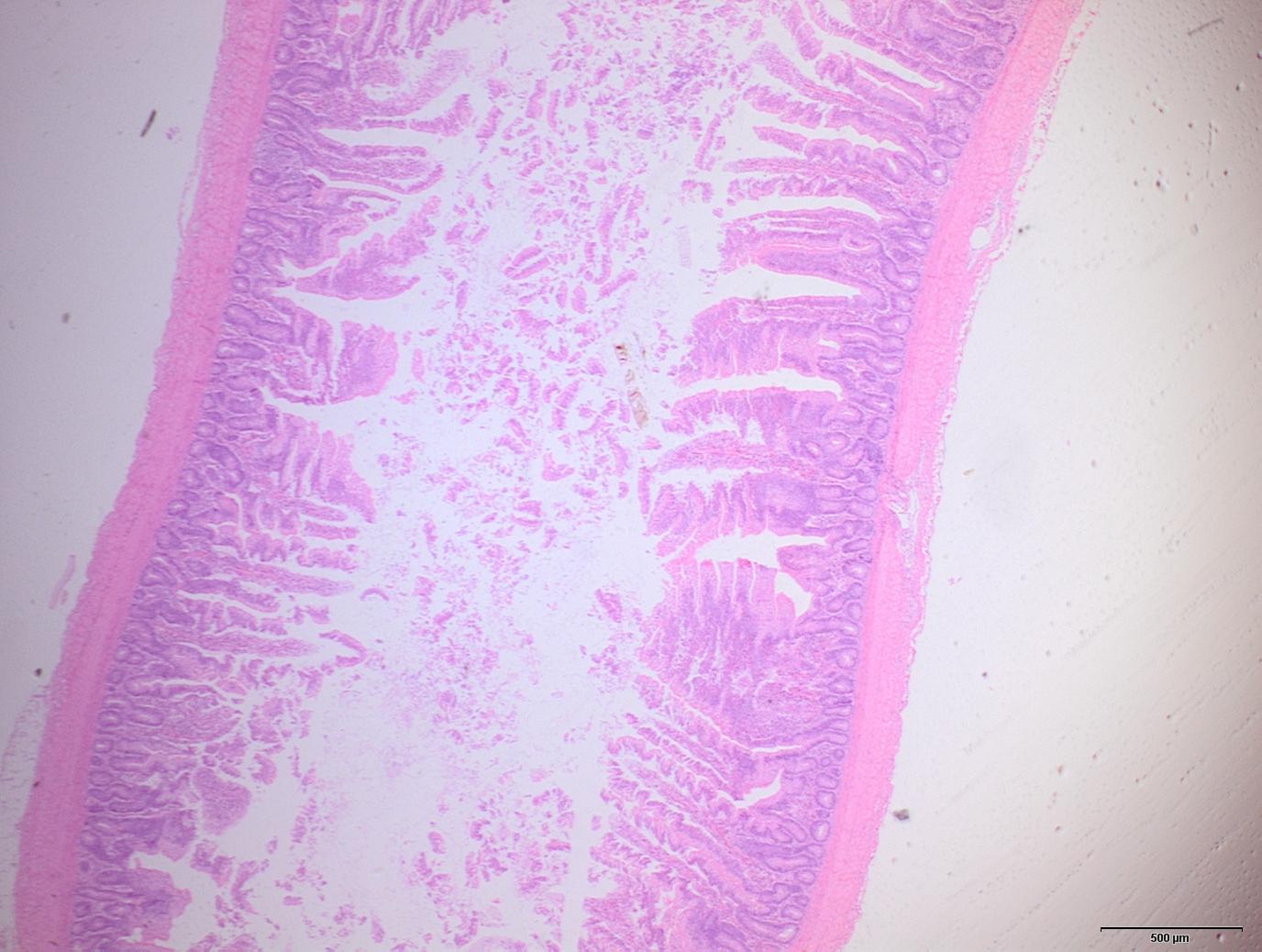

Supplement: Supplementary file 6 [file Data_Sheet_1.ZIP › Data sheet/Hematoxylin-eosin Staining/Jejunum/CON group/5.jpg]

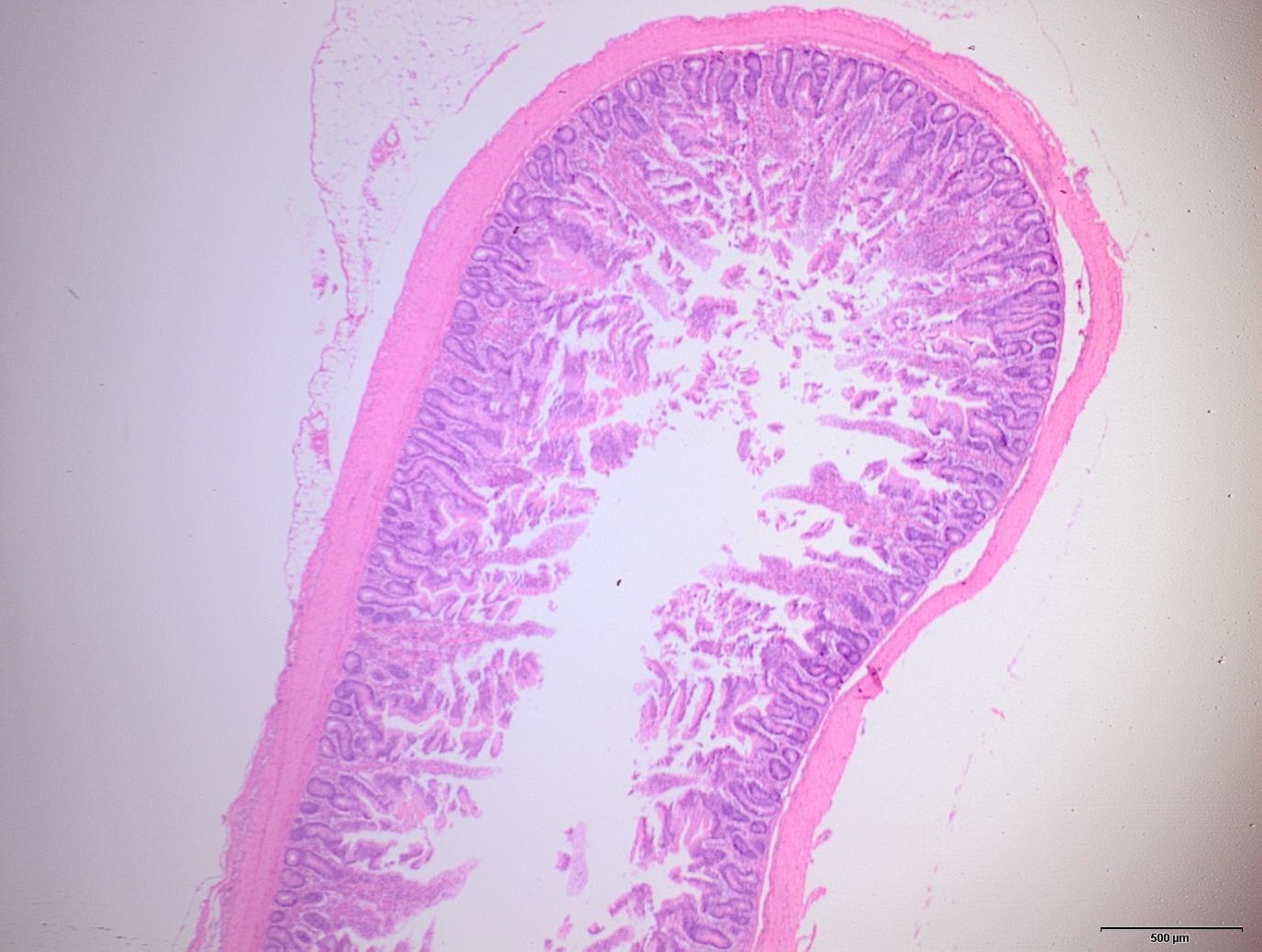

Supplement: Supplementary file 6 [file Data_Sheet_1.ZIP › Data sheet/Hematoxylin-eosin Staining/Jejunum/CON group/6.jpg]

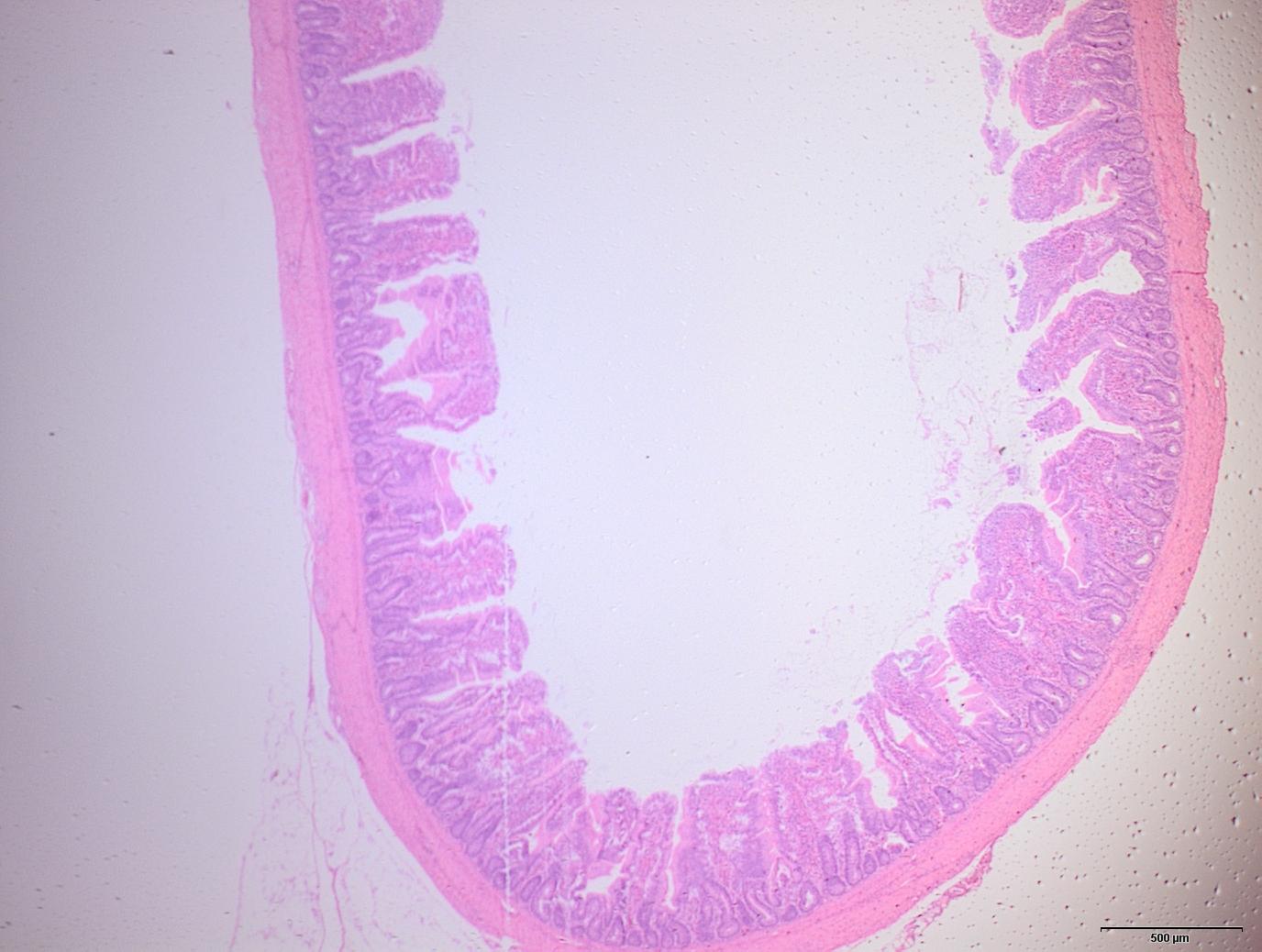

Supplement: Supplementary file 6 [file Data_Sheet_1.ZIP › Data sheet/Hematoxylin-eosin Staining/Jejunum/CON group/7.jpg]

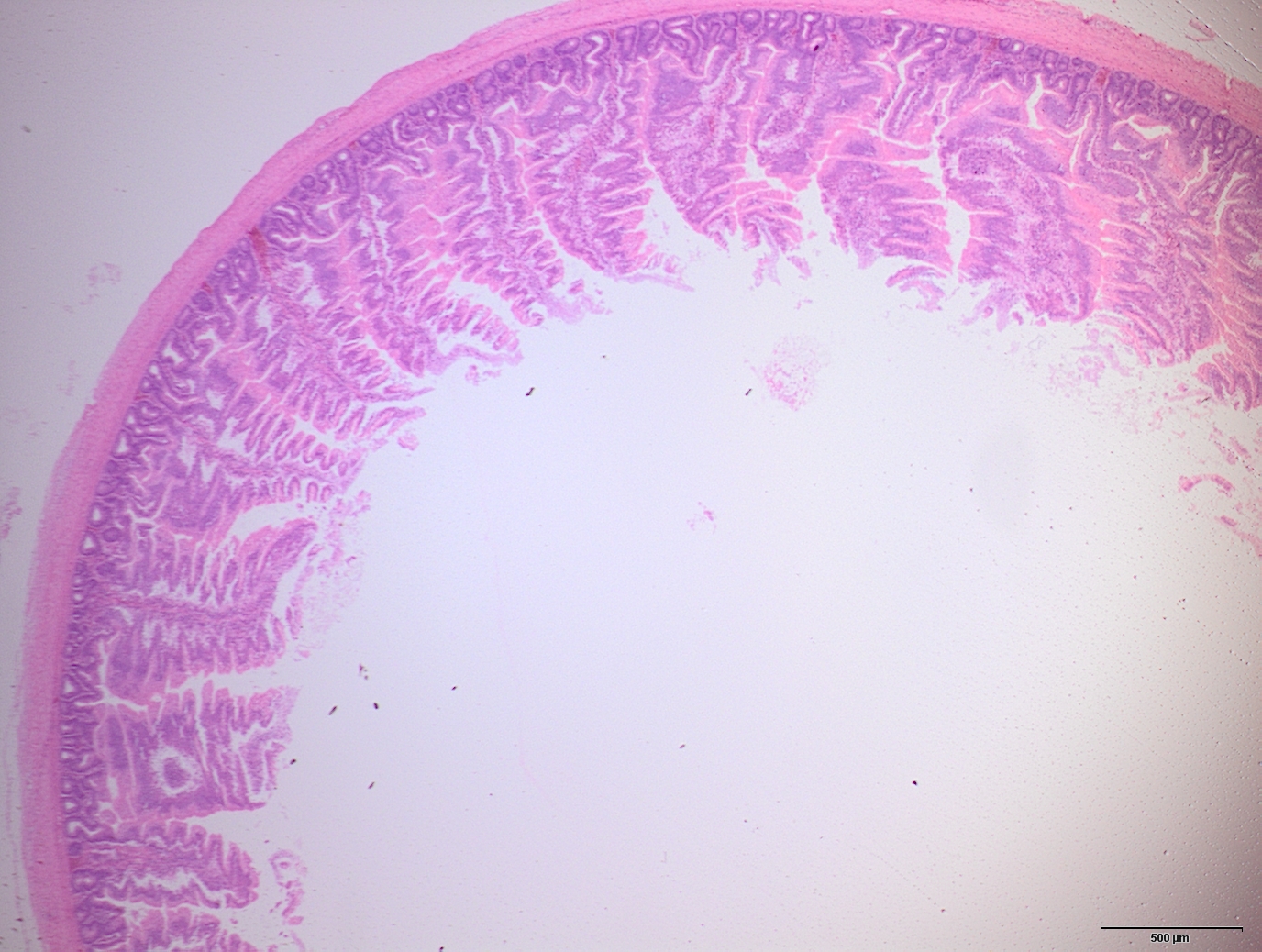

Supplement: Supplementary file 6 [file Data_Sheet_1.ZIP › Data sheet/Hematoxylin-eosin Staining/Jejunum/CON group/8.jpg]

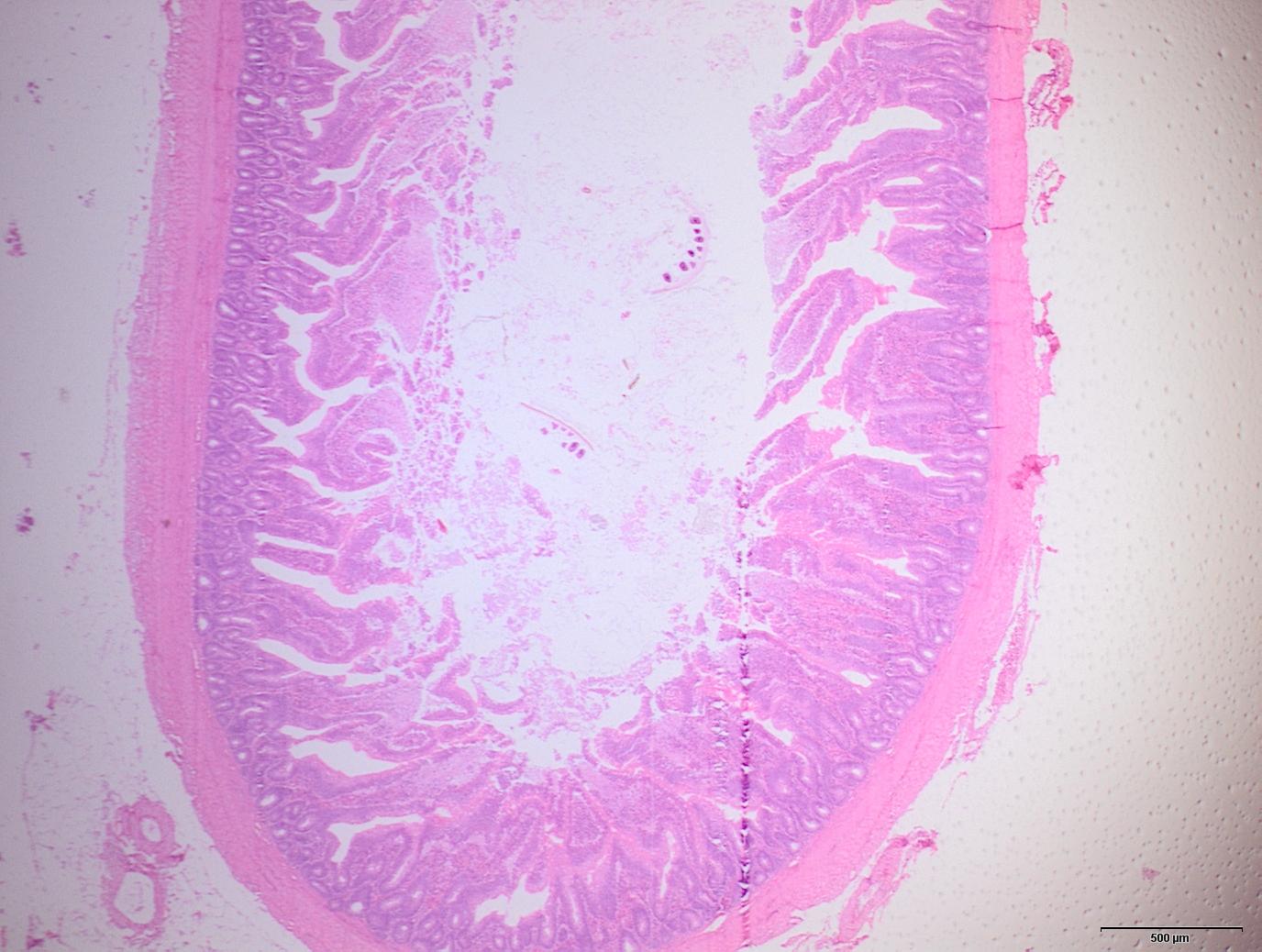

Supplement: Supplementary file 6 [file Data_Sheet_1.ZIP › Data sheet/Hematoxylin-eosin Staining/Jejunum/NE group/1.jpg]

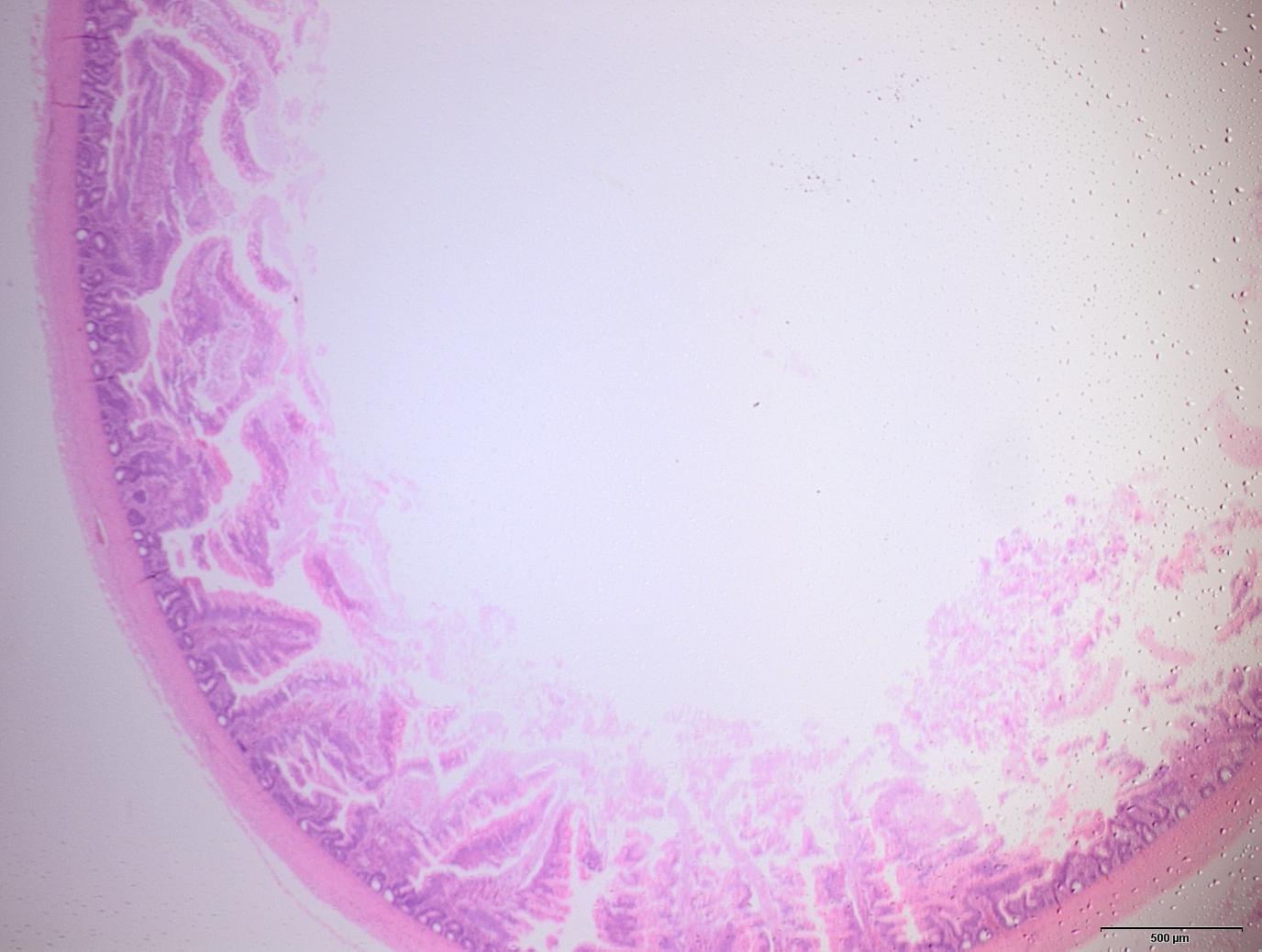

Supplement: Supplementary file 6 [file Data_Sheet_1.ZIP › Data sheet/Hematoxylin-eosin Staining/Jejunum/NE group/2.jpg]

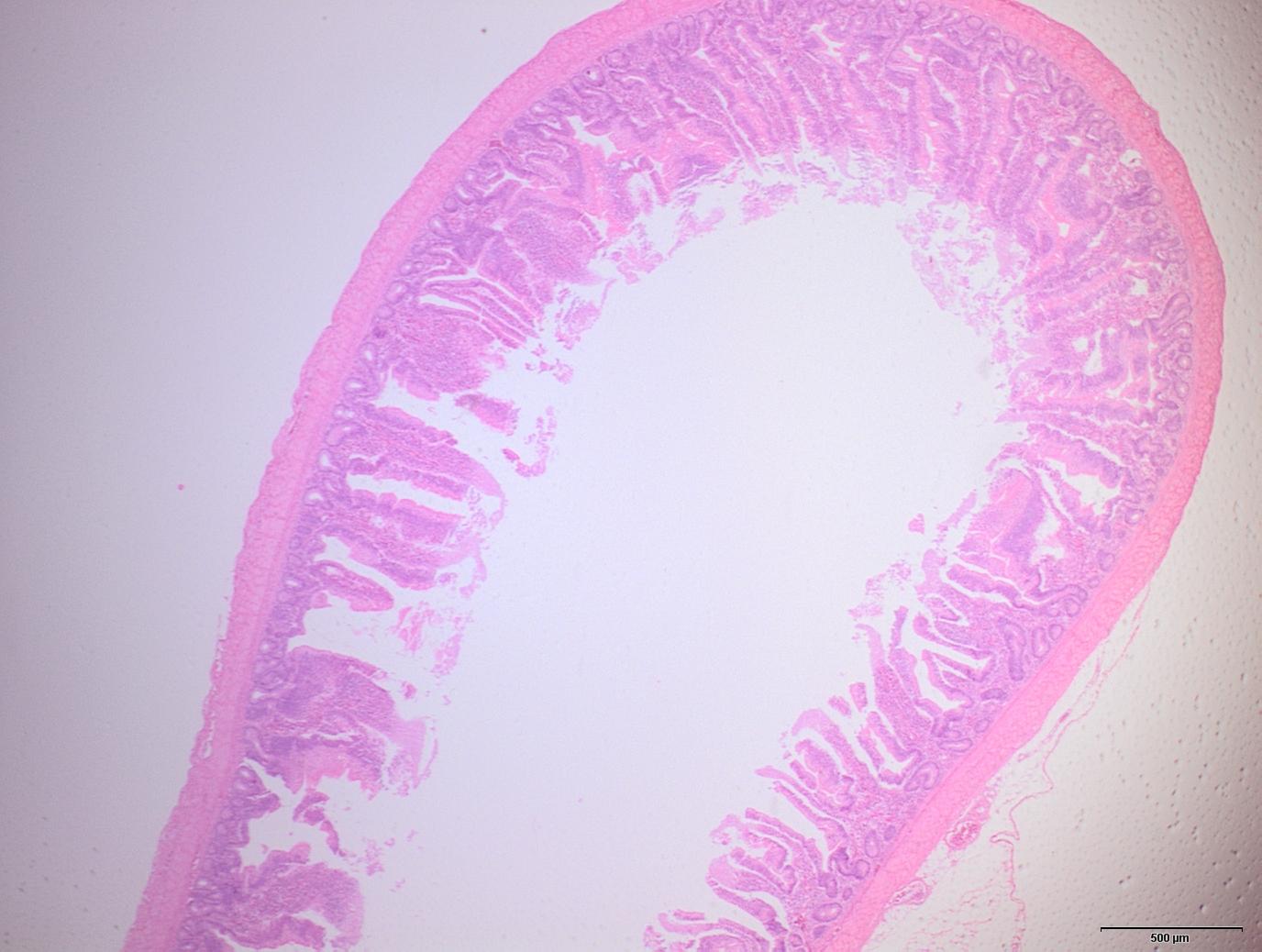

Supplement: Supplementary file 6 [file Data_Sheet_1.ZIP › Data sheet/Hematoxylin-eosin Staining/Jejunum/NE group/3.jpg]

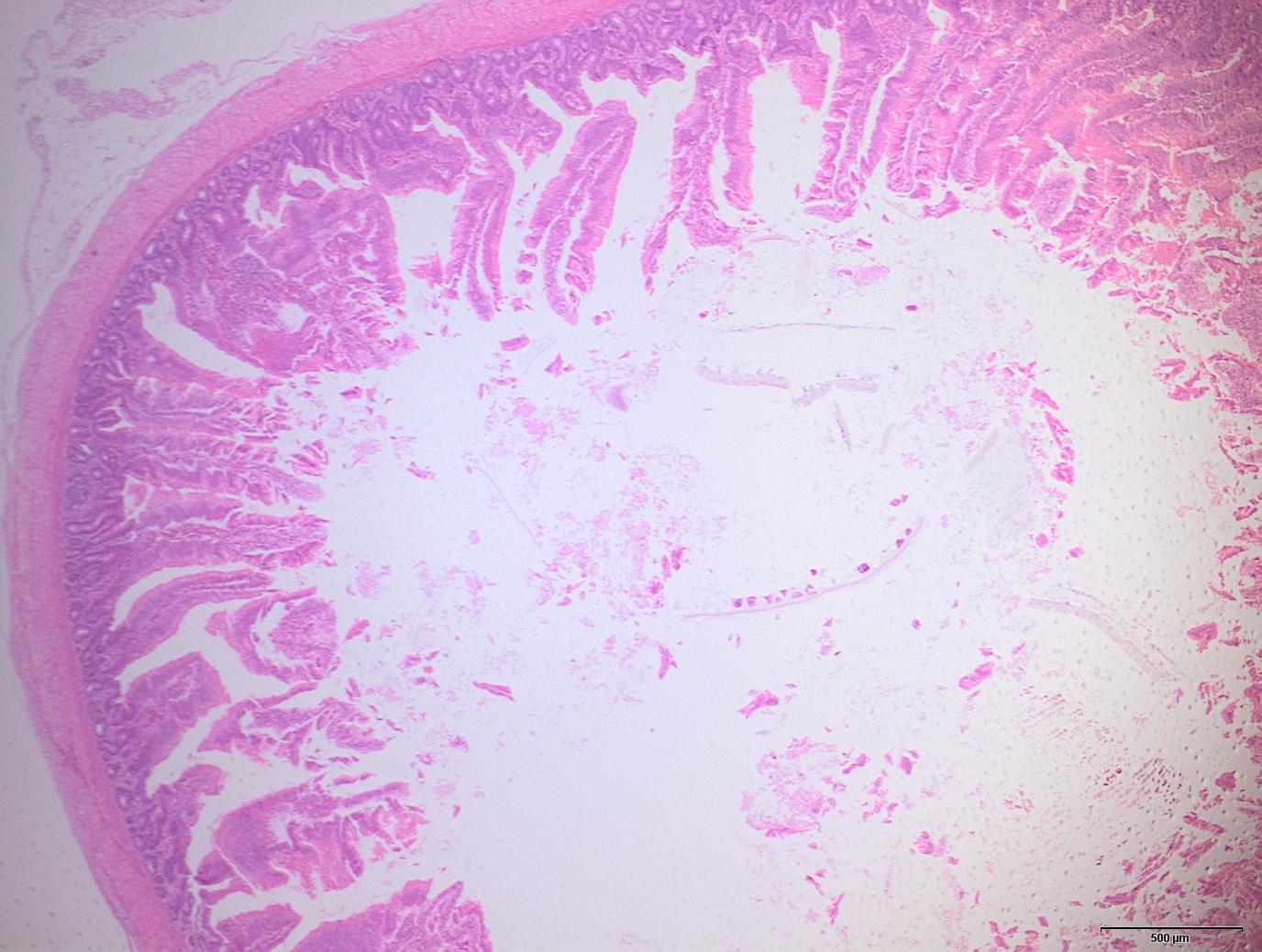

Supplement: Supplementary file 6 [file Data_Sheet_1.ZIP › Data sheet/Hematoxylin-eosin Staining/Jejunum/NE group/4.jpg]

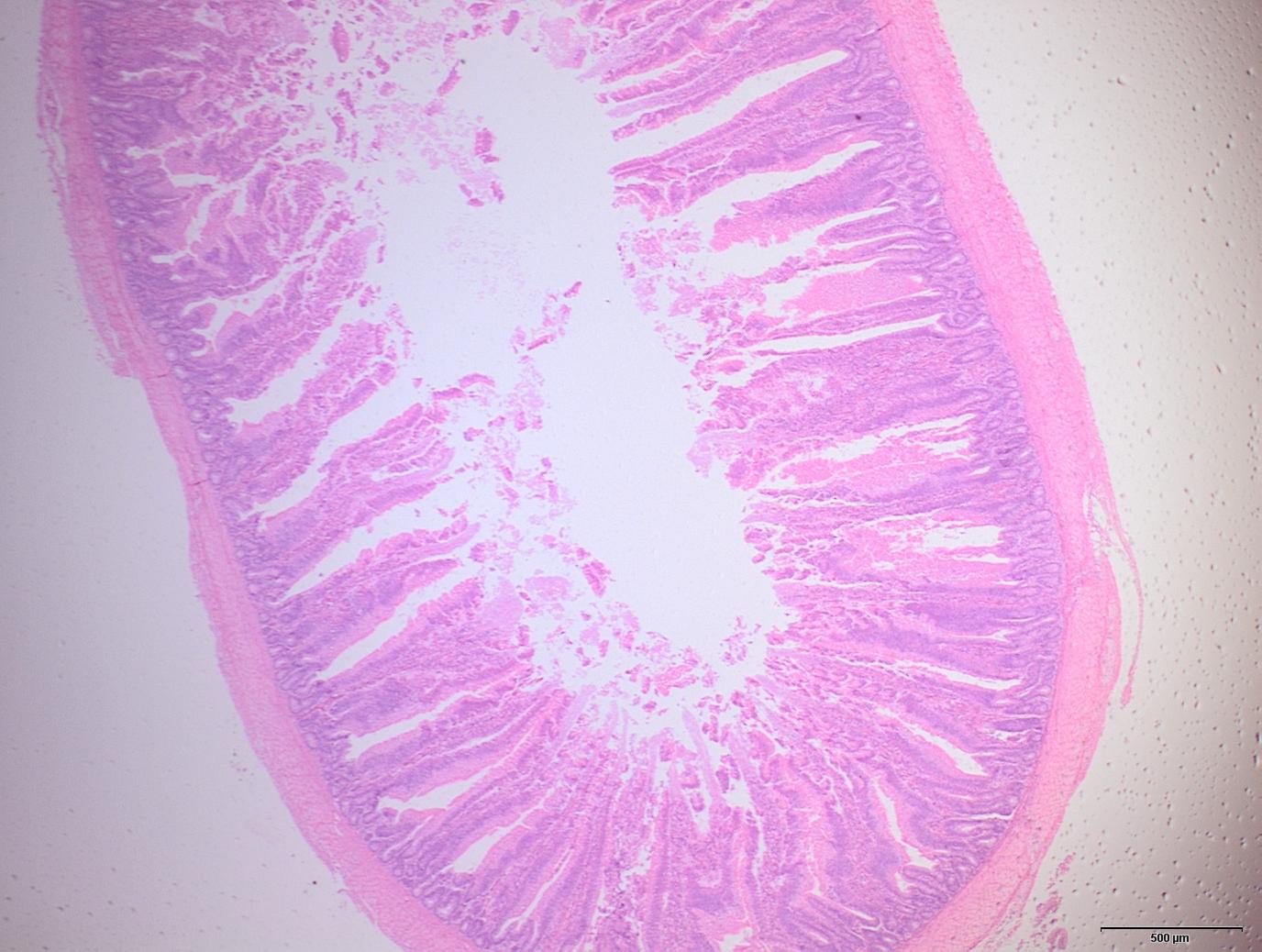

Supplement: Supplementary file 6 [file Data_Sheet_1.ZIP › Data sheet/Hematoxylin-eosin Staining/Jejunum/NE group/5.jpg]

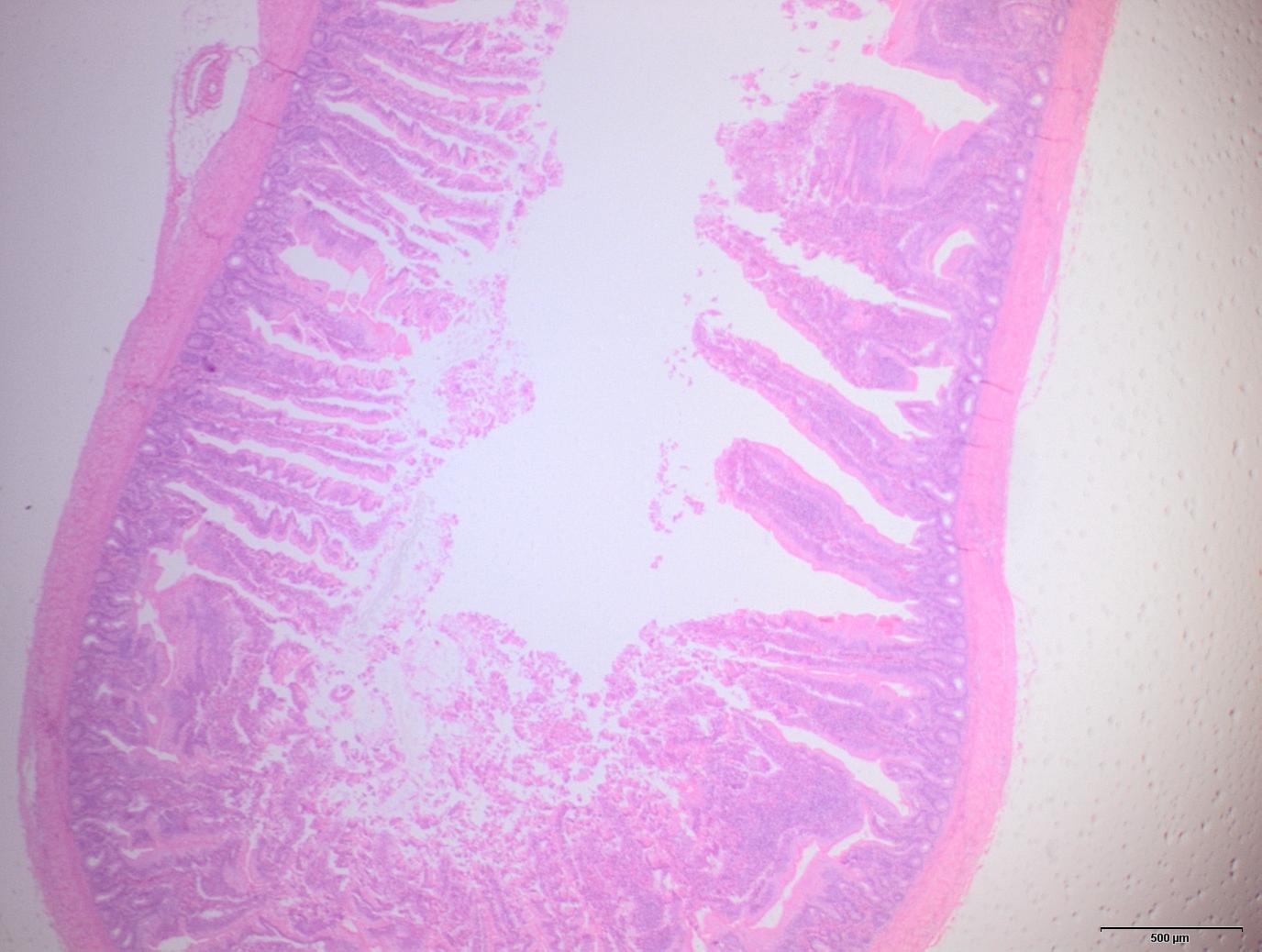

Supplement: Supplementary file 6 [file Data_Sheet_1.ZIP › Data sheet/Hematoxylin-eosin Staining/Jejunum/NE group/6.jpg]

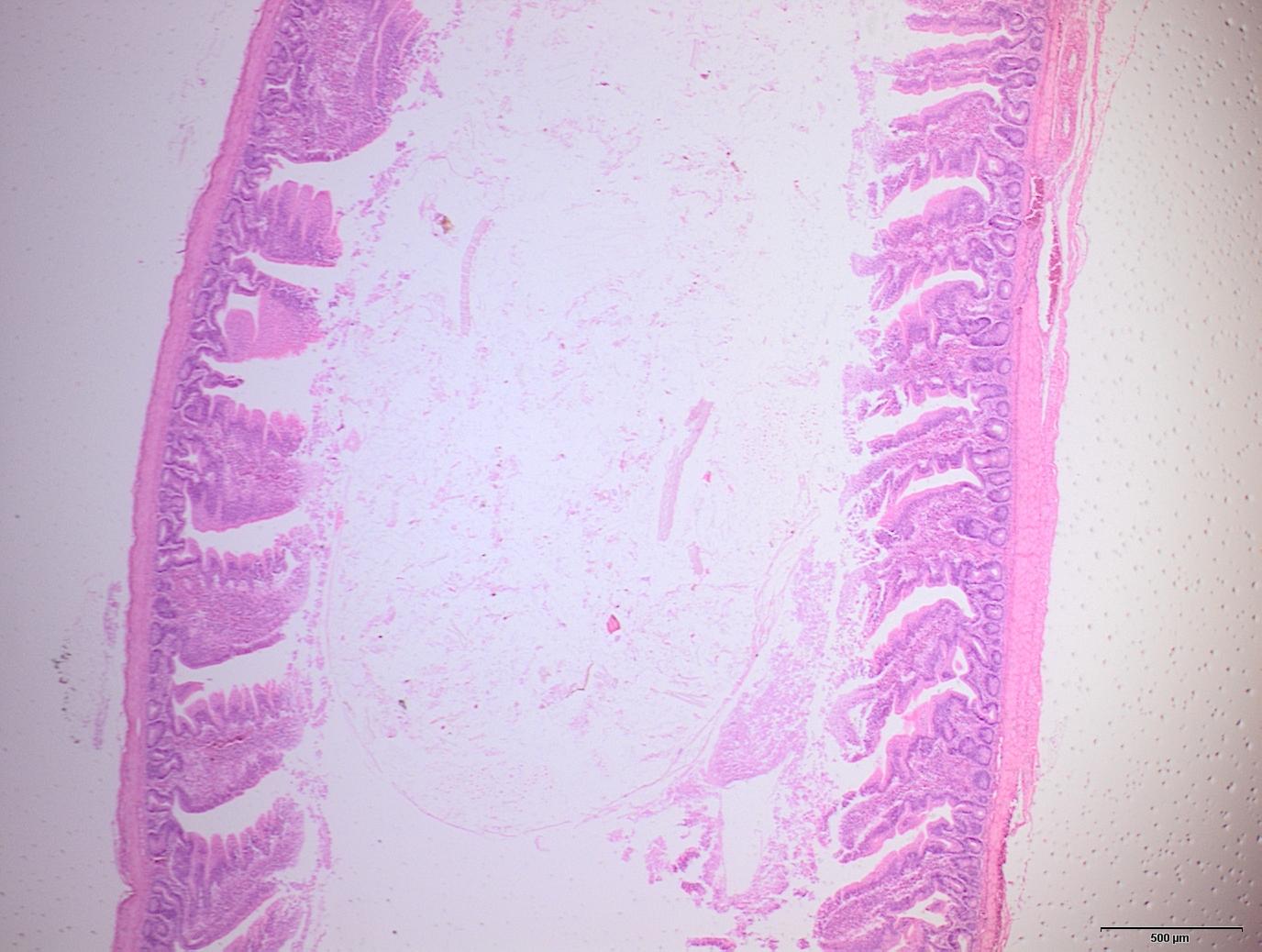

Supplement: Supplementary file 6 [file Data_Sheet_1.ZIP › Data sheet/Hematoxylin-eosin Staining/Jejunum/NE group/7.jpg]

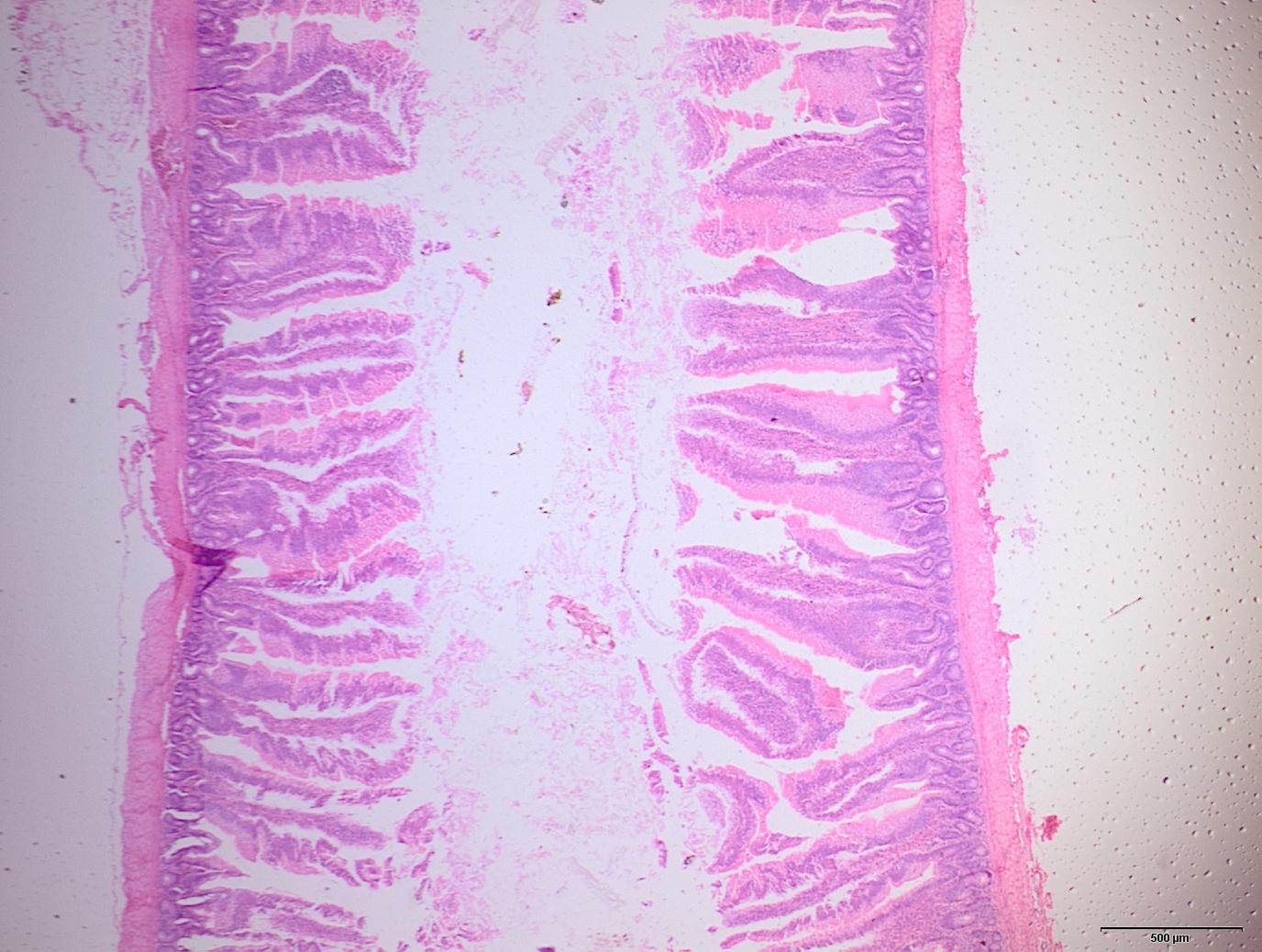

Supplement: Supplementary file 6 [file Data_Sheet_1.ZIP › Data sheet/Hematoxylin-eosin Staining/Jejunum/NE group/8.jpg]

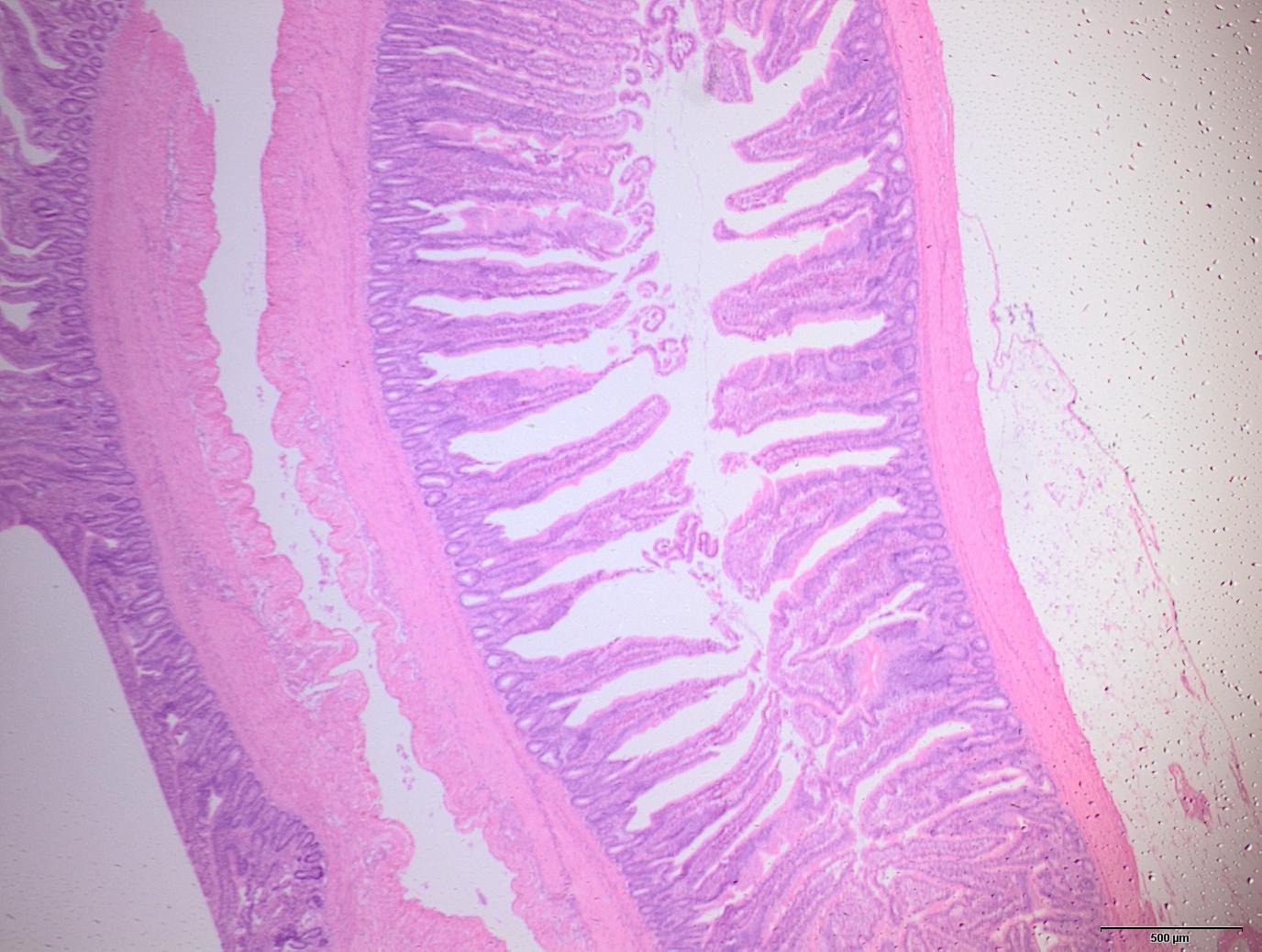

Supplement: Supplementary file 6 [file Data_Sheet_1.ZIP › Data sheet/Hematoxylin-eosin Staining/Jejunum/NE+TA400 group/1.jpg]

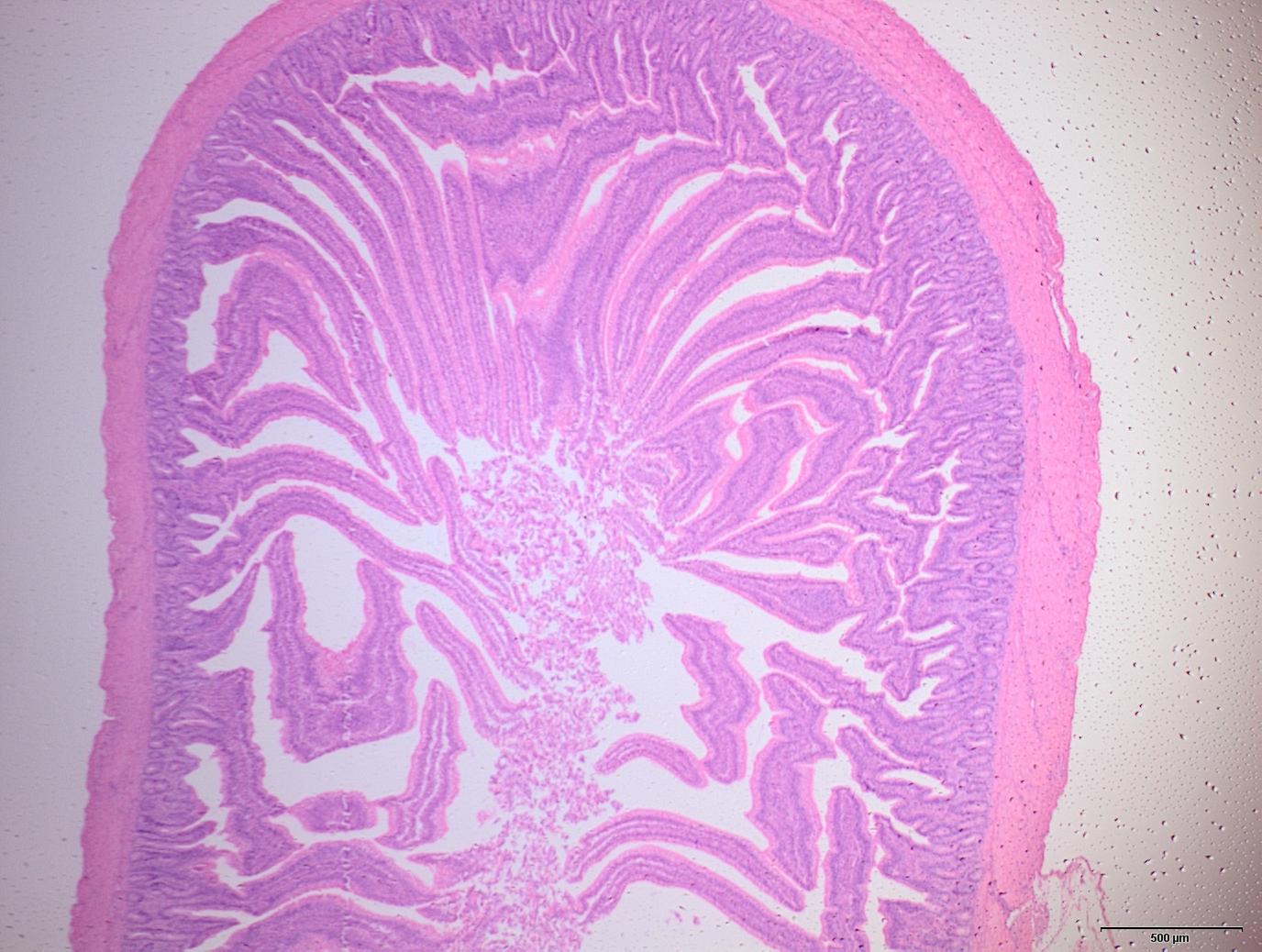

Supplement: Supplementary file 6 [file Data_Sheet_1.ZIP › Data sheet/Hematoxylin-eosin Staining/Jejunum/NE+TA400 group/2.jpg]

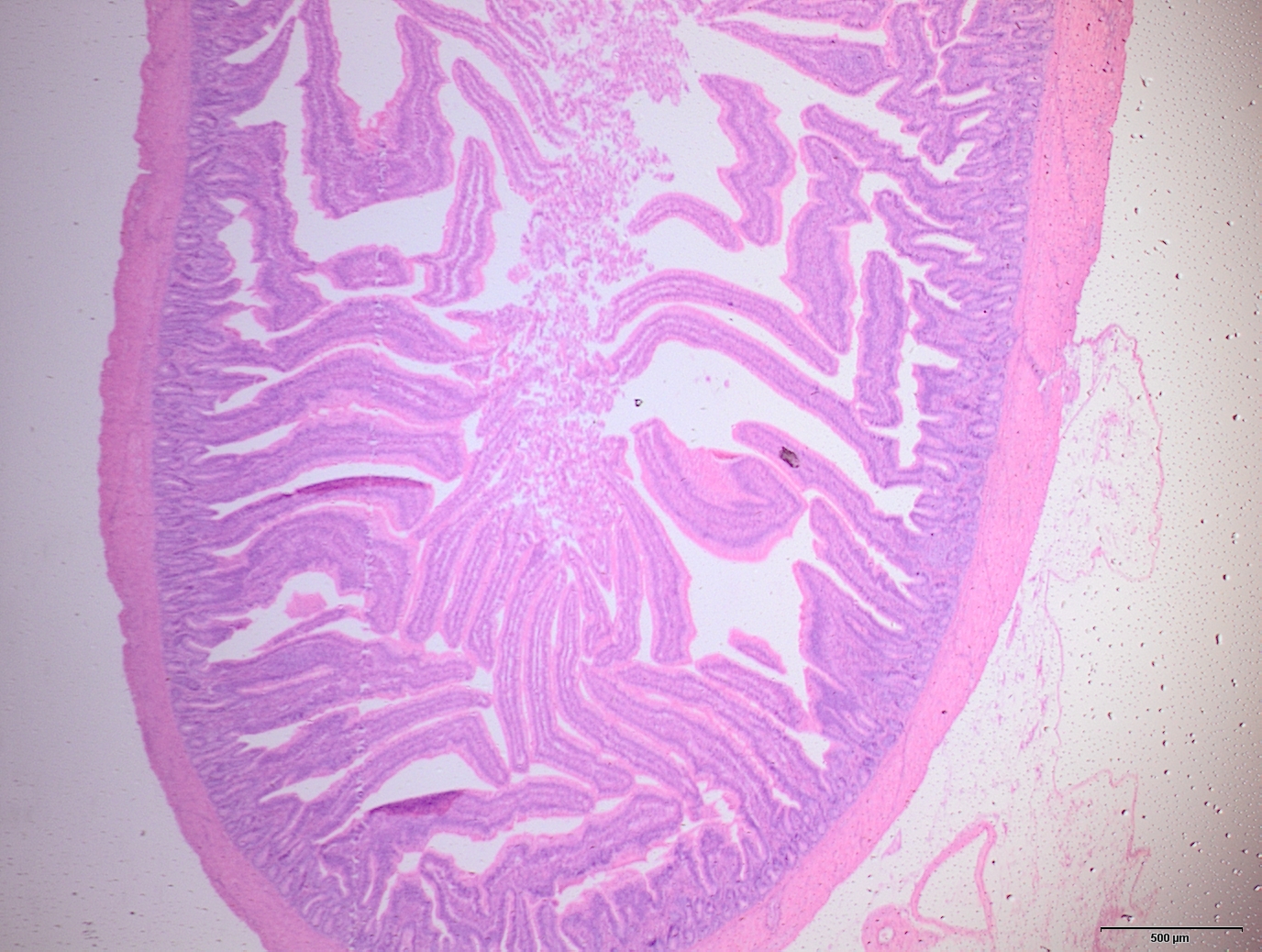

Supplement: Supplementary file 6 [file Data_Sheet_1.ZIP › Data sheet/Hematoxylin-eosin Staining/Jejunum/NE+TA400 group/3.jpg]

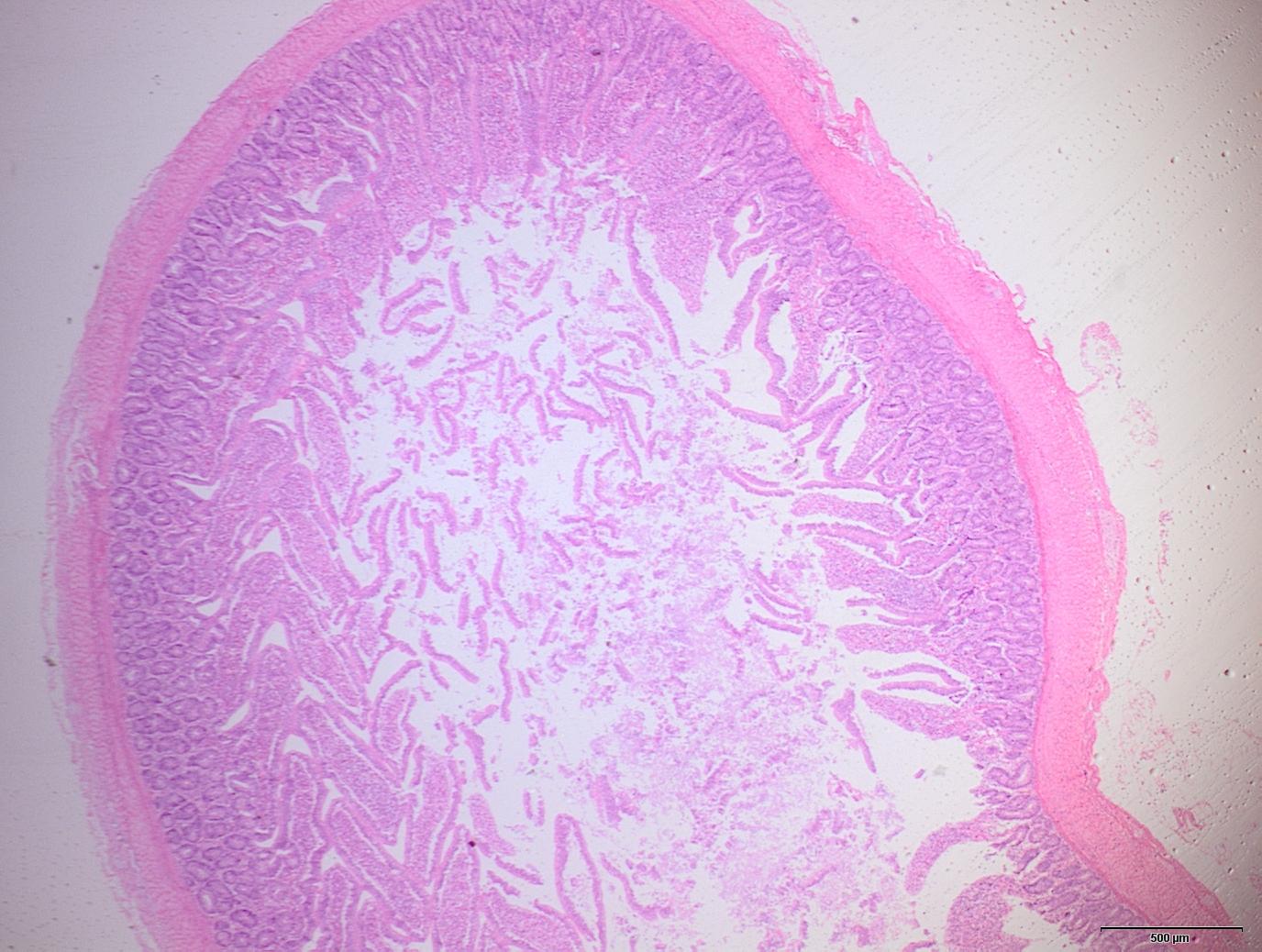

Supplement: Supplementary file 6 [file Data_Sheet_1.ZIP › Data sheet/Hematoxylin-eosin Staining/Jejunum/NE+TA400 group/4.jpg]

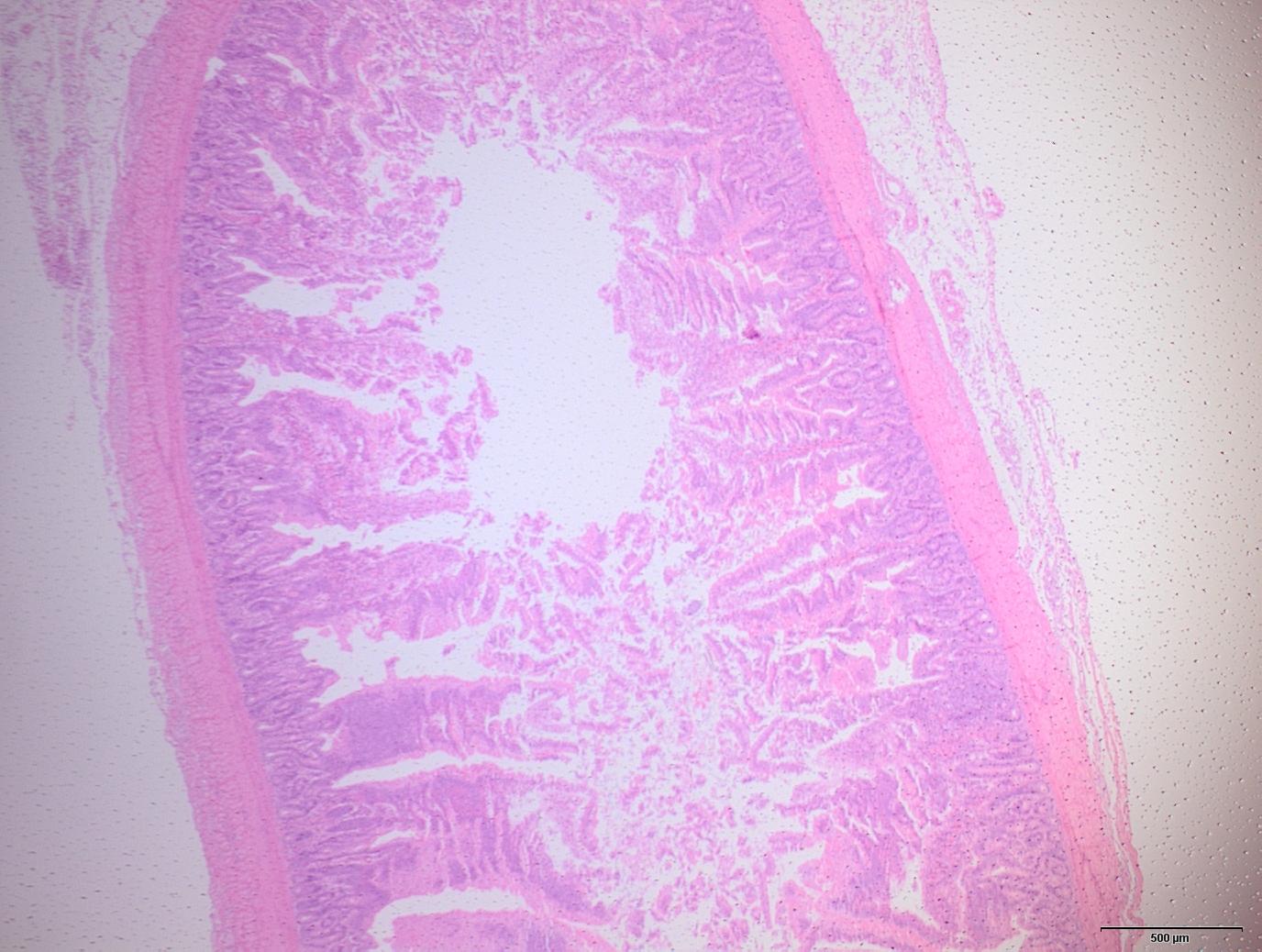

Supplement: Supplementary file 6 [file Data_Sheet_1.ZIP › Data sheet/Hematoxylin-eosin Staining/Jejunum/NE+TA400 group/5.jpg]

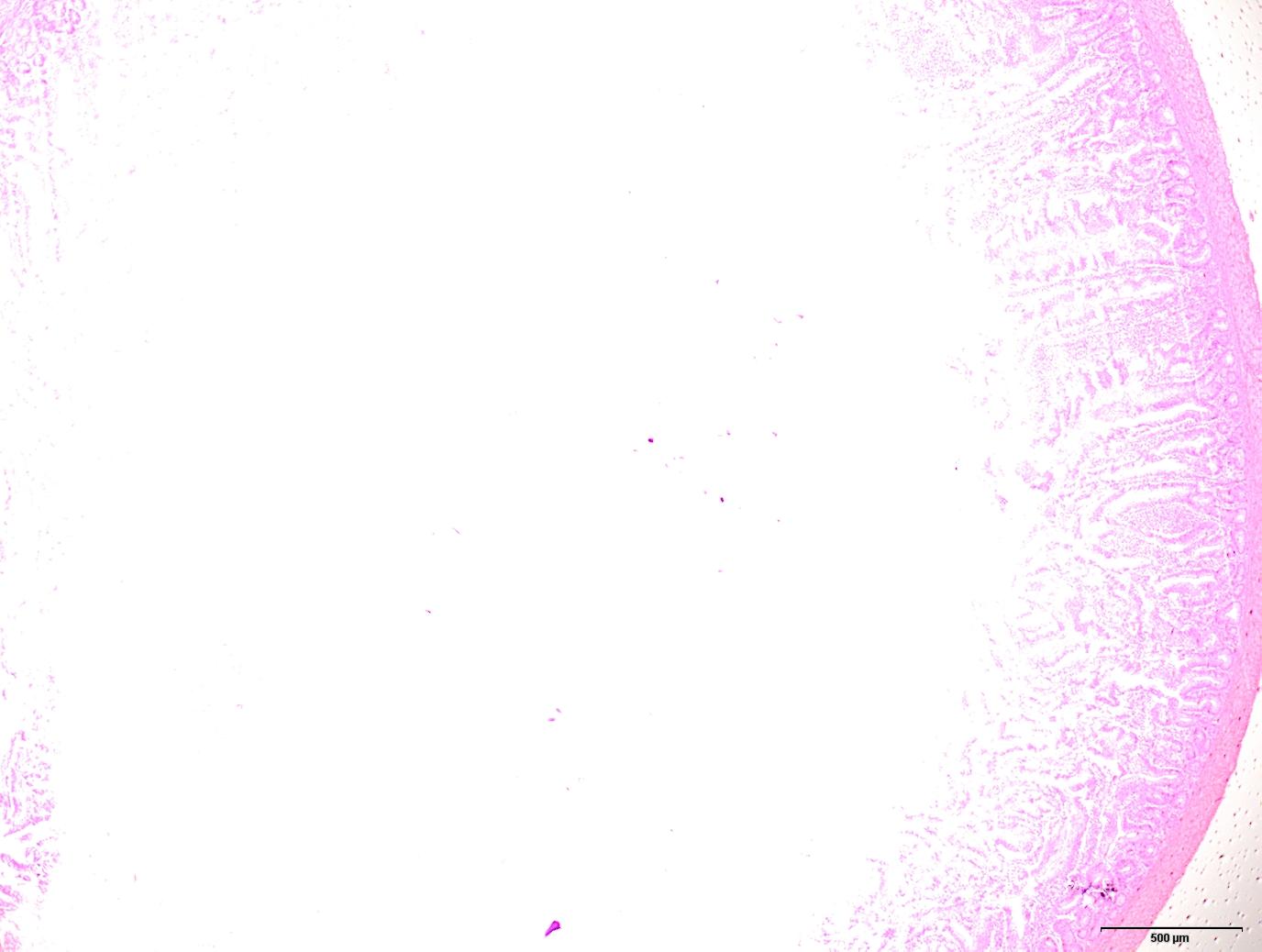

Supplement: Supplementary file 6 [file Data_Sheet_1.ZIP › Data sheet/Hematoxylin-eosin Staining/Jejunum/NE+TA400 group/6.jpg]

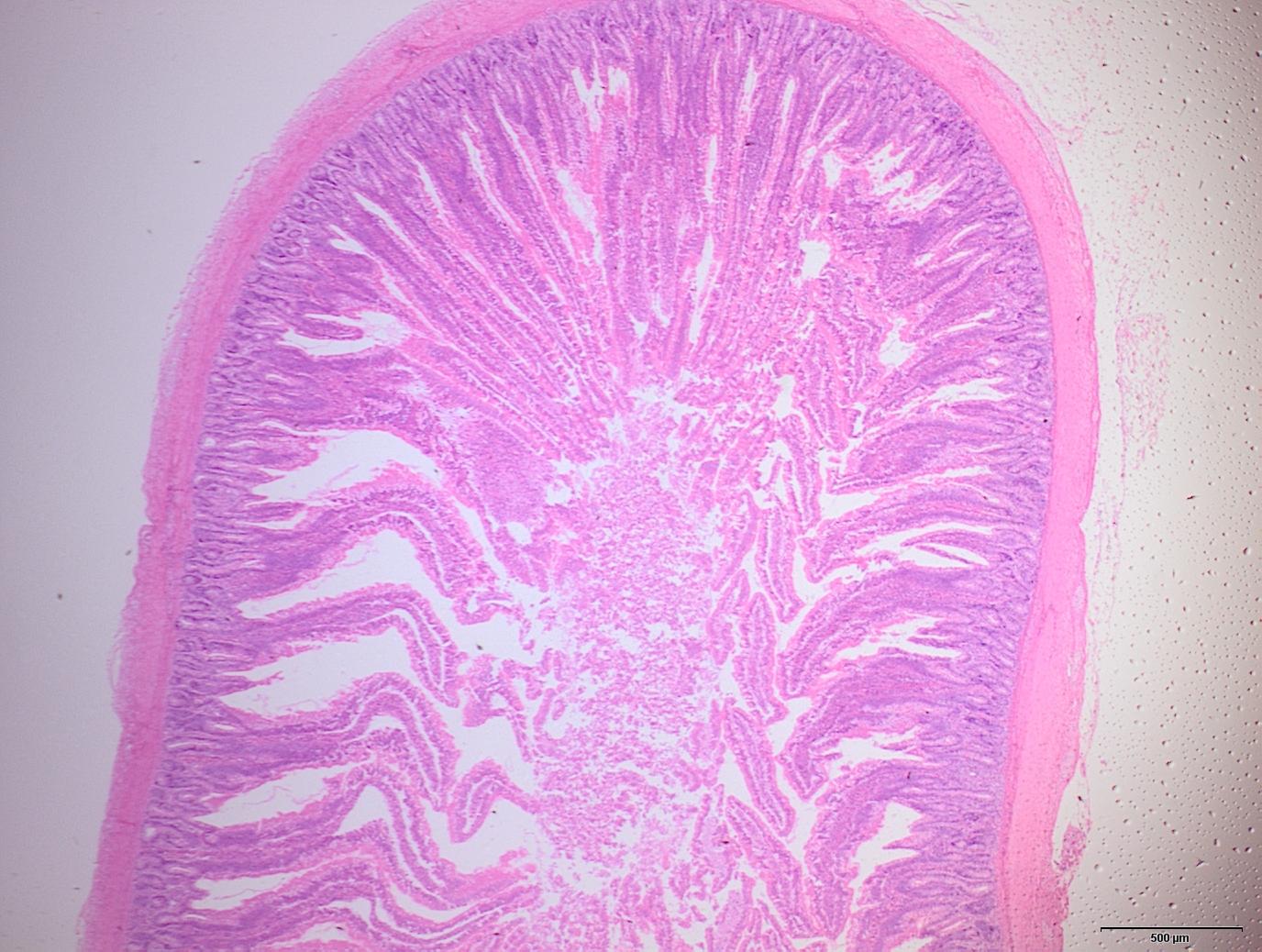

Supplement: Supplementary file 6 [file Data_Sheet_1.ZIP › Data sheet/Hematoxylin-eosin Staining/Jejunum/NE+TA400 group/7.jpg]

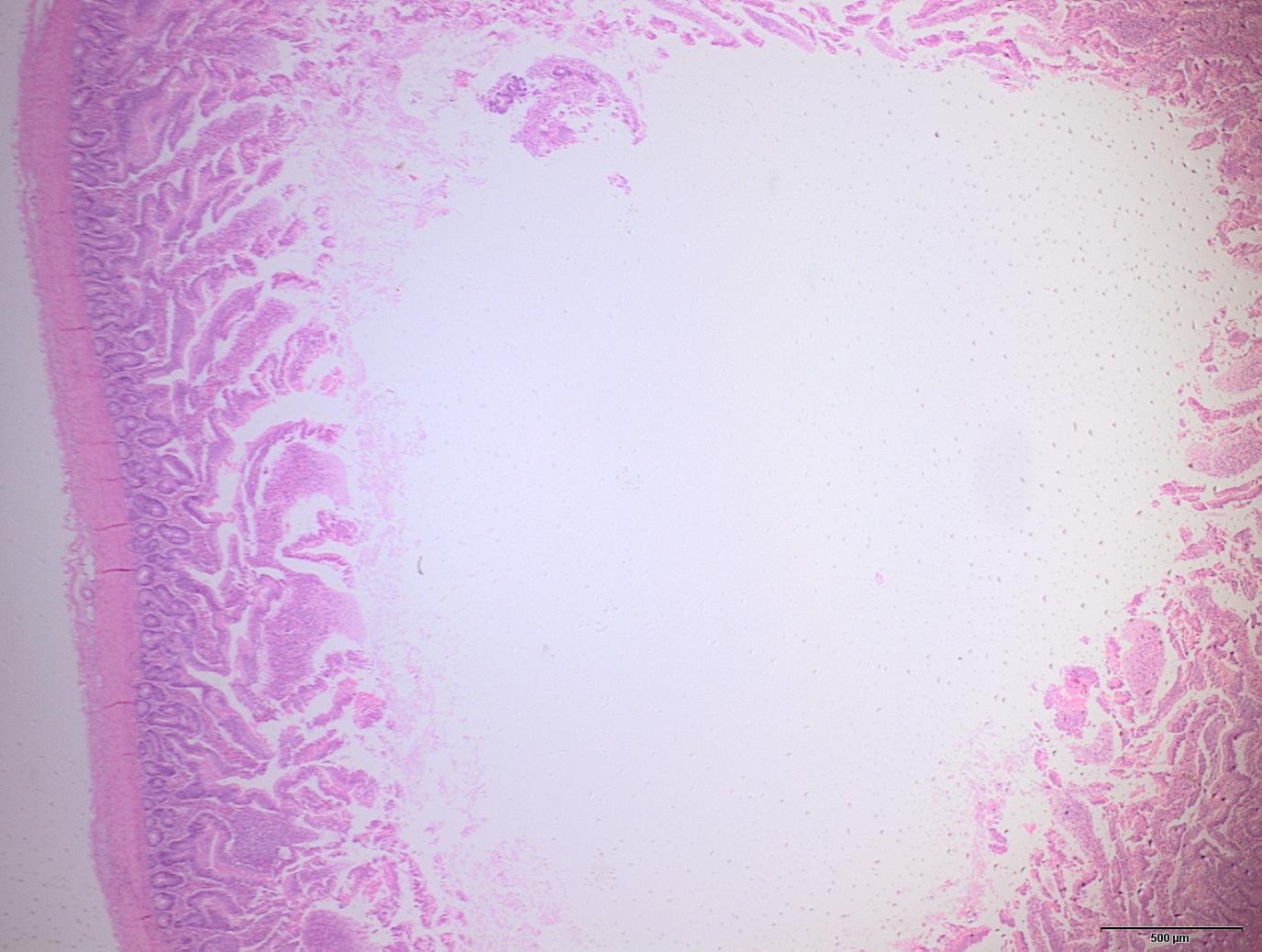

Supplement: Supplementary file 6 [file Data_Sheet_1.ZIP › Data sheet/Hematoxylin-eosin Staining/Jejunum/NE+TA400 group/8.jpg]

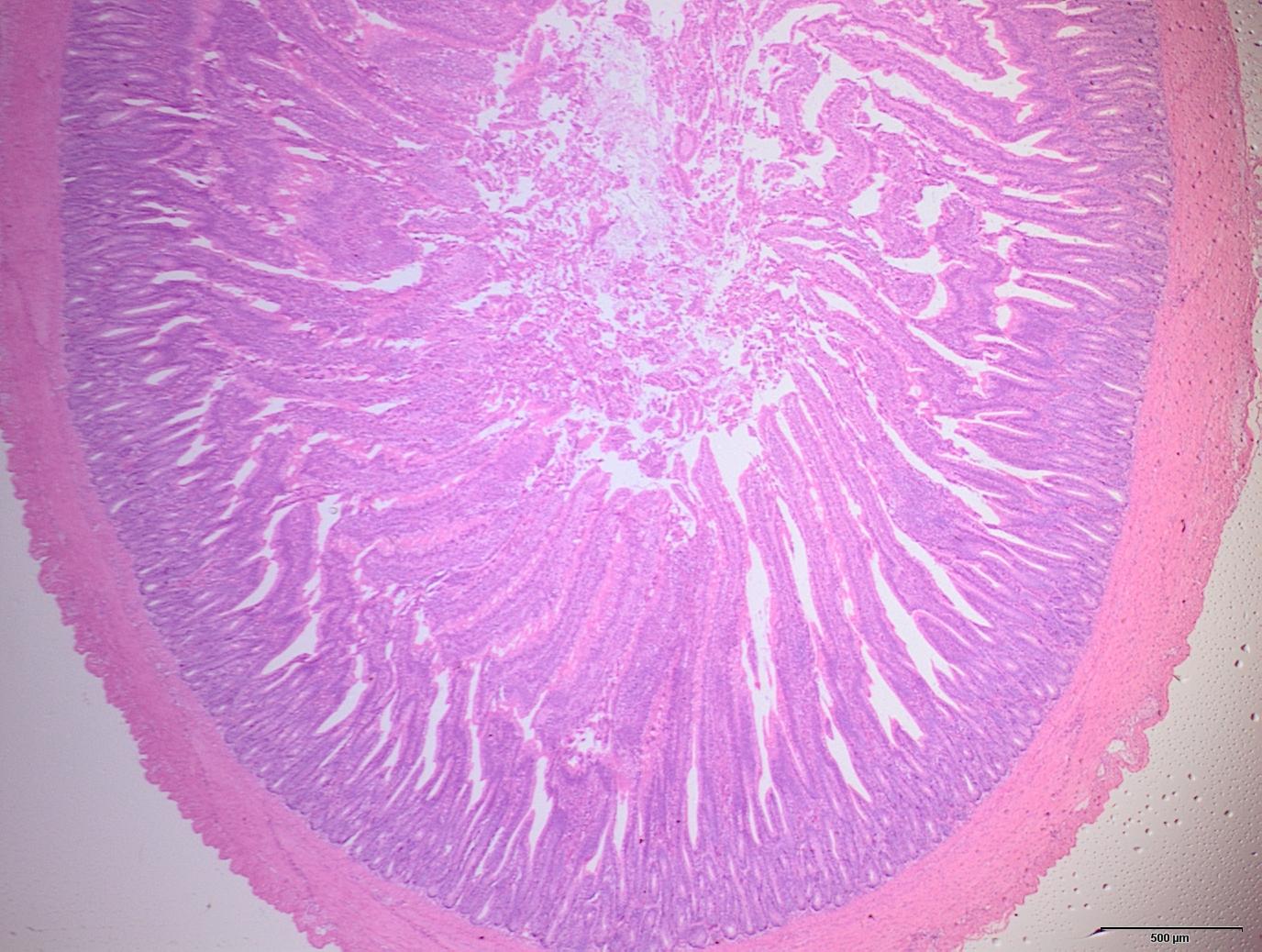

Supplement: Supplementary file 6 [file Data_Sheet_1.ZIP › Data sheet/Hematoxylin-eosin Staining/Jejunum/NE+TA600 group/1.jpg]

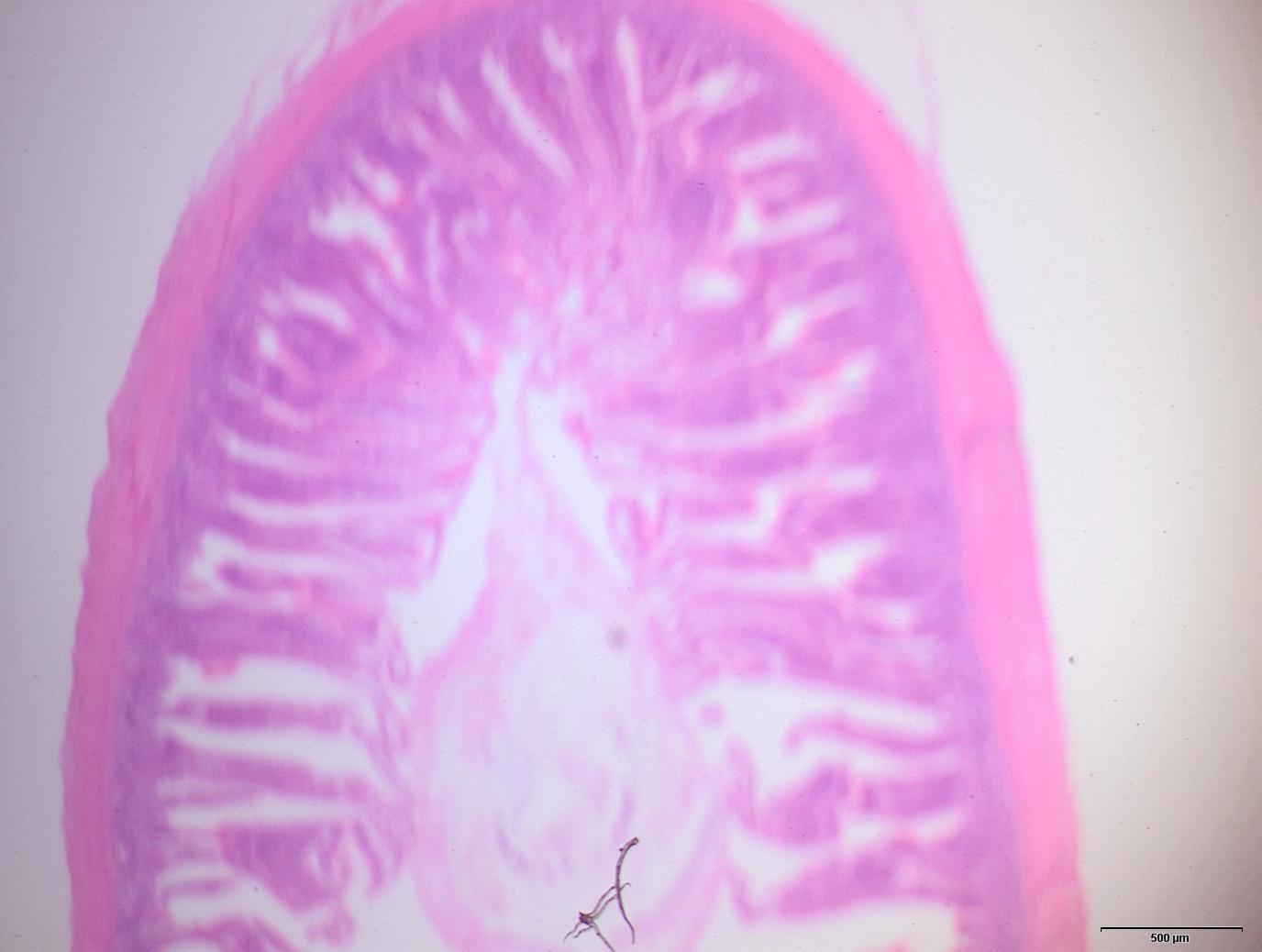

Supplement: Supplementary file 6 [file Data_Sheet_1.ZIP › Data sheet/Hematoxylin-eosin Staining/Jejunum/NE+TA600 group/2.jpg]

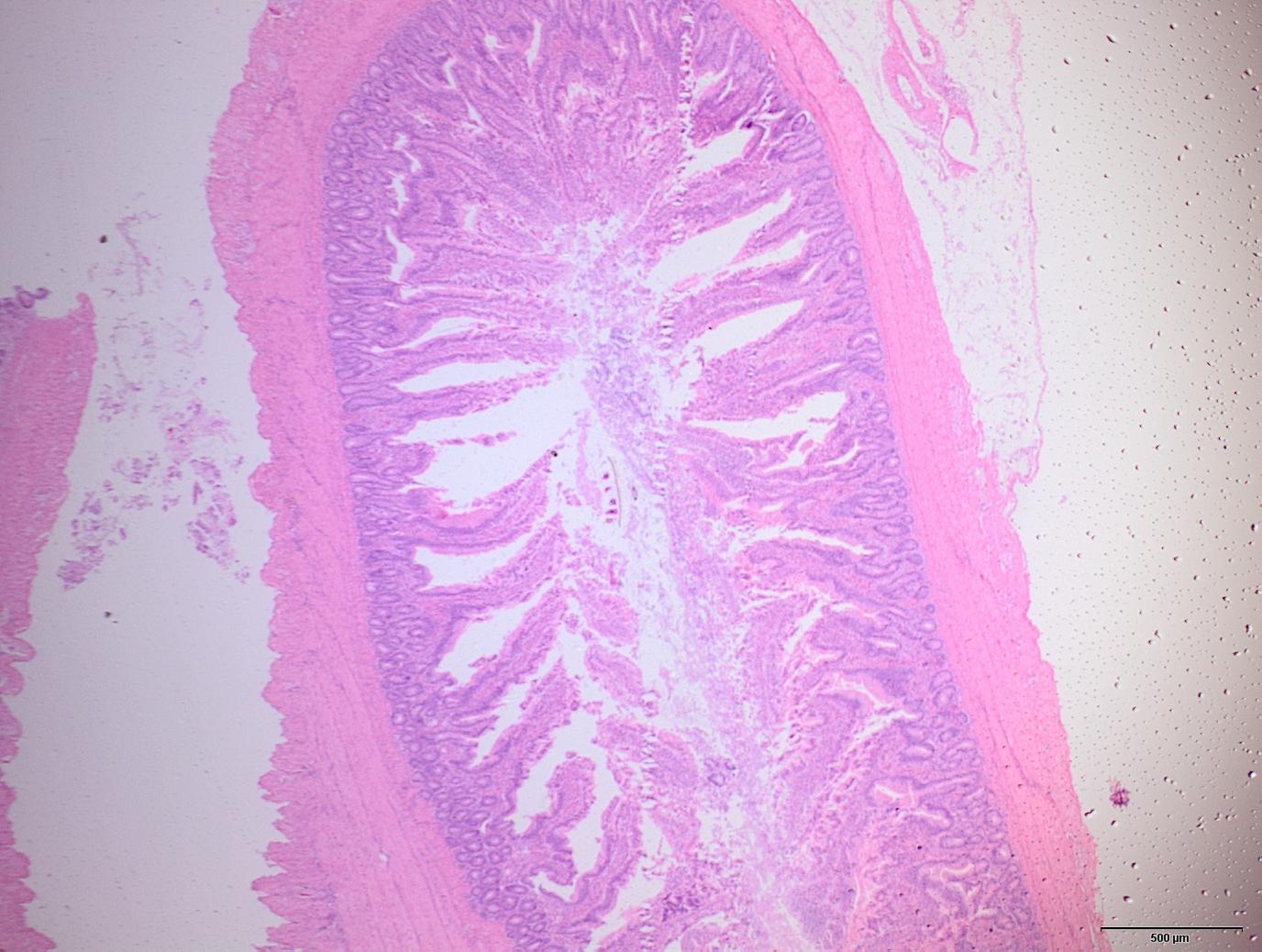

Supplement: Supplementary file 6 [file Data_Sheet_1.ZIP › Data sheet/Hematoxylin-eosin Staining/Jejunum/NE+TA600 group/3.jpg]

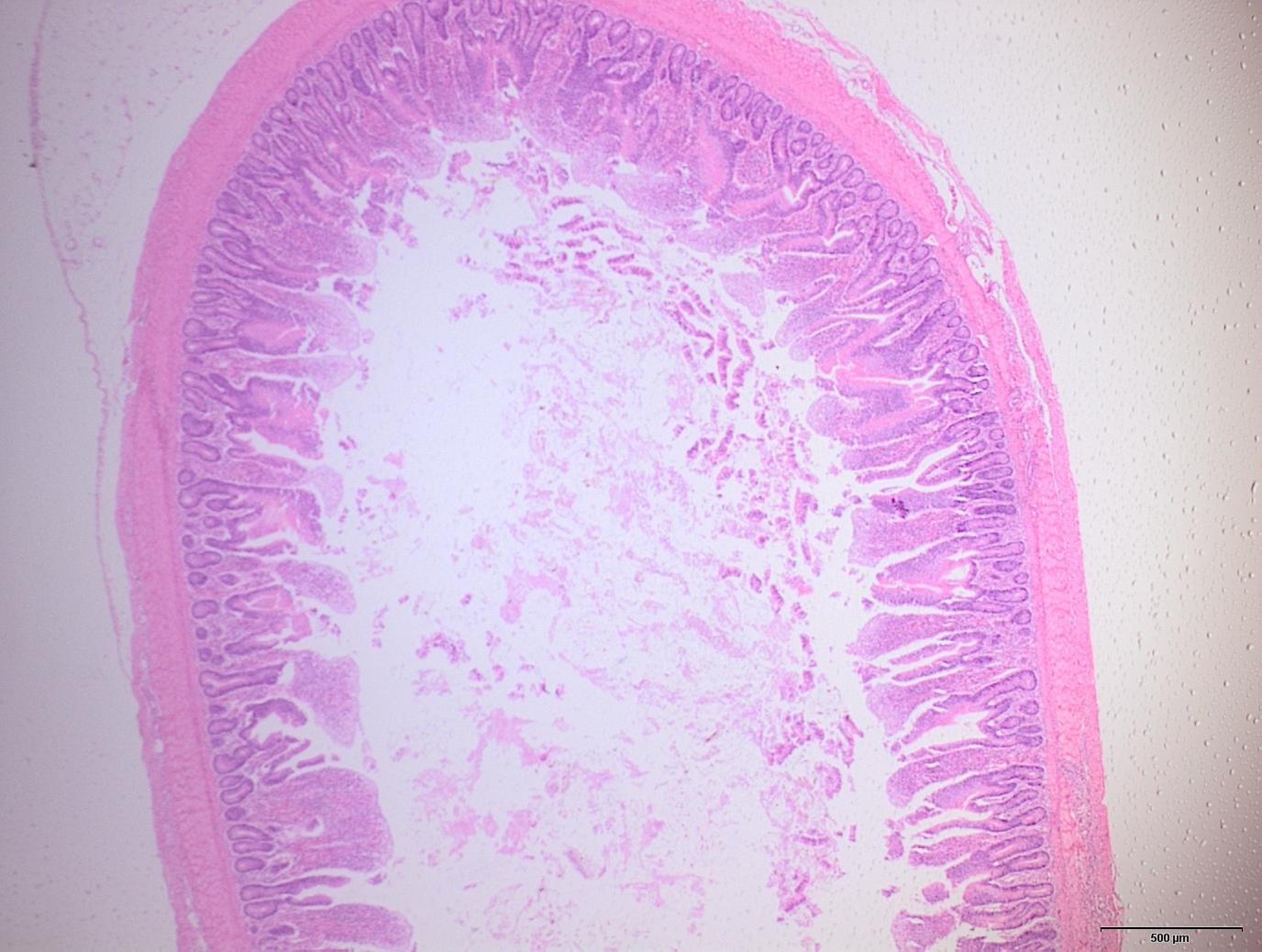

Supplement: Supplementary file 6 [file Data_Sheet_1.ZIP › Data sheet/Hematoxylin-eosin Staining/Jejunum/NE+TA600 group/4.jpg]

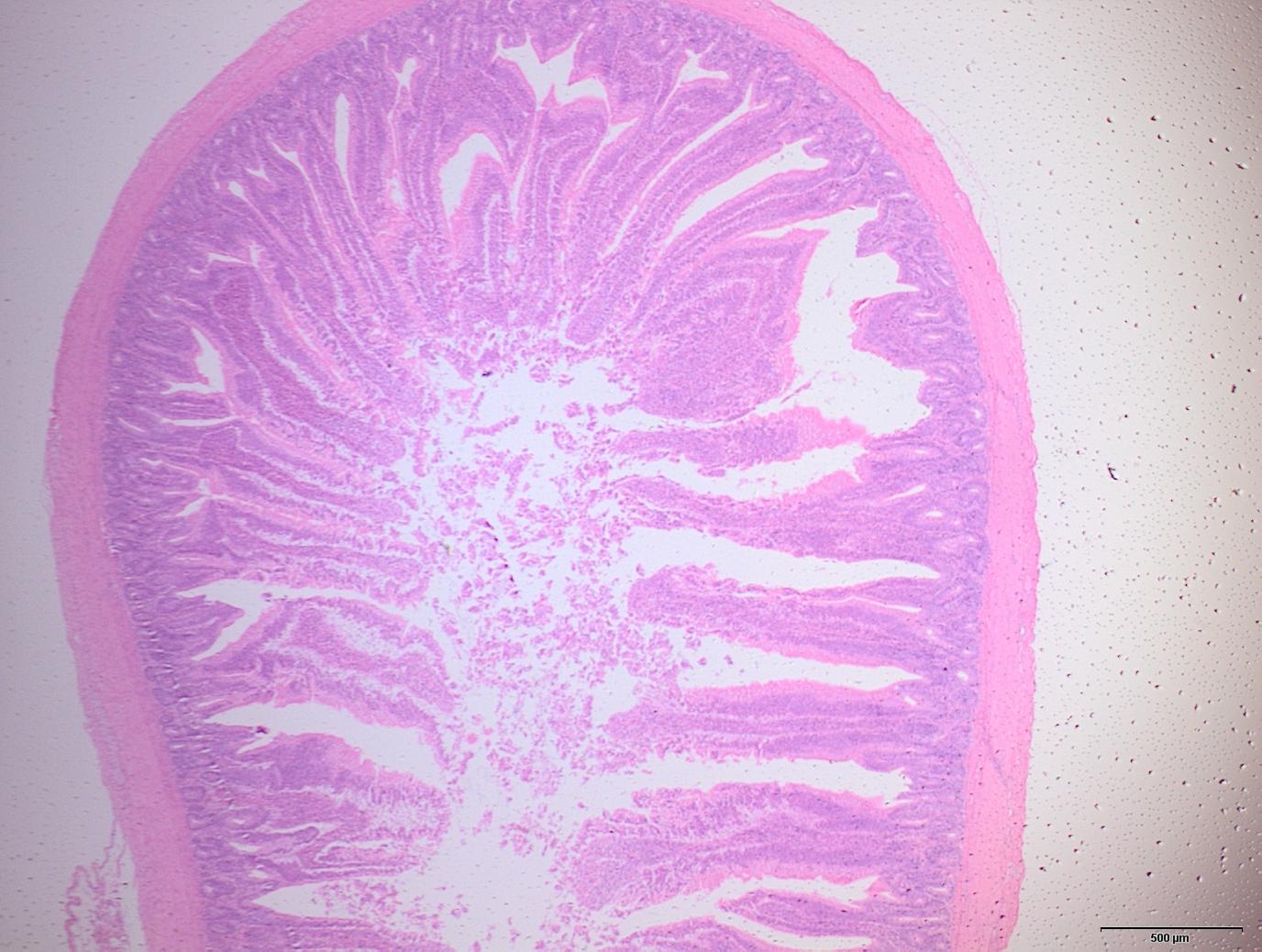

Supplement: Supplementary file 6 [file Data_Sheet_1.ZIP › Data sheet/Hematoxylin-eosin Staining/Jejunum/NE+TA600 group/5.jpg]

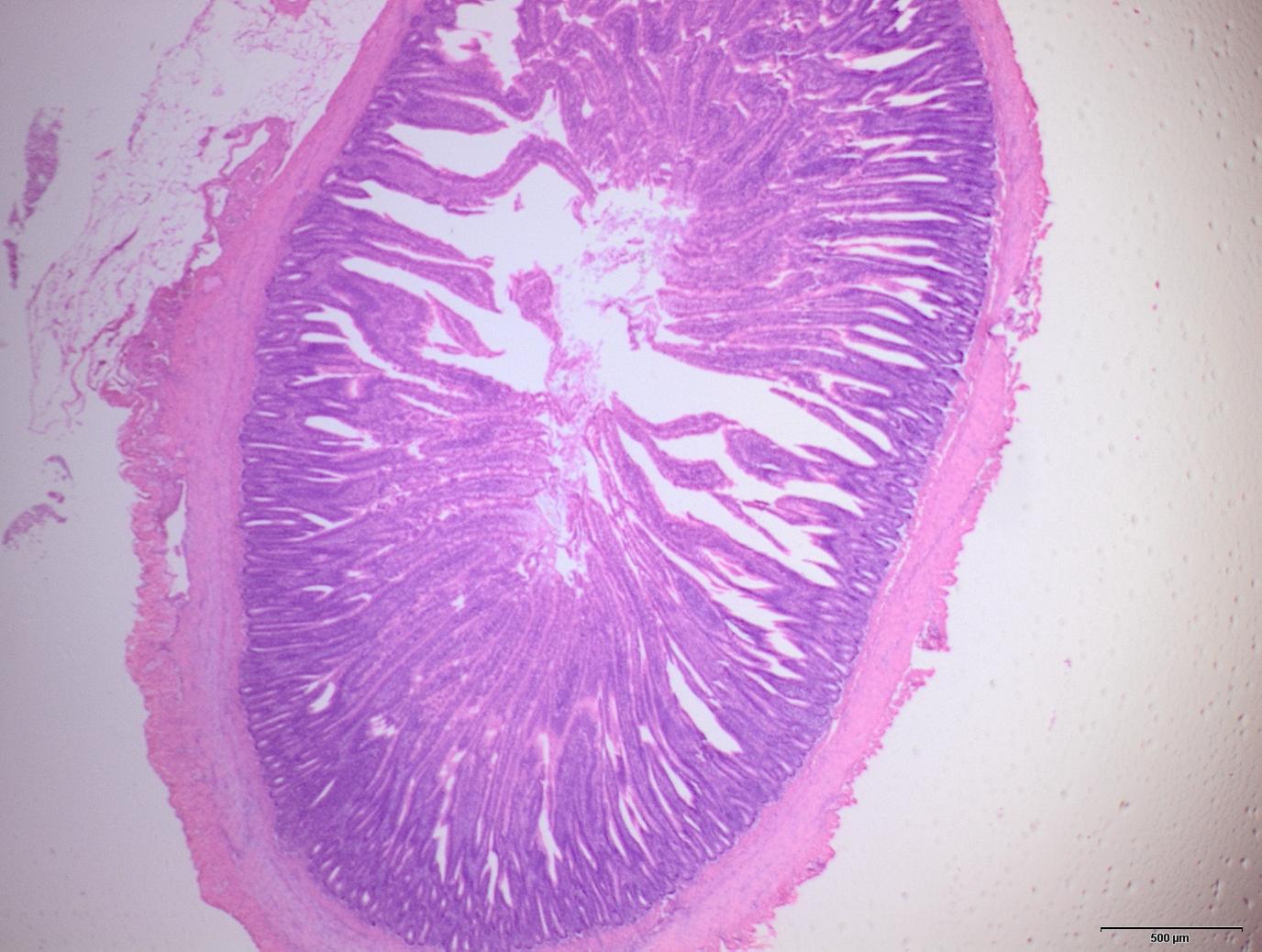

Supplement: Supplementary file 6 [file Data_Sheet_1.ZIP › Data sheet/Hematoxylin-eosin Staining/Jejunum/NE+TA600 group/6.jpg]

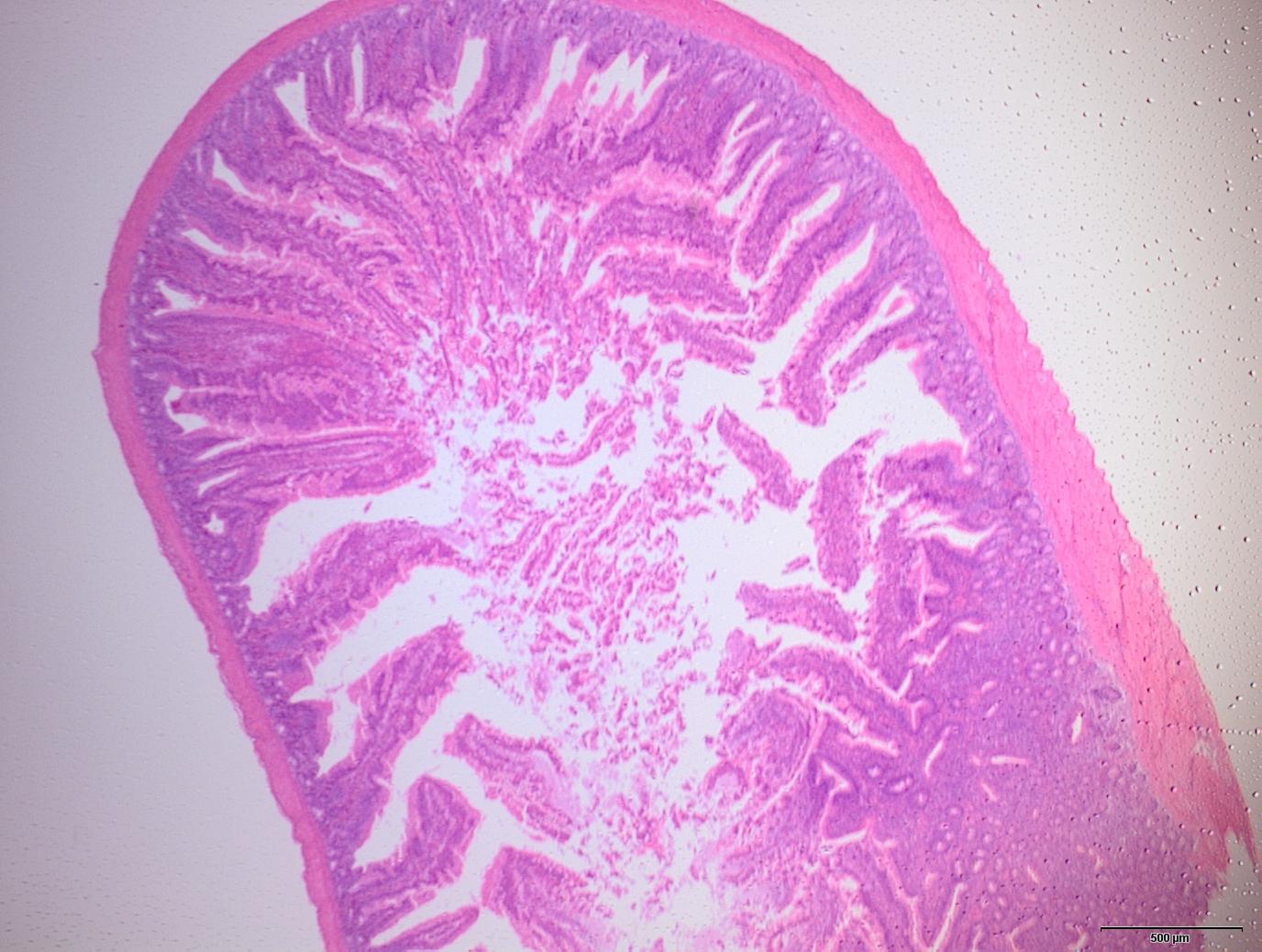

Supplement: Supplementary file 6 [file Data_Sheet_1.ZIP › Data sheet/Hematoxylin-eosin Staining/Jejunum/NE+TA600 group/7.jpg]

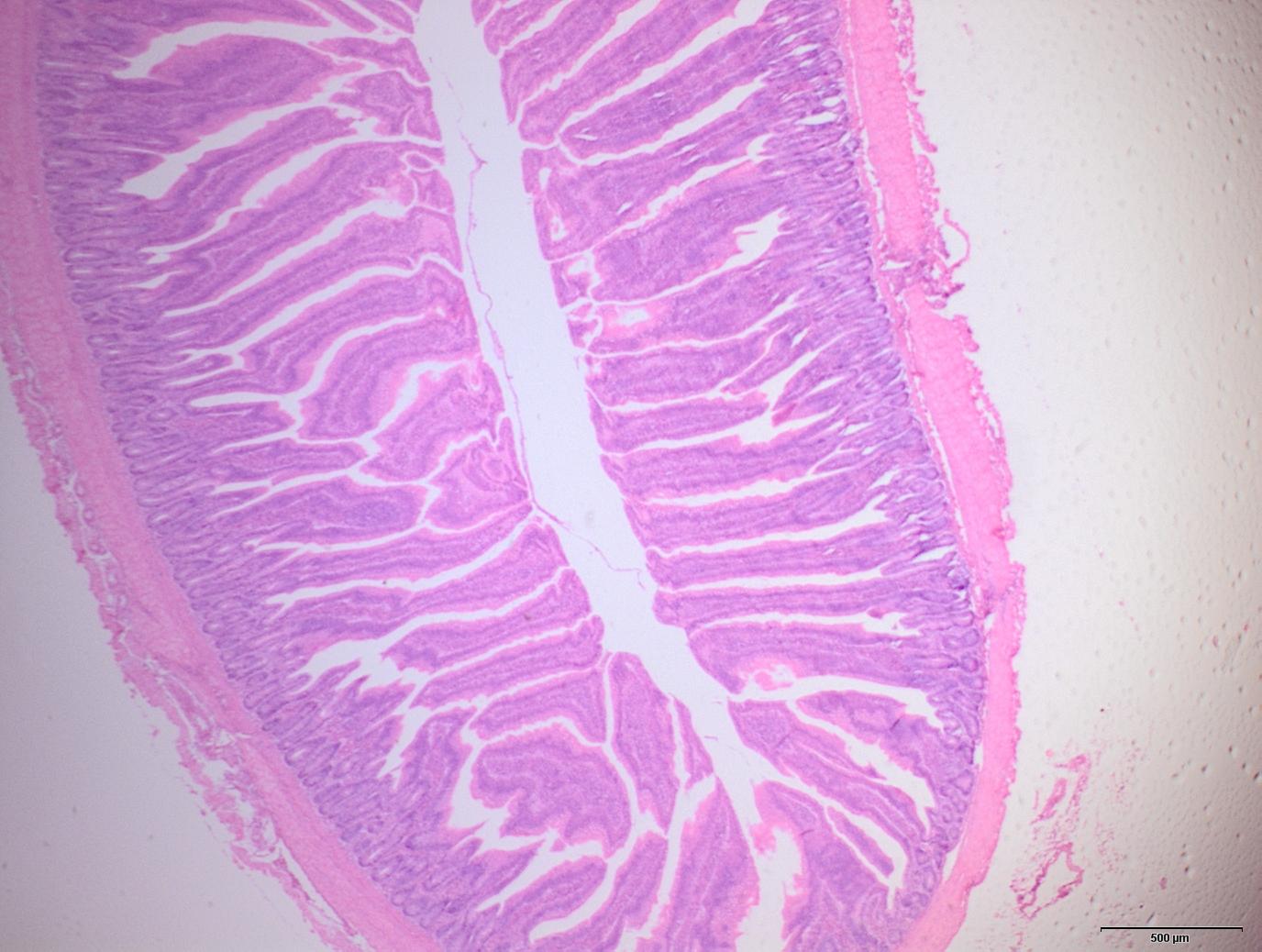

Supplement: Supplementary file 6 [file Data_Sheet_1.ZIP › Data sheet/Hematoxylin-eosin Staining/Jejunum/NE+TA600 group/8.jpg]

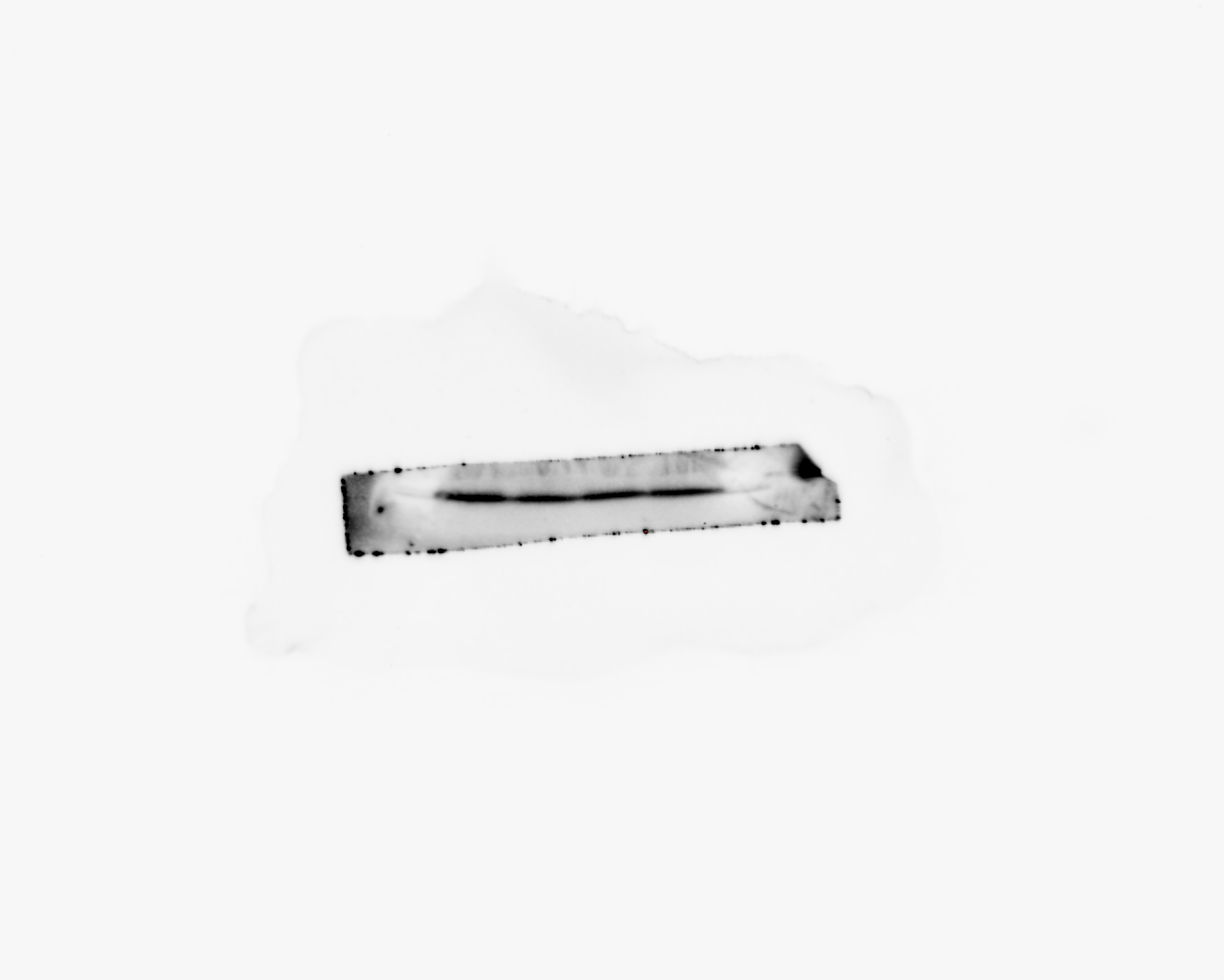

Supplement: Supplementary file 6 [file Data_Sheet_1.ZIP › Data sheet/Western Blot/CLDN-1/Ileum/CLDN1-1.jpg]

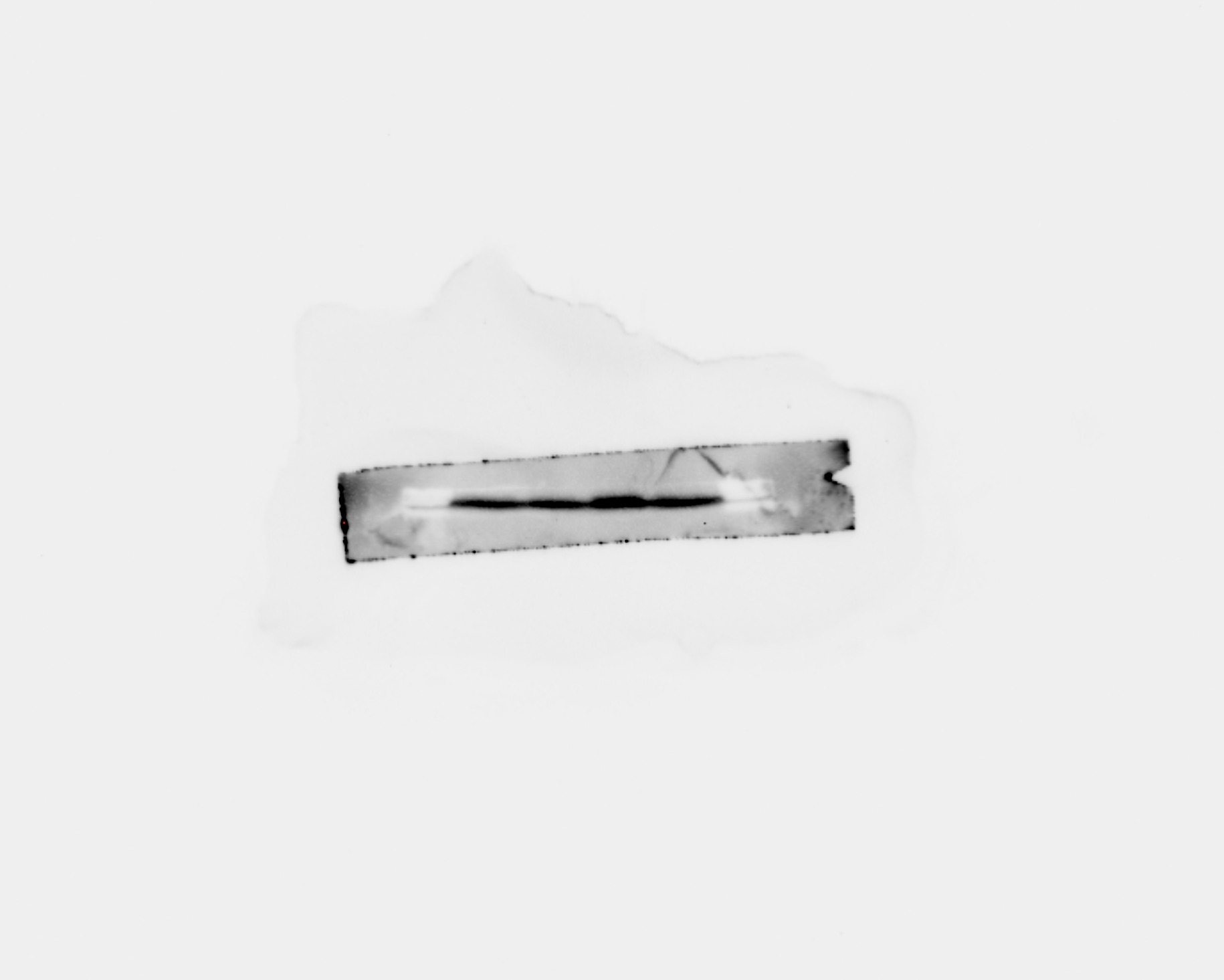

Supplement: Supplementary file 6 [file Data_Sheet_1.ZIP › Data sheet/Western Blot/CLDN-1/Ileum/CLDN1-2.jpg]

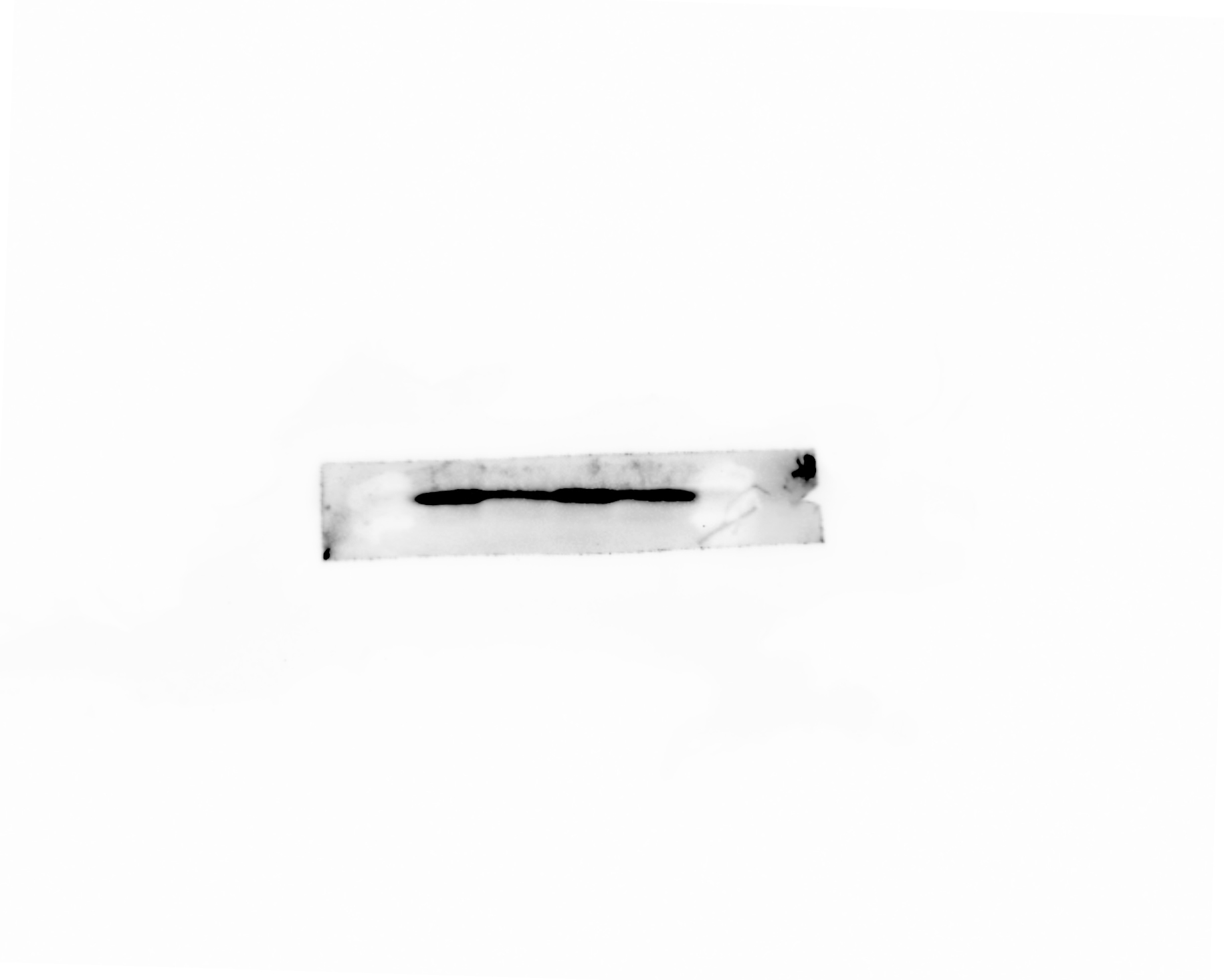

Supplement: Supplementary file 6 [file Data_Sheet_1.ZIP › Data sheet/Western Blot/CLDN-1/Ileum/CLDN1-3.jpg]

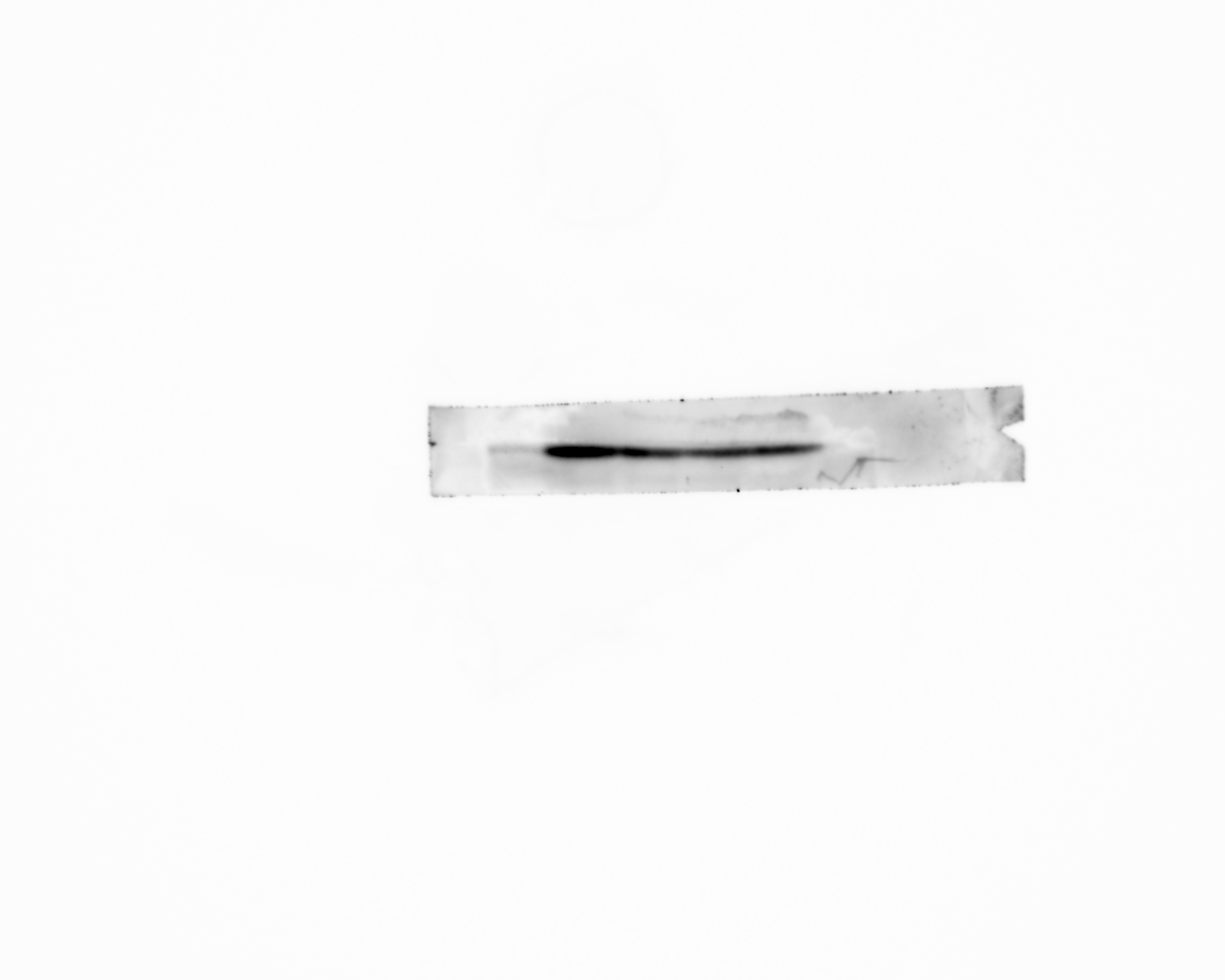

Supplement: Supplementary file 6 [file Data_Sheet_1.ZIP › Data sheet/Western Blot/CLDN-1/Jejunum/CLDN1-1.jpg]

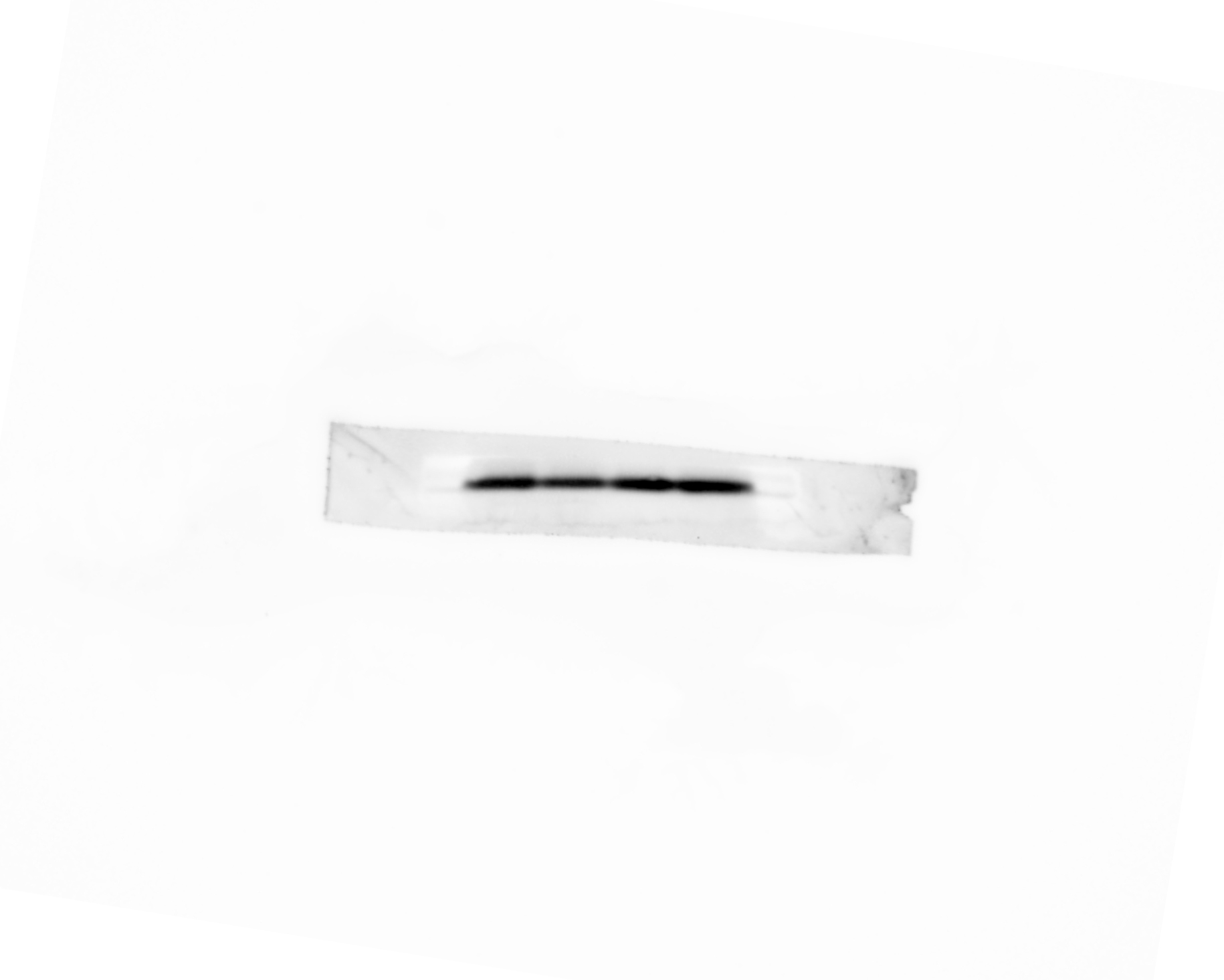

Supplement: Supplementary file 6 [file Data_Sheet_1.ZIP › Data sheet/Western Blot/CLDN-1/Jejunum/CLDN1-2.jpg]

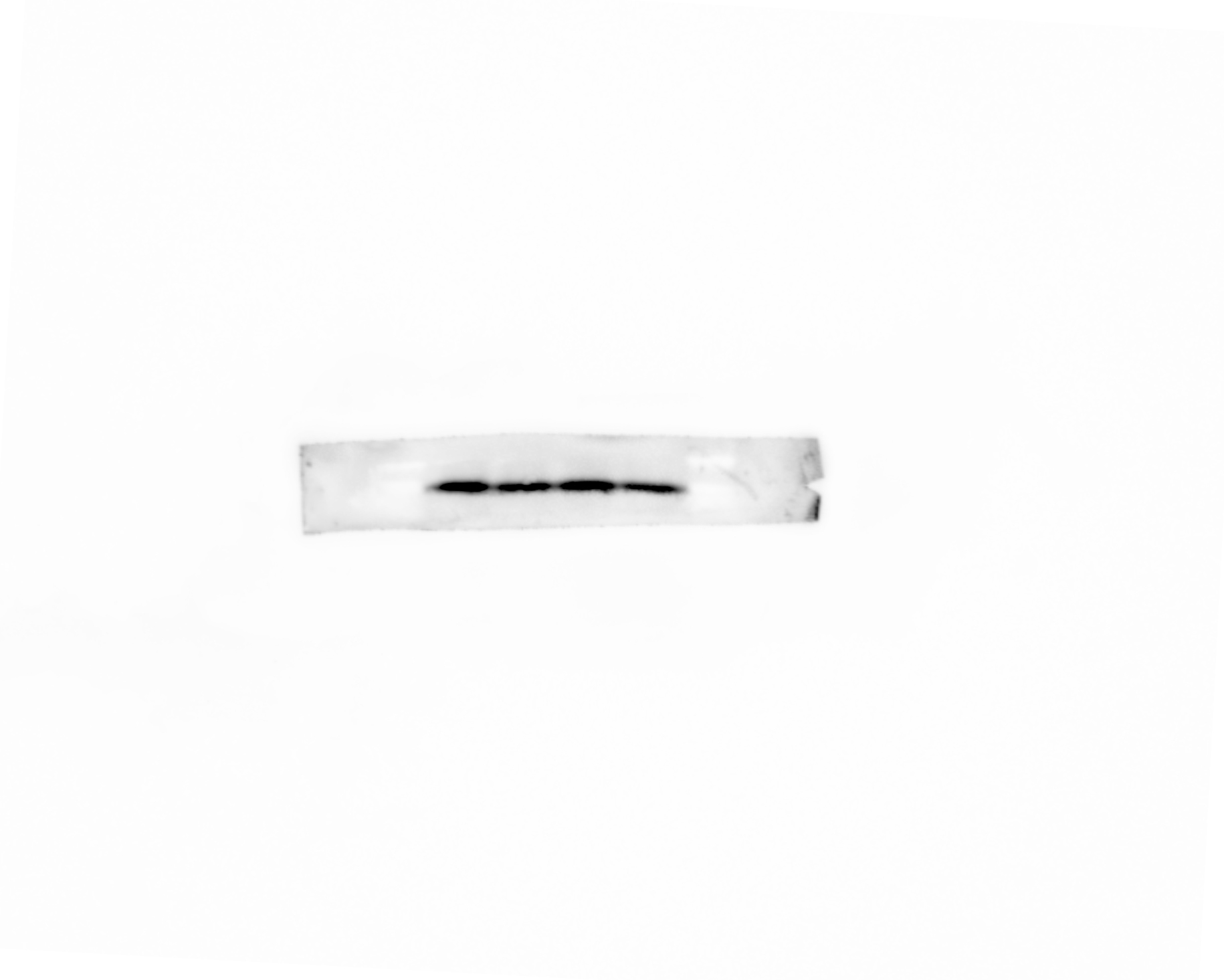

Supplement: Supplementary file 6 [file Data_Sheet_1.ZIP › Data sheet/Western Blot/CLDN-1/Jejunum/CLDN1-3.jpg]

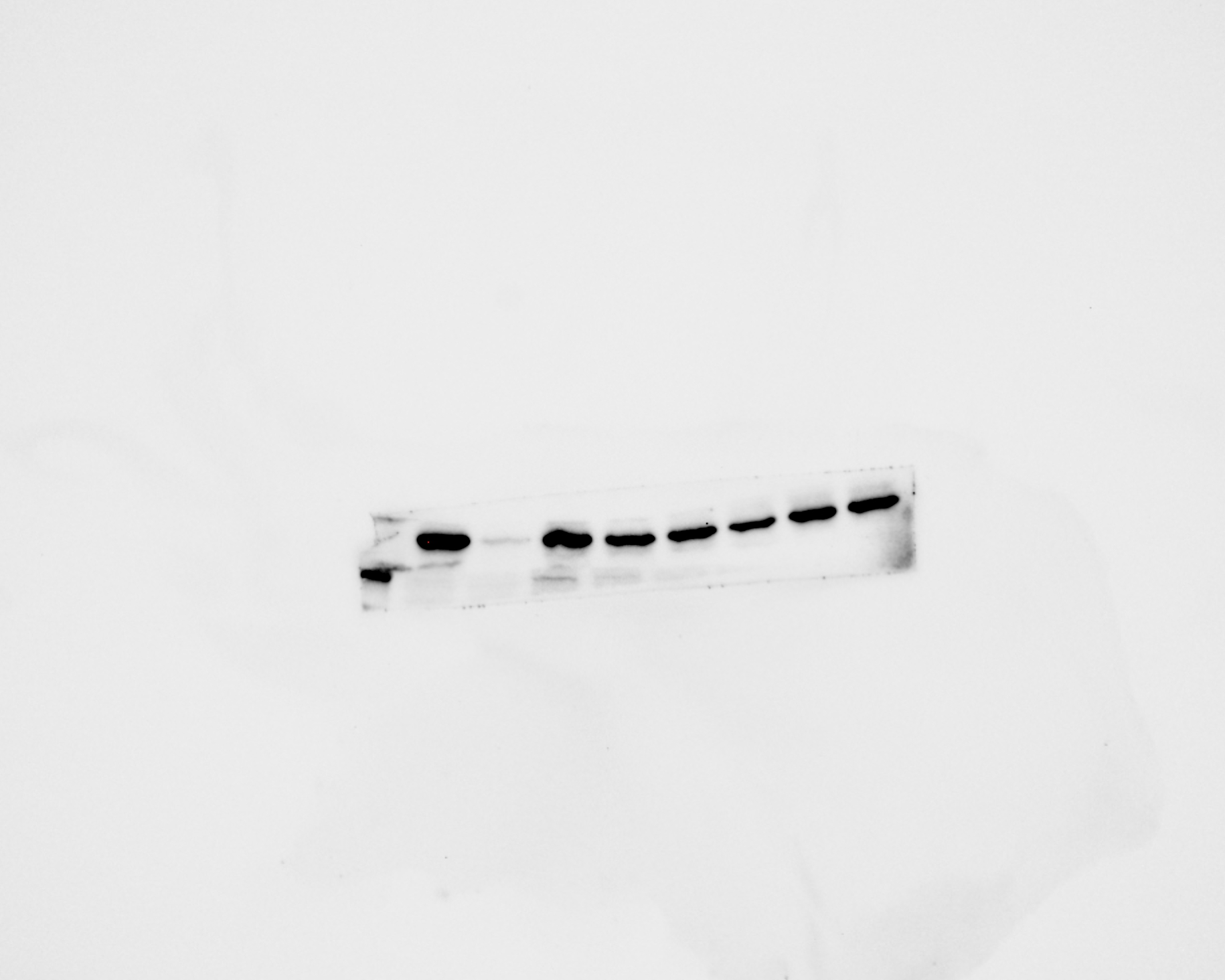

Supplement: Supplementary file 6 [file Data_Sheet_1.ZIP › Data sheet/Western Blot/Ia╩Ba┴/Ileum/IKBa┴-2,3.jpg]

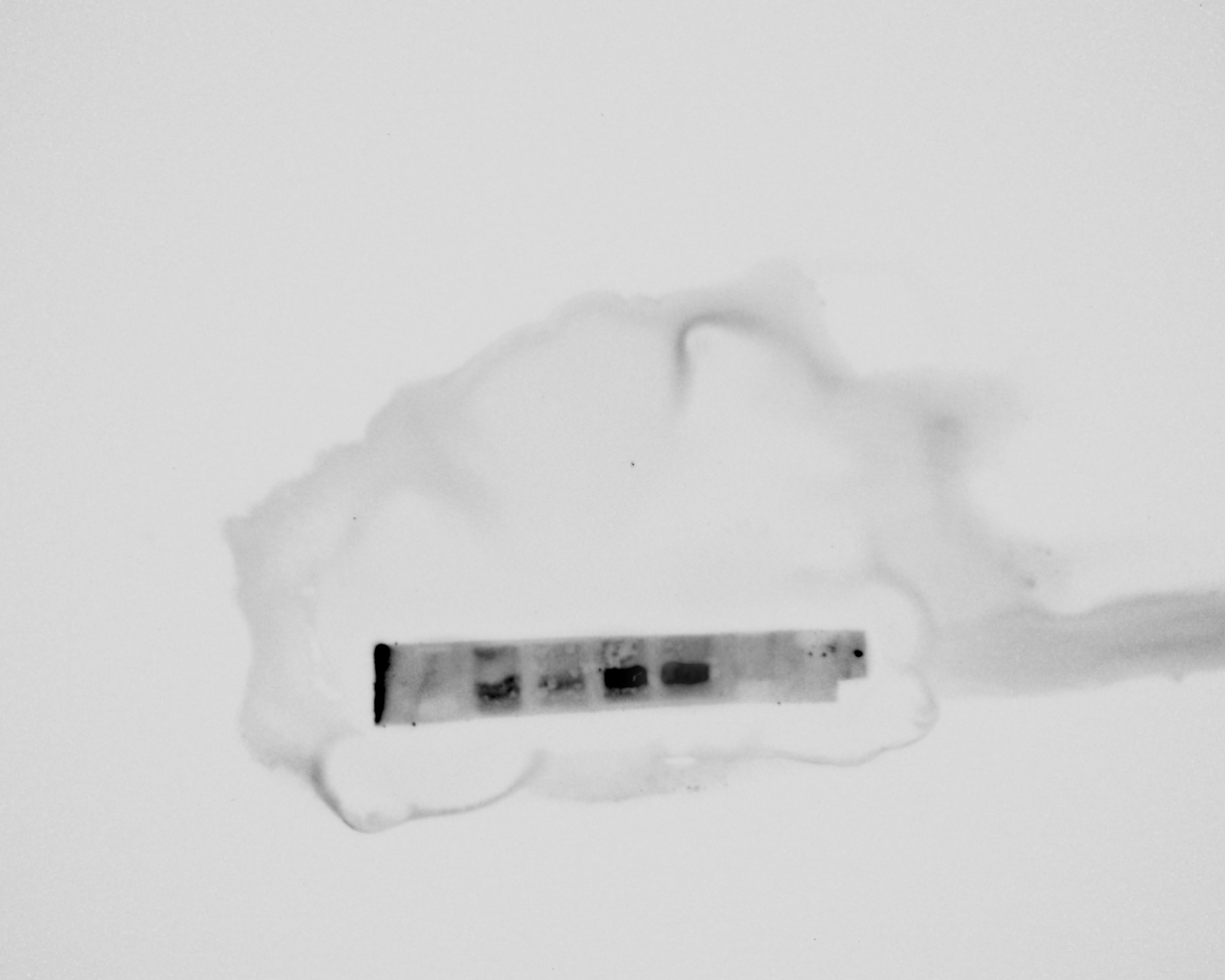

Supplement: Supplementary file 6 [file Data_Sheet_1.ZIP › Data sheet/Western Blot/Ia╩Ba┴/Ileum/Ia╩Ba┴-1.jpg]

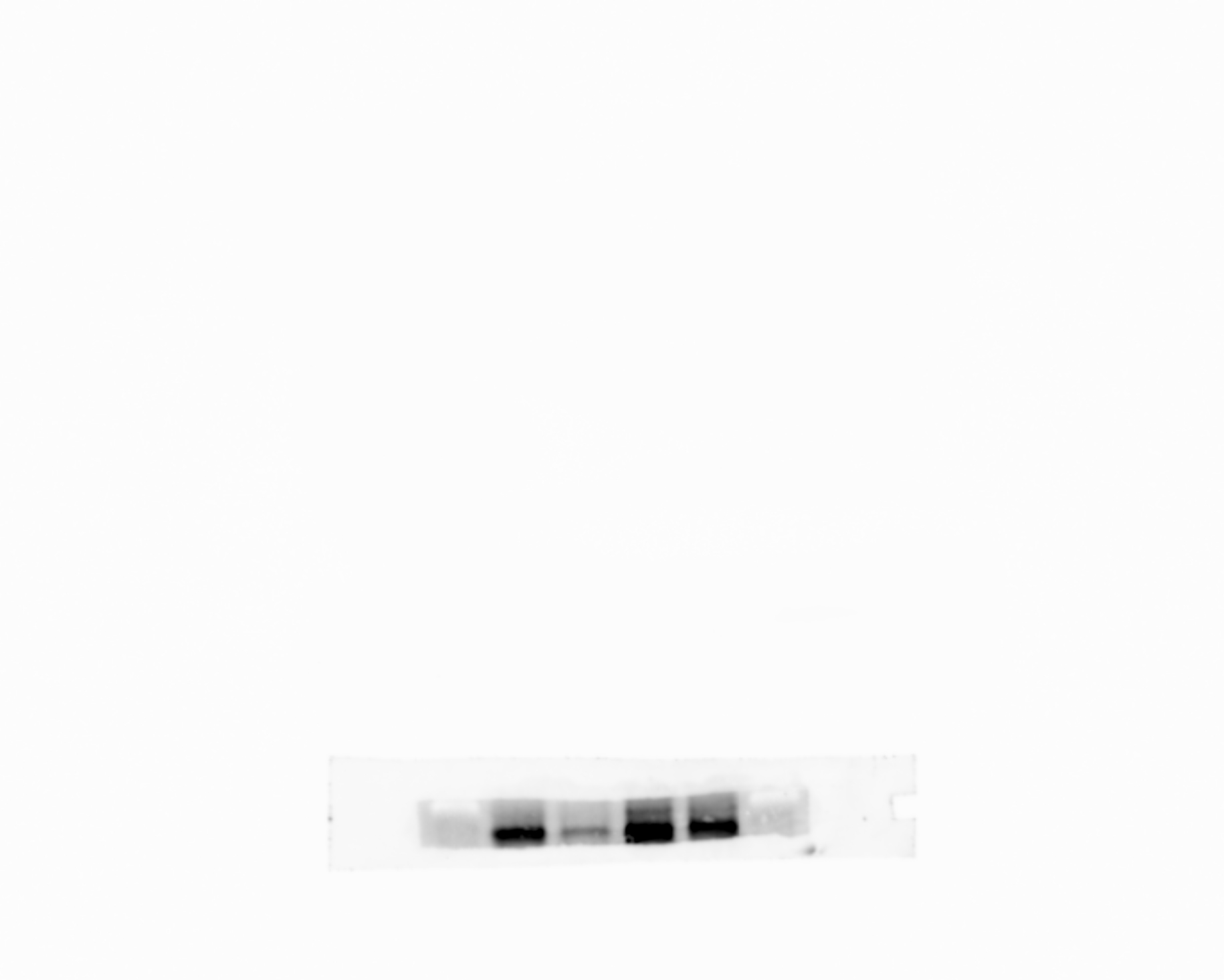

Supplement: Supplementary file 6 [file Data_Sheet_1.ZIP › Data sheet/Western Blot/Ia╩Ba┴/Jejunum/Ia╩Ba┴-1.jpg]

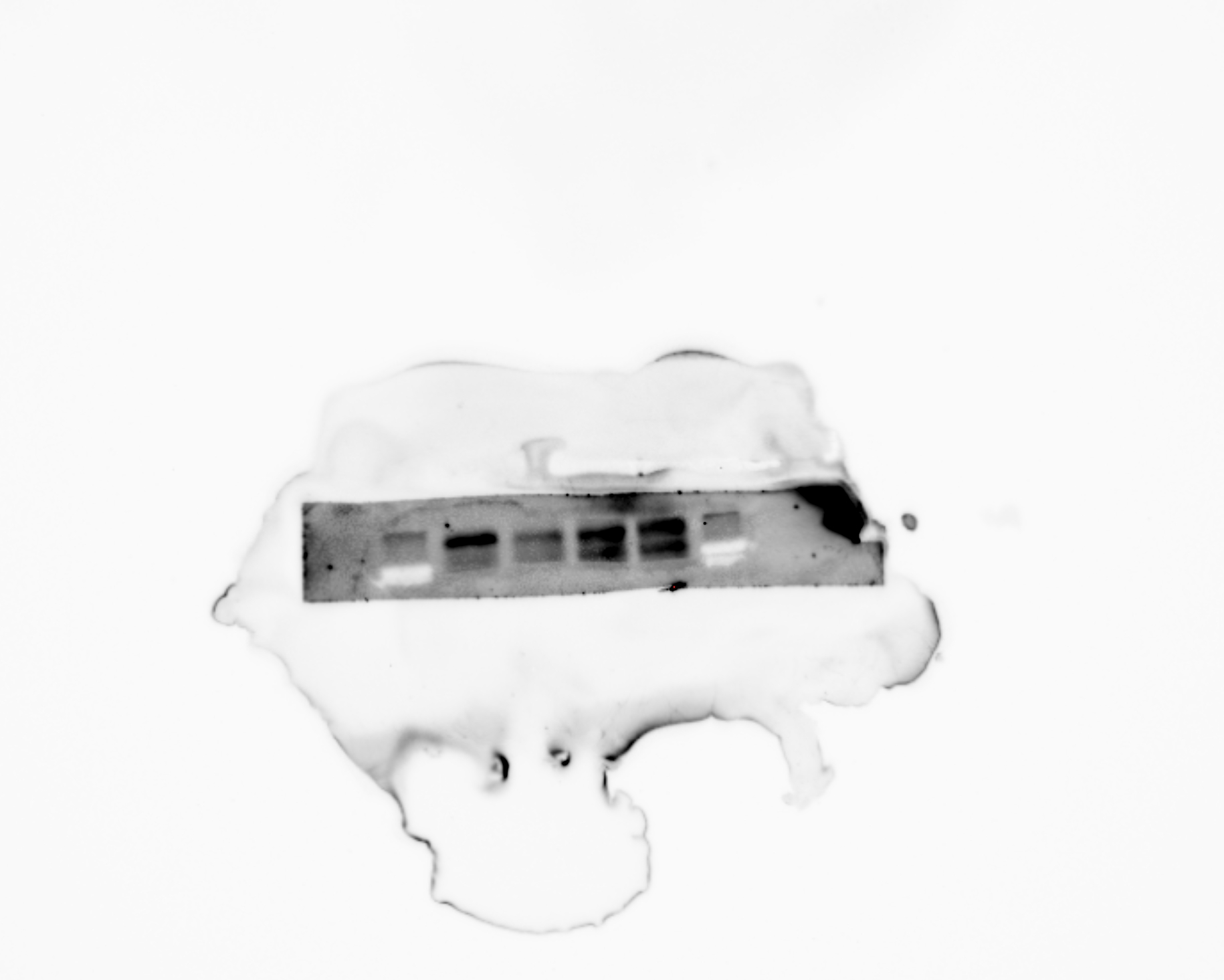

Supplement: Supplementary file 6 [file Data_Sheet_1.ZIP › Data sheet/Western Blot/Ia╩Ba┴/Jejunum/Ia╩Ba┴-2.jpg]

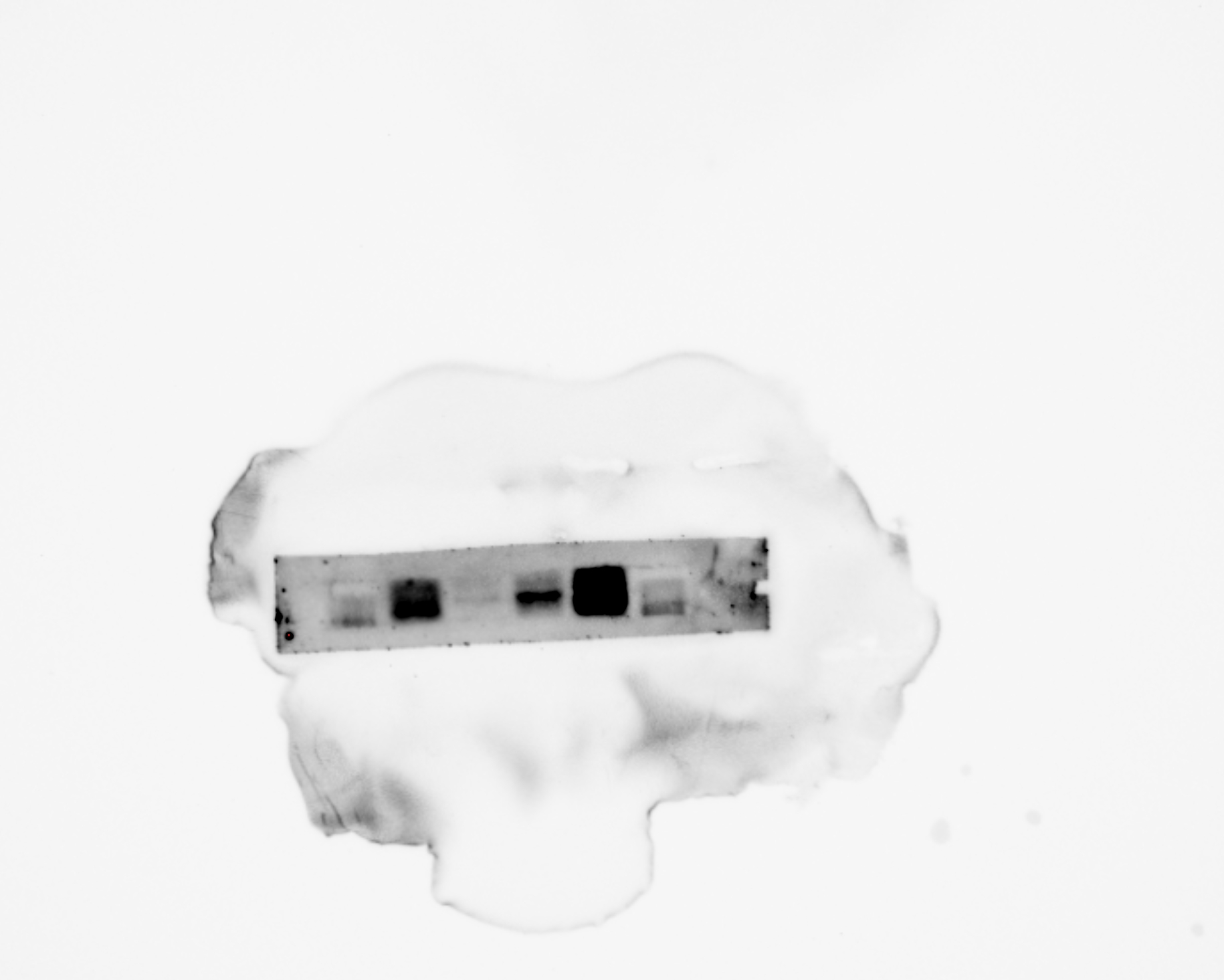

Supplement: Supplementary file 6 [file Data_Sheet_1.ZIP › Data sheet/Western Blot/Ia╩Ba┴/Jejunum/Ia╩Ba┴-3.jpg]

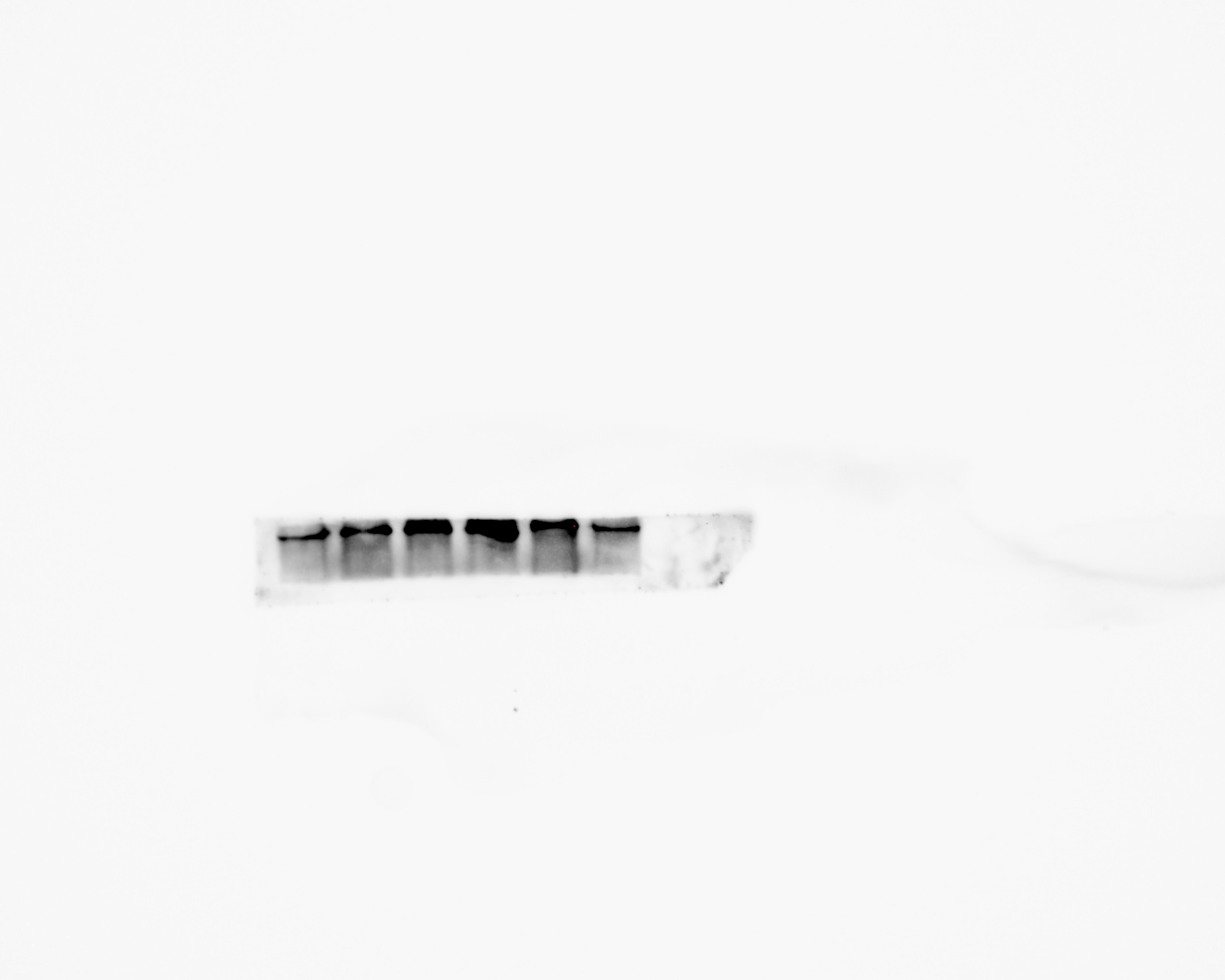

Supplement: Supplementary file 6 [file Data_Sheet_1.ZIP › Data sheet/Western Blot/NF-a╩B/Ileum/NF-a╩B 1.jpg]

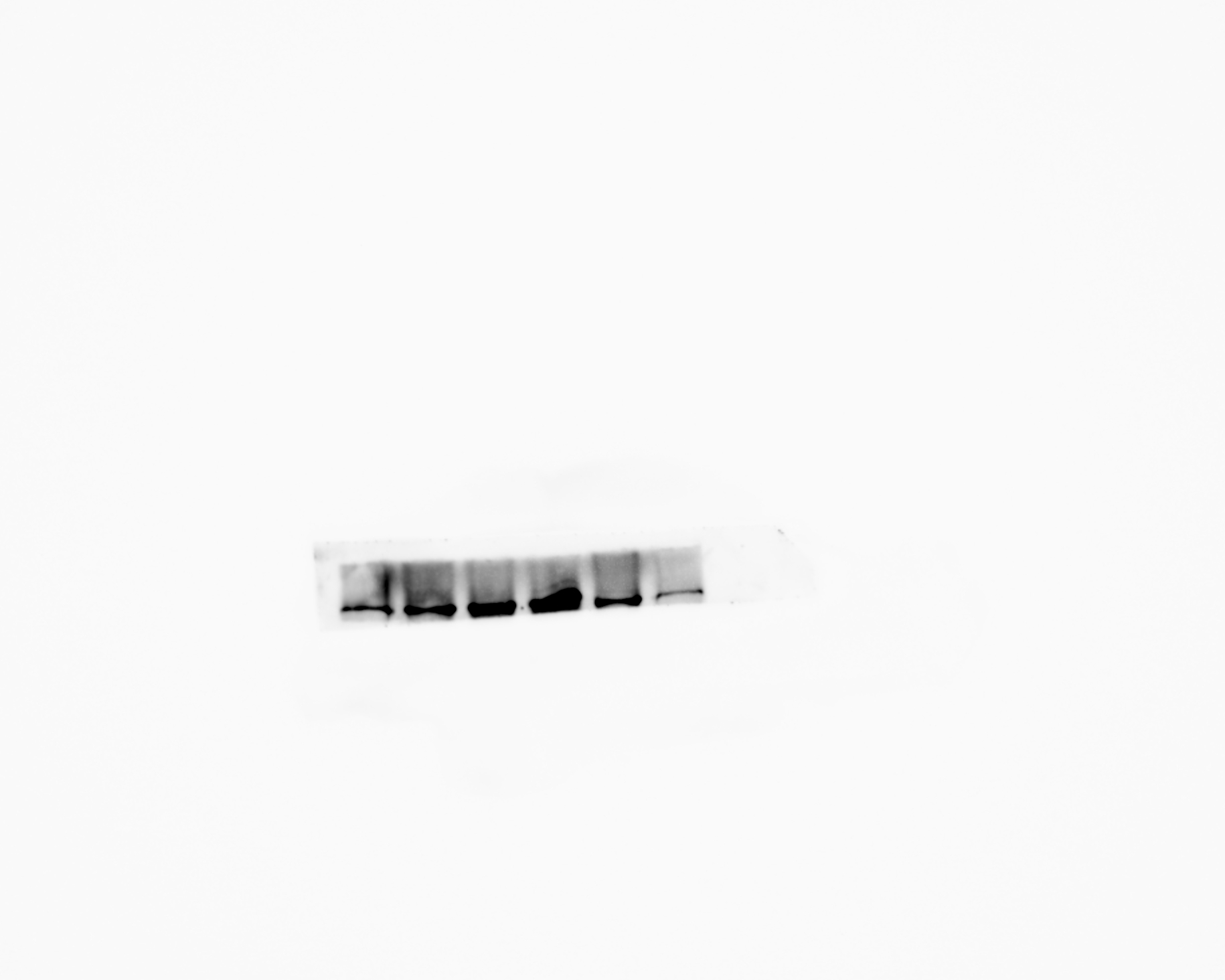

Supplement: Supplementary file 6 [file Data_Sheet_1.ZIP › Data sheet/Western Blot/NF-a╩B/Ileum/NF-a╩B 2.jpg]

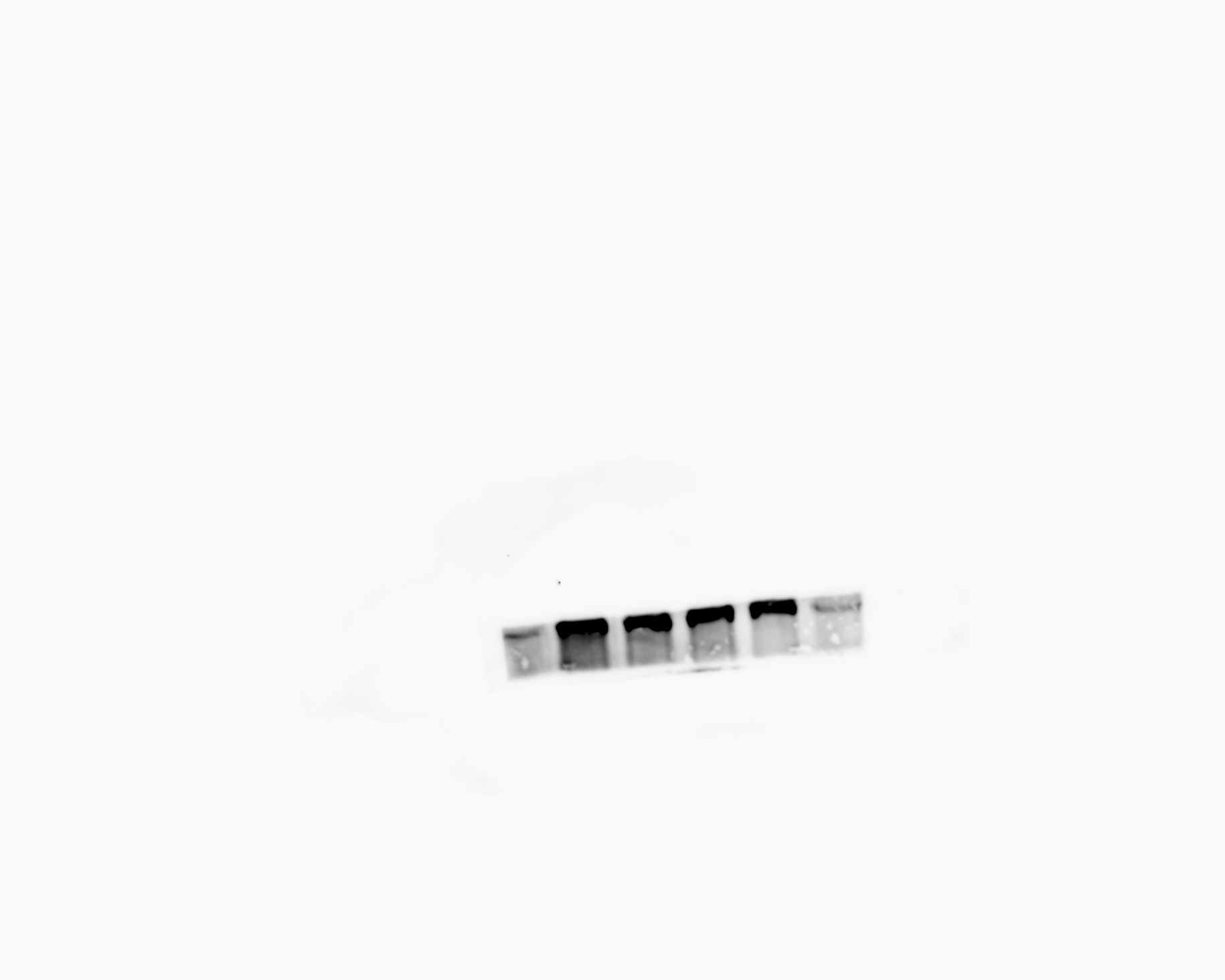

Supplement: Supplementary file 6 [file Data_Sheet_1.ZIP › Data sheet/Western Blot/NF-a╩B/Ileum/NF-a╩B 3.jpg]

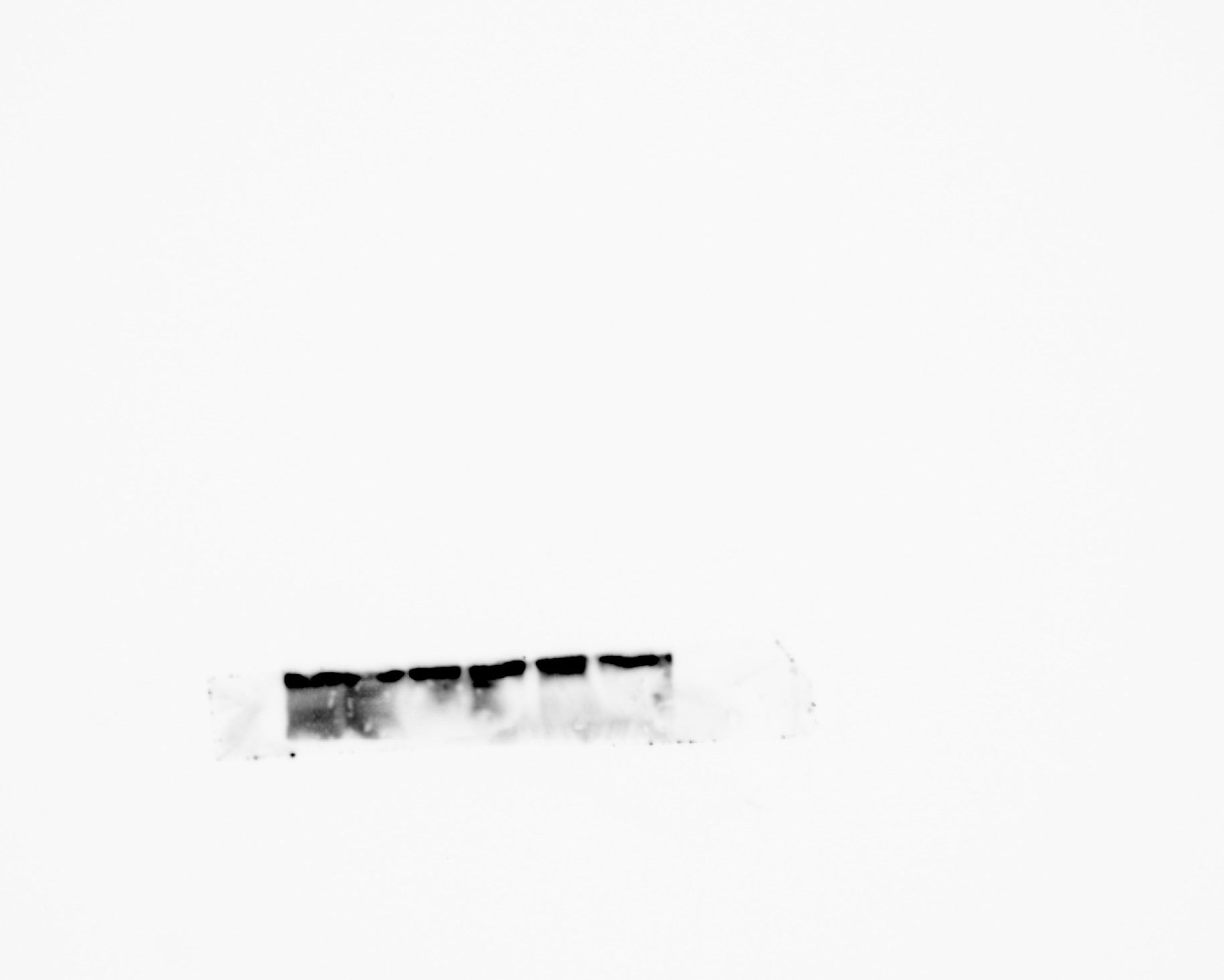

Supplement: Supplementary file 6 [file Data_Sheet_1.ZIP › Data sheet/Western Blot/NF-a╩B/Jejunum/NF-a╩B 1.jpg]

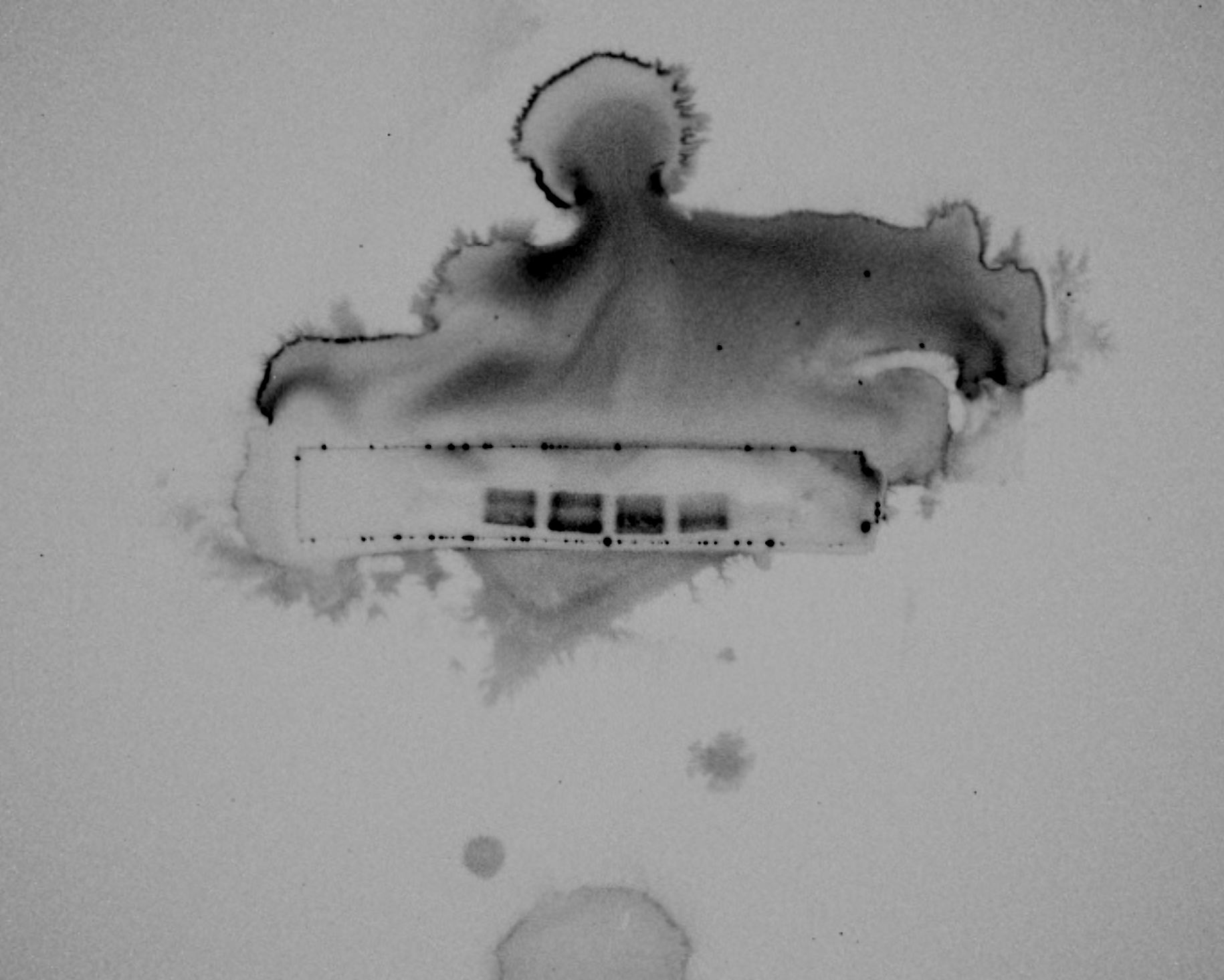

Supplement: Supplementary file 6 [file Data_Sheet_1.ZIP › Data sheet/Western Blot/NF-a╩B/Jejunum/NF-a╩B 2.jpg]

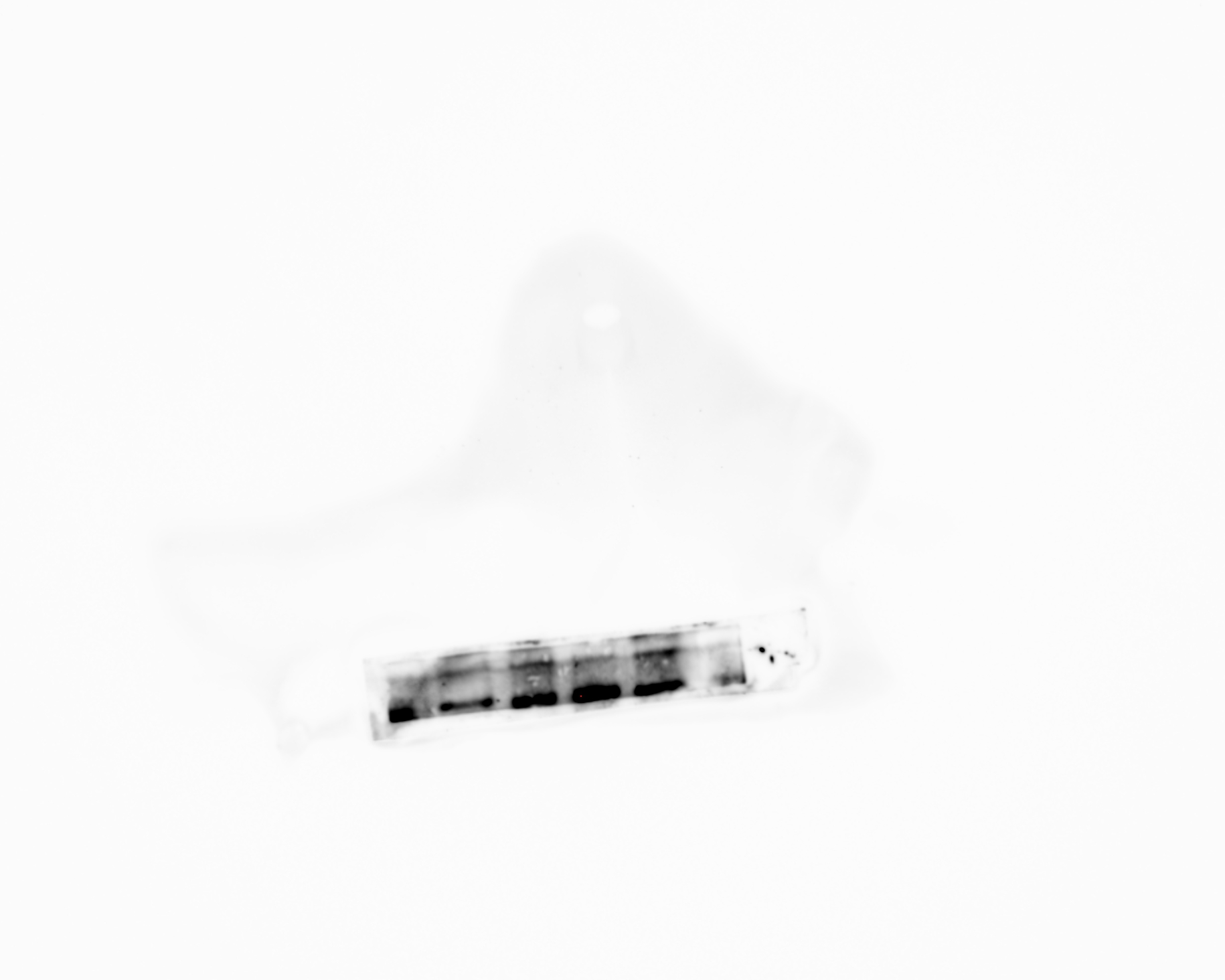

Supplement: Supplementary file 6 [file Data_Sheet_1.ZIP › Data sheet/Western Blot/NF-a╩B/Jejunum/NF-a╩B 3.jpg]

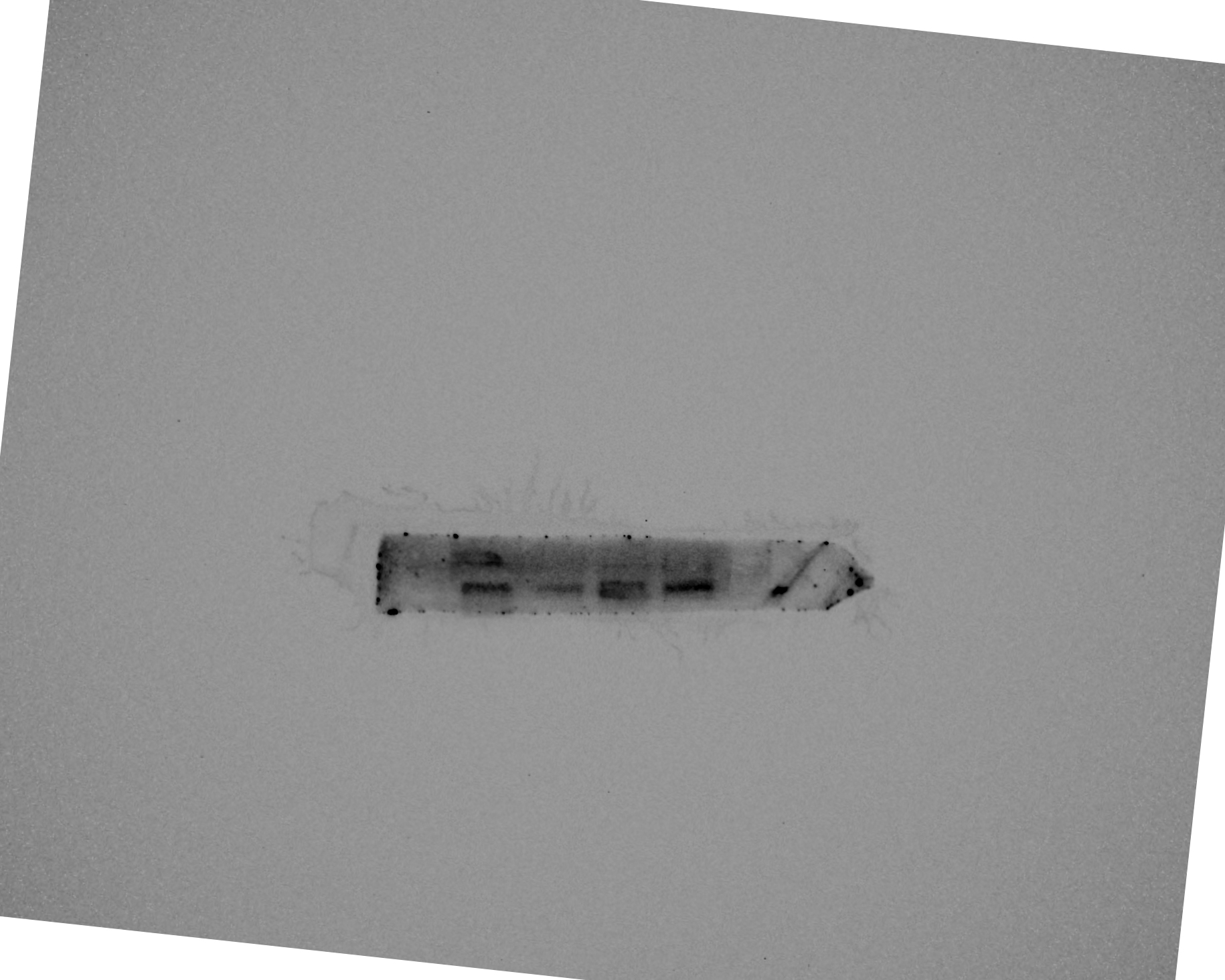

Supplement: Supplementary file 6 [file Data_Sheet_1.ZIP › Data sheet/Western Blot/Occludin/Ileum/Occludin 1.jpg]

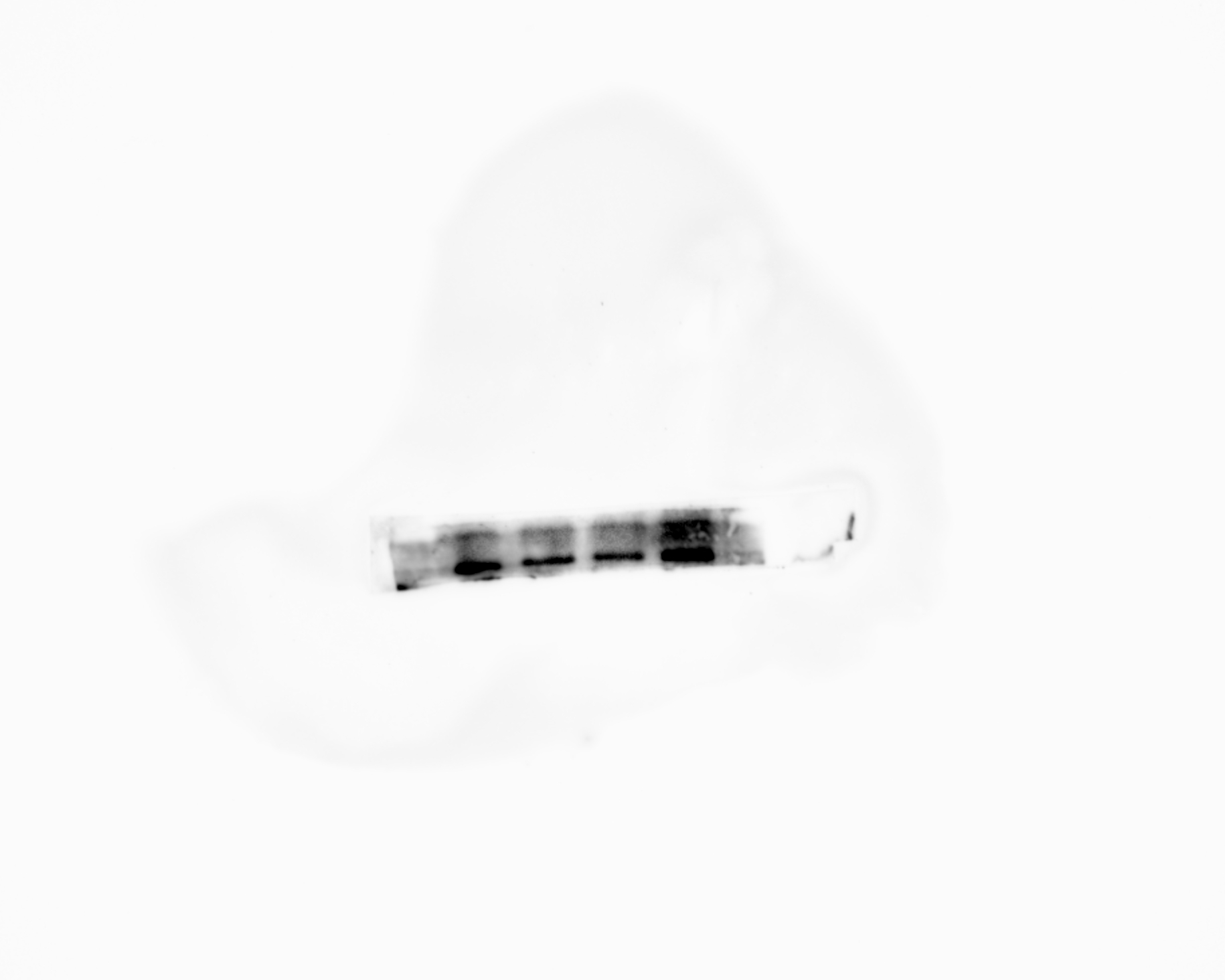

Supplement: Supplementary file 6 [file Data_Sheet_1.ZIP › Data sheet/Western Blot/Occludin/Ileum/Occludin 3.jpg]

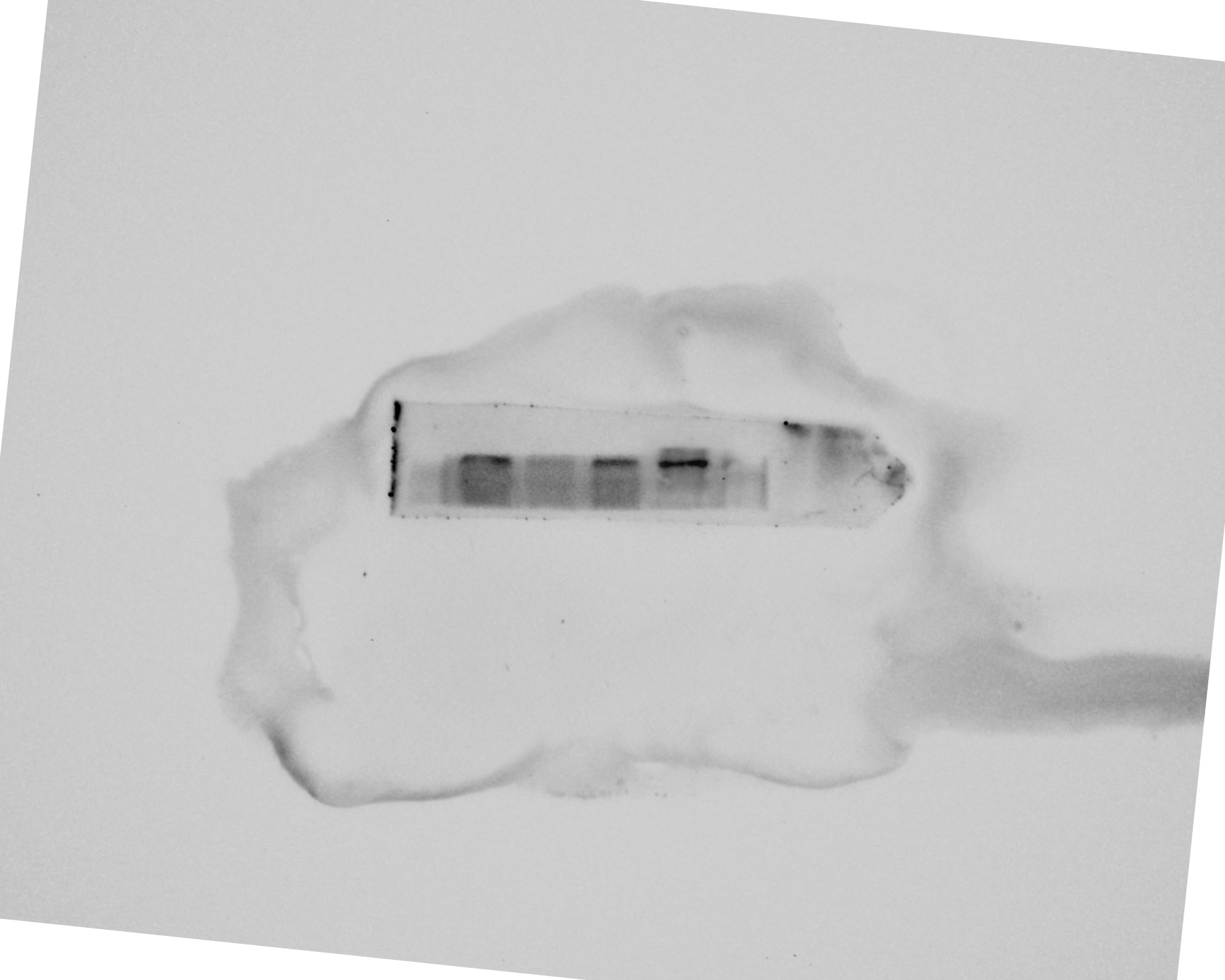

Supplement: Supplementary file 6 [file Data_Sheet_1.ZIP › Data sheet/Western Blot/Occludin/Ileum/occludin 2.jpg]

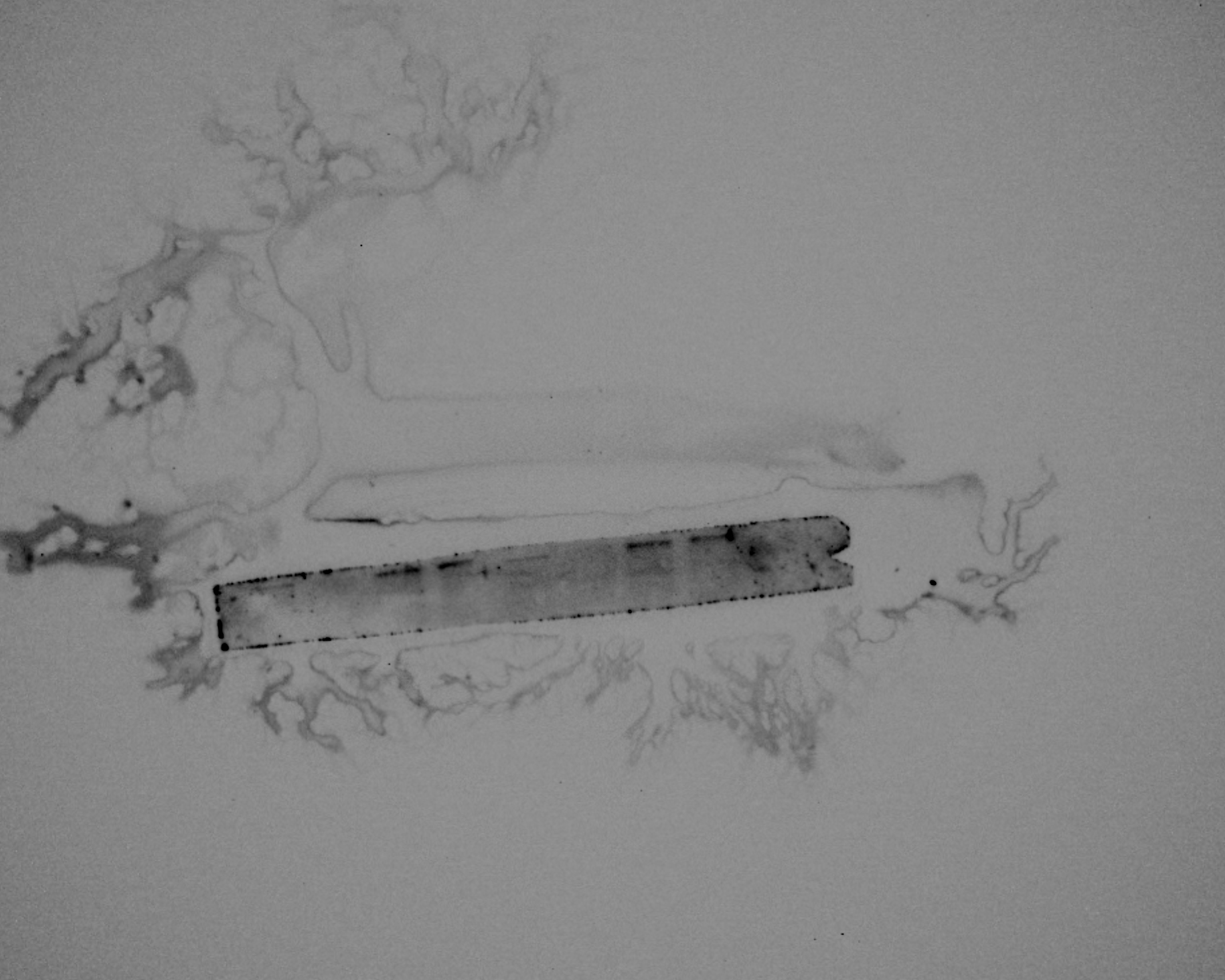

Supplement: Supplementary file 6 [file Data_Sheet_1.ZIP › Data sheet/Western Blot/Occludin/Jejunum/Occludin 1.jpg]

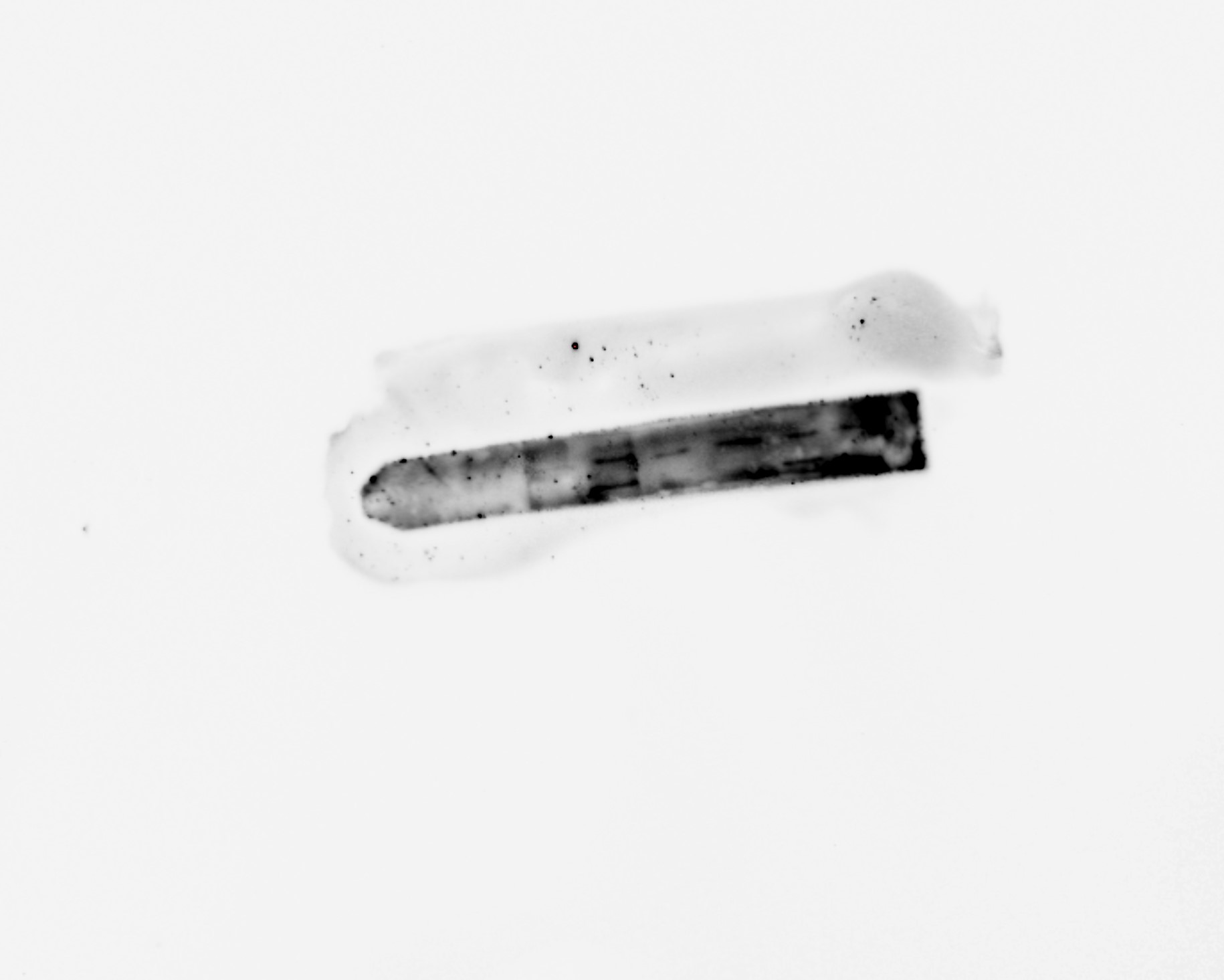

Supplement: Supplementary file 6 [file Data_Sheet_1.ZIP › Data sheet/Western Blot/Occludin/Jejunum/Occludin2.jpg]

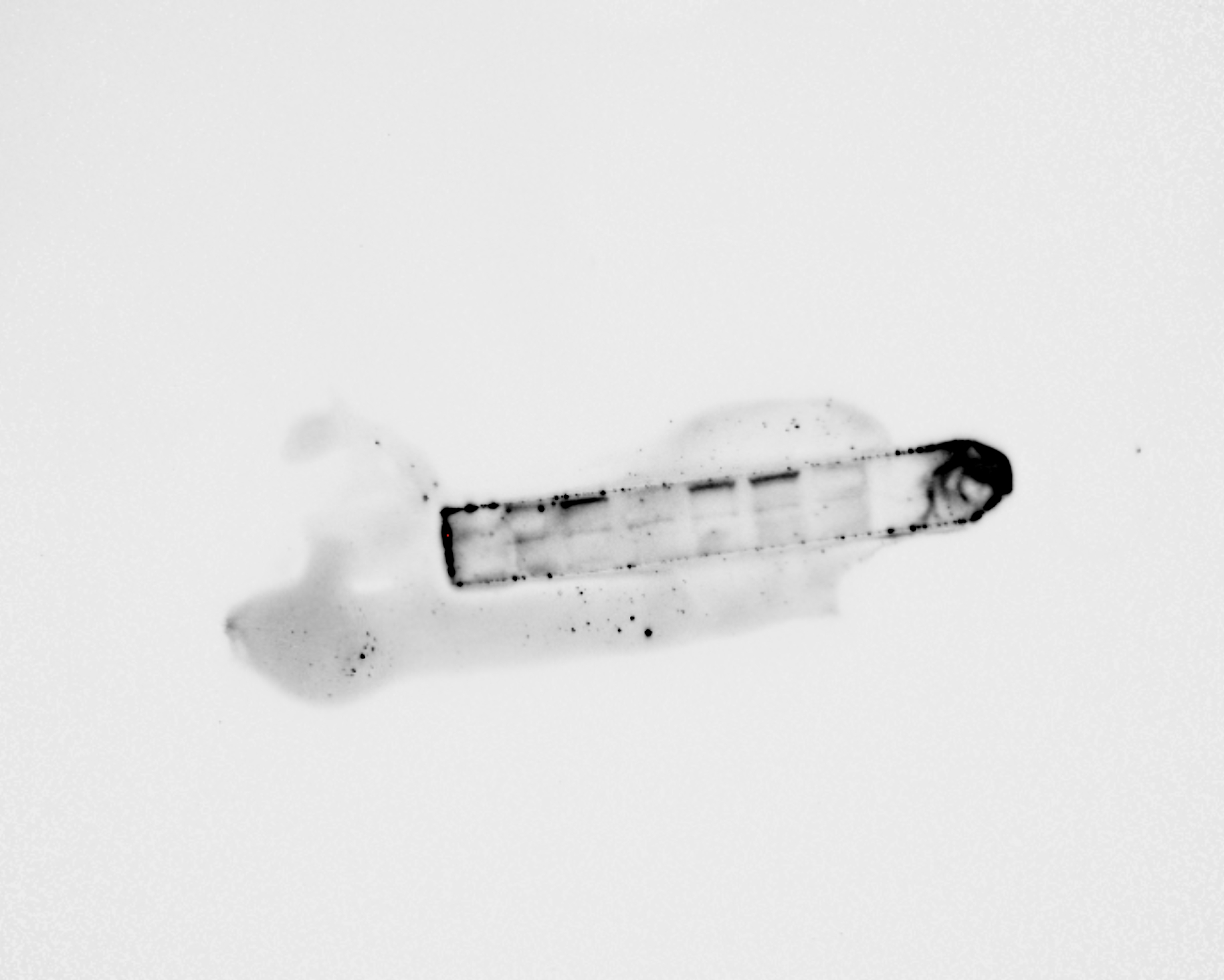

Supplement: Supplementary file 6 [file Data_Sheet_1.ZIP › Data sheet/Western Blot/Occludin/Jejunum/Occludin3.jpg]

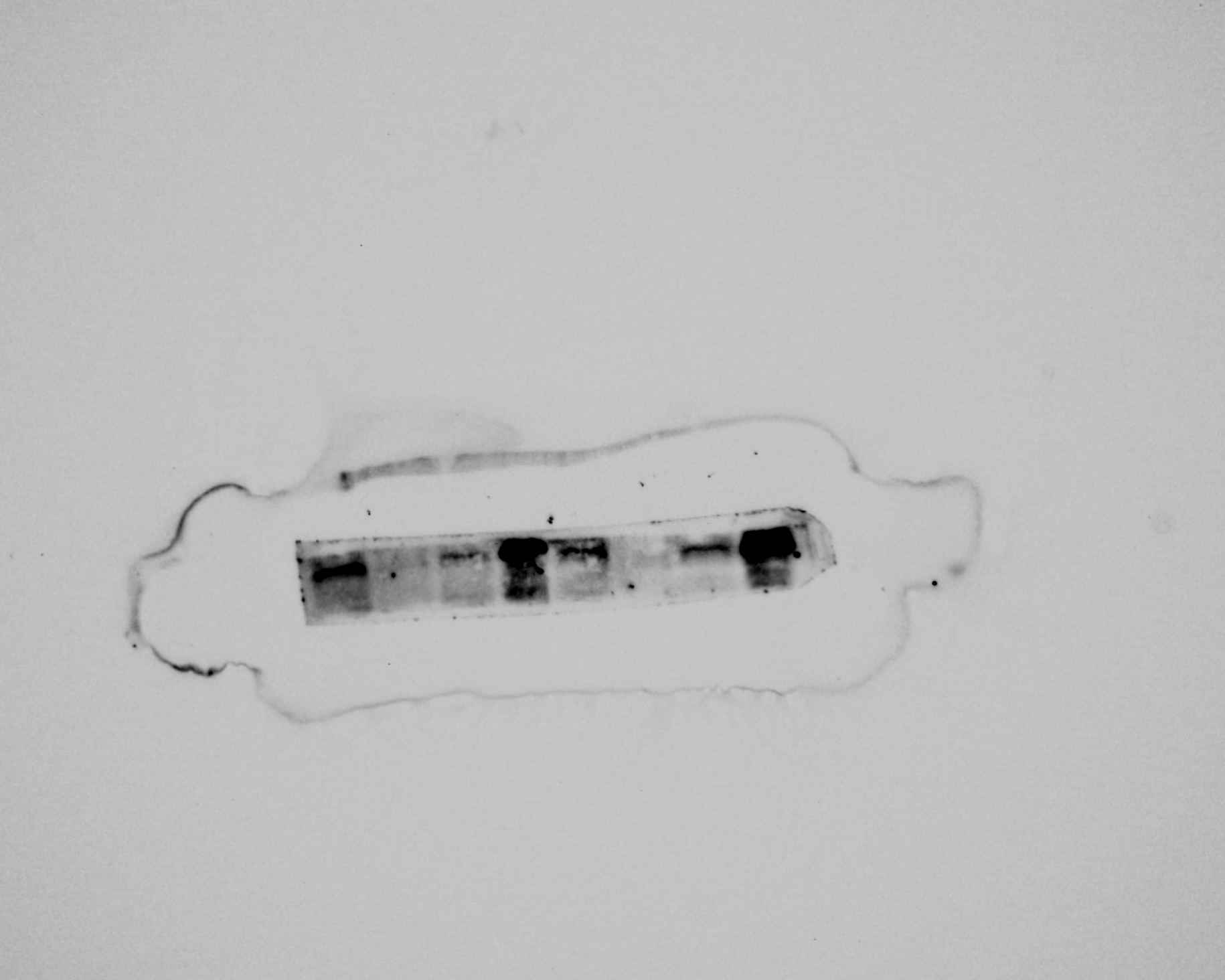

Supplement: Supplementary file 6 [file Data_Sheet_1.ZIP › Data sheet/Western Blot/ZO-1/Ileum/ZO-1 1,2.jpg]

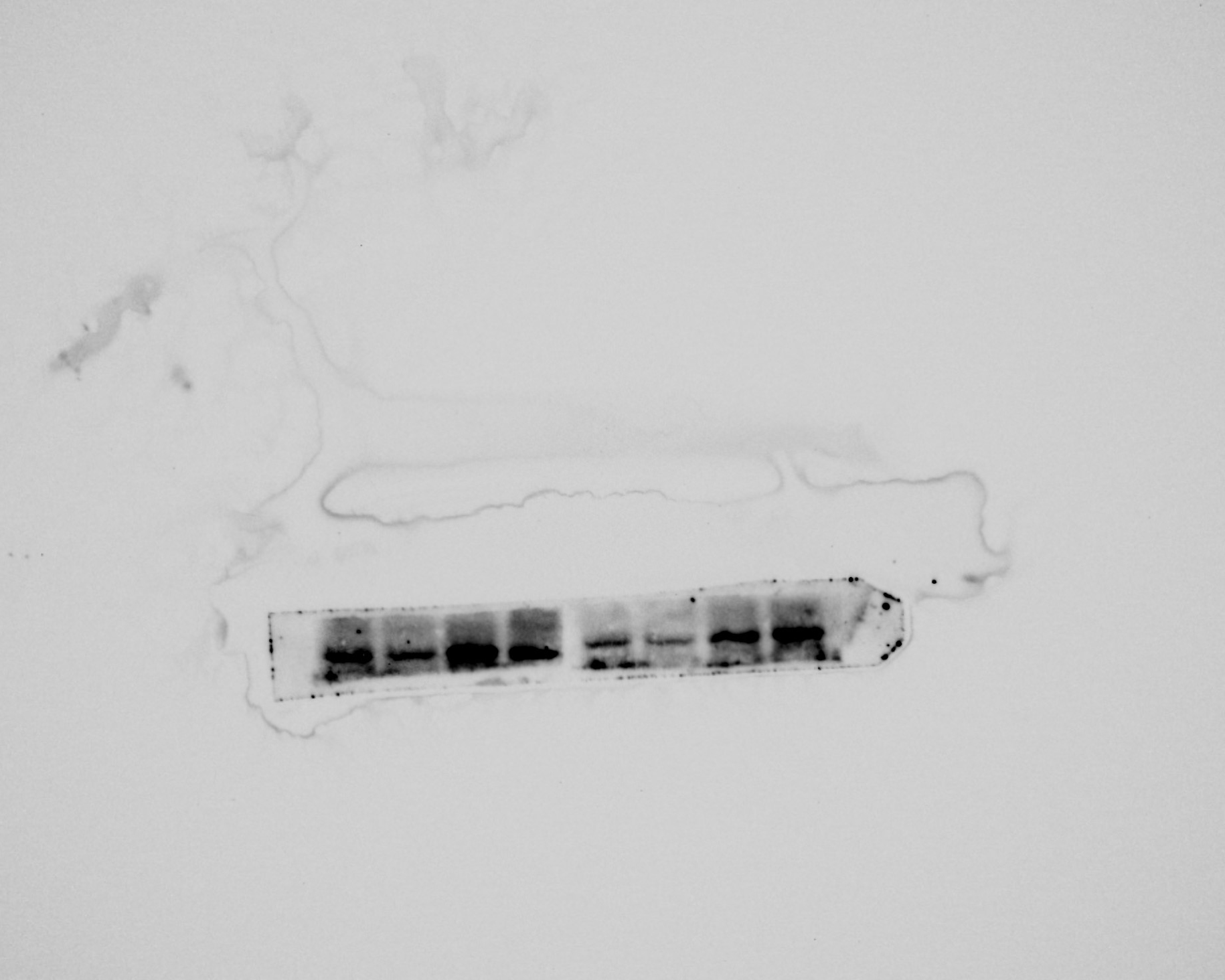

Supplement: Supplementary file 6 [file Data_Sheet_1.ZIP › Data sheet/Western Blot/ZO-1/Ileum/ZO-1 3,4.jpg]

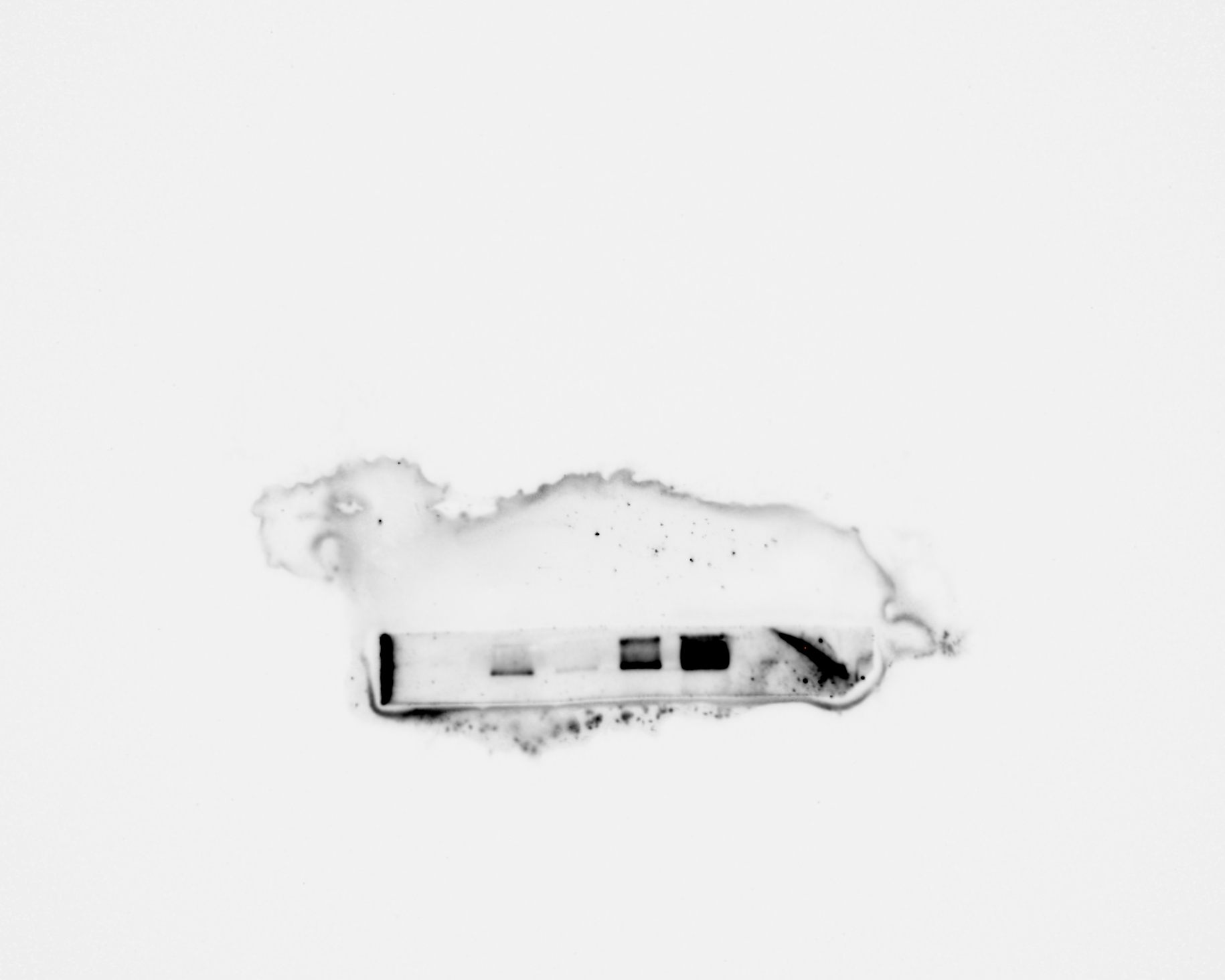

Supplement: Supplementary file 6 [file Data_Sheet_1.ZIP › Data sheet/Western Blot/ZO-1/Jejunum/ZO-1 1.jpg]

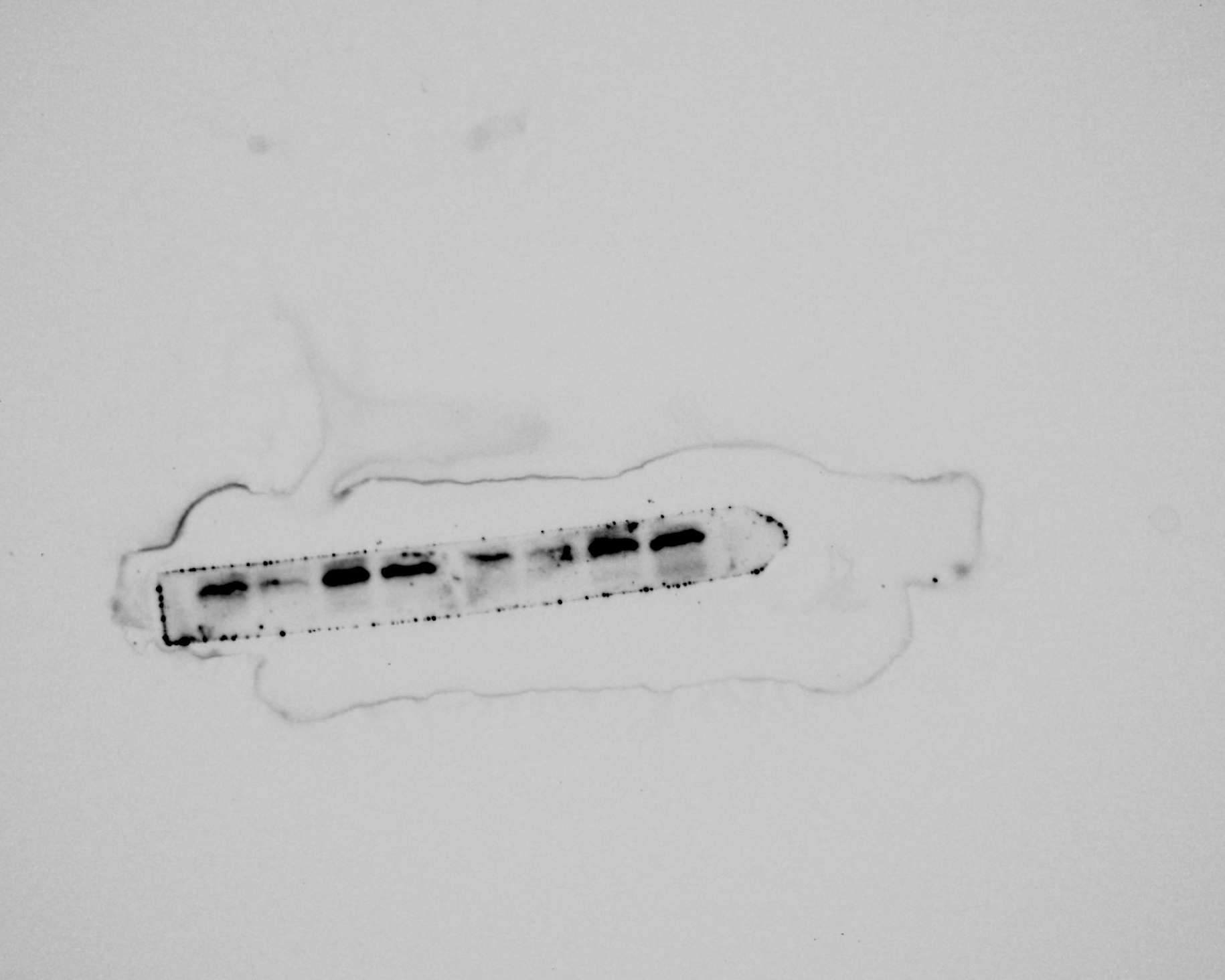

Supplement: Supplementary file 6 [file Data_Sheet_1.ZIP › Data sheet/Western Blot/ZO-1/Jejunum/ZO-1 2,3.jpg]

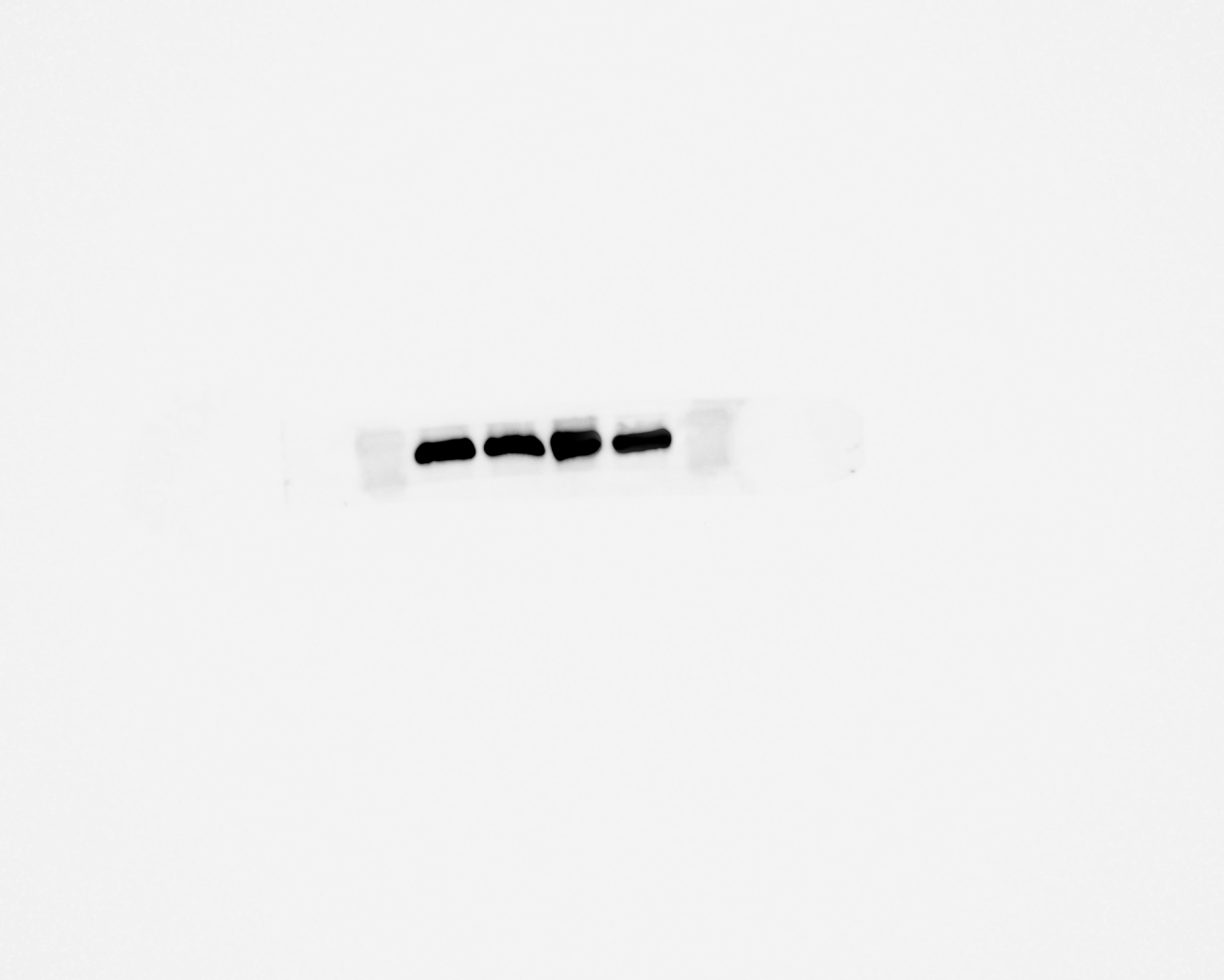

Supplement: Supplementary file 6 [file Data_Sheet_1.ZIP › Data sheet/Western Blot/a┬-actin/Ileum/a┬-actin 1.jpg]

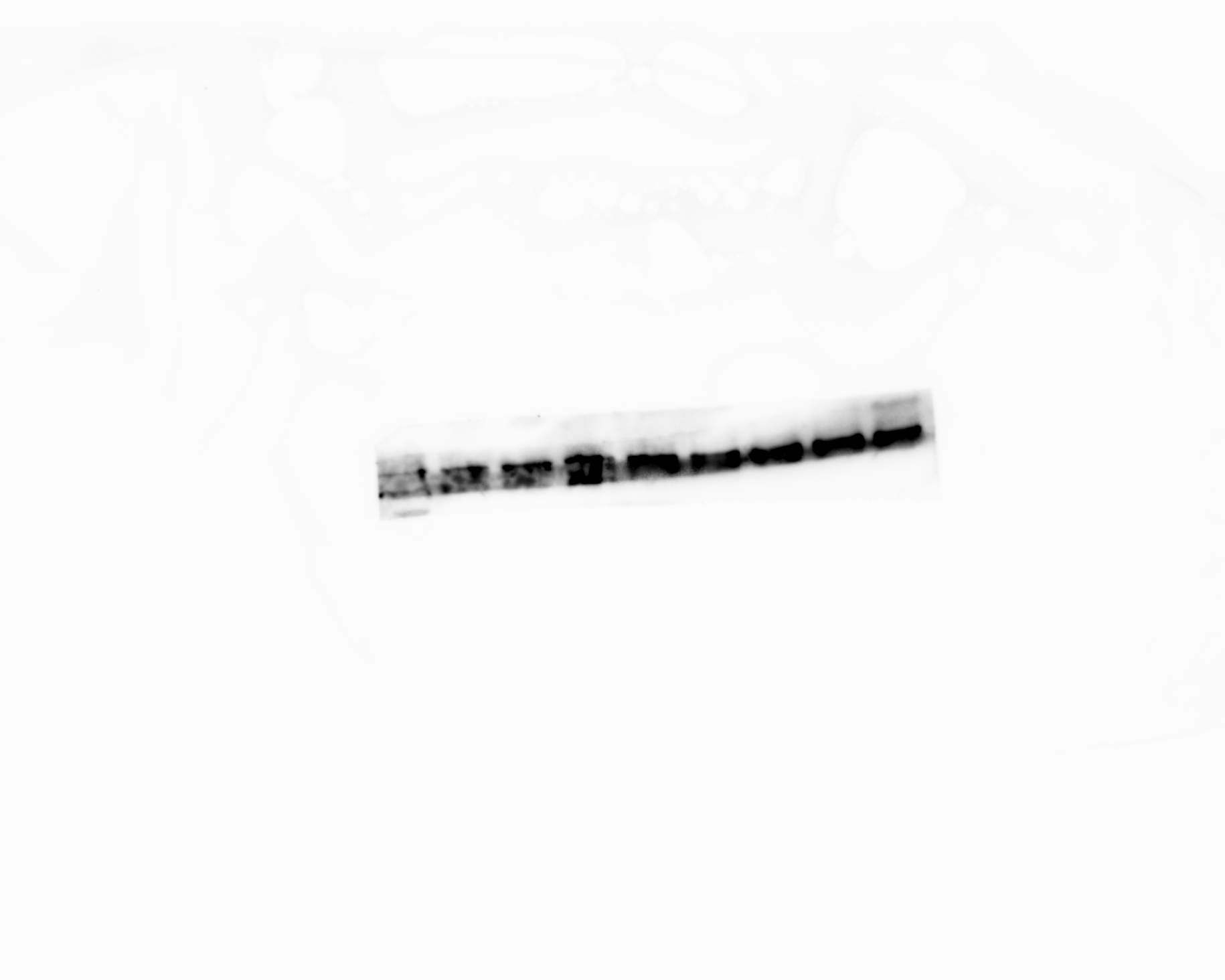

Supplement: Supplementary file 6 [file Data_Sheet_1.ZIP › Data sheet/Western Blot/a┬-actin/Ileum/a┬-actin 2.jpg]

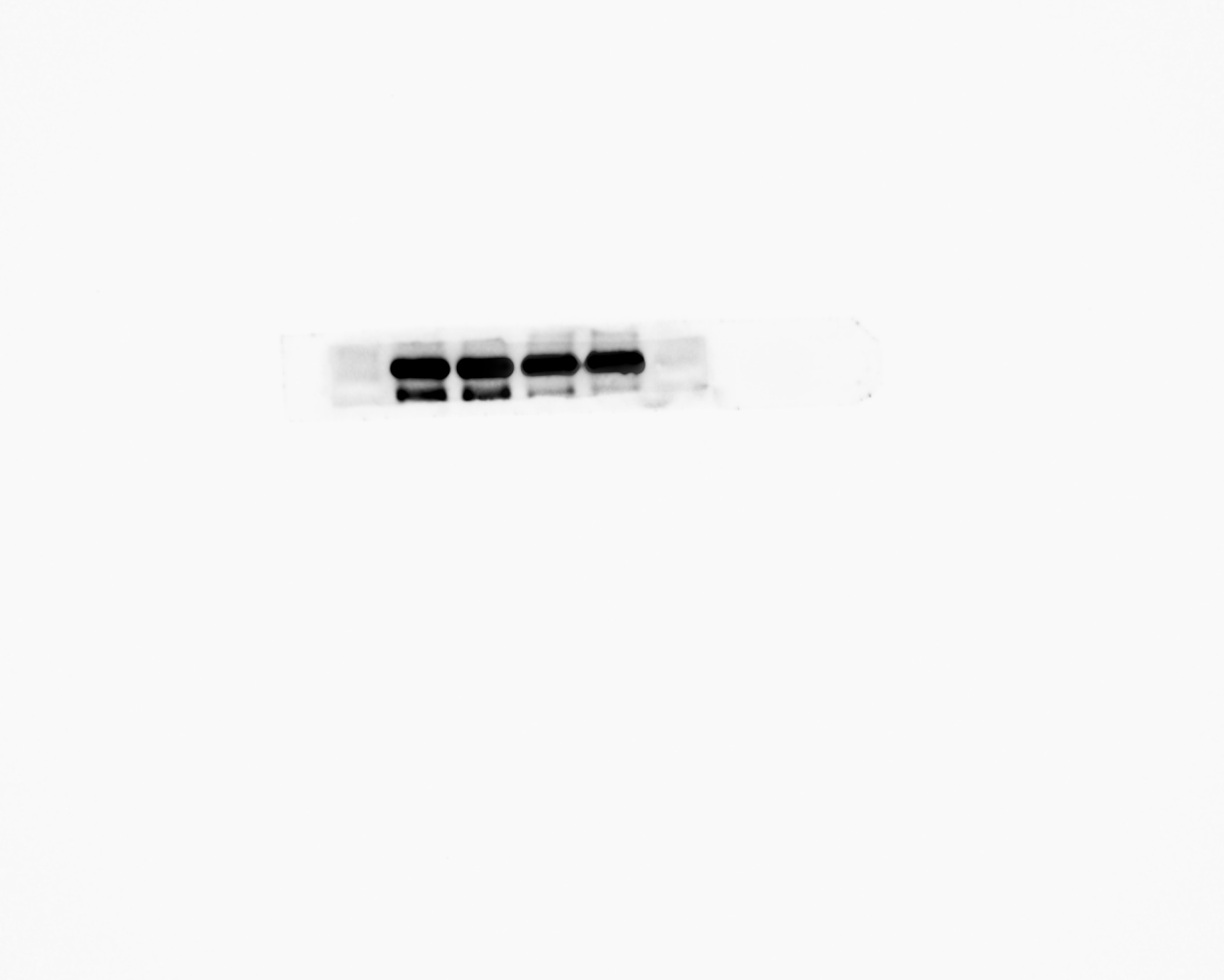

Supplement: Supplementary file 6 [file Data_Sheet_1.ZIP › Data sheet/Western Blot/a┬-actin/Jejunum/a┬-actin 1.jpg]

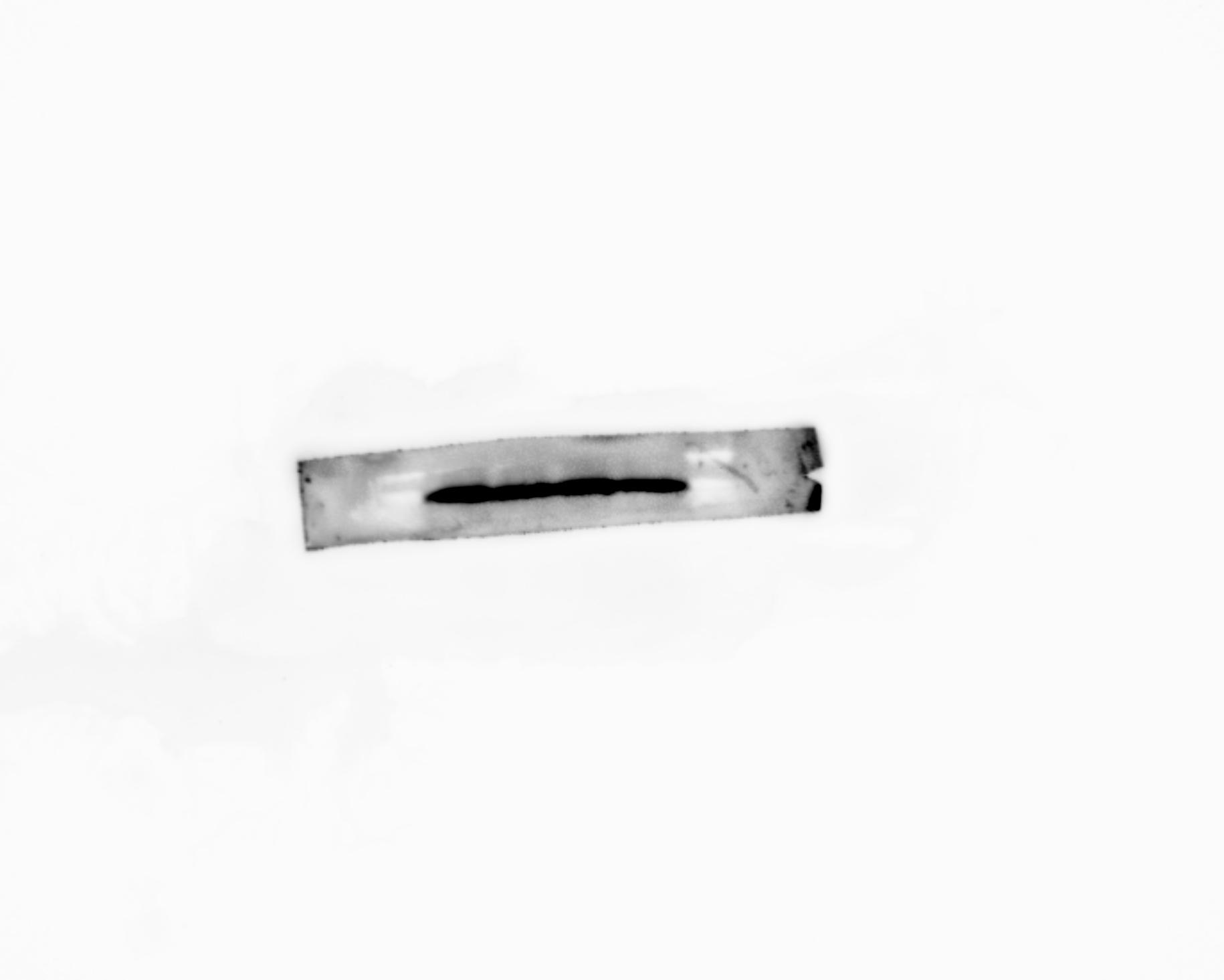

Supplement: Supplementary file 6 [file Data_Sheet_1.ZIP › Data sheet/Western Blot/a┬-actin/Jejunum/a┬-actin 2.jpg]

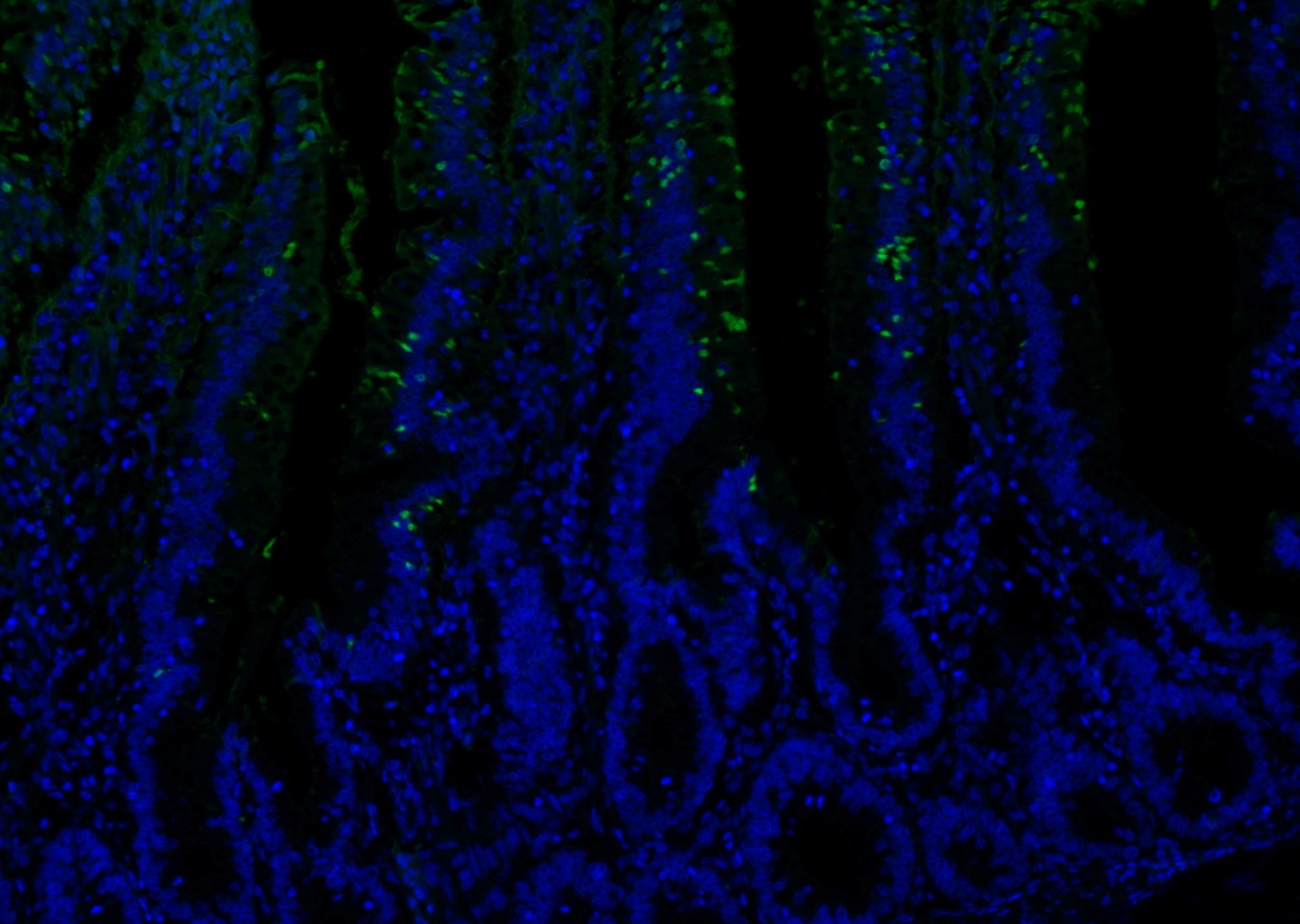

Supplement: Supplementary file 7 [file Data_Sheet_2.ZIP › CON group-Jejunal TUNEL apoptosis/200 x/CON-1 200-1 2.jpg]

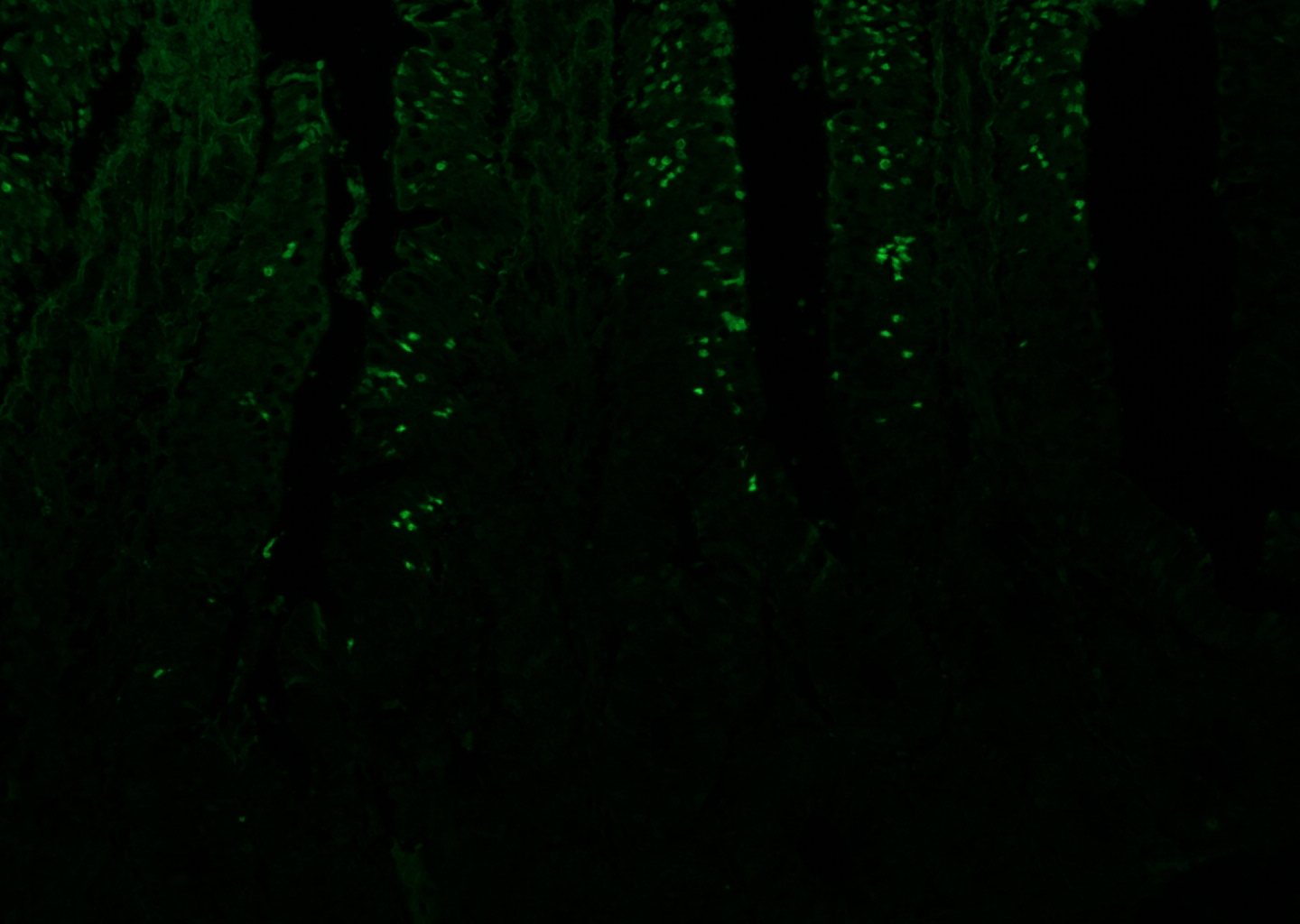

Supplement: Supplementary file 7 [file Data_Sheet_2.ZIP › CON group-Jejunal TUNEL apoptosis/200 x/CON-1 200-1.jpg]

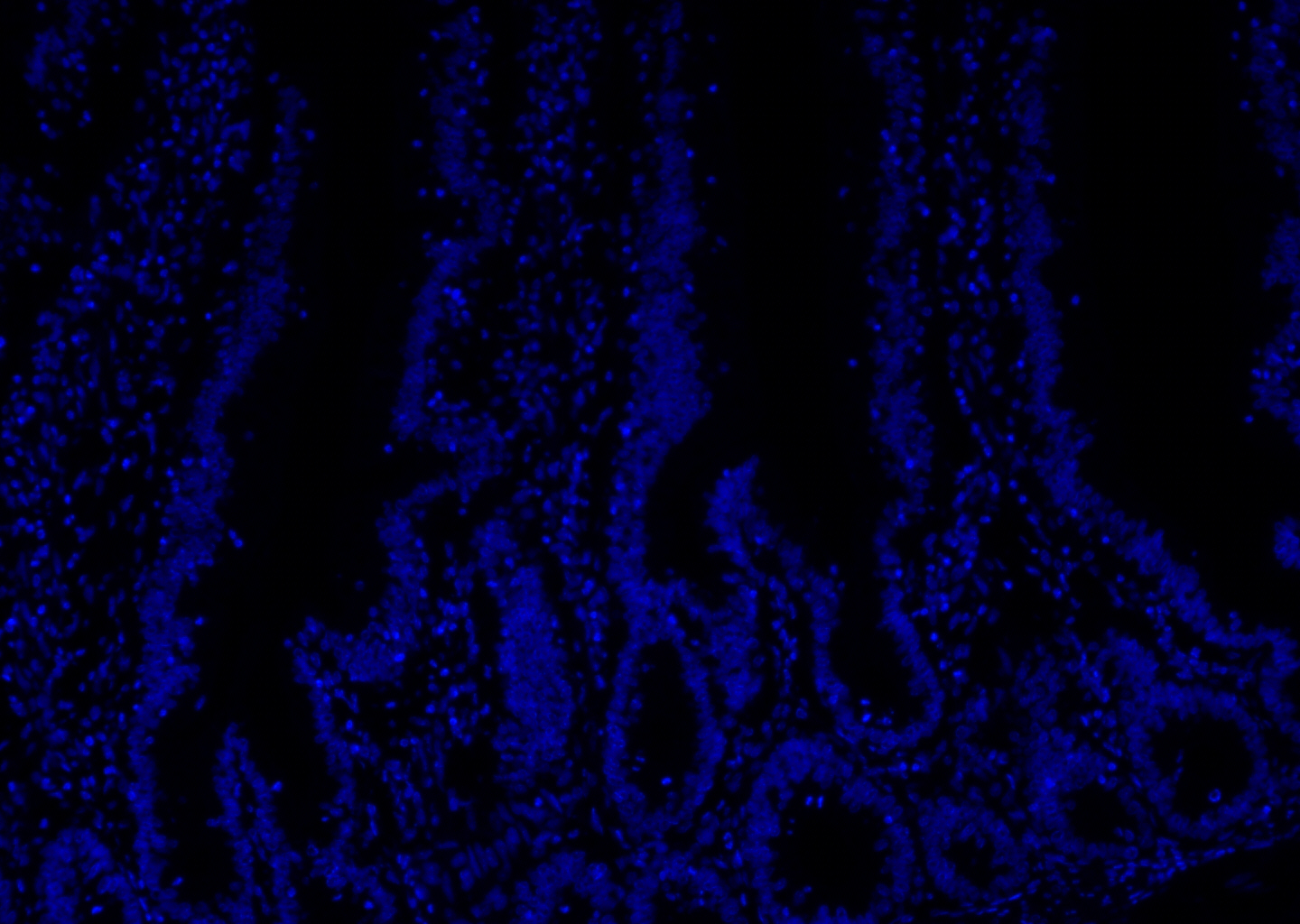

Supplement: Supplementary file 7 [file Data_Sheet_2.ZIP › CON group-Jejunal TUNEL apoptosis/200 x/CON-1 200-2.jpg]
