# Supplementary material for: Trans-Anethole Alleviates Subclinical Necro-Haemorrhagic Enteritis-Induced Intestinal Barrier Dysfunction and Intestinal Inflammation in Broilers
Source: Front Microbiol. 2022 Mar 21;13:831882. doi: 10.3389/fmicb.2022.831882 (PMC8977854; doi:10.3389/fmicb.2022.831882)
Supplement: Supplementary file 3 [file Table_1.DOCX]

**Supplementary Table 1**. Ingredients and nutrient composition of the basal diet^1^ (%, as fed-basis)

| Ingredient | % | Nutrient levels^1^ | % |
| --- | --- | --- | --- |
| Corn | 55.60 | Metabolizable energy, Mcal/kg | 2.87 |
| Expanded soybean meal | 29.00 | Crude protein | 20.95 |
| Cottonseed meal | 2.50 | Total calcium | 0.88 |
| Wheat flour | 4.00 | Total phosphorus | 0.59 |
| Hydrolyzed feather meal | 1.50 | Total lysine | 1.11 |
| Soybean oil | 2.00 | Total methionine | 0.33 |
| Dicalcium phosphate | 0.90 | Total threonine | 0.82 |
| Limestone | 1.50 |  |  |
| Bentonite | 1.00 |  |  |
| Premix^2^ | 2.00 |  |  |
| Total | 100.00 |  |  |

^1^All nutrient levels were analyzed values, except metabolizable energy.

^2^Supplied per kilogram of diet: vitamin A, 11,500 IU; cholecalciferol, 3,500 IU; vitamin E, 30 mg; vitamin K_3_, 5 mg; thiamin, 3.38 mg; riboflavin, 9.0 mg; pyridoxine, 8.96 mg; vitamin B_12_, 0.025 mg; choline chloride, 800 mg; calcium pantothenate, 13 mg; niacin, 45 mg; biotin, 0.15 mg; folic acid, 1.20 mg; Mn, 60 mg; Fe, 66.5 mg; Zn, 88 mg; Cu, 8.8 mg; I, 0.70 mg; Se, 0.288 mg.
