# Supplementary material for: Trans-Anethole Alleviates Subclinical Necro-Haemorrhagic Enteritis-Induced Intestinal Barrier Dysfunction and Intestinal Inflammation in Broilers
Source: Front Microbiol. 2022 Mar 21;13:831882. doi: 10.3389/fmicb.2022.831882 (PMC8977854; doi:10.3389/fmicb.2022.831882)
Supplement: Supplementary file 4 [file Table_2.docx]

**Supplementary Table 2**. Gene-specific primers sequences for quantitative real-time PCR

| Gene name^1^ | GenBank^2^ | Primer sequence^3^ (5'→3') | Length |
| --- | --- | --- | --- |
| *OCLN* | NM_205128.1 | ATGCACCCACTGAGTGTTGG | 93 |
|  |  | GAGGTGTGGGCCTTACACAG |  |
| *ZO-1* | XM_015278981.2 | AGCCCCTTGGTAATGTGTGG | 87 |
|  |  | TTGGGCGTGACGTATAGCTG |  |
| *CLDN1* | NM_001013611.2 | GGTATGGCAACAGAGTGGCT | 91 |
|  |  | CAGCCAATGAAGAGGGCTGA |  |
| *IL-1β* | NM_204524.1 | TGCCTGCAGAAGAAGCCTCG | 137 |
|  |  | CTCCGCAGCAGTTTGGTCAT |  |
| *IL-2* | AF000631.1 | ACTCTGCAGTGTTACCTGGG | 140 |
|  |  | CCGGTGTGATTTAGACCCGT |  |
| *IL-4* | NM_001007079.1 | TTGTTTGGGAGAGCCAGCAC | 102 |
|  |  | GACATGGTGCCTTGAGGGAG |  |
| *IL-8* | DQ393272.2 | CCTCCTCCTGGTTTCAGCTG | 136 |
|  |  | TGGCGTCAGCTTCACATCTT |  |
| *IL-10* | NM_012854.2 | CAGACCAGCACCAGTCATCA | 96 |
|  |  | TCCCGTTCTCATCCATCTTCTC |  |
| *TNF-α* | HQ739087.1 | GAACCCTCCGCAGTACTCAG | 116 |
|  |  | AACTCATCTGAACTGGGCGG |  |
| *IFN-γ* | NM_205149.1 | TGTAGCTGACGGTGGACCTA | 134 |
|  |  | GCGGCTTTGACTTGTCAGTG |  |
| *iNOS* | D85422.1 | ACTGAAGGTGGCTATTGGGC | 108 |
|  |  | TAGCAGTTGTTGGGTGGGTG |  |
| *NF-κB* | NM_001012887.2 | AAGATCTGGTGGTGTGCCTG | 137 |
|  |  | AGTGGAACCTTTCGCGGATT |  |
| *IκBα* | NM_001001472.2 | CAGCACTACACTTGGCCGTA | 101 |
|  |  | GGAGTAGCCCTGGTAGGTCA |  |
| *Bcl-2* | NM_205339.2 | TCGTCGCCTTCTTCGAGTTC | 156 |
|  |  | CAAAGGCATCCCATCCTCCG |  |
| *Bax* | NM_001025304.1 | GTGATGGCATGGGACATAGCTC | 148 |
|  |  | TGGCGTAGACCTTGCGGATAA |  |
| *Caspase-*3 | NM_204725.1 | TGGTGGAGGTGGAGGAGC | 183 |
|  |  | TGTCTGTCATCATGGCTCTTG |  |
| *β-Actin* | NM_205518.1 | ACCGGACTGTTACCAACACC | 116 |
|  |  | CCTGAGTCAAGCGCCAAAAG |  |

^1^*OCLN*, Occludin; *ZO-1*, Zonula occludens-1; *CLDN-1*, Claudin-1; *IL*, Interleukin; *TNF-α*, Tumor necrosis factor; *IFN-γ*, Interferon-γ; *iNOS*, Inducible nitric oxide synthase; *NF-κB*, Nuclear factor kappa beta; *IκBα*, NF-kappa-B inhibitor alpha; *Bcl-2*, B-cell lymphoma/leukemia 2; *Bax*, Bcl2-Associated X.

^2^GenBank Accession Number.

^3^Shown as the forward primer then the reverse primer.
