# Supplementary material for: Trans-Anethole Alleviates Subclinical Necro-Haemorrhagic Enteritis-Induced Intestinal Barrier Dysfunction and Intestinal Inflammation in Broilers
Source: Front Microbiol. 2022 Mar 21;13:831882. doi: 10.3389/fmicb.2022.831882 (PMC8977854; doi:10.3389/fmicb.2022.831882)
Supplement: Supplementary file 5 [file Table_3.docx]

**Supplementary Table 3.** Effects of TA on relative abundance of phylum in cecal microbiota of broilers challenged with subclinical NE^1^

| **Item** | **CON** | **NE** | **NE+TA400** | **NE+TA600** | ***P*-value** |
| --- | --- | --- | --- | --- | --- |
| *Firmicutes* | 75.81±8.59^ab^ | 79.05±6.90^a^ | 63.19±3.34^b^ | 64.47±8.37^b^ | 0.044 |
| *Bacteroidetes* | 18.89±2.83 | 16.20±1.01 | 31.42±3.60 | 23.44±2.11 | 0.234 |
| *Proteobacteria* | 2.79±0.31 | 2.87±0.26 | 2.59±0.39 | 2.93±0.89 | 0.982 |
| *Cyanobacteria* | 0.44±0.14 | 0.50±0.15 | 0.32±0.13 | 0.13±0.06 | 0.218 |
| *Tenericutes* | 0.44±0.13 | 0.65±0.04 | 0.29±0.21 | 0.32±0.17 | 0.148 |
| *Actinobacteria* | 0.13±0.05 | 0.09±0.07 | 0.09±0.04 | 0.11±0.08 | 0.596 |
| *Verrucomicrobia* | 0.13±0.06^a^ | 0.02±0.006^b^ | 0.03±0.004^ab^ | 0.09±0.008^ab^ | 0.018 |
| *Chloroflexi* | 0.001±0.003^b^ | 0.0002±0.0005^b^ | 0.0003±0.0007^b^ | 0.034±0.005^a^ | 0.010 |
| *Patescibacteria* | 0.002±0.002 | 0.005±0.004 | 0.008±0.001 | 0.014±0.008 | 0.302 |
| *Planctomycetes* | 0.0006±0.002^b^ | 0.00±0.00^b^ | 0.0004±0.001^b^ | 0.027±0.002^a^ | <0.001 |
| Other | 0.004±0.003^b^ | 0.002±0.003^b^ | 0.009±0.007^ab^ | 0.019±0.017^a^ | 0.005 |
| Unclassified | 0.31±0.06 | 0.29±0.016 | 0.29±0.09 | 0.26±0.12 | 0.882 |

^a-b^Means within a row with different letters differ significantly (*P* < 0.05)

^1^Data are means for 8 birds per treatment. The data in each group was expressed as mean with their standard errors (n=8)
