# Supplementary figures and images for: Trans-Anethole Alleviates Subclinical Necro-Haemorrhagic Enteritis-Induced Intestinal Barrier Dysfunction and Intestinal Inflammation in Broilers (part 2 of 5)
Source: Front Microbiol. 2022 Mar 21;13:831882. doi: 10.3389/fmicb.2022.831882 (PMC8977854; doi:10.3389/fmicb.2022.831882)

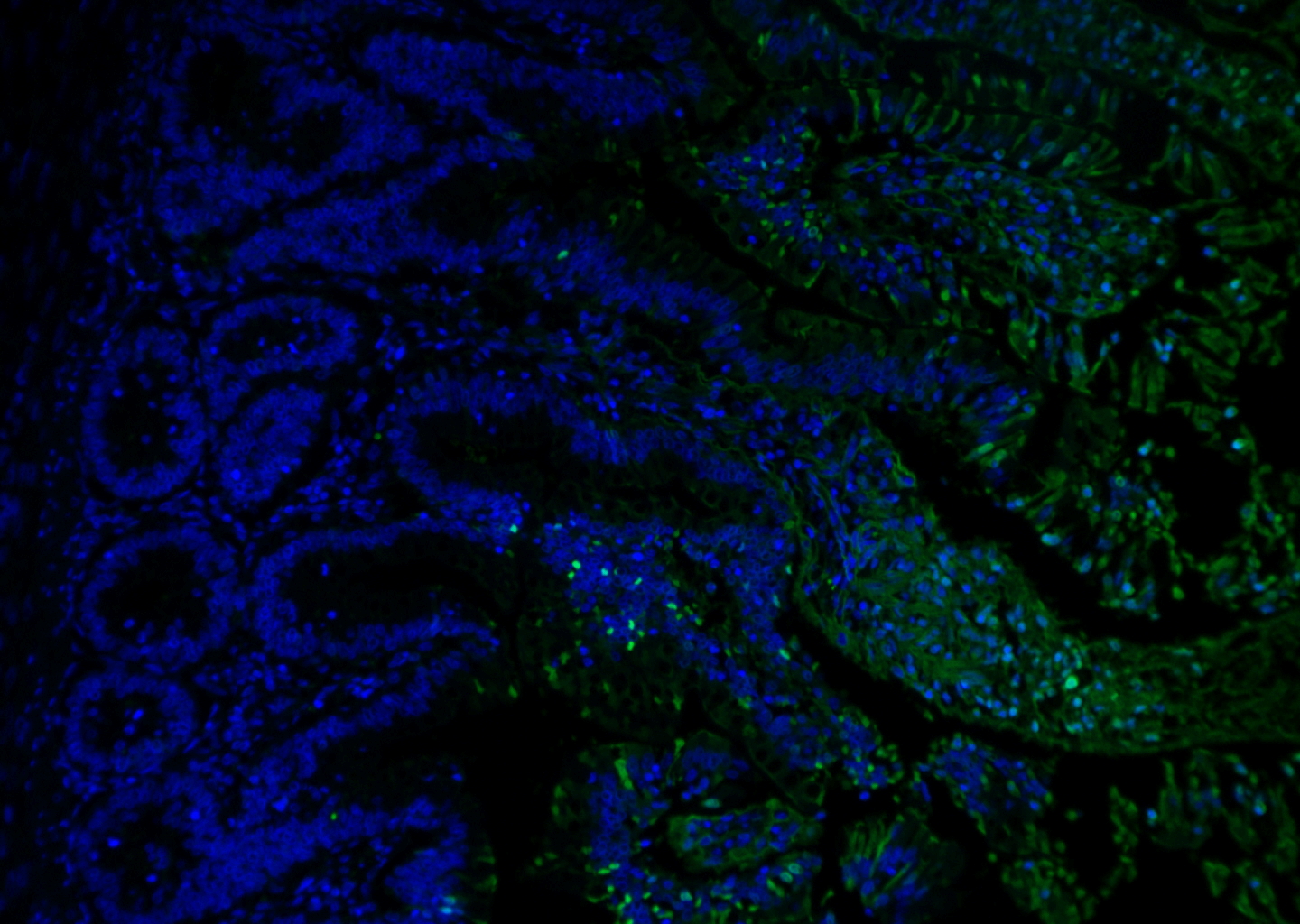

Supplement: Supplementary file 7 [file Data_Sheet_2.ZIP › CON group-Jejunal TUNEL apoptosis/200 x/CON-1 200-3 4.jpg]

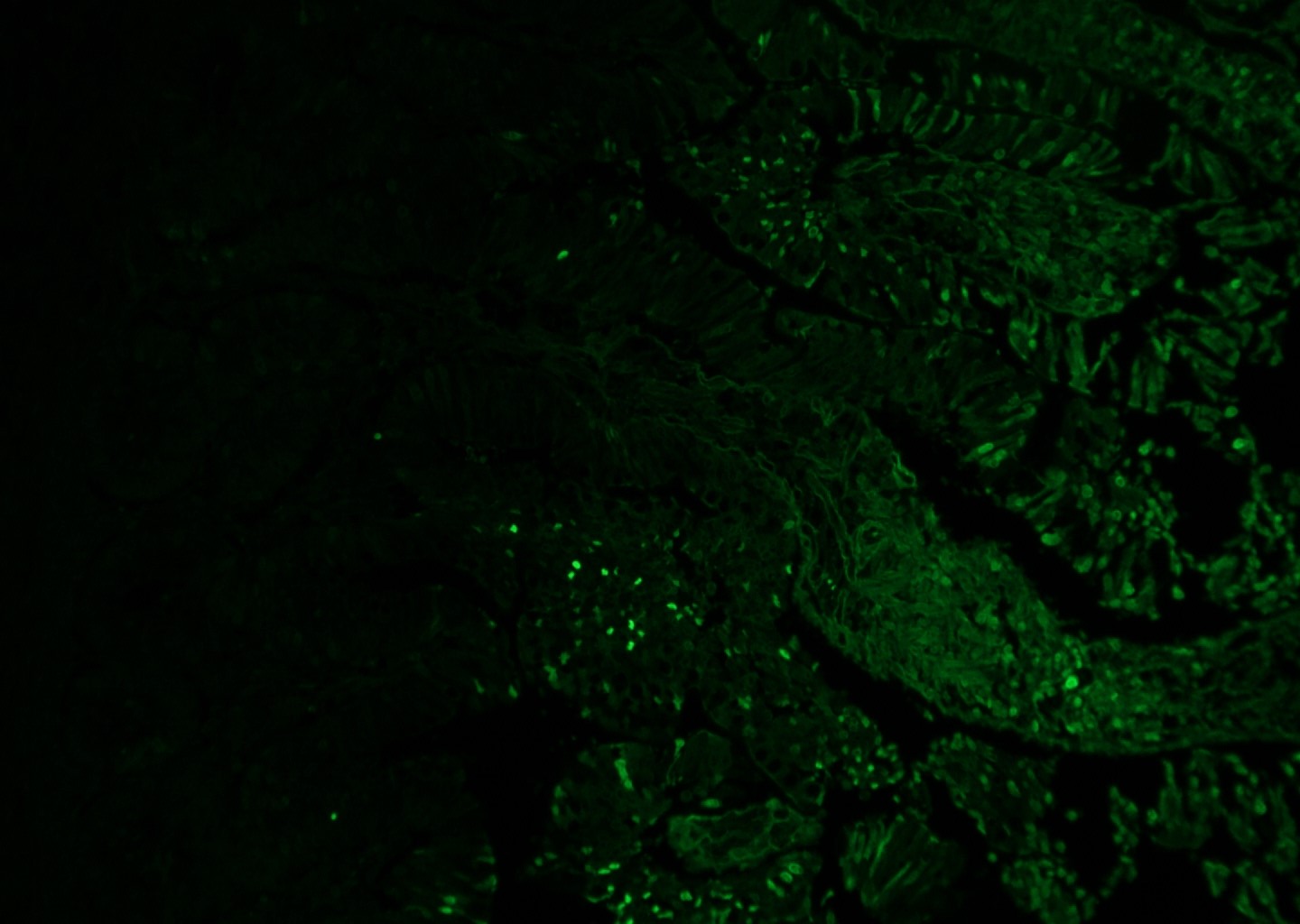

Supplement: Supplementary file 7 [file Data_Sheet_2.ZIP › CON group-Jejunal TUNEL apoptosis/200 x/CON-1 200-3.jpg]

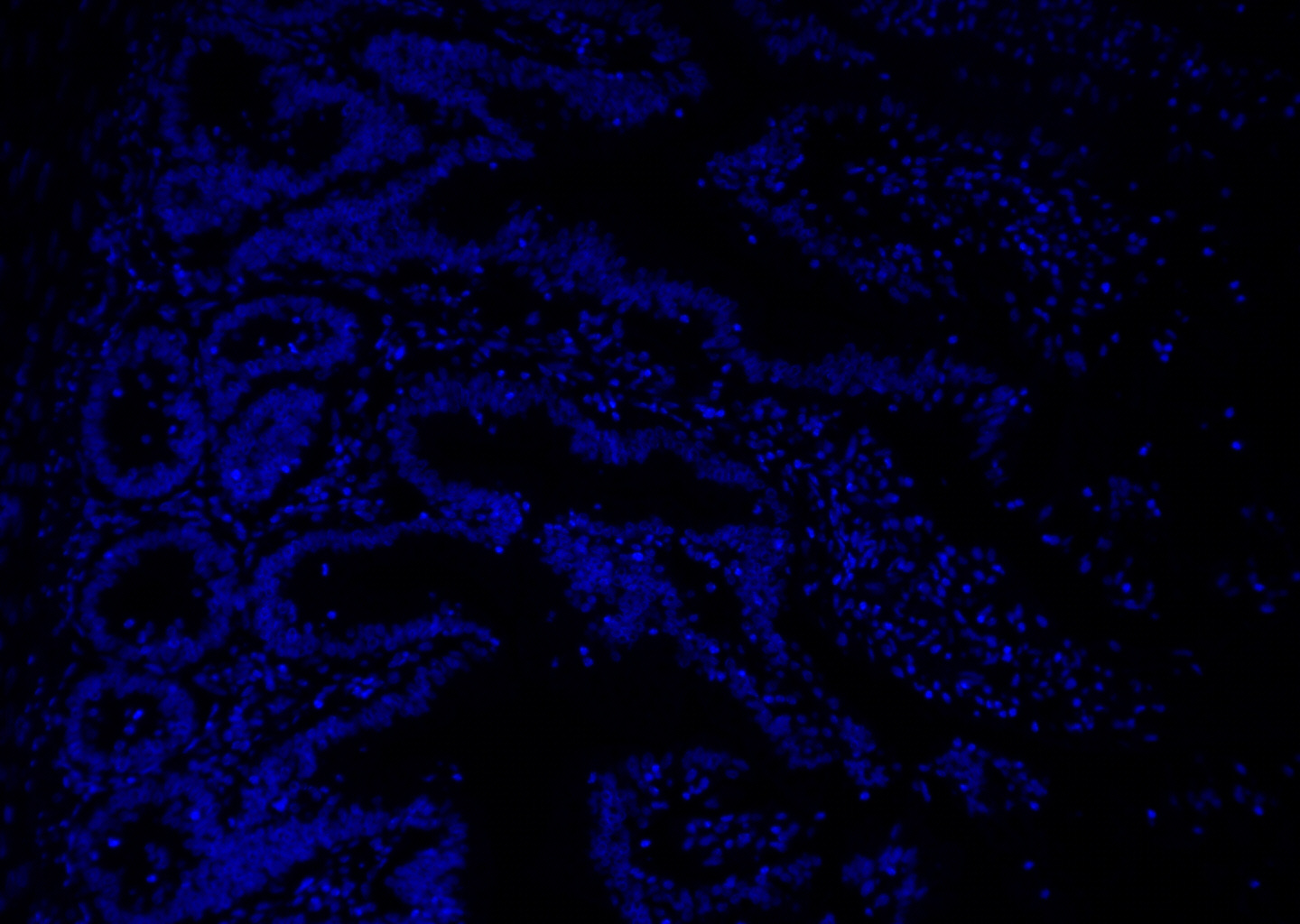

Supplement: Supplementary file 7 [file Data_Sheet_2.ZIP › CON group-Jejunal TUNEL apoptosis/200 x/CON-1 200-4.jpg]

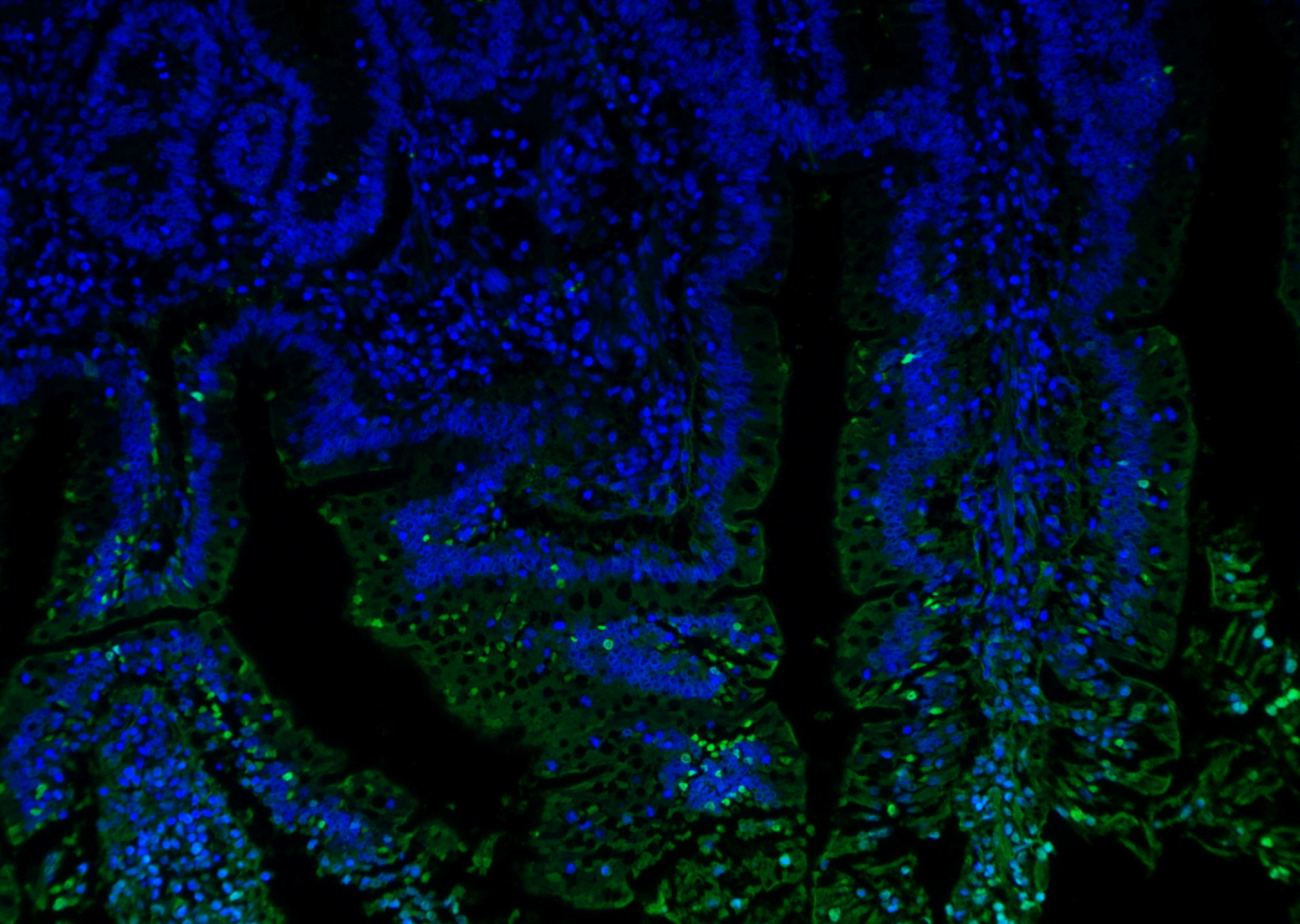

Supplement: Supplementary file 7 [file Data_Sheet_2.ZIP › CON group-Jejunal TUNEL apoptosis/200 x/CON-1 200-5 6.jpg]

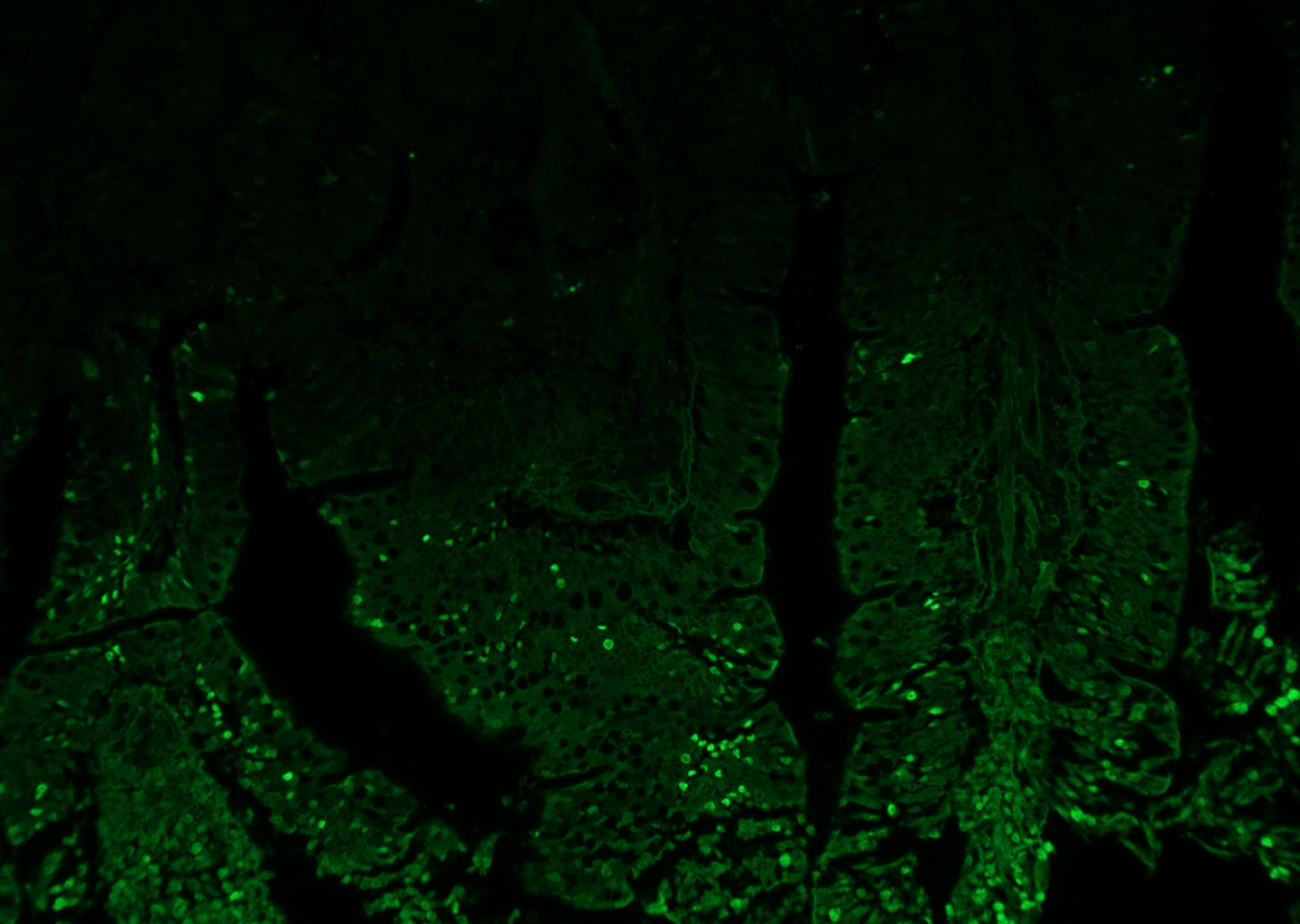

Supplement: Supplementary file 7 [file Data_Sheet_2.ZIP › CON group-Jejunal TUNEL apoptosis/200 x/CON-1 200-5.jpg]

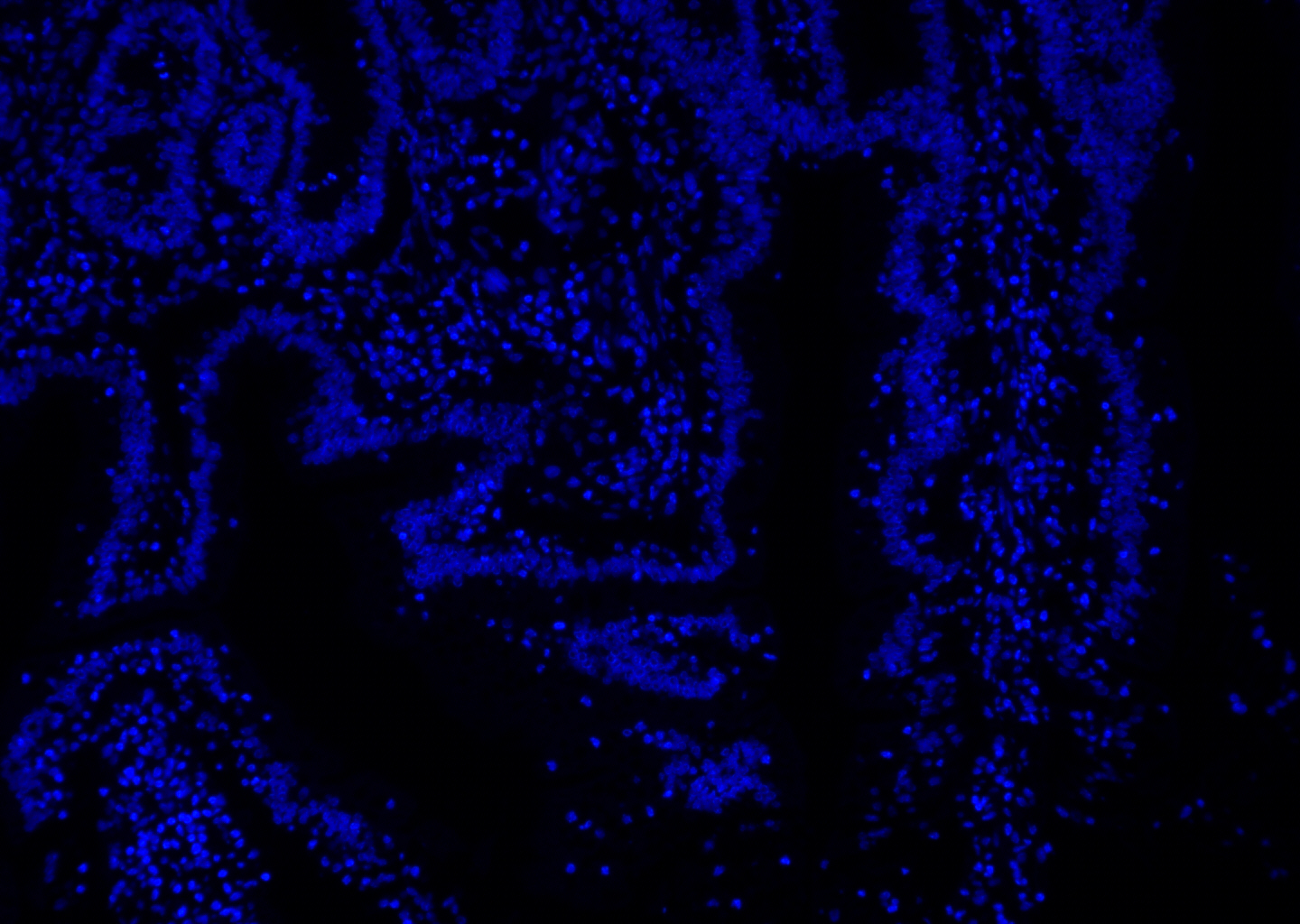

Supplement: Supplementary file 7 [file Data_Sheet_2.ZIP › CON group-Jejunal TUNEL apoptosis/200 x/CON-1 200-6.jpg]

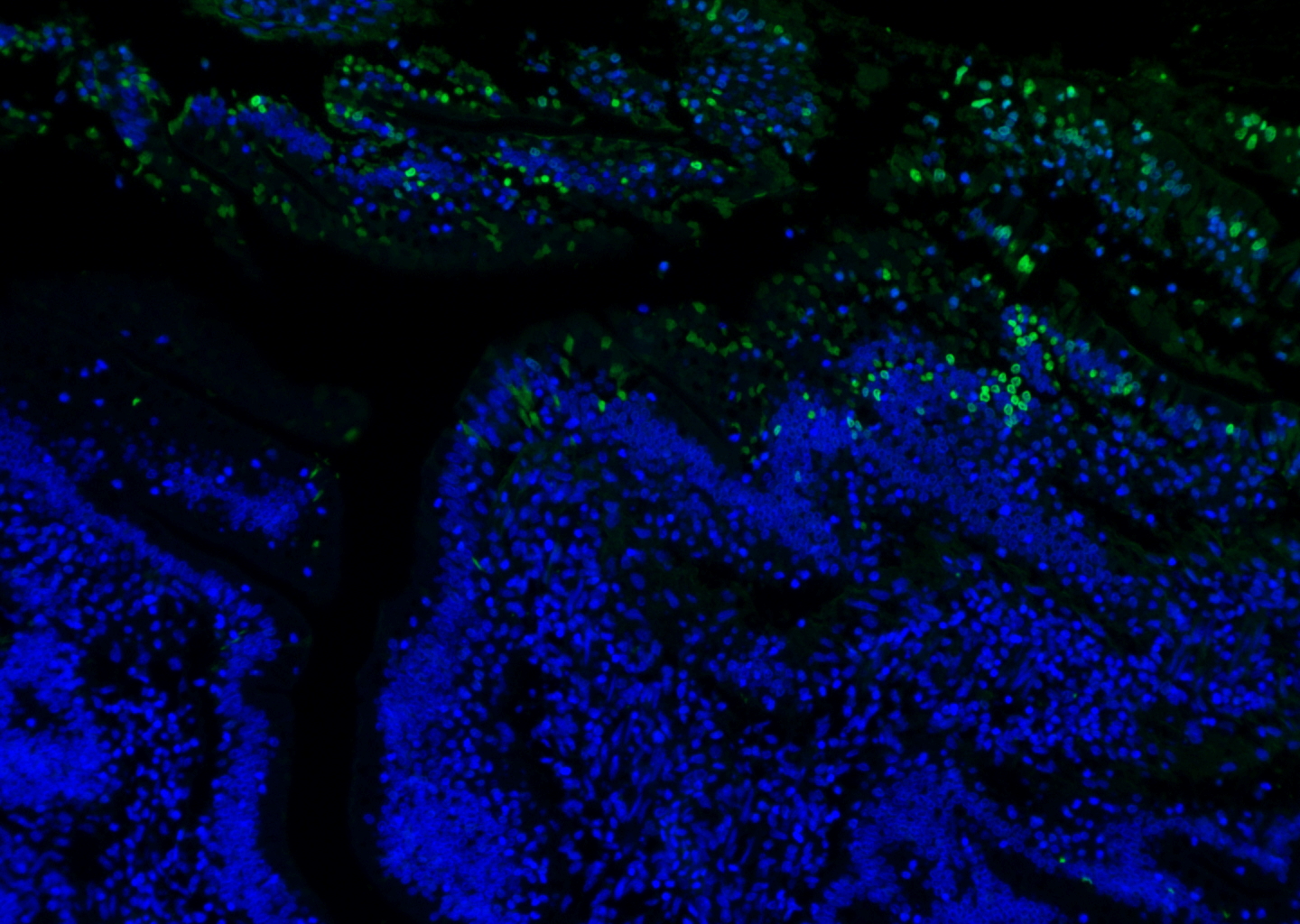

Supplement: Supplementary file 7 [file Data_Sheet_2.ZIP › CON group-Jejunal TUNEL apoptosis/200 x/CON-2 200-1 2.jpg]

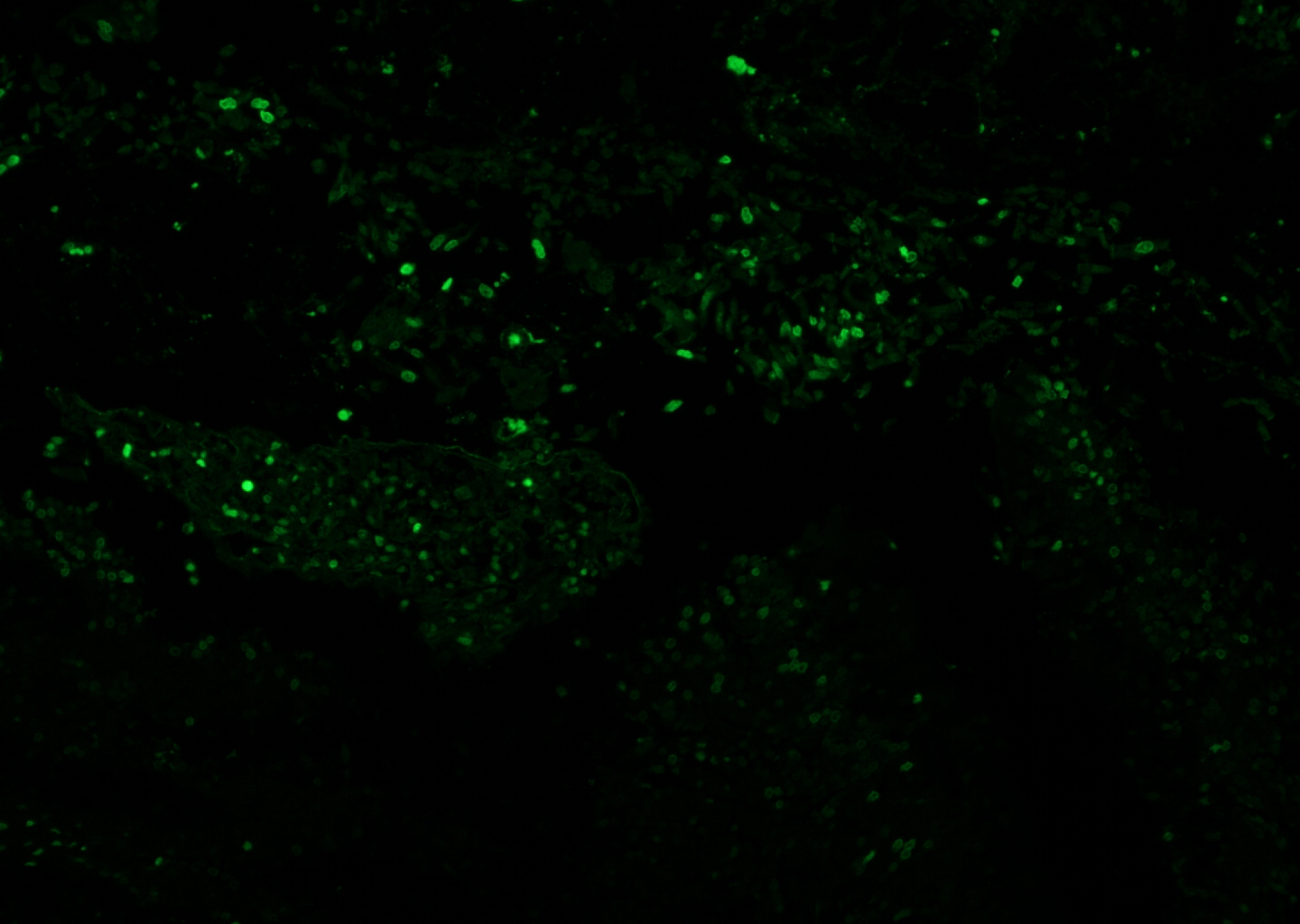

Supplement: Supplementary file 7 [file Data_Sheet_2.ZIP › CON group-Jejunal TUNEL apoptosis/200 x/CON-2 200-1.jpg]

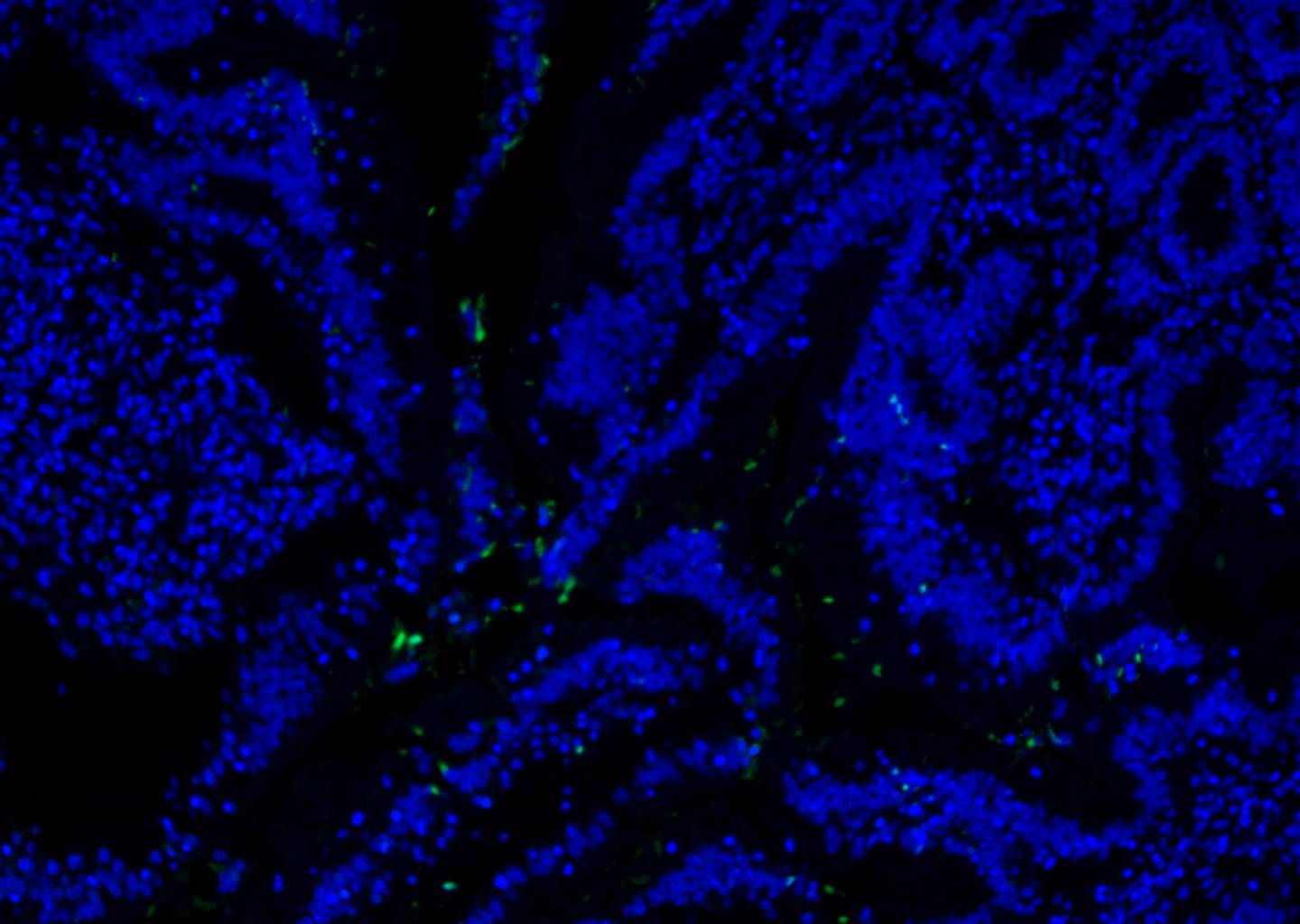

Supplement: Supplementary file 7 [file Data_Sheet_2.ZIP › CON group-Jejunal TUNEL apoptosis/200 x/CON-2 200-3 4.jpg]

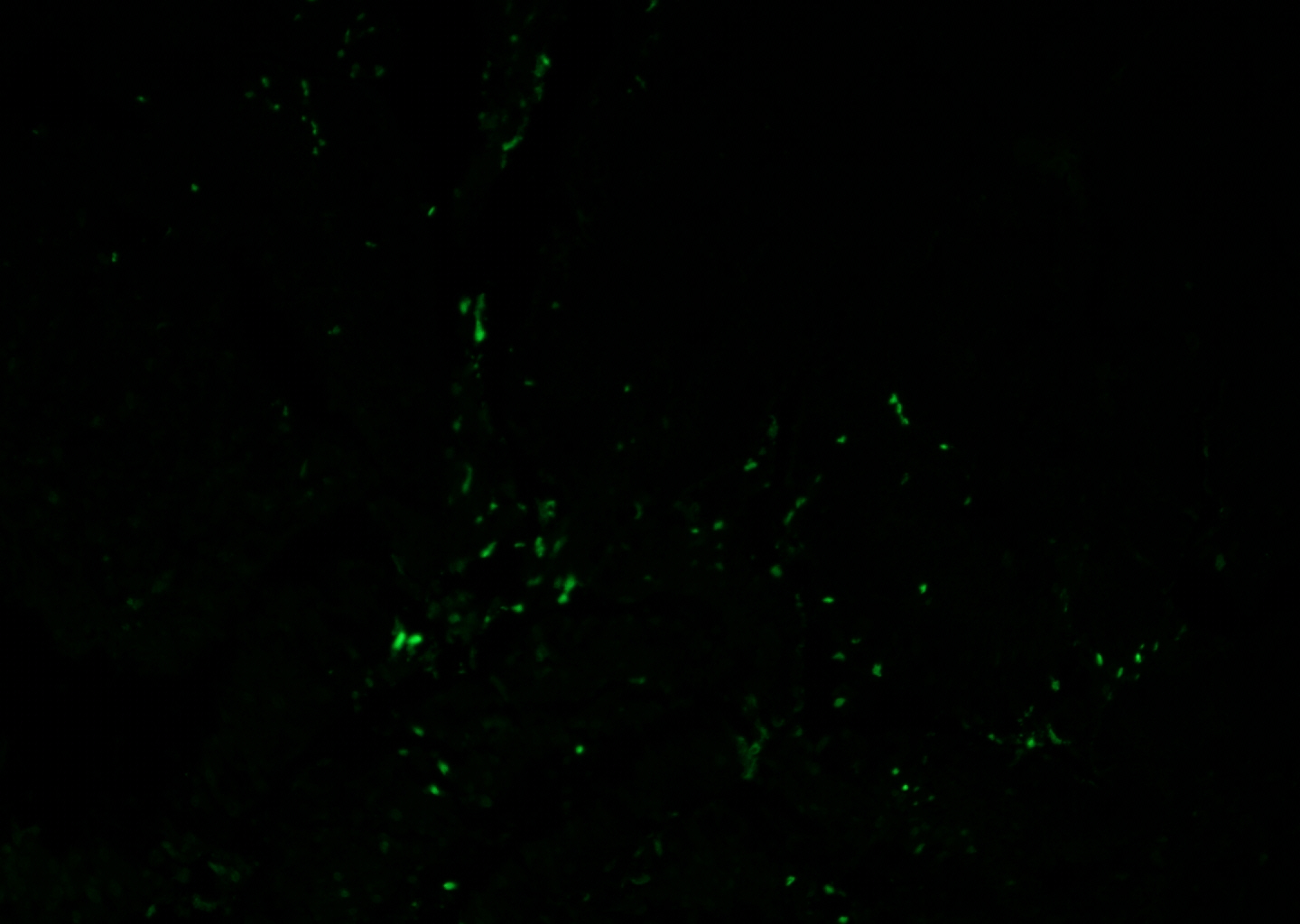

Supplement: Supplementary file 7 [file Data_Sheet_2.ZIP › CON group-Jejunal TUNEL apoptosis/200 x/CON-2 200-3.jpg]

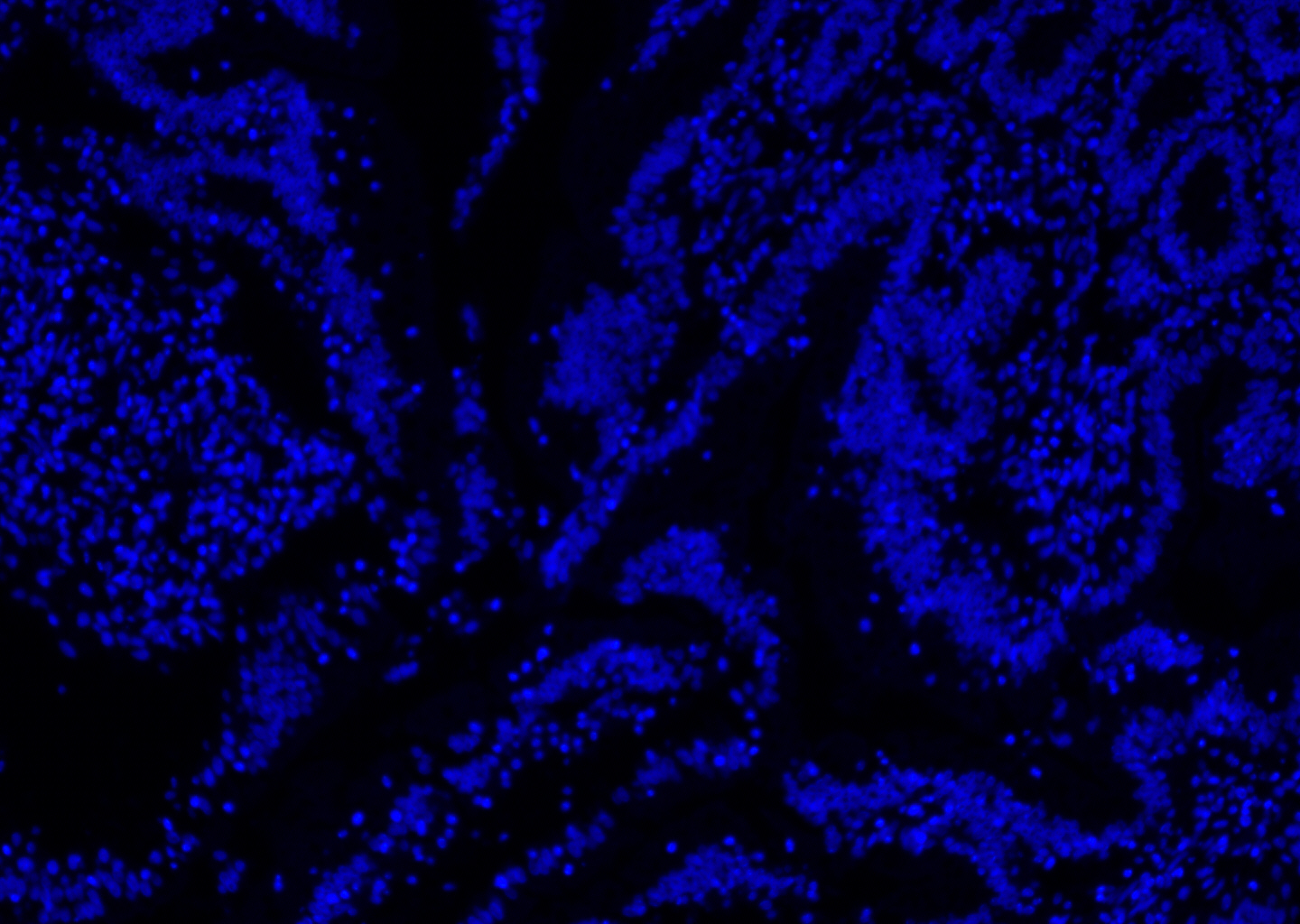

Supplement: Supplementary file 7 [file Data_Sheet_2.ZIP › CON group-Jejunal TUNEL apoptosis/200 x/CON-2 200-4.jpg]

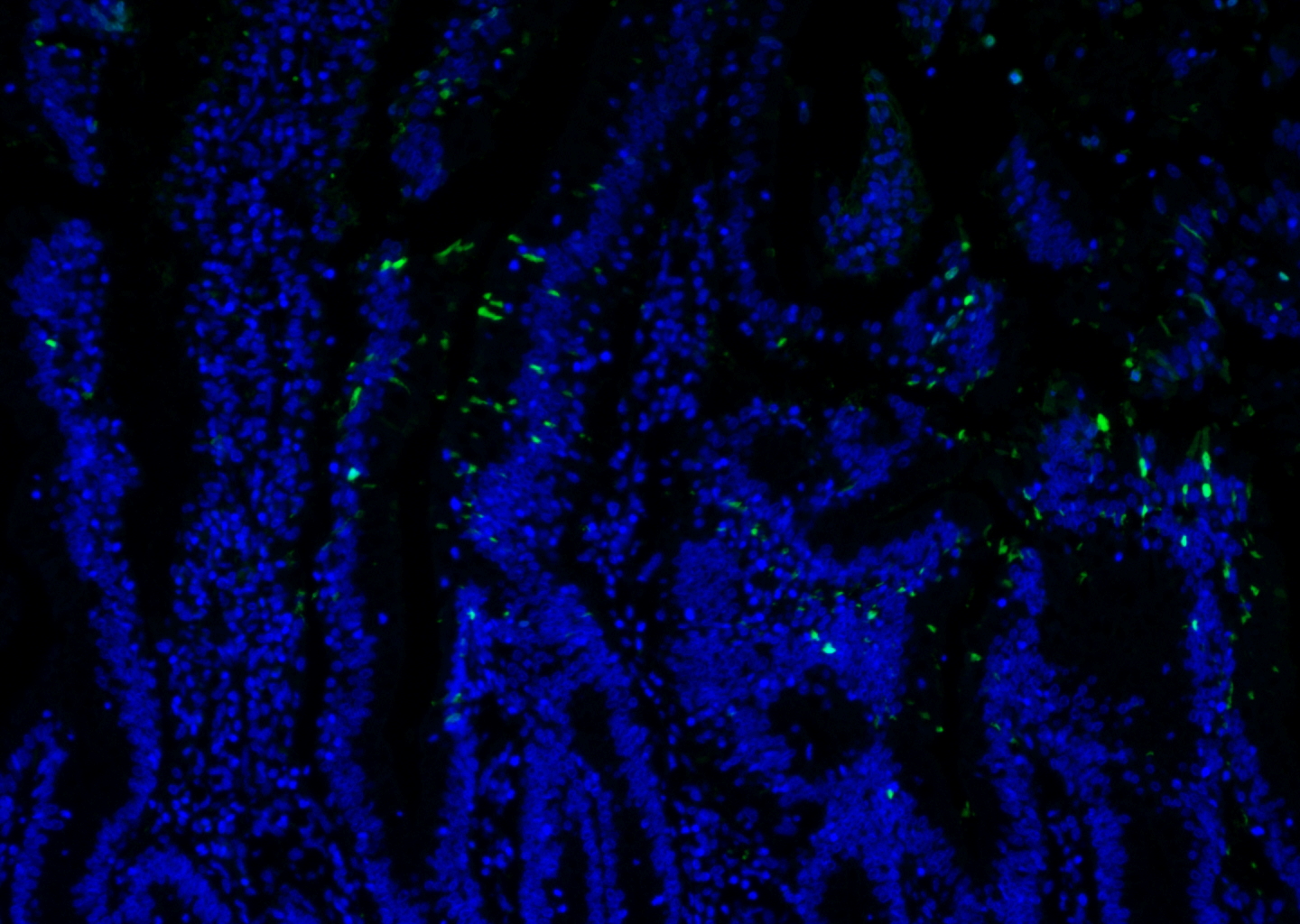

Supplement: Supplementary file 7 [file Data_Sheet_2.ZIP › CON group-Jejunal TUNEL apoptosis/200 x/CON-2 200-5 6.jpg]

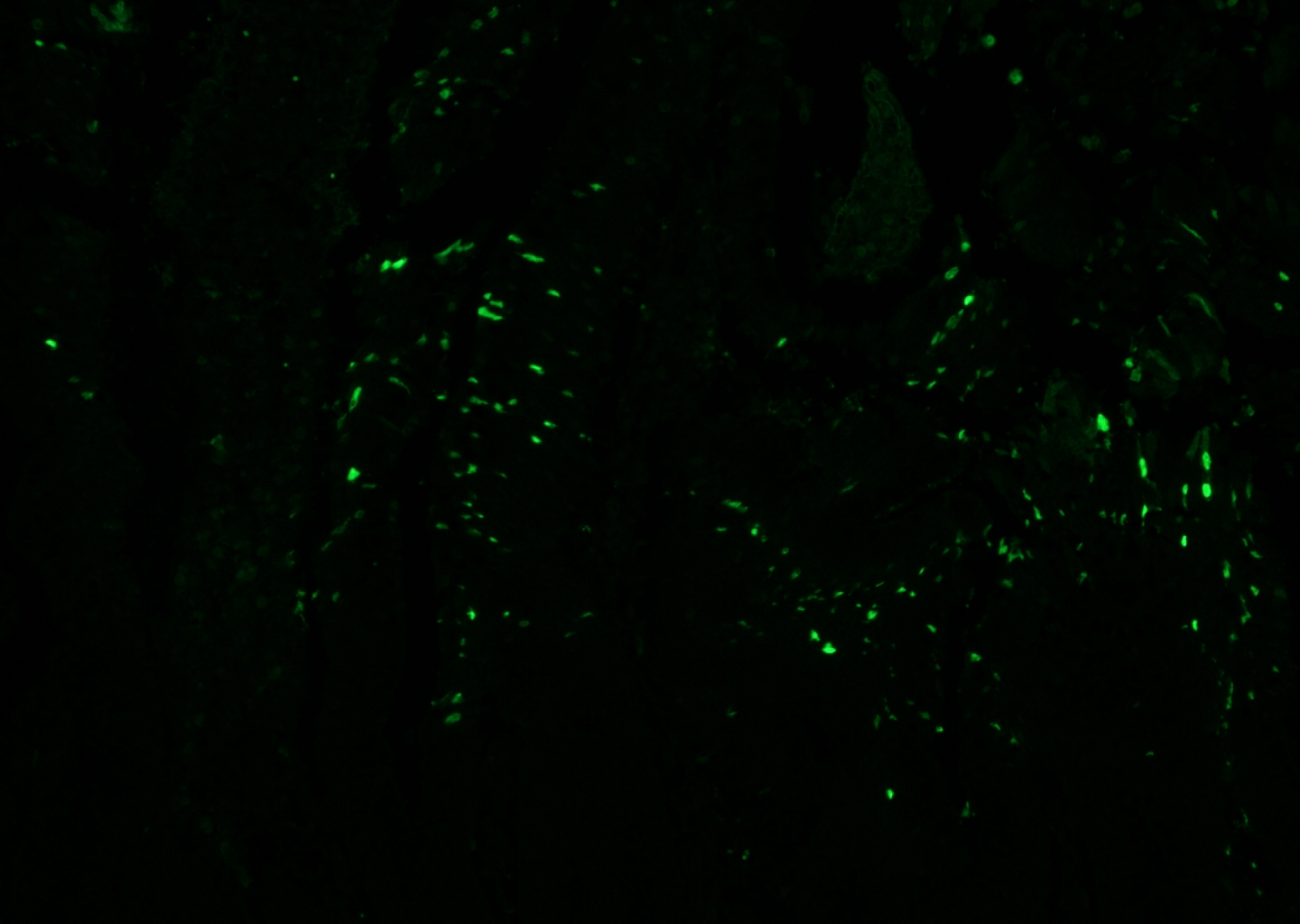

Supplement: Supplementary file 7 [file Data_Sheet_2.ZIP › CON group-Jejunal TUNEL apoptosis/200 x/CON-2 200-5.jpg]

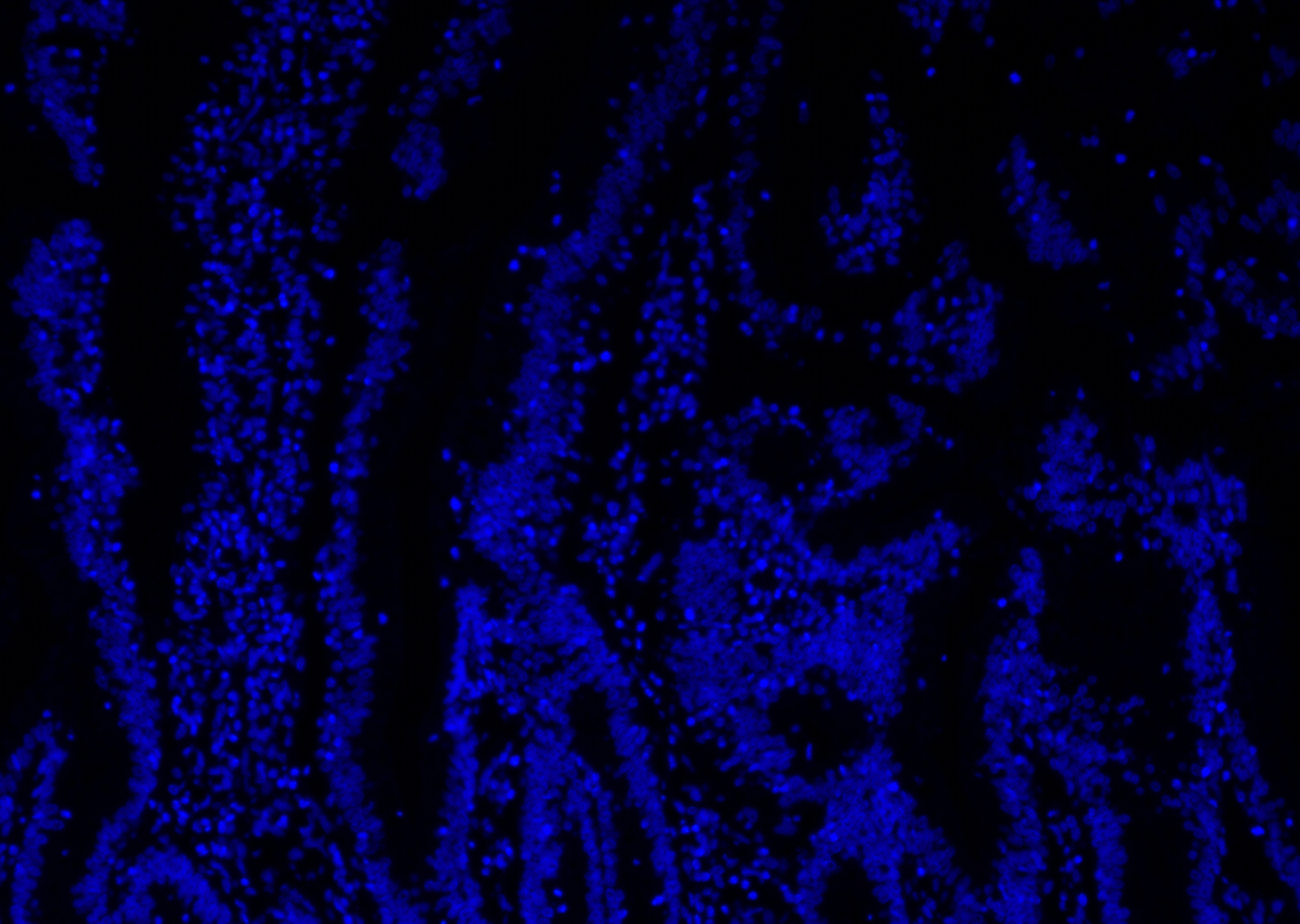

Supplement: Supplementary file 7 [file Data_Sheet_2.ZIP › CON group-Jejunal TUNEL apoptosis/200 x/CON-2 200-6.jpg]

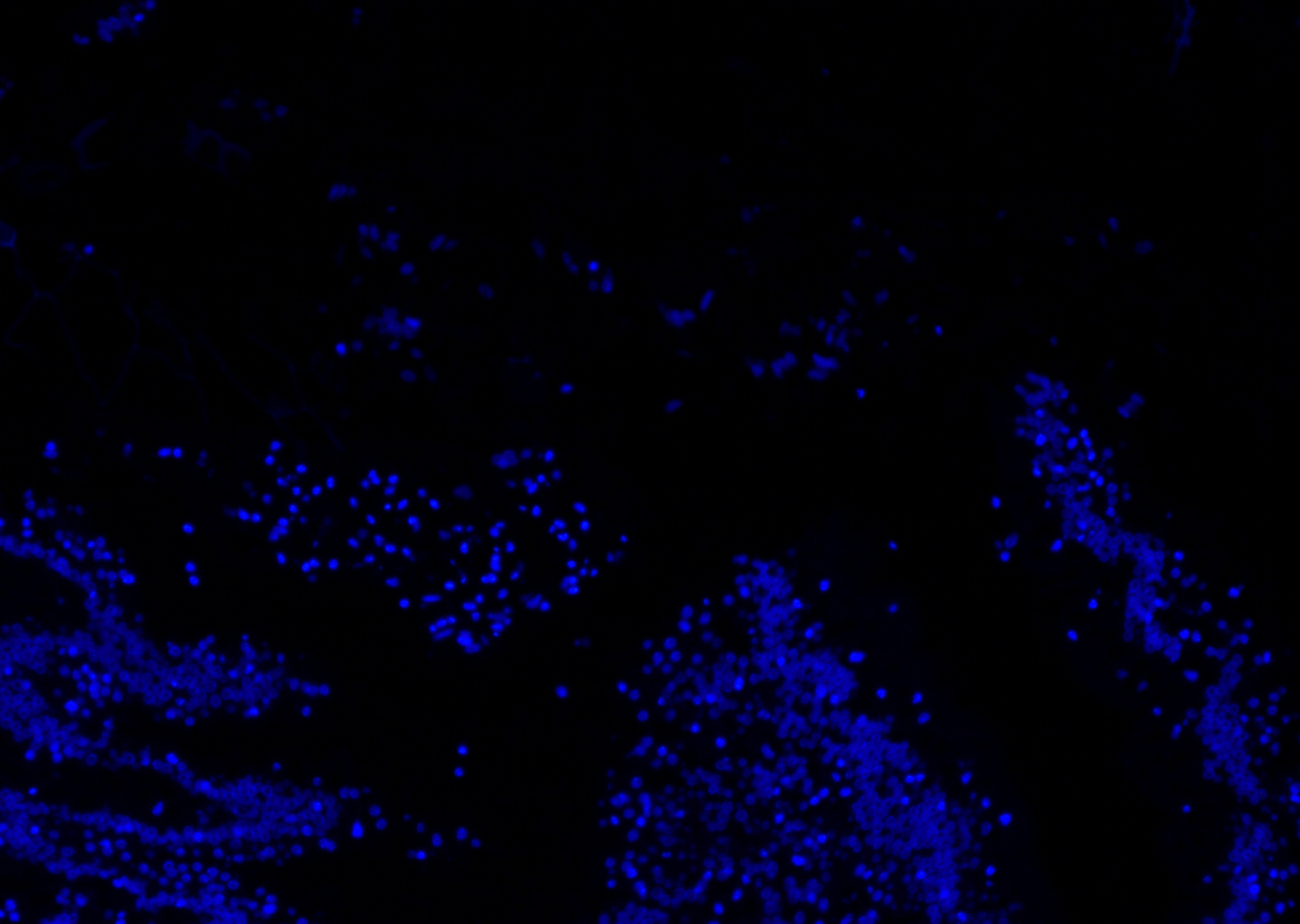

Supplement: Supplementary file 7 [file Data_Sheet_2.ZIP › CON group-Jejunal TUNEL apoptosis/200 x/CON-2 200-2.jpg]

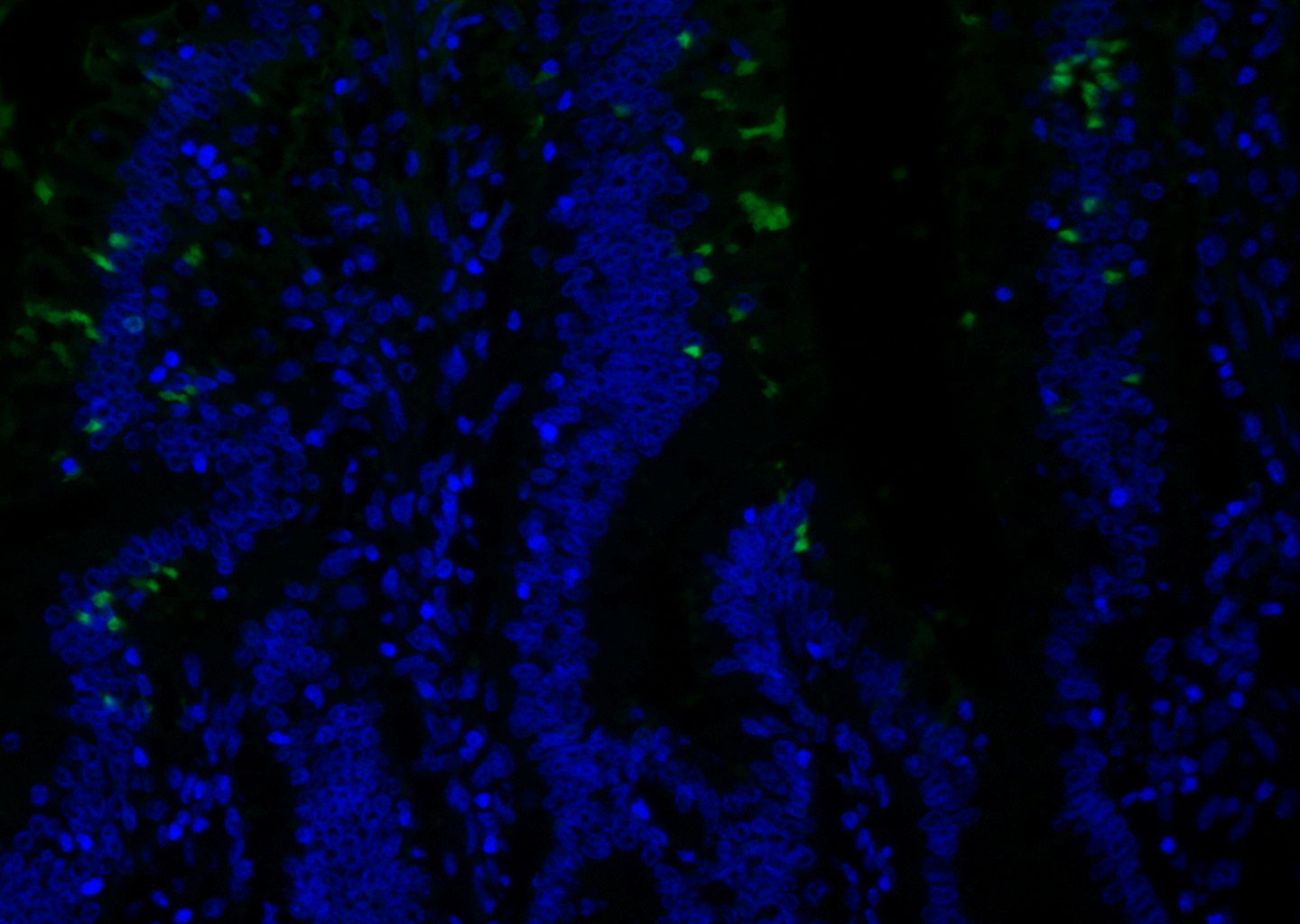

Supplement: Supplementary file 7 [file Data_Sheet_2.ZIP › CON group-Jejunal TUNEL apoptosis/400 x/CON-1 400-1 2.jpg]

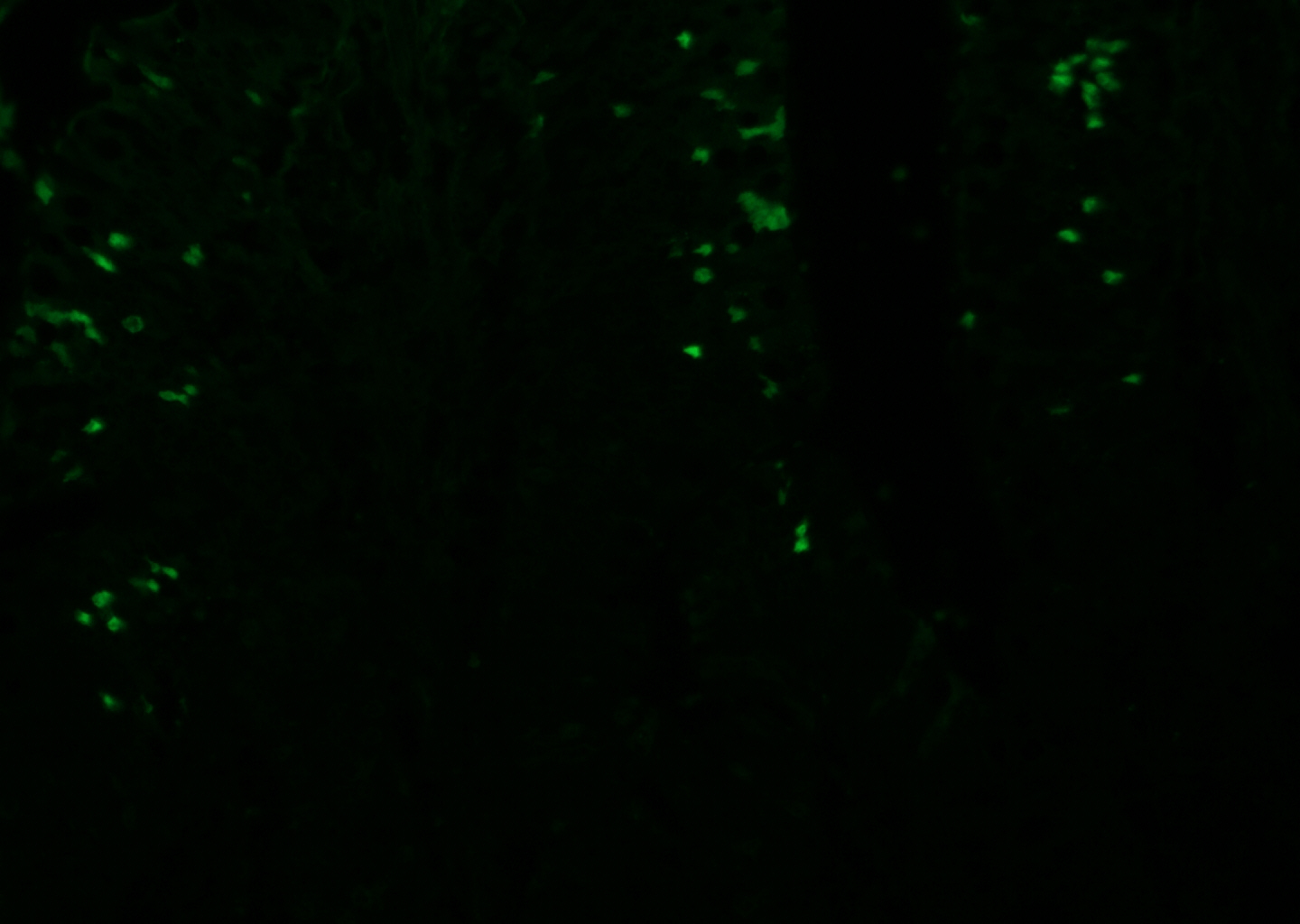

Supplement: Supplementary file 7 [file Data_Sheet_2.ZIP › CON group-Jejunal TUNEL apoptosis/400 x/CON-1 400-1.jpg]

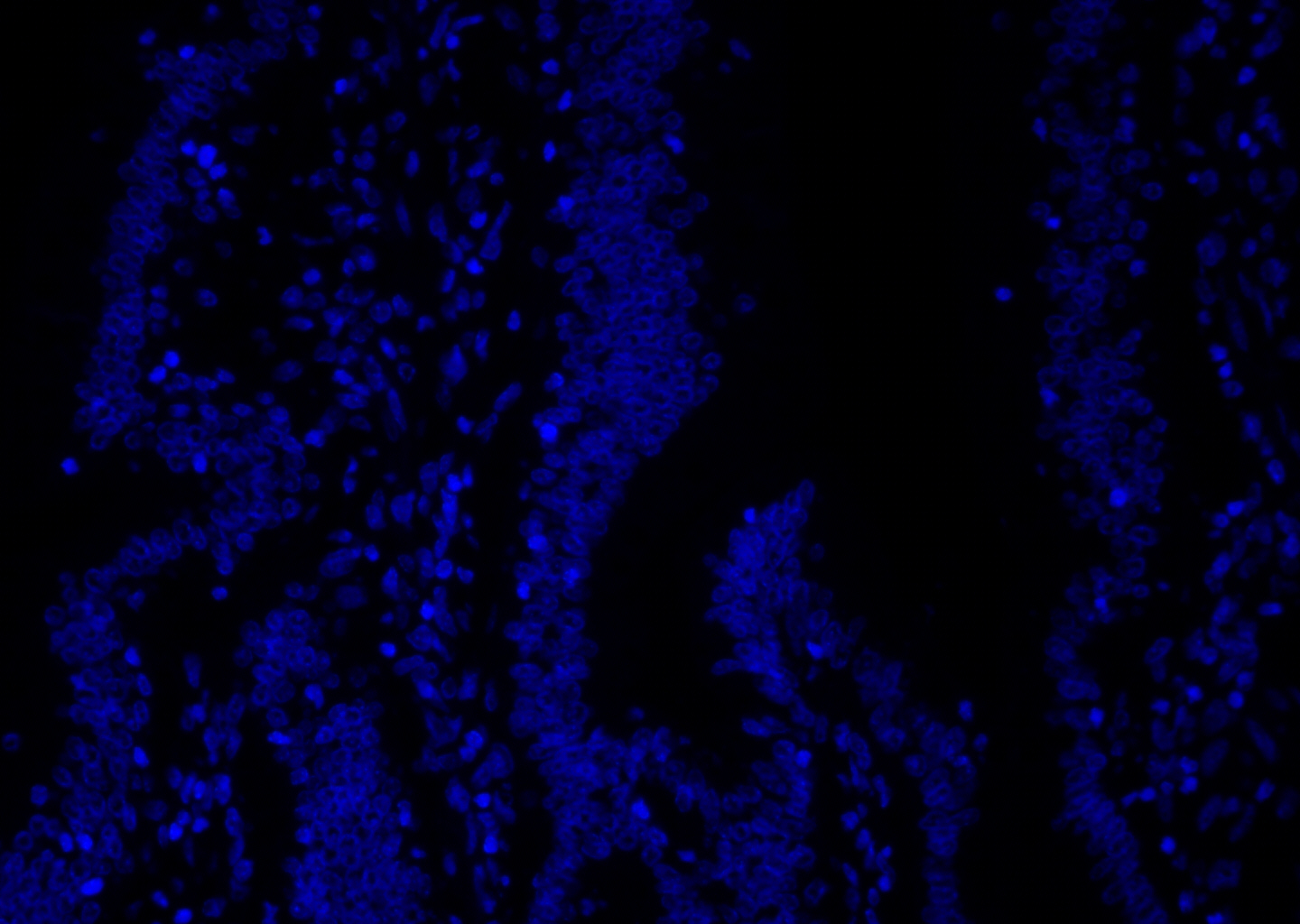

Supplement: Supplementary file 7 [file Data_Sheet_2.ZIP › CON group-Jejunal TUNEL apoptosis/400 x/CON-1 400-2.jpg]

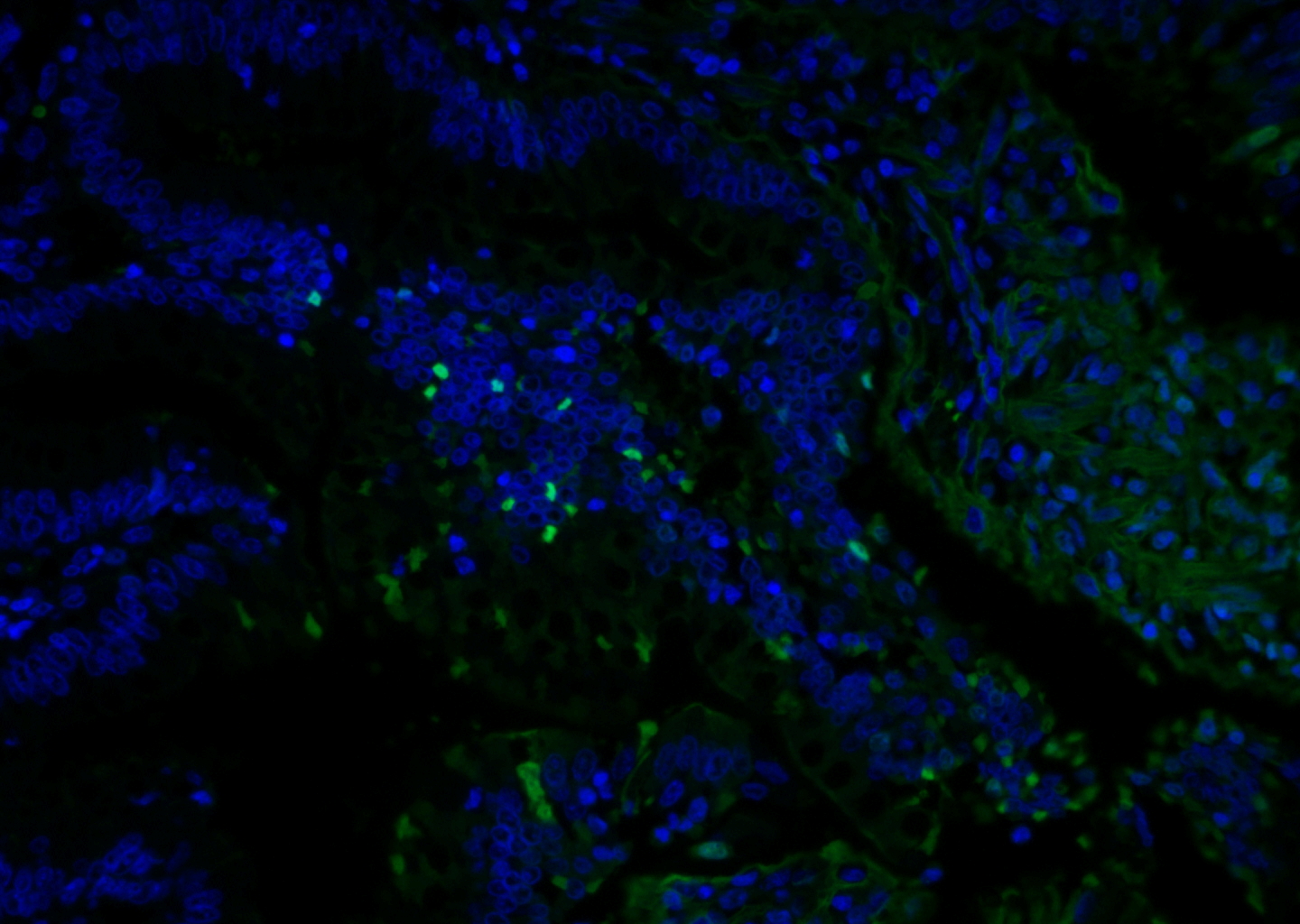

Supplement: Supplementary file 7 [file Data_Sheet_2.ZIP › CON group-Jejunal TUNEL apoptosis/400 x/CON-1 400-3 4.jpg]

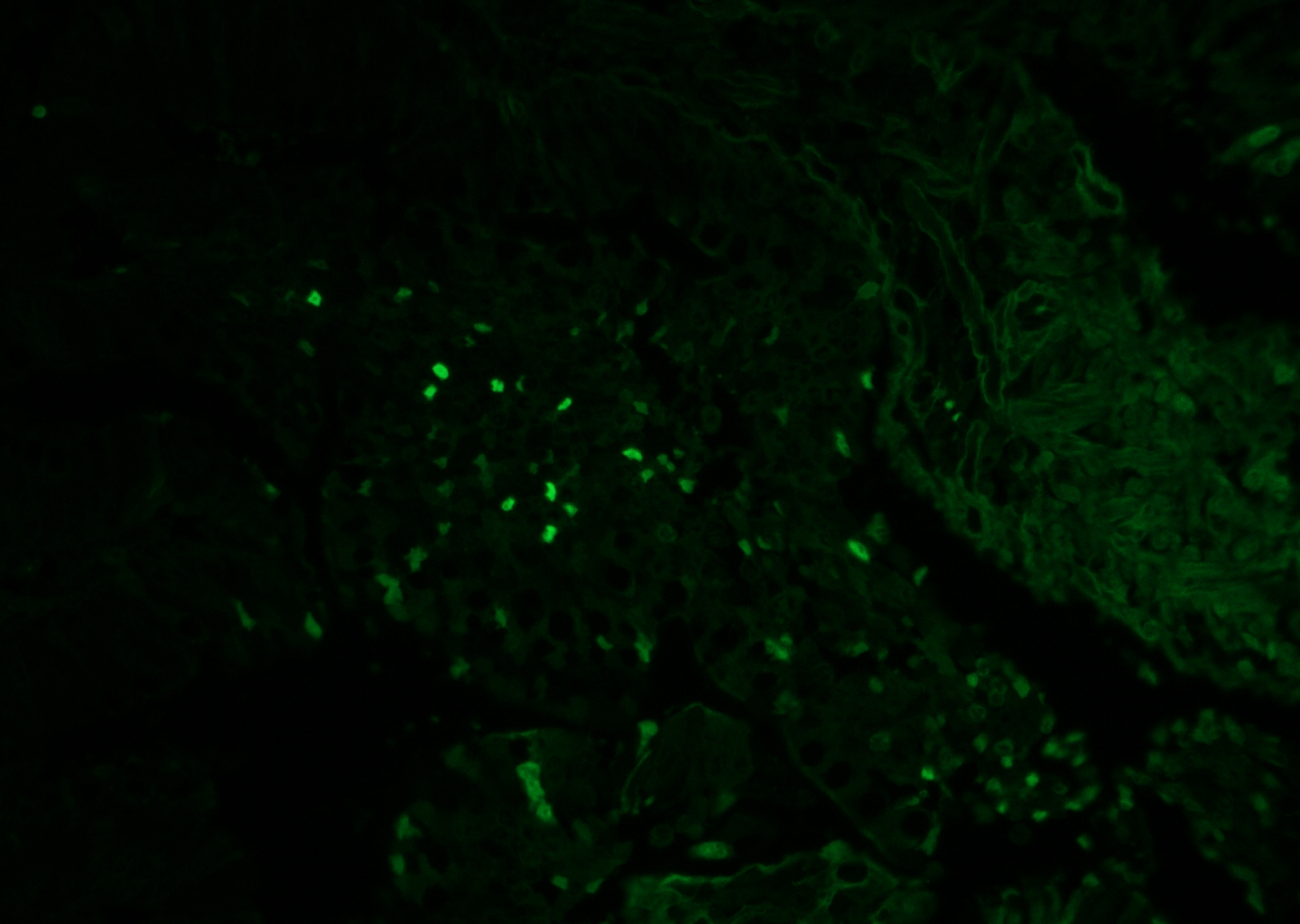

Supplement: Supplementary file 7 [file Data_Sheet_2.ZIP › CON group-Jejunal TUNEL apoptosis/400 x/CON-1 400-3.jpg]

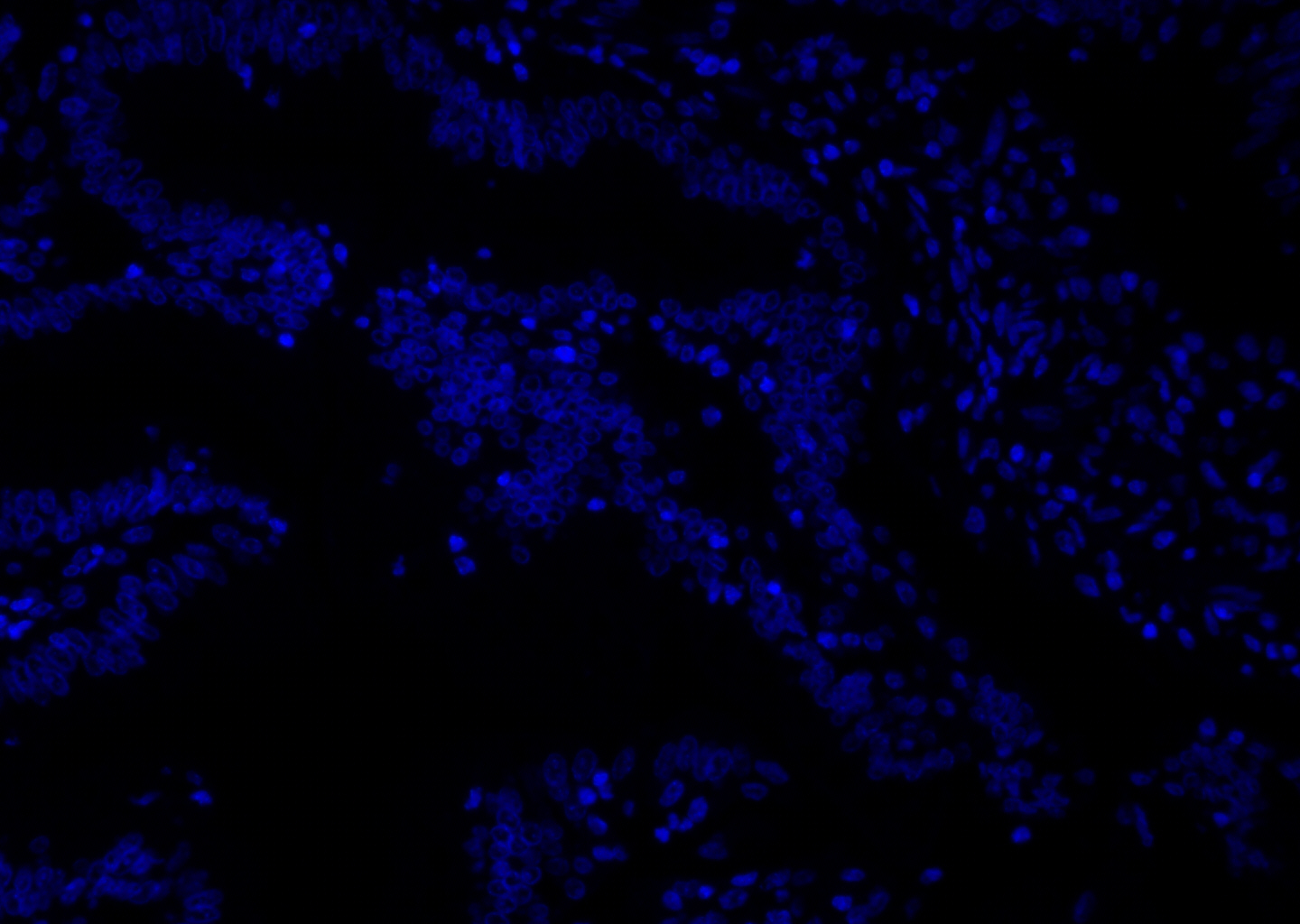

Supplement: Supplementary file 7 [file Data_Sheet_2.ZIP › CON group-Jejunal TUNEL apoptosis/400 x/CON-1 400-4.jpg]

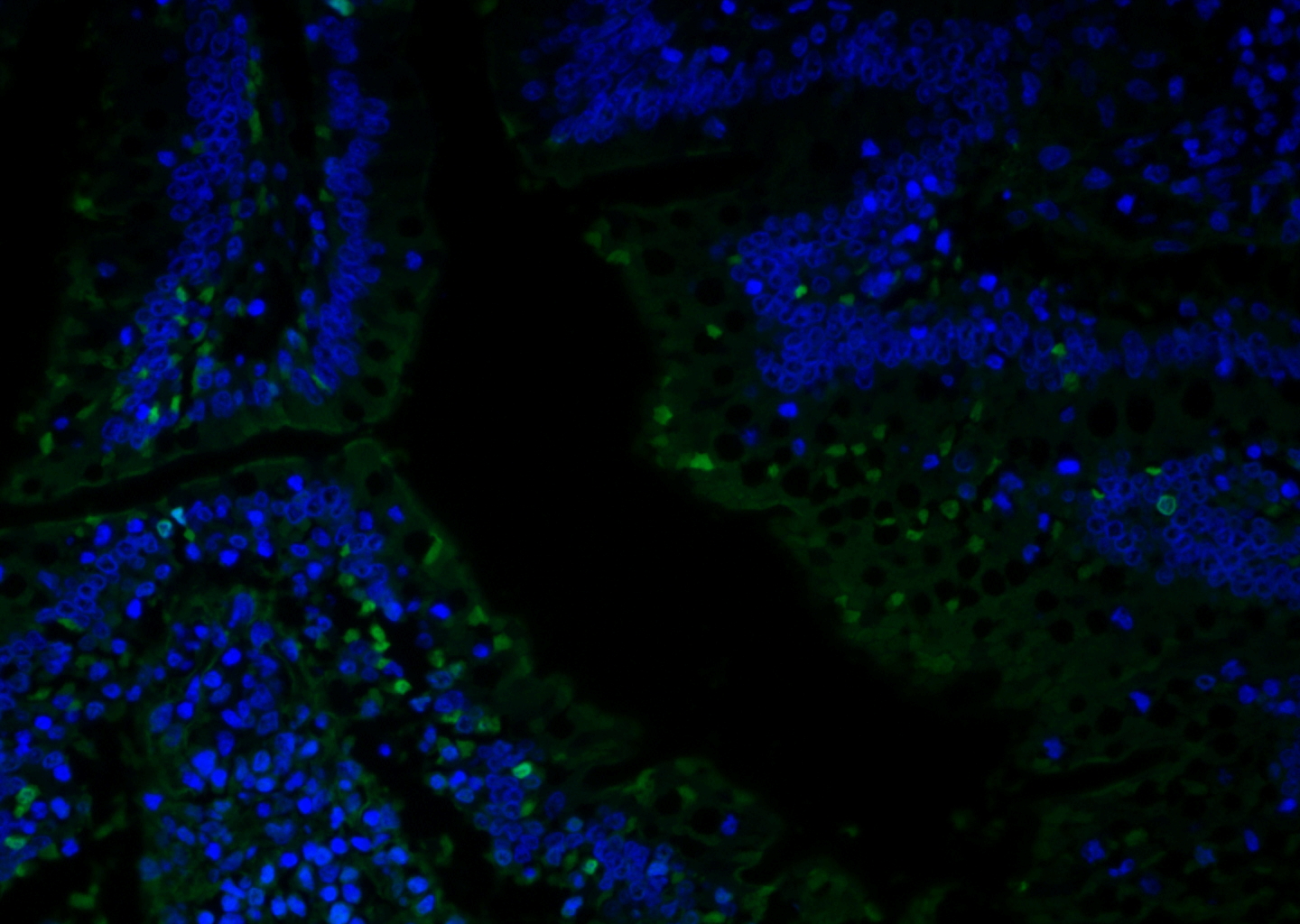

Supplement: Supplementary file 7 [file Data_Sheet_2.ZIP › CON group-Jejunal TUNEL apoptosis/400 x/CON-1 400-5 6.jpg]

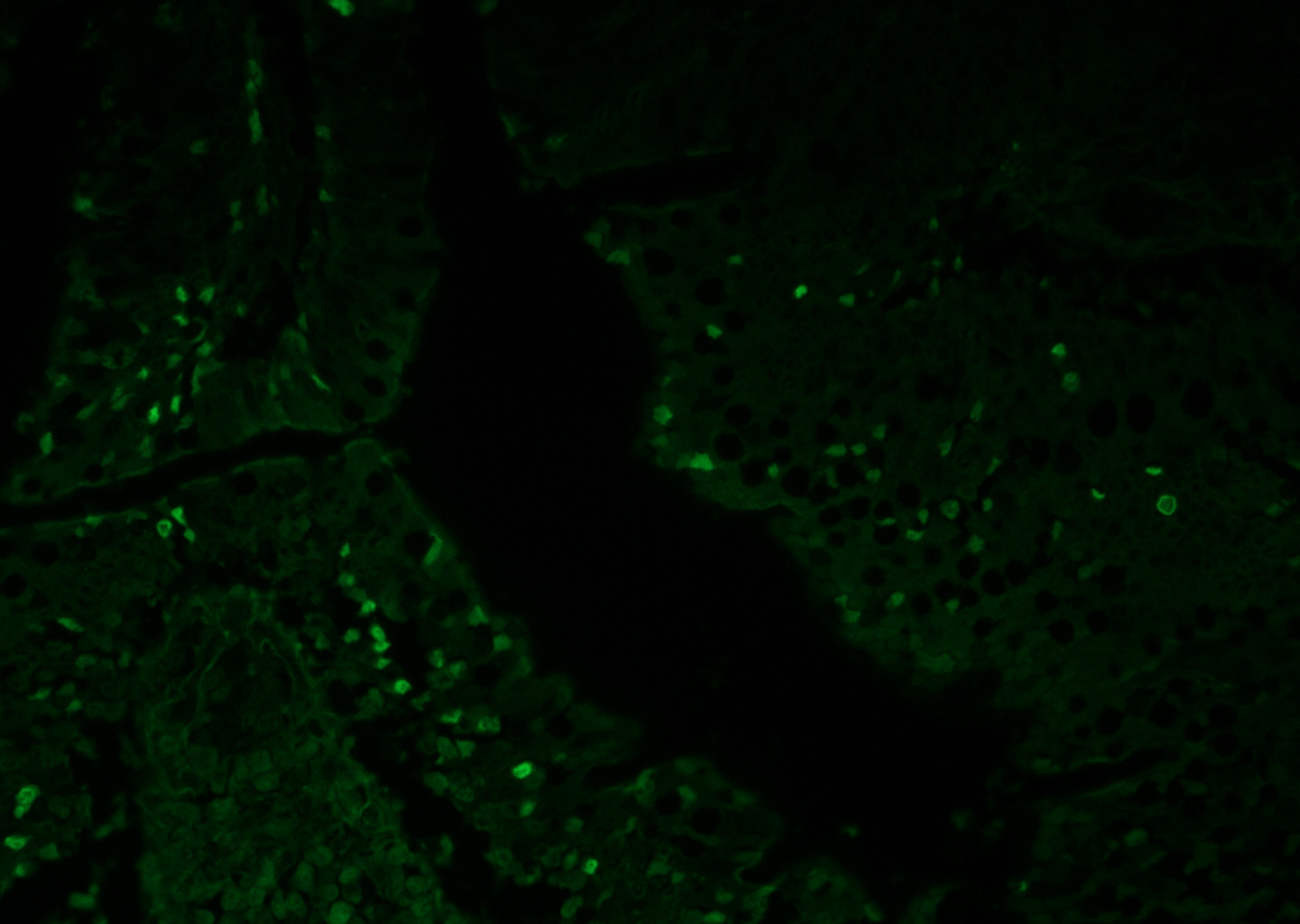

Supplement: Supplementary file 7 [file Data_Sheet_2.ZIP › CON group-Jejunal TUNEL apoptosis/400 x/CON-1 400-5.jpg]

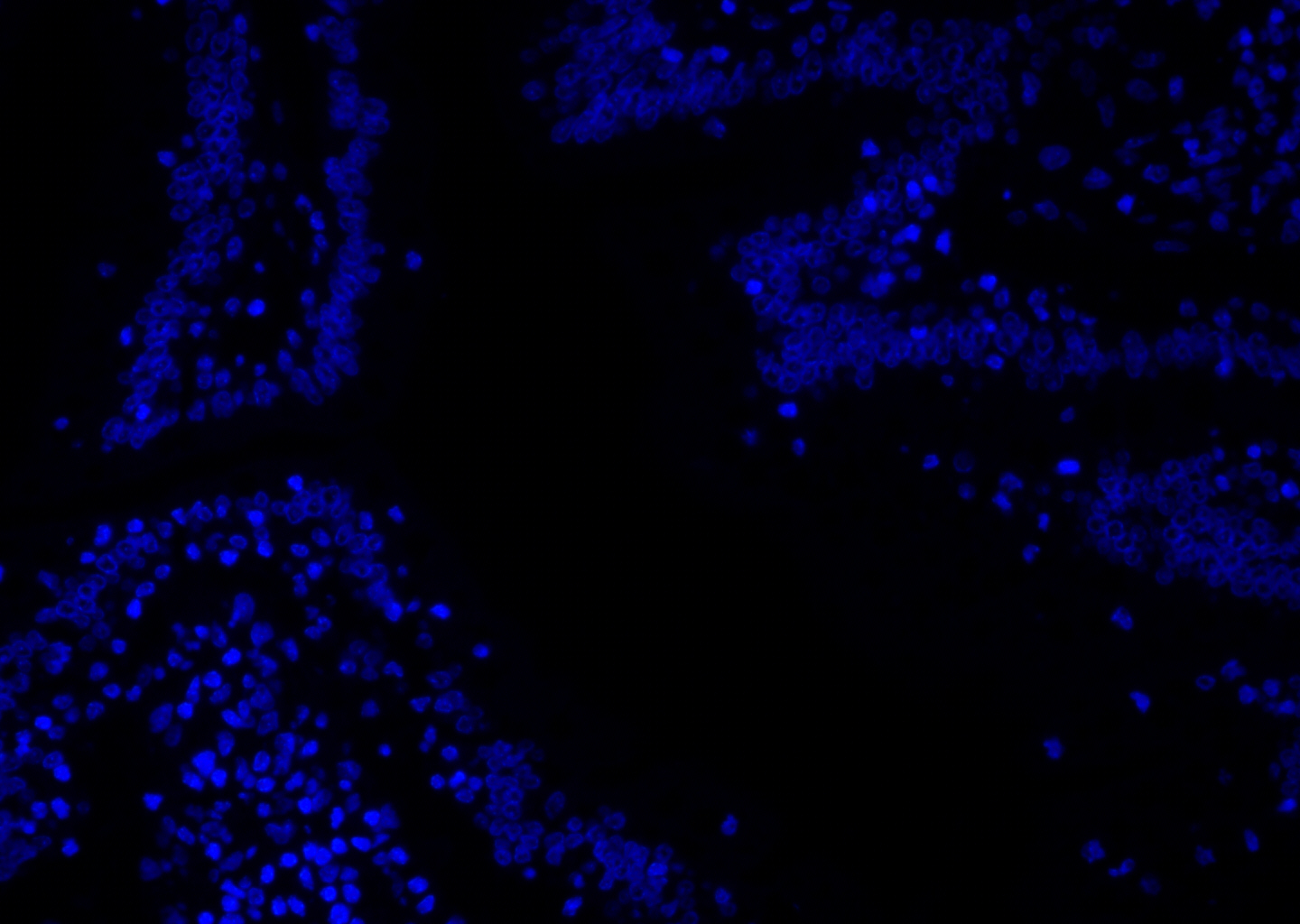

Supplement: Supplementary file 7 [file Data_Sheet_2.ZIP › CON group-Jejunal TUNEL apoptosis/400 x/CON-1 400-6.jpg]

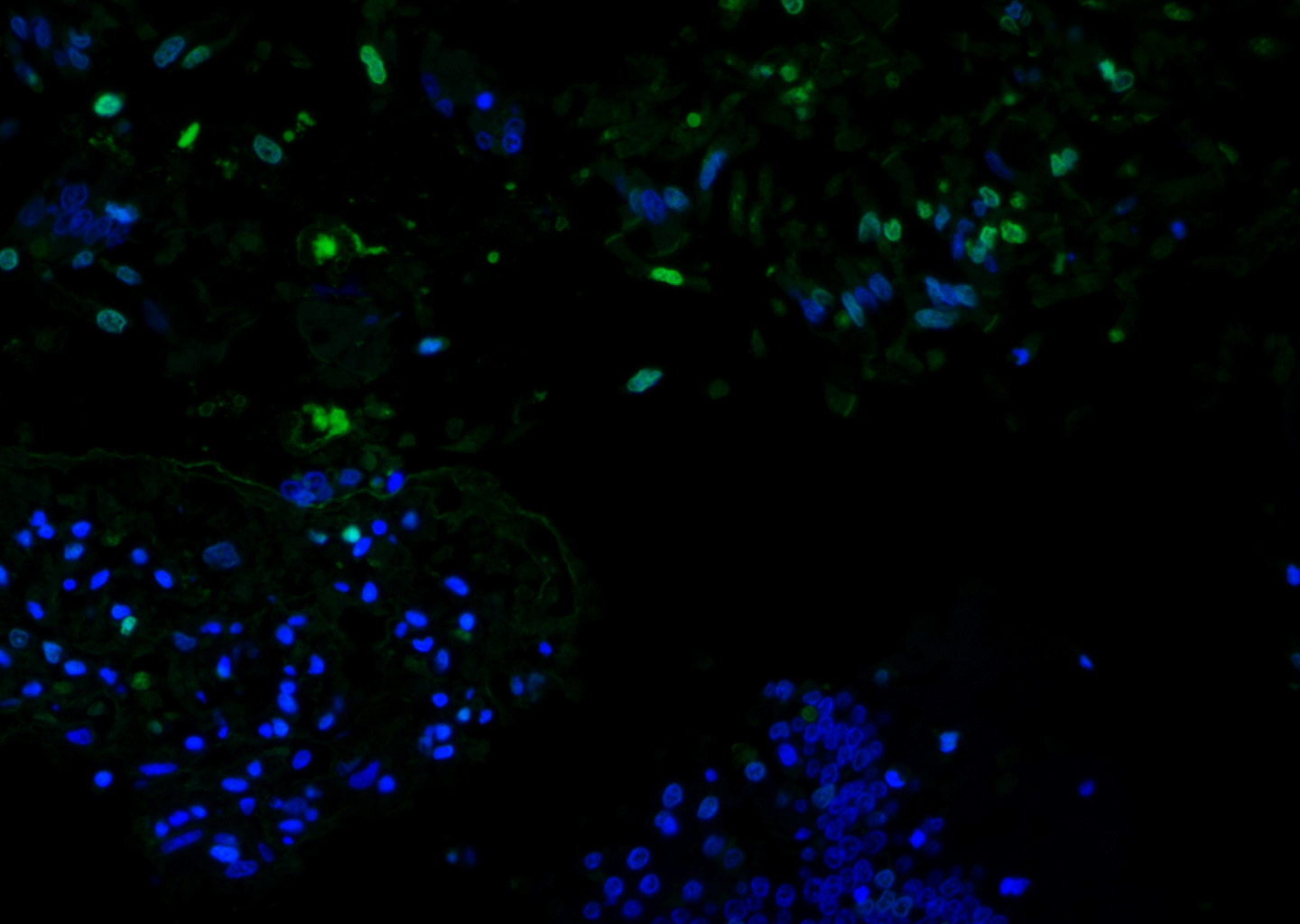

Supplement: Supplementary file 7 [file Data_Sheet_2.ZIP › CON group-Jejunal TUNEL apoptosis/400 x/CON-2 400-1 2.jpg]

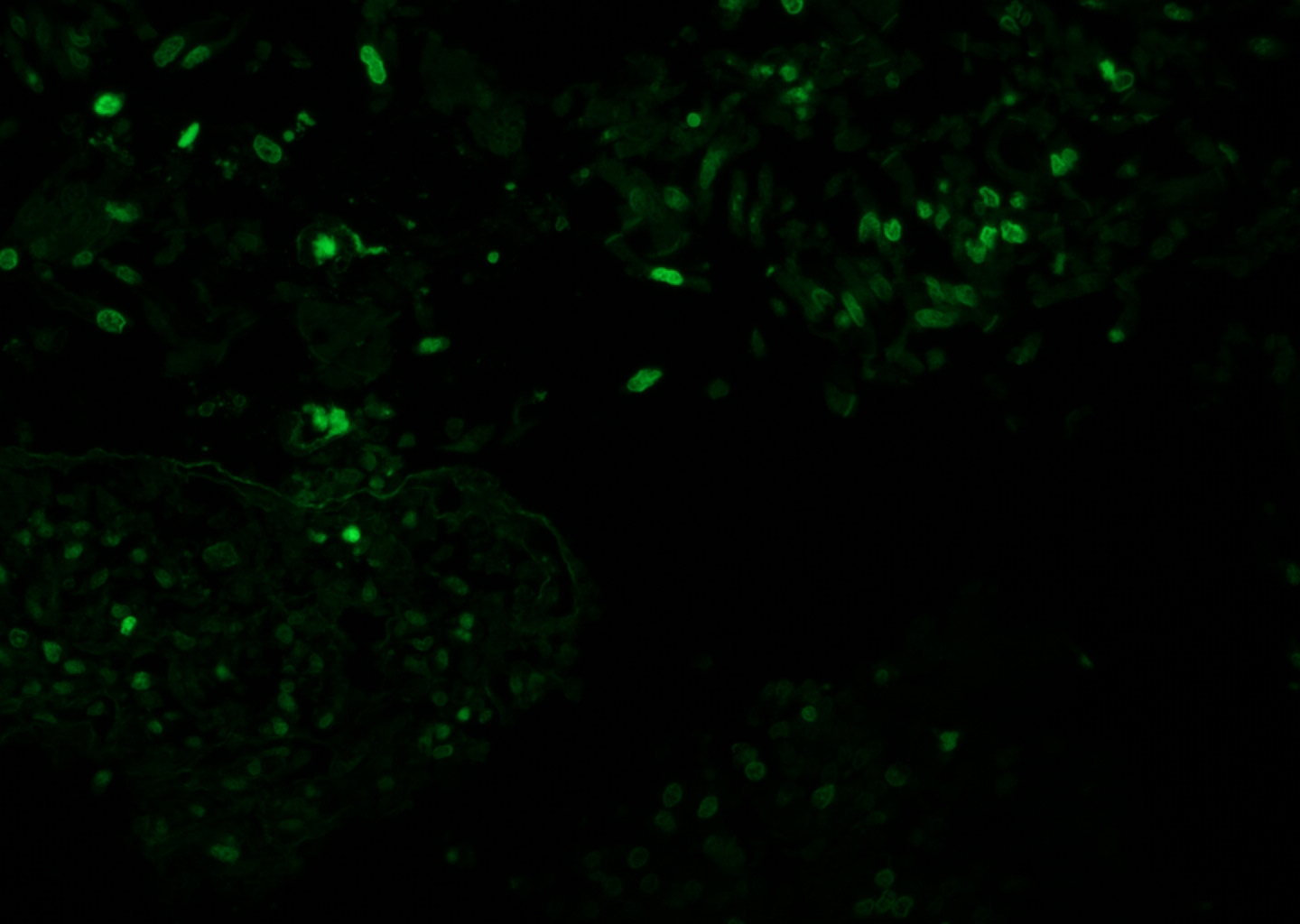

Supplement: Supplementary file 7 [file Data_Sheet_2.ZIP › CON group-Jejunal TUNEL apoptosis/400 x/CON-2 400-1.jpg]

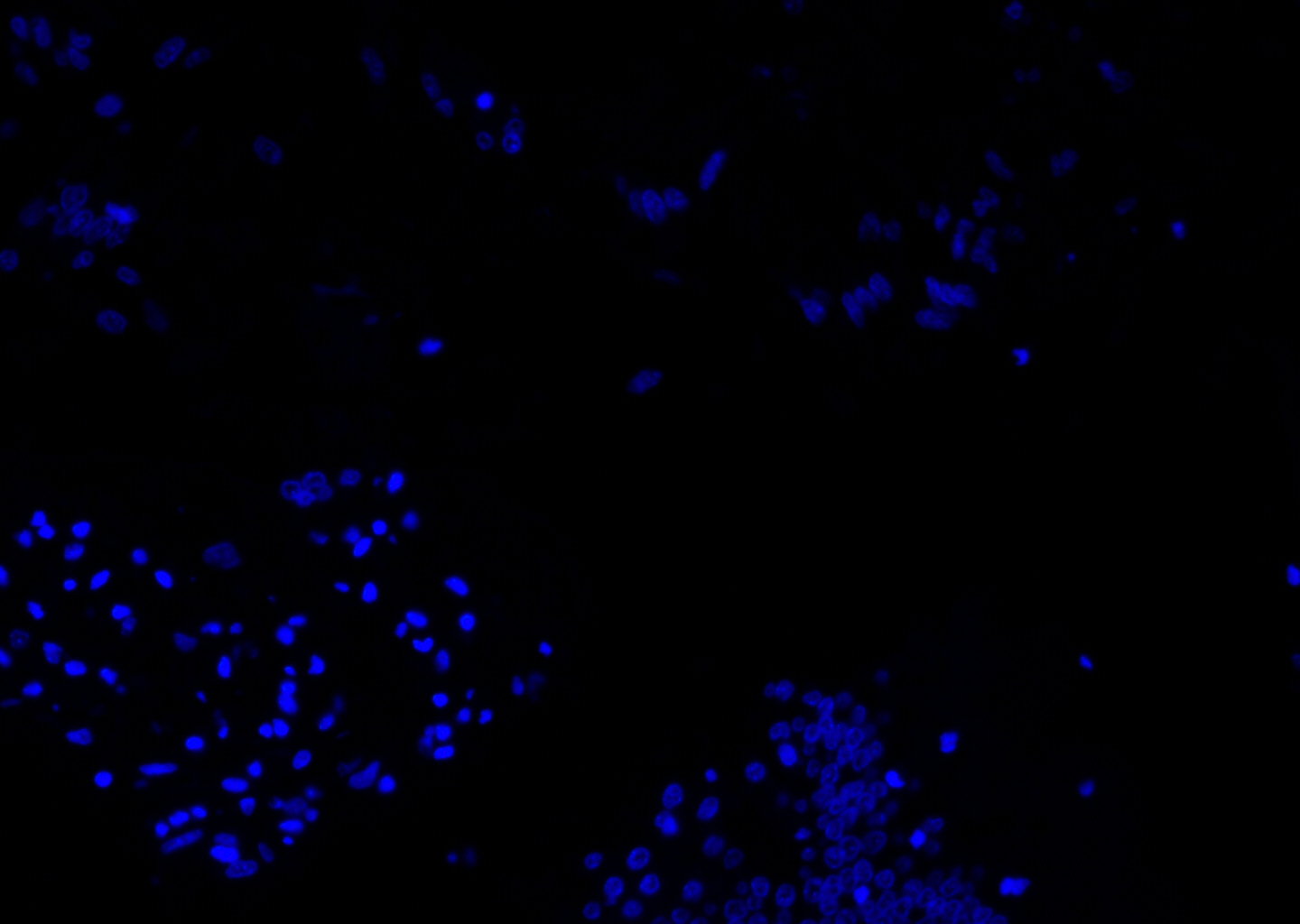

Supplement: Supplementary file 7 [file Data_Sheet_2.ZIP › CON group-Jejunal TUNEL apoptosis/400 x/CON-2 400-2.jpg]

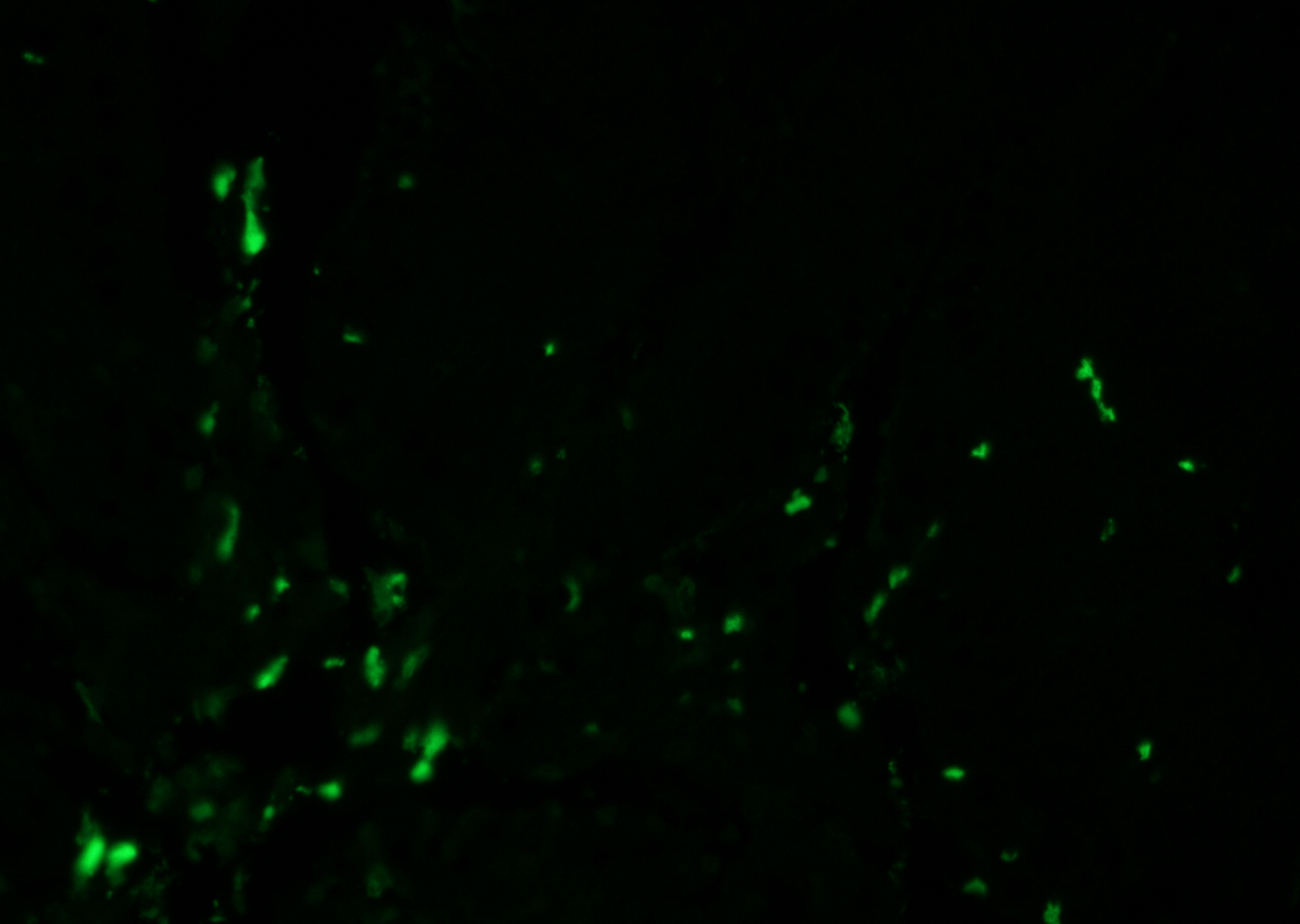

Supplement: Supplementary file 7 [file Data_Sheet_2.ZIP › CON group-Jejunal TUNEL apoptosis/400 x/CON-2 400-3.jpg]

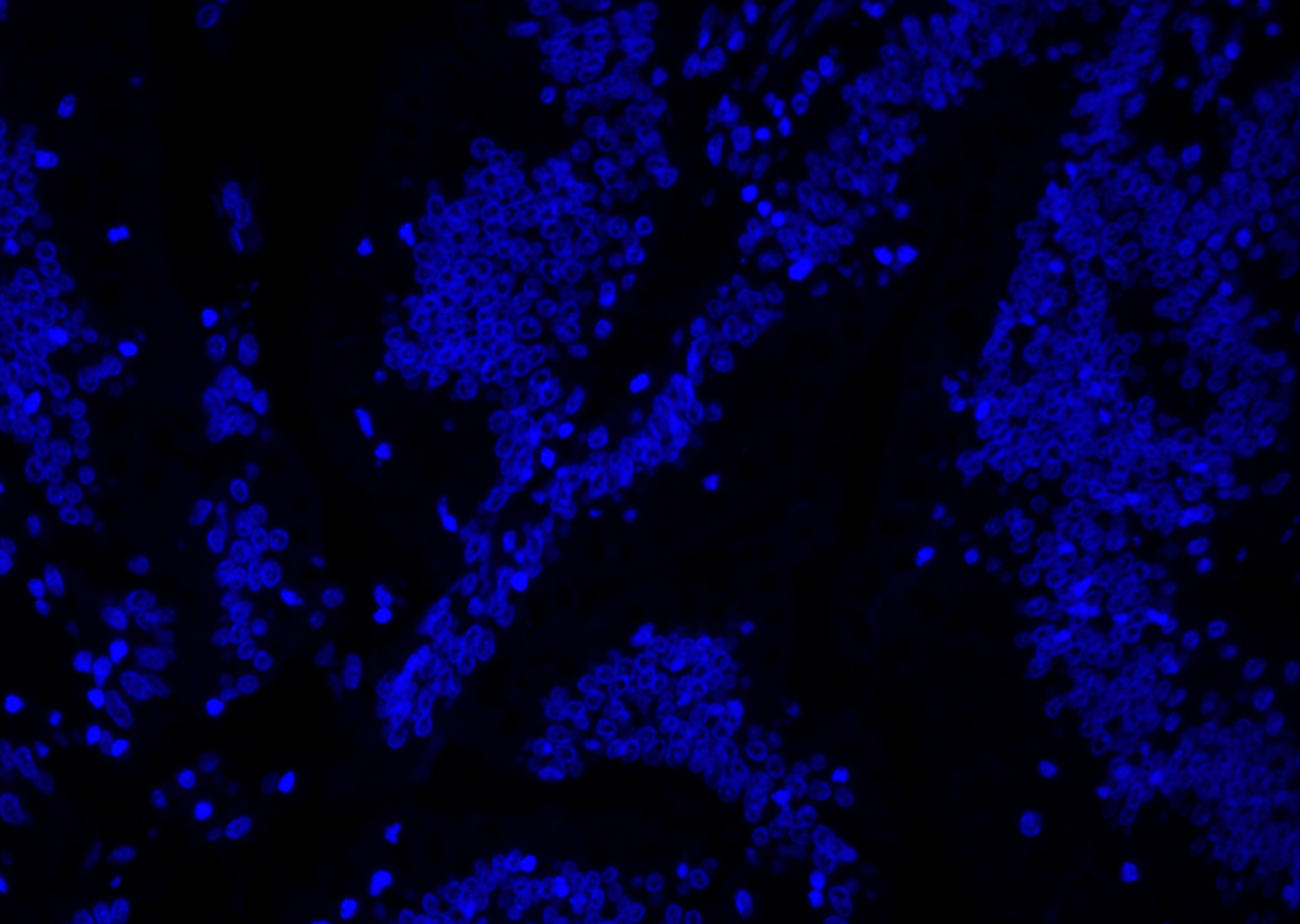

Supplement: Supplementary file 7 [file Data_Sheet_2.ZIP › CON group-Jejunal TUNEL apoptosis/400 x/CON-2 400-4.jpg]

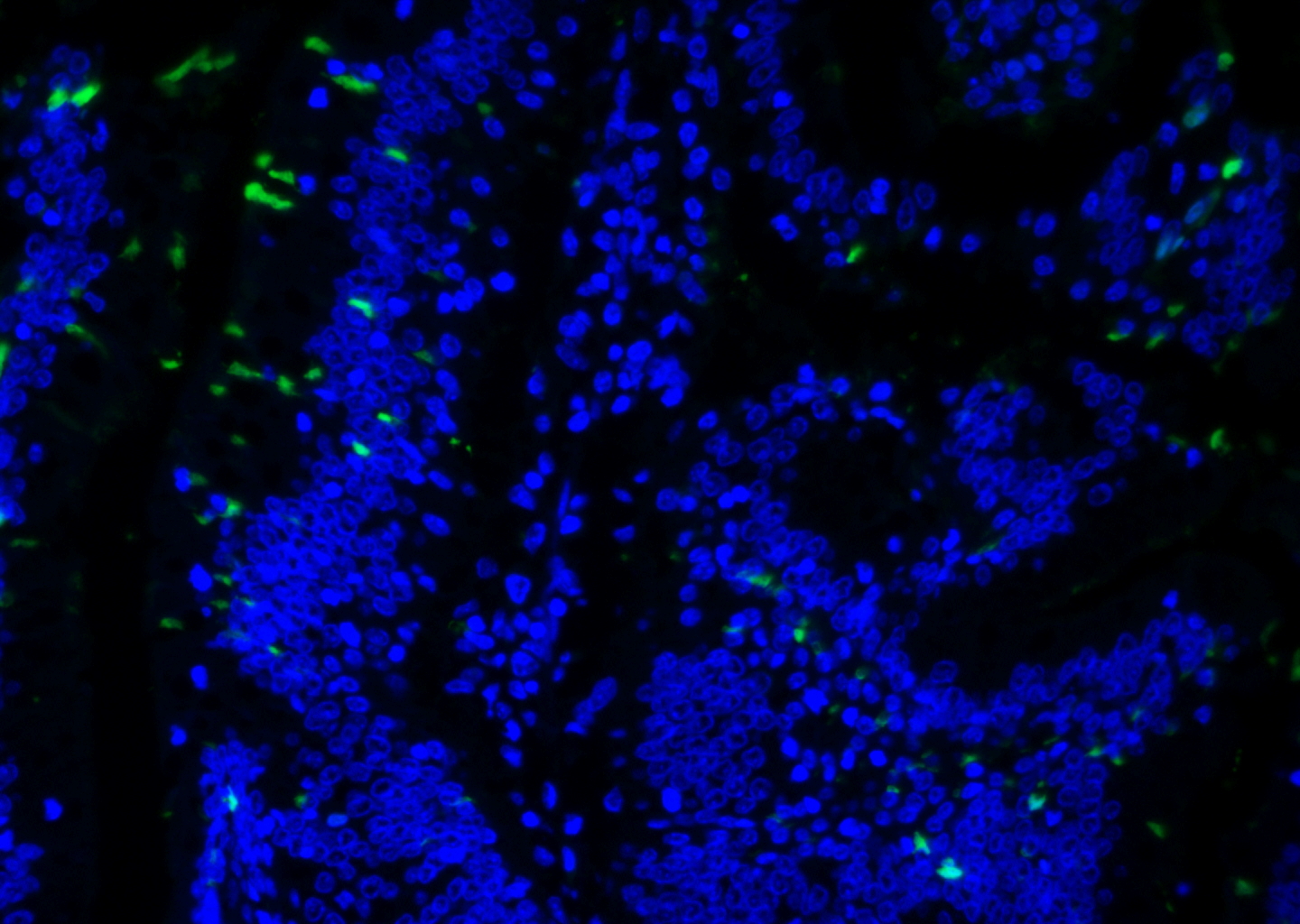

Supplement: Supplementary file 7 [file Data_Sheet_2.ZIP › CON group-Jejunal TUNEL apoptosis/400 x/CON-2 400-5 6.jpg]

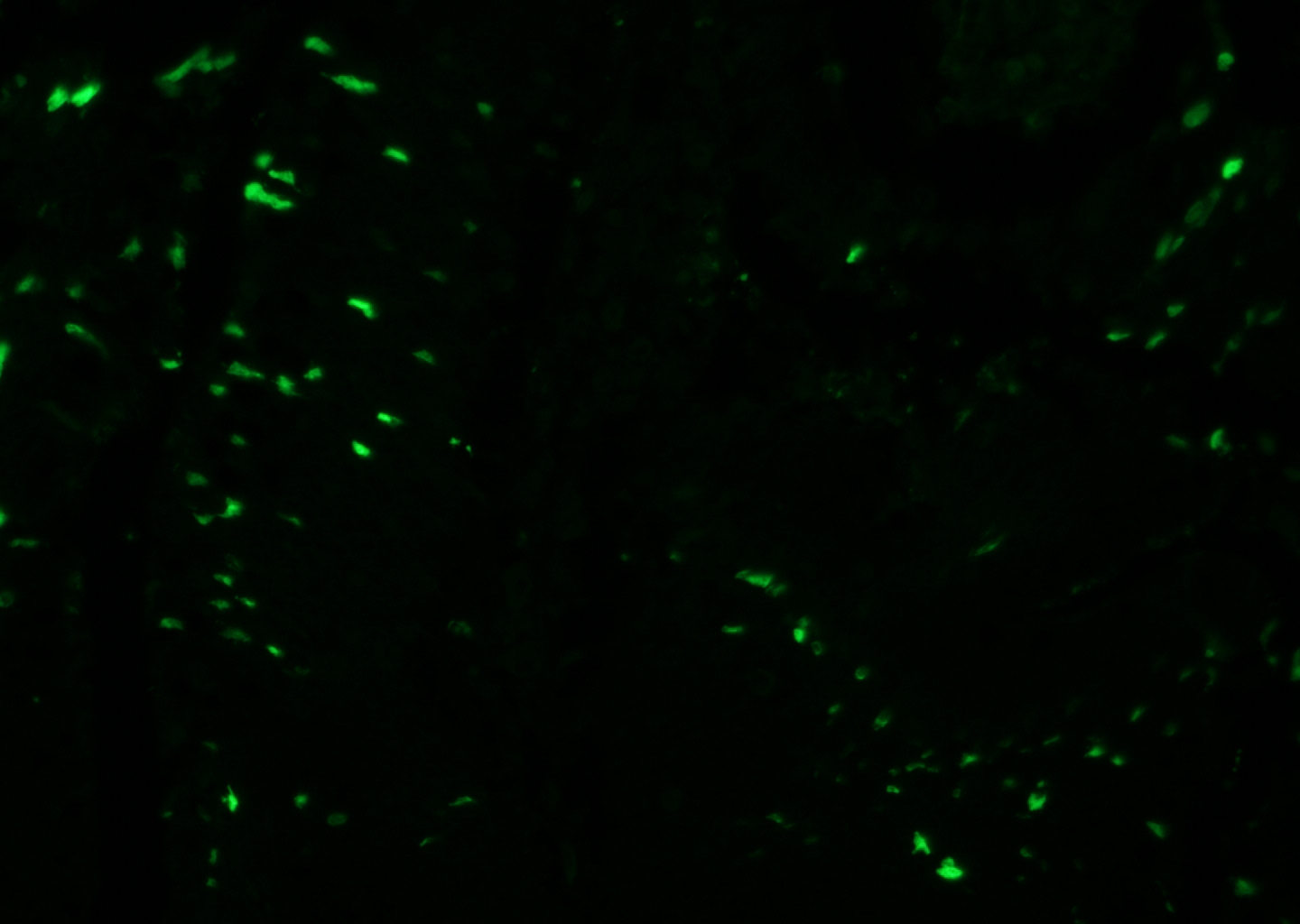

Supplement: Supplementary file 7 [file Data_Sheet_2.ZIP › CON group-Jejunal TUNEL apoptosis/400 x/CON-2 400-5.jpg]

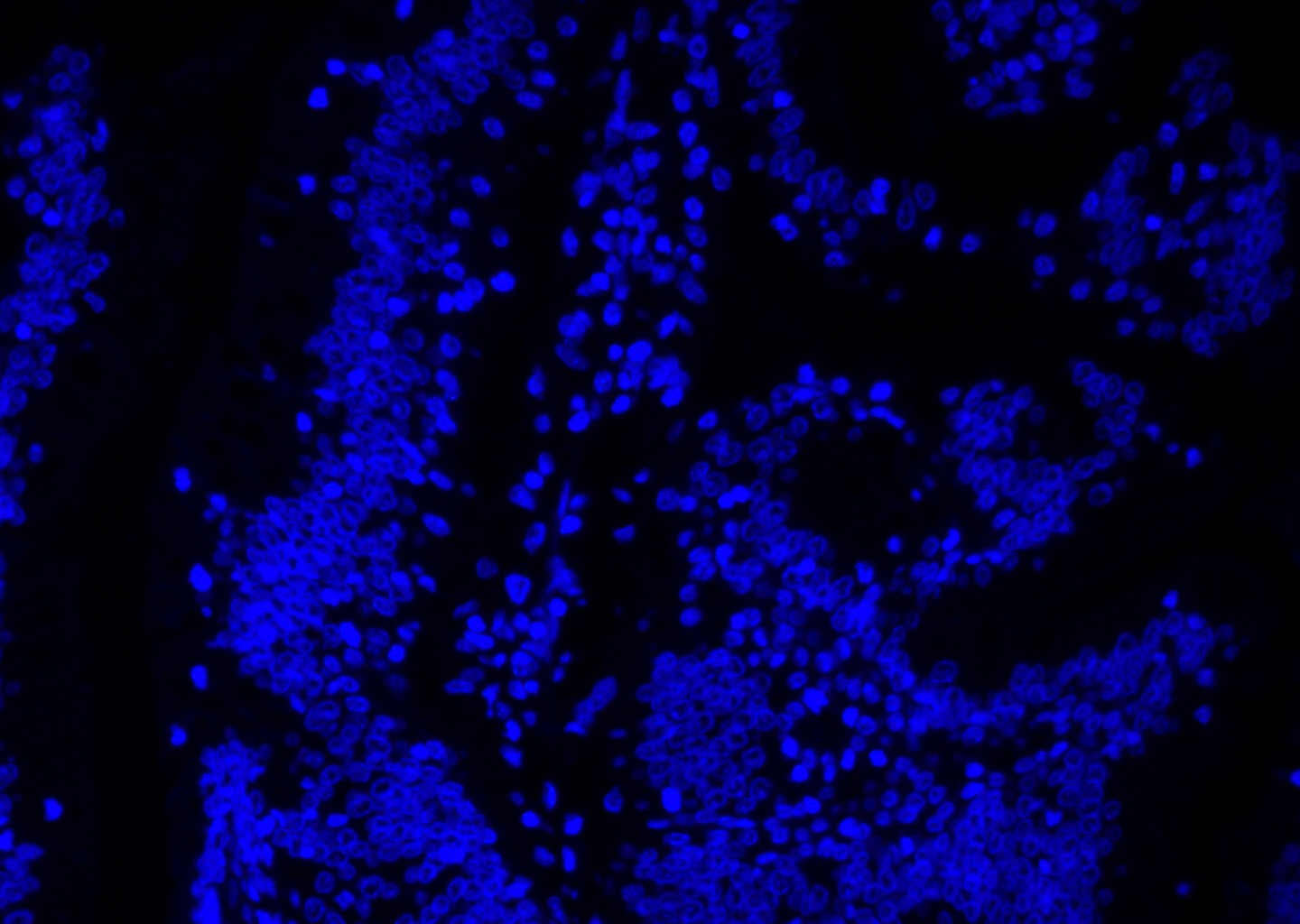

Supplement: Supplementary file 7 [file Data_Sheet_2.ZIP › CON group-Jejunal TUNEL apoptosis/400 x/CON-2 400-6.jpg]

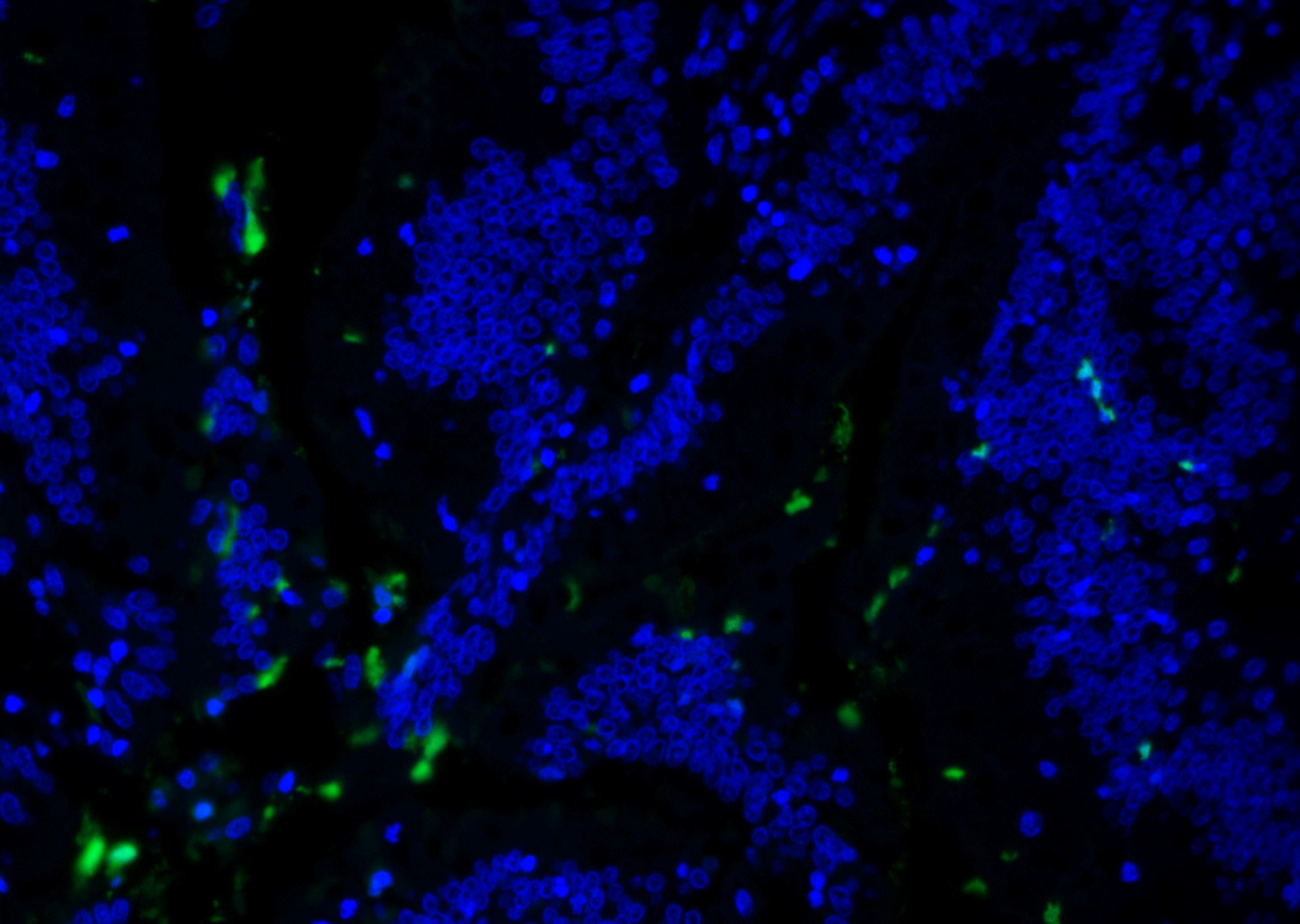

Supplement: Supplementary file 7 [file Data_Sheet_2.ZIP › CON group-Jejunal TUNEL apoptosis/400 x/CON-2 400-3 4.jpg]

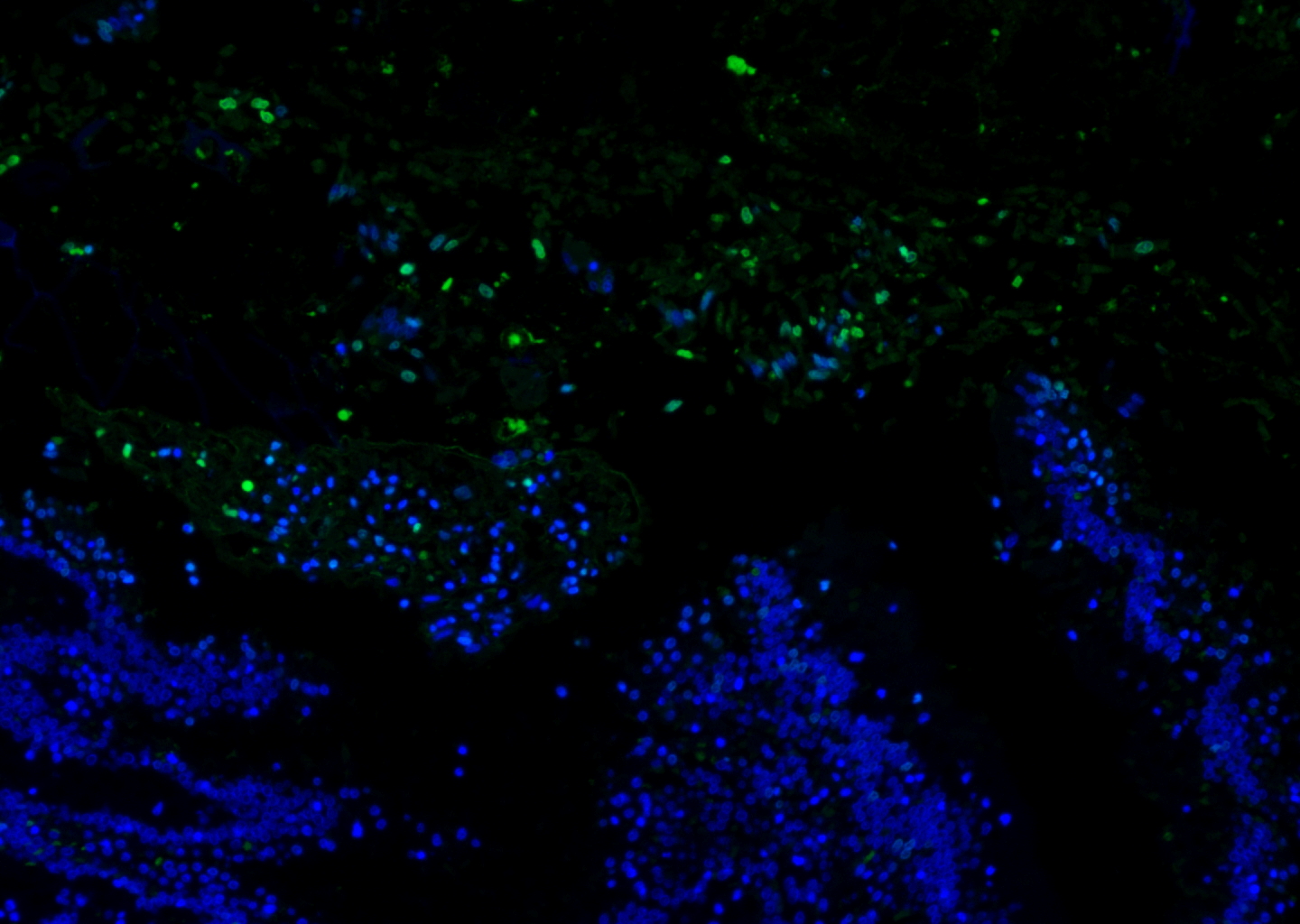

Supplement: Supplementary file 8 [file Data_Sheet_3.ZIP › NE group-Jejunal TUNEL apoptosis/200 x/NE-1 200-1 2.jpg]

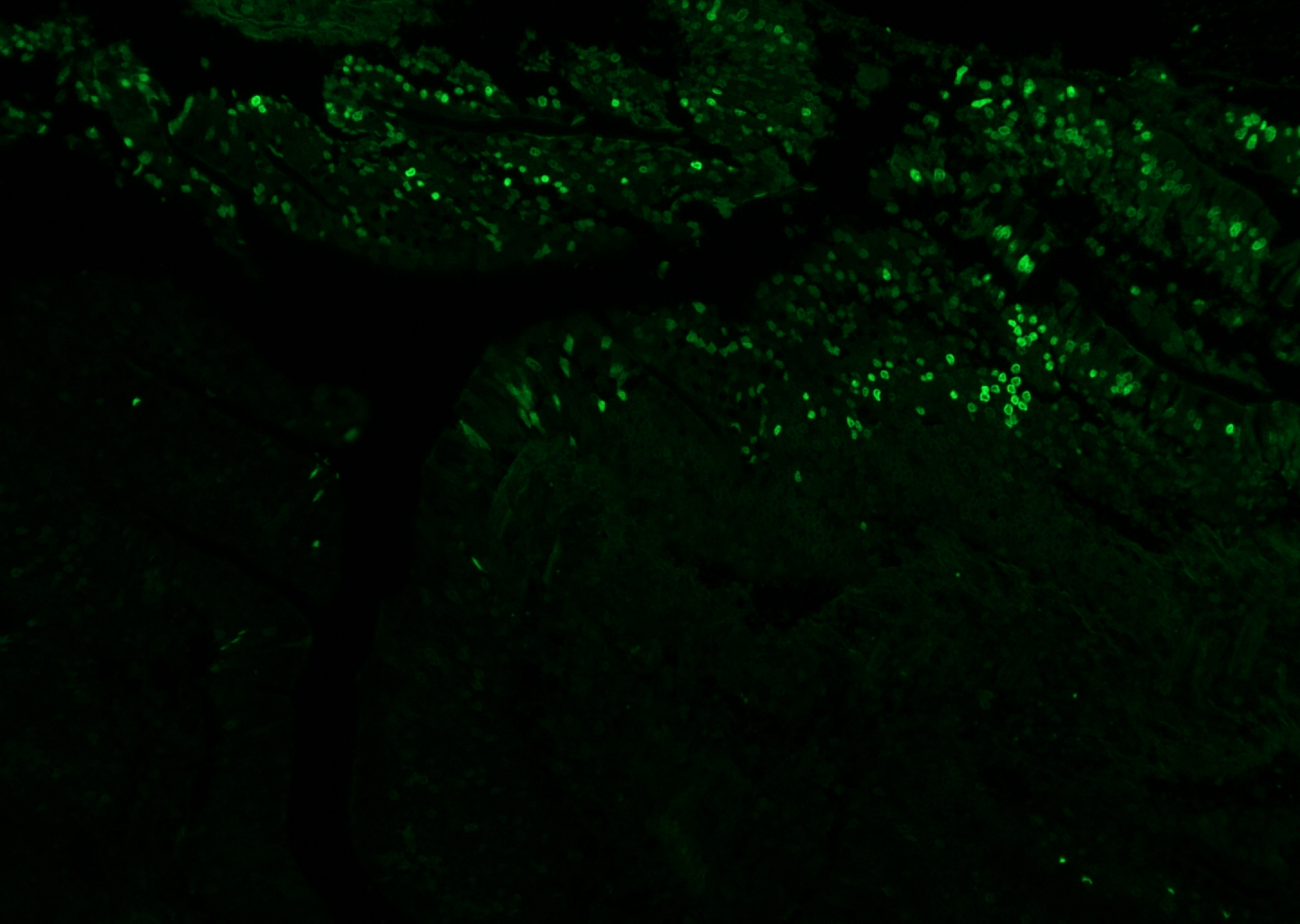

Supplement: Supplementary file 8 [file Data_Sheet_3.ZIP › NE group-Jejunal TUNEL apoptosis/200 x/NE-1 200-1.jpg]

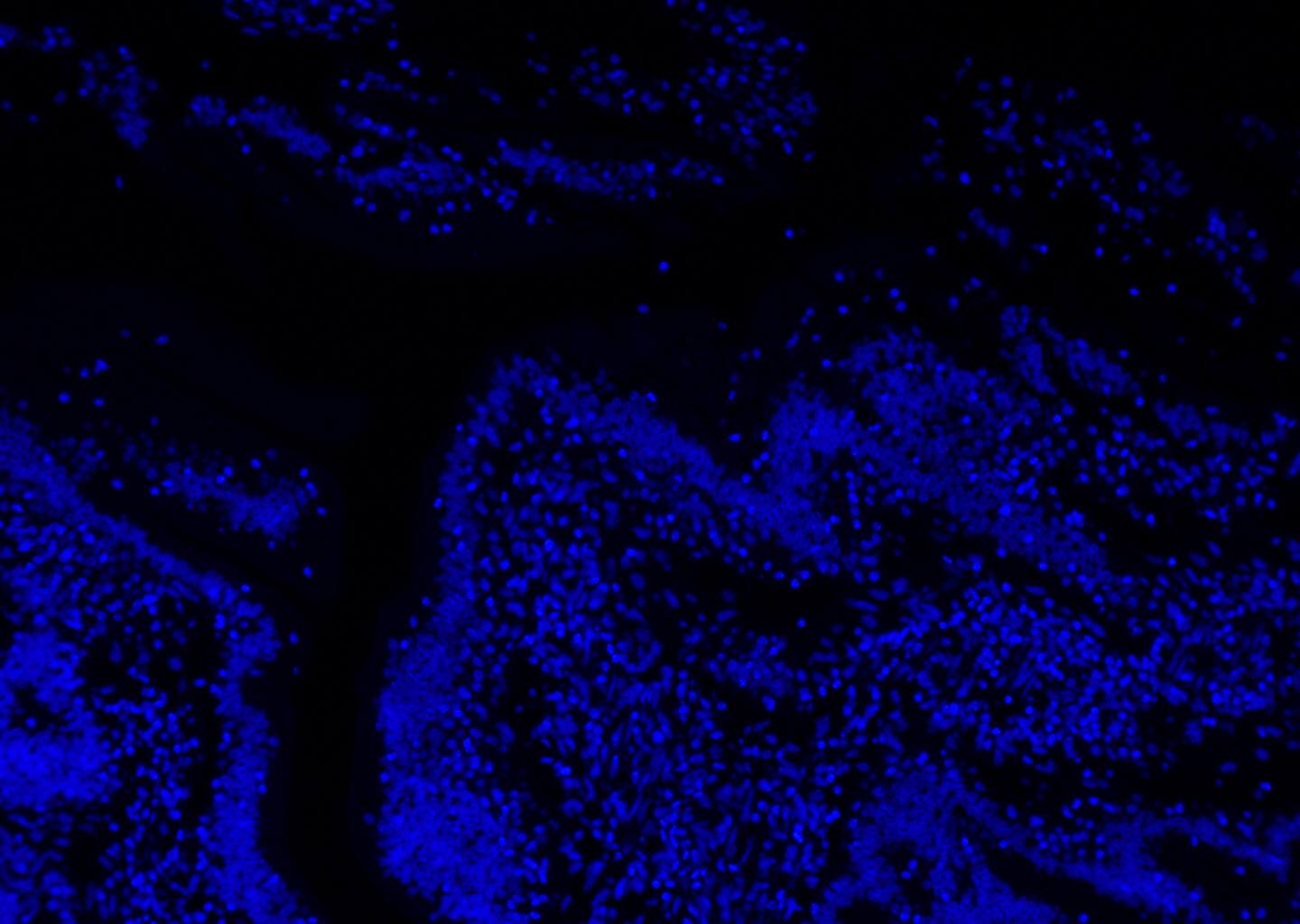

Supplement: Supplementary file 8 [file Data_Sheet_3.ZIP › NE group-Jejunal TUNEL apoptosis/200 x/NE-1 200-2.jpg]

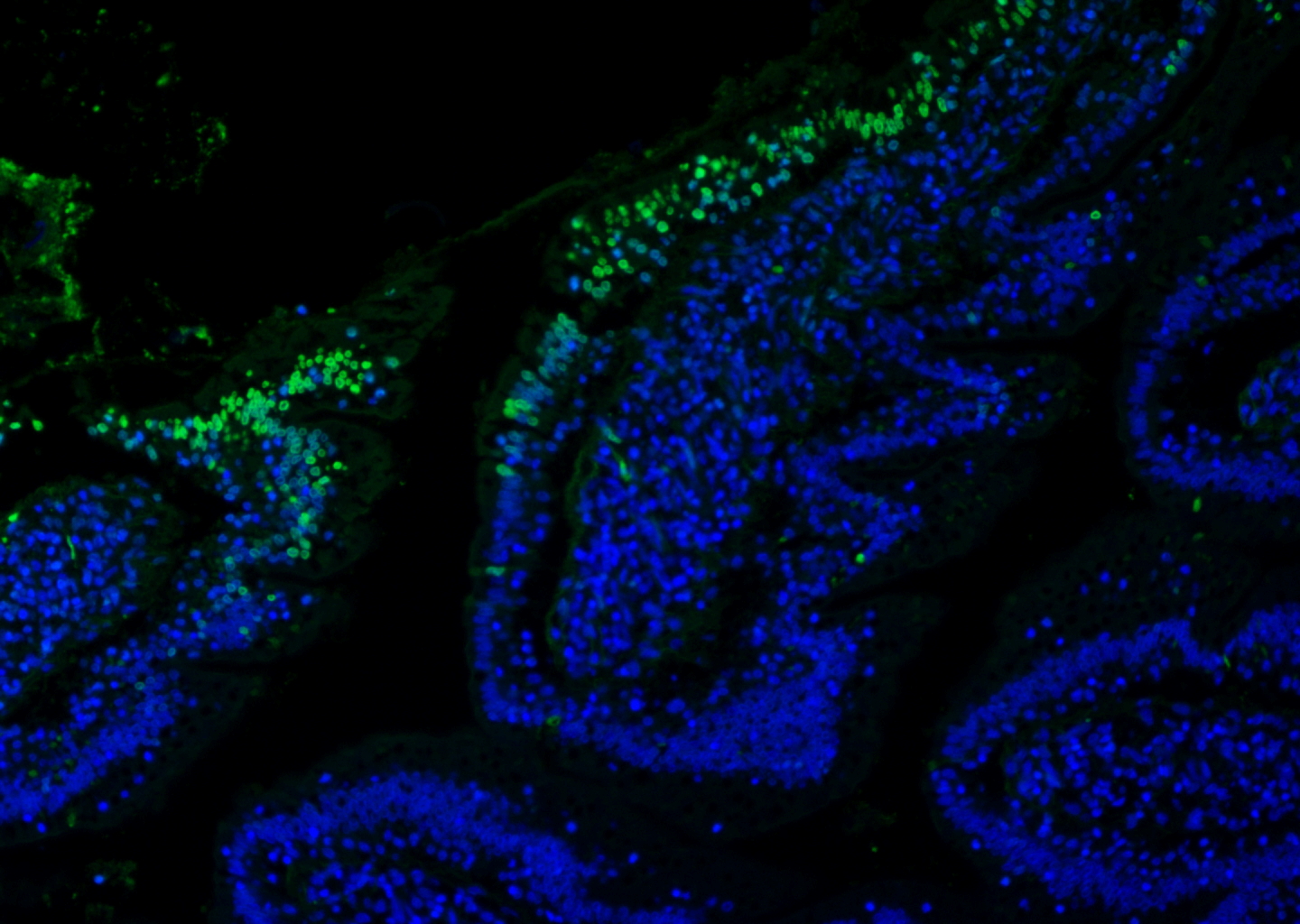

Supplement: Supplementary file 8 [file Data_Sheet_3.ZIP › NE group-Jejunal TUNEL apoptosis/200 x/NE-1 200-3 4.jpg]

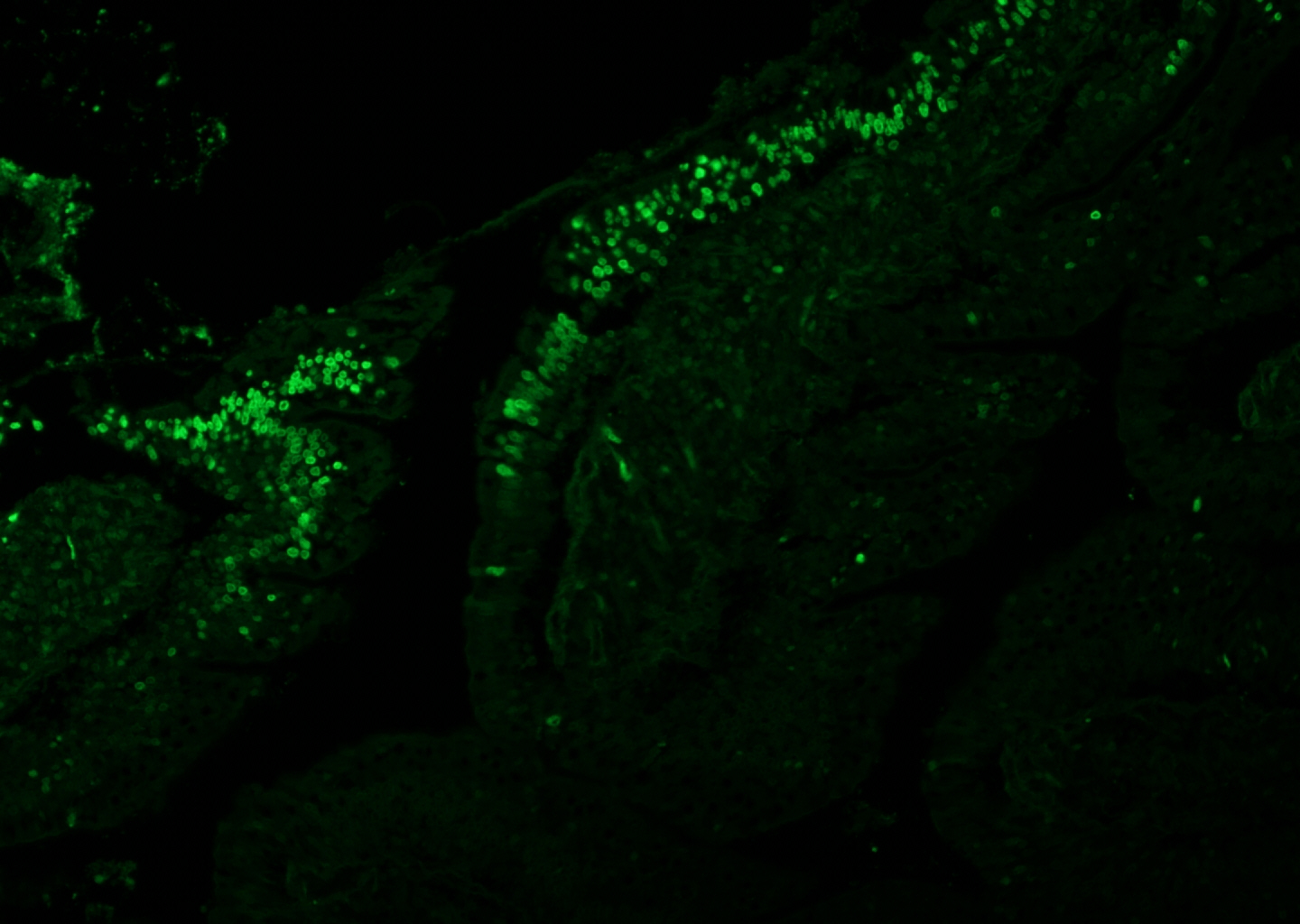

Supplement: Supplementary file 8 [file Data_Sheet_3.ZIP › NE group-Jejunal TUNEL apoptosis/200 x/NE-1 200-3.jpg]

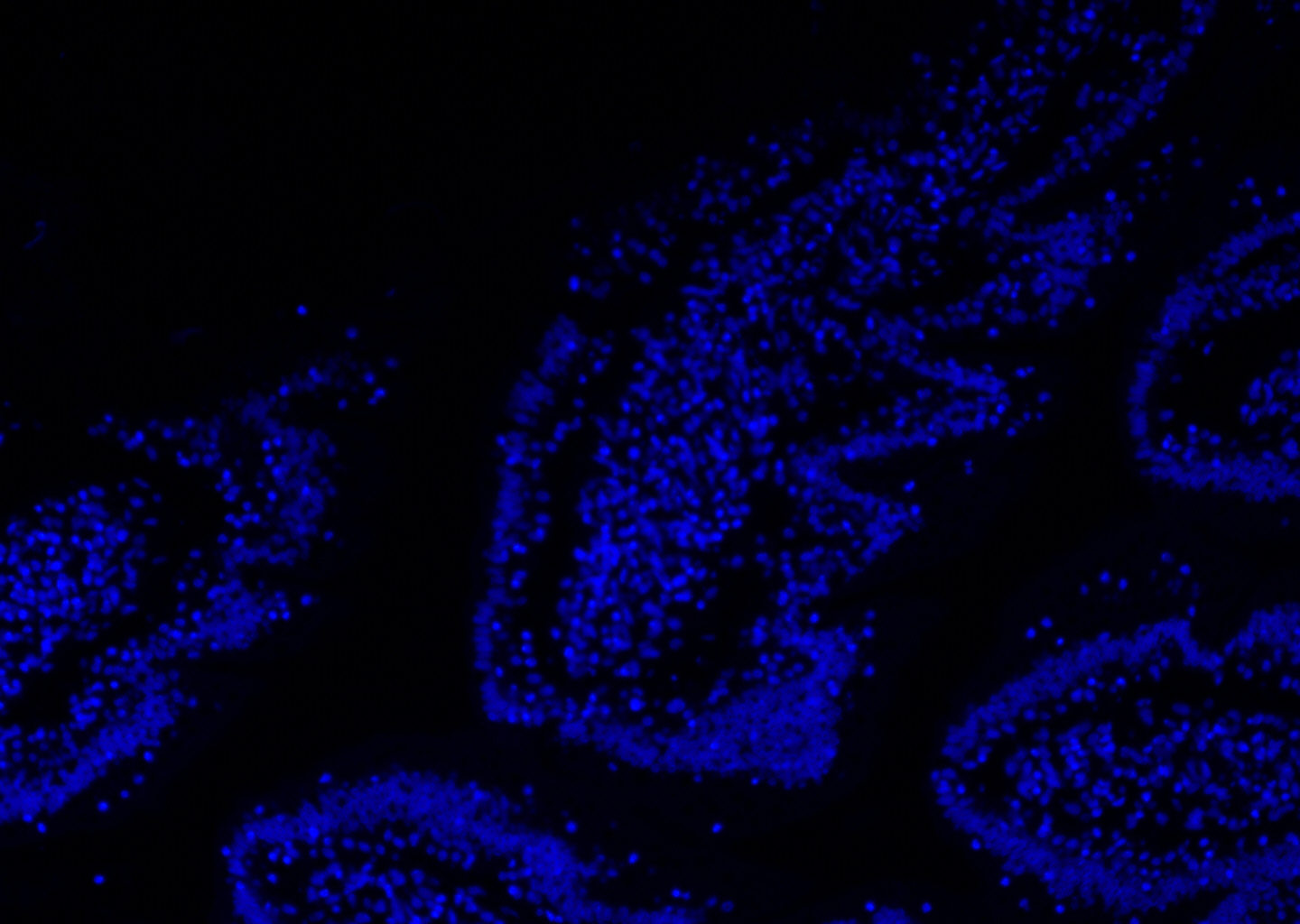

Supplement: Supplementary file 8 [file Data_Sheet_3.ZIP › NE group-Jejunal TUNEL apoptosis/200 x/NE-1 200-4.jpg]

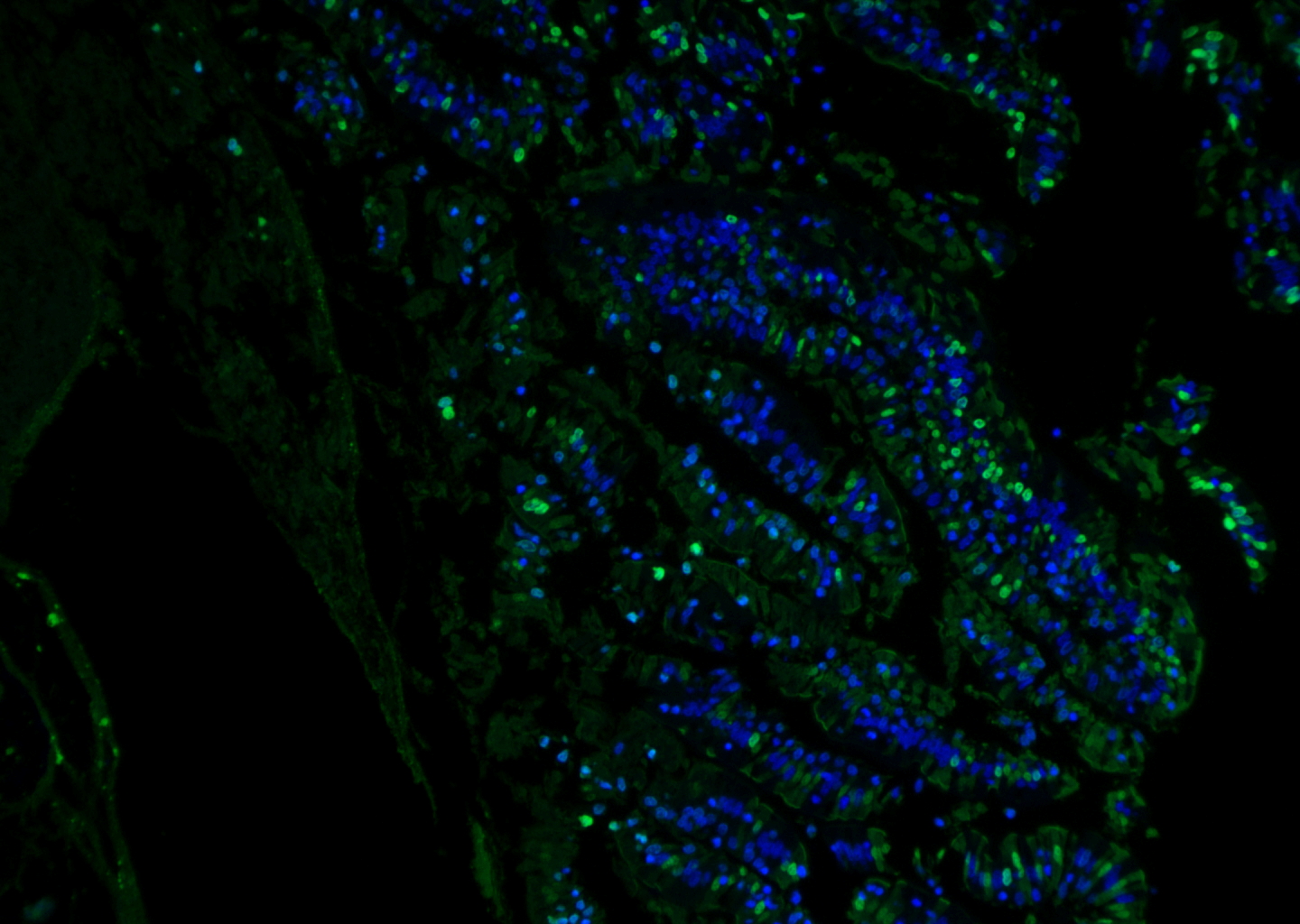

Supplement: Supplementary file 8 [file Data_Sheet_3.ZIP › NE group-Jejunal TUNEL apoptosis/200 x/NE-1 200-5 6.jpg]

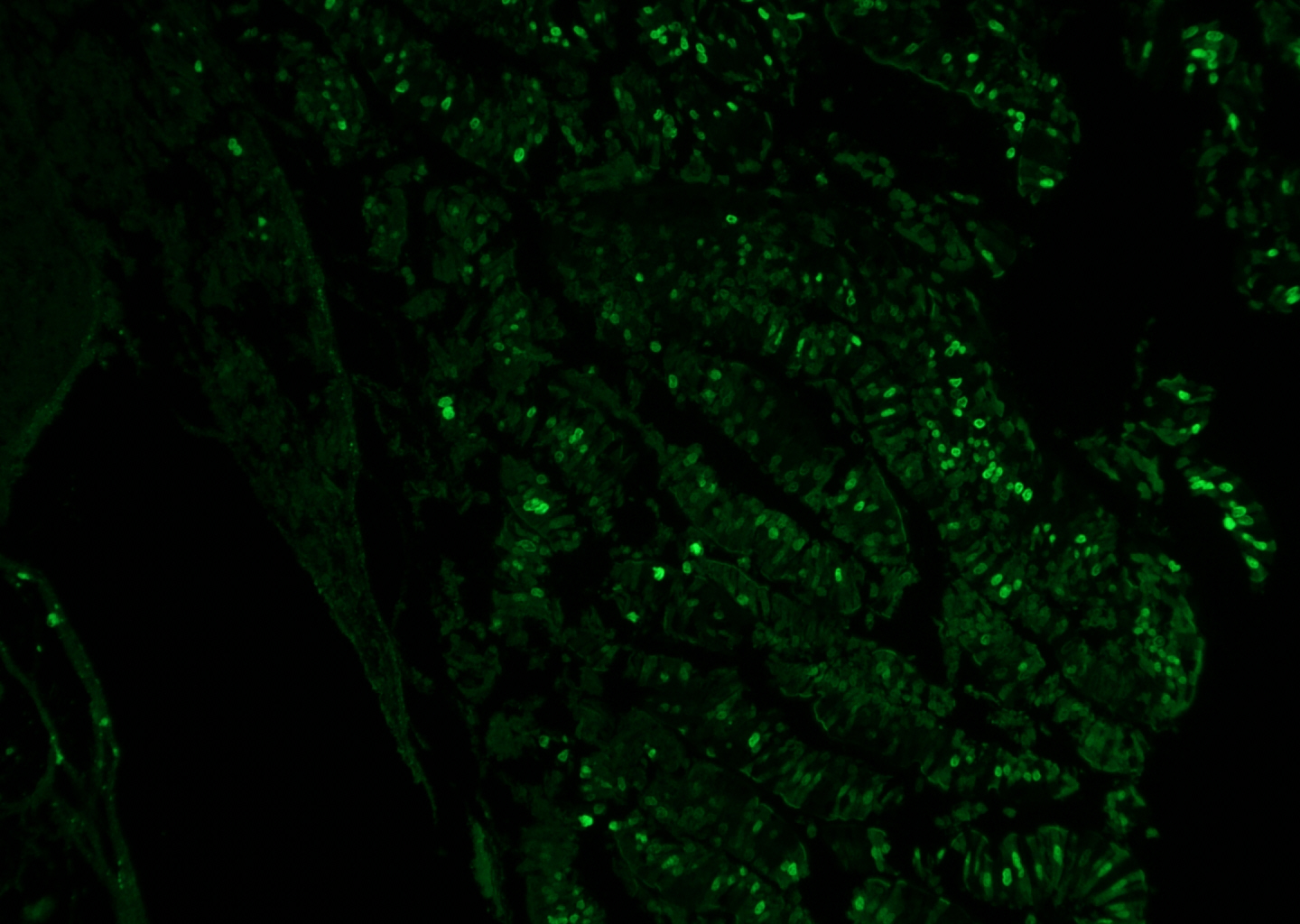

Supplement: Supplementary file 8 [file Data_Sheet_3.ZIP › NE group-Jejunal TUNEL apoptosis/200 x/NE-1 200-5.jpg]

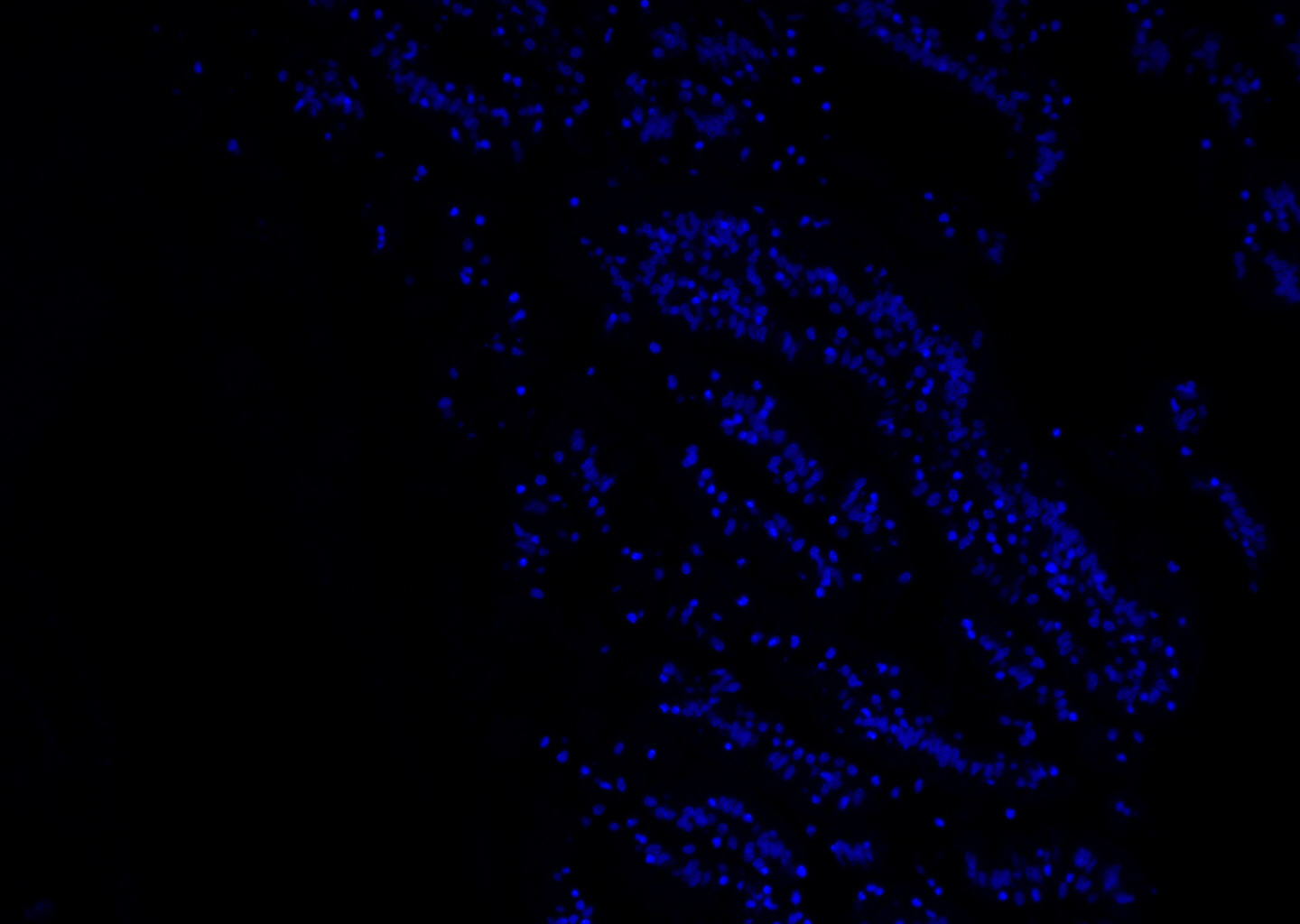

Supplement: Supplementary file 8 [file Data_Sheet_3.ZIP › NE group-Jejunal TUNEL apoptosis/200 x/NE-1 200-6.jpg]

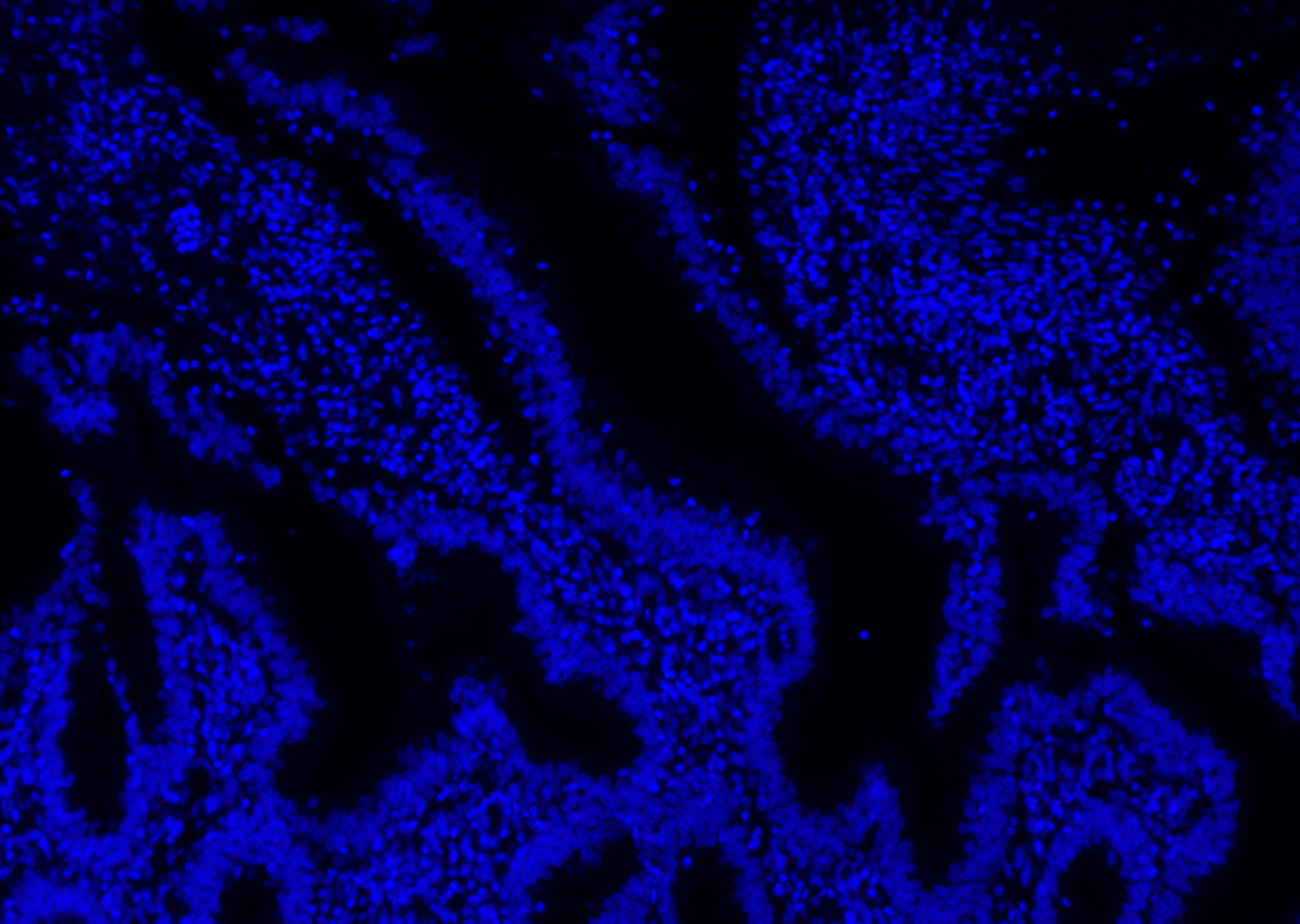

Supplement: Supplementary file 8 [file Data_Sheet_3.ZIP › NE group-Jejunal TUNEL apoptosis/200 x/NE-2 200-2.jpg]

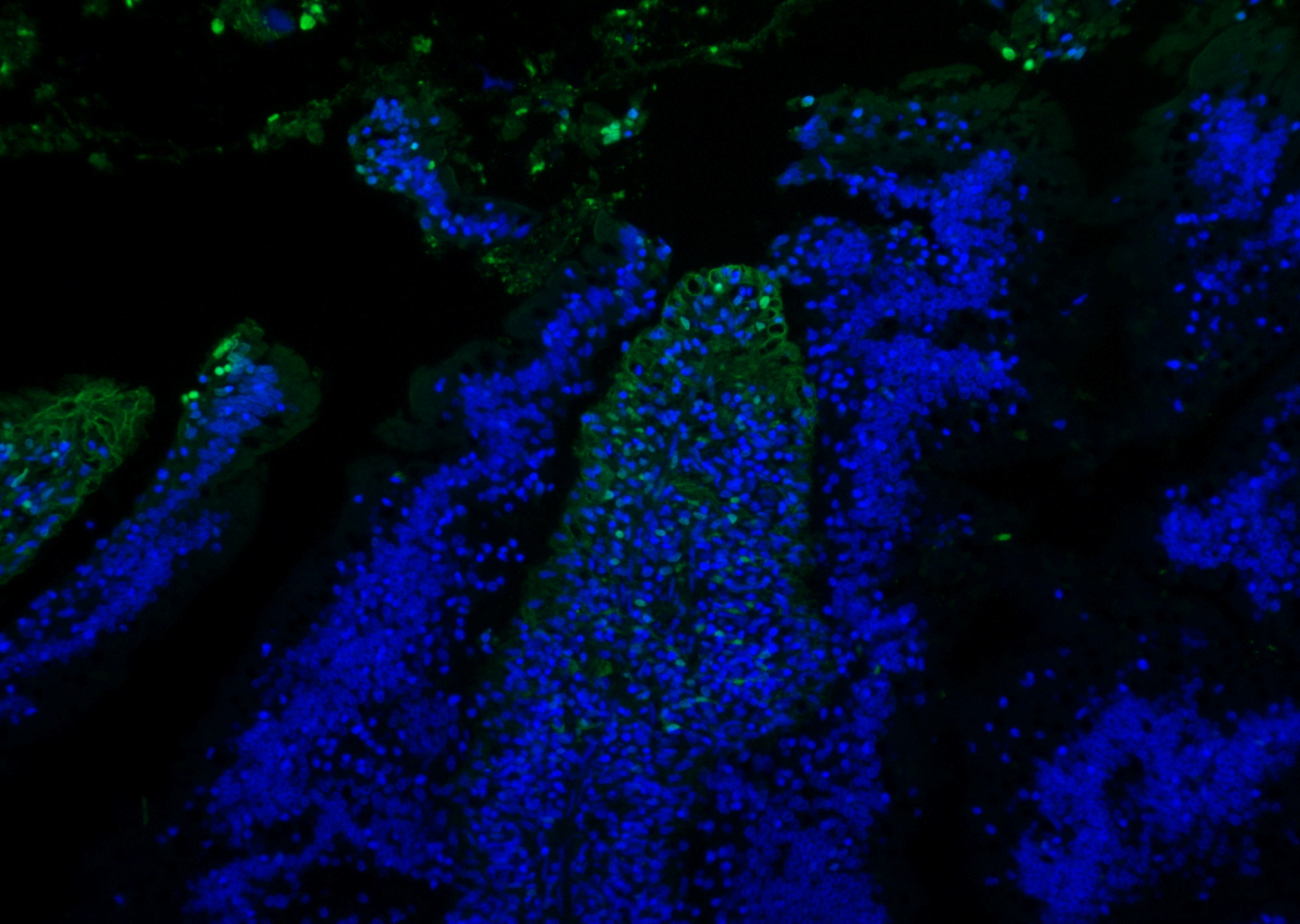

Supplement: Supplementary file 8 [file Data_Sheet_3.ZIP › NE group-Jejunal TUNEL apoptosis/200 x/NE-2 200-3 4.jpg]

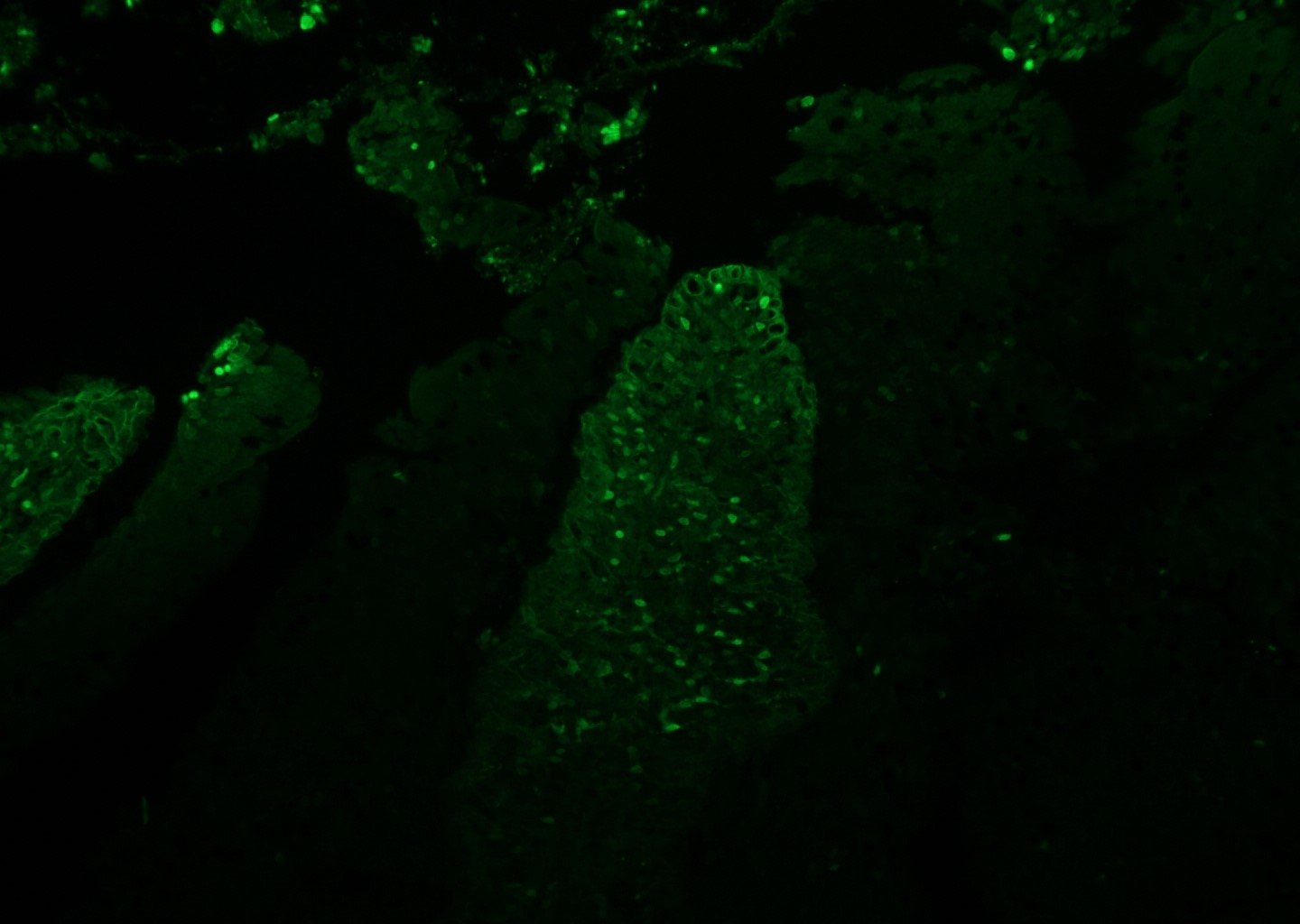

Supplement: Supplementary file 8 [file Data_Sheet_3.ZIP › NE group-Jejunal TUNEL apoptosis/200 x/NE-2 200-3.jpg]

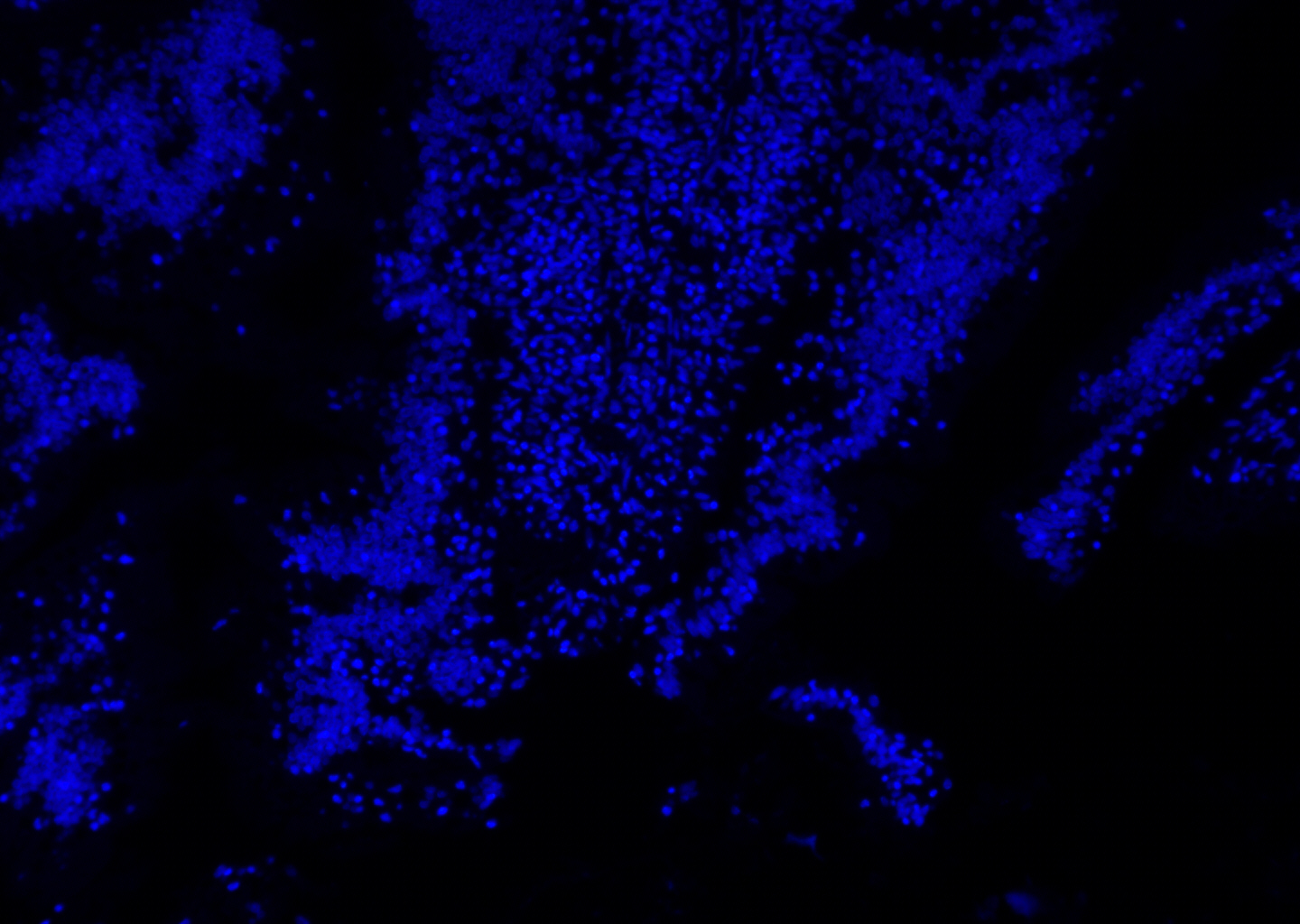

Supplement: Supplementary file 8 [file Data_Sheet_3.ZIP › NE group-Jejunal TUNEL apoptosis/200 x/NE-2 200-4.jpg]

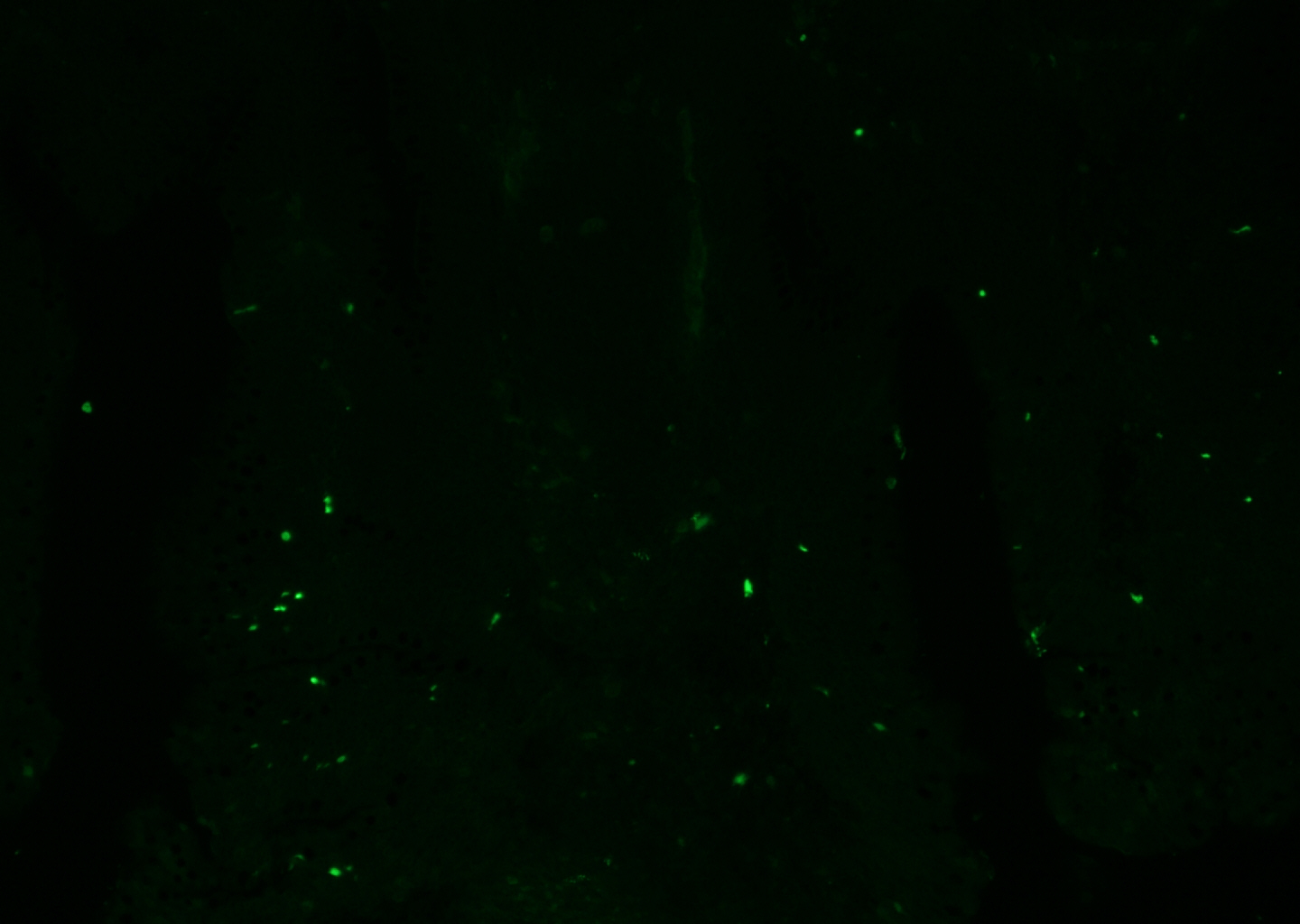

Supplement: Supplementary file 8 [file Data_Sheet_3.ZIP › NE group-Jejunal TUNEL apoptosis/200 x/NE-2 200-5.jpg]

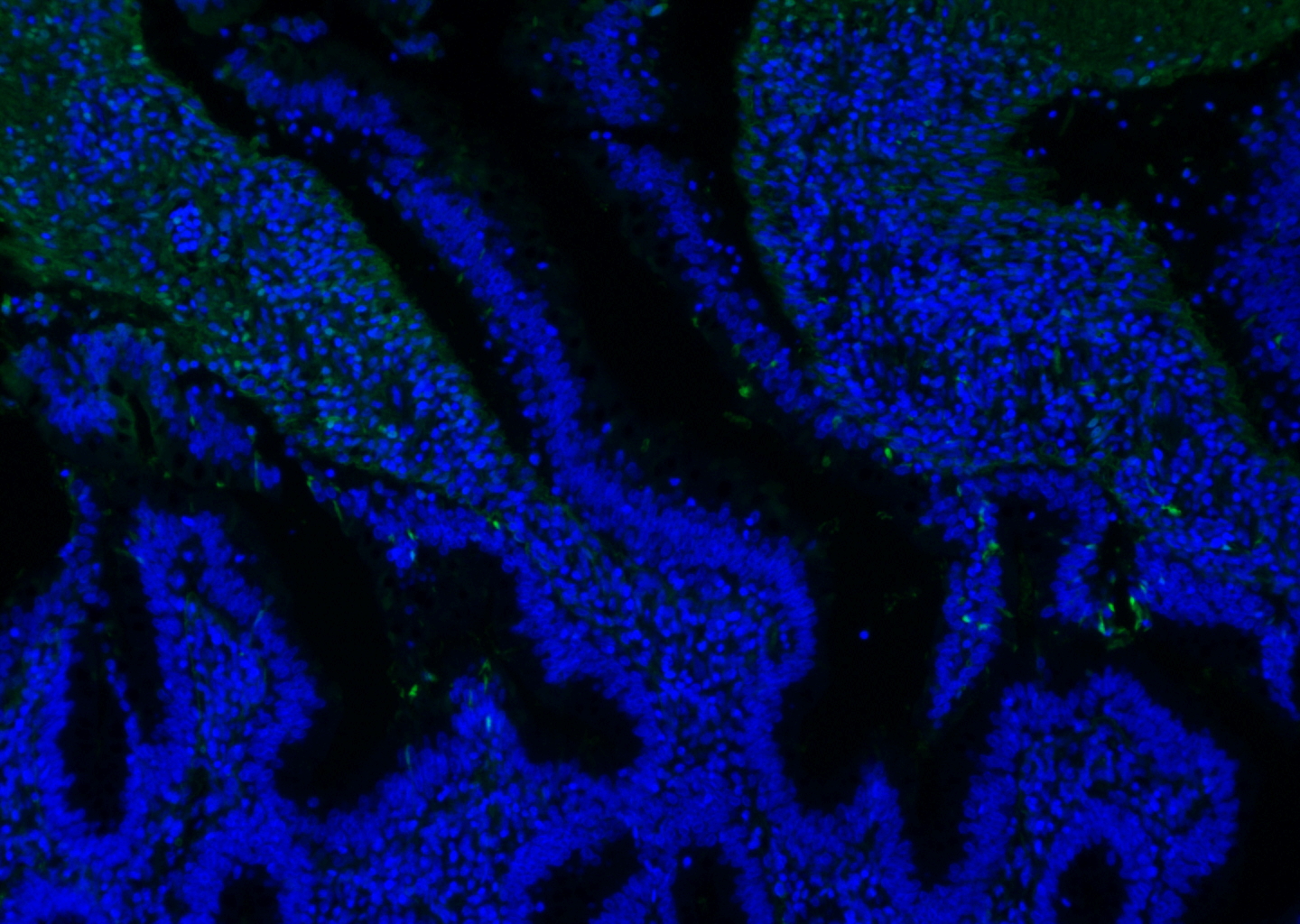

Supplement: Supplementary file 8 [file Data_Sheet_3.ZIP › NE group-Jejunal TUNEL apoptosis/200 x/NE-2 200-1 2.jpg]

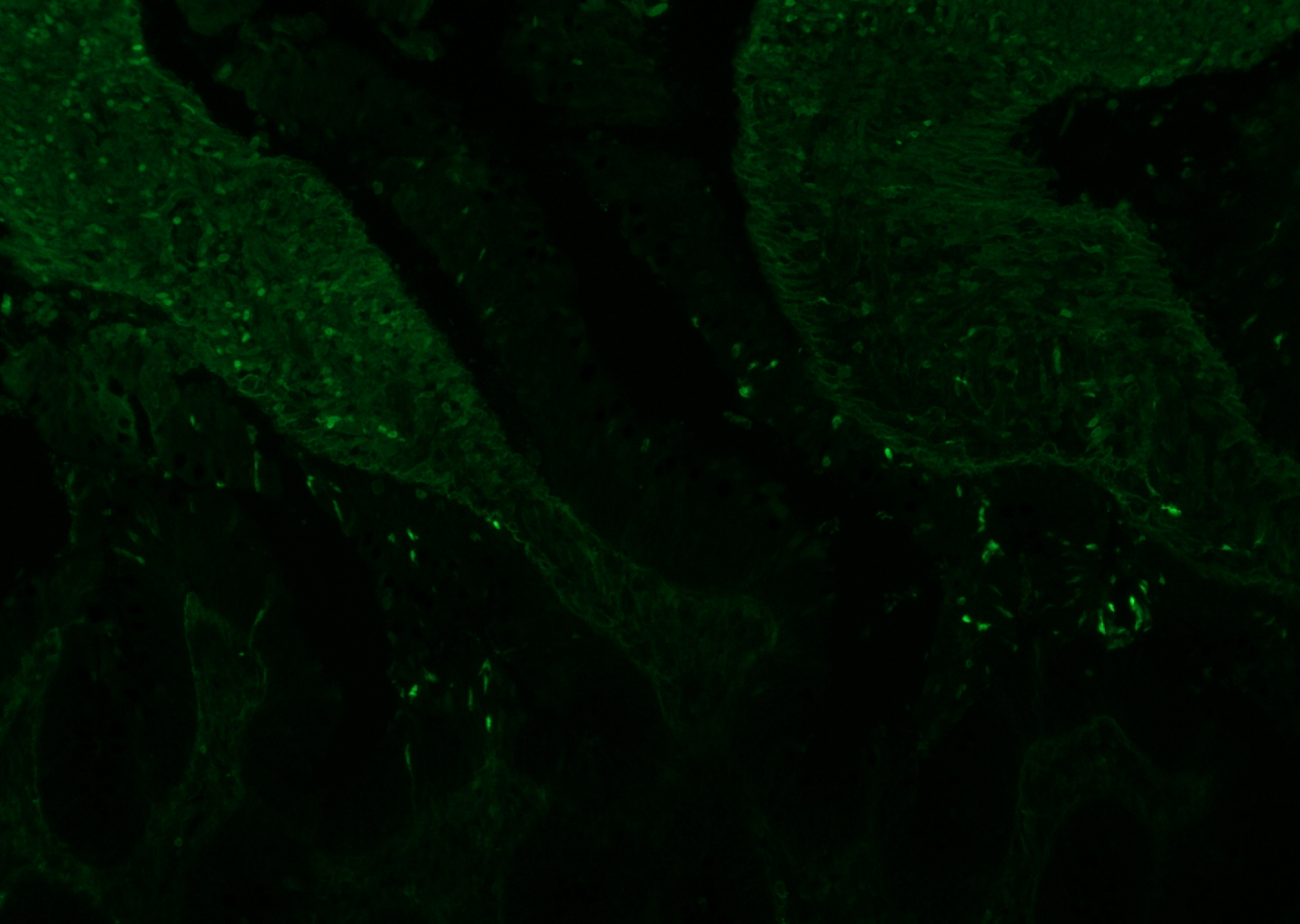

Supplement: Supplementary file 8 [file Data_Sheet_3.ZIP › NE group-Jejunal TUNEL apoptosis/200 x/NE-2 200-1.jpg]

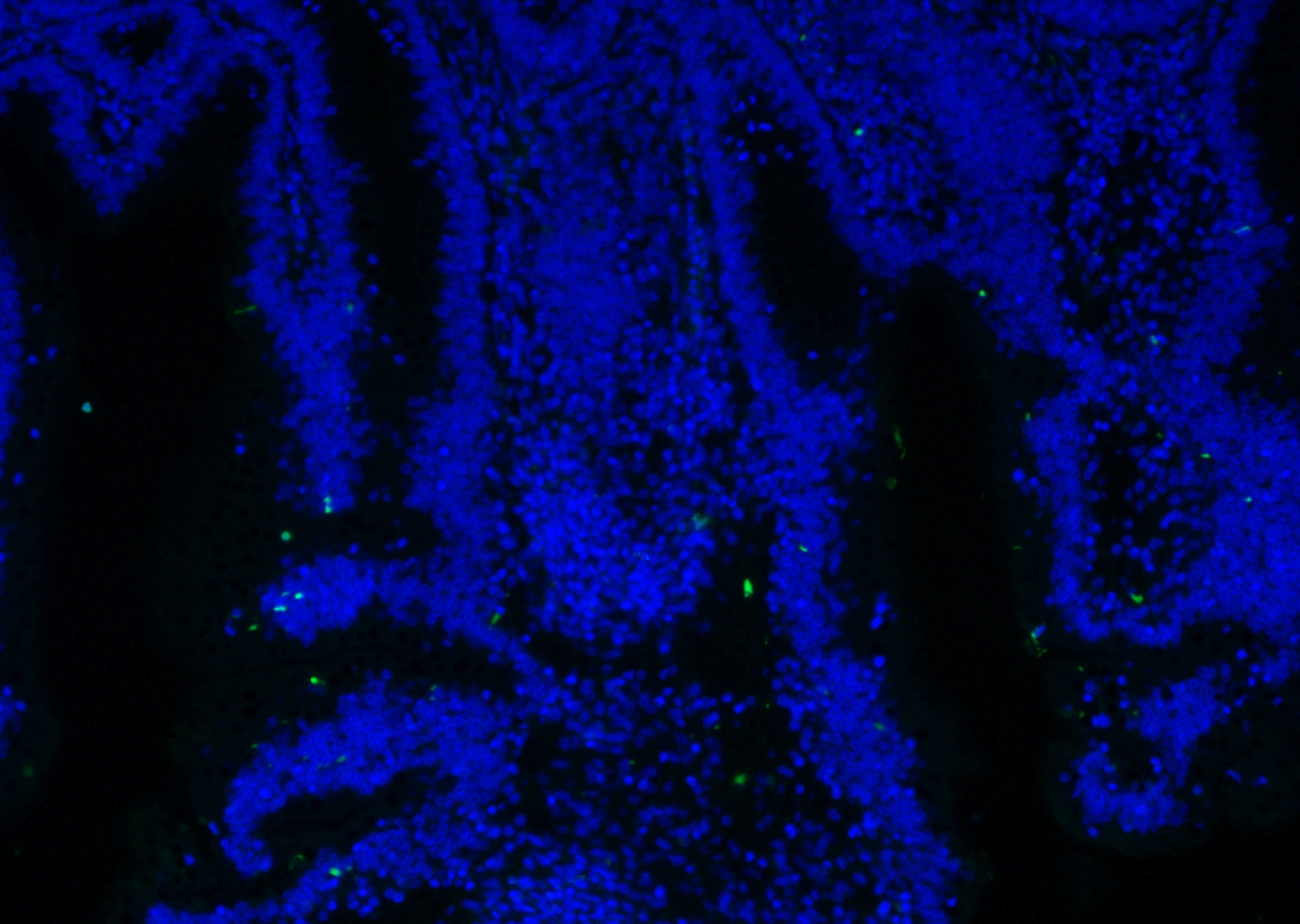

Supplement: Supplementary file 8 [file Data_Sheet_3.ZIP › NE group-Jejunal TUNEL apoptosis/200 x/NE-2 200-5 6.jpg]

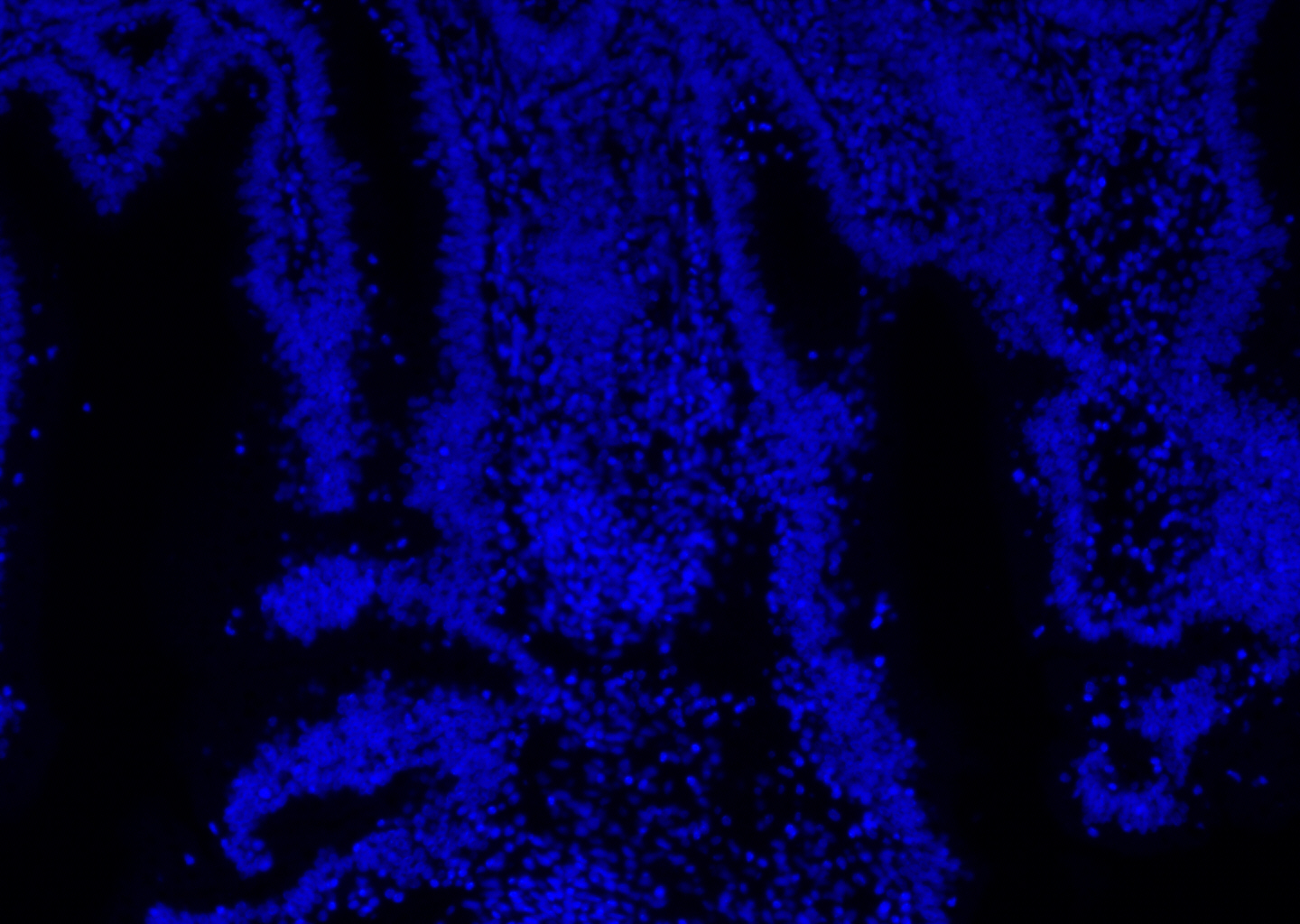

Supplement: Supplementary file 8 [file Data_Sheet_3.ZIP › NE group-Jejunal TUNEL apoptosis/200 x/NE-2 200-6.jpg]

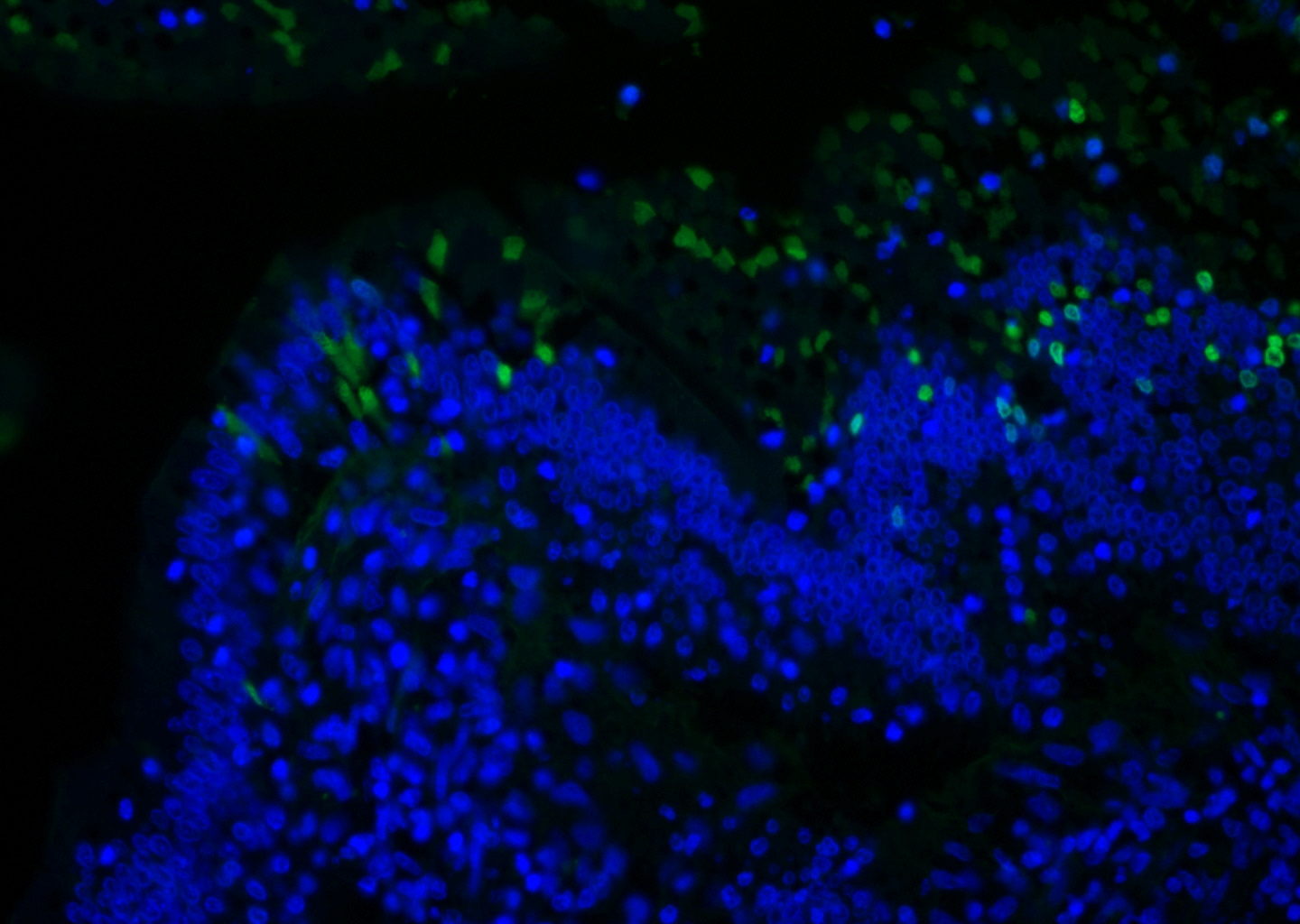

Supplement: Supplementary file 8 [file Data_Sheet_3.ZIP › NE group-Jejunal TUNEL apoptosis/400 x/NE-1 400-1 2.jpg]

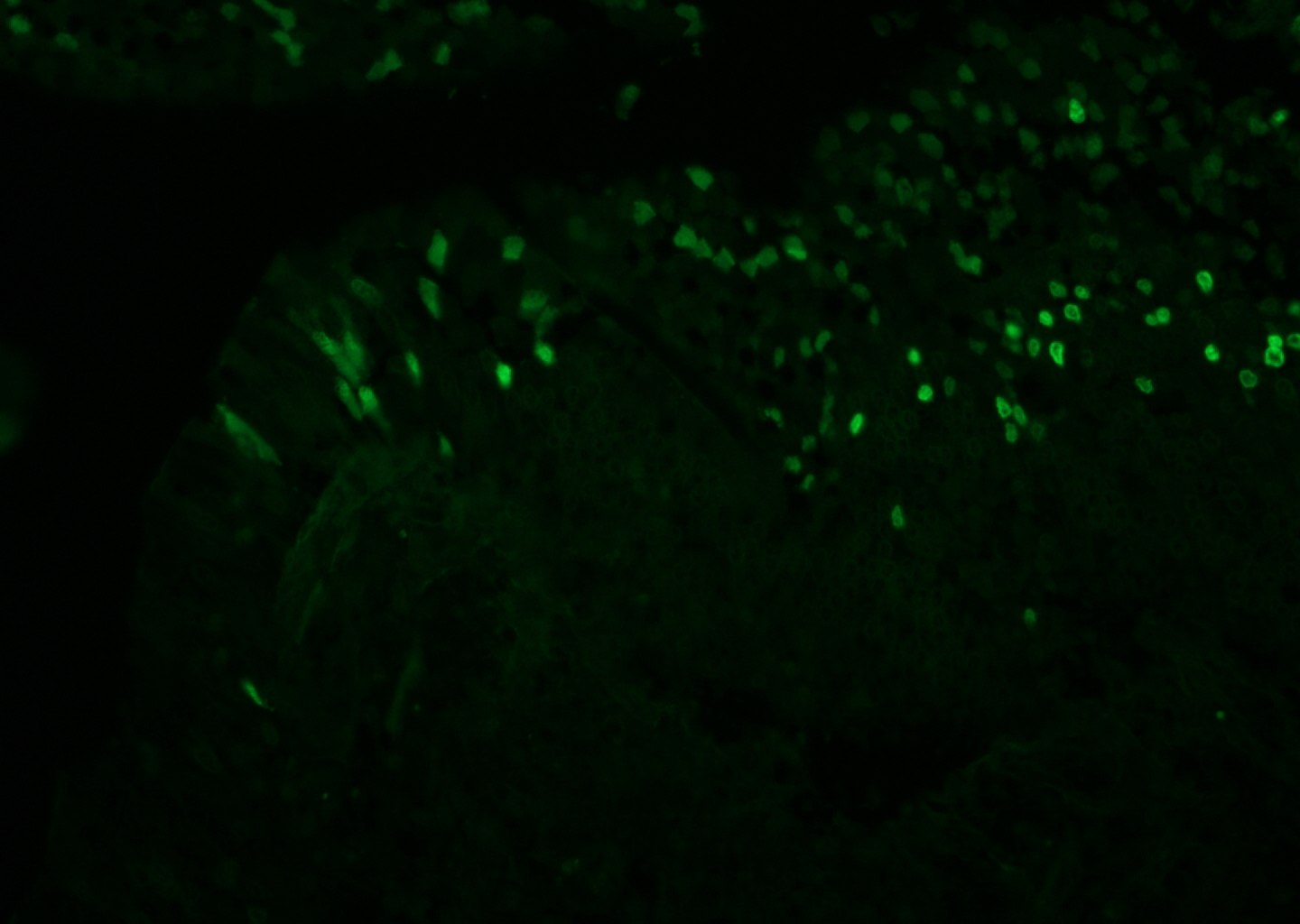

Supplement: Supplementary file 8 [file Data_Sheet_3.ZIP › NE group-Jejunal TUNEL apoptosis/400 x/NE-1 400-1.jpg]

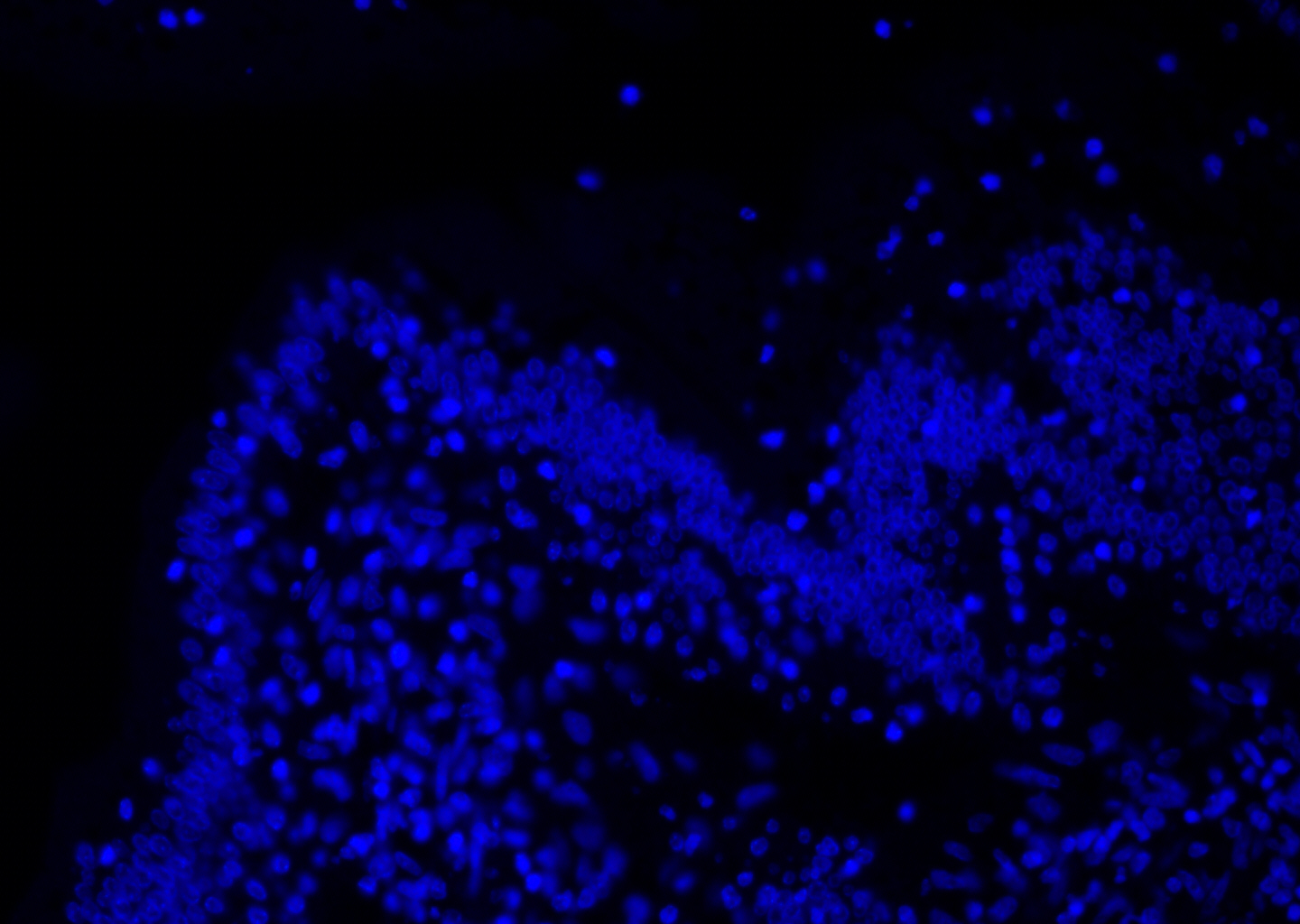

Supplement: Supplementary file 8 [file Data_Sheet_3.ZIP › NE group-Jejunal TUNEL apoptosis/400 x/NE-1 400-2.jpg]

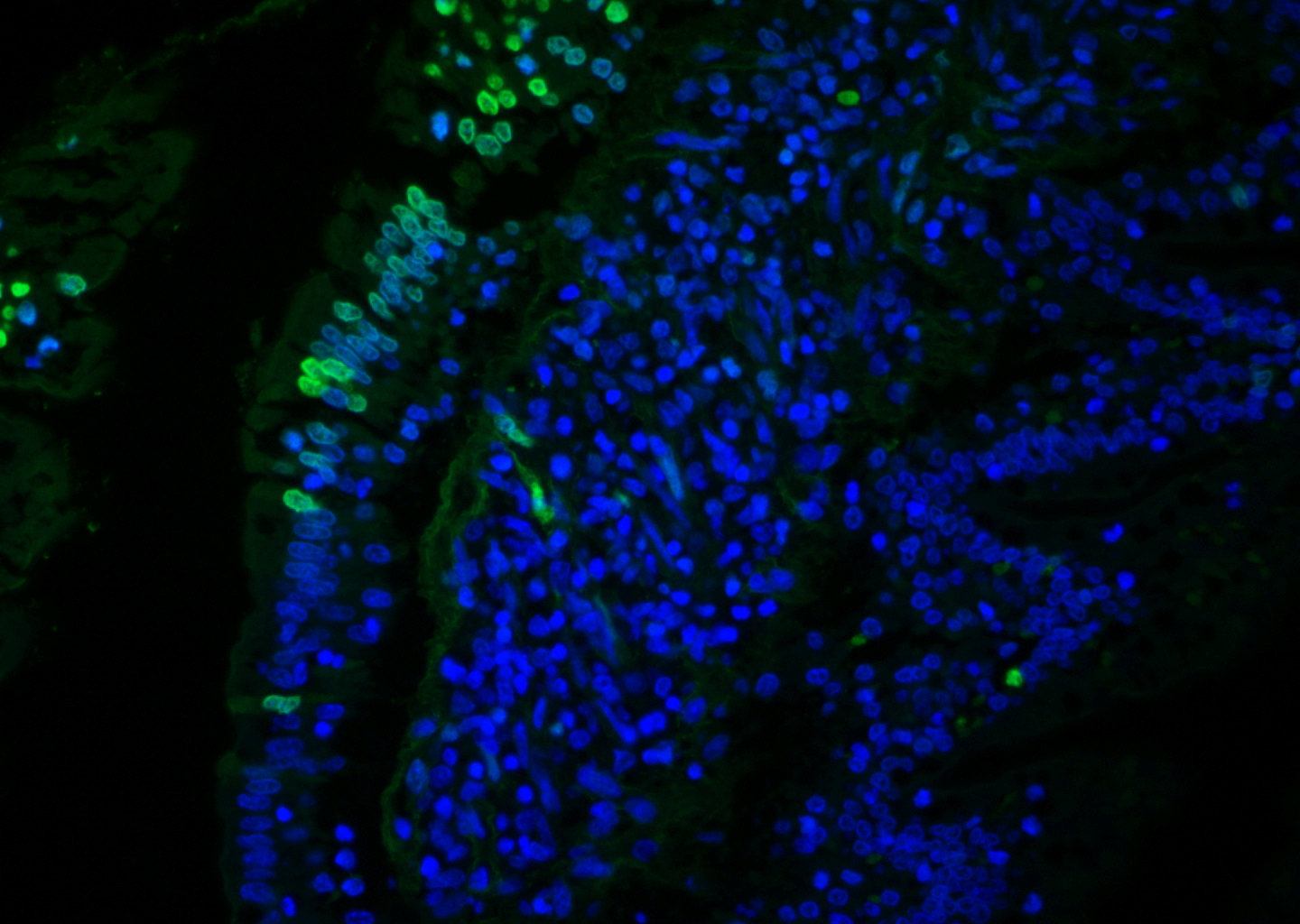

Supplement: Supplementary file 8 [file Data_Sheet_3.ZIP › NE group-Jejunal TUNEL apoptosis/400 x/NE-1 400-3 4.jpg]

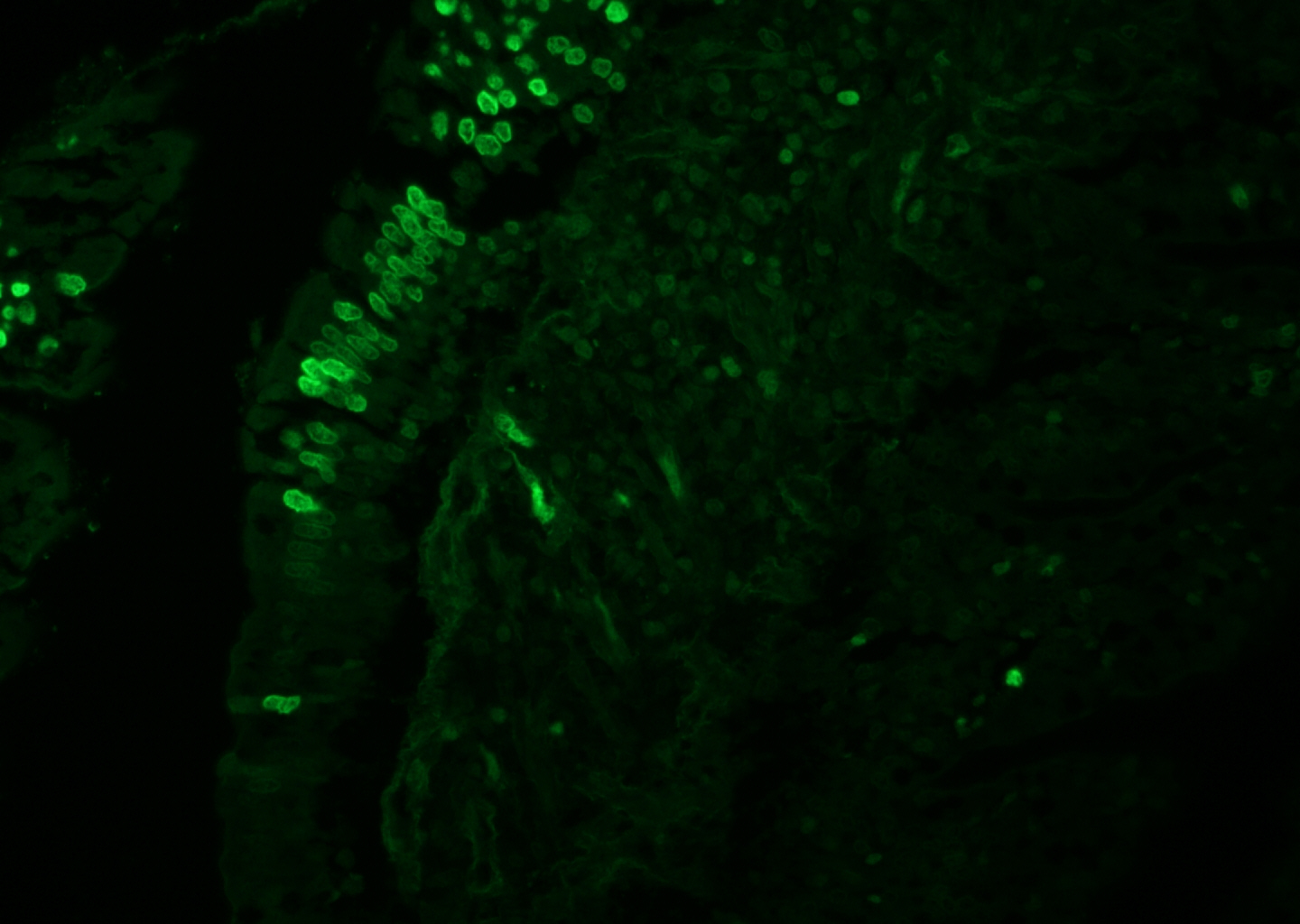

Supplement: Supplementary file 8 [file Data_Sheet_3.ZIP › NE group-Jejunal TUNEL apoptosis/400 x/NE-1 400-3.jpg]

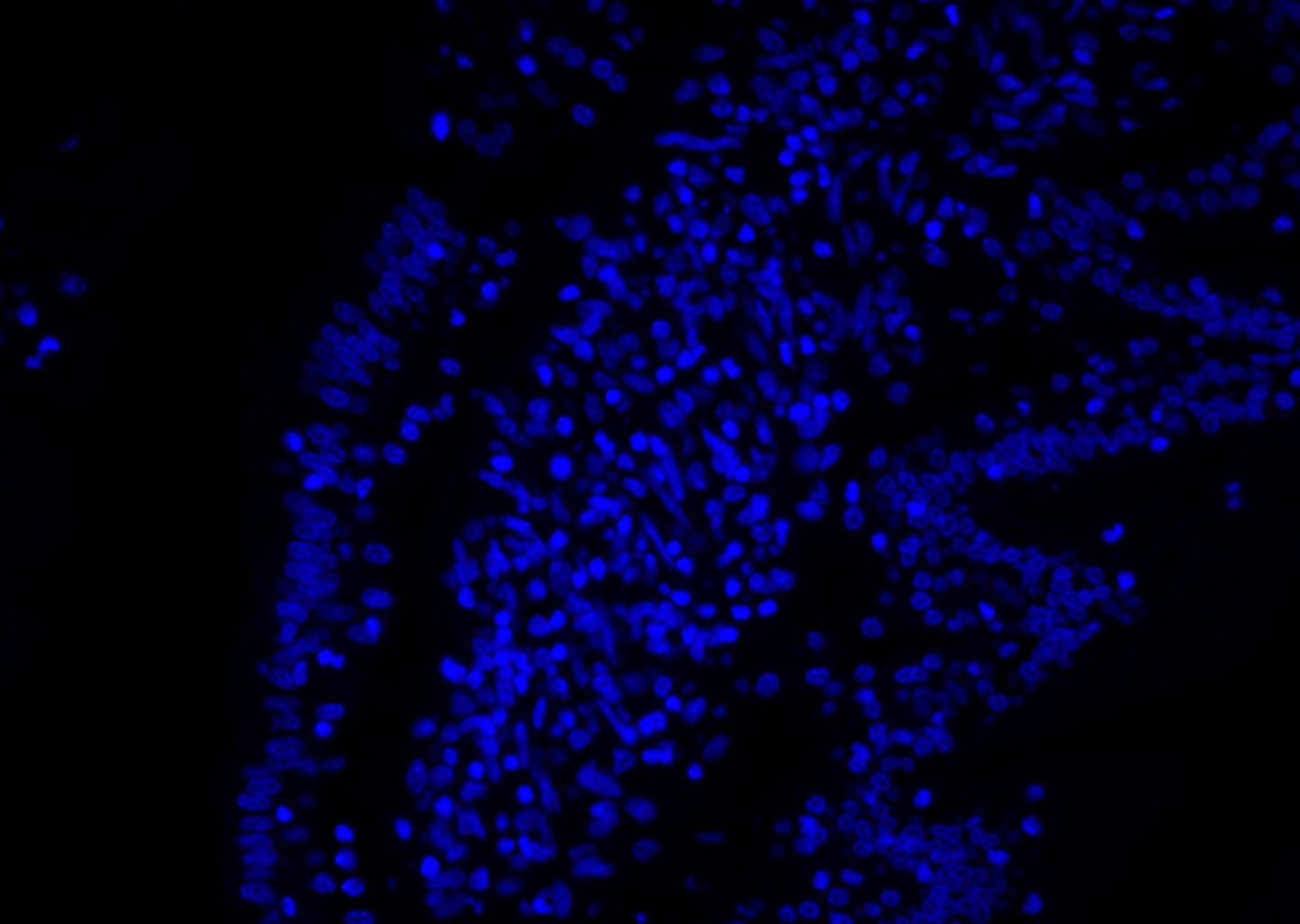

Supplement: Supplementary file 8 [file Data_Sheet_3.ZIP › NE group-Jejunal TUNEL apoptosis/400 x/NE-1 400-4.jpg]

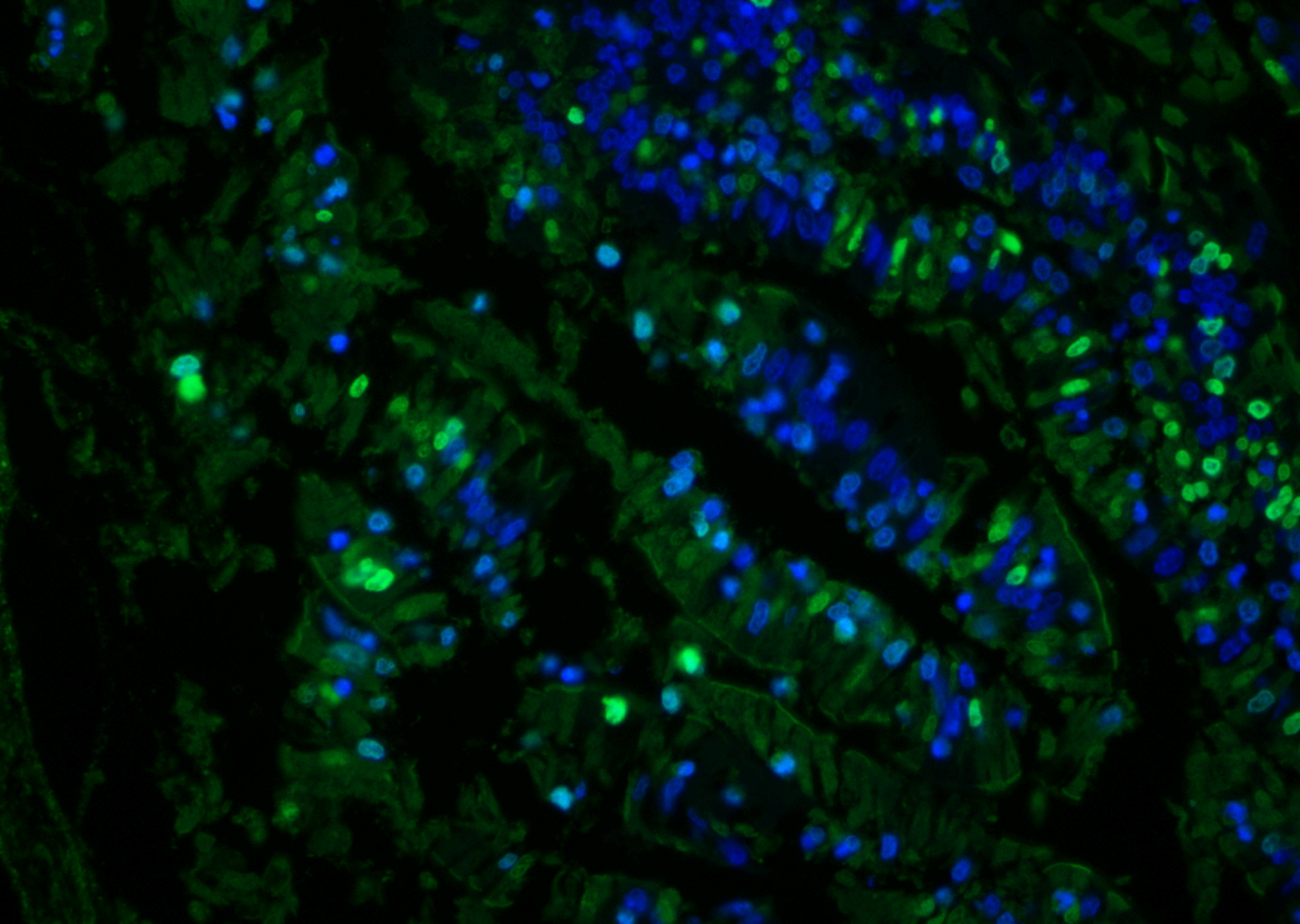

Supplement: Supplementary file 8 [file Data_Sheet_3.ZIP › NE group-Jejunal TUNEL apoptosis/400 x/NE-1 400-5 6.jpg]

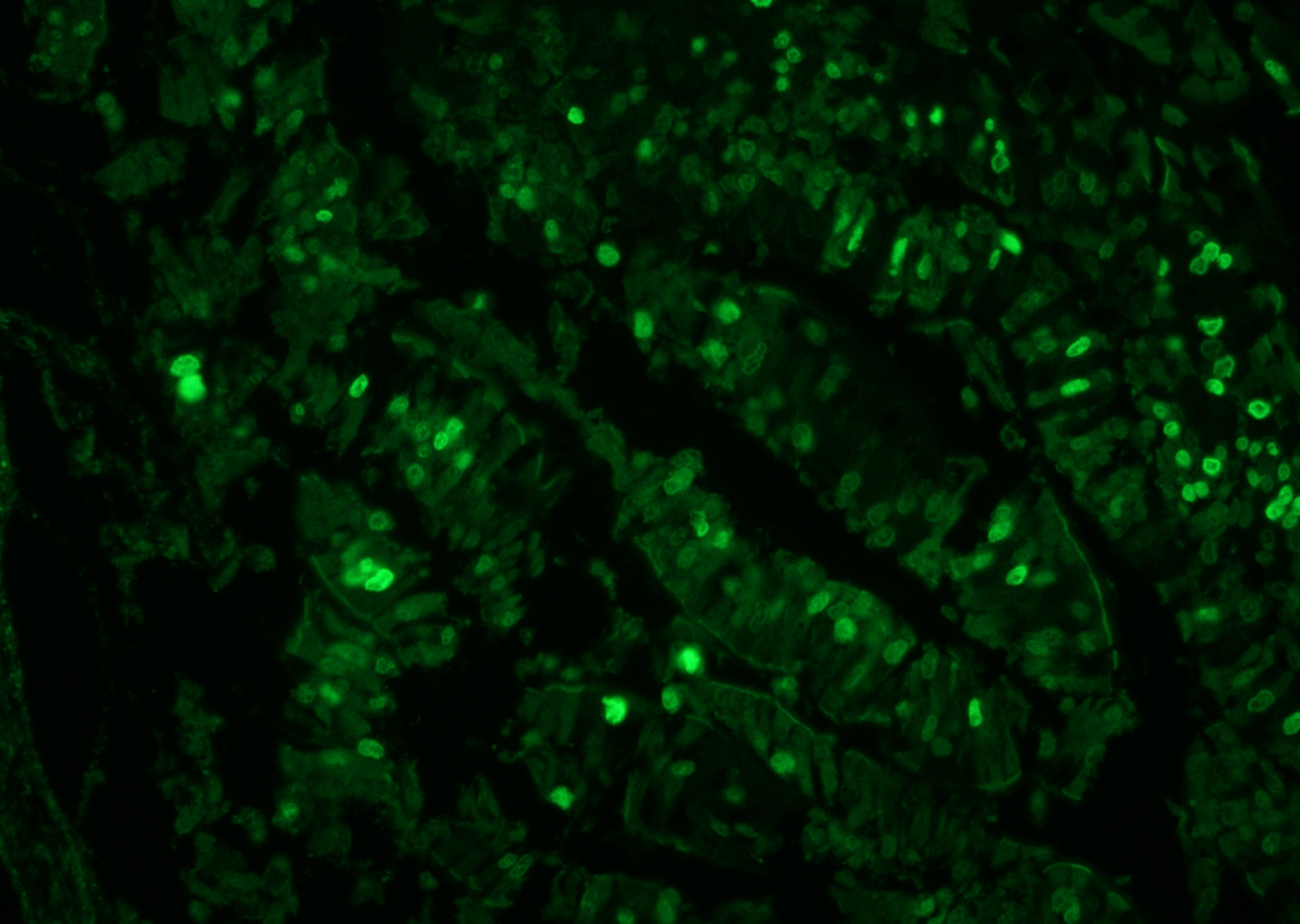

Supplement: Supplementary file 8 [file Data_Sheet_3.ZIP › NE group-Jejunal TUNEL apoptosis/400 x/NE-1 400-5.jpg]

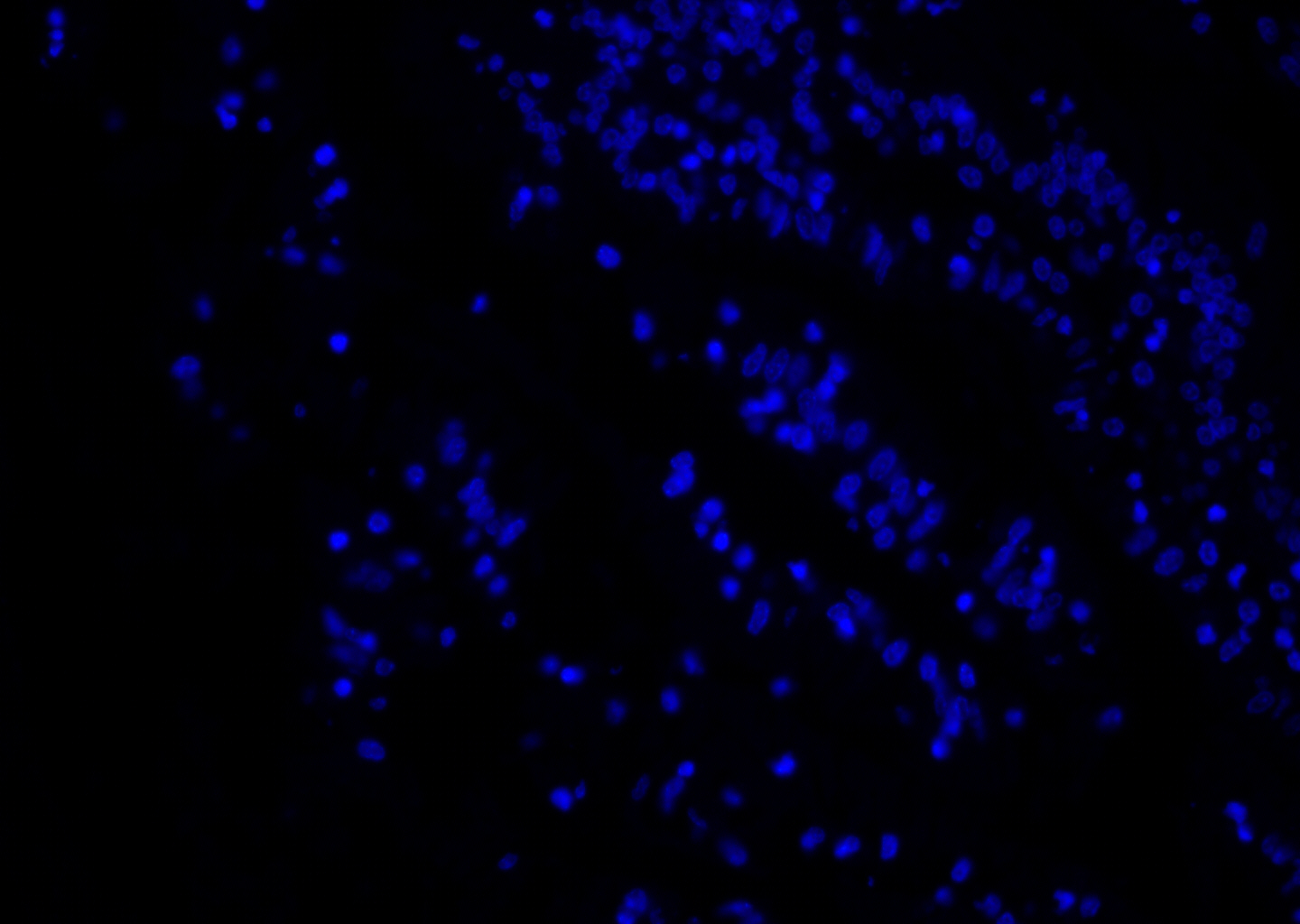

Supplement: Supplementary file 8 [file Data_Sheet_3.ZIP › NE group-Jejunal TUNEL apoptosis/400 x/NE-1 400-6.jpg]

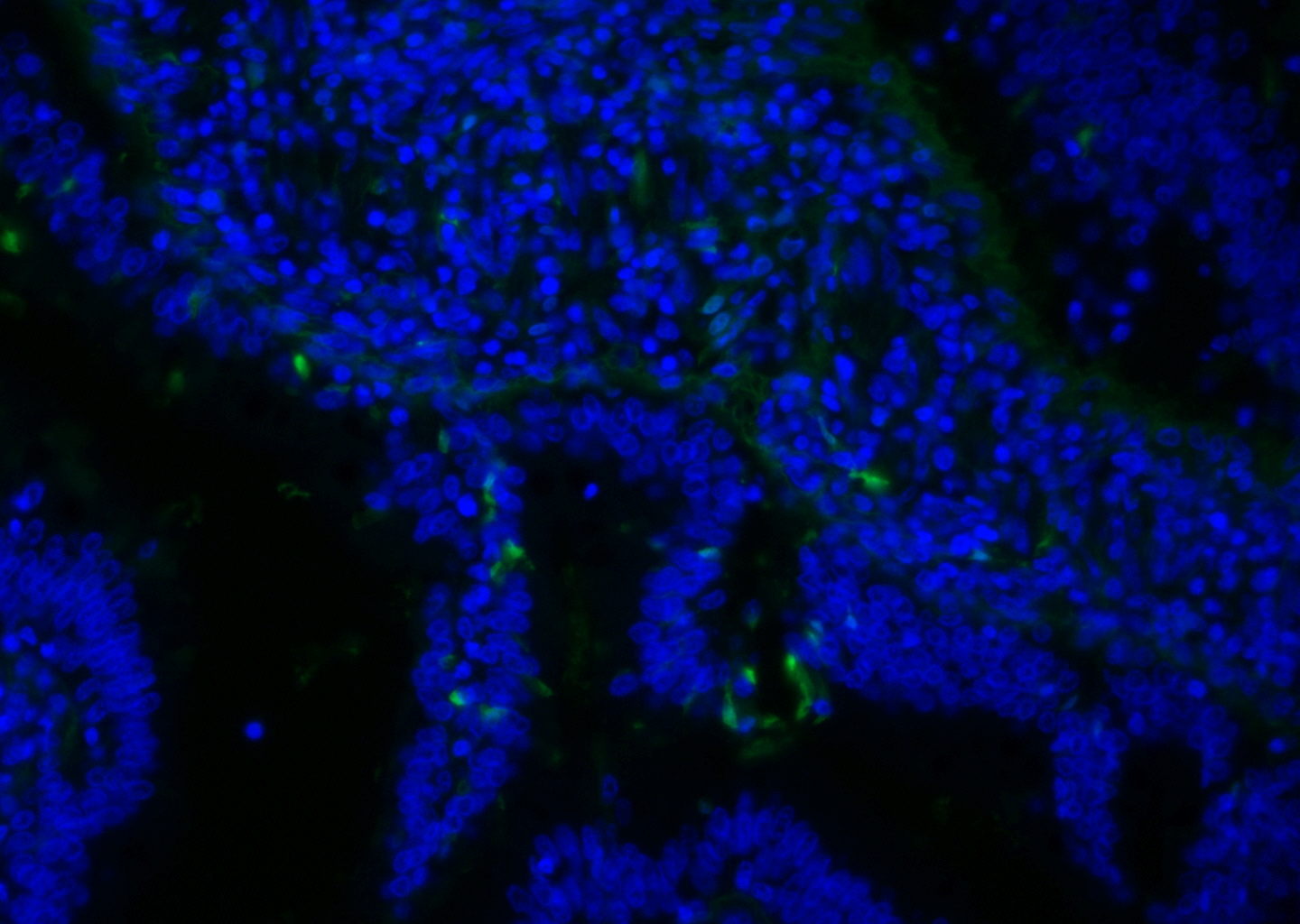

Supplement: Supplementary file 8 [file Data_Sheet_3.ZIP › NE group-Jejunal TUNEL apoptosis/400 x/NE-2 400-1 2.jpg]

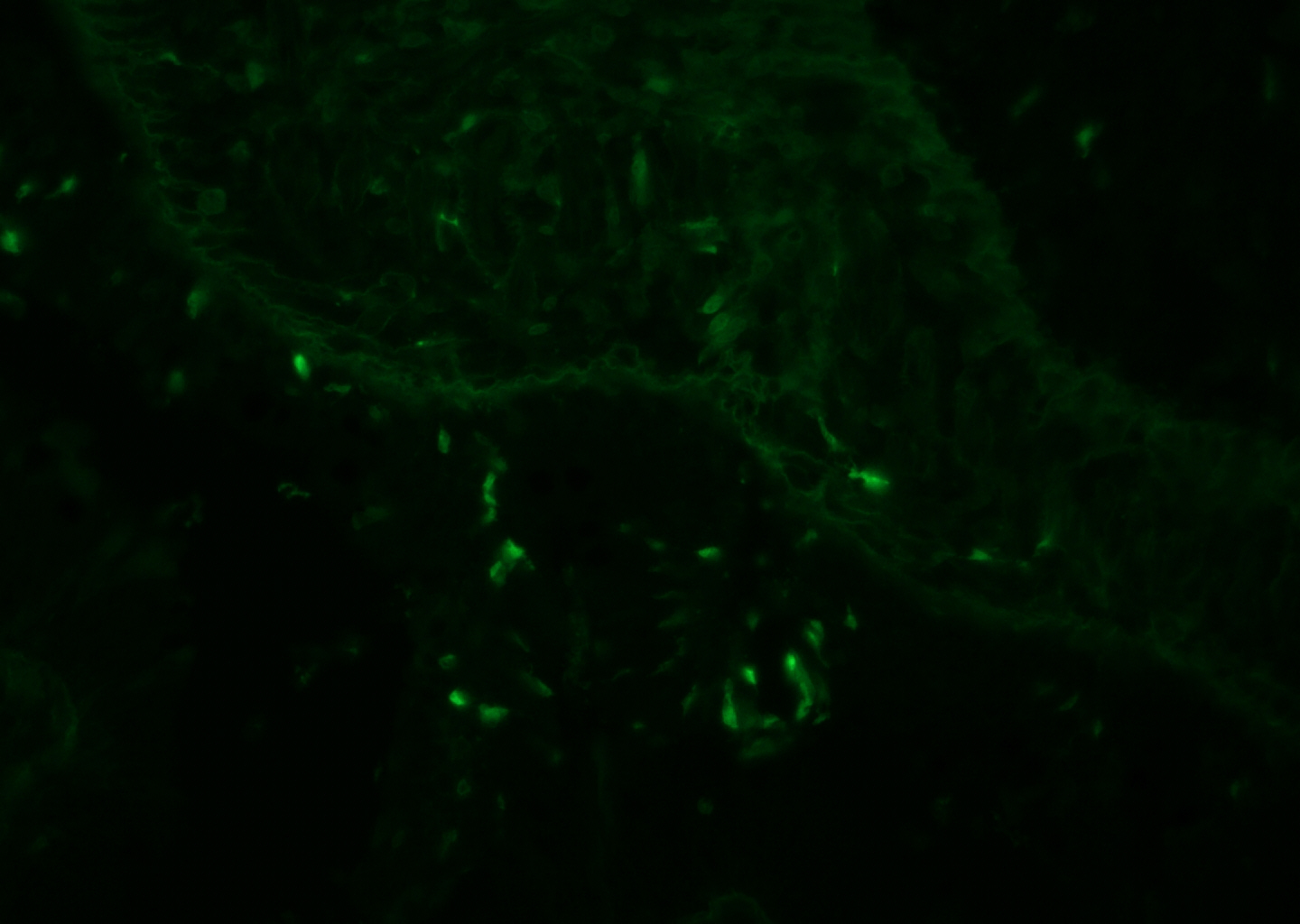

Supplement: Supplementary file 8 [file Data_Sheet_3.ZIP › NE group-Jejunal TUNEL apoptosis/400 x/NE-2 400-1.jpg]

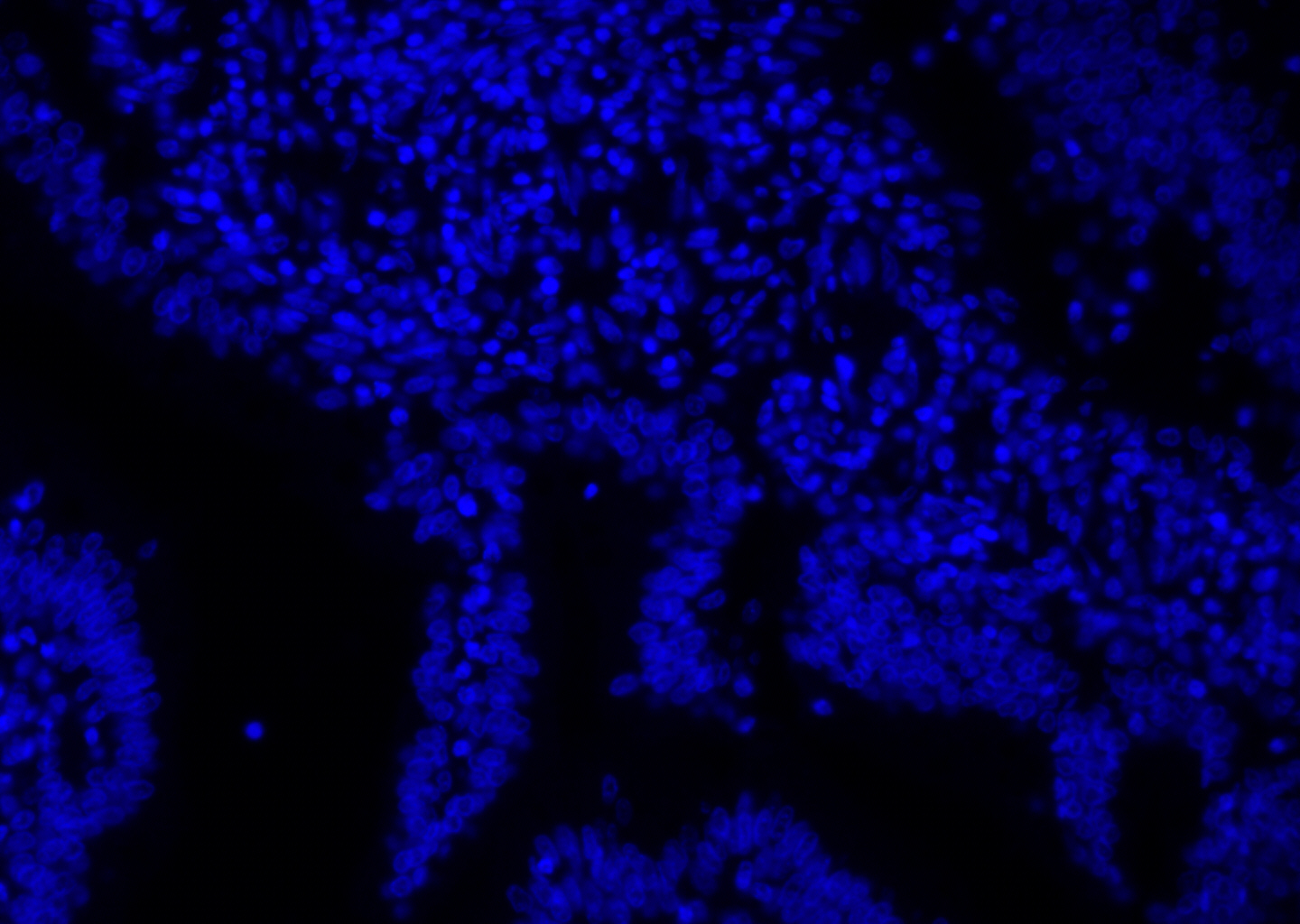

Supplement: Supplementary file 8 [file Data_Sheet_3.ZIP › NE group-Jejunal TUNEL apoptosis/400 x/NE-2 400-2.jpg]

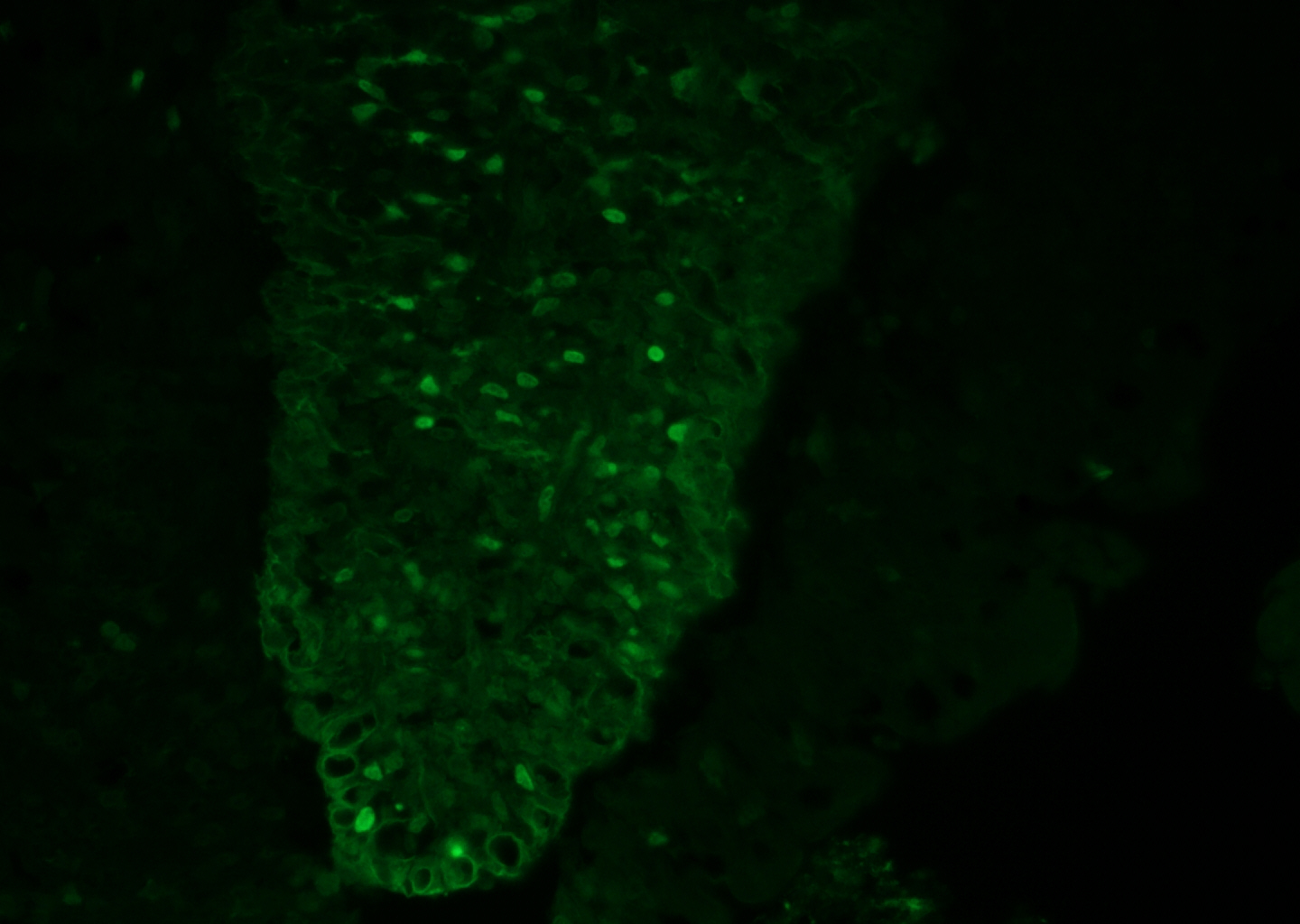

Supplement: Supplementary file 8 [file Data_Sheet_3.ZIP › NE group-Jejunal TUNEL apoptosis/400 x/NE-2 400-3.jpg]

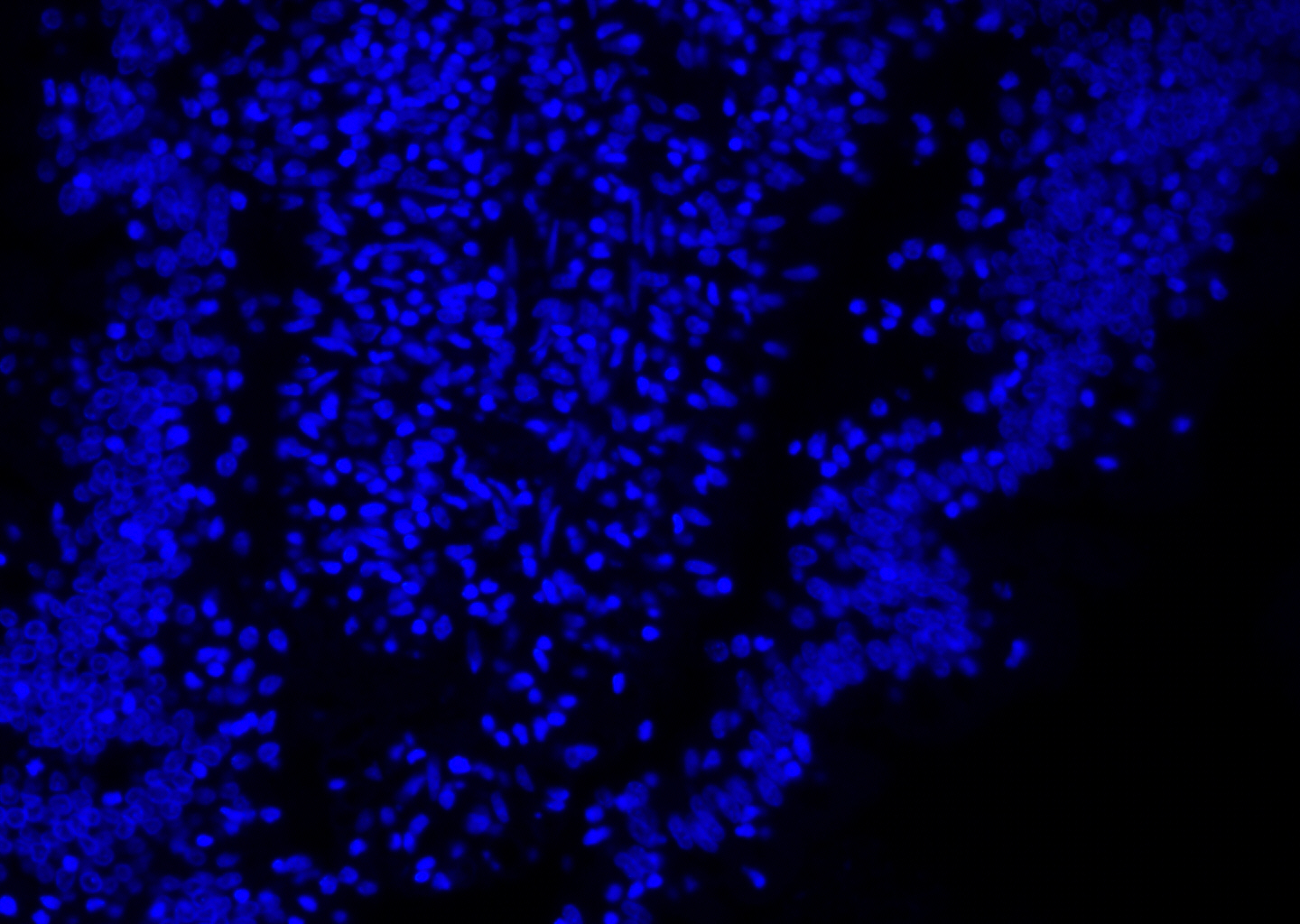

Supplement: Supplementary file 8 [file Data_Sheet_3.ZIP › NE group-Jejunal TUNEL apoptosis/400 x/NE-2 400-4.jpg]

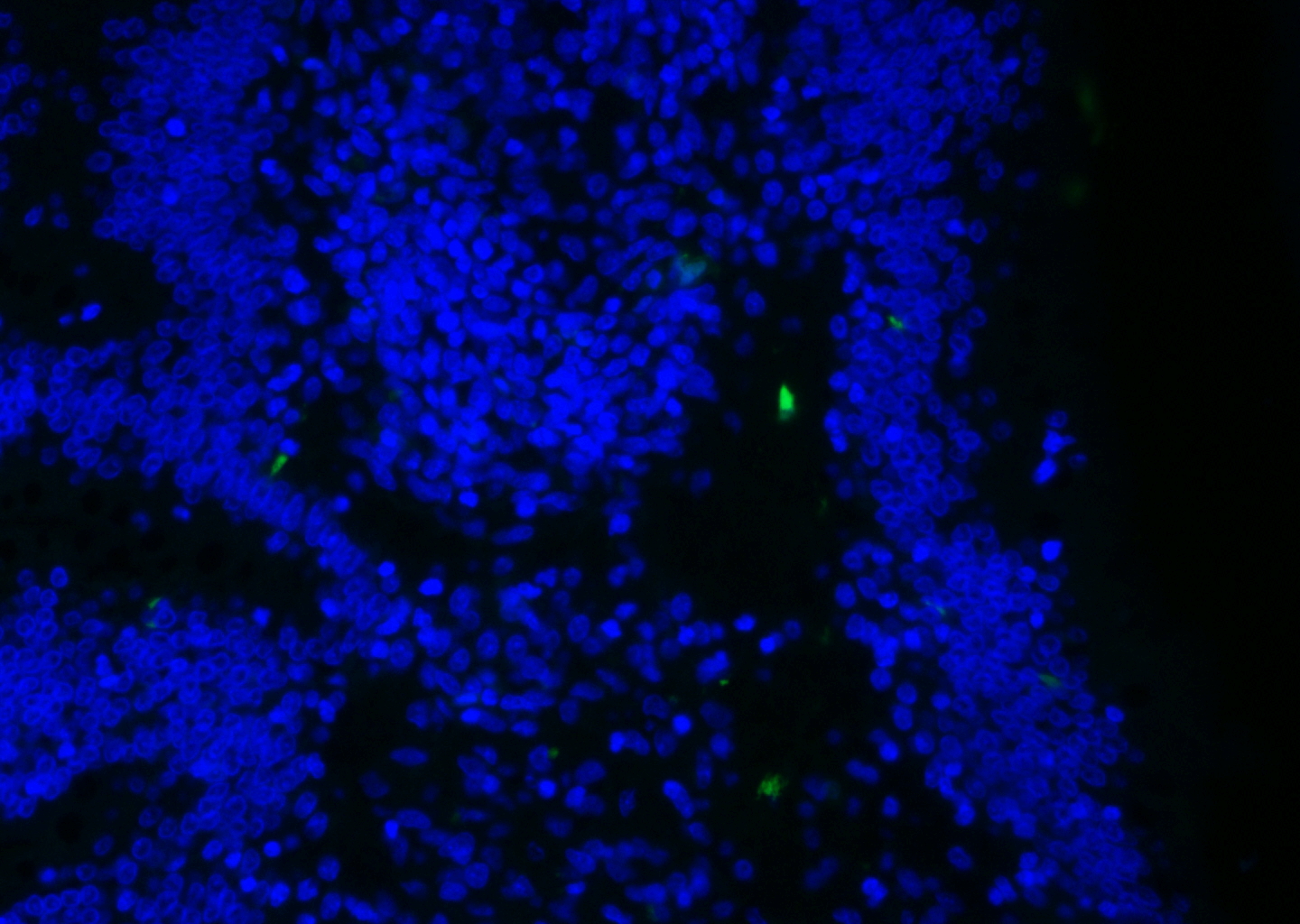

Supplement: Supplementary file 8 [file Data_Sheet_3.ZIP › NE group-Jejunal TUNEL apoptosis/400 x/NE-2 400-5 6.jpg]

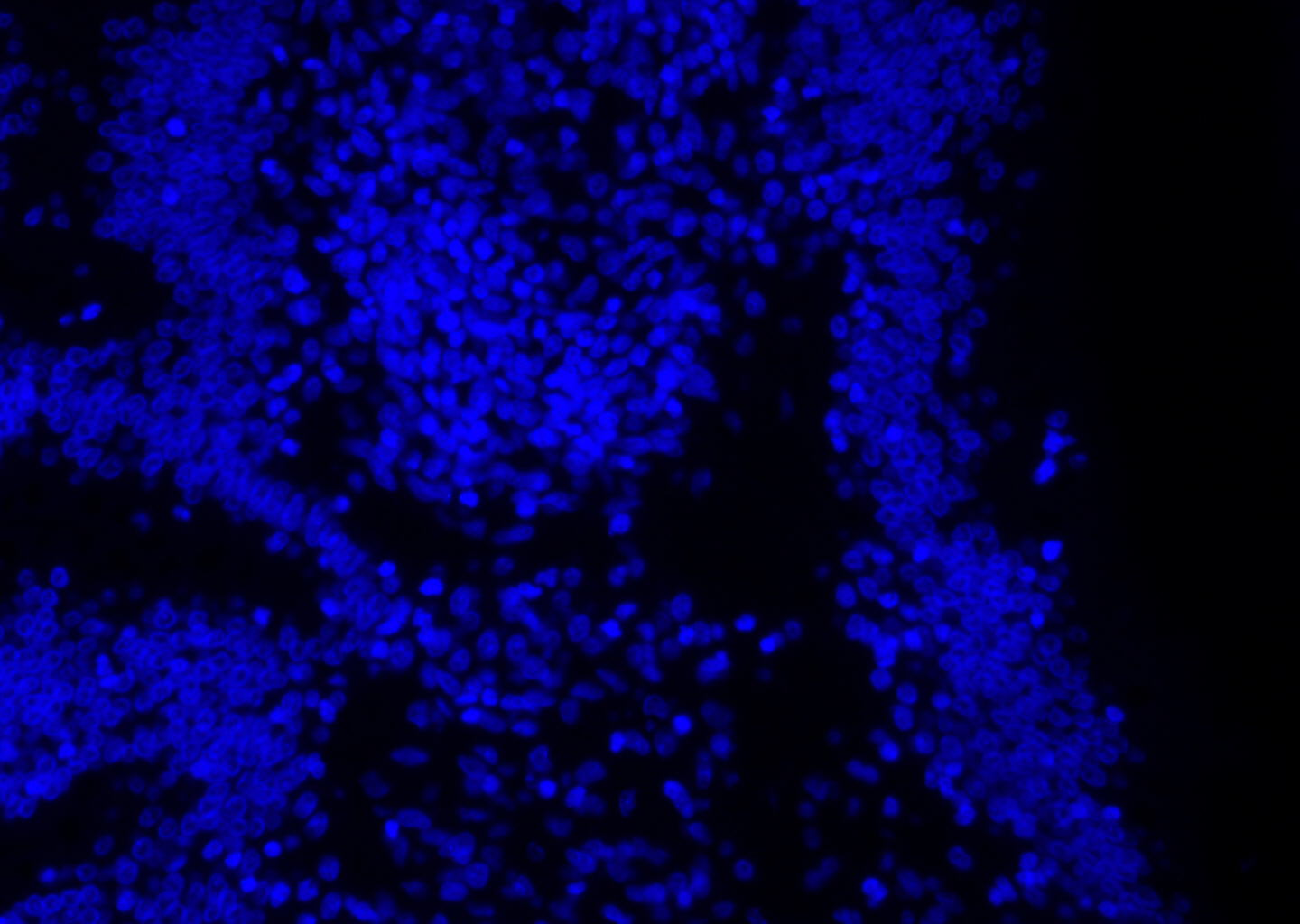

Supplement: Supplementary file 8 [file Data_Sheet_3.ZIP › NE group-Jejunal TUNEL apoptosis/400 x/NE-2 400-6.jpg]

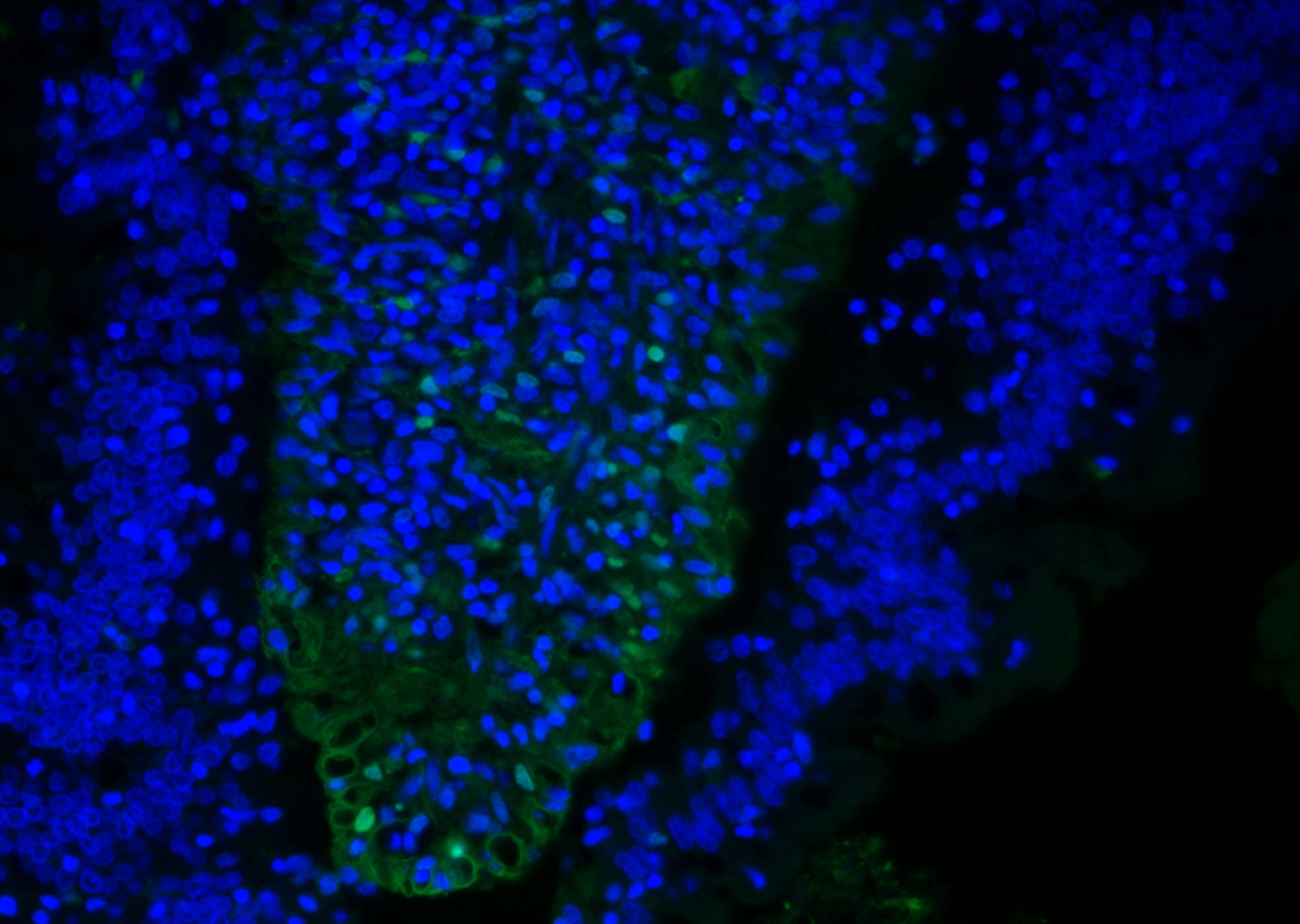

Supplement: Supplementary file 8 [file Data_Sheet_3.ZIP › NE group-Jejunal TUNEL apoptosis/400 x/NE-2 400-3 4.jpg]

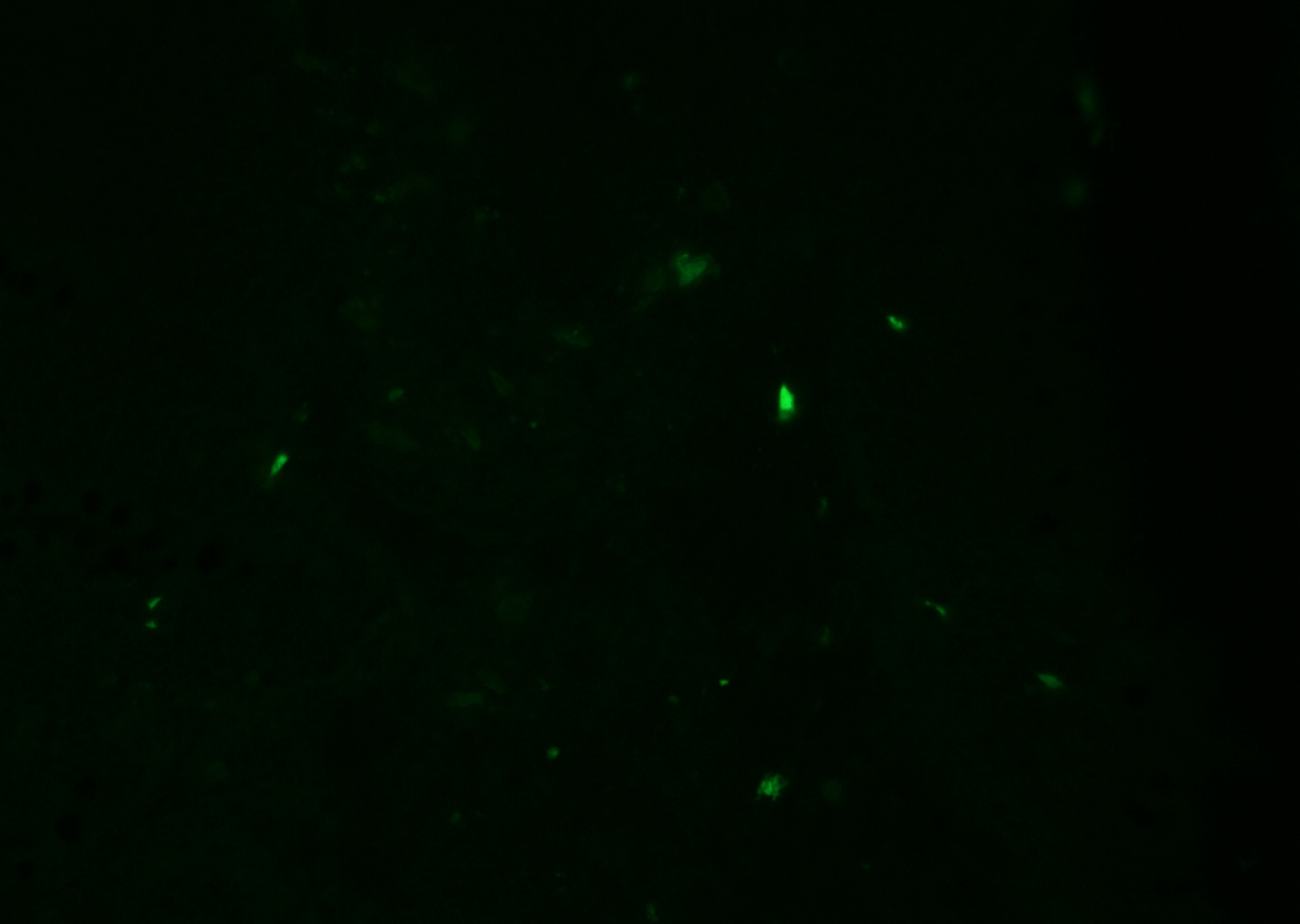

Supplement: Supplementary file 8 [file Data_Sheet_3.ZIP › NE group-Jejunal TUNEL apoptosis/400 x/NE-2 400-5.jpg]

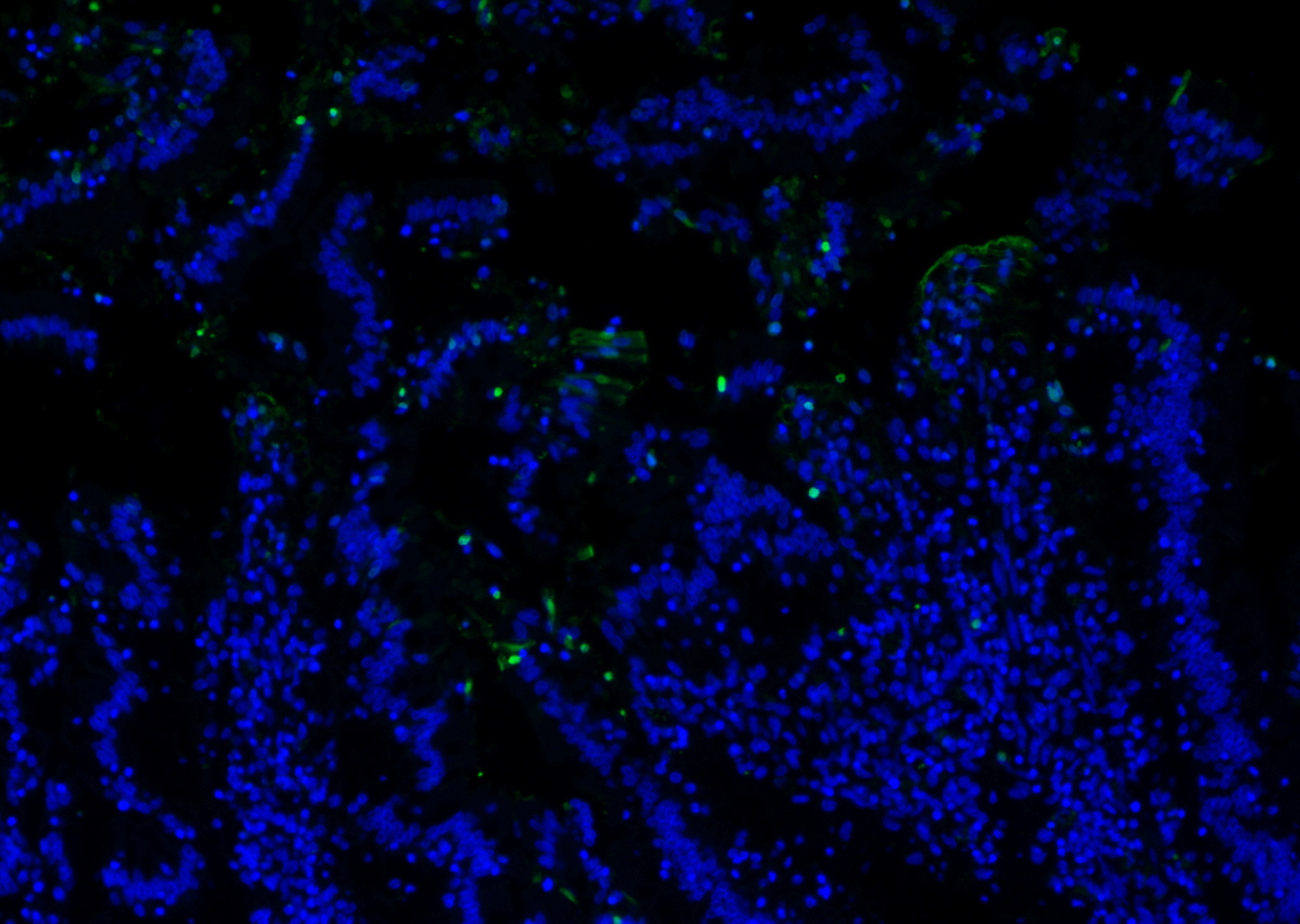

Supplement: Supplementary file 9 [file Data_Sheet_4.ZIP › NE+TA400 group-Jejunal TUNEL apoptosis/200 x/NE+TA400-1 200-1 2.jpg]

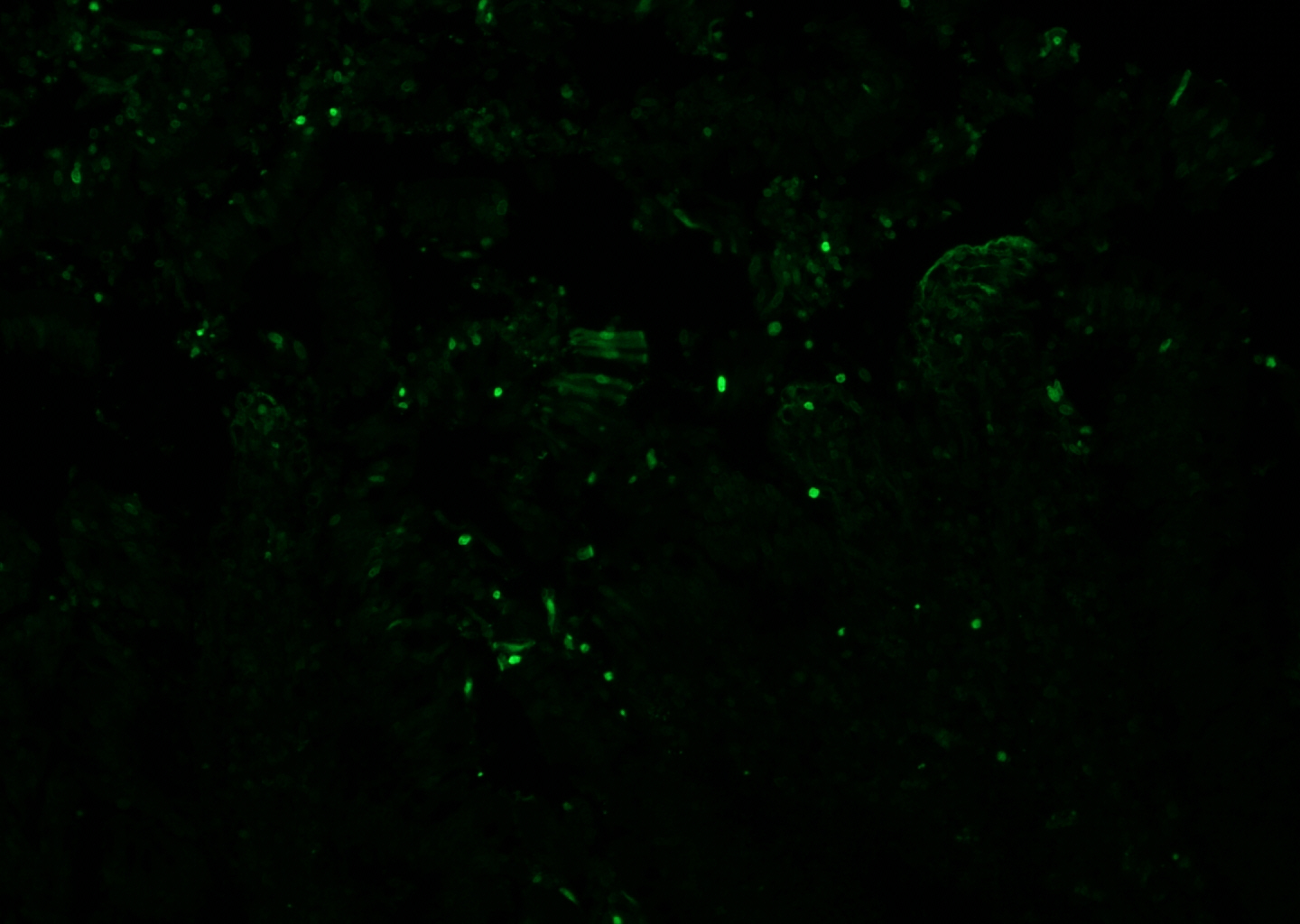

Supplement: Supplementary file 9 [file Data_Sheet_4.ZIP › NE+TA400 group-Jejunal TUNEL apoptosis/200 x/NE+TA400-1 200-1.jpg]

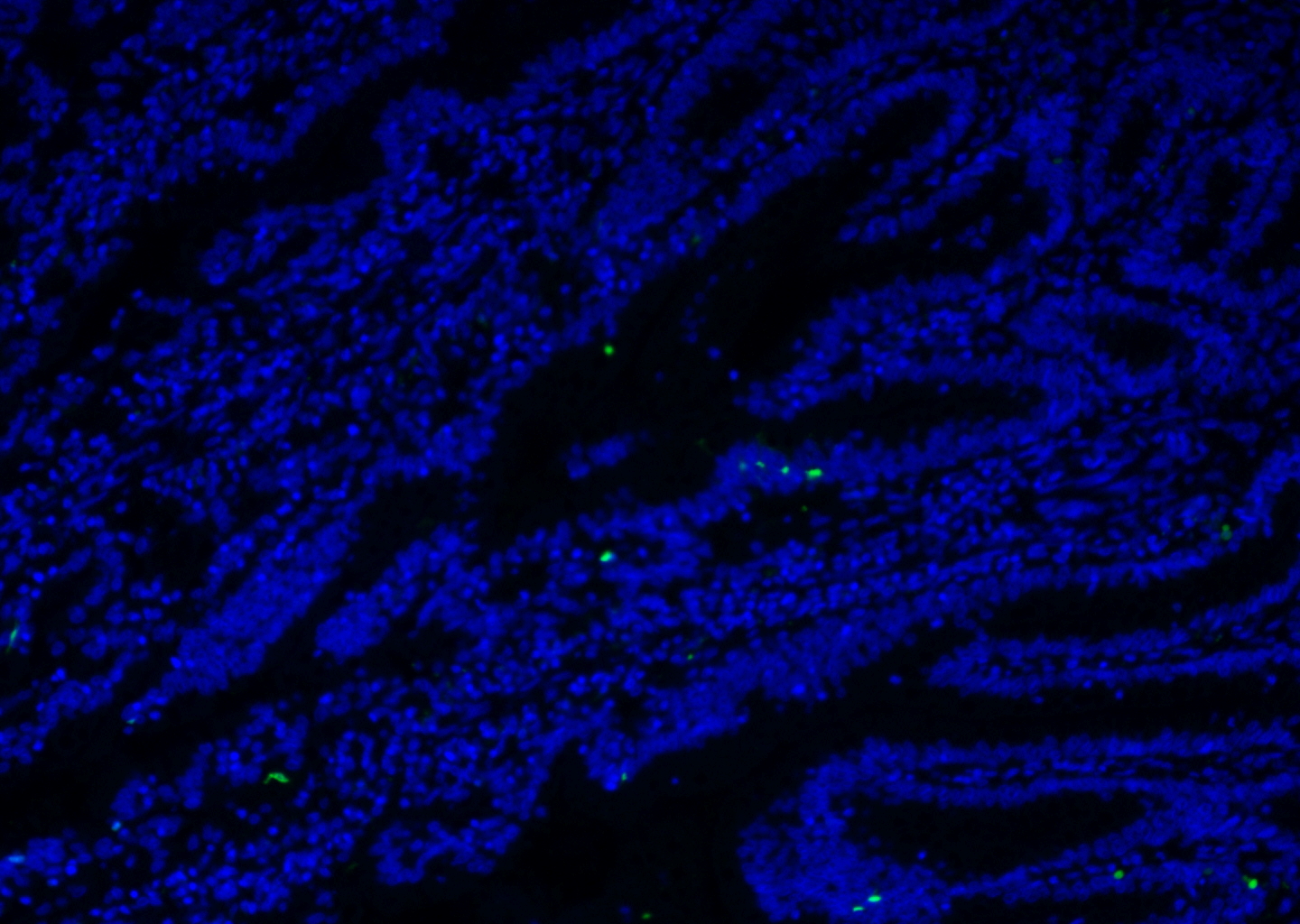

Supplement: Supplementary file 9 [file Data_Sheet_4.ZIP › NE+TA400 group-Jejunal TUNEL apoptosis/200 x/NE+TA400-1 200-3 4.jpg]

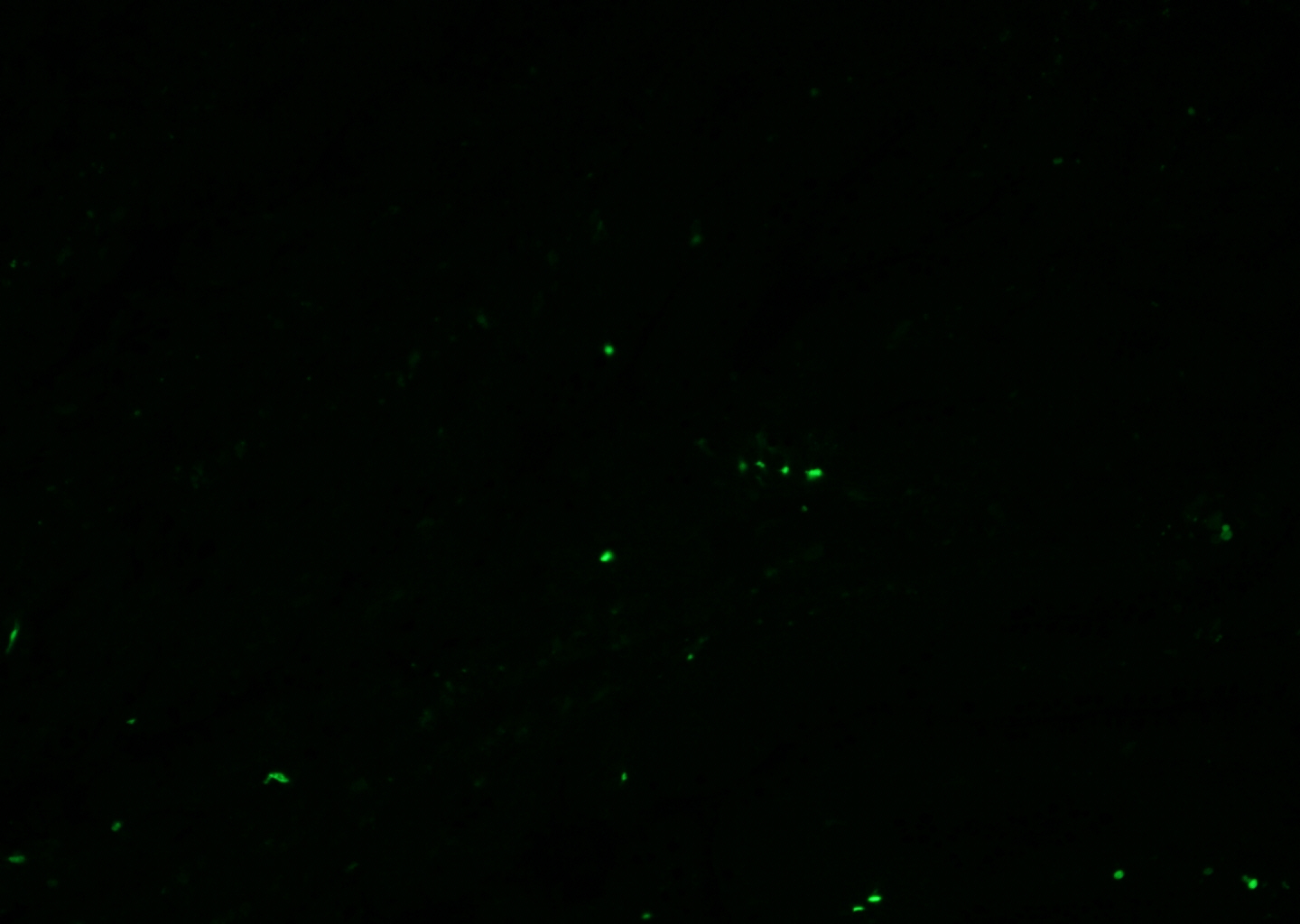

Supplement: Supplementary file 9 [file Data_Sheet_4.ZIP › NE+TA400 group-Jejunal TUNEL apoptosis/200 x/NE+TA400-1 200-3.jpg]

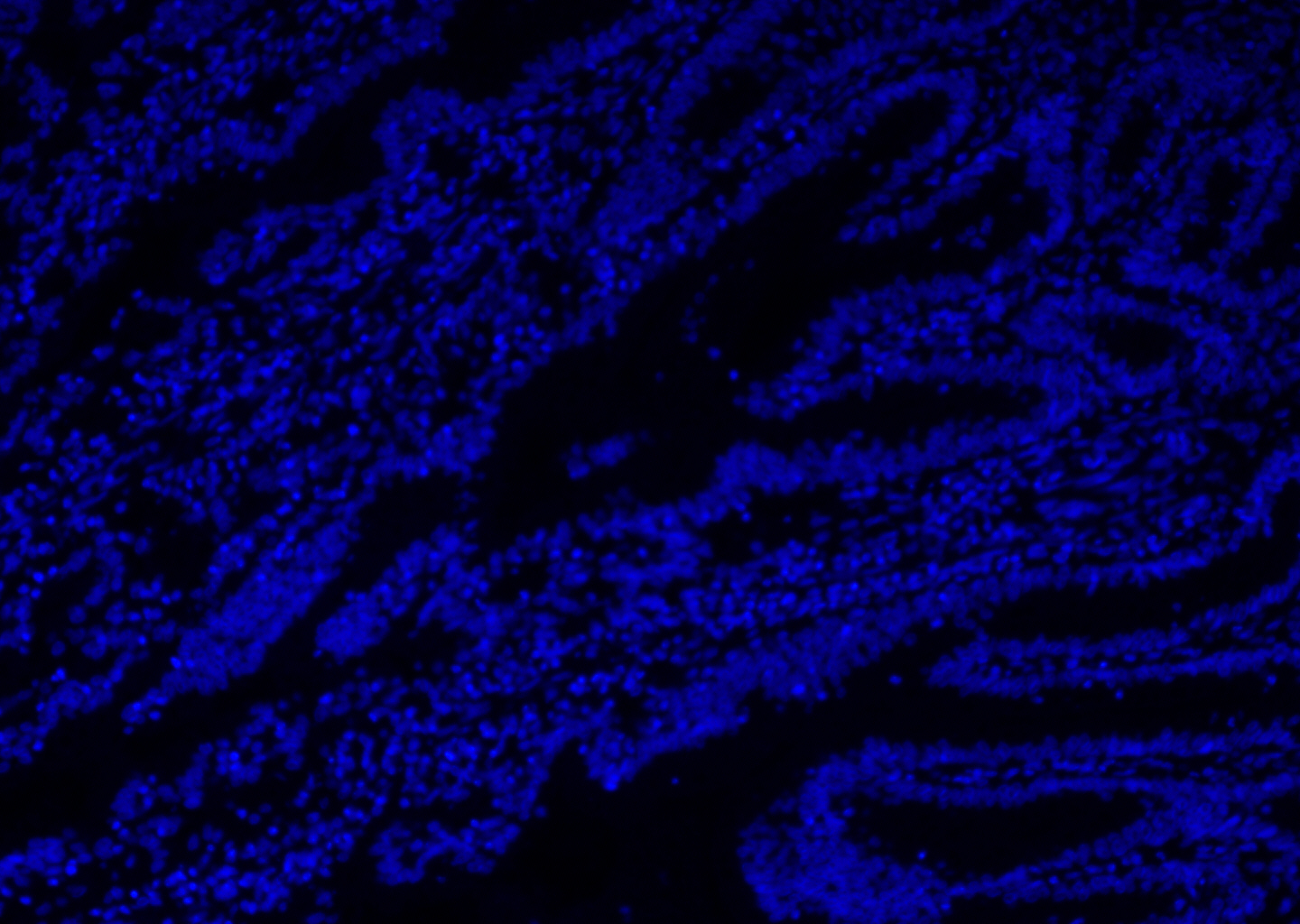

Supplement: Supplementary file 9 [file Data_Sheet_4.ZIP › NE+TA400 group-Jejunal TUNEL apoptosis/200 x/NE+TA400-1 200-4.jpg]

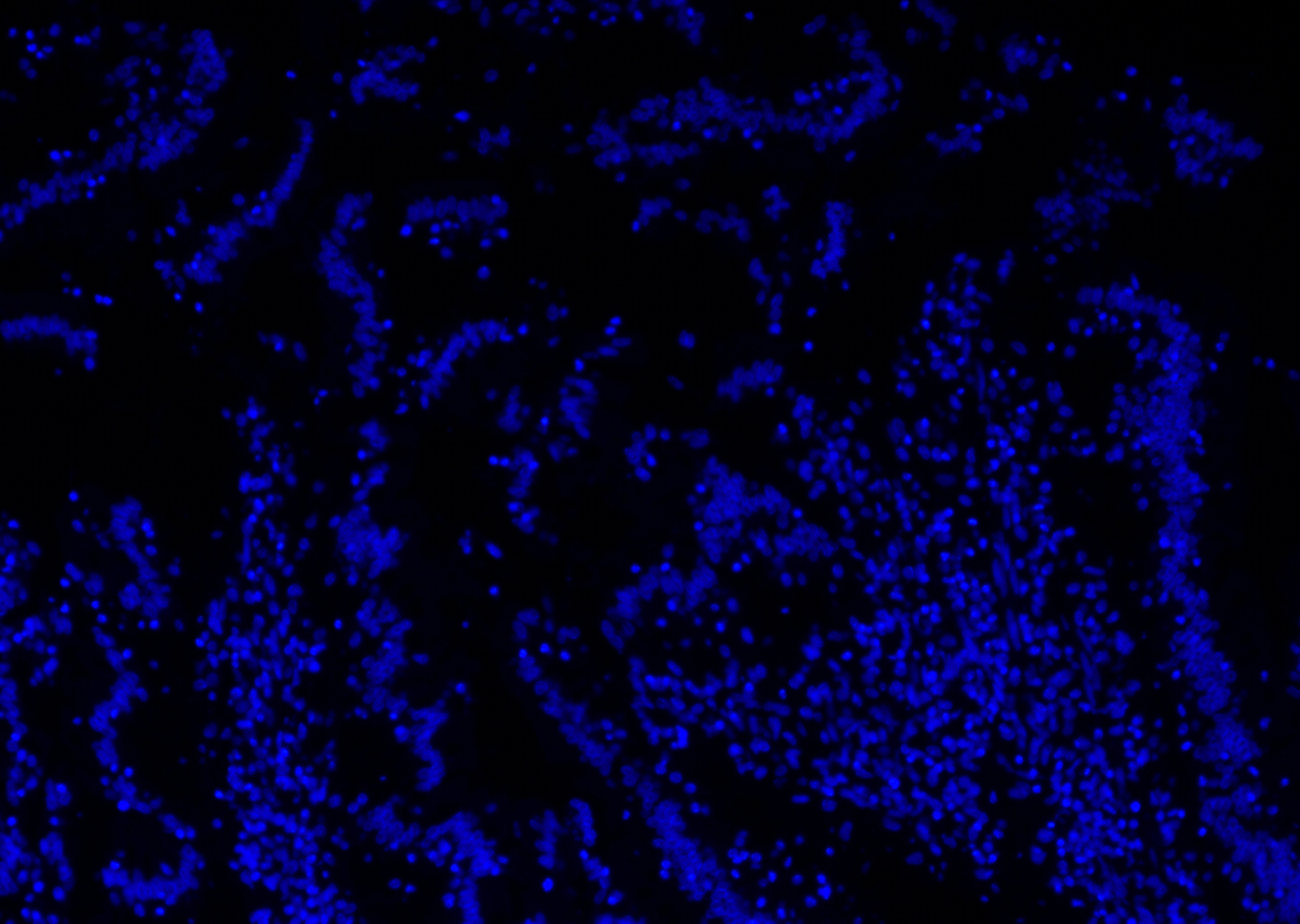

Supplement: Supplementary file 9 [file Data_Sheet_4.ZIP › NE+TA400 group-Jejunal TUNEL apoptosis/200 x/NE+TA400-1 200-2.jpg]

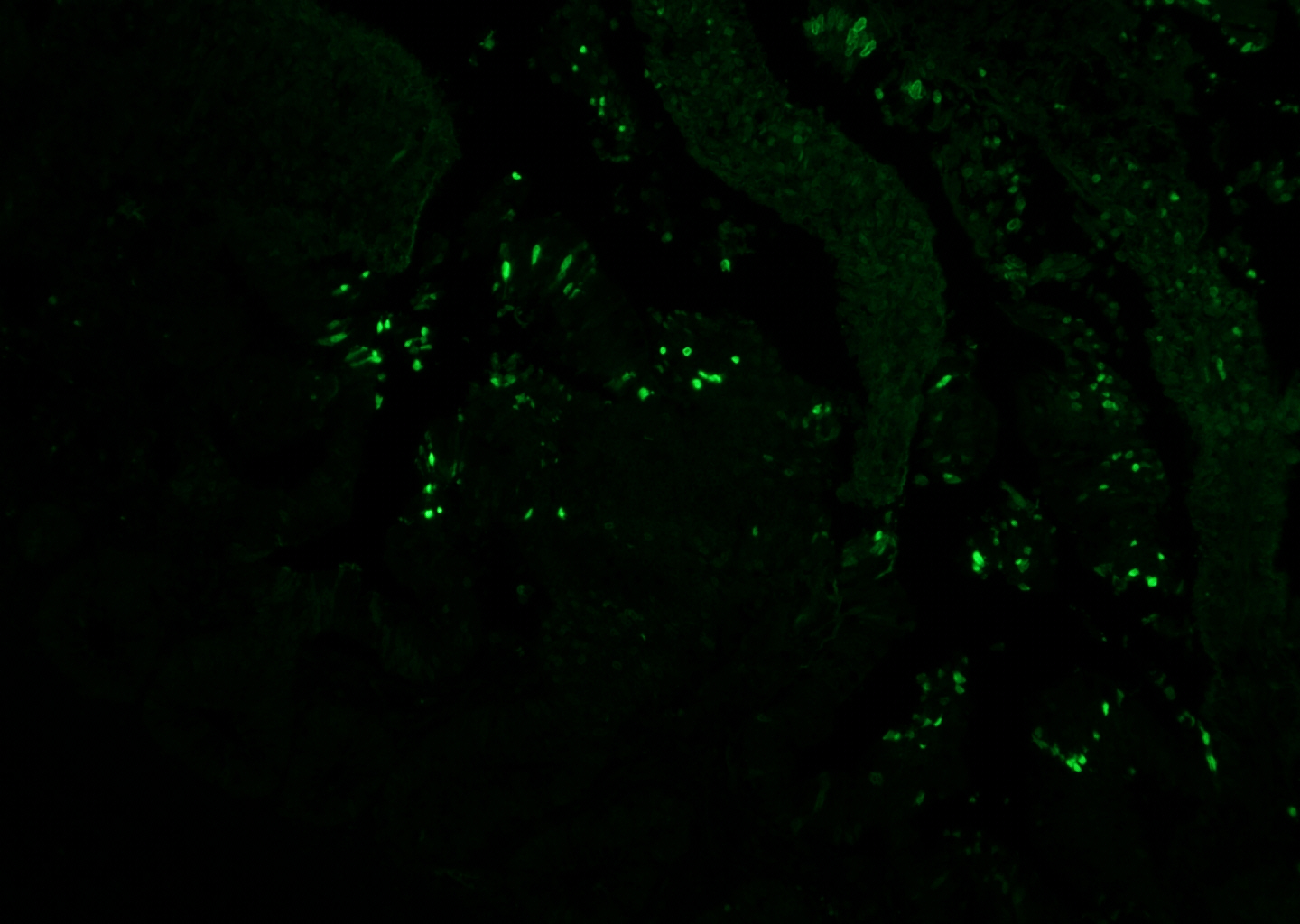

Supplement: Supplementary file 9 [file Data_Sheet_4.ZIP › NE+TA400 group-Jejunal TUNEL apoptosis/200 x/NE+TA400-2 200-1.jpg]

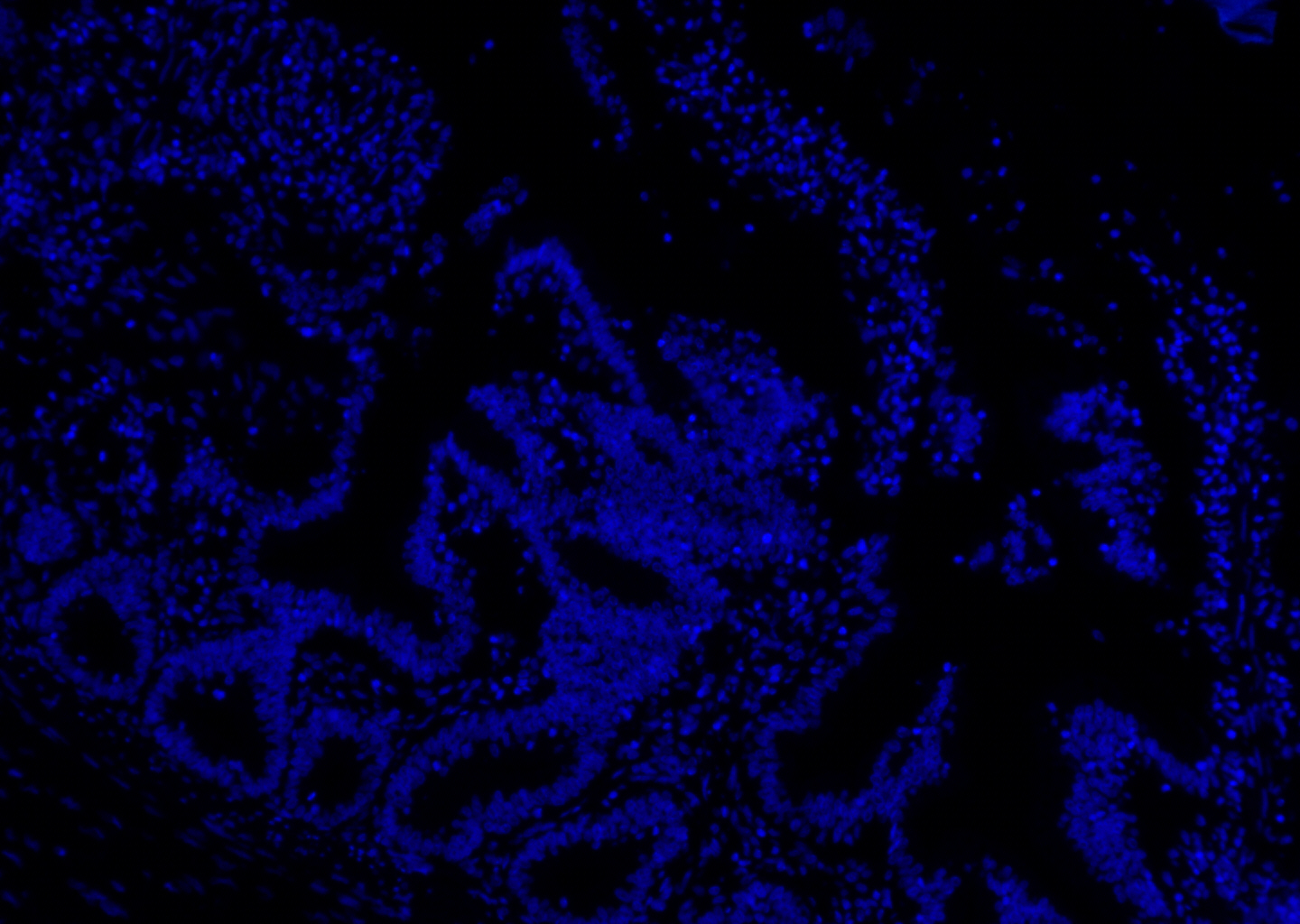

Supplement: Supplementary file 9 [file Data_Sheet_4.ZIP › NE+TA400 group-Jejunal TUNEL apoptosis/200 x/NE+TA400-2 200-2.jpg]

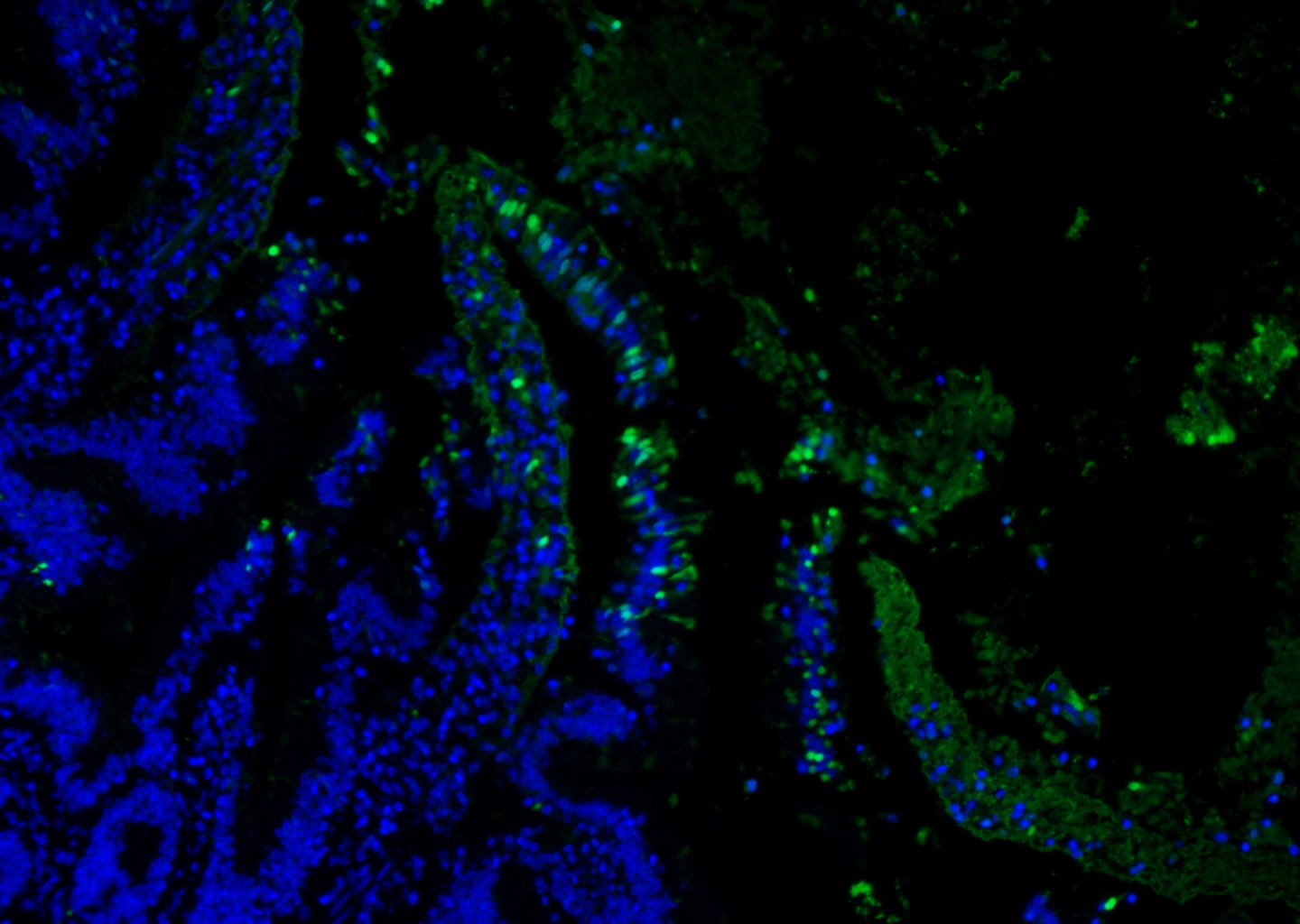

Supplement: Supplementary file 9 [file Data_Sheet_4.ZIP › NE+TA400 group-Jejunal TUNEL apoptosis/200 x/NE+TA400-2 200-3 4.jpg]

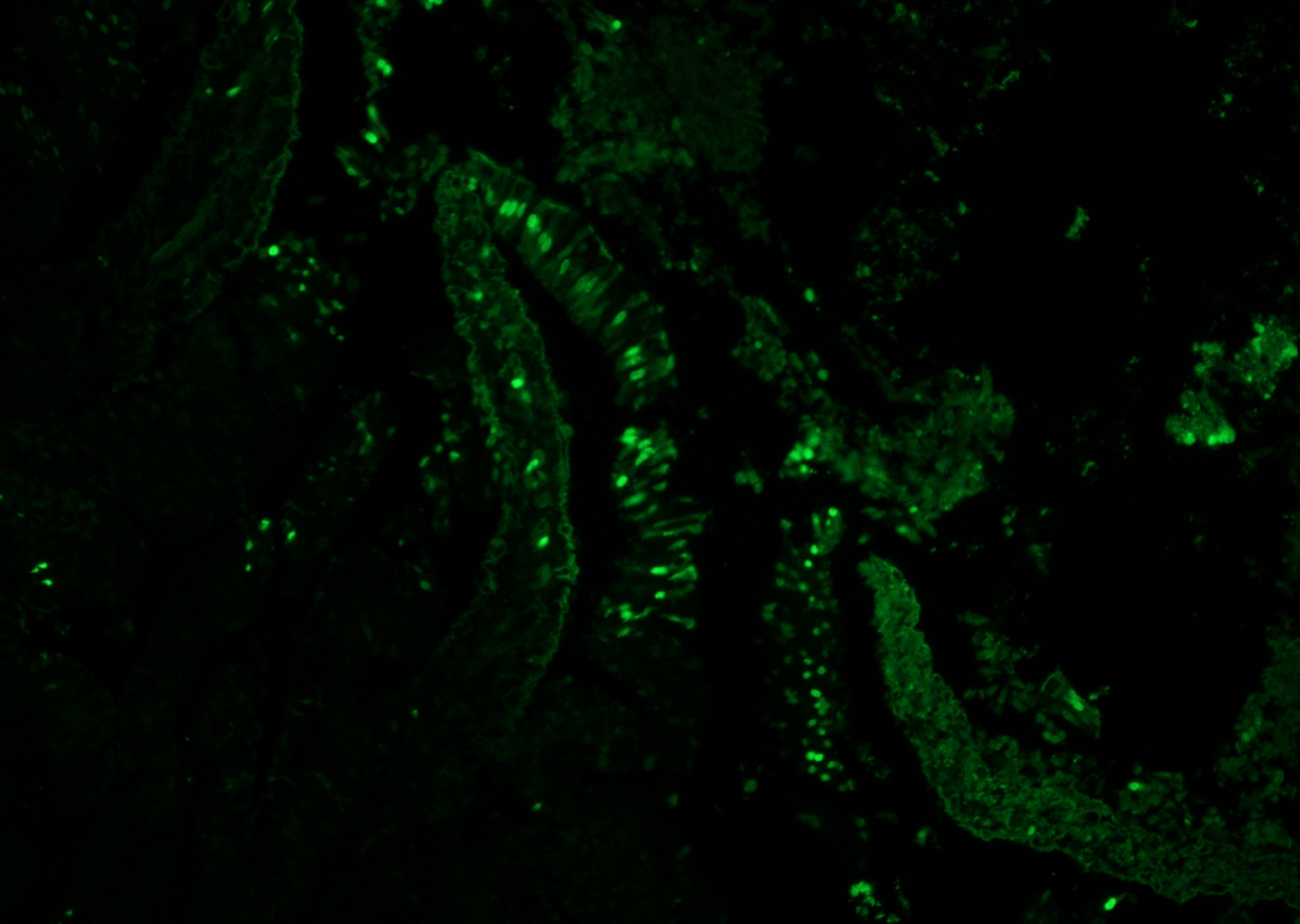

Supplement: Supplementary file 9 [file Data_Sheet_4.ZIP › NE+TA400 group-Jejunal TUNEL apoptosis/200 x/NE+TA400-2 200-3.jpg]

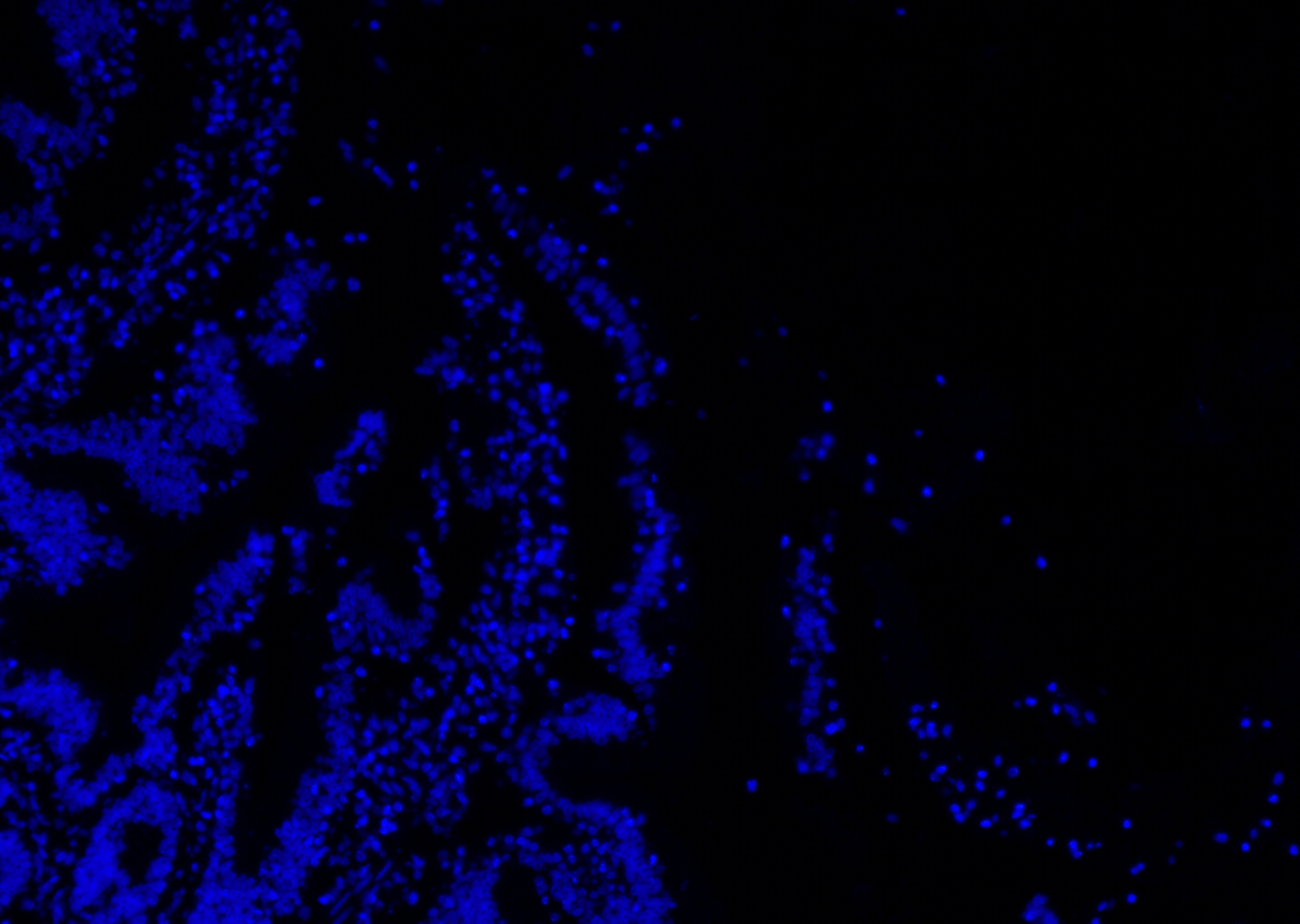

Supplement: Supplementary file 9 [file Data_Sheet_4.ZIP › NE+TA400 group-Jejunal TUNEL apoptosis/200 x/NE+TA400-2 200-4.jpg]

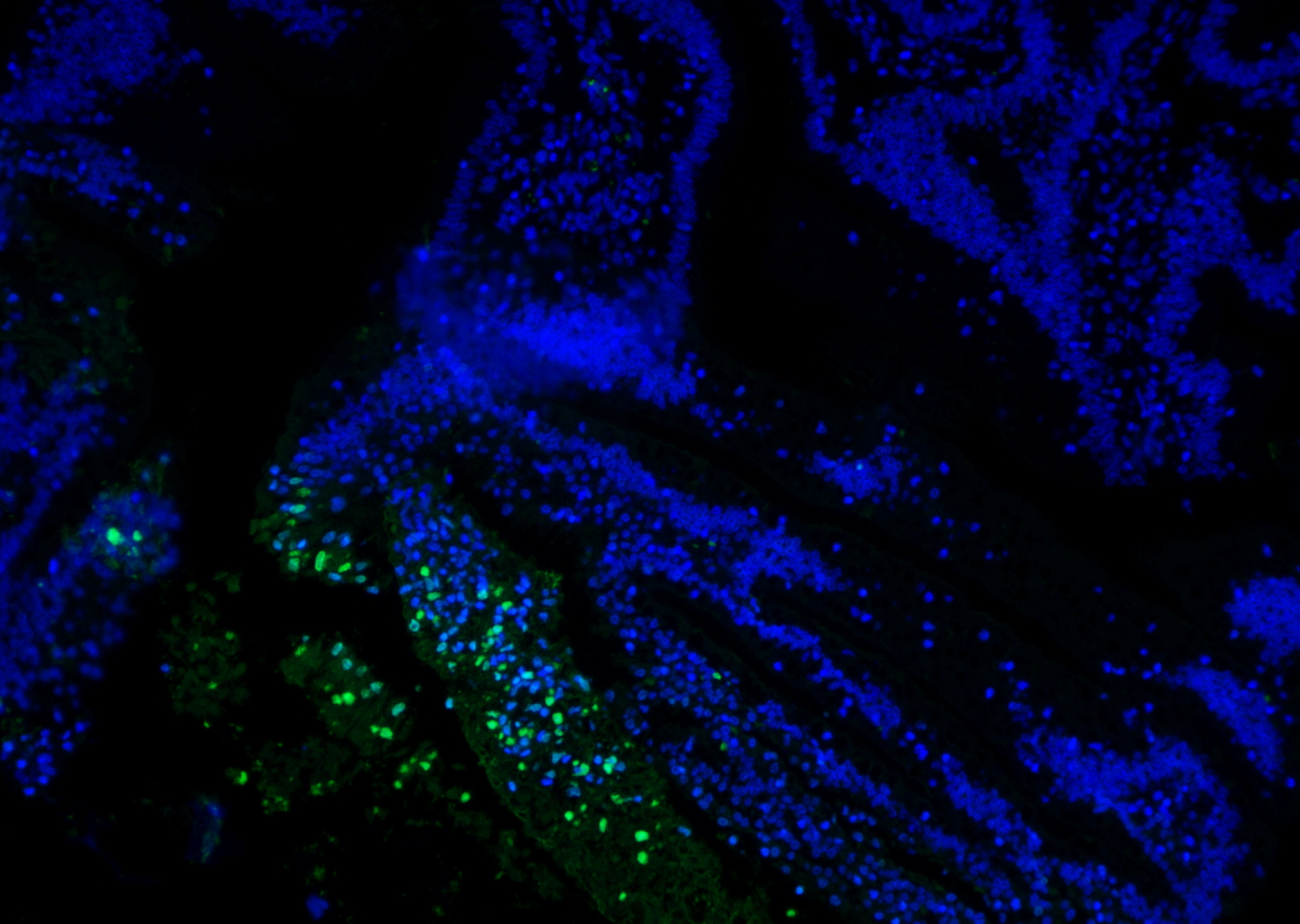

Supplement: Supplementary file 9 [file Data_Sheet_4.ZIP › NE+TA400 group-Jejunal TUNEL apoptosis/200 x/NE+TA400-2 200-5 6.jpg]

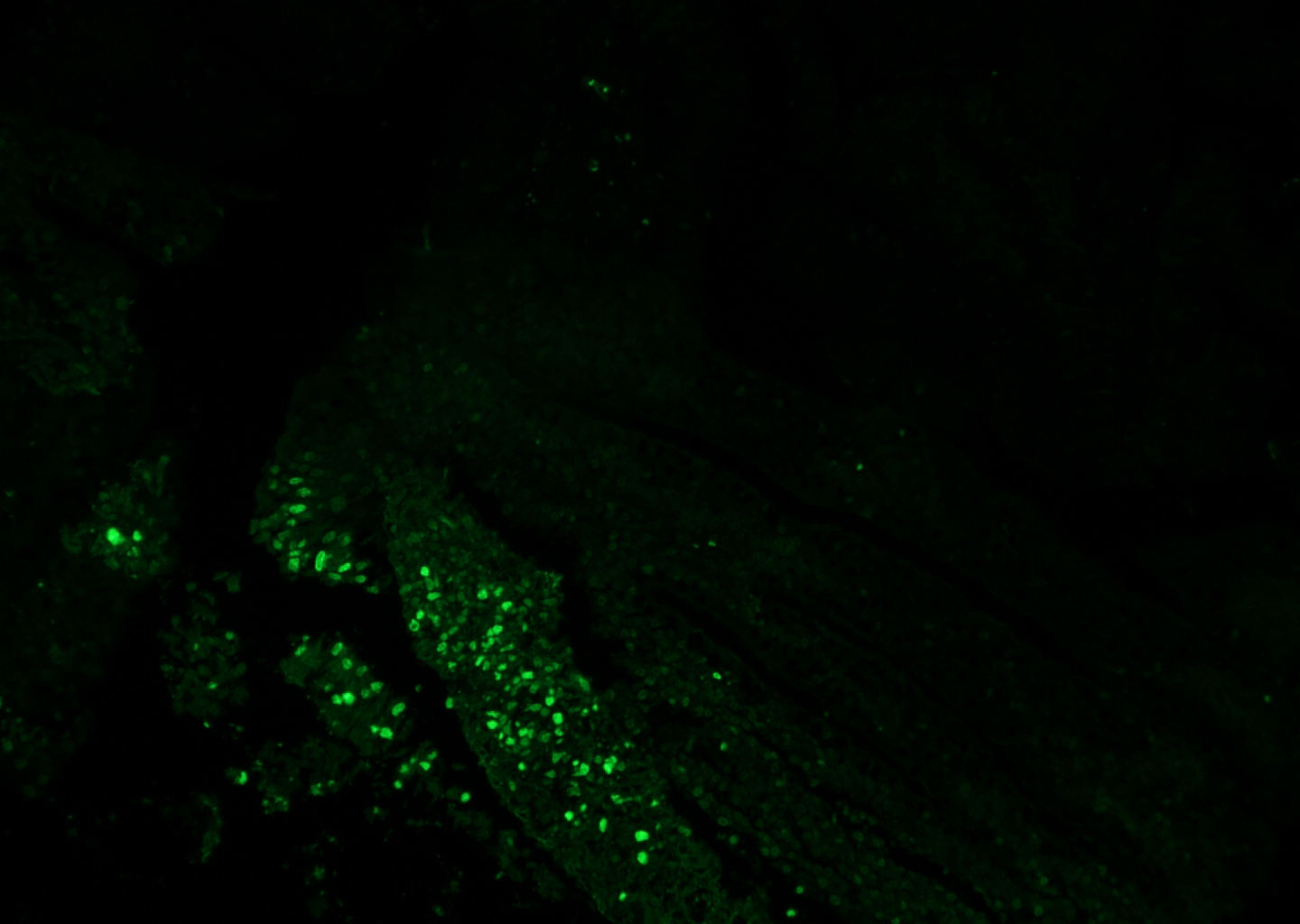

Supplement: Supplementary file 9 [file Data_Sheet_4.ZIP › NE+TA400 group-Jejunal TUNEL apoptosis/200 x/NE+TA400-2 200-5.jpg]

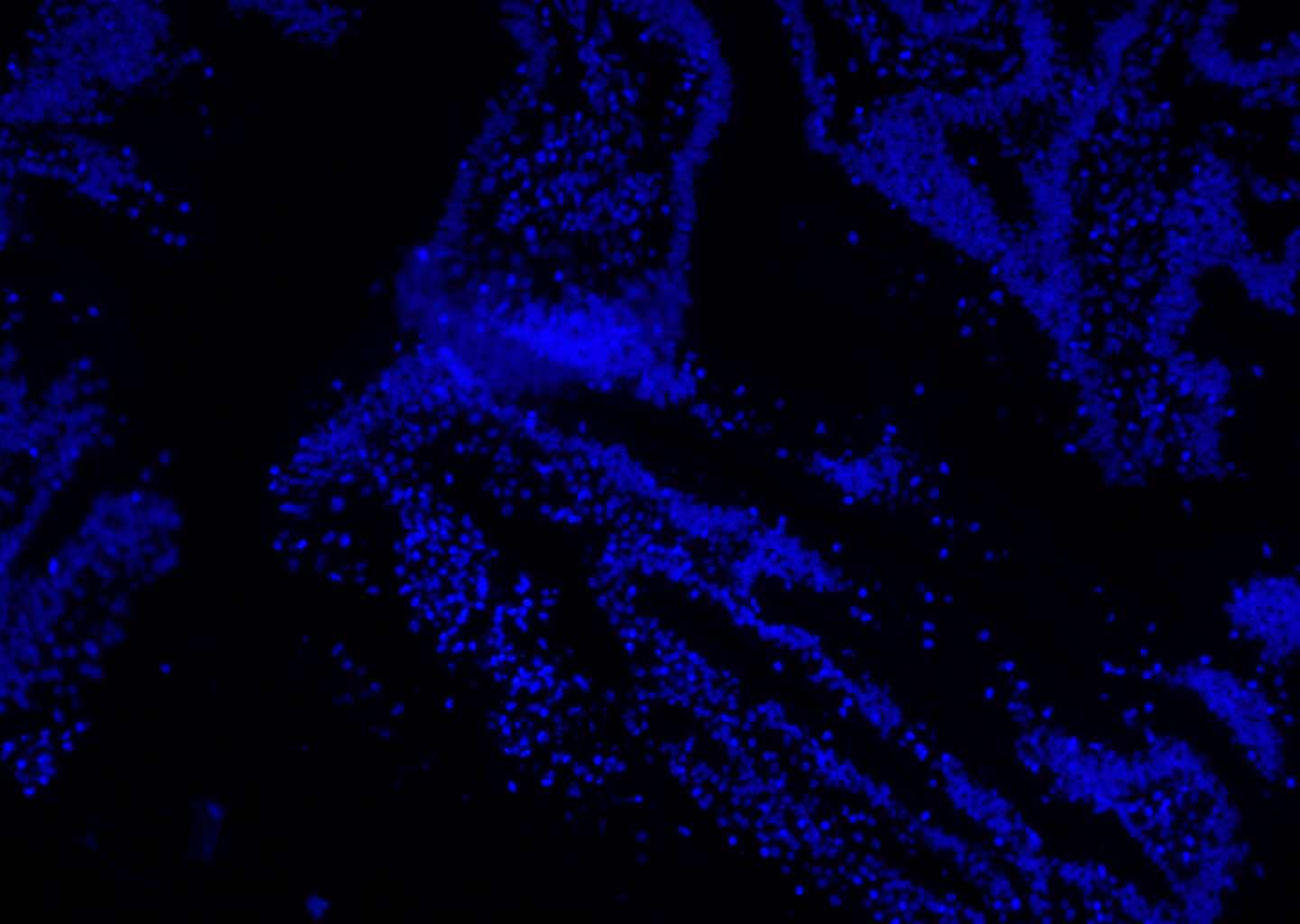

Supplement: Supplementary file 9 [file Data_Sheet_4.ZIP › NE+TA400 group-Jejunal TUNEL apoptosis/200 x/NE+TA400-2 200-6.jpg]

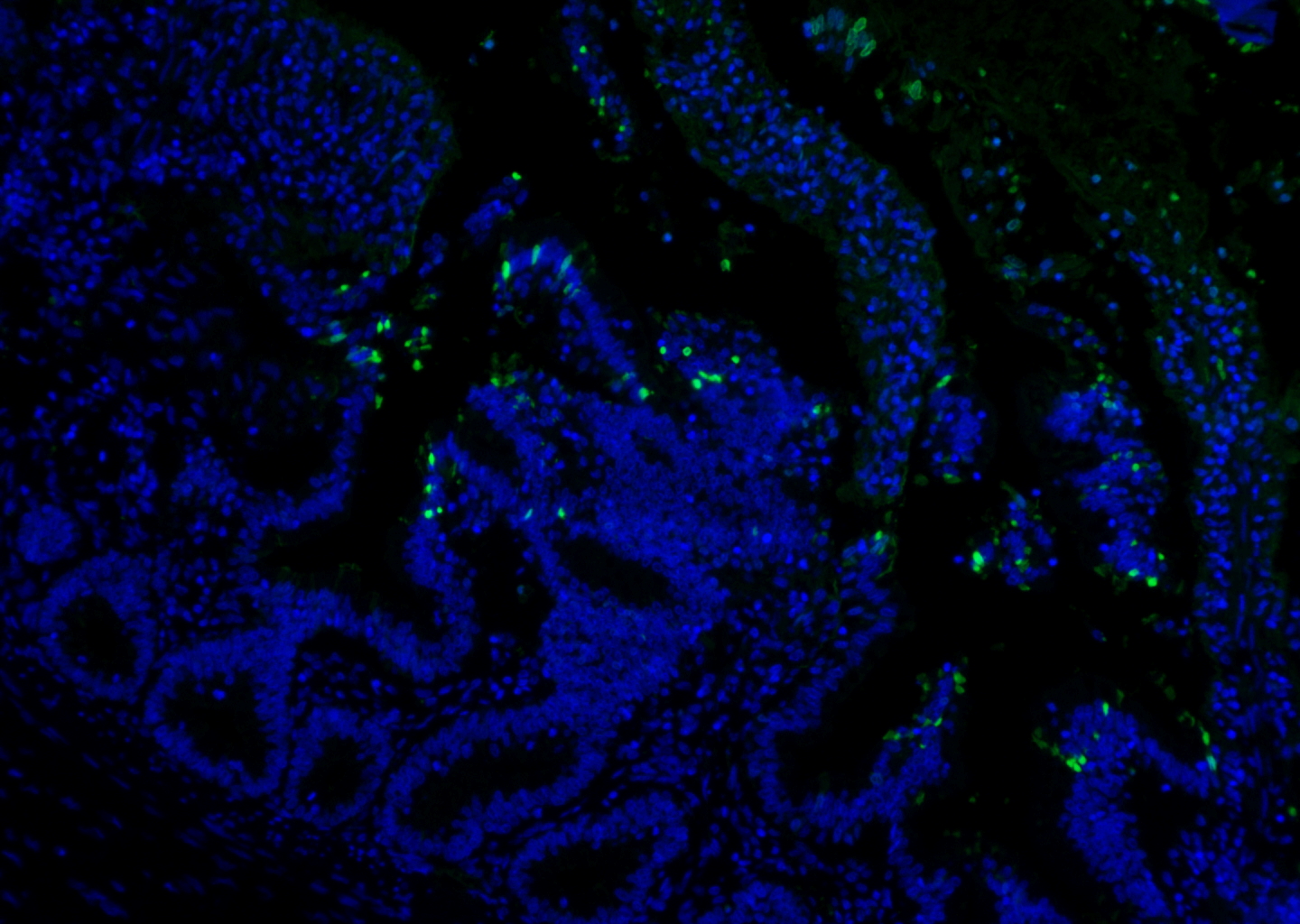

Supplement: Supplementary file 9 [file Data_Sheet_4.ZIP › NE+TA400 group-Jejunal TUNEL apoptosis/200 x/NE+TA400-2 200-1 2.jpg]

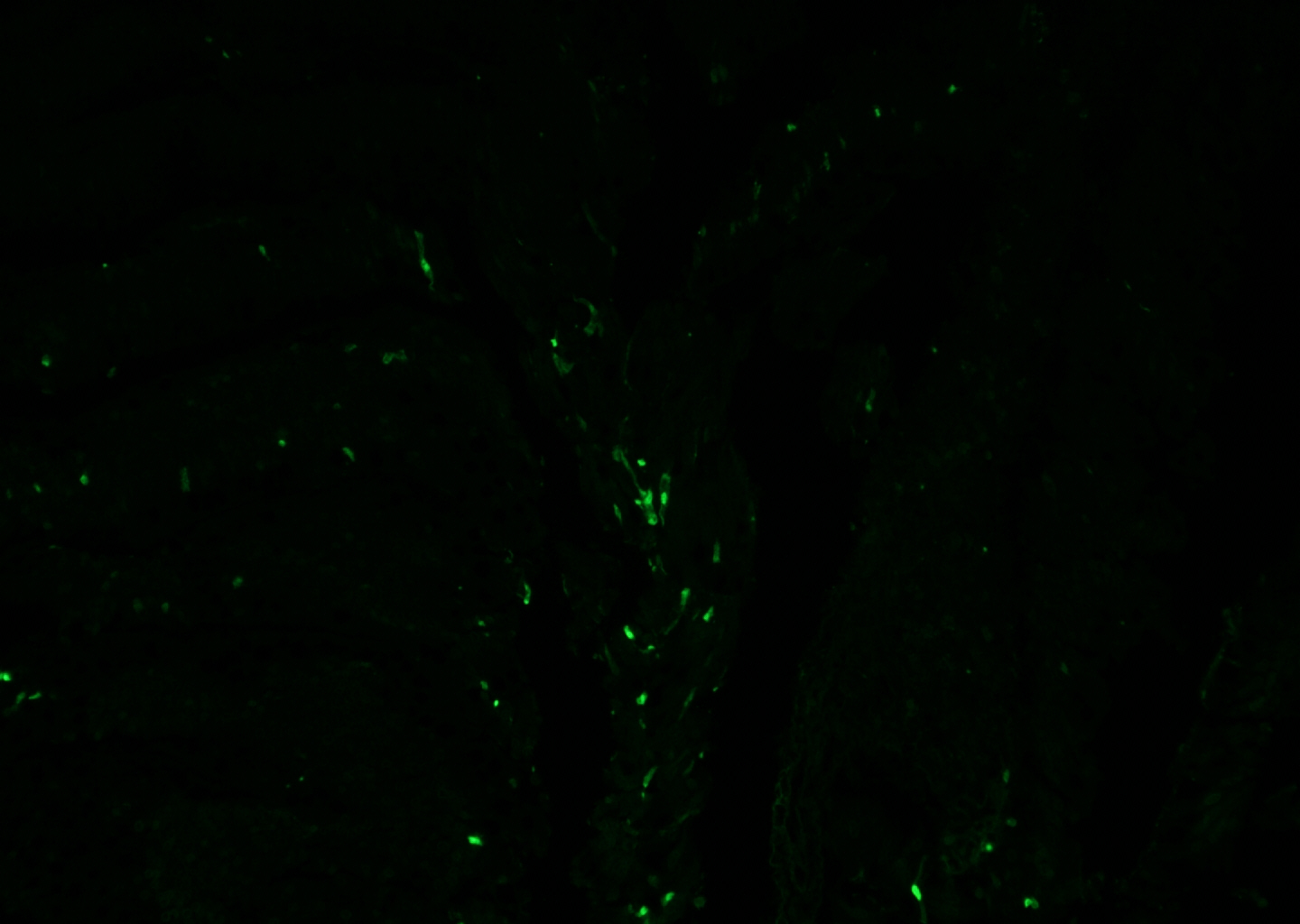

Supplement: Supplementary file 9 [file Data_Sheet_4.ZIP › NE+TA400 group-Jejunal TUNEL apoptosis/400 x/NE+TA400-1 200-5.jpg]

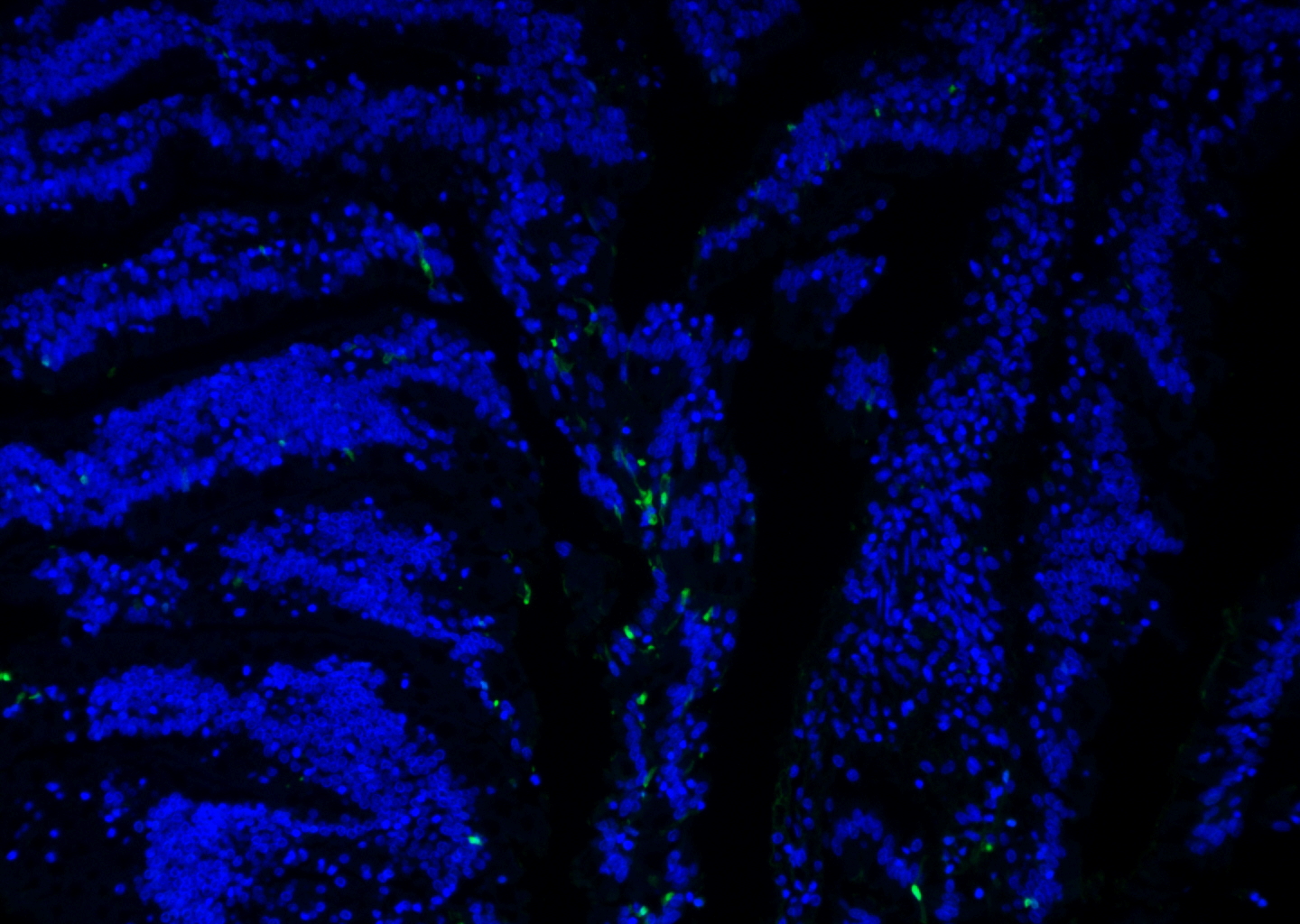

Supplement: Supplementary file 9 [file Data_Sheet_4.ZIP › NE+TA400 group-Jejunal TUNEL apoptosis/400 x/NE+TA400-1 200-5 6.jpg]

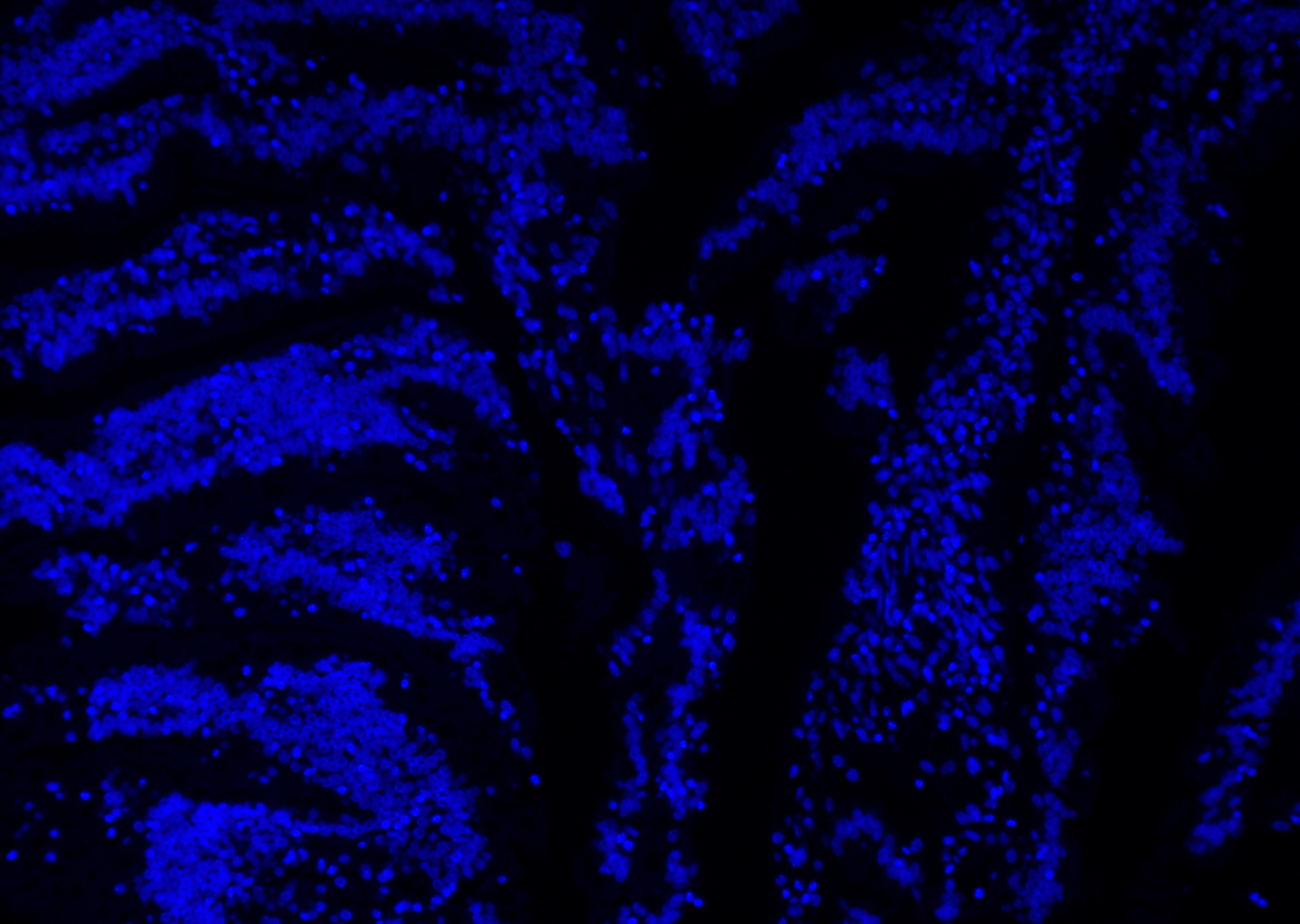

Supplement: Supplementary file 9 [file Data_Sheet_4.ZIP › NE+TA400 group-Jejunal TUNEL apoptosis/400 x/NE+TA400-1 200-6.jpg]

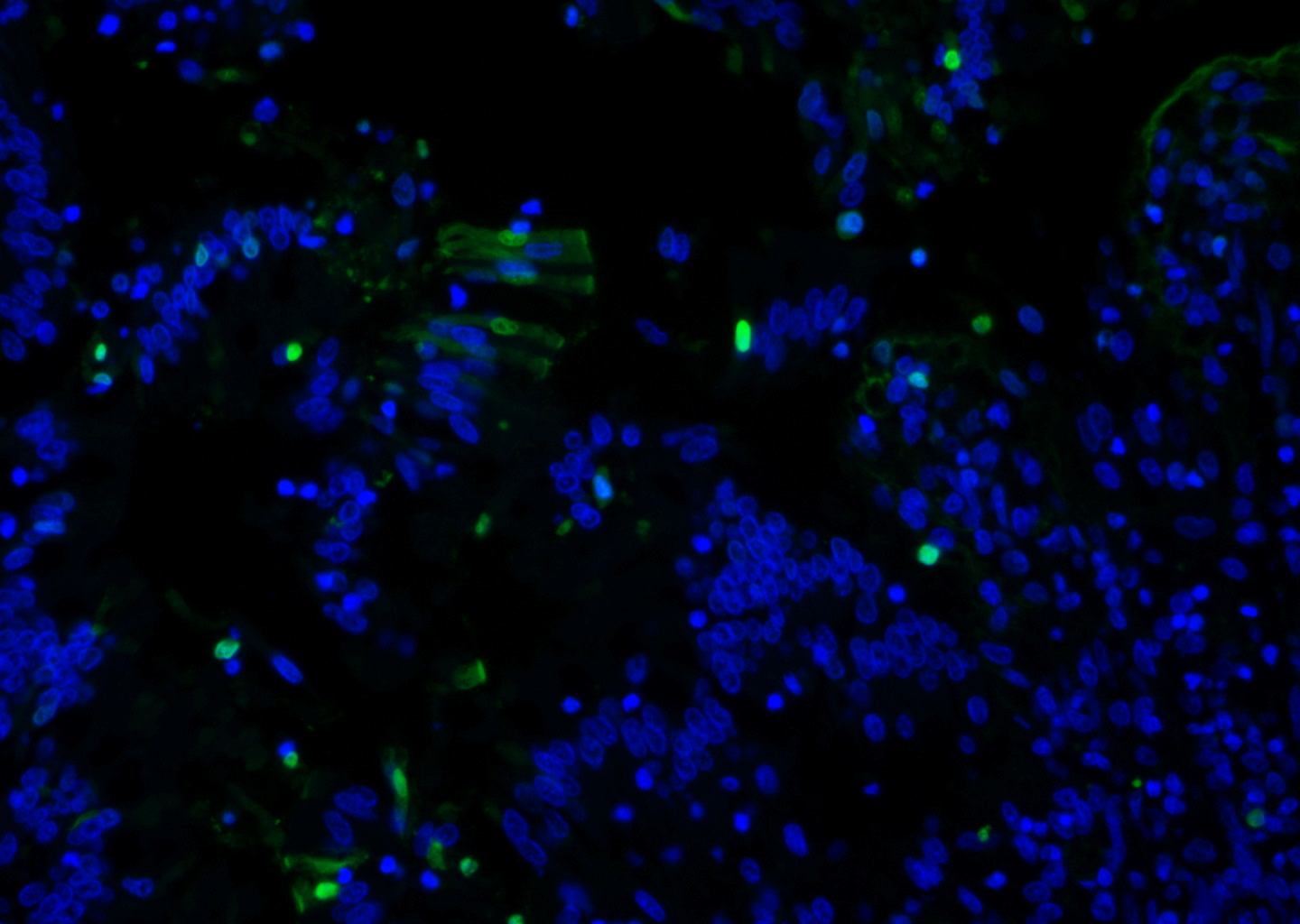

Supplement: Supplementary file 9 [file Data_Sheet_4.ZIP › NE+TA400 group-Jejunal TUNEL apoptosis/400 x/NE+TA400-1 400-1 2.jpg]

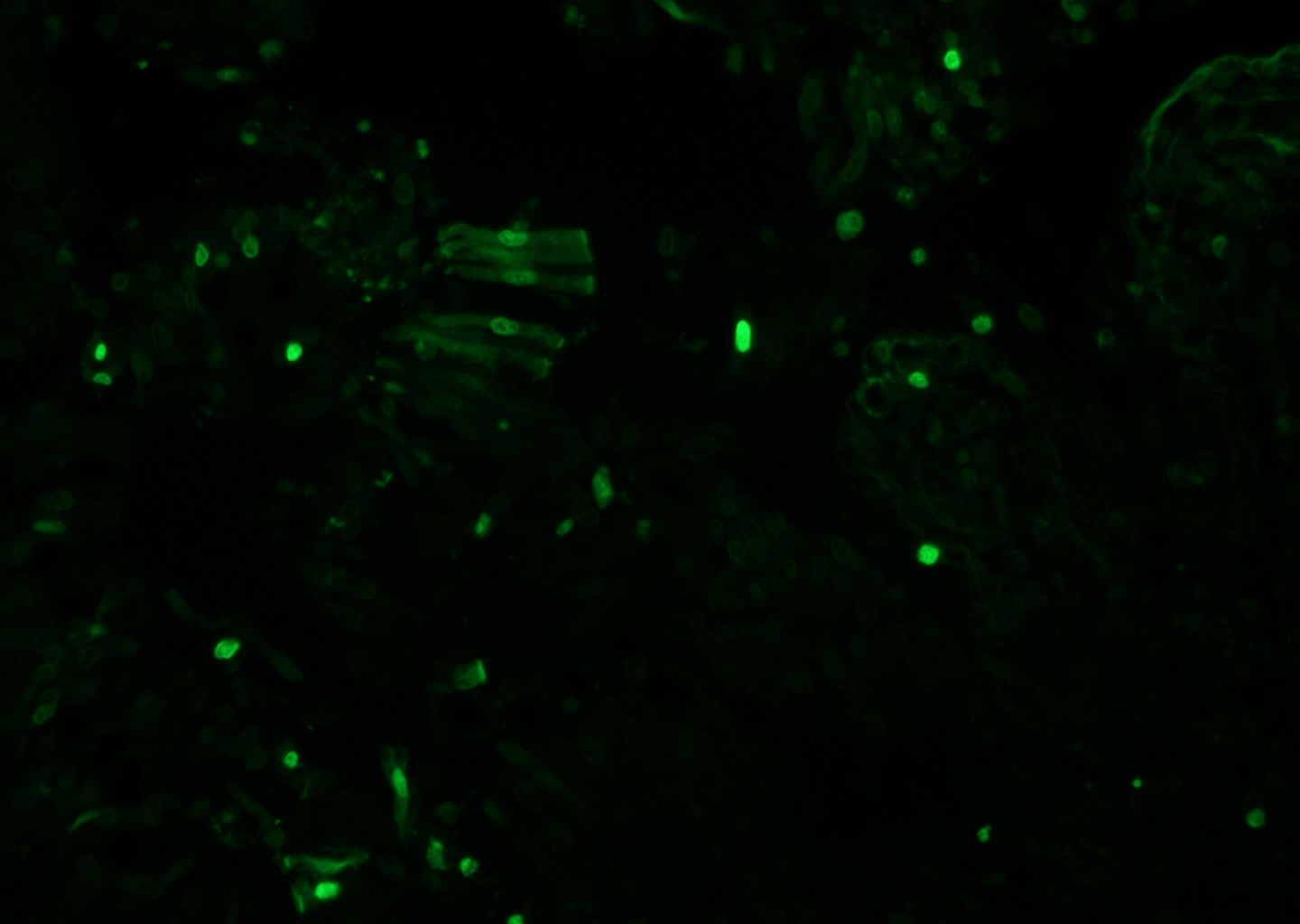

Supplement: Supplementary file 9 [file Data_Sheet_4.ZIP › NE+TA400 group-Jejunal TUNEL apoptosis/400 x/NE+TA400-1 400-1.jpg]

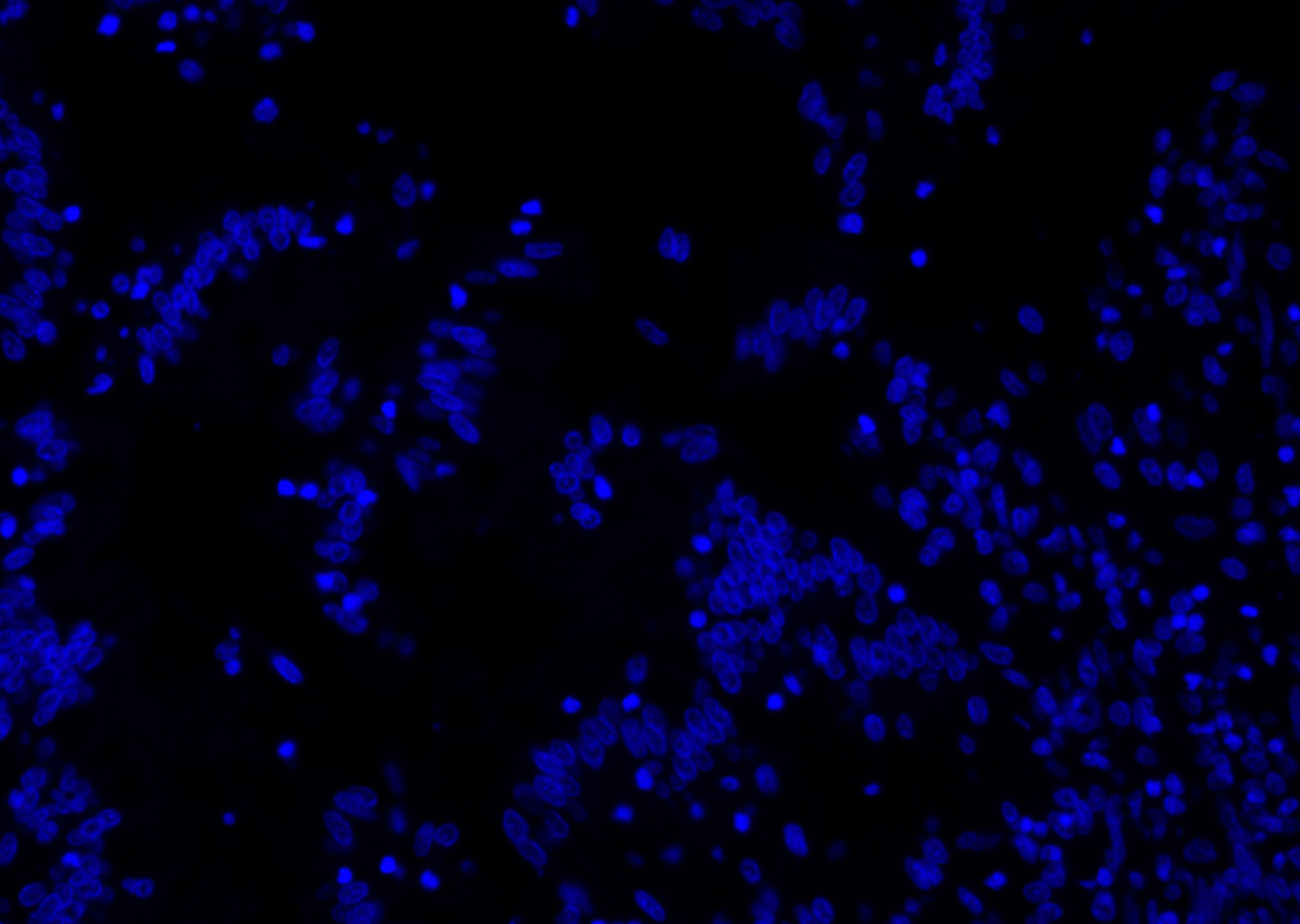

Supplement: Supplementary file 9 [file Data_Sheet_4.ZIP › NE+TA400 group-Jejunal TUNEL apoptosis/400 x/NE+TA400-1 400-2.jpg]

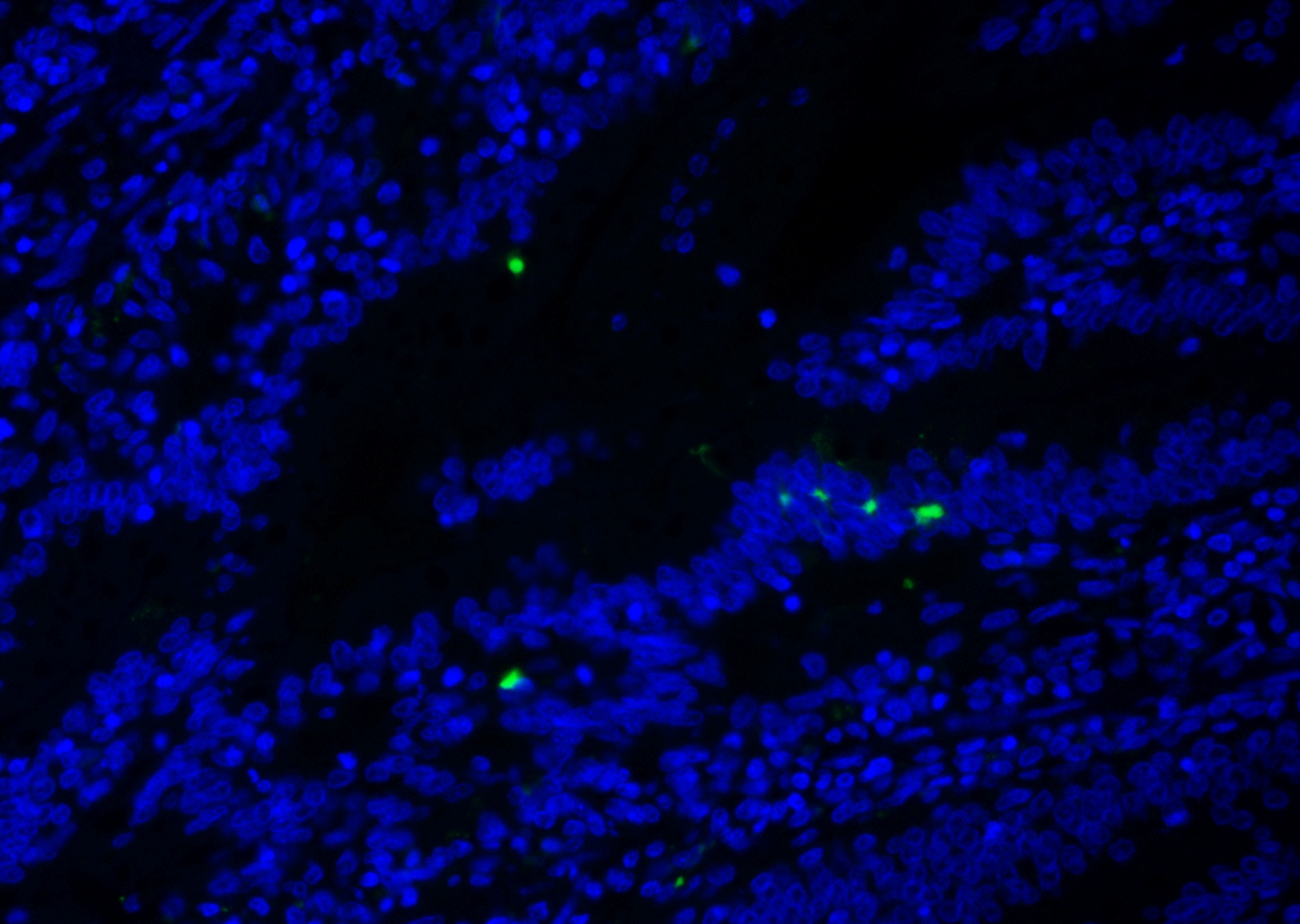

Supplement: Supplementary file 9 [file Data_Sheet_4.ZIP › NE+TA400 group-Jejunal TUNEL apoptosis/400 x/NE+TA400-1 400-3 4.jpg]

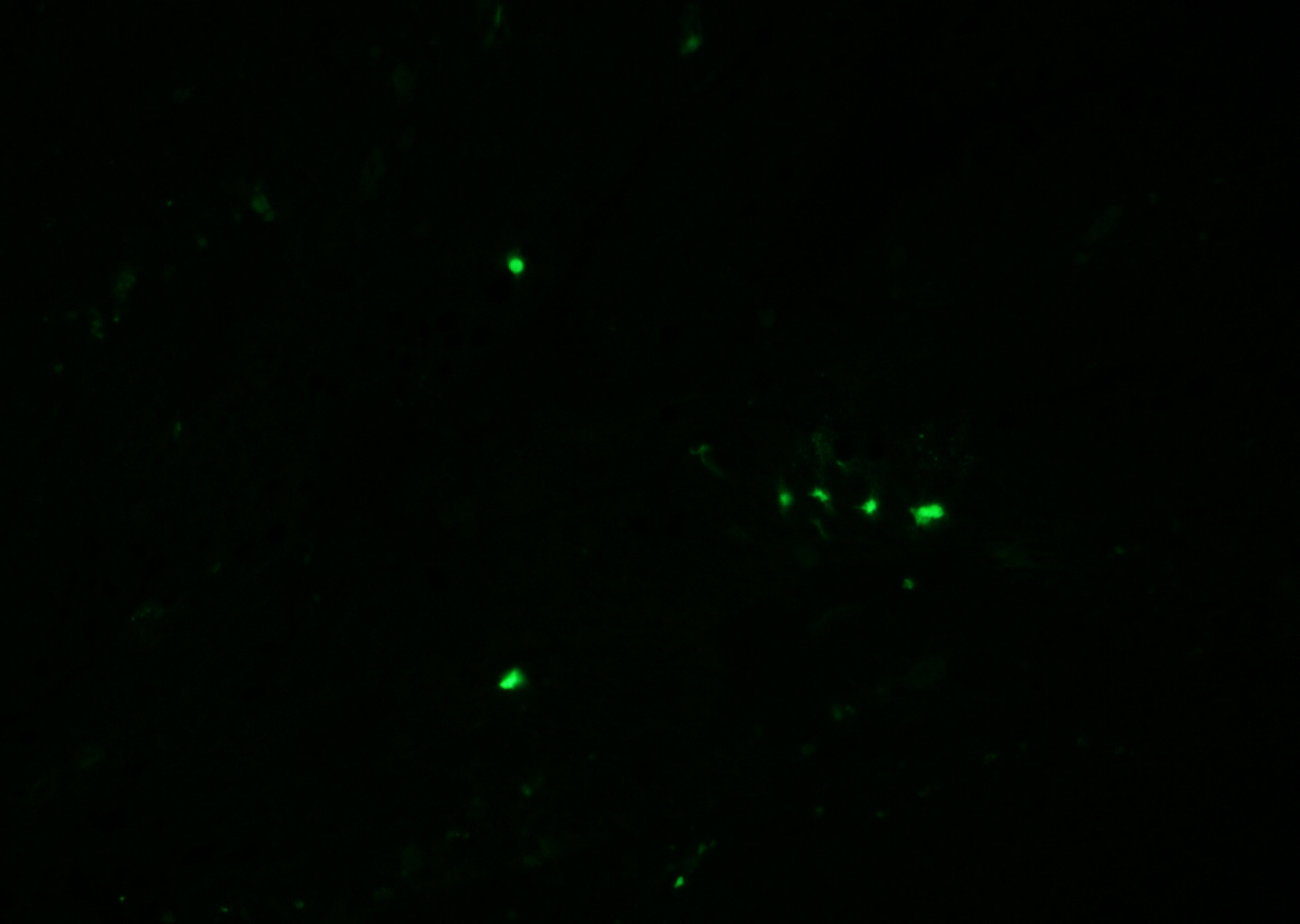

Supplement: Supplementary file 9 [file Data_Sheet_4.ZIP › NE+TA400 group-Jejunal TUNEL apoptosis/400 x/NE+TA400-1 400-3.jpg]

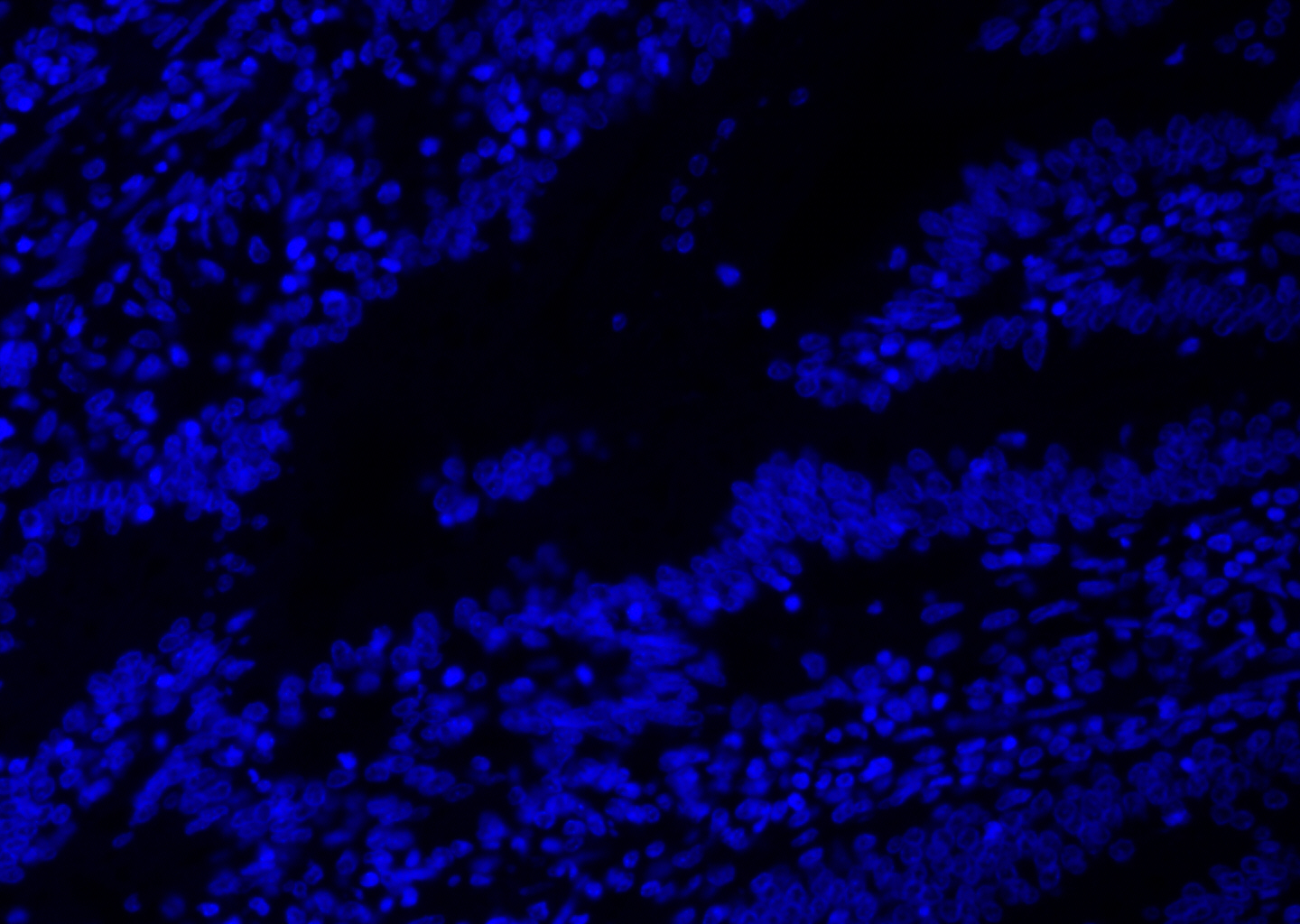

Supplement: Supplementary file 9 [file Data_Sheet_4.ZIP › NE+TA400 group-Jejunal TUNEL apoptosis/400 x/NE+TA400-1 400-4.jpg]

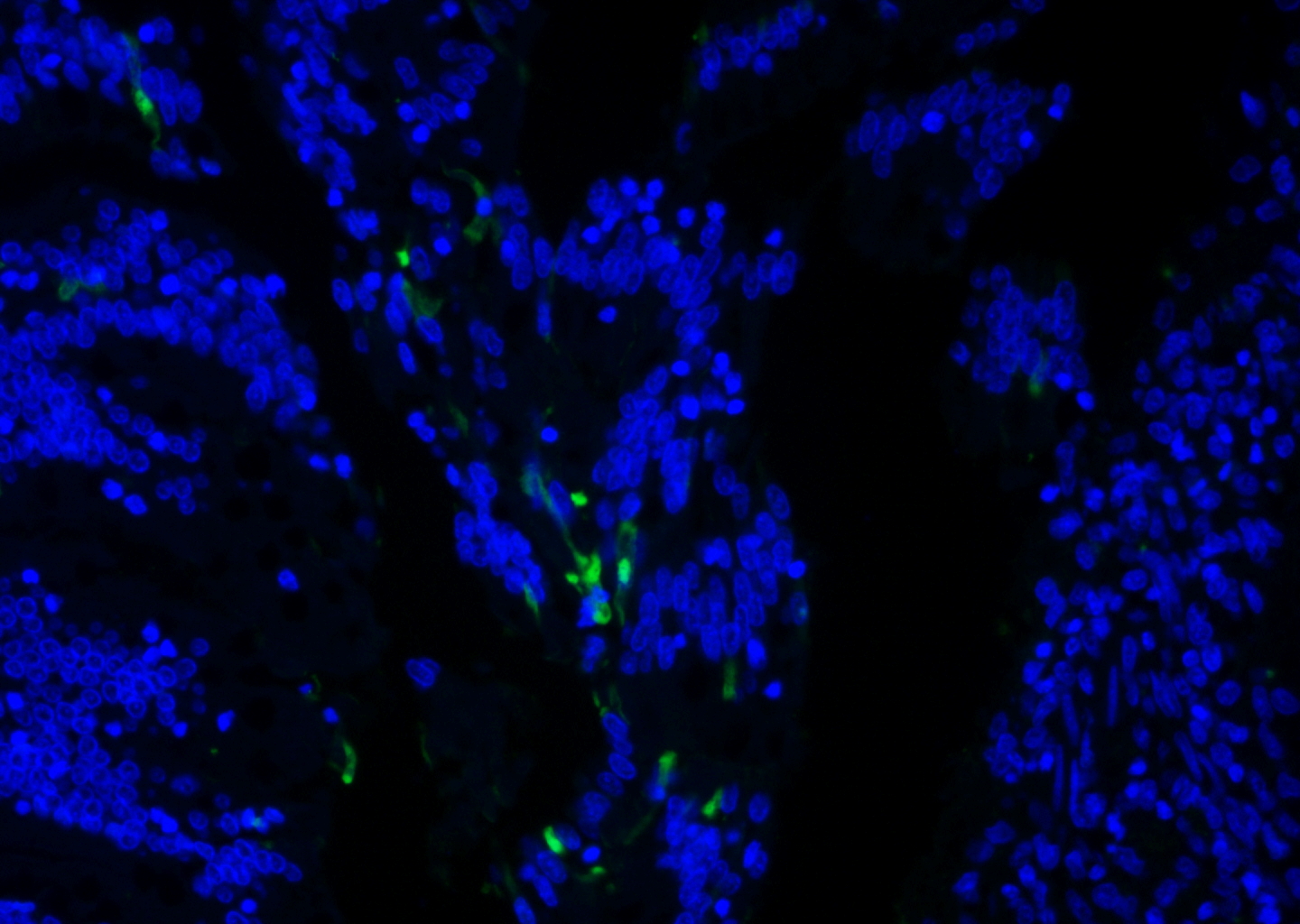

Supplement: Supplementary file 9 [file Data_Sheet_4.ZIP › NE+TA400 group-Jejunal TUNEL apoptosis/400 x/NE+TA400-1 400-5 6.jpg]

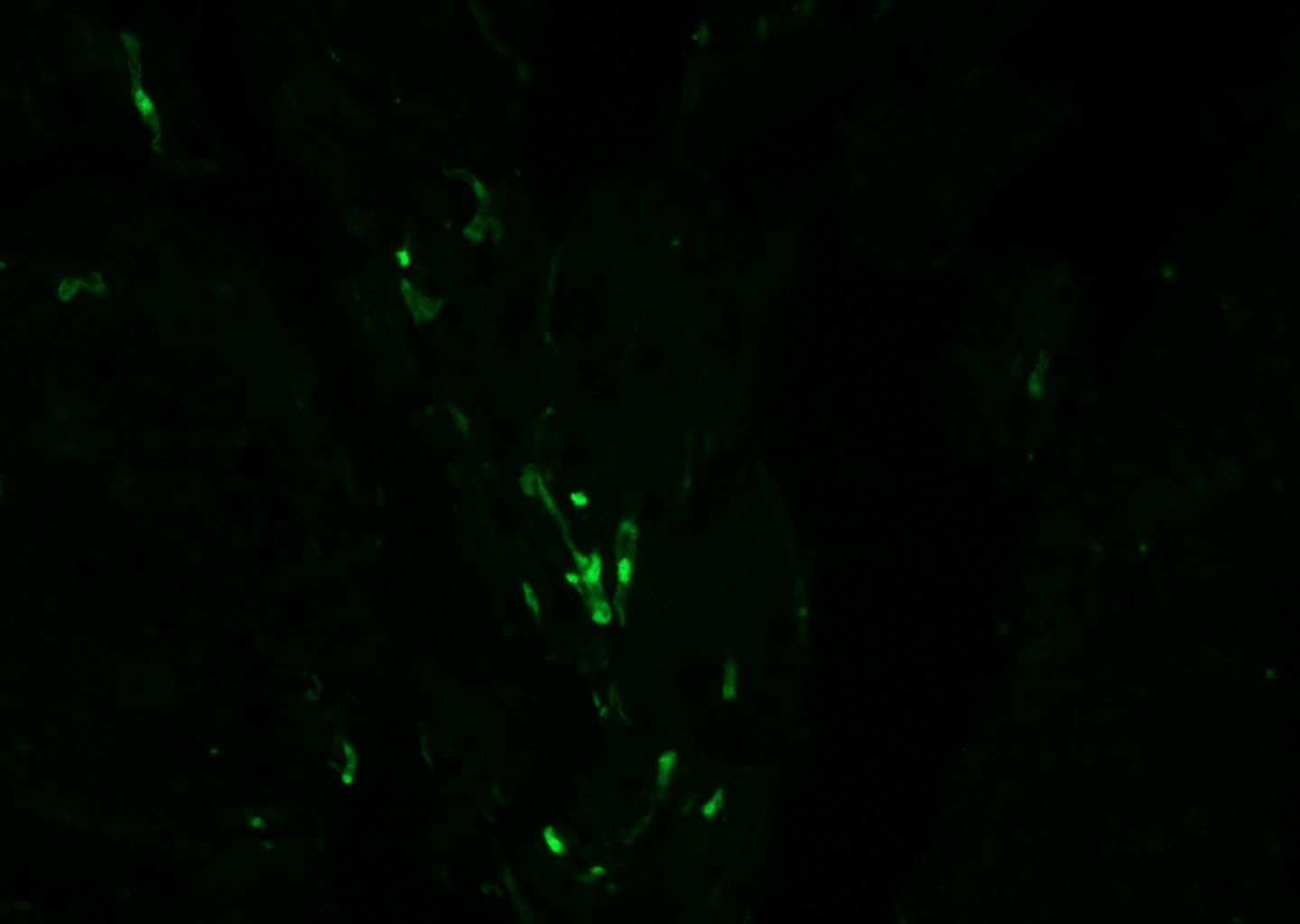

Supplement: Supplementary file 9 [file Data_Sheet_4.ZIP › NE+TA400 group-Jejunal TUNEL apoptosis/400 x/NE+TA400-1 400-5.jpg]

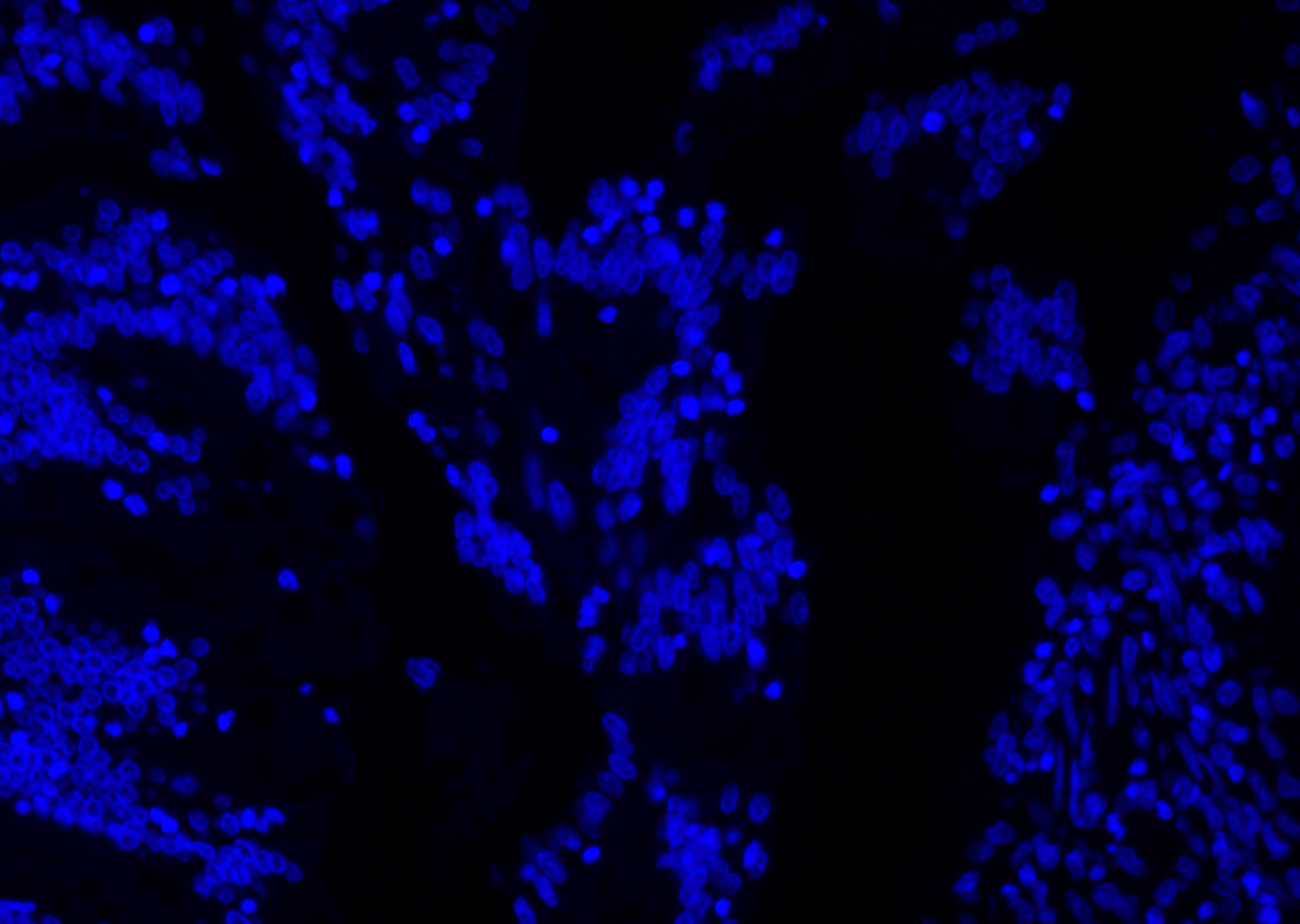

Supplement: Supplementary file 9 [file Data_Sheet_4.ZIP › NE+TA400 group-Jejunal TUNEL apoptosis/400 x/NE+TA400-1 400-6.jpg]

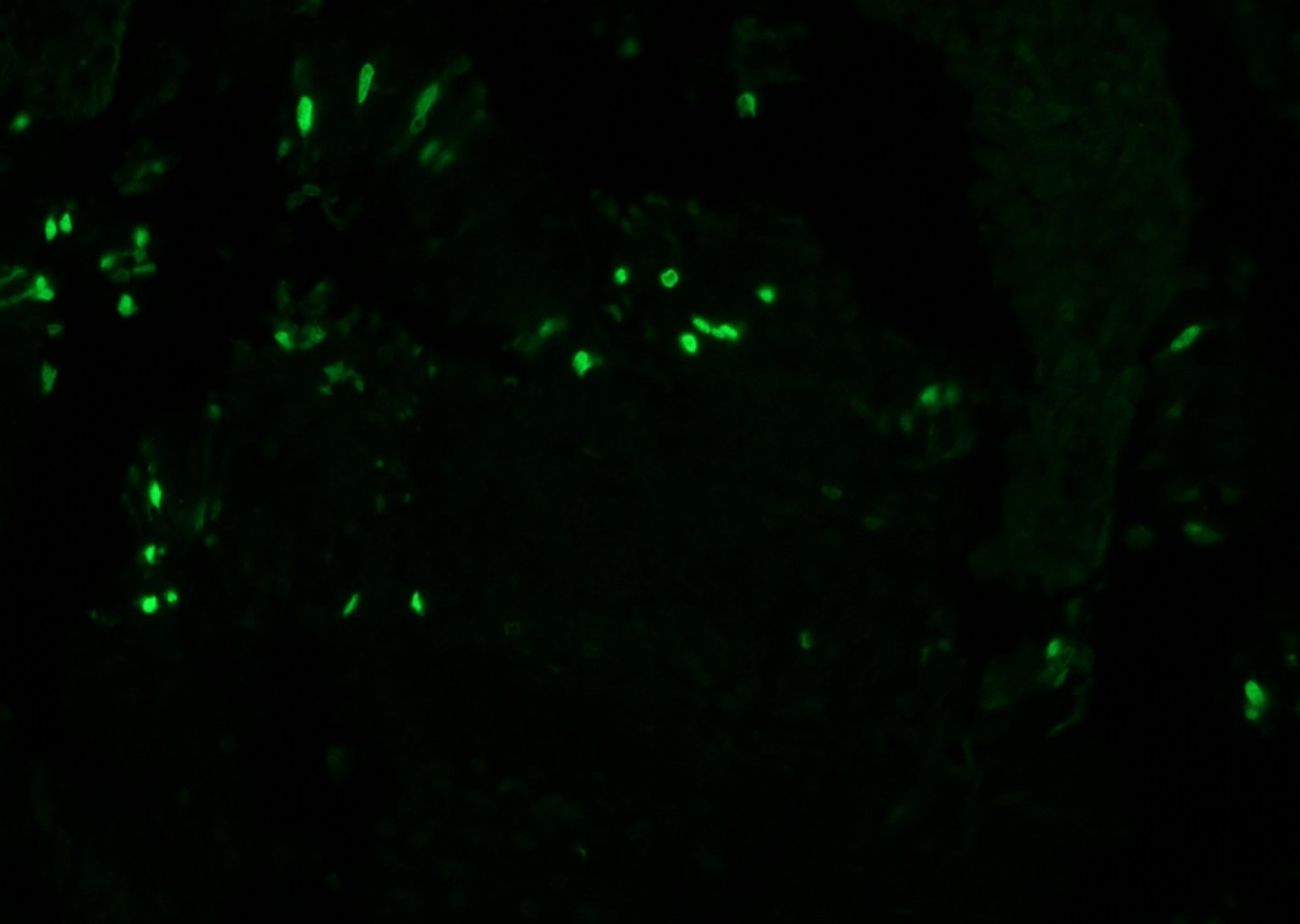

Supplement: Supplementary file 9 [file Data_Sheet_4.ZIP › NE+TA400 group-Jejunal TUNEL apoptosis/400 x/NE+TA400-2 400-1.jpg]

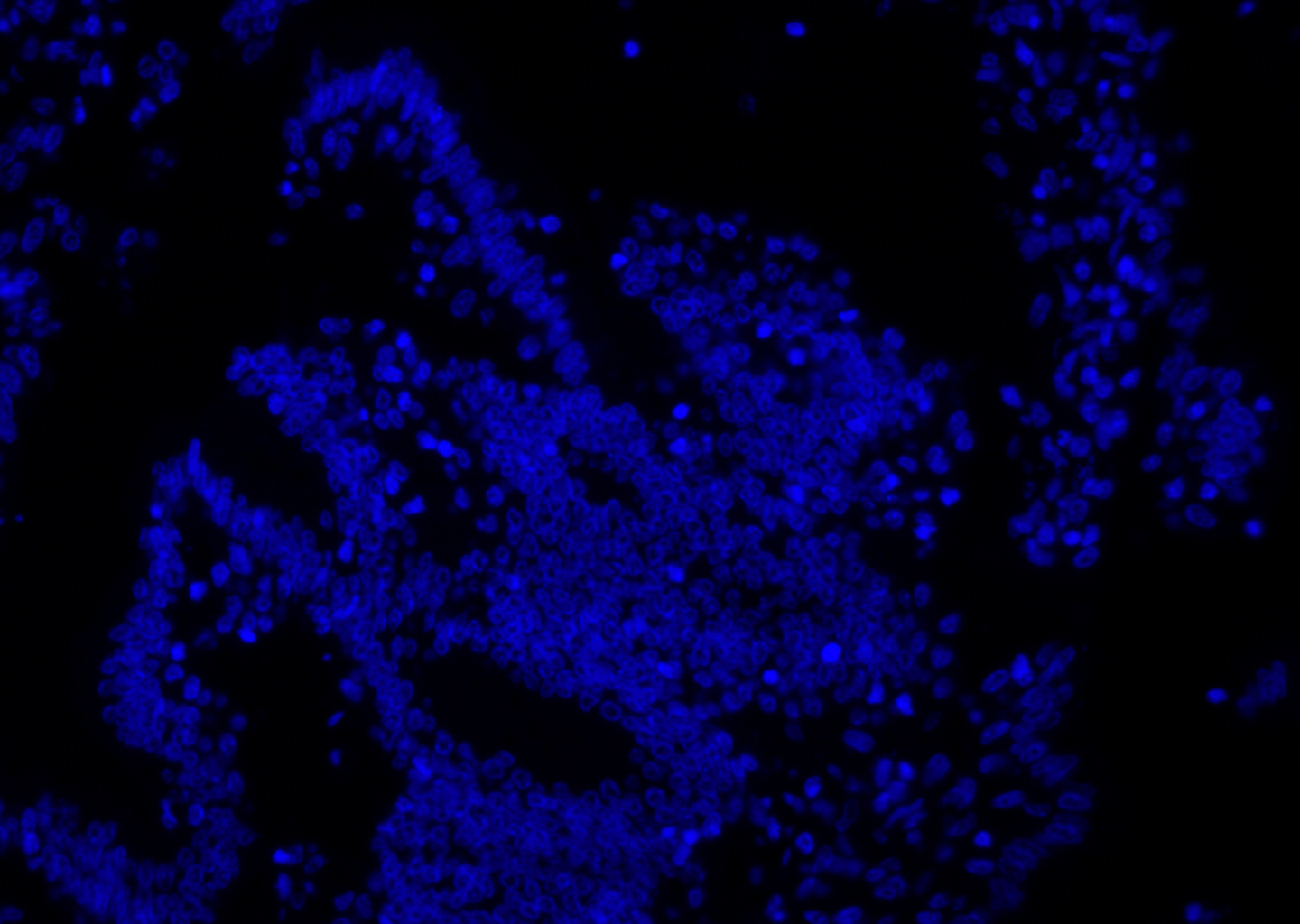

Supplement: Supplementary file 9 [file Data_Sheet_4.ZIP › NE+TA400 group-Jejunal TUNEL apoptosis/400 x/NE+TA400-2 400-2.jpg]

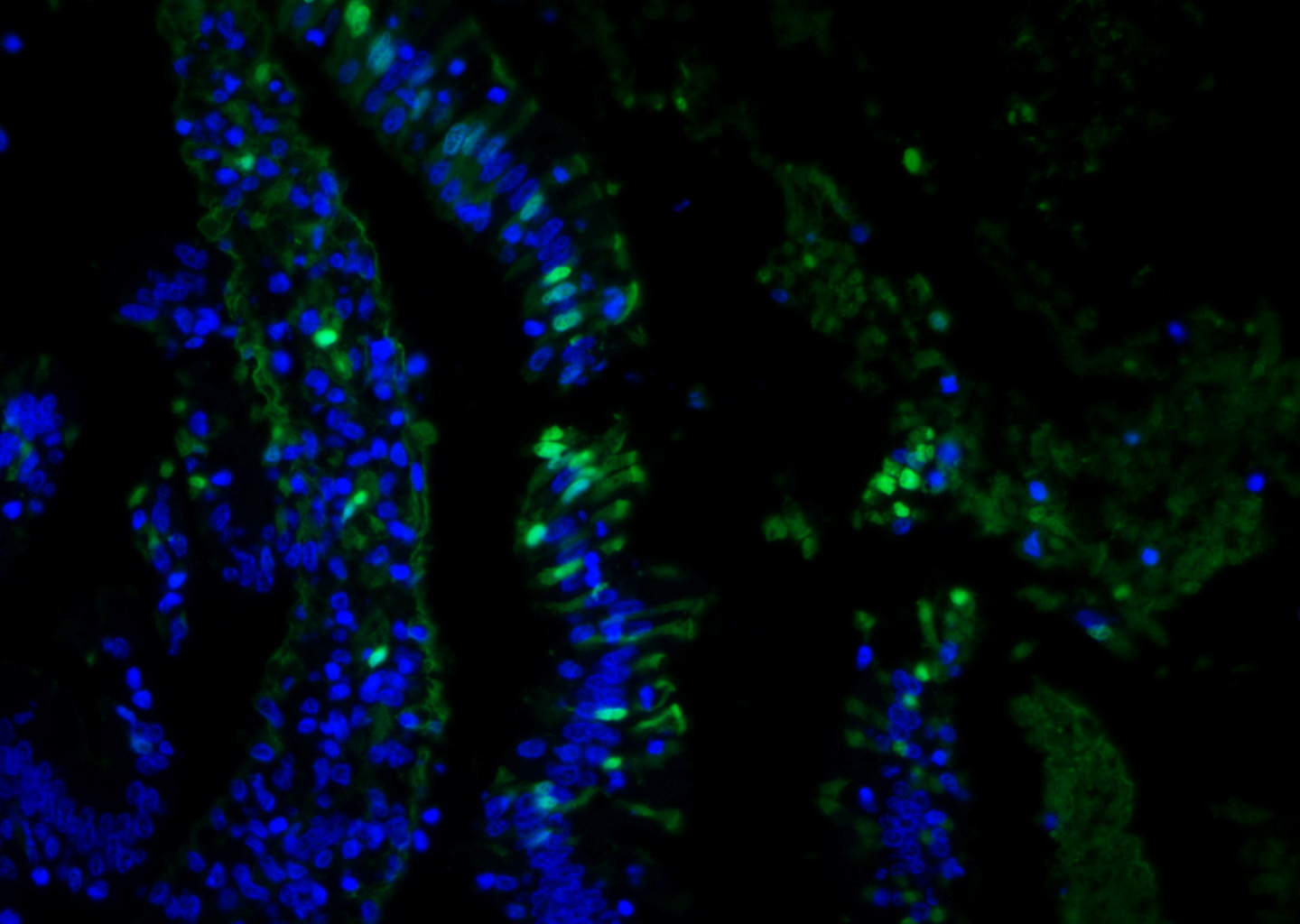

Supplement: Supplementary file 9 [file Data_Sheet_4.ZIP › NE+TA400 group-Jejunal TUNEL apoptosis/400 x/NE+TA400-2 400-3 4.jpg]

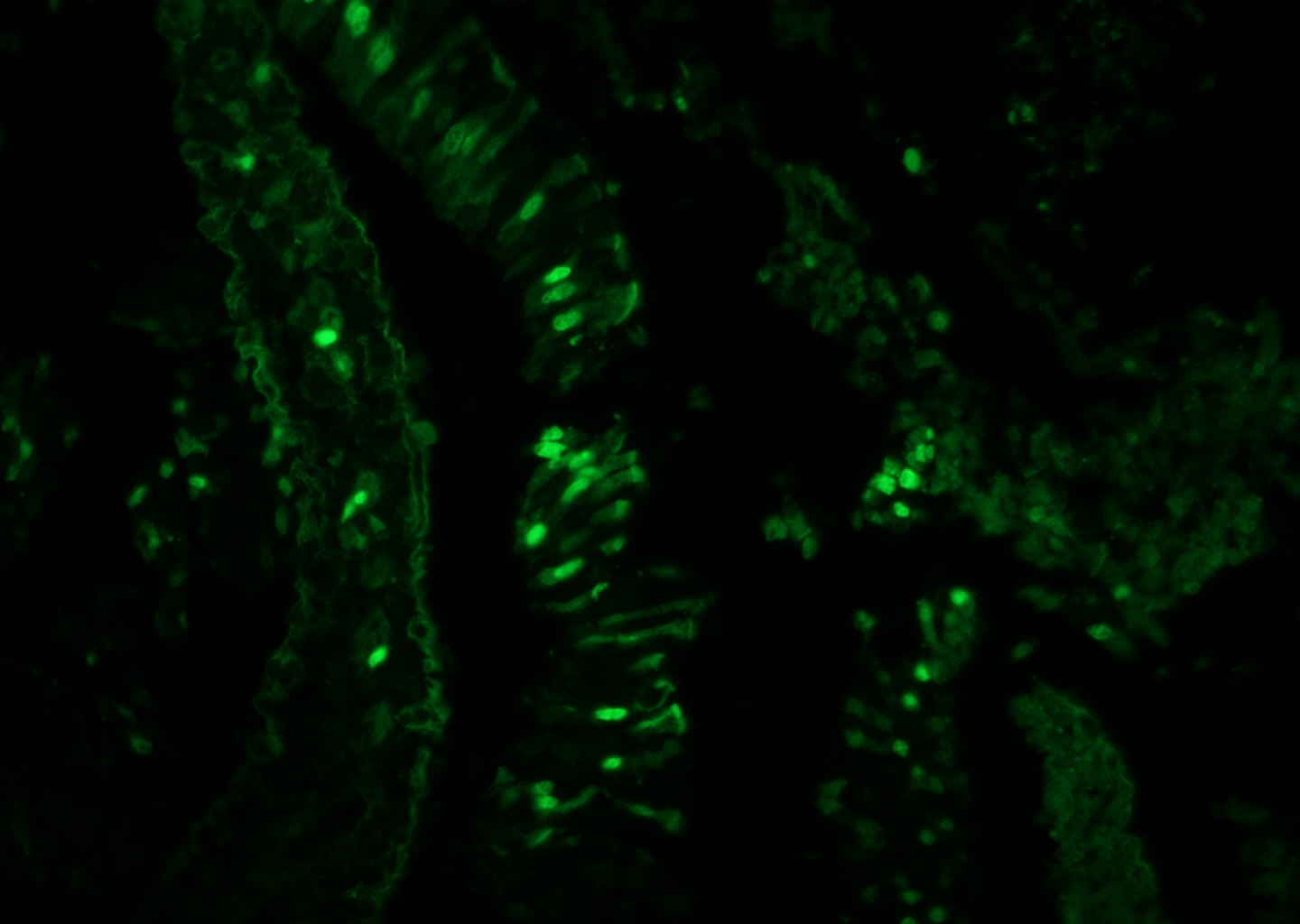

Supplement: Supplementary file 9 [file Data_Sheet_4.ZIP › NE+TA400 group-Jejunal TUNEL apoptosis/400 x/NE+TA400-2 400-3.jpg]
