# Supplementary figures and images for: Trans-Anethole Alleviates Subclinical Necro-Haemorrhagic Enteritis-Induced Intestinal Barrier Dysfunction and Intestinal Inflammation in Broilers (part 5 of 5)
Source: Front Microbiol. 2022 Mar 21;13:831882. doi: 10.3389/fmicb.2022.831882 (PMC8977854; doi:10.3389/fmicb.2022.831882)

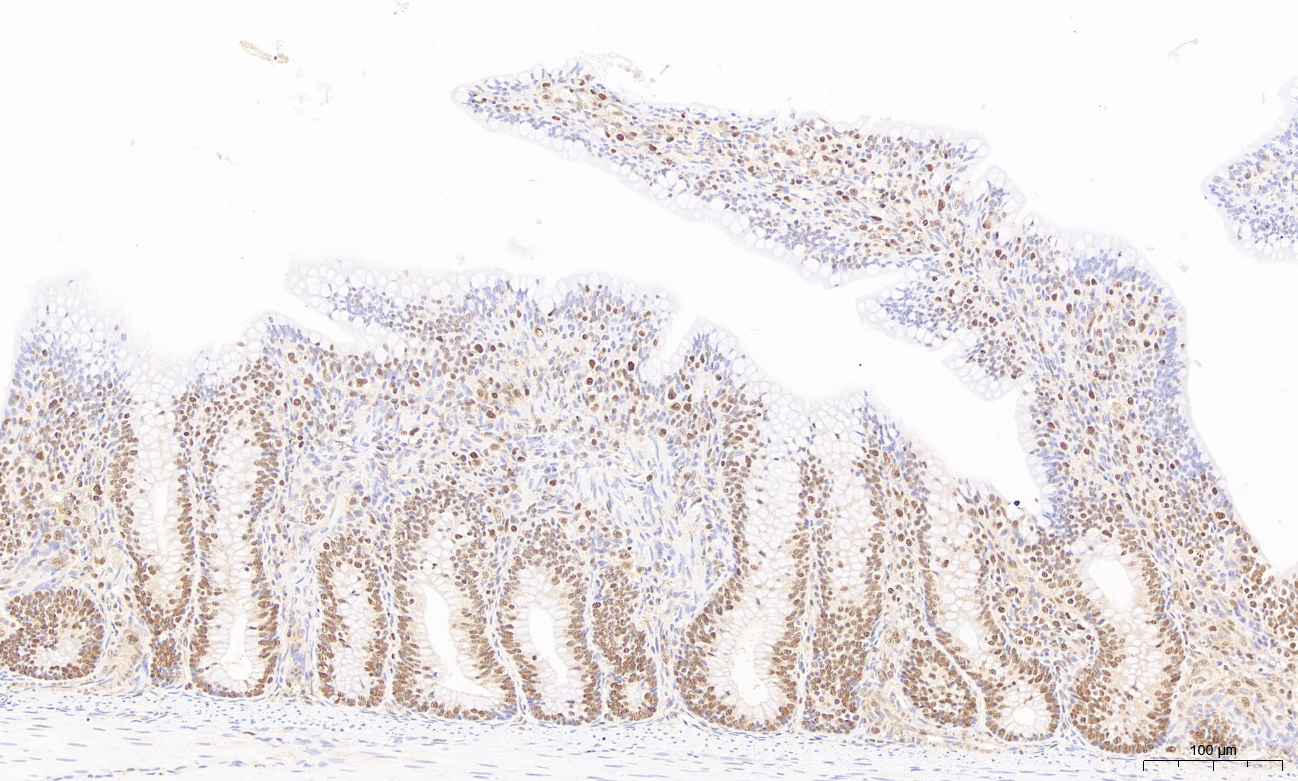

Supplement: Supplementary file 15 [file Data_Sheet_10.ZIP › Ileal PCNA Immunohistochemical staining 1/NE group/8.jpg]

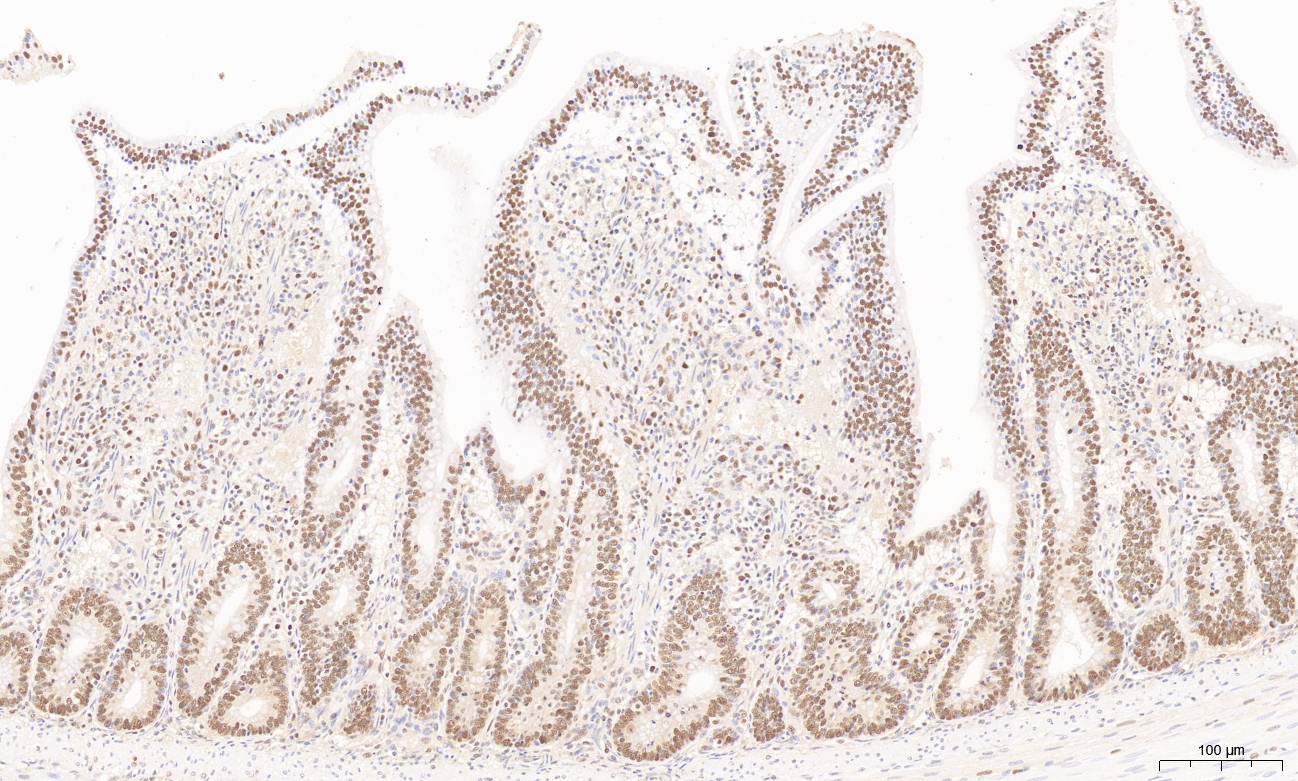

Supplement: Supplementary file 16 [file Data_Sheet_11.ZIP › Ileal PCNA Immunohistochemical staining 2/NE+TA400 group/1.jpg]

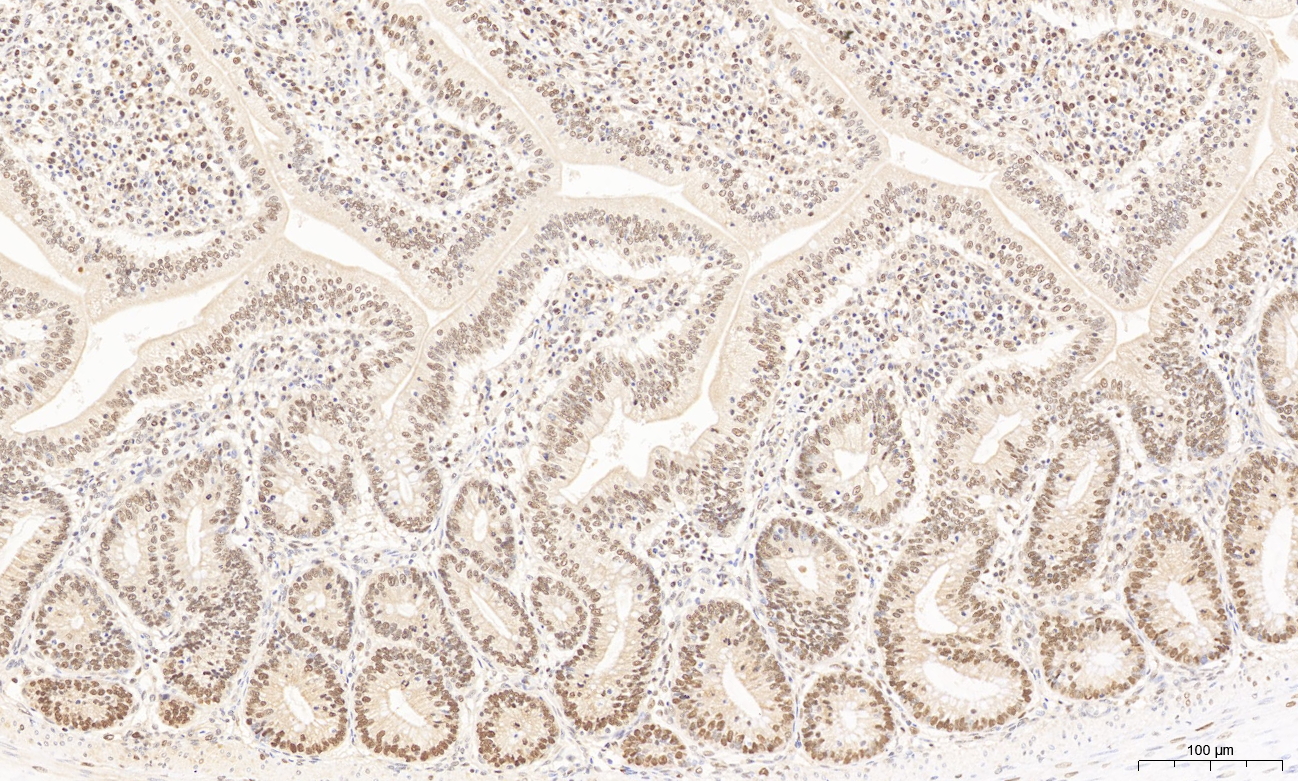

Supplement: Supplementary file 16 [file Data_Sheet_11.ZIP › Ileal PCNA Immunohistochemical staining 2/NE+TA400 group/2.jpg]

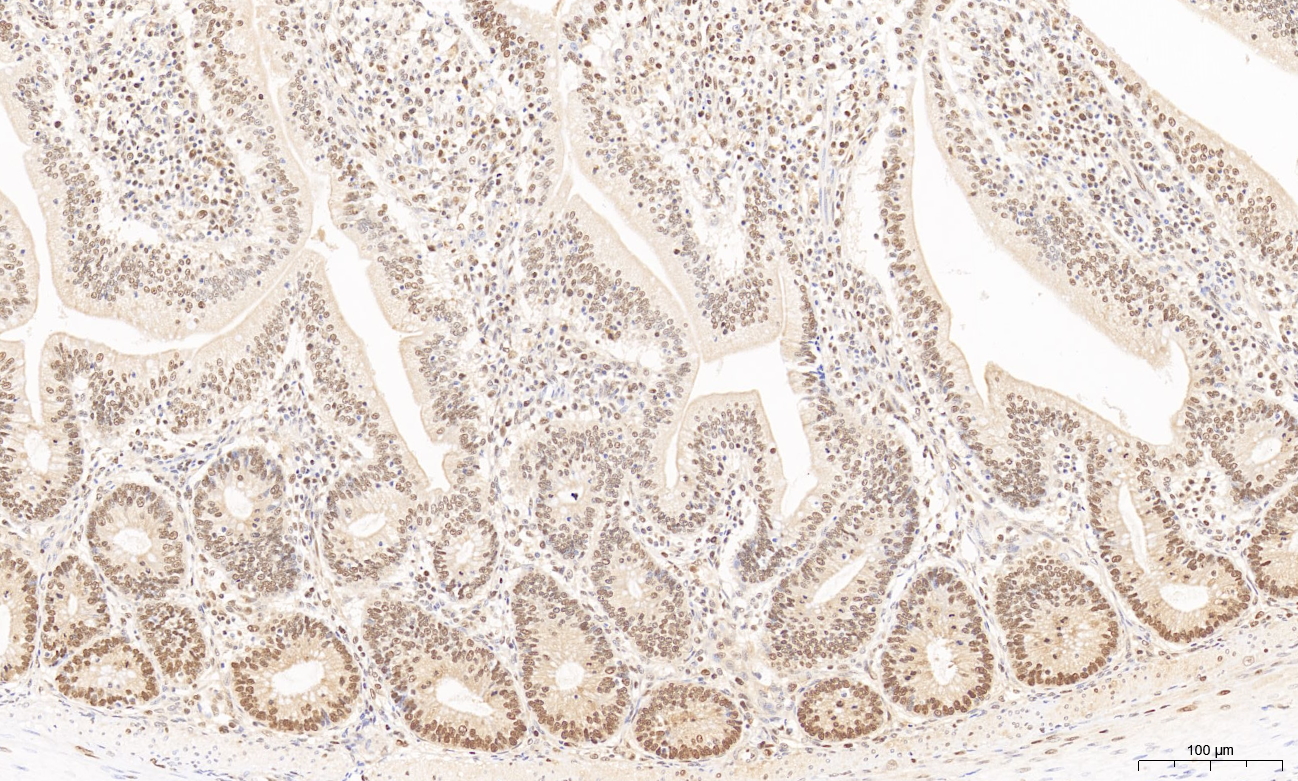

Supplement: Supplementary file 16 [file Data_Sheet_11.ZIP › Ileal PCNA Immunohistochemical staining 2/NE+TA400 group/3.jpg]

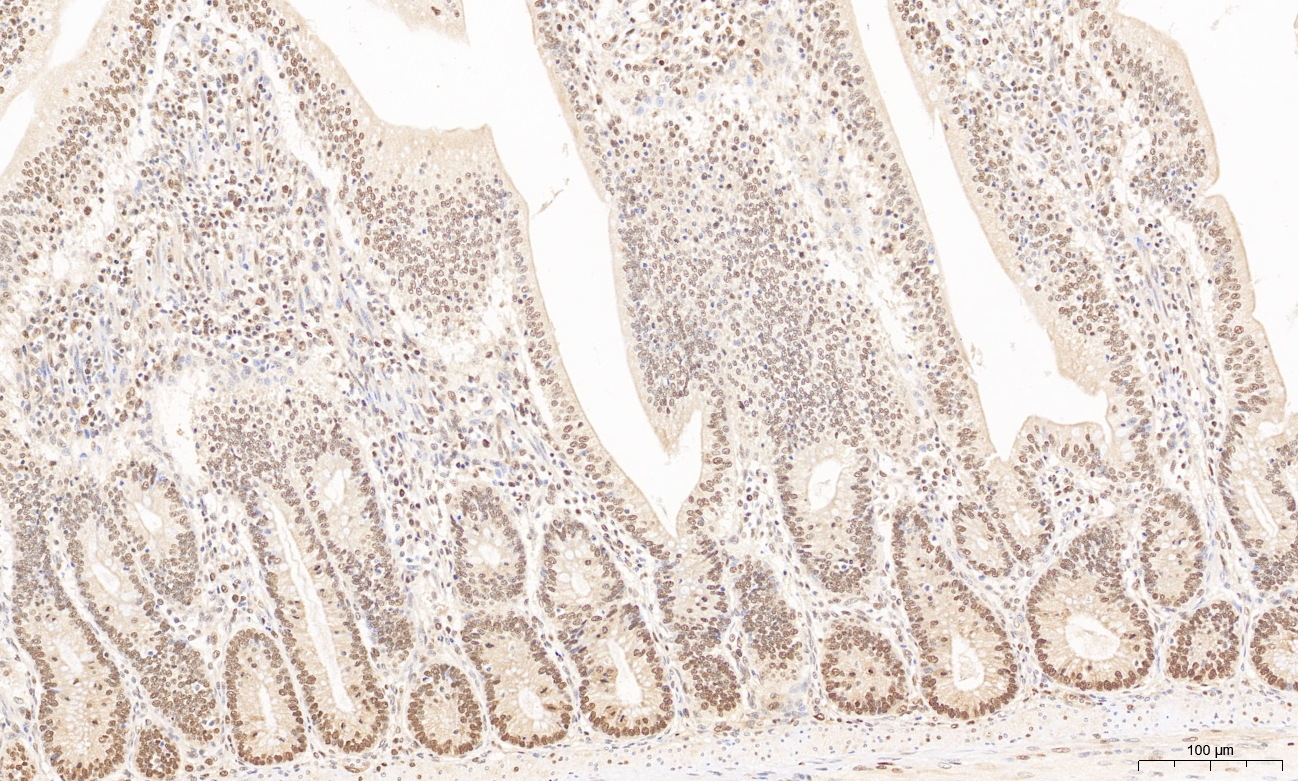

Supplement: Supplementary file 16 [file Data_Sheet_11.ZIP › Ileal PCNA Immunohistochemical staining 2/NE+TA400 group/4.jpg]

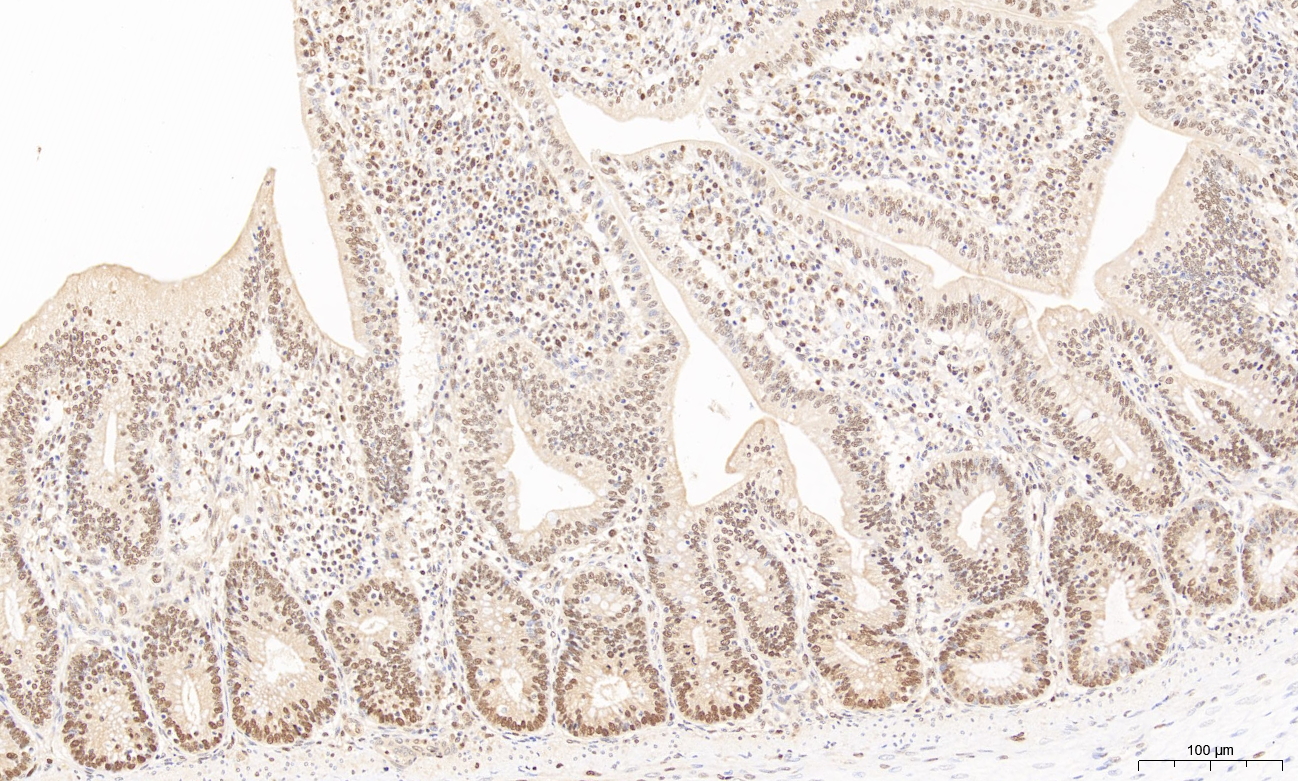

Supplement: Supplementary file 16 [file Data_Sheet_11.ZIP › Ileal PCNA Immunohistochemical staining 2/NE+TA400 group/5.jpg]

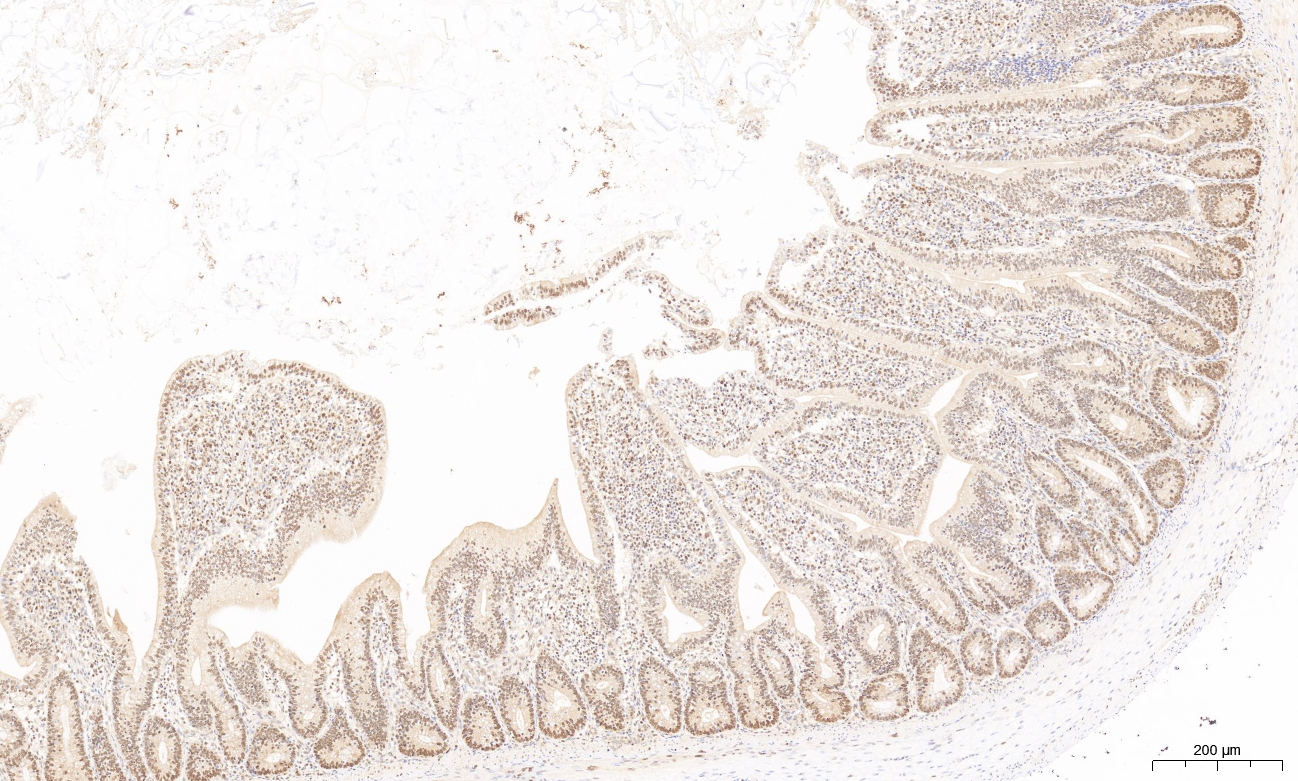

Supplement: Supplementary file 16 [file Data_Sheet_11.ZIP › Ileal PCNA Immunohistochemical staining 2/NE+TA400 group/6.jpg]

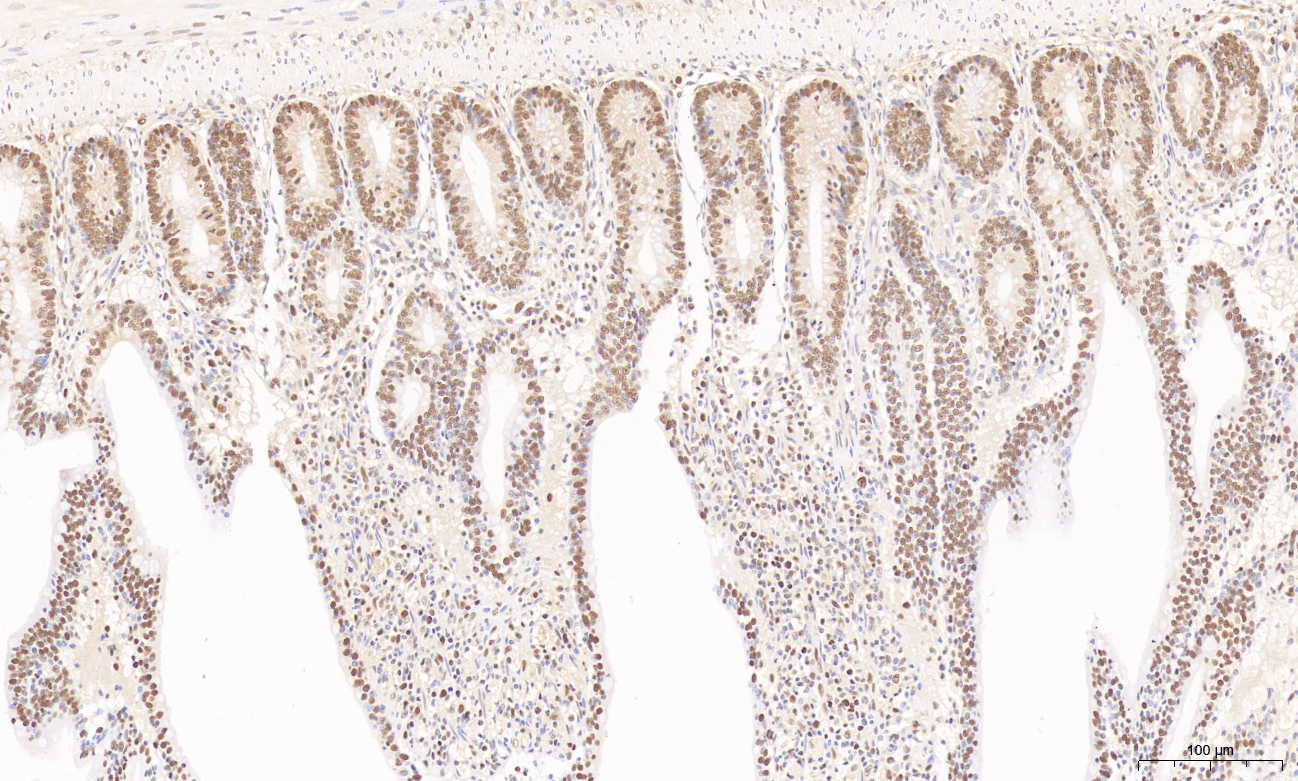

Supplement: Supplementary file 16 [file Data_Sheet_11.ZIP › Ileal PCNA Immunohistochemical staining 2/NE+TA400 group/7.jpg]

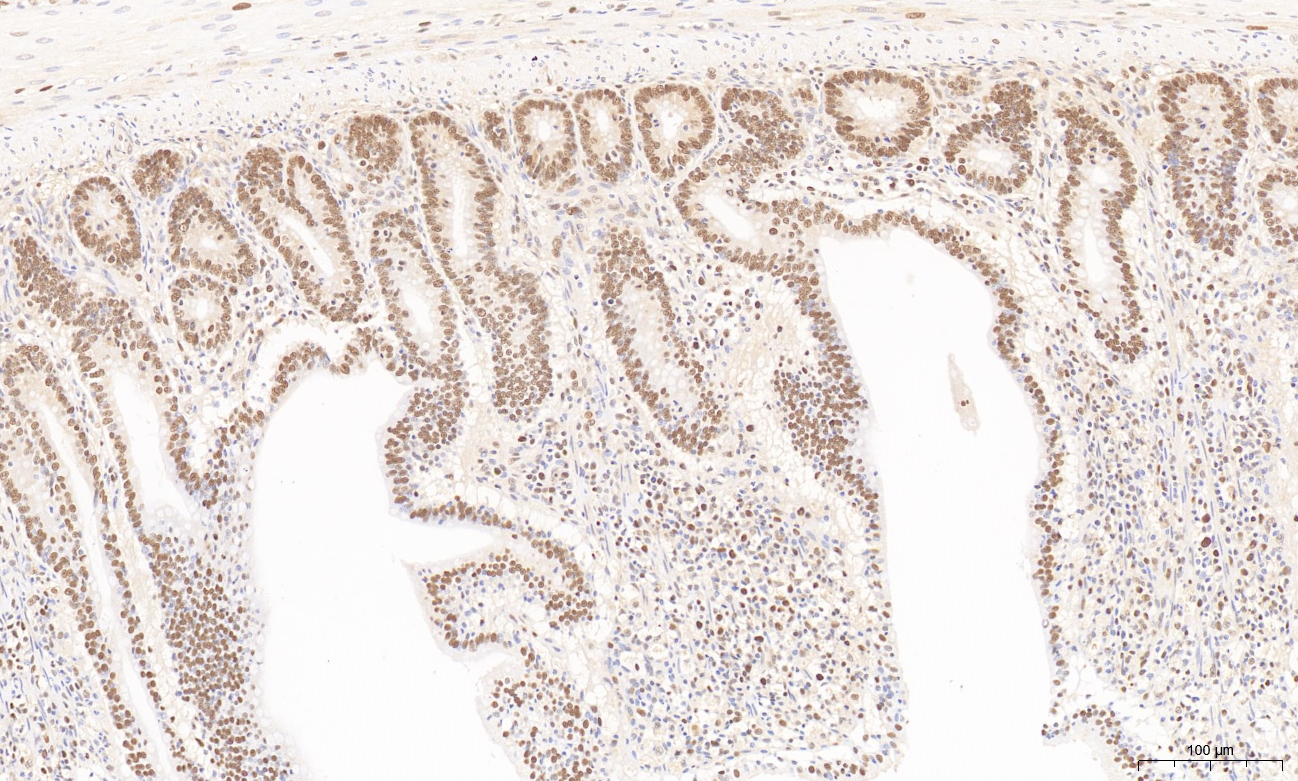

Supplement: Supplementary file 16 [file Data_Sheet_11.ZIP › Ileal PCNA Immunohistochemical staining 2/NE+TA400 group/8.jpg]

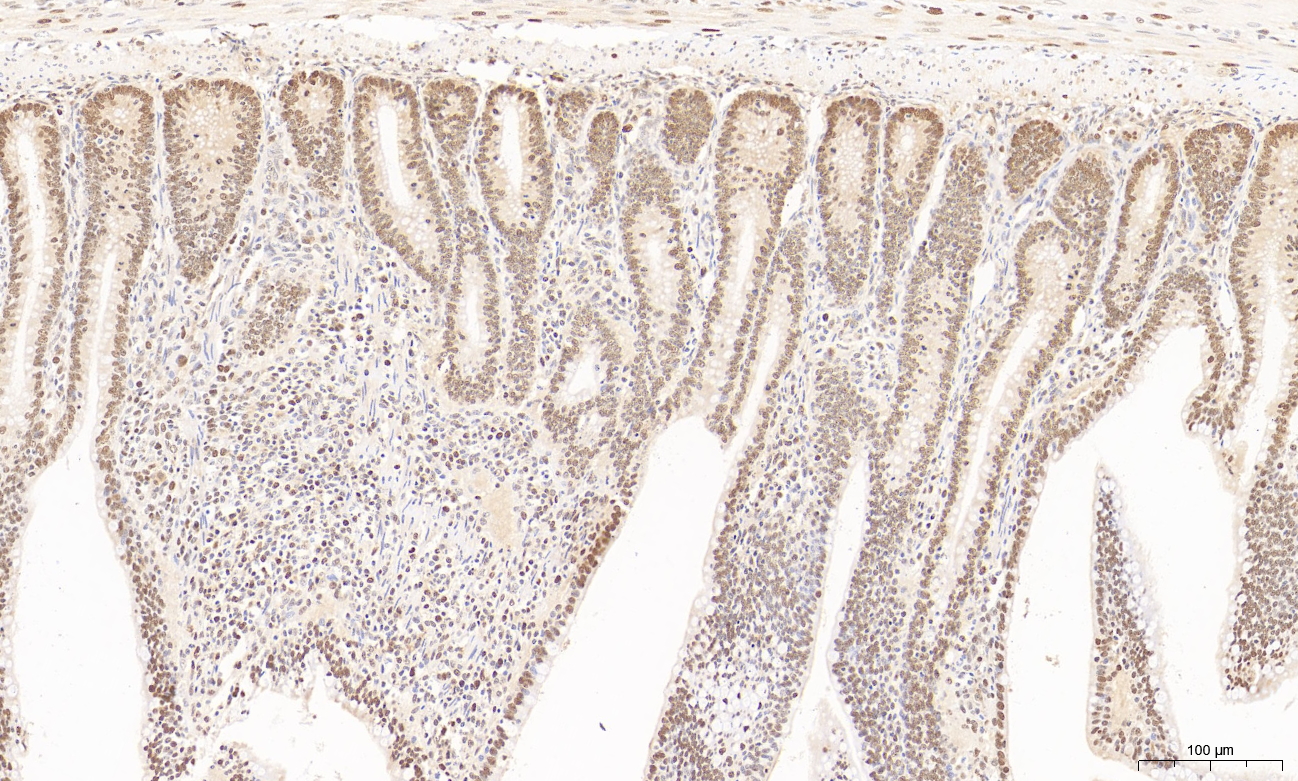

Supplement: Supplementary file 16 [file Data_Sheet_11.ZIP › Ileal PCNA Immunohistochemical staining 2/NE+TA600 group/1.jpg]

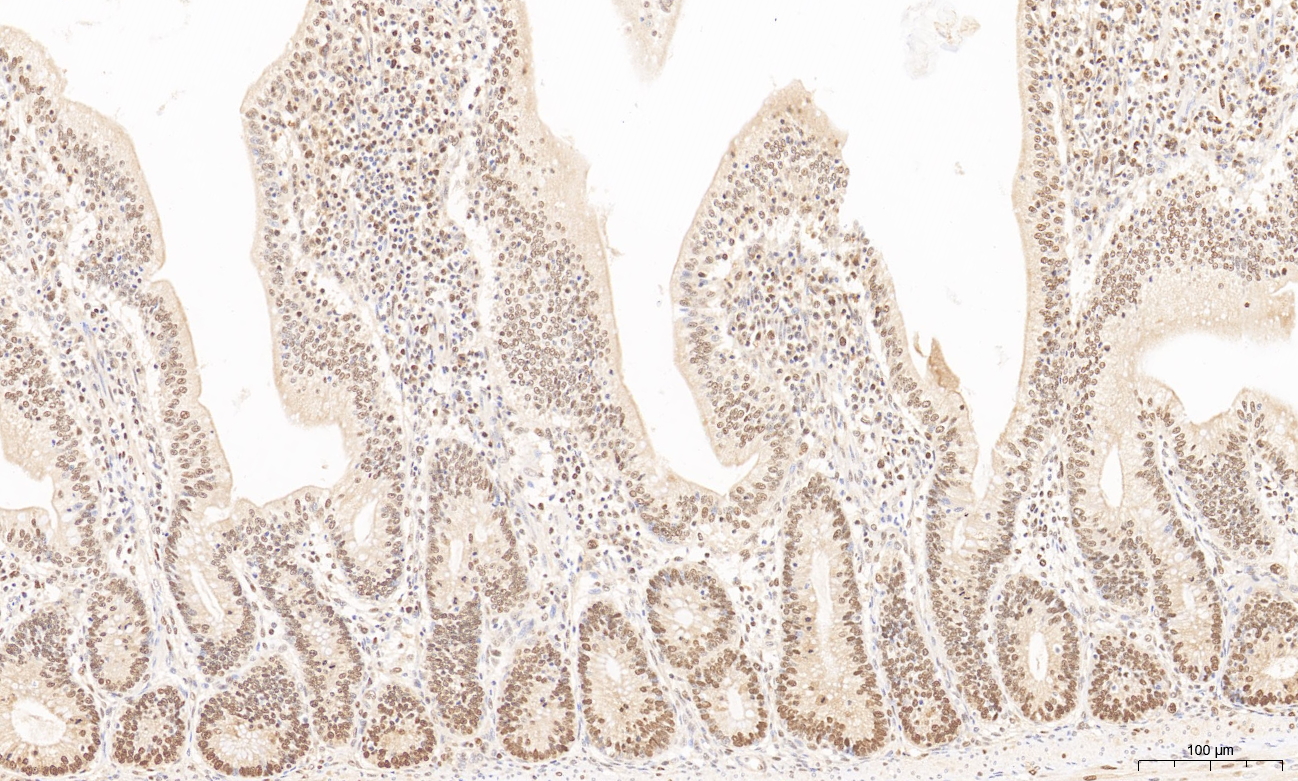

Supplement: Supplementary file 16 [file Data_Sheet_11.ZIP › Ileal PCNA Immunohistochemical staining 2/NE+TA600 group/3-1-5.jpg]

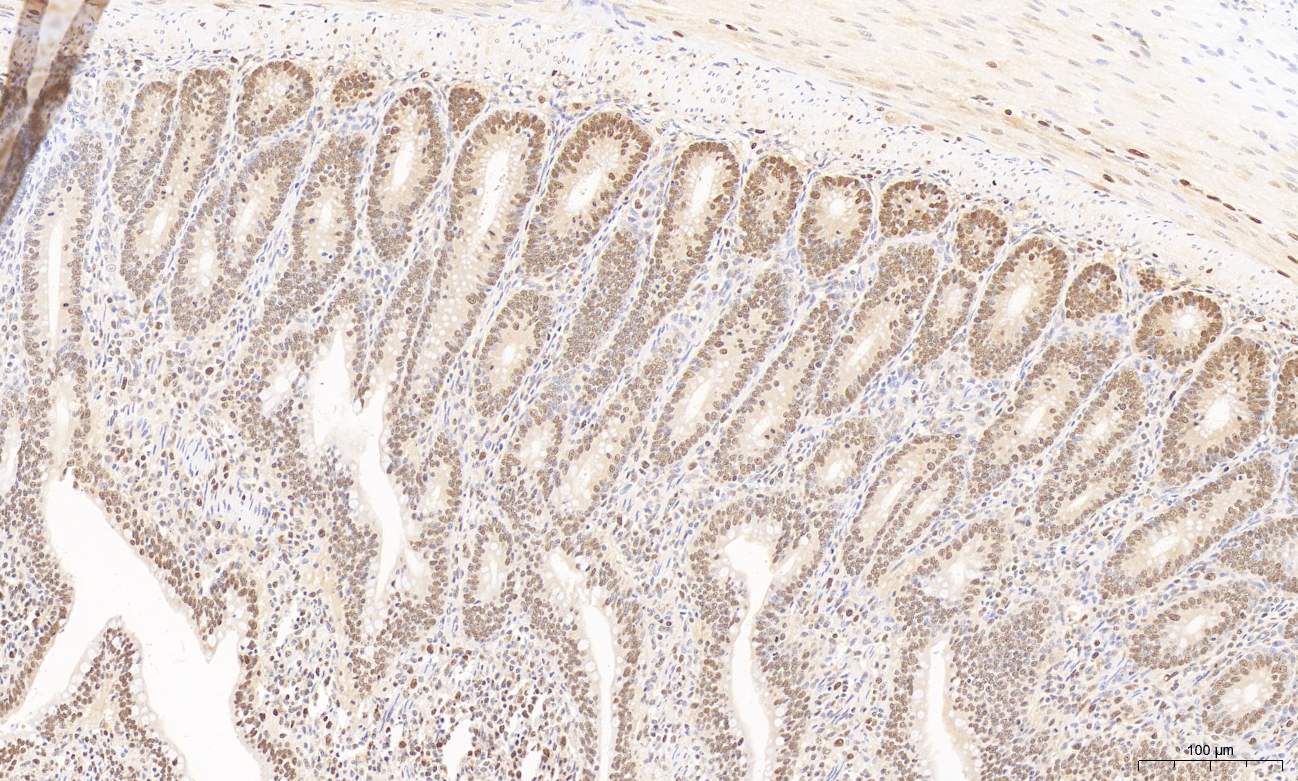

Supplement: Supplementary file 16 [file Data_Sheet_11.ZIP › Ileal PCNA Immunohistochemical staining 2/NE+TA600 group/3.jpg]

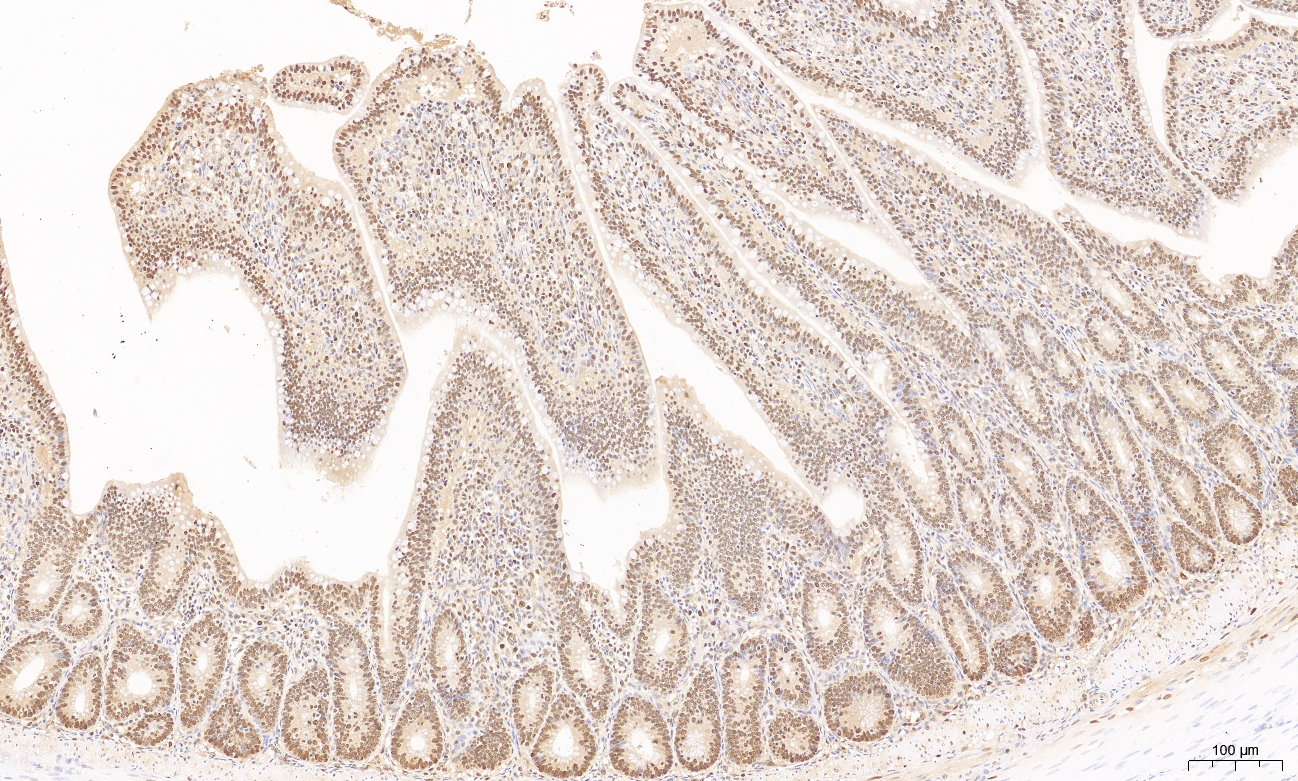

Supplement: Supplementary file 16 [file Data_Sheet_11.ZIP › Ileal PCNA Immunohistochemical staining 2/NE+TA600 group/4.jpg]

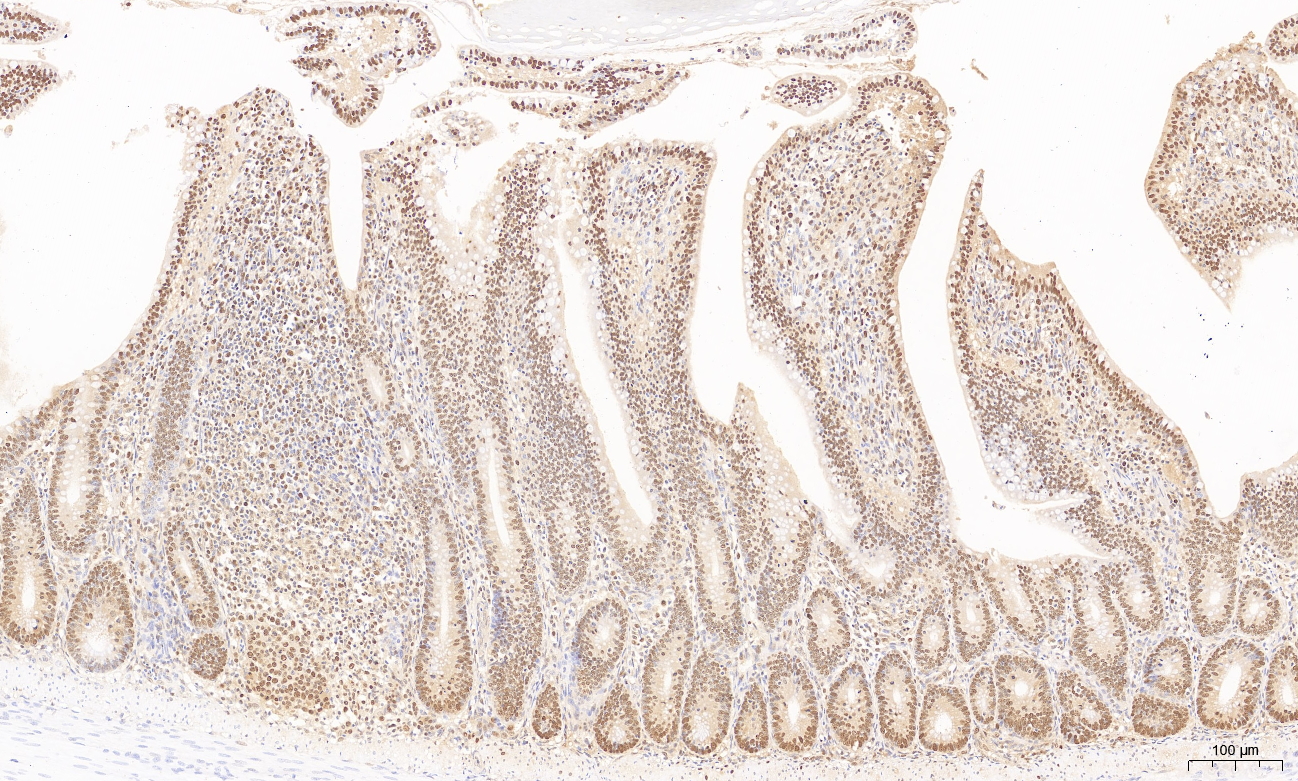

Supplement: Supplementary file 16 [file Data_Sheet_11.ZIP › Ileal PCNA Immunohistochemical staining 2/NE+TA600 group/5.jpg]

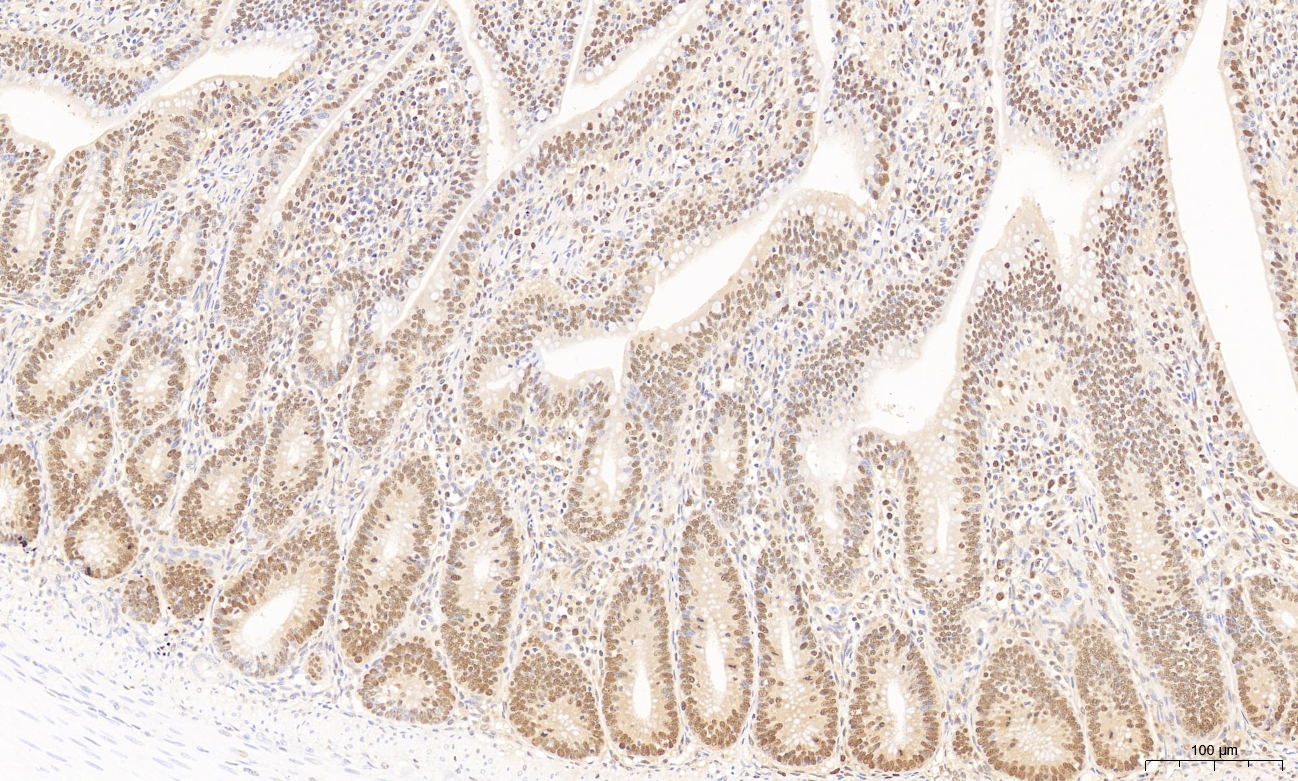

Supplement: Supplementary file 16 [file Data_Sheet_11.ZIP › Ileal PCNA Immunohistochemical staining 2/NE+TA600 group/6.jpg]

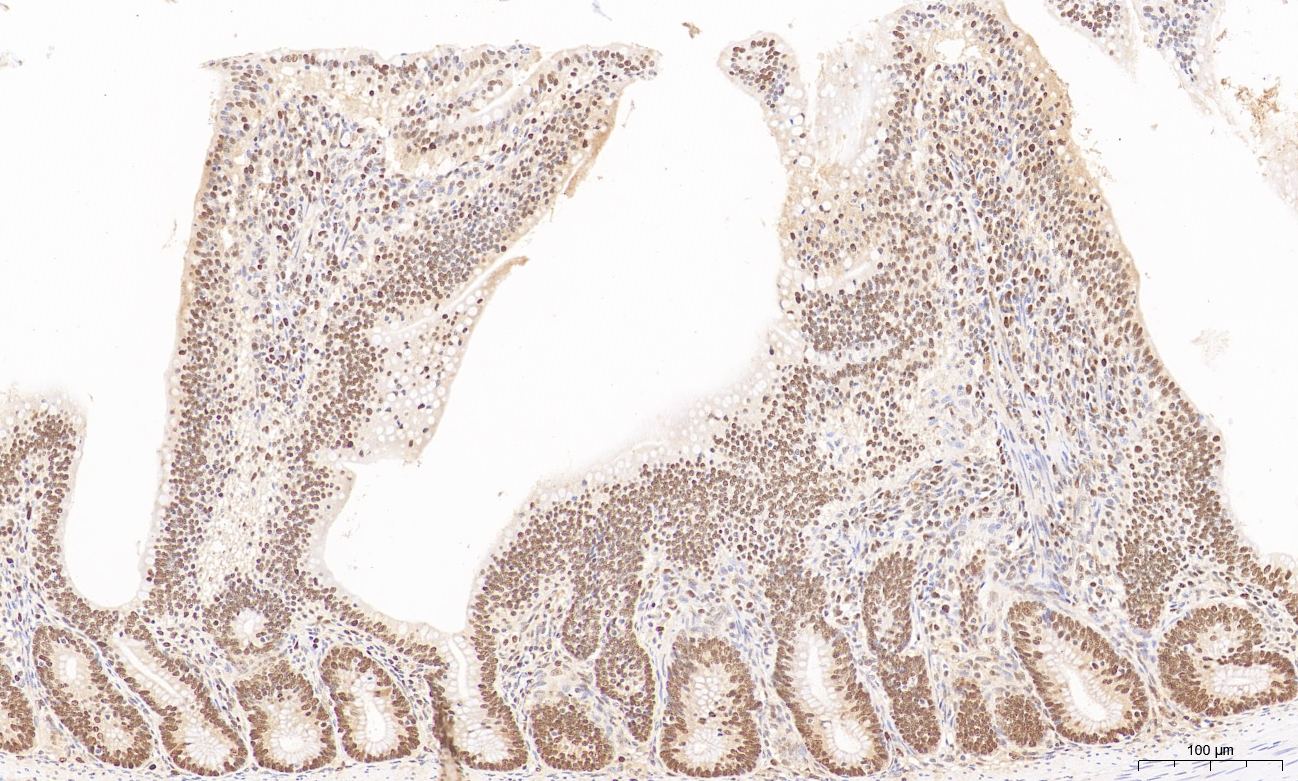

Supplement: Supplementary file 16 [file Data_Sheet_11.ZIP › Ileal PCNA Immunohistochemical staining 2/NE+TA600 group/7.jpg]

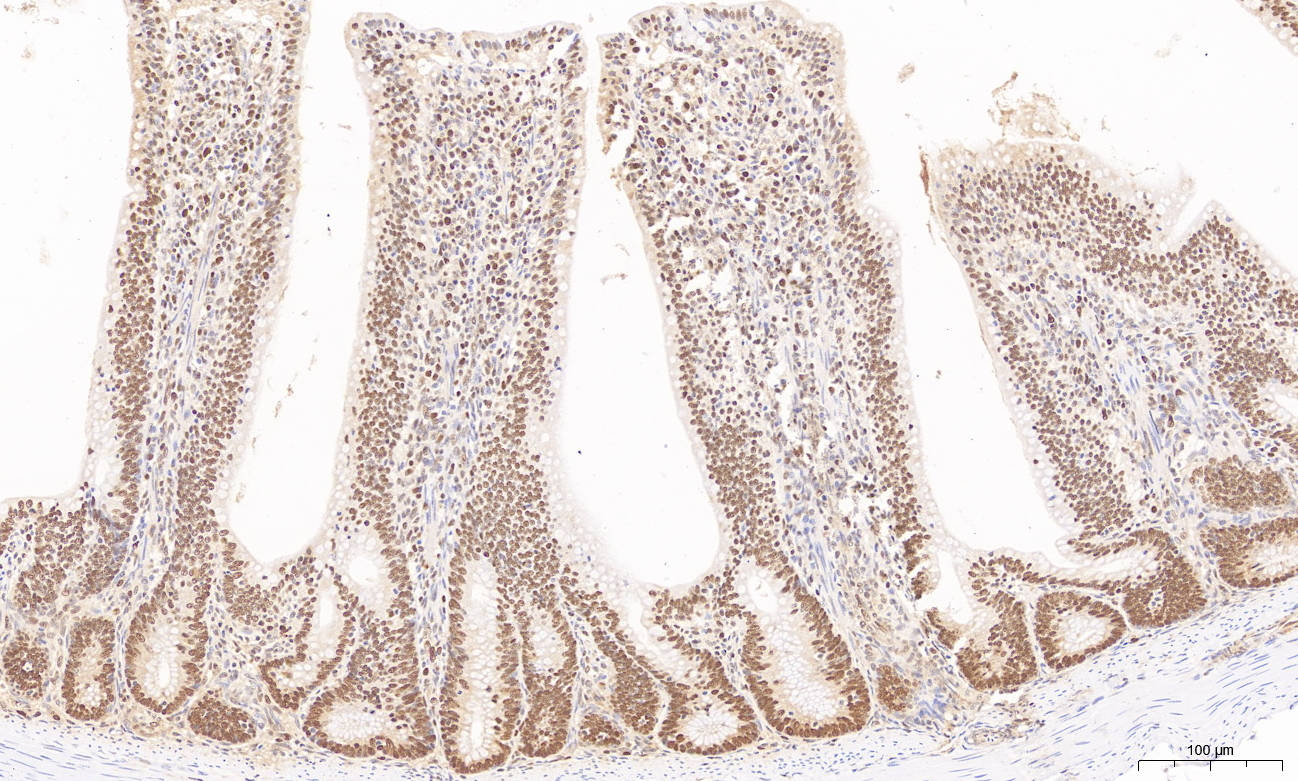

Supplement: Supplementary file 16 [file Data_Sheet_11.ZIP › Ileal PCNA Immunohistochemical staining 2/NE+TA600 group/8.jpg]

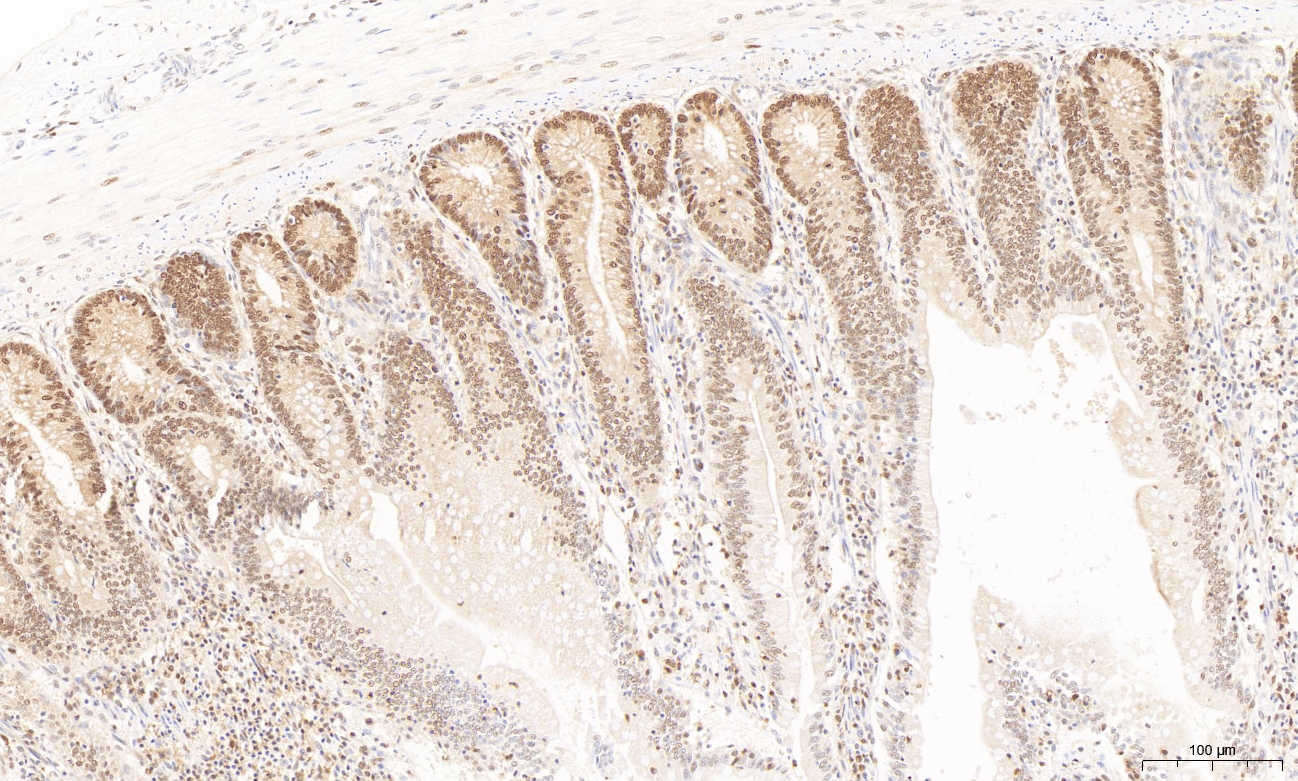

Supplement: Supplementary file 17 [file Data_Sheet_12.ZIP › Jejunal PCNA Immunohistochemical staining 1/CON group/1.jpg]

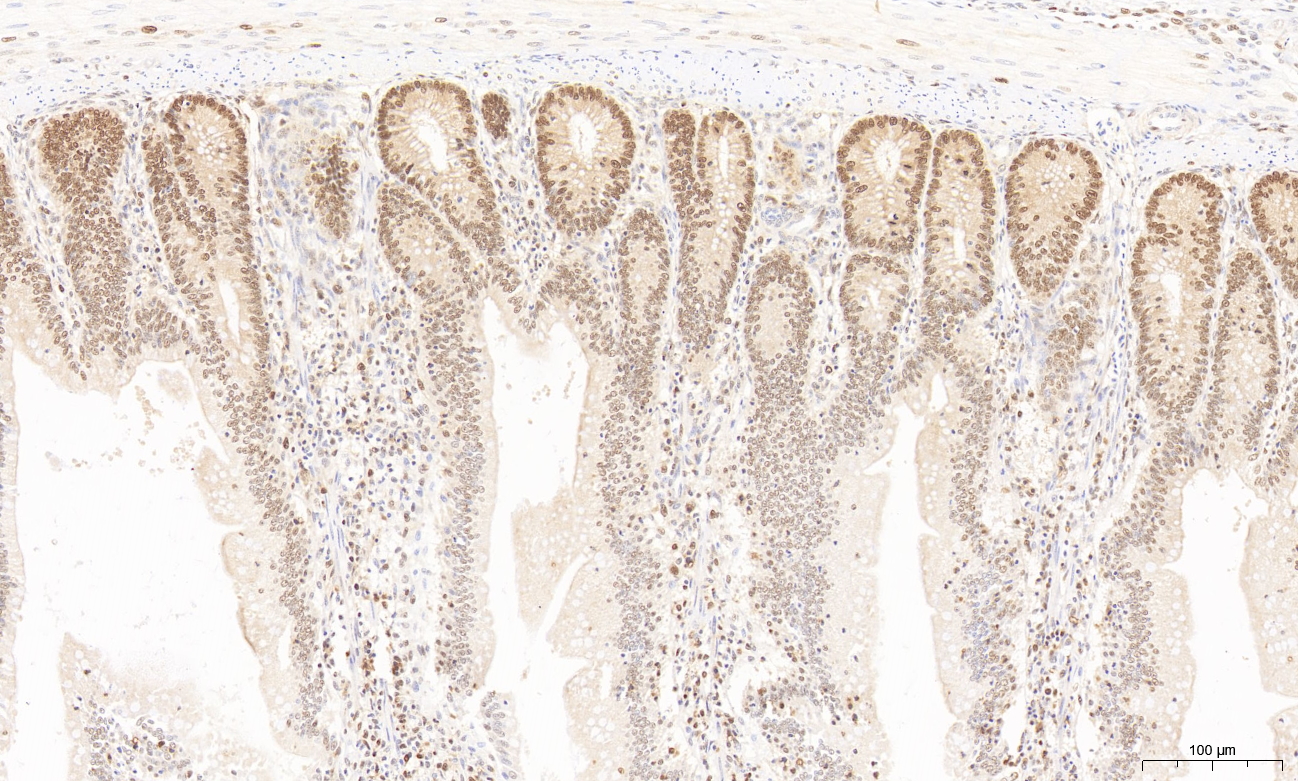

Supplement: Supplementary file 17 [file Data_Sheet_12.ZIP › Jejunal PCNA Immunohistochemical staining 1/CON group/2.jpg]

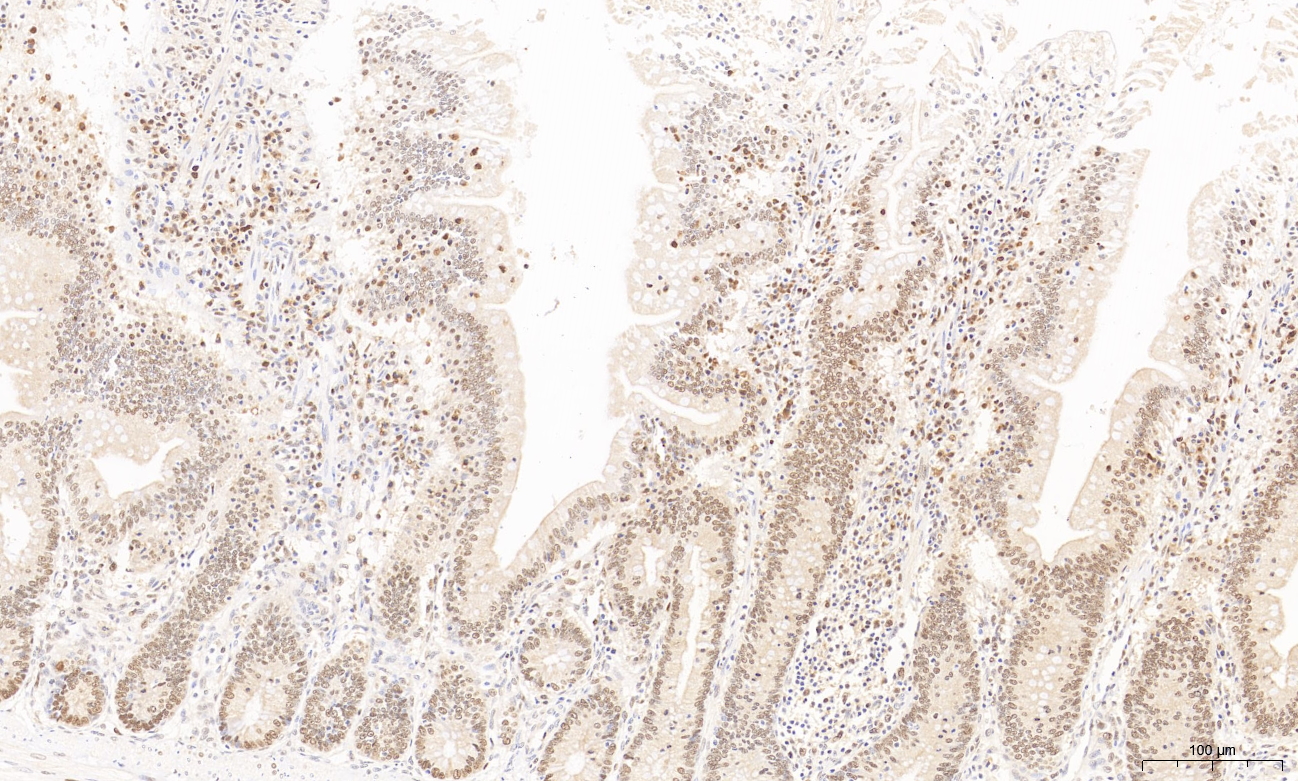

Supplement: Supplementary file 17 [file Data_Sheet_12.ZIP › Jejunal PCNA Immunohistochemical staining 1/CON group/3.jpg]

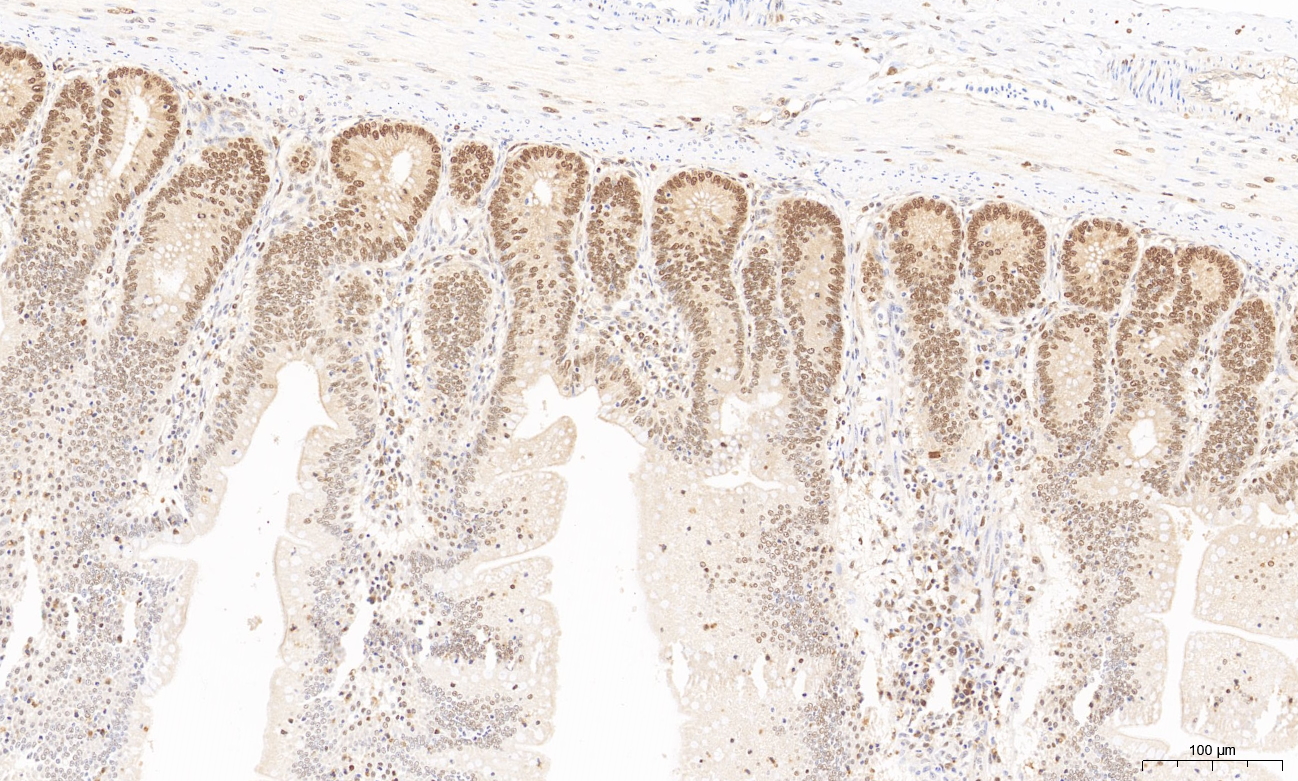

Supplement: Supplementary file 17 [file Data_Sheet_12.ZIP › Jejunal PCNA Immunohistochemical staining 1/CON group/4.jpg]

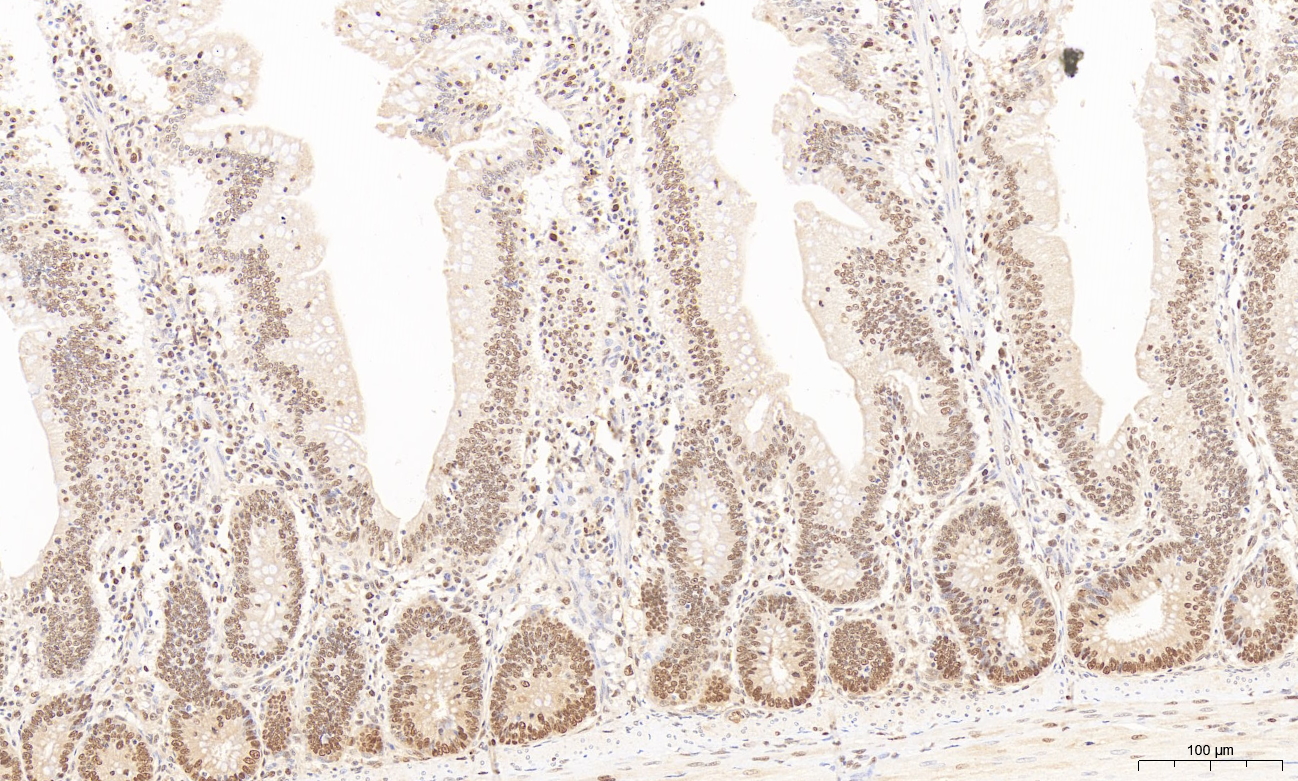

Supplement: Supplementary file 17 [file Data_Sheet_12.ZIP › Jejunal PCNA Immunohistochemical staining 1/CON group/5.jpg]

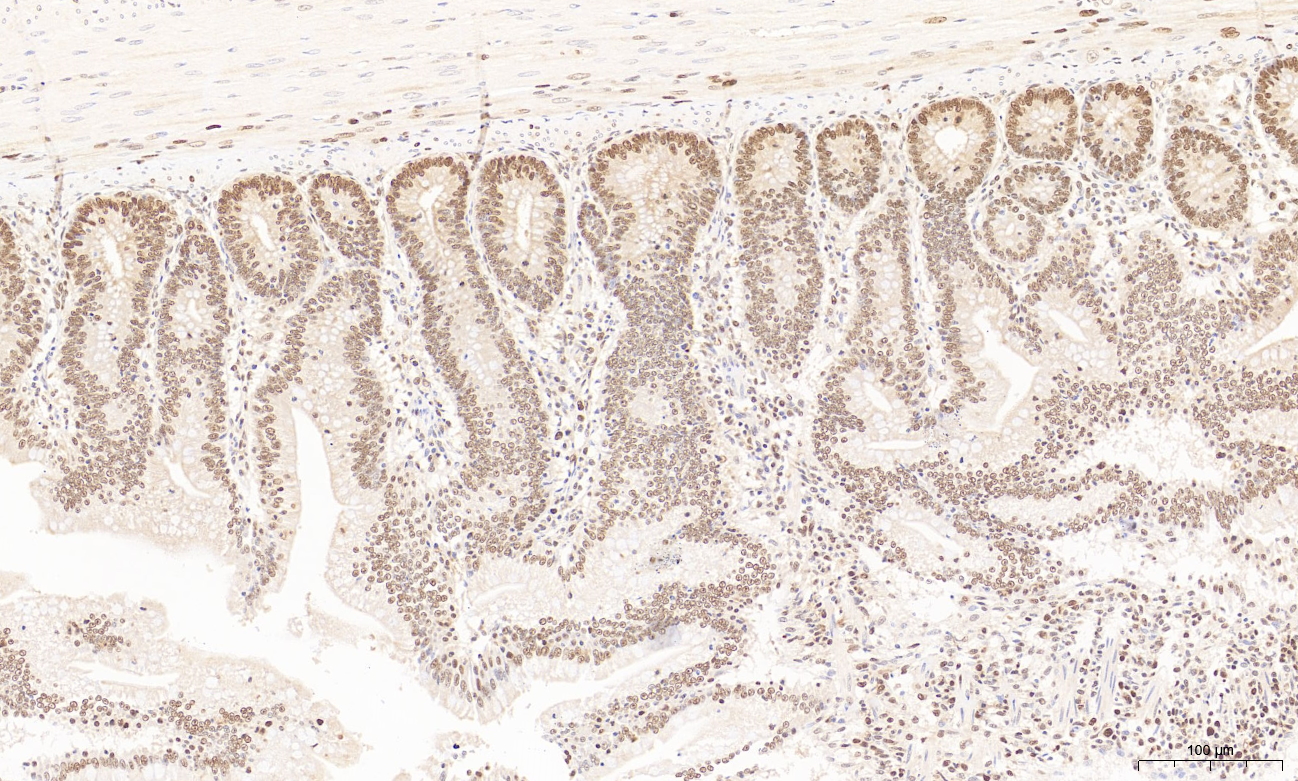

Supplement: Supplementary file 17 [file Data_Sheet_12.ZIP › Jejunal PCNA Immunohistochemical staining 1/CON group/6.jpg]

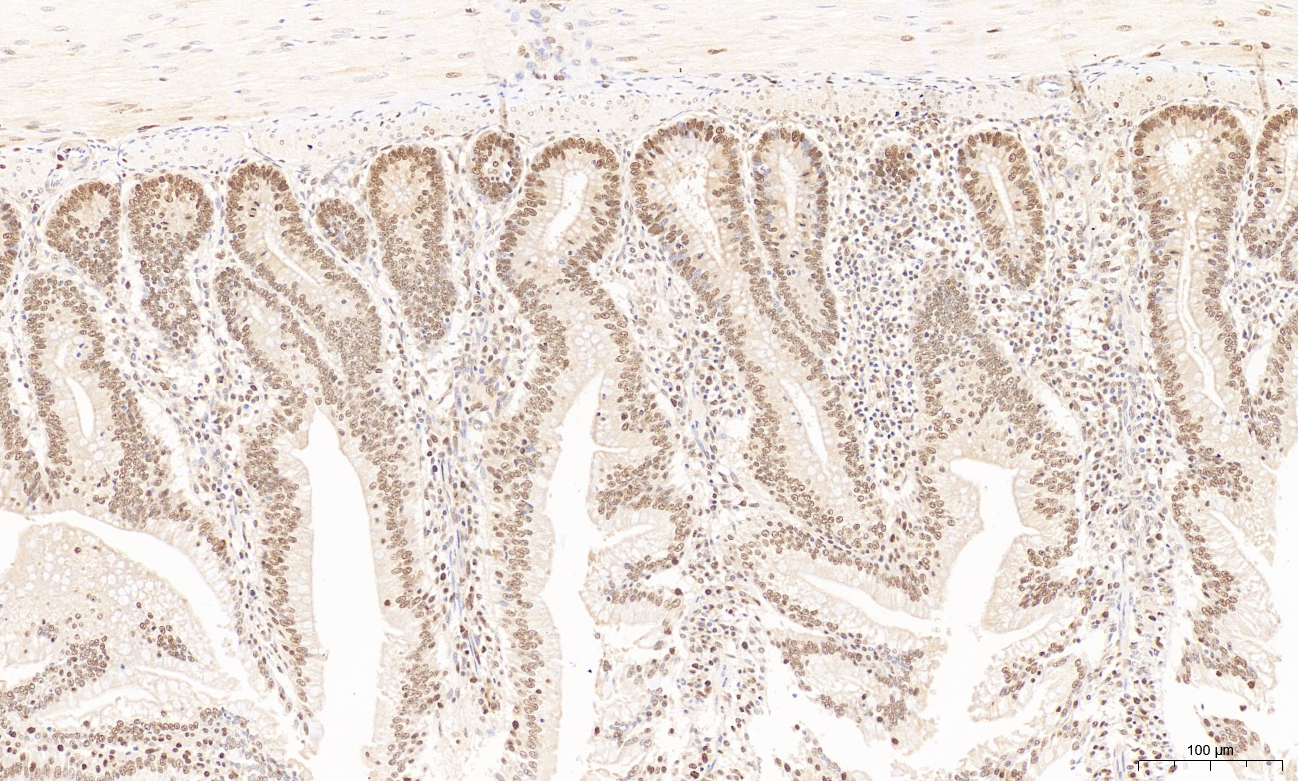

Supplement: Supplementary file 17 [file Data_Sheet_12.ZIP › Jejunal PCNA Immunohistochemical staining 1/CON group/7.jpg]

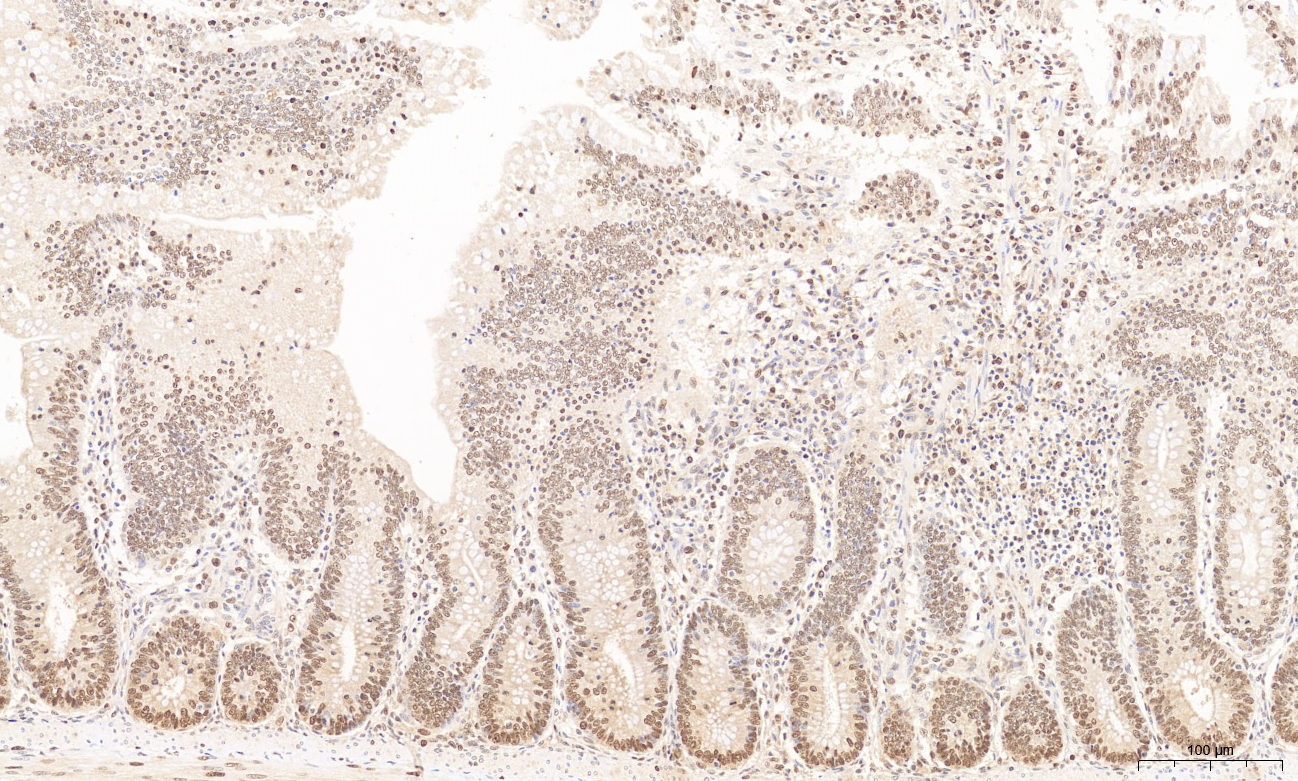

Supplement: Supplementary file 17 [file Data_Sheet_12.ZIP › Jejunal PCNA Immunohistochemical staining 1/CON group/8.jpg]

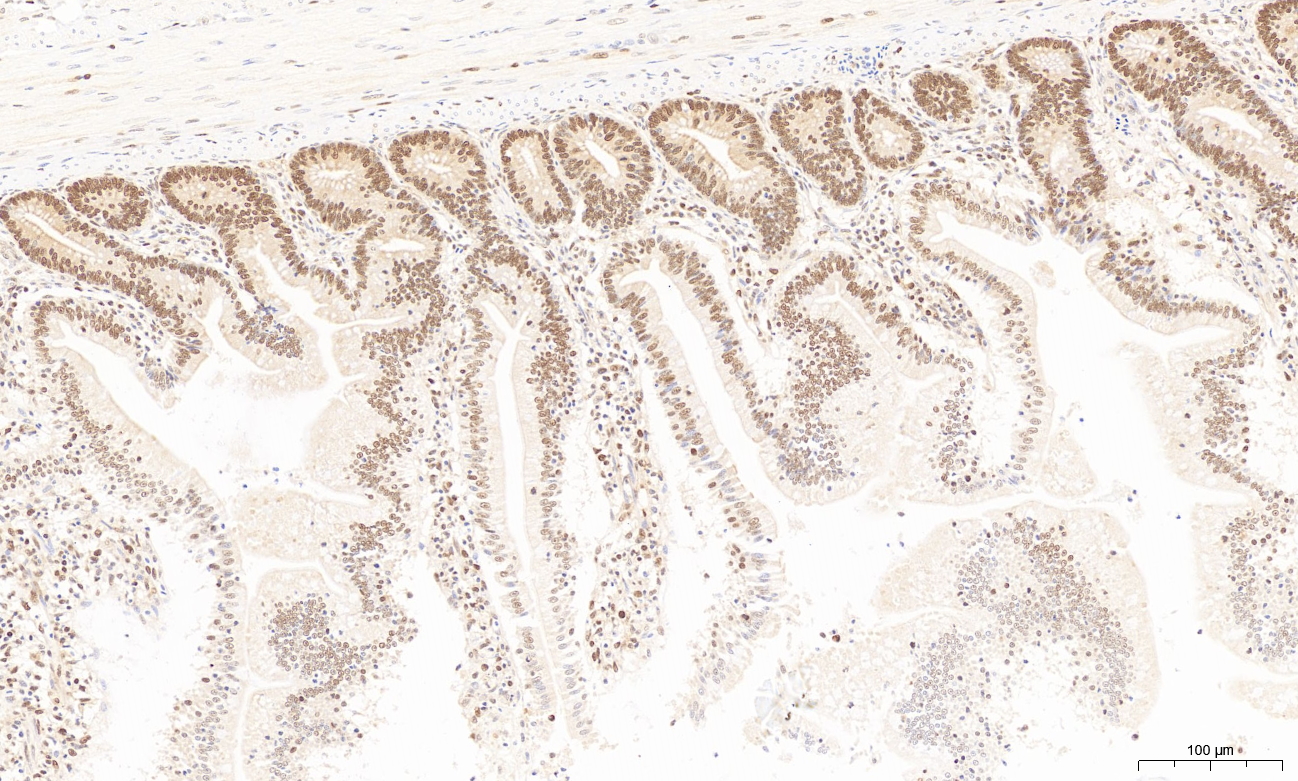

Supplement: Supplementary file 17 [file Data_Sheet_12.ZIP › Jejunal PCNA Immunohistochemical staining 1/NE group/1.jpg]

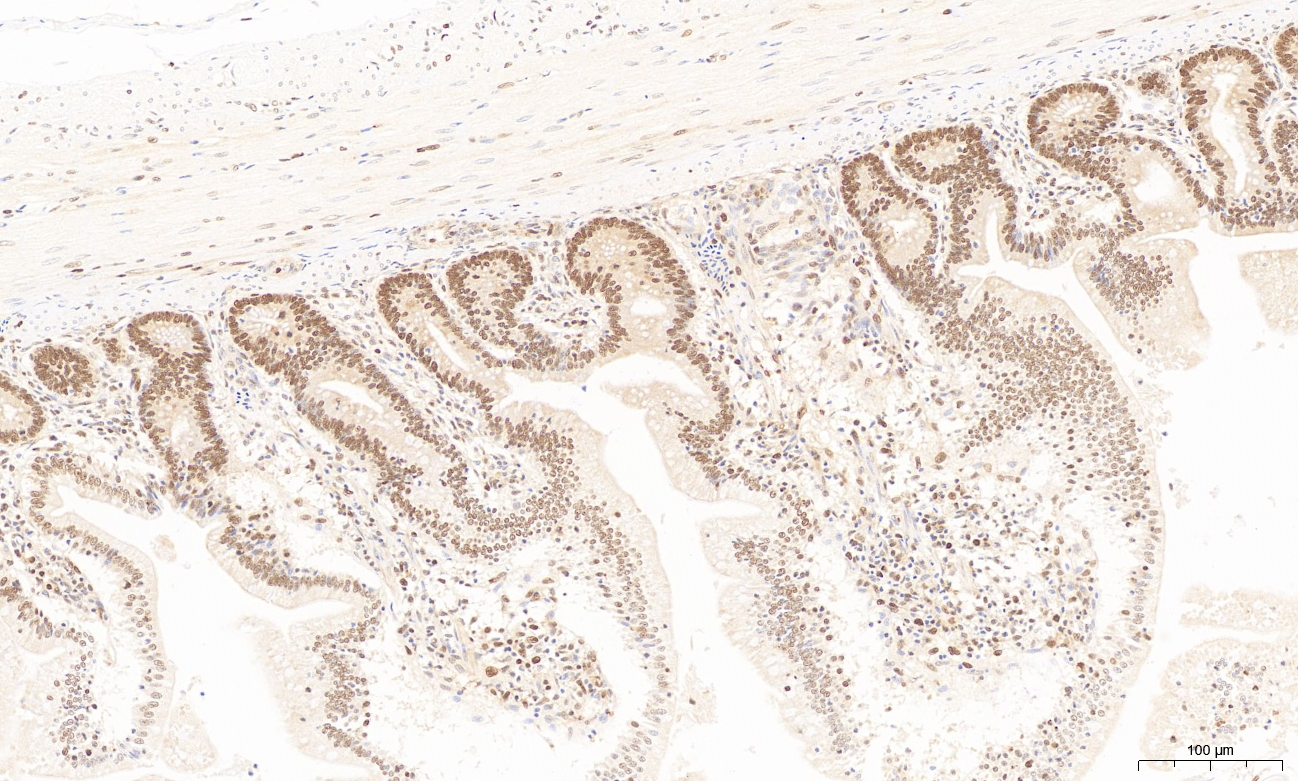

Supplement: Supplementary file 17 [file Data_Sheet_12.ZIP › Jejunal PCNA Immunohistochemical staining 1/NE group/2.jpg]

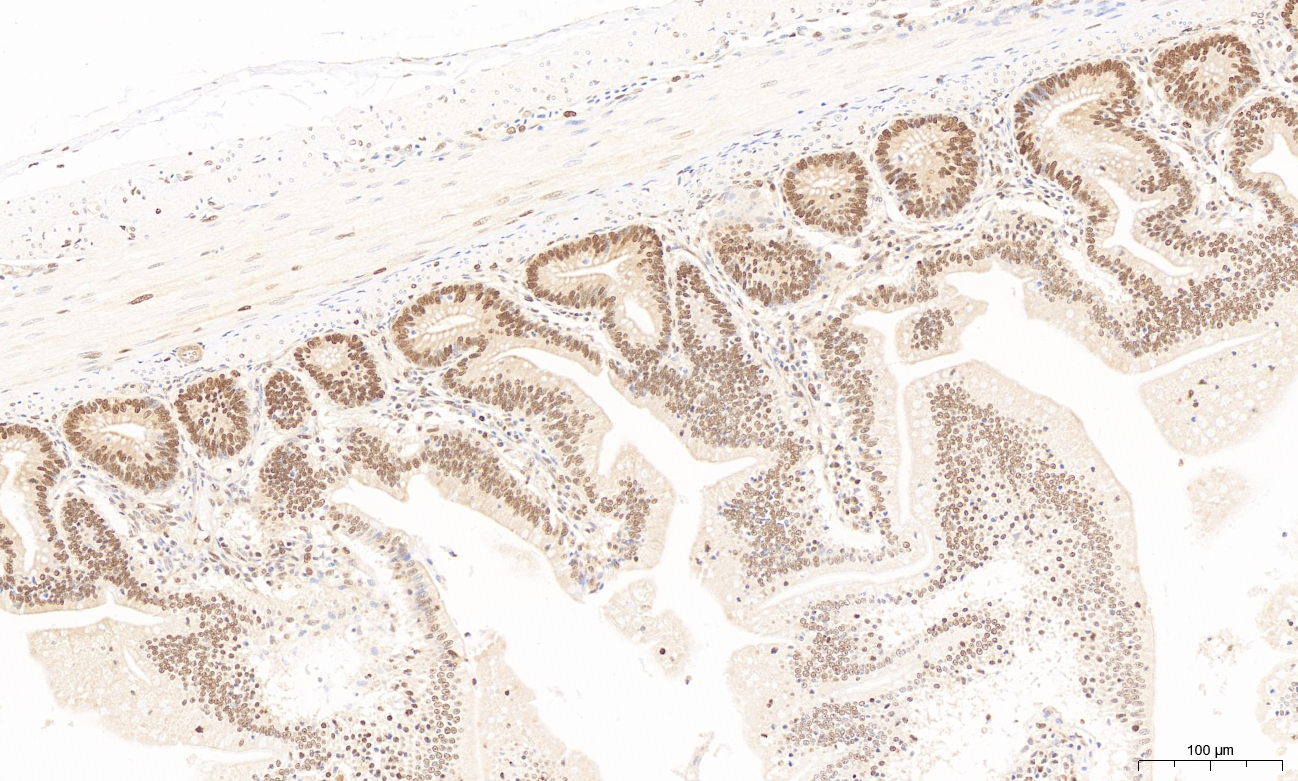

Supplement: Supplementary file 17 [file Data_Sheet_12.ZIP › Jejunal PCNA Immunohistochemical staining 1/NE group/3.jpg]

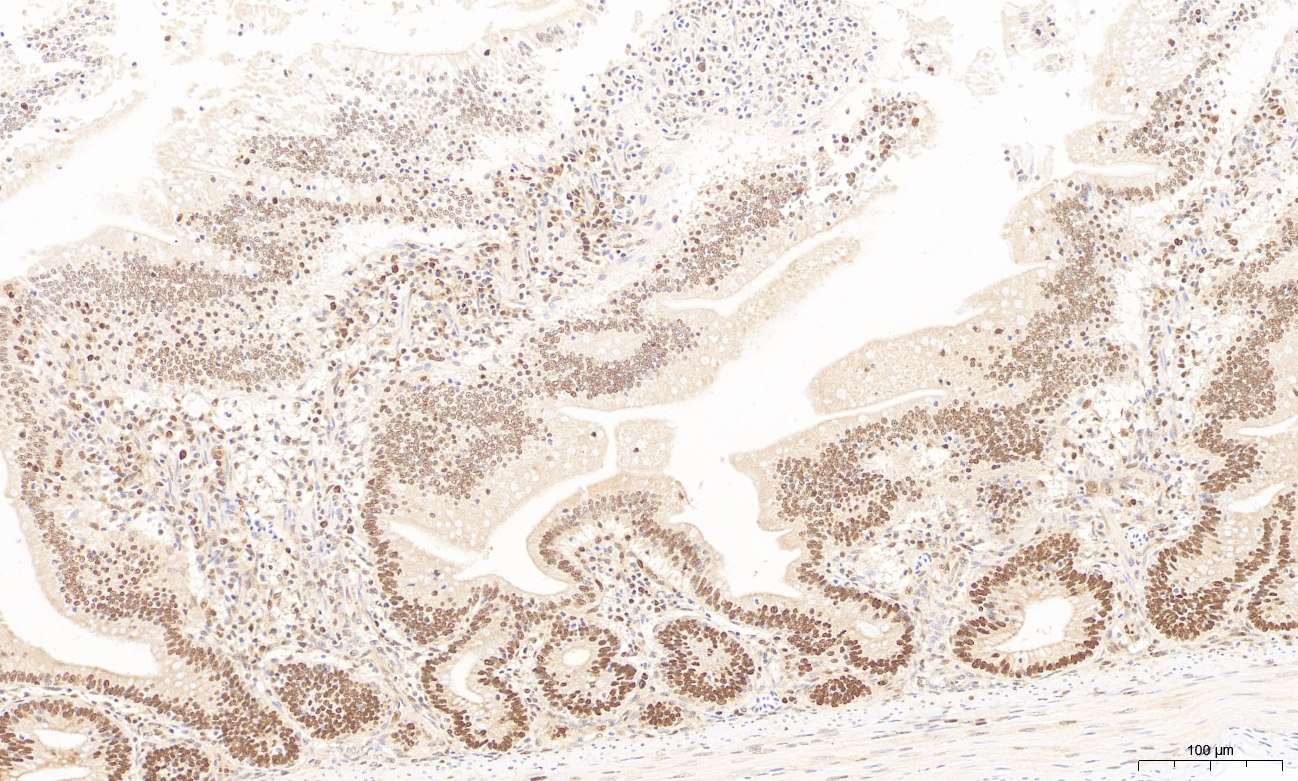

Supplement: Supplementary file 17 [file Data_Sheet_12.ZIP › Jejunal PCNA Immunohistochemical staining 1/NE group/4.jpg]

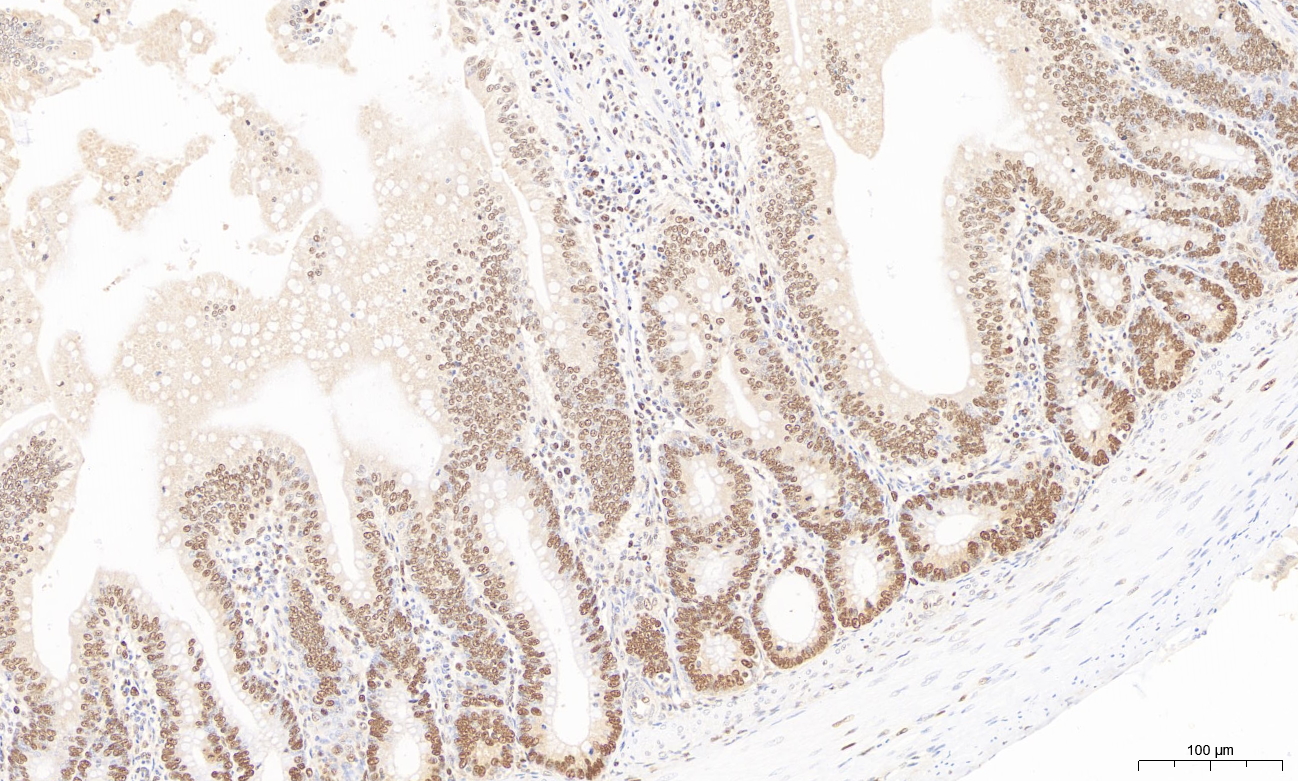

Supplement: Supplementary file 17 [file Data_Sheet_12.ZIP › Jejunal PCNA Immunohistochemical staining 1/NE group/5.jpg]

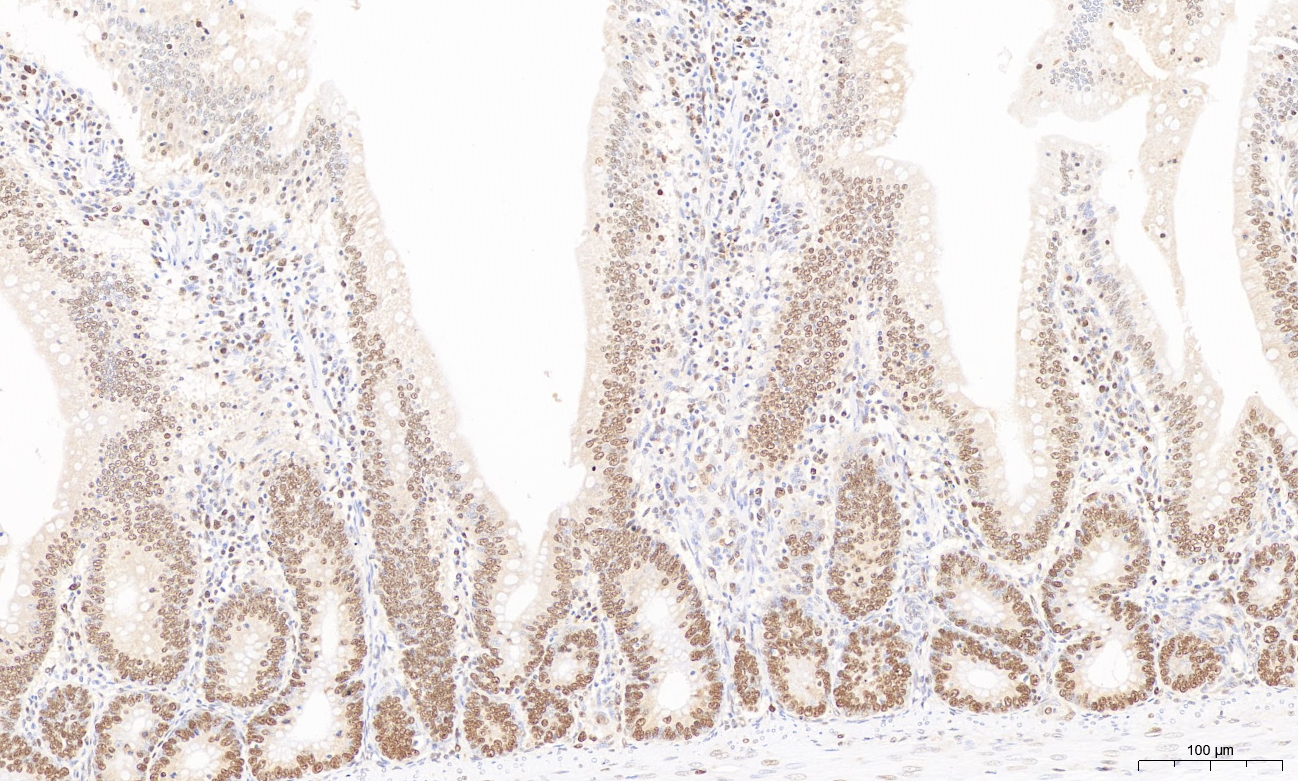

Supplement: Supplementary file 17 [file Data_Sheet_12.ZIP › Jejunal PCNA Immunohistochemical staining 1/NE group/6.jpg]

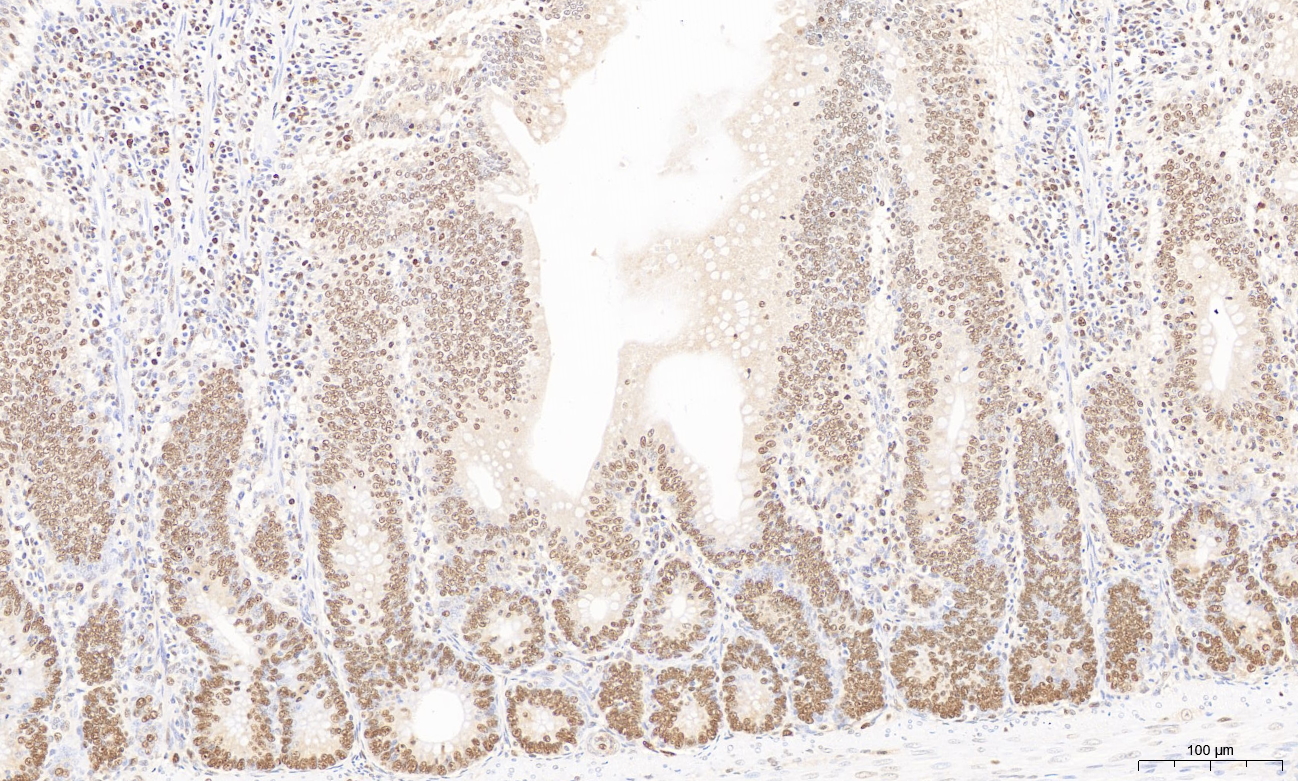

Supplement: Supplementary file 17 [file Data_Sheet_12.ZIP › Jejunal PCNA Immunohistochemical staining 1/NE group/7.jpg]

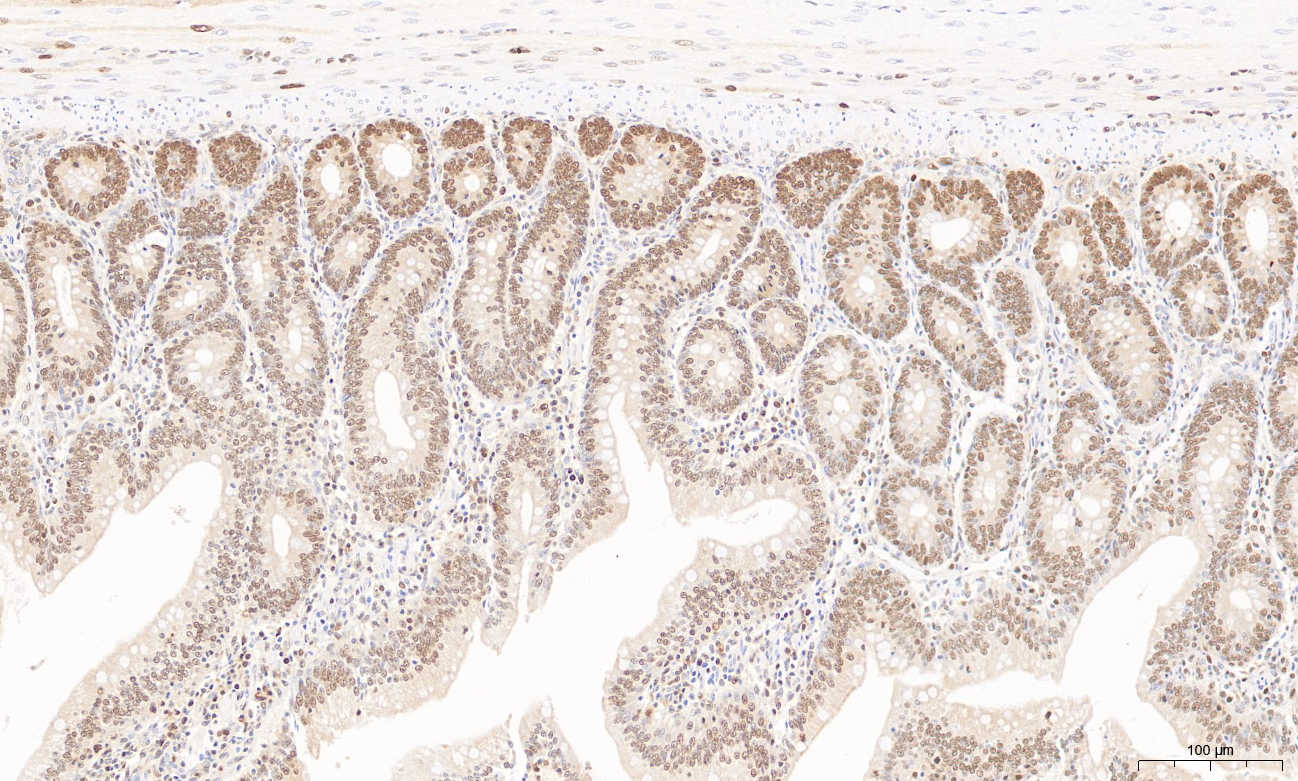

Supplement: Supplementary file 17 [file Data_Sheet_12.ZIP › Jejunal PCNA Immunohistochemical staining 1/NE group/8.jpg]

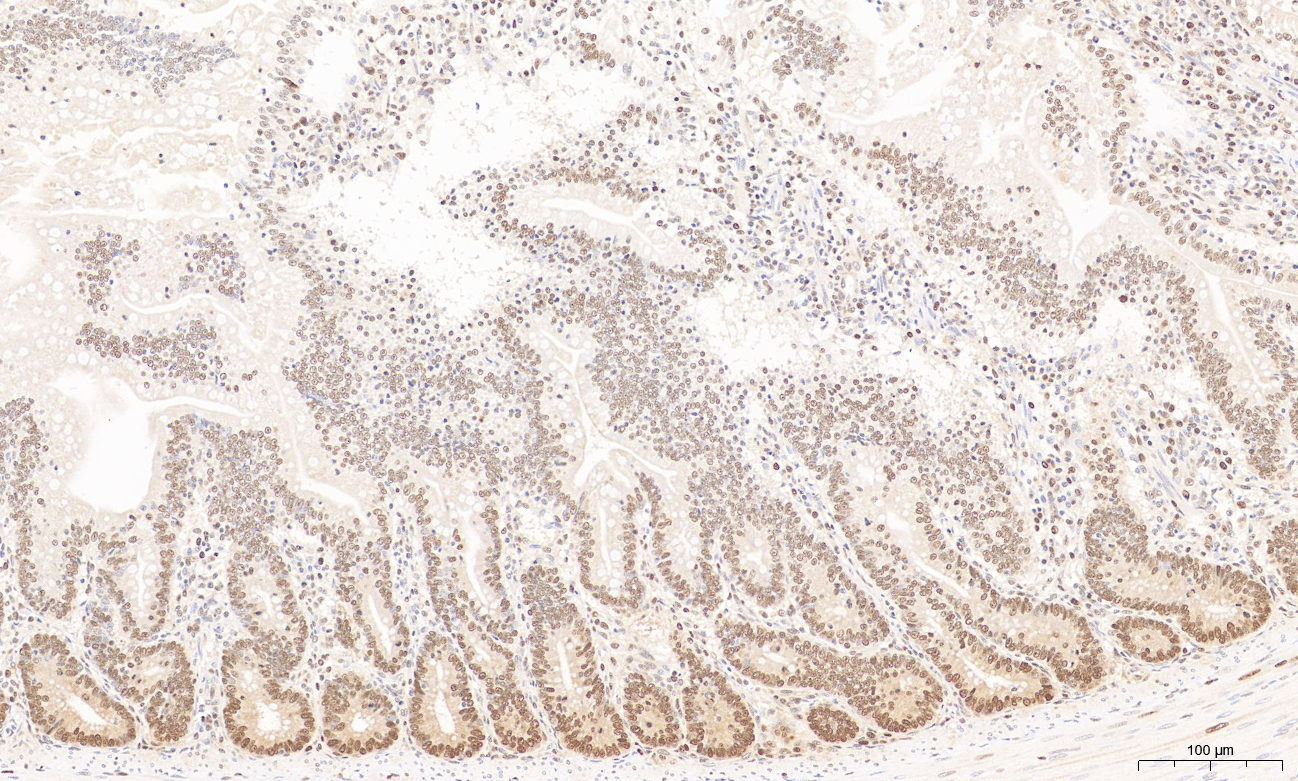

Supplement: Supplementary file 18 [file Data_Sheet_13.ZIP › Jejunal PCNA Immunohistochemical staining 2/NE+TA400 group/1.jpg]

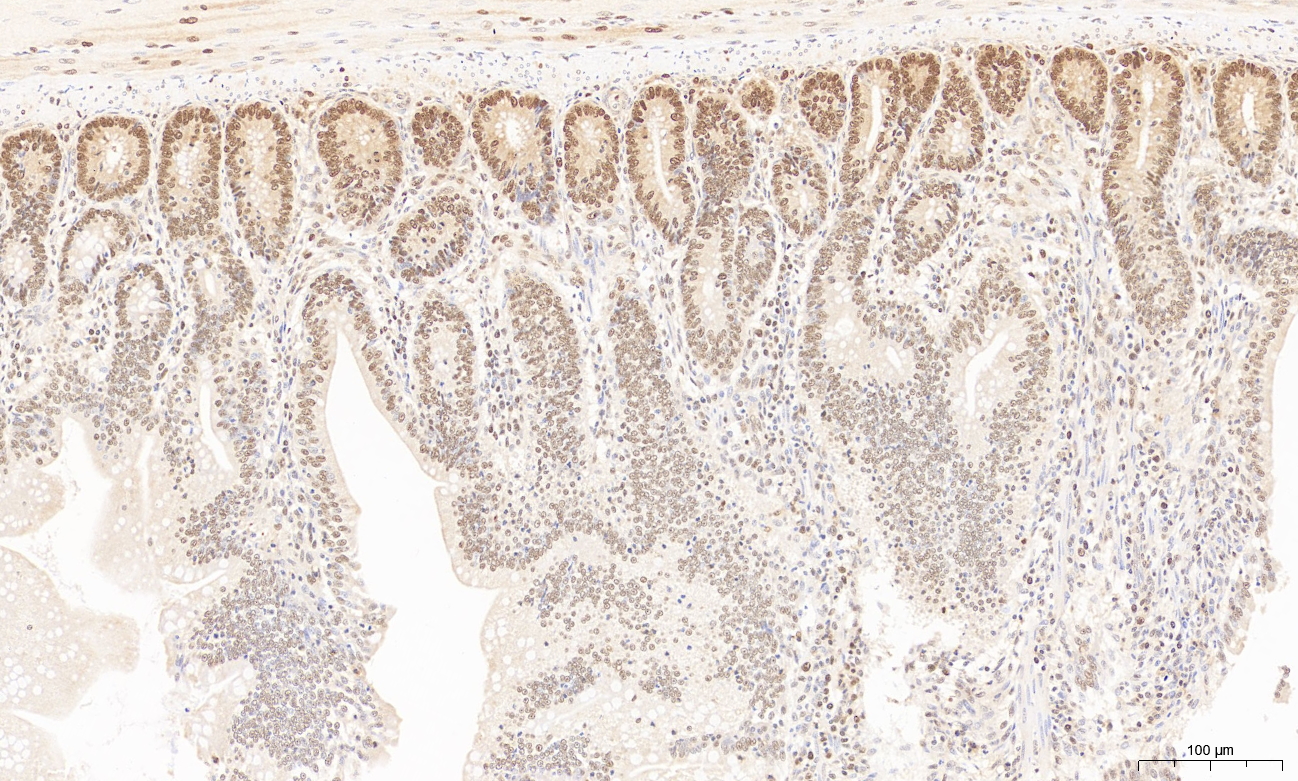

Supplement: Supplementary file 18 [file Data_Sheet_13.ZIP › Jejunal PCNA Immunohistochemical staining 2/NE+TA400 group/2.jpg]

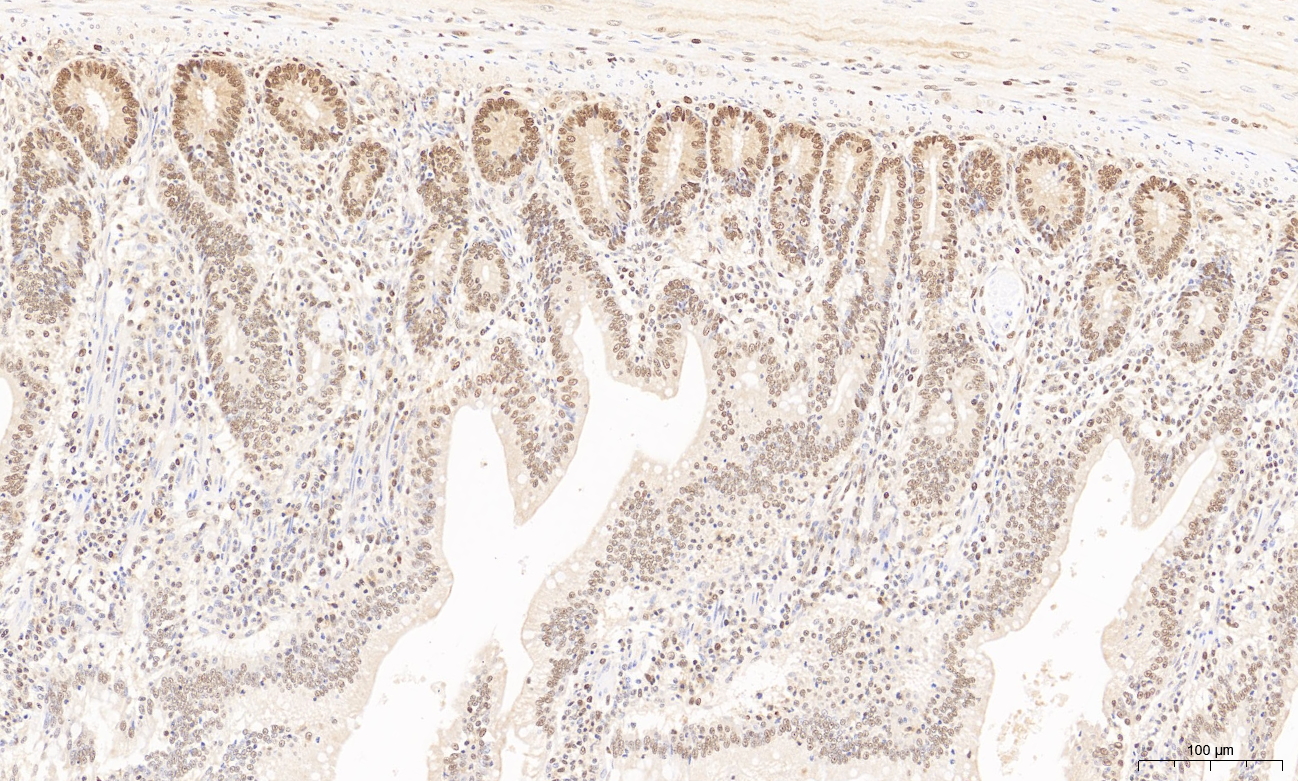

Supplement: Supplementary file 18 [file Data_Sheet_13.ZIP › Jejunal PCNA Immunohistochemical staining 2/NE+TA400 group/3.jpg]

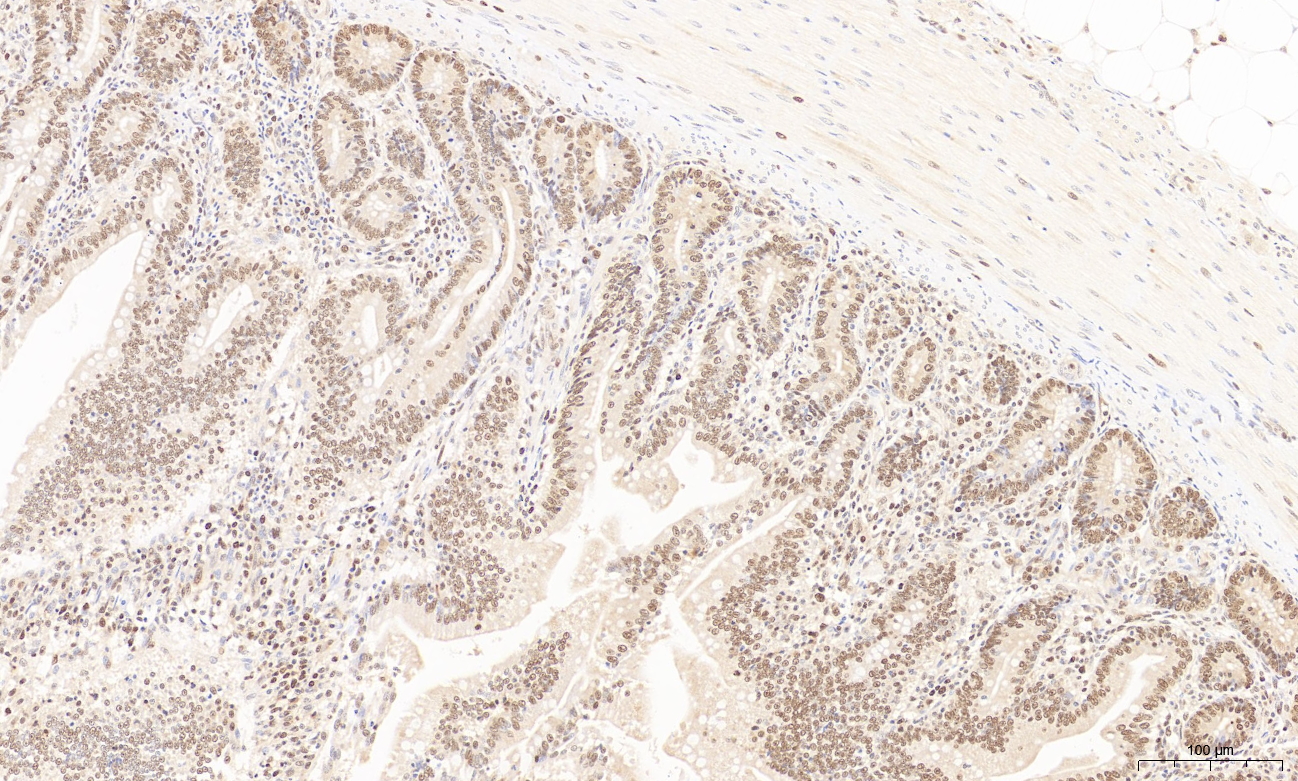

Supplement: Supplementary file 18 [file Data_Sheet_13.ZIP › Jejunal PCNA Immunohistochemical staining 2/NE+TA400 group/4.jpg]

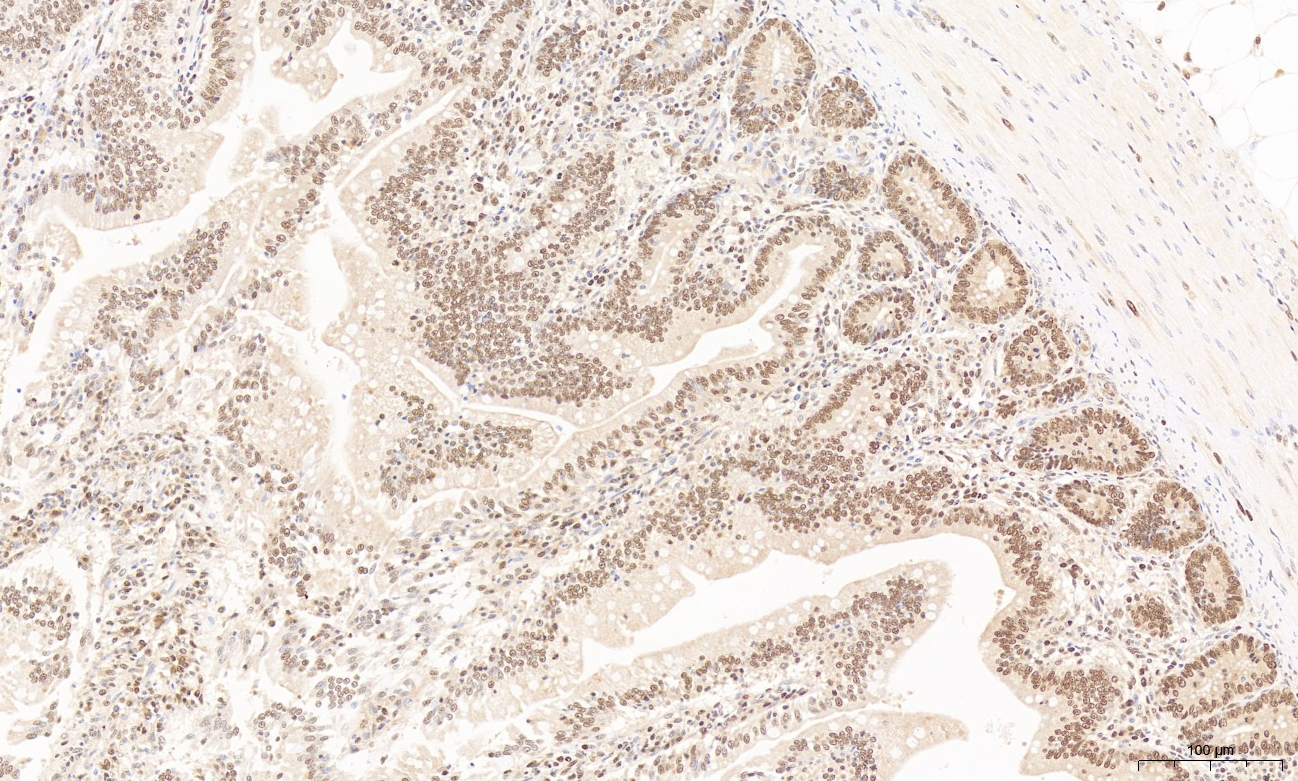

Supplement: Supplementary file 18 [file Data_Sheet_13.ZIP › Jejunal PCNA Immunohistochemical staining 2/NE+TA400 group/5.jpg]

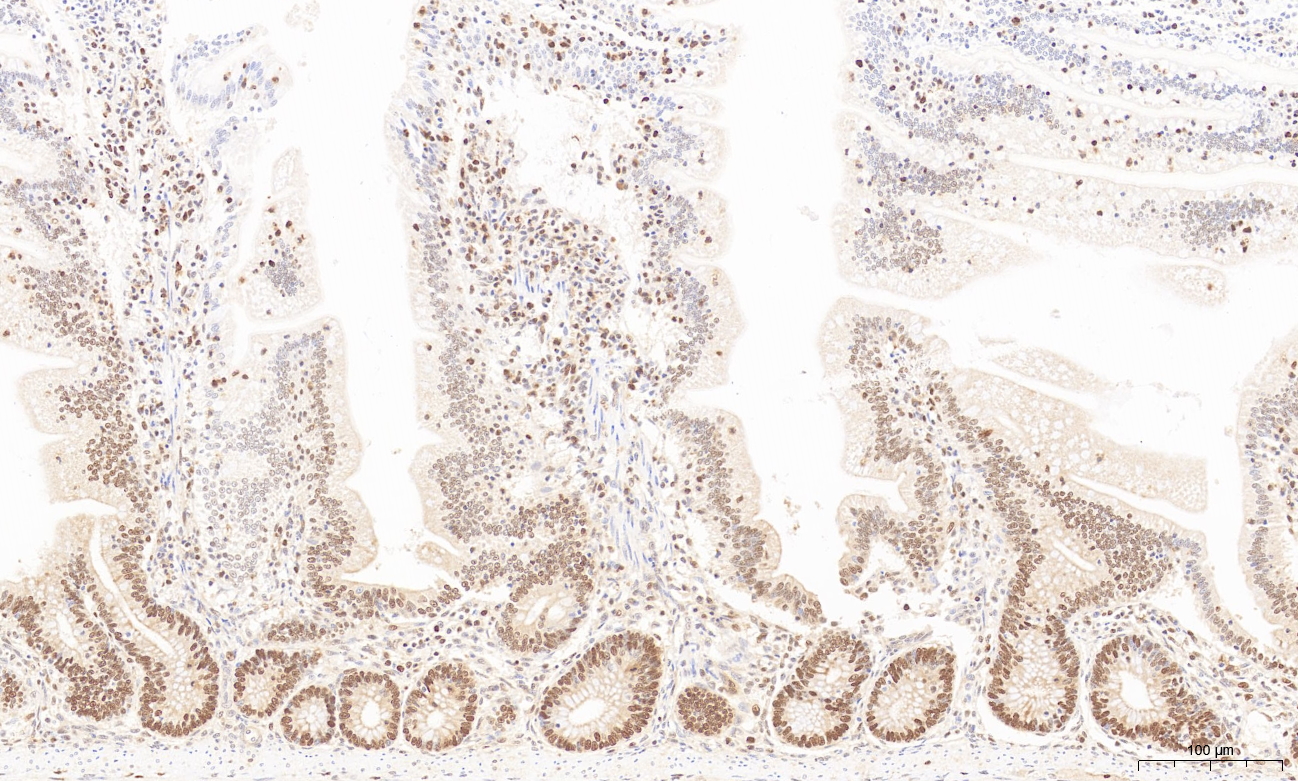

Supplement: Supplementary file 18 [file Data_Sheet_13.ZIP › Jejunal PCNA Immunohistochemical staining 2/NE+TA400 group/6.jpg]

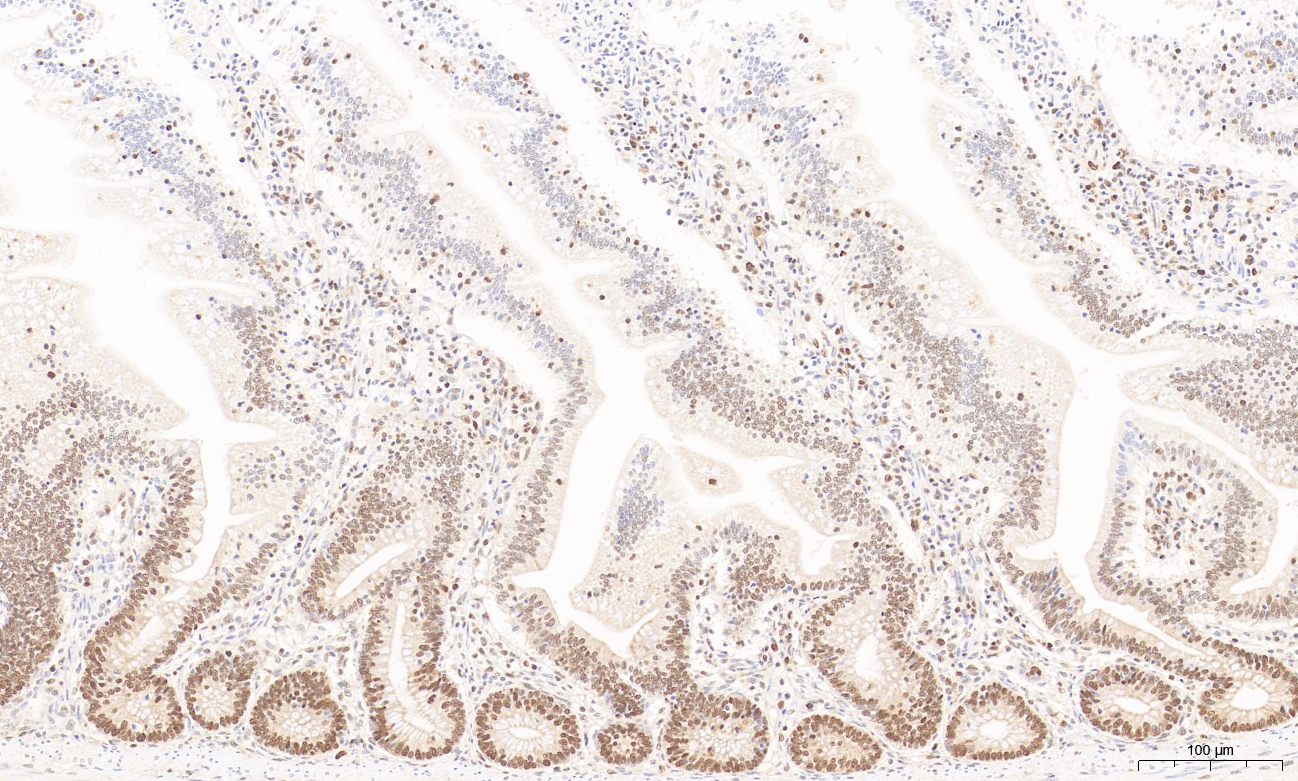

Supplement: Supplementary file 18 [file Data_Sheet_13.ZIP › Jejunal PCNA Immunohistochemical staining 2/NE+TA400 group/7.jpg]

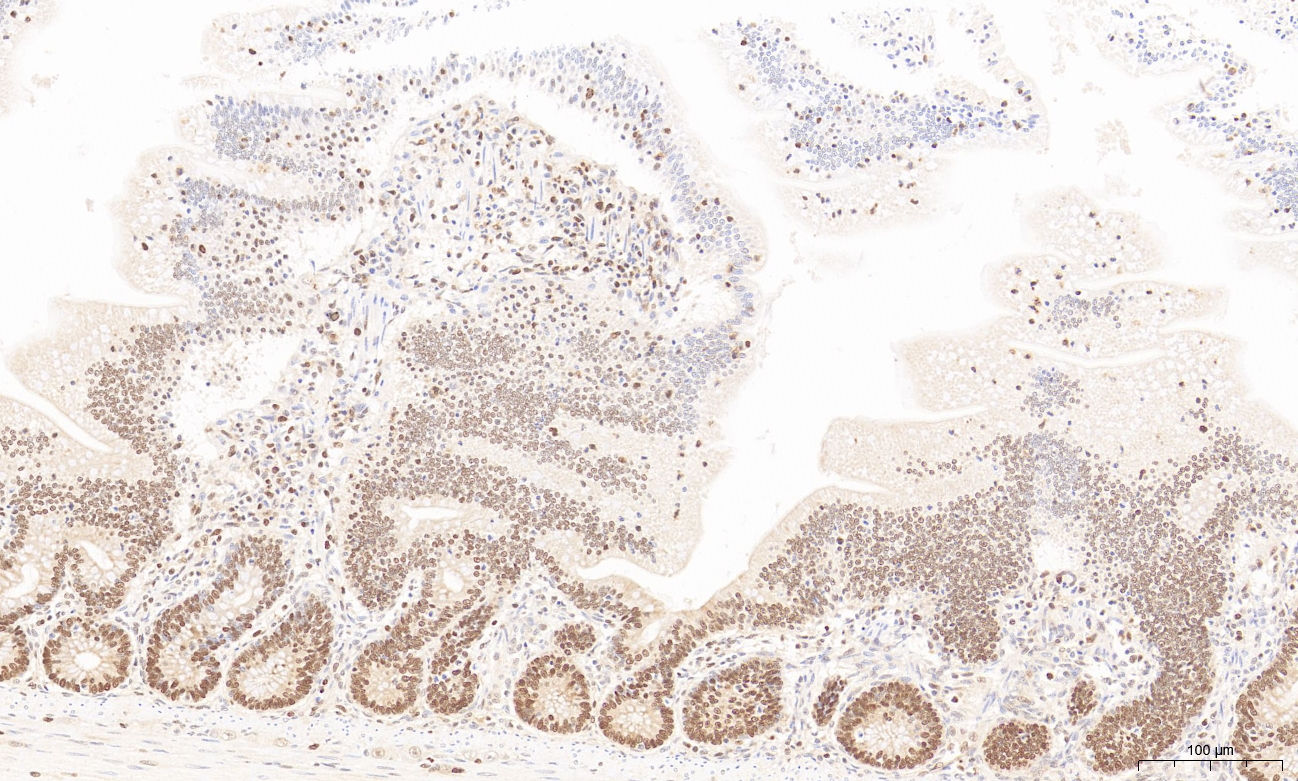

Supplement: Supplementary file 18 [file Data_Sheet_13.ZIP › Jejunal PCNA Immunohistochemical staining 2/NE+TA400 group/8.jpg]

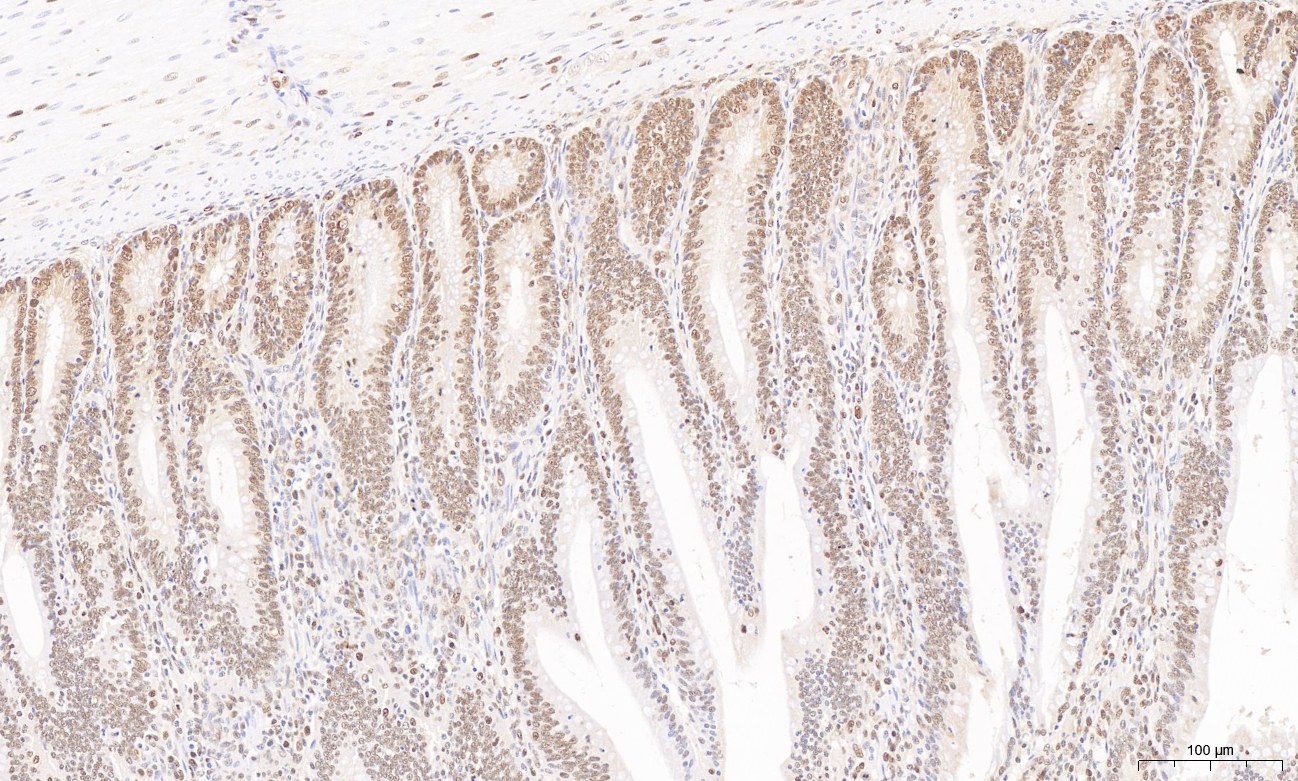

Supplement: Supplementary file 18 [file Data_Sheet_13.ZIP › Jejunal PCNA Immunohistochemical staining 2/NE+TA600 group/1.jpg]

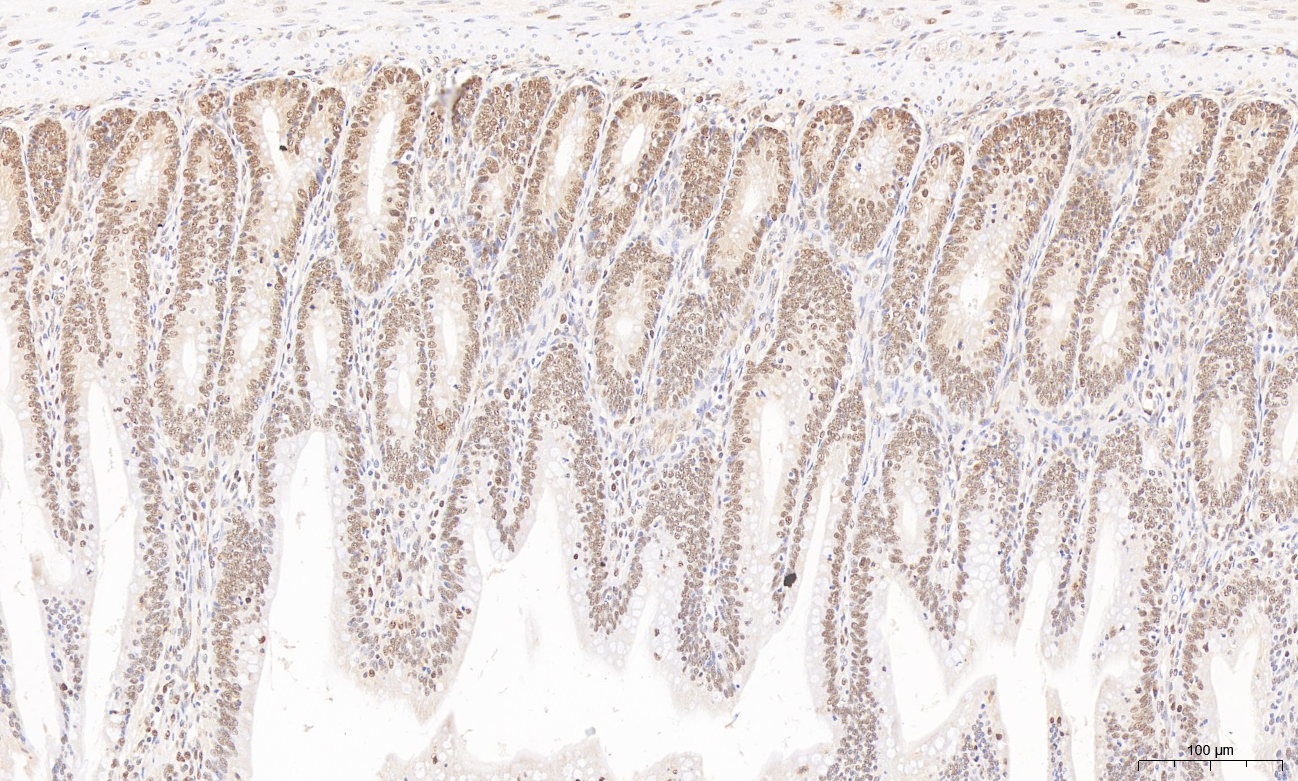

Supplement: Supplementary file 18 [file Data_Sheet_13.ZIP › Jejunal PCNA Immunohistochemical staining 2/NE+TA600 group/2.jpg]

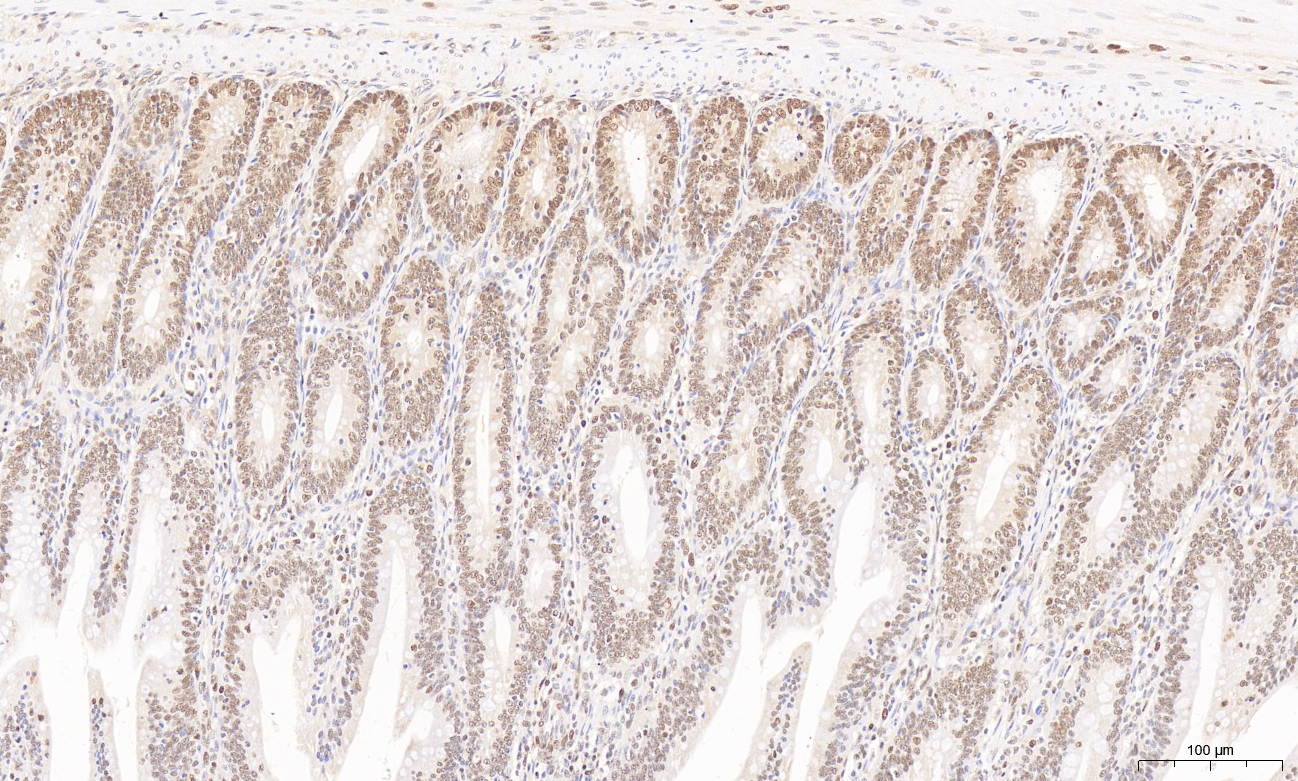

Supplement: Supplementary file 18 [file Data_Sheet_13.ZIP › Jejunal PCNA Immunohistochemical staining 2/NE+TA600 group/3.jpg]

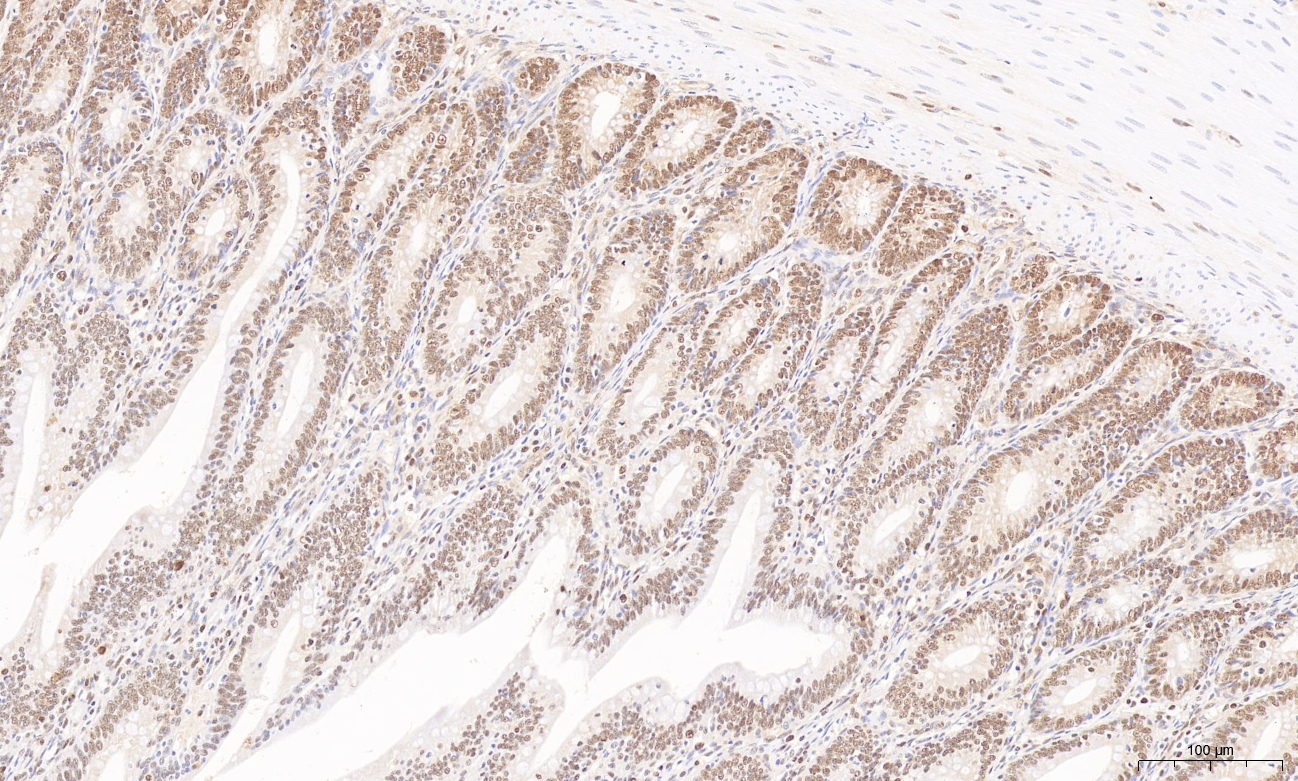

Supplement: Supplementary file 18 [file Data_Sheet_13.ZIP › Jejunal PCNA Immunohistochemical staining 2/NE+TA600 group/4.jpg]

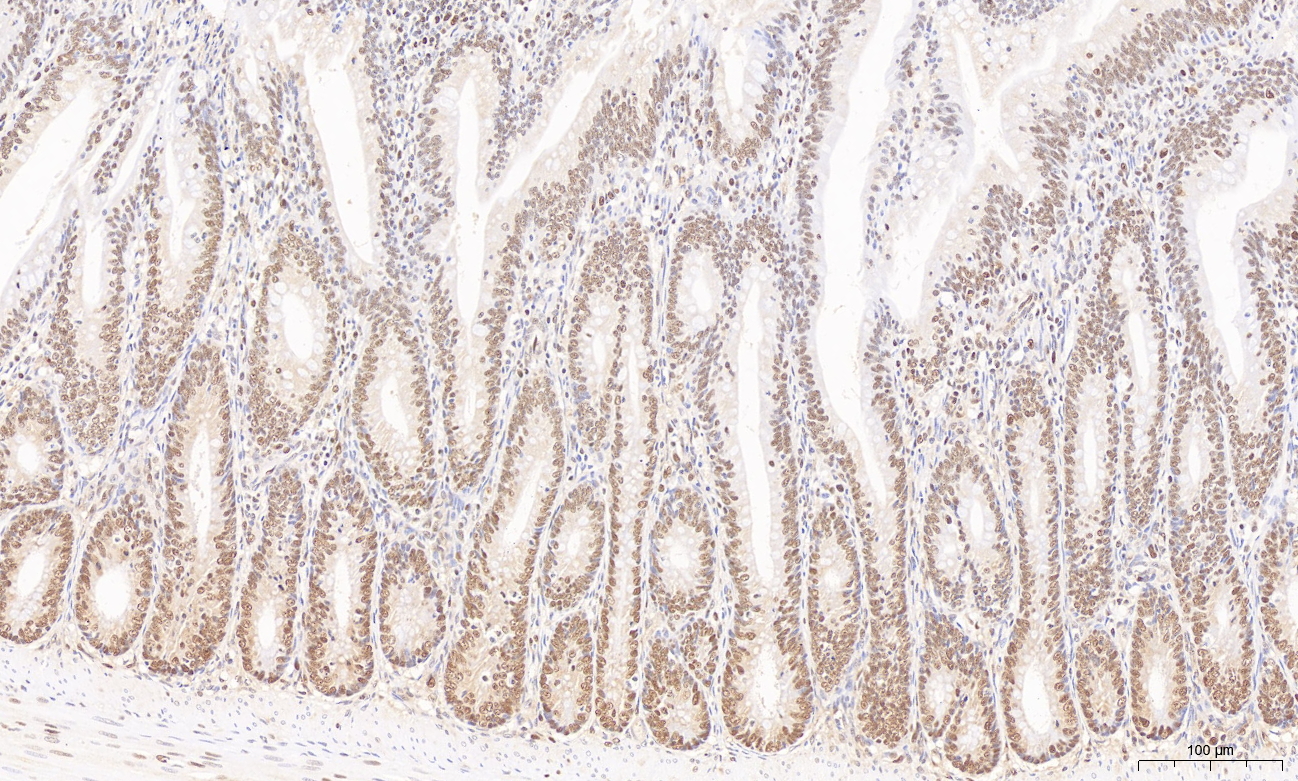

Supplement: Supplementary file 18 [file Data_Sheet_13.ZIP › Jejunal PCNA Immunohistochemical staining 2/NE+TA600 group/5.jpg]

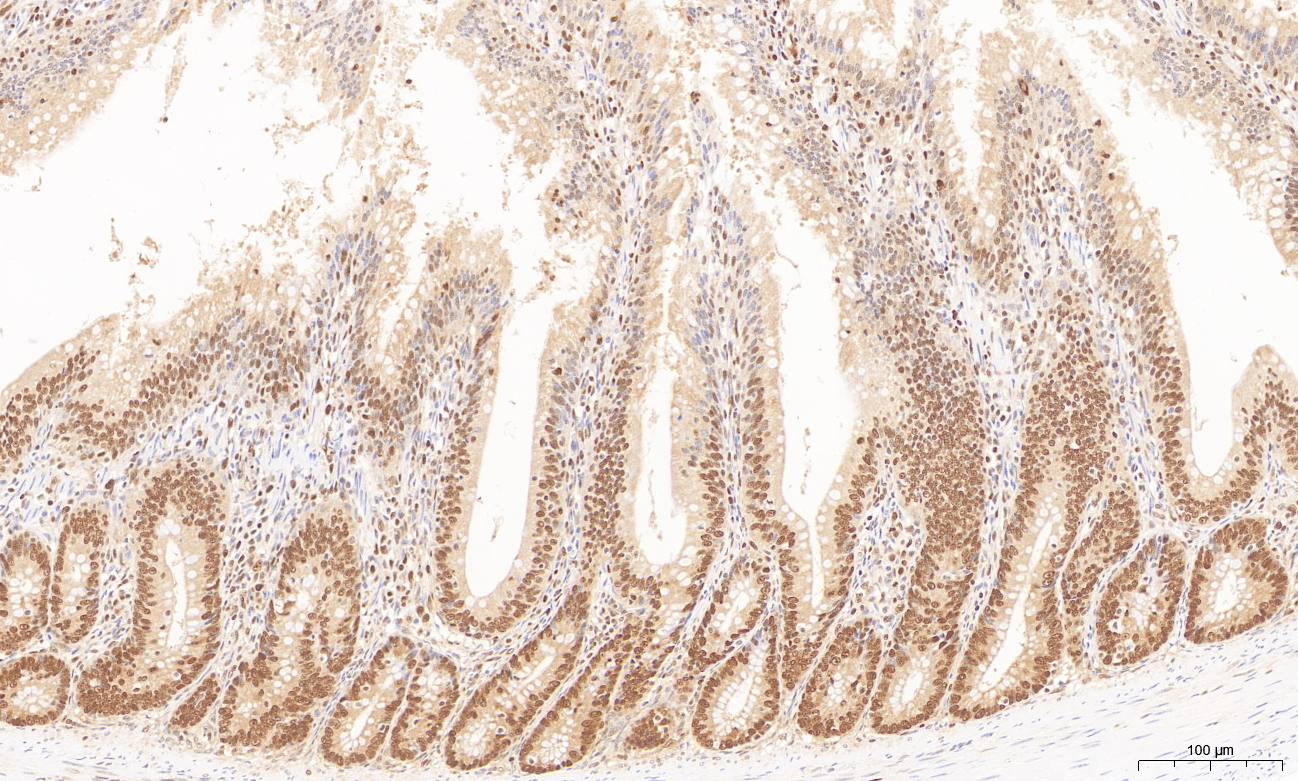

Supplement: Supplementary file 18 [file Data_Sheet_13.ZIP › Jejunal PCNA Immunohistochemical staining 2/NE+TA600 group/6.jpg]

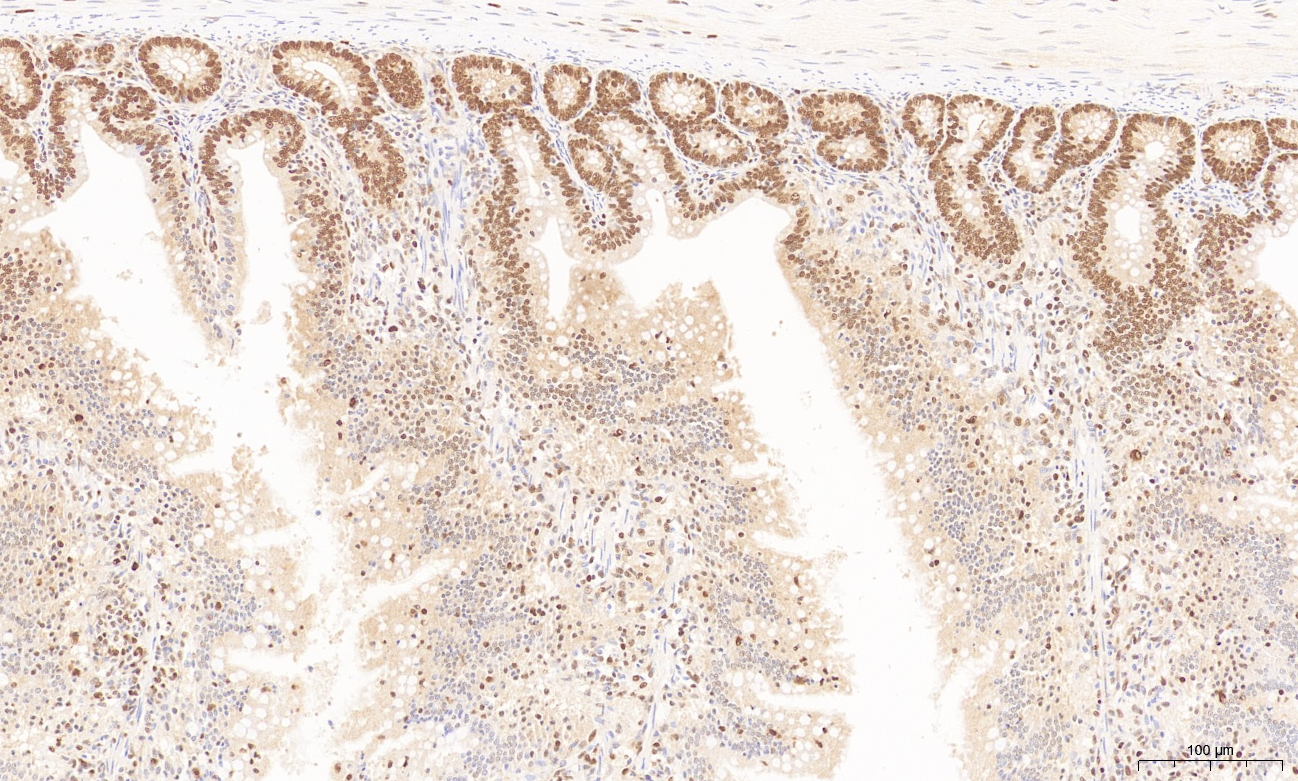

Supplement: Supplementary file 18 [file Data_Sheet_13.ZIP › Jejunal PCNA Immunohistochemical staining 2/NE+TA600 group/7.jpg]

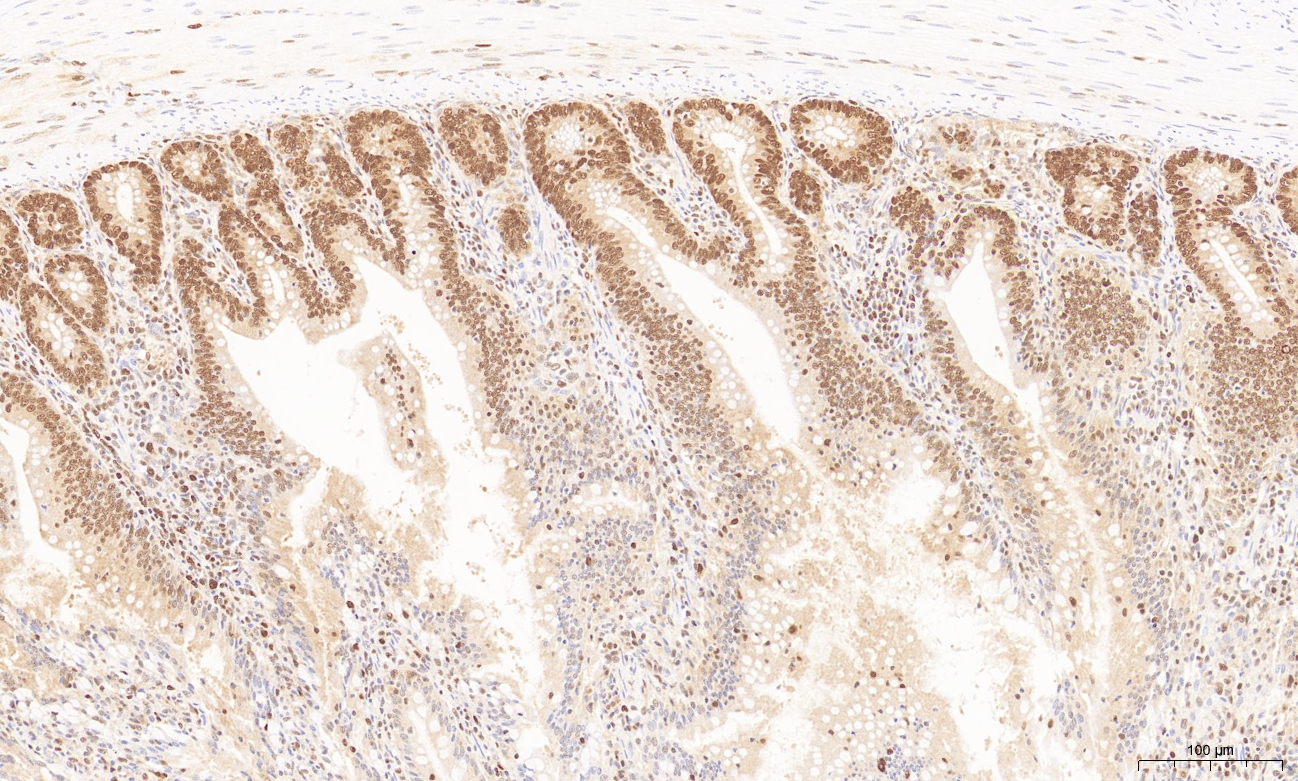

Supplement: Supplementary file 18 [file Data_Sheet_13.ZIP › Jejunal PCNA Immunohistochemical staining 2/NE+TA600 group/8.jpg]
